# Supplementary material for: EGFRvIII Promotes the Proneural–Mesenchymal Transition of Glioblastoma Multiforme and Reduces Its Sensitivity to Temozolomide by Regulating the NF-κB/ALDH1A3 Axis
Source: Genes (Basel). 2023 Mar 4;14(3):651. doi: 10.3390/genes14030651 (PMC10048499; doi:10.3390/genes14030651)
Supplement: Supplementary file 1 [file genes-14-00651-s001.zip › genes-2203856-supplementary.pdf]

gene_id	U87wt	U87vIII	FC-Value	P-Value
DNAH14	4.100084	0.000753	0.000184	0.000157
IGF2BP1	22.47238	0.005392	0.00024	0.000806
CENPV	54.32009	0.013797	0.000254	0.00076
HOOK1	8.560078	0.002182	0.000255	1.02E-06
CCND2	19.24316	0.004925	0.000256	1.41E-05
KIF1A	16.71083	0.00524	0.000314	0.000354
LOC11226	6.710218	0.002224	0.000331	0.000164
PCSK1N	45.67376	0.015194	0.000333	0.000505
PCSK6	7.049571	0.002535	0.00036	0.001166
PALD1	7.288944	0.002765	0.000379	2.47E-08
CACNA1H	4.210525	0.001691	0.000402	0.00167
CKMT1B	15.42437	0.007122	0.000462	0.002737
TP53I11	11.11984	0.005239	0.000471	0.000725
G0S2	99.54628	0.050504	0.000507	0.018538
MSI1	11.86956	0.006024	0.000508	0.001365
LGALSL	8.363392	0.004316	0.000516	6.48E-05
MAGEB2	18.44621	0.010092	0.000547	0.002236
PDZD4	6.997267	0.003832	0.000548	0.004839
RHOU	13.84107	0.007607	0.00055	0.001256
ELOVL2	4.636569	0.002723	0.000587	0.000288
MGAT4A	3.200968	0.001886	0.000589	0.000141
PALM	15.22194	0.009062	0.000595	0.000377
NEFL	13.50471	0.008362	0.000619	0.041251
SLC44A5	2.973825	0.001862	0.000626	0.005224
ZNF567	4.955524	0.003142	0.000634	0.001167
CNTN1	2.655764	0.001696	0.000639	0.003209
FAF1	60.4842	0.039121	0.000647	0.002092
MATK	16.67449	0.01087	0.000652	0.008077
VANGL2	8.14215	0.005376	0.00066	0.000183
AUTS2	2.627043	0.001804	0.000687	0.000597
ANKRD18.	2.737602	0.001901	0.000694	1.29E-05
GPR27	9.005893	0.006301	0.0007	0.007321
FAM155B	2.914914	0.002051	0.000704	7.57E-05
CCDC8	6.707579	0.004817	0.000718	0.011228
SERPINF1	80.26929	0.060709	0.000756	0.000229
SULF1	6.299597	0.00482	0.000765	0.001712
DMD	0.825593	0.000662	0.000802	6.85E-05
3-Sep	7.883351	0.006417	0.000814	0.001414
CHDH	2.314059	0.0019	0.000821	0.009345
GATA4	3.661128	0.003061	0.000836	2.89E-05
CA8	5.940604	0.004996	0.000841	0.012415
ZCCHC12	8.268046	0.007034	0.000851	0.020151
CHST15	1.866563	0.001589	0.000851	2.31E-07
SLC7A3	17.25093	0.014764	0.000856	0.004557
LRP2	2.423569	0.002108	0.00087	0.009135
GPC3	35.4997	0.031929	0.000899	0.025672
SHANK3	4.998616	0.004616	0.000923	0.000694
SPTBN2	3.162747	0.003083	0.000975	0.000915
NYNRIN	1.861937	0.001817	0.000976	0.000771
BARX2	8.600893	0.008395	0.000976	7.16E-05
RIMS4	9.325683	0.009247	0.000992	0.000189
CCDC120	3.758362	0.00374	0.000995	0.00014
ABCG1	1.376174	0.0014	0.001017	0.003344
MAPK15	10.30498	0.0105	0.001019	1.25E-05
CDIP1	21.06031	0.02248	0.001067	0.00283
ZSCAN18	9.307425	0.00999	0.001073	7.81E-05
MIPOL1	0.584824	0.000644	0.001102	0.000459

HOXB8	7.320244	0.008071	0.001102	0.000665
WSCD1	3.110153	0.003476	0.001117	3.00E-05
CAMKV	4.179575	0.004677	0.001119	0.011104
N4BP3	1.350068	0.00152	0.001126	0.002201
ADD2	9.037891	0.010193	0.001128	2.54E-05
ZNF483	2.38015	0.002687	0.001129	0.011093
VWDE	1.784791	0.002017	0.00113	4.61E-05
MNX1	10.58044	0.012287	0.001161	0.002012
ZNF569	2.964656	0.003452	0.001165	0.004305
LAMC3	2.277374	0.00266	0.001168	0.011546
CECR2	4.089351	0.004806	0.001175	0.002027
PPP1R14A	18.05009	0.021548	0.001194	0.003012
EMILIN3	3.509942	0.004196	0.001196	0.002151
CNTFR	4.151897	0.004978	0.001199	0.000862
CALCB	143.3127	0.174658	0.001219	0.023922
PPP2R2C	2.200717	0.002719	0.001236	0.006253
LPAR3	6.114641	0.007601	0.001243	0.010012
BAIAP2L1	25.06588	0.031439	0.001254	0.000122
MGAT3	1.466929	0.001881	0.001283	2.70E-05
PRDM16	1.249364	0.001665	0.001333	0.000845
LOC10537	5.62338	0.007514	0.001336	5.30E-06
SYK	2.205718	0.00297	0.001346	0.009378
GAREM2	3.408941	0.004605	0.001351	0.00018
PRICKLE1	2.297644	0.003129	0.001362	0.000579
TTC9	2.302315	0.003209	0.001394	0.000194
PLEKHG1	1.45907	0.002051	0.001406	0.000639
MSX2	10.21298	0.014379	0.001408	0.000831
CNTNAP2	3.310386	0.004722	0.001426	0.018383
CA2	83.28906	0.119231	0.001432	0.056085
RAB6B	11.81819	0.017465	0.001478	0.000966
TUBB4A	11.84548	0.017951	0.001515	0.002728
AMOT	49.31786	0.074849	0.001518	0.017711
GPR50	3.086823	0.00471	0.001526	0.028344
SORL1	1.668885	0.00255	0.001528	0.000196
2-Mar	9.01474	0.014194	0.001575	5.29E-05
COL26A1	2.88265	0.004623	0.001604	0.00303
FLT4	7.947511	0.012799	0.00161	0.000219
KREMEN2	4.919677	0.008029	0.001632	0.011951
APBA2	3.024361	0.004957	0.001639	0.001819
FAM69B	16.85168	0.027746	0.001647	0.000328
RGMA	9.958472	0.016454	0.001652	0.002147
LRRC61	11.30992	0.019119	0.00169	0.002787
SH3GL3	4.094419	0.006934	0.001693	0.000766
HRK	6.296892	0.010666	0.001694	0.00741
GRID1	1.613597	0.002743	0.0017	0.009131
USH1C	2.0245	0.003503	0.00173	0.006597
KLRG2	2.984256	0.005182	0.001737	0.073777
HOXB13	3.160216	0.005511	0.001744	0.066054
HOXC13	7.794937	0.013691	0.001756	0.000547
ZNF529	2.816026	0.004988	0.001771	0.000802
TMEM74B	3.677424	0.006599	0.001795	0.000378
JUP	22.89203	0.041178	0.001799	1.22E-06
NELL2	6.115549	0.011034	0.001804	0.02467
HOXC11	4.369225	0.007884	0.001804	0.000568
HEYL	2.126659	0.003848	0.00181	0.002068
GPR160	8.166819	0.014973	0.001833	0.012722
MOB3B	1.332062	0.002461	0.001848	0.002014
SLC46A1	2.71314	0.005023	0.001852	6.92E-05

NAT8L	7.572757	0.014104	0.001862	9.72E-05
FRMD4B	1.787051	0.003343	0.001871	0.000472
SELENOP	11.15702	0.020924	0.001875	0.001646
B4GALNT3	4.968546	0.009342	0.00188	0.000469
ZIC5	3.734513	0.007022	0.00188	0.002527
EBF4	14.26166	0.026881	0.001885	9.45E-05
WNT11	3.348557	0.006338	0.001893	0.001503
IFITM1	47.03666	0.0915	0.001945	0.004678
EFHD1	19.30376	0.037777	0.001957	9.99E-05
NEIL1	4.12869	0.008091	0.00196	0.001375
KLHL9	11.58212	0.022732	0.001963	0.0054
OLFM2	10.77772	0.021411	0.001987	0.001448
FAT3	0.586682	0.00118	0.002011	0.003448
SLIT2	26.06321	0.052899	0.00203	0.00328
EPHA6	0.690676	0.001406	0.002036	0.003953
NSUN5	12.08363	0.024998	0.002069	0.000657
HAPLN2	2.417504	0.005014	0.002074	0.004254
TNFRSF13	8.524923	0.017683	0.002074	0.00188
LRCH2	15.05411	0.031666	0.002103	0.001052
PCDH10	5.397143	0.011357	0.002104	0.001183
EXD2	7.289388	0.015347	0.002105	0.000173
KCNH3	1.70319	0.003644	0.002139	0.002632
OSTN	4.49474	0.009676	0.002153	0.026066
TDRP	1.701157	0.003692	0.00217	0.001443
INAVA	1.382895	0.003003	0.002172	2.26E-05
CSAG3	4.025065	0.008748	0.002173	0.000802
FUT3	2.669871	0.005893	0.002207	0.121404
DSP	13.17845	0.030115	0.002285	0.00301
CAND2	7.044659	0.016126	0.002289	0.000149
NEFM	94.31531	0.217878	0.00231	0.047375
HOXA10	24.42554	0.057459	0.002352	0.000433
P2RX2	2.42654	0.00584	0.002407	0.00662
SLC16A9	1.466727	0.003545	0.002417	0.006331
PPP1R16B	1.822671	0.004412	0.002421	0.000297
CDO1	8.191641	0.019904	0.00243	0.004159
ZFP30	2.977085	0.007237	0.002431	0.000702
TMEM63C	2.434348	0.005946	0.002442	0.00175
EGFL7	12.39534	0.030292	0.002444	0.019561
SEMA3F	4.163965	0.010209	0.002452	0.004812
FOXN4	2.033791	0.004994	0.002455	0.010208
EMB	7.567022	0.018681	0.002469	0.000739
ZNF256	3.027216	0.007473	0.002469	1.33E-05
MPPED2	0.745454	0.001845	0.002475	0.000209
LRRC4B	3.546784	0.008852	0.002496	0.000519
KCNK1	4.557486	0.01143	0.002508	0.000855
TESMIN	2.172544	0.005515	0.002539	1.09E-07
SELENBP1	11.98273	0.030563	0.002551	0.001072
TJP3	1.886382	0.004816	0.002553	0.03313
ROR1	1.289414	0.003309	0.002566	0.000427
SIM2	3.923432	0.010072	0.002567	0.000221
ZNF506	1.929933	0.004969	0.002575	0.000215
CYP26A1	2.494226	0.006478	0.002597	0.001763
MAPK4	0.445297	0.001173	0.002635	0.002766
STXBP2	16.08364	0.042707	0.002655	0.000175
RCAN2	2.7394	0.007453	0.002721	0.00039
MERTK	6.282218	0.017134	0.002727	0.002567
SYCP2L	5.781065	0.015975	0.002763	0.00016
LIN28B	10.24605	0.028387	0.00277	0.003427

WNK2	2.898917	0.008039	0.002773	0.002827
FNDC10	5.654499	0.015921	0.002816	0.068359
EPHX2	3.190997	0.008991	0.002818	0.000254
HES4	92.288	0.264114	0.002862	0.013131
MKX	1.23532	0.003561	0.002883	0.002348
PRKCQ	1.067921	0.003112	0.002914	0.017324
SCN5A	0.624996	0.001868	0.002989	0.000612
SCN4B	1.114551	0.003333	0.00299	0.009094
NTN1	8.022733	0.024113	0.003006	7.51E-07
FOCAD	7.911964	0.023889	0.003019	8.63E-05
GLS2	1.955494	0.005925	0.00303	0.008825
SLC38A3	3.512908	0.010677	0.003039	0.00077
IL17RB	4.172218	0.012737	0.003053	0.000113
ZNF568	1.169268	0.003579	0.003061	9.90E-06
SBK1	7.216321	0.022287	0.003088	0.002703
MEGF11	0.709014	0.002197	0.003099	0.004289
HS6ST2	3.690958	0.01144	0.0031	0.007699
HOXB9	48.80211	0.151432	0.003103	0.000997
JAG2	11.71492	0.036405	0.003108	2.86E-05
TMEM35A	2.562724	0.007975	0.003112	0.062865
RASGEF1A	4.431892	0.013796	0.003113	0.00491
CERS4	6.23603	0.019473	0.003123	0.00099
MAFA	2.1289	0.006768	0.003179	0.005371
EPHA7	3.208643	0.010205	0.00318	0.013377
USP2	4.893047	0.015607	0.00319	0.000213
TPD52L1	13.8064	0.044082	0.003193	0.005998
TMEM59L	31.02996	0.100603	0.003242	0.000971
TM6SF1	0.786551	0.002554	0.003247	0.000112
HPCAL4	3.007207	0.009771	0.003249	0.004704
ZNF501	3.003831	0.009901	0.003296	0.033066
C1orf115	15.07306	0.04986	0.003308	0.000331
GRIN2D	1.886004	0.006241	0.003309	4.87E-05
EPHA8	0.811628	0.002693	0.003318	0.057419
MYCN	1.646799	0.005511	0.003347	4.55E-06
PLEKHA7	1.462776	0.004911	0.003357	0.001195
STUM	1.892564	0.006356	0.003359	0.001874
ZNF260	5.269404	0.017789	0.003376	0.000311
CACNA1B	0.478768	0.00164	0.003426	0.000178
COL25A1	0.990253	0.003424	0.003458	0.002145
CFAP221	0.692168	0.002412	0.003484	0.000681
ZNF420	2.598508	0.009065	0.003489	0.002849
ZNF695	3.741794	0.013131	0.003509	0.001353
PRR5	3.046361	0.010698	0.003512	0.016047
FOXF2	17.01829	0.060101	0.003532	0.001569
LPL	1.231563	0.004408	0.003579	1.03E-05
ADCY1	2.844318	0.010193	0.003584	2.56E-05
SPSB4	0.841911	0.00303	0.003599	0.001444
NAALADL	1.4462	0.005208	0.003601	0.147247
ZNF429	0.535906	0.001942	0.003624	0.055907
OGDHL	4.317015	0.015661	0.003628	0.019671
ELFN1	0.460648	0.001671	0.003629	0.001376
ART5	4.575936	0.016642	0.003637	0.013359
FGFR4	14.68882	0.053826	0.003664	0.002934
SPINT2	29.9732	0.110107	0.003674	0.000707
TSPAN18	6.574432	0.024216	0.003683	1.55E-06
AGAP11	8.58684	0.031697	0.003691	0.007829
FGF9	1.784996	0.006596	0.003695	0.000806
BCL6B	1.224447	0.004556	0.003721	0.007396

RDH16	1.245335	0.004636	0.003723	0.027814
NEBL	1.500085	0.005596	0.003731	0.00692
TTC12	1.580979	0.005912	0.003739	0.037152
FGFR3	9.799909	0.036802	0.003755	0.000304
COL14A1	7.353855	0.027685	0.003765	0.000222
GDF7	2.185534	0.008238	0.003769	5.53E-05
ME1	8.737107	0.032982	0.003775	0.00051
ASXL3	0.395072	0.001514	0.003832	0.002081
POU3F2	4.235226	0.016276	0.003843	0.024197
LRP5	13.29031	0.0512	0.003852	1.35E-05
KCNQ2	1.956461	0.007559	0.003864	0.011936
SMIM10L2	2.359599	0.009194	0.003897	0.000248
MAP3K15	3.447382	0.013459	0.003904	4.96E-05
ABLIM1	4.10202	0.016239	0.003959	0.001195
CMTM8	5.84161	0.023383	0.004003	0.001616
SDK1	1.804885	0.007225	0.004003	0.004189
SLCO2A1	0.835846	0.003358	0.004017	0.009704
FXYD6	8.600991	0.034636	0.004027	0.002731
EEF1A2	200.5368	0.808659	0.004032	0.000387
SCN4A	0.506941	0.002058	0.004059	0.026099
ZNF704	2.036112	0.008385	0.004118	0.003199
RBM20	3.187825	0.013155	0.004127	0.0004
FAM241B	7.834647	0.032351	0.004129	0.000234
KLHL41	4.907896	0.020293	0.004135	0.00574
PTER	6.713465	0.027805	0.004142	0.007649
SULT2B1	8.581442	0.035589	0.004147	0.000283
ZNF582	1.27083	0.00528	0.004155	0.004484
SEMA6A	5.210834	0.021709	0.004166	0.001158
SMTNL2	2.470072	0.010331	0.004183	0.004557
CARMIL3	1.663288	0.006982	0.004198	0.000171
SFMBT2	4.988308	0.020944	0.004199	0.000444
MPZL2	1.190811	0.005038	0.00423	0.018254
MAP1LC3	4.894085	0.020854	0.004261	0.002417
GPX3	10.47363	0.044643	0.004262	0.000739
ZNF570	1.312727	0.005616	0.004278	0.003732
ADGRL3	0.871475	0.003735	0.004286	0.007544
SRCIN1	1.436855	0.006181	0.004302	4.08E-05
PREX2	0.323487	0.001397	0.004318	0.036719
ZNF471	0.403007	0.001748	0.004339	0.006022
ZBTB8B	1.919083	0.008332	0.004342	0.004404
RASGEF1E	4.590523	0.020018	0.004361	0.000518
HOXD10	31.35869	0.136946	0.004367	0.005928
SULF2	6.363687	0.027824	0.004372	0.003507
SHISA2	5.130043	0.022472	0.004381	0.035557
SHISA8	2.634931	0.011546	0.004382	0.002987
ITIH6	1.474472	0.006649	0.004509	0.08963
CXCR4	9.287961	0.042174	0.004541	0.001926
PODXL2	32.85886	0.149325	0.004544	0.002714
PDE3B	0.854155	0.003886	0.004549	0.007709
CDHR1	1.343835	0.006124	0.004557	0.001566
GNG4	12.60249	0.057434	0.004557	0.001537
PDIA2	6.183696	0.028392	0.004591	0.054962
HHIP	1.264648	0.005807	0.004592	0.000738
KIF26A	5.194011	0.023869	0.004595	0.000333
ROBO2	1.354907	0.006282	0.004637	0.002662
PHF21B	8.32171	0.038891	0.004673	4.89E-05
ID4	73.26732	0.342908	0.00468	0.001379
ZDHHC22	1.30507	0.00611	0.004682	0.009929

SALL2	6.082901	0.028584	0.004699	0.000894
ZNF816	1.258681	0.005991	0.004759	0.005987
CFD	5.872208	0.028052	0.004777	0.084429
SOX4	65.67539	0.316604	0.004821	0.012864
ZNF239	0.809996	0.003907	0.004824	0.029896
KCNJ8	5.06241	0.024822	0.004903	0.000206
MYH10	29.62172	0.145401	0.004909	0.001242
FZD9	1.43643	0.007052	0.00491	0.002351
ETNK2	12.26532	0.062263	0.005076	0.000162
B4GALNT4	8.667117	0.044048	0.005082	0.001351
LDHC	4.855405	0.02486	0.00512	0.032258
PNMA6A	1.429622	0.00735	0.005141	0.00028
COBL	2.747064	0.014181	0.005162	0.000157
CKB	550.4856	2.853136	0.005183	0.000879
MBNL3	1.051274	0.005479	0.005212	0.00918
SEMA6B	10.22656	0.053771	0.005258	0.002653
MGST2	5.072944	0.026804	0.005284	0.015327
ADGRG2	0.535428	0.00283	0.005285	0.004558
ACHE	1.331805	0.007059	0.0053	0.001574
RNF150	3.410151	0.018083	0.005303	3.04E-06
ABCC8	0.951866	0.005072	0.005328	0.001433
NUDT10	7.398529	0.039467	0.005334	0.015126
MRAP2	0.692667	0.003696	0.005336	0.110106
SYNGR1	15.04067	0.080327	0.005341	0.000266
TGIF2	12.66722	0.06773	0.005347	0.000216
FAM189A2	0.893547	0.004792	0.005363	0.013171
KCTD15	6.238213	0.033474	0.005366	0.002159
TF	5.522771	0.029699	0.005378	0.007996
ERICH5	5.820673	0.031378	0.005391	0.001869
MLLT3	5.247315	0.0283	0.005393	0.00189
RPRML	2.822273	0.015318	0.005428	0.101564
MYH14	5.431243	0.029582	0.005447	0.000403
KCNQ1	0.830865	0.004579	0.005512	0.000256
MYO15B	0.881467	0.004876	0.005531	0.001122
TUSC3	10.45184	0.057882	0.005538	3.93E-05
PLCL1	0.39268	0.002185	0.005565	0.00079
CUX2	0.583301	0.003247	0.005567	0.003479
PAX9	2.836426	0.015947	0.005622	7.55E-05
RGS16	25.13323	0.142328	0.005663	0.004473
ZFP28	0.824518	0.004681	0.005677	0.000326
A2M	6.41779	0.036466	0.005682	0.00128
ZNF331	3.338414	0.01897	0.005682	0.006999
RHOV	1.652696	0.009419	0.005699	0.01661
CALY	1.09442	0.006292	0.005749	0.000132
LRG1	1.567746	0.009022	0.005755	0.010978
SP9	1.910675	0.011038	0.005777	0.029976
JPH3	4.260975	0.024637	0.005782	0.000751
SH3RF3	1.302102	0.007529	0.005782	0.003836
PANX2	3.419048	0.019789	0.005788	0.001284
GUCY2D	0.430244	0.00249	0.005788	0.000266
SNX10	6.827028	0.03996	0.005853	0.002498
LCN2	52.5642	0.308408	0.005867	0.044092
RBPMS2	8.343678	0.049066	0.005881	0.000708
SYCP2	1.247841	0.007366	0.005903	0.00082
ICA1	4.31395	0.025574	0.005928	0.001034
FAM83H	1.431539	0.008487	0.005928	0.017404
SLC40A1	0.771521	0.004576	0.005932	0.06234
C2CD4C	3.119144	0.018769	0.006017	0.005415

TMC6	3.202626	0.01928	0.00602	0.00246
ZNF566	2.887989	0.017442	0.00604	1.15E-05
WT1	0.796547	0.004839	0.006075	0.009654
SLC13A3	3.155113	0.019283	0.006112	0.0071
ADAP1	5.70508	0.034958	0.006128	0.002179
ANKRD6	2.658253	0.016354	0.006152	2.36E-06
ZNF605	2.970102	0.018436	0.006207	0.000564
CEBPA	4.981344	0.031213	0.006266	0.009586
SSPO	0.512616	0.003221	0.006284	0.000283
SUSD5	3.824641	0.024069	0.006293	0.005279
SCUBE1	1.781128	0.011307	0.006348	0.006971
HFM1	0.461733	0.002959	0.006408	0.002341
DMBX1	3.142833	0.020157	0.006413	0.005993
CGA	9.025689	0.057894	0.006414	0.068852
PDE1B	0.534537	0.00343	0.006417	0.02812
BRSK2	10.49627	0.067391	0.006421	7.60E-06
KCNJ4	3.504812	0.022694	0.006475	0.017898
ESPN	0.305809	0.001983	0.006484	0.000924
KIF25	0.637862	0.004143	0.006494	2.55E-05
GRID2IP	0.523906	0.003421	0.00653	0.007053
CHFR	6.725836	0.044189	0.00657	0.000114
DLL1	5.235831	0.034451	0.00658	0.000735
RASAL1	0.390974	0.00259	0.006624	0.003012
TCF7L1	5.913847	0.039189	0.006627	0.000154
TMEM269	1.485325	0.009849	0.006631	0.03813
LPIN3	3.154382	0.021058	0.006676	0.000421
FAM83B	0.31667	0.002114	0.006676	0.010117
COL4A1	3.480101	0.023276	0.006688	7.57E-05
NNT	8.603286	0.057599	0.006695	0.000459
MTAP	11.07477	0.074282	0.006707	6.08E-05
SCARA5	4.059097	0.027247	0.006713	0.027901
MUC1	1.329623	0.008986	0.006758	8.02E-06
FGF19	1.104494	0.007515	0.006804	0.012489
VASH2	1.068042	0.007292	0.006827	0.000997
CHRD	4.01441	0.027414	0.006829	2.33E-05
SMPDL3B	3.462718	0.023752	0.006859	0.00489
ALDH1A2	17.37126	0.119339	0.00687	0.002113
OTX2	0.911156	0.006303	0.006917	0.024981
ZNF607	3.707556	0.025661	0.006921	0.000369
CHRNA7	0.329711	0.002315	0.007023	0.001036
TEX15	0.33941	0.002407	0.007092	0.038808
TMEM179	0.227525	0.001618	0.00711	0.162935
GFRA1	1.363091	0.009713	0.007126	0.001206
VWA5B2	0.791754	0.005703	0.007203	0.000887
HOXA6	5.65874	0.040901	0.007228	0.000793
CLUL1	0.822048	0.005959	0.007249	0.012785
FBN3	0.221192	0.001608	0.007271	0.000191
HID1	8.026297	0.058449	0.007282	0.003183
TP73	1.629722	0.011881	0.00729	2.44E-05
CPVL	16.37256	0.119585	0.007304	0.002832
HOXC10	15.10926	0.110588	0.007319	0.000133
STRC	0.414169	0.003036	0.00733	0.118896
NFATC1	3.170336	0.023276	0.007342	6.06E-06
VAV1	3.003785	0.022105	0.007359	0.000629
CERS1	1.793025	0.013206	0.007365	0.00143
GNG7	3.464422	0.025723	0.007425	8.54E-05
TINAGL1	4.670141	0.034764	0.007444	0.003022
BMP7	13.50675	0.100732	0.007458	0.000392

TMEM130	0.719905	0.005389	0.007485	0.019519
INHBB	0.687342	0.005153	0.007497	0.034546
ZNF493	1.37543	0.010475	0.007616	0.003712
TRMT10B	2.080113	0.015952	0.007669	8.42E-07
SLC6A1	0.286559	0.002199	0.007675	0.02766
WNT9A	1.365425	0.010563	0.007736	0.0012
NKX2-3	0.878873	0.006848	0.007792	0.00435
RGS7	0.328912	0.002566	0.0078	0.01661
REEP1	5.373113	0.04248	0.007906	7.04E-05
RAMP2	5.222327	0.041498	0.007946	0.001233
CRLF1	4.861517	0.038652	0.007951	0.041209
HAP1	4.094133	0.032633	0.007971	0.006965
RHPN1	7.287936	0.058413	0.008015	0.000775
NPPC	1.40063	0.011226	0.008015	0.000154
ARHGEF4	1.314555	0.010581	0.008049	0.000652
PRSS50	1.542776	0.01243	0.008057	0.067409
ZNF28	1.193078	0.009735	0.008159	0.042121
ASRGL1	12.57693	0.103011	0.00819	0.000239
GFI1	2.527197	0.020709	0.008194	0.007731
ZNF736	3.390283	0.027953	0.008245	0.00018
EPB41L4A	0.959293	0.008089	0.008433	0.002177
CCDC88C	2.057234	0.017437	0.008476	0.002167
OBSCN	1.042348	0.008852	0.008493	0.000193
DUSP9	17.36201	0.147835	0.008515	0.046195
CHRNA4	0.61123	0.005206	0.008517	0.012307
APOC1	18.74687	0.160271	0.008549	0.000168
SHANK2	0.25344	0.002172	0.008571	8.33E-07
TEKT3	0.337027	0.002889	0.008572	0.003192
COL18A1	13.25523	0.11472	0.008655	0.004994
CES3	4.112689	0.0356	0.008656	0.000295
ENO4	0.195783	0.001703	0.008698	0.038054
PDGFB	0.476977	0.004165	0.008732	0.002494
ZNF737	0.438163	0.003829	0.008738	0.018926
HOXC9	7.380152	0.064614	0.008755	0.00011
SPON1	3.266171	0.028708	0.00879	0.010781
FAM198A	1.624216	0.014432	0.008886	0.00134
GALC	1.141424	0.010143	0.008886	0.002451
CR1L	1.010492	0.009114	0.00902	0.005839
CHST8	1.343623	0.012191	0.009073	0.006487
PGGHG	13.92712	0.126839	0.009107	0.000465
TMEM266	3.295991	0.030072	0.009124	0.015561
PLIN2	18.6991	0.172632	0.009232	0.000331
NOL4	0.73543	0.006803	0.009251	0.002395
PACRG	0.123918	0.001152	0.009297	0.001833
RAB11FIP1	9.494654	0.088368	0.009307	0.000304
PPM1H	3.791731	0.035369	0.009328	0.001564
ZNF829	1.307445	0.012279	0.009392	0.019037
HOXB5	3.806145	0.035903	0.009433	0.003236
RASSF2	2.378966	0.022588	0.009495	0.000421
ZNF709	1.063839	0.010113	0.009507	2.79E-05
GOLGA8T	0.57047	0.005444	0.009542	0.004511
CD207	0.559554	0.005375	0.009605	0.003325
PPM1E	2.850065	0.027384	0.009608	0.006121
CADM2	0.330214	0.003185	0.009646	0.008543
S100P	6.79718	0.065659	0.00966	0.06396
CACNA2D	3.754579	0.036335	0.009678	0.004369
IGSF11	0.360765	0.00352	0.009756	0.003633
CBS	8.365413	0.081798	0.009778	0.000537

SLC30A3	4.099568	0.040155	0.009795	0.000605
LINGO3	0.799189	0.007861	0.009836	0.028561
UNC5CL	3.173753	0.031242	0.009844	0.0192
ZNF467	3.399132	0.03354	0.009867	5.38E-05
CABLES1	3.155929	0.03117	0.009877	0.003063
KRT18	48.68062	0.481204	0.009885	2.58E-05
SYT17	2.079094	0.020589	0.009903	8.71E-05
FRMPD1	0.184157	0.001832	0.009951	0.023042
ONECUT2	4.708108	0.046869	0.009955	0.00264
NELL1	0.479314	0.004788	0.00999	0.01826
C10orf82	1.996022	0.02003	0.010035	0.003237
XCL1	0.88115	0.008876	0.010074	0.002547
FOXO6	3.443271	0.034757	0.010094	0.001686
TFCP2L1	1.747995	0.01773	0.010143	3.35E-05
HOXA7	1.598658	0.016255	0.010168	0.021129
CDC42EP5	7.336333	0.074656	0.010176	0.003618
TM6SF2	2.165353	0.022119	0.010215	0.003919
GATA2	10.87922	0.112206	0.010314	0.000852
NINL	2.986003	0.030893	0.010346	9.03E-05
EFNB3	4.956718	0.051307	0.010351	0.008724
APOD	45.11826	0.468102	0.010375	0.028512
ABLIM2	0.569209	0.005957	0.010465	0.003915
MLC1	0.367182	0.003845	0.010471	0.07599
NID1	68.21924	0.715638	0.01049	7.87E-06
ST6GAL1	3.606829	0.037936	0.010518	0.007659
EFNA2	1.920313	0.020284	0.010563	0.002299
ZNF613	0.995461	0.010527	0.010575	0.010671
SALL4	1.268422	0.013486	0.010632	0.004962
CELF4	0.508311	0.005415	0.010653	0.010375
ADM2	1.48758	0.015862	0.010663	0.009125
SLC6A16	2.464883	0.026517	0.010758	0.000938
CCDC170	0.284403	0.003061	0.010762	0.051599
PLLP	2.98657	0.032184	0.010776	0.02191
CLIC5	0.372129	0.004014	0.010788	0.002532
HOXB6	10.81507	0.116916	0.01081	0.00648
SSUH2	0.12585	0.001364	0.01084	0.036595
RET	1.100074	0.01196	0.010872	0.013647
ADGRG6	1.470097	0.015996	0.010881	0.001241
LDOC1	25.36226	0.276847	0.010916	0.00033
ZNF850	1.15195	0.012687	0.011013	9.33E-05
MYT1	1.854793	0.020434	0.011017	0.001914
CHRFAM7	0.80533	0.008901	0.011053	0.000108
SERPINA3	163.5324	1.810946	0.011074	0.001701
CBFA2T3	0.543961	0.00604	0.011103	0.000948
KLC3	4.731013	0.052647	0.011128	0.000445
EMX2	1.526586	0.01703	0.011156	0.003875
EN1	1.505808	0.016881	0.011211	0.001072
RBP7	4.368604	0.049037	0.011225	0.001867
MAF	1.508791	0.016957	0.011239	0.002525
SEMA4A	2.778119	0.031257	0.011251	1.88E-05
FUT1	5.165123	0.058244	0.011276	0.050479
ZNF738	2.189869	0.024751	0.011303	5.93E-05
CCDC3	2.299237	0.026003	0.011309	0.015407
TRO	11.07929	0.125985	0.011371	0.000972
MYOZ1	0.891231	0.010145	0.011383	0.025204
SMO	13.36562	0.15252	0.011411	1.51E-06
SLC25A27	15.95138	0.182753	0.011457	0.002703
NXN	8.543494	0.098372	0.011514	0.000225

TEX19	4.019845	0.046375	0.011537	0.008272
PTH1R	0.592339	0.006855	0.011573	0.009935
PRSS33	0.737939	0.008542	0.011576	0.034857
CNNM1	1.641726	0.019198	0.011693	0.006137
SLC15A1	0.456022	0.005354	0.011741	0.001187
ACSL6	0.592549	0.006966	0.011757	1.87E-05
CYP39A1	0.991926	0.011701	0.011797	0.000352
TIAM2	1.807634	0.021559	0.011926	4.51E-06
NKD1	0.509642	0.00609	0.01195	0.009759
CASKIN1	4.047342	0.048741	0.012043	8.44E-05
TRPV4	1.666565	0.020091	0.012055	0.002522
ZNF83	3.490379	0.042108	0.012064	0.000145
ZNF790	0.941133	0.011363	0.012074	0.005559
BMP8B	3.486554	0.042387	0.012157	0.001094
RBP5	0.787544	0.009617	0.012211	0.017721
TTLL6	0.271184	0.003315	0.012226	0.00141
IRF6	0.291488	0.003565	0.01223	0.000674
NRG4	0.110264	0.00135	0.012243	0.06651
GNAL	3.060418	0.037553	0.012271	0.001693
GPM6B	5.079725	0.062343	0.012273	0.00028
ALDH1A1	2.206487	0.027116	0.012289	0.085521
TAC1	1.146376	0.014093	0.012294	0.041273
LOC10272	0.841731	0.010355	0.012302	0.02327
GOLGA8H	1.024138	0.0126	0.012303	0.01777
DAPK1	4.817584	0.059334	0.012316	0.000853
LOC10537	0.467482	0.005768	0.012337	0.002406
CHN2	0.733493	0.009064	0.012357	0.005278
THPO	0.609198	0.007555	0.012402	0.077465
FRRS1L	0.233144	0.002893	0.012409	0.005864
ATRNL1	0.427729	0.005351	0.01251	0.000857
PRKCB	0.480464	0.006012	0.012513	0.003017
CDK18	14.05074	0.176125	0.012535	0.002092
RHOXF1	1.494714	0.018783	0.012566	0.059912
LOC10536	0.162278	0.002041	0.012579	0.031778
SLC2A14	0.492307	0.006199	0.012592	0.015025
ADGRB1	1.237809	0.015602	0.012604	0.000104
ADAMTS1	0.463447	0.005857	0.012638	0.01271
DMTN	1.905527	0.024145	0.012671	0.000442
RYR2	1.69468	0.021501	0.012687	0.013205
HBE1	4.710818	0.05988	0.012711	0.000776
SLAIN1	9.921482	0.126387	0.012739	0.041246
C15orf59	1.080847	0.013773	0.012743	0.011478
ALX4	1.879059	0.024005	0.012775	0.006827
ZDHHC11	0.648597	0.008299	0.012796	0.000381
BARX1	4.286947	0.054952	0.012818	0.037025
PAPLN	1.239032	0.015936	0.012861	0.001807
ST6GALN/	0.7263	0.009349	0.012872	0.004351
AIF1L	17.54421	0.22629	0.012898	0.002716
LMOD1	3.18593	0.041287	0.012959	0.009011
COL3A1	3.011015	0.039191	0.013016	0.001817
RUNX3	0.753093	0.00982	0.01304	0.013408
CCDC160	0.765717	0.009992	0.013049	0.087498
PRELP	1.266332	0.016791	0.01326	9.46E-05
CCSER1	0.06539	0.000872	0.01334	0.000156
REPS2	0.626525	0.00838	0.013376	0.004292
LOC10798	0.119943	0.001605	0.013385	0.008356
NR2E1	0.357912	0.004791	0.013386	0.000945
CACNG6	1.923014	0.025804	0.013418	0.002205

SPNS2	1.378022	0.018535	0.01345	0.01324
IGDCC4	2.707418	0.036483	0.013475	0.0016
FAM183A	0.969459	0.013068	0.01348	0.001129
DEF6	3.713141	0.050056	0.013481	0.010893
RASGRP2	1.141123	0.015473	0.013559	0.005016
GRIN1	0.434204	0.005912	0.013616	0.007189
NDRG2	5.547342	0.075814	0.013667	0.000266
TEC	0.681299	0.009336	0.013703	0.005383
ITGA2B	3.82077	0.05242	0.01372	8.44E-05
DOC2A	6.801601	0.093617	0.013764	8.41E-05
MAG	0.472654	0.00652	0.013795	0.033394
MYCL	2.139216	0.029516	0.013798	0.002211
FRAS1	2.658943	0.036793	0.013837	0.000591
SHISA7	0.192164	0.002661	0.013848	0.07966
ZNF527	1.310657	0.018177	0.013869	3.24E-05
ZNF682	0.32757	0.004554	0.013901	0.000313
CCM2L	0.786087	0.01096	0.013942	0.014723
CLDN6	0.863923	0.012056	0.013955	7.63E-05
ARAP2	0.892402	0.012498	0.014005	1.45E-05
PKNOX2	3.674639	0.05156	0.014031	0.000859
ZNF91	2.548034	0.035983	0.014122	4.40E-05
PRR36	4.648443	0.065649	0.014123	0.00326
LOC40049	0.572649	0.008103	0.014149	0.006988
DUSP15	1.676036	0.02374	0.014164	0.000192
PRRT4	1.689439	0.023985	0.014197	0.000493
SYNGR3	10.08227	0.144614	0.014343	0.004033
IL27RA	2.964627	0.042825	0.014445	0.000253
CXCL16	2.773387	0.0404	0.014567	0.002597
RAB11FIP4	4.620968	0.067348	0.014574	8.48E-05
GLDC	14.199	0.207287	0.014599	0.006474
KCNC3	0.36039	0.005272	0.014628	0.001505
PALM3	5.926019	0.0873	0.014732	0.008742
NPTX1	6.987979	0.102985	0.014737	0.000352
NTRK2	0.104616	0.001545	0.014767	0.058614
CRACR2A	0.595978	0.008825	0.014808	0.073579
RNF125	1.649996	0.024468	0.014829	0.004807
MLXIPL	4.598153	0.068252	0.014843	1.53E-06
NOXA1	8.93186	0.133487	0.014945	0.000433
DLGAP3	0.105772	0.001586	0.014996	0.006012
RASL10A	2.168099	0.032619	0.015045	0.005684
BMP4	3.010525	0.045304	0.015048	0.00035
COLEC11	0.498018	0.007497	0.015054	0.12339
QPRT	37.7886	0.570345	0.015093	0.001159
TET1	2.525989	0.038193	0.01512	0.000274
PCP2	0.406425	0.006215	0.015292	0.001758
NKX6-2	0.418364	0.006439	0.015392	0.035234
ZNF597	0.588895	0.009078	0.015415	9.34E-05
ATF3	330.0478	5.105181	0.015468	0.008974
ZNF114	0.365528	0.005671	0.015514	2.98E-05
PI15	0.775937	0.012054	0.015535	8.17E-06
NLRX1	5.646989	0.08842	0.015658	0.000976
WDR86	0.165647	0.0026	0.015698	0.01005
IGSF9	0.252434	0.003969	0.015724	0.001565
SEMA5B	0.325984	0.005142	0.015774	1.32E-05
IGF2	0.961601	0.015222	0.01583	0.01835
COL9A1	0.462203	0.00732	0.015837	0.000163
HTR2C	0.215366	0.003411	0.015838	0.008628
SH2D2A	3.29905	0.052313	0.015857	0.021673

ZNF583	0.418865	0.006648	0.015872	0.032703
LOC10272	0.415201	0.006605	0.015907	0.135695
SYN2	1.214859	0.019396	0.015966	0.01497
TRMT9B	0.160897	0.00257	0.015971	0.030866
LHX3	0.318905	0.005129	0.016084	0.09767
ADAP2	2.591754	0.041749	0.016108	0.026111
IQGAP2	4.763919	0.076864	0.016135	0.008138
FOLR1	0.817969	0.013213	0.016153	0.119957
LOC10537	0.375604	0.006092	0.01622	0.062794
KCNJ3	0.413053	0.006715	0.016257	0.011842
PNCK	2.207991	0.035933	0.016274	0.001078
PBX1	7.739981	0.126691	0.016368	0.00744
SKAP1	0.397	0.006507	0.016391	4.09E-05
CAMK2B	1.386165	0.022802	0.01645	0.03816
HOXC4	18.21876	0.300579	0.016498	0.000968
TMEM216	8.107327	0.133783	0.016502	0.000236
PAX7	0.308011	0.005088	0.016518	0.022153
SLC13A4	1.884583	0.031148	0.016528	0.017831
CSMD1	0.063783	0.001054	0.016528	0.082921
VN1R1	1.293715	0.021383	0.016528	0.00419
CDC42BP(	1.032456	0.017069	0.016532	0.017494
PDLIM3	9.376207	0.156226	0.016662	0.000199
MAP7	4.126362	0.0688	0.016673	0.001463
DCST2	0.936213	0.015628	0.016693	0.043451
ZNF701	0.522315	0.008768	0.016787	0.002165
ZNF320	0.921465	0.015552	0.016877	0.010403
SLC35F1	4.923476	0.083223	0.016903	0.002433
CFAP77	0.322793	0.00548	0.016976	0.040119
KLHDC9	2.153233	0.037032	0.017198	0.014562
ITGA9	1.433116	0.024724	0.017252	0.00138
MMP11	5.419499	0.093616	0.017274	5.95E-05
LOC10106	1.035815	0.017918	0.017298	0.011404
SLC25A21	0.362664	0.006282	0.017322	0.012944
ABCB1	3.985801	0.069266	0.017378	0.000867
ITGA8	1.470128	0.025579	0.017399	0.003125
TLR5	0.525718	0.009149	0.017402	4.06E-05
PTPN5	0.338723	0.005909	0.017446	0.050435
SPTB	1.369584	0.02402	0.017538	0.000246
LOC10537	1.53597	0.026943	0.017542	0.06952
ITPKB	0.952622	0.016711	0.017542	5.24E-05
PDZD2	0.368598	0.006479	0.017578	0.000363
RASL10B	5.202307	0.091669	0.017621	0.005214
PRKCG	1.953162	0.03455	0.017689	4.76E-05
CCDC39	0.241432	0.004285	0.01775	0.050934
GIPC3	3.579054	0.06357	0.017762	8.11E-05
ATP7B	2.222647	0.039748	0.017883	7.50E-08
SLC2A4	1.17662	0.021051	0.017891	0.017632
SOX7	1.920098	0.034642	0.018042	0.00031
VIPR1	0.692513	0.012504	0.018056	4.46E-05
CITED1	23.65747	0.427288	0.018061	0.030741
EPHA3	3.850106	0.069903	0.018156	0.004905
TMEM178	1.073051	0.019497	0.01817	0.032595
SERPINA5	4.691935	0.085291	0.018178	0.000243
CDH12	0.556293	0.010118	0.018188	0.025088
STMN3	15.12375	0.275177	0.018195	0.000363
GUCY1A1	0.608955	0.011135	0.018285	0.005195
KCNIP3	0.905334	0.016573	0.018306	0.003764
TSPAN7	31.19747	0.573227	0.018374	0.000159

PLA2G7	0.703564	0.012979	0.018448	0.103357
CELF2	1.217843	0.022542	0.01851	0.001075
GOLGA8R	0.17483	0.003237	0.018516	2.97E-05
FXYD3	0.724456	0.013438	0.018549	0.057156
IZUMO1	0.522145	0.009686	0.018551	0.011742
NBPF15	15.04715	0.281123	0.018683	0.001431
LRRC36	0.635207	0.011991	0.018878	0.004608
TSKS	0.430311	0.008202	0.019061	0.003234
ATP1B2	9.688886	0.184687	0.019062	0.006348
SLC29A2	12.14093	0.231477	0.019066	9.13E-05
ZNF714	3.628144	0.069223	0.019079	0.000667
ZNF300	3.518456	0.067297	0.019127	0.011415
ZNF888	1.015519	0.019429	0.019133	0.024
TAS2R5	2.980358	0.057048	0.019141	0.045728
GLDN	0.308391	0.005909	0.019161	0.017076
MAPT	9.16478	0.175645	0.019165	9.35E-05
HCN4	0.579204	0.011102	0.019167	0.0001
FLRT1	0.681903	0.013075	0.019174	0.000378
SCARF2	13.6904	0.262514	0.019175	0.003571
ZNF846	1.417172	0.027236	0.019218	0.017938
TTBK1	0.065358	0.001259	0.019262	0.043917
FHIT	4.117043	0.079339	0.019271	0.003494
HEY1	74.37818	1.436254	0.01931	0.000495
ZNF880	0.473328	0.009166	0.019365	2.04E-05
AATK	0.974101	0.018919	0.019422	0.004241
INPP5D	2.059201	0.040125	0.019486	0.002427
CIB2	12.46063	0.243311	0.019526	0.002327
PRTG	1.083856	0.021207	0.019566	0.000351
KCNN1	2.547964	0.05004	0.019639	0.017189
LLGL2	3.588421	0.070566	0.019665	0.003478
MYO5B	1.573533	0.030964	0.019678	2.19E-05
SHC2	9.91787	0.195849	0.019747	0.001125
NIPSNAP3	0.698915	0.013837	0.019798	0.007742
HOXA11	9.010913	0.178901	0.019854	0.003912
GRIK4	0.175243	0.003483	0.019873	0.044933
SERTAD4	5.784665	0.114965	0.019874	0.002048
EDA	1.381514	0.027505	0.019909	0.008222
ACSM3	4.293529	0.085594	0.019936	0.007721
SORCS1	2.085584	0.04162	0.019956	0.000205
CCNI2	0.304247	0.006072	0.019957	0.04147
ZNF675	7.829016	0.156389	0.019976	0.000133
ZNF680	4.289475	0.085948	0.020037	1.10E-05
PLPPR3	4.096717	0.082534	0.020146	0.004792
LOC10798	0.74228	0.014955	0.020147	0.11367
CD1D	0.637989	0.012868	0.02017	0.035126
EGFLAM	1.035904	0.020935	0.020209	0.00442
MPZL3	2.371665	0.048125	0.020292	0.000445
HES1	54.7906	1.115368	0.020357	0.000439
PACSIN1	0.581671	0.011915	0.020483	0.011223
STOX1	0.649685	0.013357	0.020559	0.037191
KCNA2	0.060669	0.001248	0.020565	0.041678
ABCG4	1.571099	0.032476	0.020671	6.45E-05
ST8SIA6	0.127132	0.002634	0.020722	0.026983
SNAI3	0.469733	0.009774	0.020808	0.005474
SLC5A5	0.704445	0.01467	0.020825	0.008041
PRAM1	0.624767	0.013099	0.020967	0.017653
ZSCAN23	1.470705	0.030849	0.020975	0.005725
GABRE	0.179395	0.003773	0.021029	0.062513

ATP6V1C2	2.267881	0.047864	0.021105	8.64E-06
UNC5C	0.832709	0.017604	0.02114	0.026222
SEC31B	12.97372	0.274602	0.021166	0.022119
GAL3ST1	0.694416	0.014717	0.021194	0.041406
TENM1	0.451432	0.009586	0.021234	0.043613
MN1	1.233665	0.026199	0.021237	0.000123
HLA-DOA	5.832204	0.124	0.021261	0.000238
PRDM6	1.478975	0.031469	0.021277	0.000313
CDH1	2.078026	0.044417	0.021374	0.022293
FREM2	1.335928	0.028611	0.021417	0.029564
GOLGA8A	11.07605	0.237468	0.02144	0.0062
MARVELD	1.140667	0.024629	0.021592	0.000358
DCT	0.999799	0.021606	0.02161	0.020836
TTC39A	2.531384	0.054825	0.021658	0.005078
ZNF14	4.073263	0.08849	0.021724	0.016787
LOC10537	2.258073	0.049103	0.021745	0.02473
IQSEC3	0.294236	0.006438	0.021882	0.027624
ZNF385C	2.021377	0.04427	0.021901	0.001291
IGFBP2	37.30101	0.817508	0.021917	0.003753
FEZ1	10.60131	0.232421	0.021924	0.000138
ARHGAP4	6.227038	0.136606	0.021938	0.000158
ISYNA1	27.0939	0.596443	0.022014	0.000288
BCL7A	24.17593	0.535421	0.022147	4.05E-05
ZNF717	2.899249	0.064452	0.022231	5.89E-05
ZNF540	1.056801	0.023638	0.022367	0.00082
ALOX15B	0.263442	0.005913	0.022445	0.037651
SUSD4	5.490128	0.123384	0.022474	0.001827
CCDC175	0.186848	0.004209	0.022524	0.008963
TTYH2	2.237934	0.050546	0.022586	0.000458
ACVRL1	0.642819	0.014527	0.022598	0.034783
BOK	0.500986	0.011328	0.022612	0.014327
TSPAN2	4.037691	0.091361	0.022627	0.000855
LGI3	0.223046	0.005057	0.022673	0.020961
MTUS1	1.571288	0.035684	0.02271	0.004996
RERG	1.140566	0.026099	0.022883	0.052092
MYRF	3.242174	0.07435	0.022932	0.001049
TMEM145	3.756453	0.086449	0.023013	0.006481
KCNK5	2.078645	0.047844	0.023017	0.000187
RP1L1	0.090808	0.002099	0.023111	0.076068
MTMR7	0.482591	0.011162	0.023129	1.68E-05
FCER2	0.297848	0.00689	0.023131	0.085008
MSH4	0.217831	0.00504	0.023137	0.008937
MAP4K1	0.945438	0.021912	0.023177	0.001708
CNTNAP3	1.373765	0.031846	0.023182	0.001339
DNAJB13	0.156781	0.003648	0.02327	0.132496
GIPR	3.009274	0.070264	0.023349	0.002553
TP53TG3D	0.190278	0.004451	0.023393	0.078198
NAPSA	3.677703	0.086172	0.023431	0.00661
CNTNAP3	0.871086	0.020534	0.023573	9.76E-05
PIWIL4	0.218538	0.005163	0.023625	0.003435
SYT16	0.237227	0.005609	0.023644	0.000208
AP1G2	13.76714	0.328018	0.023826	0.001931
TERT	2.046738	0.048814	0.02385	0.022394
TRIM59	10.10716	0.241523	0.023896	8.04E-05
C1orf167	0.088032	0.002107	0.023938	0.117471
ZNF595	0.766569	0.018382	0.02398	0.002536
COL11A1	16.94697	0.407522	0.024047	0.015252
RSPO4	0.143036	0.003451	0.024125	0.029224

NFE2L3	9.021267	0.217888	0.024153	0.011555
KCNQ4	1.159156	0.028124	0.024263	0.007362
AOC3	1.962047	0.047933	0.02443	0.077778
SDC3	16.86617	0.41226	0.024443	2.69E-06
PGM5	0.188958	0.004645	0.024583	0.069728
LYRM9	1.759201	0.043274	0.024599	0.001415
MMEL1	0.198141	0.004875	0.024606	0.027156
FBLN1	30.31573	0.749017	0.024707	0.000391
ADAM11	5.262314	0.130169	0.024736	1.84E-05
IRX4	0.266218	0.006592	0.024761	0.000376
BIK	0.680573	0.016852	0.024761	0.100691
PLAC1	16.51394	0.408998	0.024767	0.000885
C20orf203	0.233788	0.00583	0.024936	0.062074
SOX6	0.695217	0.017396	0.025022	0.012463
HTRA3	2.797928	0.070263	0.025112	0.017044
FNDC5	0.384187	0.009648	0.025114	0.014788
DACH1	2.250356	0.056553	0.025131	0.02306
SOBP	1.469826	0.037	0.025173	0.000291
LOC10537	0.330412	0.008353	0.025281	0.063092
BSN	0.615902	0.01558	0.025297	0.018725
RASGRP1	0.388775	0.009852	0.025341	0.002053
TPPP	1.949106	0.049548	0.025421	0.003028
FAAH2	1.530759	0.038963	0.025453	0.012973
RGPD8	0.546736	0.01394	0.025496	0.012245
PPARG	3.322337	0.085078	0.025608	0.000375
SLC2A10	0.582731	0.014935	0.025629	0.018138
OCLN	2.331971	0.059894	0.025684	0.004992
TMEM221	0.567868	0.014586	0.025686	0.03161
GALNTL5	0.152357	0.003966	0.026029	0.002828
ZFP82	0.08768	0.002285	0.026058	0.052226
ZNF816-Z	0.465943	0.012155	0.026087	0.047964
ITIH5	0.638306	0.016762	0.026259	0.001005
EFNA1	14.97653	0.393483	0.026273	6.31E-06
SIGLEC1	0.148554	0.003925	0.026423	0.038785
PRPH	2.354524	0.062348	0.02648	1.78E-05
LRRTM4	0.641613	0.017143	0.026719	0.006994
NCALD	0.877037	0.023481	0.026773	0.02195
OVGP1	10.34911	0.278402	0.026901	0.009497
COL6A5	0.279256	0.007552	0.027043	0.005564
PRODH	1.512392	0.040964	0.027086	0.021024
TMEFF1	0.723326	0.019655	0.027173	0.004724
DKK2	0.324287	0.008831	0.027233	0.065178
CORIN	0.113958	0.003105	0.027243	0.006425
SOX2	3.875161	0.106149	0.027392	0.003674
SYNGR4	0.27818	0.007626	0.027413	0.001895
NHSL1	2.30274	0.063175	0.027435	0.003361
CRYBG2	0.216272	0.005936	0.027446	7.68E-05
IRX2	0.097252	0.002681	0.027568	0.056101
NKX2-2	1.515453	0.0419	0.027649	0.002748
RHOB	412.5301	11.44744	0.027749	0.000716
C3orf70	0.417836	0.011618	0.027804	0.000998
CACNB2	0.397607	0.011058	0.027812	0.000298
GOLGA8C	0.108153	0.003018	0.027903	0.071782
TES	35.99059	1.009578	0.028051	0.003983
EBLN2	1.038598	0.029239	0.028152	0.025446
ADAMTSL	0.114658	0.003228	0.028154	0.051496
PPP1R9A	0.837425	0.023614	0.028198	0.003484
ANO5	3.546104	0.100068	0.028219	0.001691

DNAH9	0.079018	0.002233	0.028255	0.041454
MFSD4A	0.994927	0.028169	0.028312	0.008209
RGS14	5.867204	0.166912	0.028448	0.001626
DSCAML1	0.11379	0.003246	0.028526	0.000399
PTCH2	0.370655	0.010594	0.028582	0.00111
BAHCC1	2.555894	0.073158	0.028623	0.000949
SERPING1	7.532826	0.215625	0.028625	0.00037
HOXD11	20.51477	0.588269	0.028675	0.000785
SOX3	0.280643	0.008073	0.028765	0.001273
VSTM2B	0.21757	0.006264	0.028789	0.075088
BTG2	26.54215	0.76618	0.028867	0.002353
STOX2	0.942862	0.027316	0.028971	1.06E-05
ZNF585A	6.341231	0.183861	0.028994	0.000617
SDK2	2.009778	0.058287	0.029002	0.008161
FRMPD3	0.859576	0.02496	0.029037	0.000631
CDH23	1.314689	0.038208	0.029062	0.001226
FAM189A:	1.833475	0.053315	0.029079	4.16E-05
RASIP1	1.557654	0.045295	0.029079	6.18E-05
CNKSR1	0.440949	0.012829	0.029093	0.00054
TSPAN15	2.876118	0.083811	0.02914	0.00027
DRC1	0.229339	0.006705	0.029237	0.198828
TUBB2B	30.30561	0.89298	0.029466	0.01523
GNAO1	0.874162	0.025774	0.029484	0.003481
DLEC1	0.352621	0.010456	0.029651	0.064276
PTH2	1.222208	0.036477	0.029845	0.028195
PGBD5	1.955047	0.058579	0.029963	0.006511
USP28	21.75736	0.652154	0.029974	0.000274
SPX	1.416527	0.042535	0.030027	0.001336
WNT9B	0.154833	0.004651	0.030038	0.005219
NEURL1	0.375214	0.011274	0.030048	0.002806
NECTIN4	1.063416	0.031982	0.030075	0.001088
KLHL32	0.098759	0.002972	0.030097	0.002617
ZNF423	0.4438	0.01337	0.030126	0.001224
C11orf45	0.847759	0.025578	0.030171	0.002223
TBX4	0.13062	0.003941	0.030172	0.025294
COLGALT:	3.012645	0.091079	0.030232	0.002937
SYT6	0.569278	0.017282	0.030359	0.000102
LOC10192	0.06965	0.002115	0.030367	0.032843
C16orf74	3.661675	0.111363	0.030413	0.042924
ZNF678	6.895344	0.209891	0.03044	0.001259
PLCH1	1.437487	0.044135	0.030703	0.002786
IFNLR1	1.657853	0.050915	0.030712	9.66E-05
LHX4	2.331152	0.071681	0.030749	0.000871
LOC10798	2.058268	0.063361	0.030784	6.20E-05
FSTL5	0.110834	0.003416	0.030821	0.186489
RBP1	12.61556	0.38985	0.030902	0.00103
COX6B2	1.586633	0.049245	0.031038	0.016138
TOX3	0.086052	0.002673	0.031057	0.033568
GSTM3	14.04157	0.437827	0.031181	0.003774
TSC22D3	105.0444	3.279499	0.03122	0.007884
OTUD7A	0.500877	0.015641	0.031228	8.90E-05
ZNF85	2.639732	0.082472	0.031243	0.036066
CTSH	18.82686	0.588403	0.031253	0.005131
NLGN3	0.546501	0.017126	0.031337	0.015005
FBLL1	2.321935	0.072819	0.031361	0.037526
TDRD12	0.604963	0.018994	0.031397	0.003223
ADCY5	0.303292	0.009524	0.031402	0.000385
FLT1	2.358908	0.074273	0.031486	0.005011

AFAP1L2	1.716353	0.054068	0.031502	9.36E-05
ZNF257	0.279558	0.008808	0.031508	0.030175
MTNR1A	0.198172	0.006251	0.031546	0.07745
RUFY4	0.115865	0.003662	0.031609	0.001642
CDKN1C	2.872849	0.090878	0.031634	0.002223
LRFN5	0.188751	0.005974	0.031652	0.037315
SULT1A1	2.163055	0.068687	0.031755	0.001534
SH2D3C	1.838306	0.058391	0.031764	0.003586
HOXD3	0.451587	0.014349	0.031774	0.014953
ITGAL	0.285673	0.009079	0.031782	0.00017
PAQR9	1.215464	0.038779	0.031905	0.009873
MYOM2	3.773119	0.121128	0.032103	0.002268
PCDH18	1.353175	0.043567	0.032196	0.010428
ZNF468	1.670526	0.053823	0.032219	0.024969
NIPAL1	0.568061	0.018389	0.032371	0.000833
PTGDR2	0.700914	0.022703	0.03239	0.048124
RPS6KL1	3.55861	0.11533	0.032409	0.003314
TXK	0.072406	0.002349	0.032448	0.102383
PRKX	5.412806	0.176075	0.032529	0.0002
COL9A3	15.80037	0.514261	0.032547	0.00141
DAPK2	0.272676	0.008891	0.032607	0.001642
ISL1	3.596452	0.11734	0.032627	0.0082
ERBB3	4.897829	0.159972	0.032662	0.018817
SIMC1	4.561037	0.149045	0.032678	0.00083
SLC25A48	0.389372	0.012732	0.0327	0.092394
CYP2S1	4.202771	0.137602	0.032741	5.55E-05
CHRNE	0.386748	0.012665	0.032748	0.087651
METTL24	0.46631	0.015301	0.032814	0.01618
PTGES3L	0.761286	0.025039	0.032891	0.003469
PPP1R13L	17.44649	0.57713	0.03308	0.003472
DAND5	0.292026	0.009667	0.033103	0.006405
APBA1	0.551083	0.018246	0.033109	0.000626
EBF3	4.11661	0.137834	0.033482	0.000774
ACSS1	9.003626	0.302206	0.033565	2.99E-05
TMEM163	0.703286	0.023638	0.033611	0.010533
YBX2	0.610515	0.020569	0.033691	0.001354
EVX1	0.155146	0.005233	0.033731	0.009017
GAL3ST2	0.337498	0.011398	0.033773	0.030958
RIMKLA	0.720792	0.024368	0.033808	0.001242
COL5A3	2.962266	0.100291	0.033856	0.010784
CFHR1	0.374503	0.012724	0.033977	0.031132
ATP11A	2.557162	0.086939	0.033998	5.36E-05
ABCC11	0.10105	0.003438	0.034023	0.003166
PXDN	9.264241	0.317002	0.034218	0.000533
SPINT1	2.359522	0.080767	0.03423	0.000756
ADAMTS1	1.466474	0.050271	0.034281	0.007959
KSR1	3.261502	0.111915	0.034314	8.48E-06
ARL11	0.122314	0.004198	0.034318	0.115045
PAQR6	6.219135	0.214133	0.034431	0.013313
ARHGAP4	1.062907	0.03661	0.034443	8.16E-05
ZNF551	1.663461	0.057301	0.034447	0.023053
GRIN2C	0.167845	0.005807	0.034597	0.000608
AFF2	0.13369	0.004626	0.034601	0.114137
TMEM25	5.129119	0.178565	0.034814	4.33E-06
LOC11226	3.99542	0.139365	0.034881	0.001191
GPR83	0.111479	0.003906	0.035042	0.086064
C1orf61	0.751042	0.026348	0.035082	0.004974
P2RY2	0.117861	0.004142	0.035146	0.049482

HEY2	4.745876	0.167281	0.035248	0.000121
BMP8A	0.273175	0.009641	0.035291	6.70E-06
CCDC68	0.400203	0.014139	0.035329	0.01196
PPP1R32	8.160855	0.288716	0.035378	0.036504
DNAH2	0.13581	0.004809	0.035409	0.01129
AMHR2	1.247307	0.044167	0.03541	0.003421
SLC6A12	0.184111	0.006535	0.035495	0.000105
GUCA1B	5.104323	0.181388	0.035536	0.049299
FOXO1	2.221967	0.079136	0.035615	0.000112
CGN	6.232644	0.22235	0.035675	4.11E-07
C17orf113	1.240387	0.044289	0.035706	0.032905
IPCEF1	0.067268	0.002408	0.035797	0.023135
CFAP74	0.06052	0.002168	0.035831	0.033587
ACTN2	2.586193	0.092854	0.035904	8.95E-05
AOC2	5.066213	0.181969	0.035918	0.027997
ESAM	1.214805	0.043696	0.03597	0.001586
FAM72D	2.482317	0.089297	0.035973	0.004468
TMEM121	1.182823	0.042626	0.036038	2.24E-05
ANKRD18	3.423655	0.123488	0.036069	0.00014
WNK4	1.382473	0.049929	0.036115	0.073375
ALDH8A1	2.473927	0.089499	0.036177	0.023219
FOXC1	73.82688	2.678908	0.036286	0.000599
FAM71E1	7.65666	0.277923	0.036298	0.000737
ZNF589	11.16412	0.406171	0.036382	0.000318
SEMA3E	1.076111	0.039406	0.036619	0.047524
FOXS1	0.331871	0.012185	0.036716	0.064284
ACE	2.077033	0.076263	0.036717	7.72E-05
DLL4	0.521907	0.019183	0.036755	9.43E-05
CDKL2	1.167387	0.04298	0.036817	0.015863
GGACT	1.442112	0.053107	0.036826	0.061748
PCDHA3	0.081646	0.003007	0.036828	0.019656
GPM6A	2.564859	0.094552	0.036865	0.004553
NGFR	4.013392	0.148038	0.036886	0.024313
LGI2	0.610497	0.022551	0.036939	0.010404
EXPH5	0.528729	0.019564	0.037003	0.00196
RRH	0.278063	0.010308	0.03707	0.101869
EPHX4	1.214351	0.045307	0.03731	0.088289
CGNL1	1.239134	0.04627	0.03734	2.00E-05
CDCP2	0.312897	0.011719	0.037455	0.047268
HOXC5	1.892902	0.071391	0.037715	1.70E-05
PMAIP1	168.4221	6.401426	0.038008	0.000737
APOE	12.60144	0.479241	0.038031	0.002614
SEMA4G	3.854878	0.146998	0.038133	0.001509
MEGF6	0.693603	0.026459	0.038148	0.015096
DACT3	2.985767	0.114009	0.038184	0.003151
JPH1	4.07621	0.155966	0.038262	0.004836
KIF17	2.408522	0.09236	0.038347	0.018134
CT45A10	0.28635	0.010993	0.038391	0.026746
CDH19	1.062479	0.040822	0.038421	0.030308
EYA2	4.677029	0.179828	0.038449	0.004448
GRTP1	2.927496	0.112784	0.038526	0.000116
TLL2	0.317271	0.01233	0.038863	0.040896
CX3CL1	0.893329	0.034812	0.038969	0.029195
RAG1	0.246798	0.009619	0.038974	1.33E-06
AREG	0.321932	0.012586	0.039095	0.099838
IGFN1	0.727465	0.028486	0.039158	0.253952
FAAH	1.815777	0.071168	0.039194	0.03977
TKTL1	0.736291	0.028877	0.03922	0.009025

UNC13A	0.540703	0.02121	0.039227	0.000302
RCOR2	3.390587	0.133014	0.03923	0.03225
PTPRZ1	0.565957	0.022209	0.039242	0.068044
MAK	0.688301	0.027166	0.039468	0.000459
AZU1	0.905101	0.035733	0.039479	0.004921
MDK	136.7401	5.401764	0.039504	1.24E-05
ZNF813	0.199547	0.007887	0.039527	0.071677
PATJ	5.904993	0.234239	0.039668	1.25E-05
AQP3	4.150115	0.165523	0.039884	0.010864
PARM1	5.608728	0.22451	0.040029	0.000981
TPTE2	0.161087	0.006478	0.040216	0.039366
LOC10537	0.062631	0.002522	0.04026	0.040016
ZNF208	0.036645	0.001477	0.040302	0.005321
SRPK3	0.198908	0.008022	0.040329	0.022584
FAM169A	2.667411	0.107771	0.040403	0.003441
GAGE12H	0.733846	0.029686	0.040453	5.87E-05
FLRT3	2.559284	0.103557	0.040463	0.006731
CHGA	0.497248	0.020124	0.040471	0.004199
CHST6	0.744804	0.030184	0.040526	1.96E-05
EFEMP1	4.559659	0.185449	0.040672	0.003888
FOXA3	1.82538	0.074272	0.040688	0.010127
FBXL16	3.268261	0.133064	0.040714	0.006199
ATOH7	0.518811	0.021145	0.040757	0.000741
CTAG2	2.448373	0.100025	0.040853	0.001108
CEL	0.99956	0.041054	0.041072	0.040741
GRID2	0.079475	0.003265	0.041081	0.012253
NRCAM	3.047503	0.125354	0.041133	0.000648
C1QTNF4	0.723786	0.029809	0.041185	0.012902
TSPAN33	13.21388	0.546041	0.041323	0.002008
FA2H	0.348993	0.014439	0.041373	0.011849
LOC10798	0.305435	0.012643	0.041393	0.002214
DRD2	0.144805	0.006006	0.041476	0.022719
PRSS42	0.228341	0.009476	0.041498	0.036746
CELF5	0.541579	0.022494	0.041535	0.026528
MUC19	0.031273	0.001299	0.041538	0.003629
SOX21	0.679899	0.028262	0.041568	0.072803
FAM124A	0.493333	0.020543	0.041642	0.000727
CCDC125	3.127673	0.130368	0.041682	0.002085
ERBB4	0.095871	0.003996	0.041684	0.023
CACHD1	7.843195	0.327501	0.041756	0.000189
TBC1D3C	0.105579	0.004412	0.041787	0.065728
ZNF667	0.151639	0.006342	0.041821	0.049121
GNG8	0.303551	0.012705	0.041856	0.000736
SYT12	1.035216	0.043478	0.041999	0.005543
LOC64658	0.2398	0.010073	0.042006	0.033854
GAS7	4.846	0.204037	0.042104	0.004545
RTN4R	2.426804	0.102207	0.042116	0.009236
CYP4F2	0.158332	0.006675	0.042157	0.052002
TMEM31	2.666282	0.113121	0.042427	0.077067
ETNPPL	0.690017	0.029347	0.042531	0.004866
CCDC141	0.026479	0.00113	0.042668	0.087938
PCDH7	1.275951	0.054677	0.042852	0.010497
CABCOCC	0.320565	0.013746	0.04288	0.041733
TMEM121	5.983488	0.257417	0.043021	0.001745
FBXL2	3.936975	0.169503	0.043054	0.000994
FAM110C	0.081527	0.003516	0.043123	0.048421
PLP1	5.059306	0.218262	0.043141	0.000862
HSPA12B	0.107274	0.004628	0.043143	0.249308

IL1RAPL1	0.043986	0.001899	0.04318	0.102316
TNIK	6.630877	0.286483	0.043204	0.000944
CACNB4	0.857811	0.037158	0.043318	0.000263
VAMP8	17.48702	0.757575	0.043322	0.004596
ZNF280A	0.346346	0.015088	0.043565	0.025615
CAPN12	1.222301	0.053385	0.043676	0.00711
ENPP3	0.587976	0.025714	0.043733	0.031814
CCNB1IP1	196.5977	8.601515	0.043752	0.000355
UHRF1BP1	8.395333	0.367339	0.043755	0.004452
PLEKHH1	4.29058	0.188547	0.043944	0.000651
EXOC1L	1.771612	0.077907	0.043975	0.057475
FES	0.250288	0.011019	0.044024	0.013239
USP49	3.515391	0.155431	0.044214	0.002703
SLC46A3	3.350367	0.148177	0.044227	0.02031
L1CAM	2.559086	0.113336	0.044288	0.002031
PDE8B	0.207518	0.009199	0.044329	0.006463
SPON2	0.302474	0.013422	0.044373	0.002314
RNASEH2I	13.27079	0.59145	0.044568	0.012974
FN3K	1.186008	0.052912	0.044614	0.022608
CYTL1	0.665413	0.029802	0.044787	0.043691
FSIP2	0.017157	0.00077	0.044904	0.059138
POF1B	0.088614	0.003979	0.044904	0.059138
ADGRA2	6.403042	0.287877	0.044959	4.04E-05
RFPL4A	0.146157	0.006576	0.044996	0.01027
BGLAP	1.44095	0.064844	0.045001	0.194683
CRMP1	0.716691	0.032255	0.045005	0.005991
HUS1B	0.34699	0.015653	0.04511	0.029943
COL4A3	0.248883	0.011239	0.04516	0.000777
LOC10798	0.354232	0.016004	0.045179	0.005672
CBX2	8.372747	0.378579	0.045216	1.25E-05
WDR72	0.18284	0.008301	0.045398	0.000832
SOX10	19.5423	0.887605	0.04542	0.011779
ZNF33B	2.422791	0.110148	0.045463	0.027342
DMC1	8.172018	0.372987	0.045642	0.016422
EPHB6	0.131219	0.005989	0.045644	0.023289
MUM1L1	0.164003	0.007488	0.045657	0.012401
ARID3B	25.22827	1.155608	0.045806	0.000364
ZNF391	4.328854	0.198376	0.045826	0.007871
LAYN	6.683502	0.306335	0.045834	0.002112
GPLD1	0.361678	0.016593	0.045877	0.001808
HOXA5	21.28592	0.976572	0.045879	0.000333
ZCCHC18	0.66939	0.030861	0.046103	0.001904
PPEF1	0.325979	0.015037	0.046129	0.045618
PLA2G4B	0.265362	0.012243	0.046139	0.081725
ZBED6	4.609713	0.213112	0.046231	0.044564
CLTRN	1.825453	0.08453	0.046306	0.002601
SRRM3	2.196158	0.101816	0.046361	0.009625
MAJIN	0.166334	0.007751	0.046598	0.159556
CASZ1	0.992013	0.046229	0.046601	5.94E-05
KCNT2	0.805174	0.037531	0.046612	5.28E-05
GALNTL6	0.19181	0.008949	0.046654	0.016902
RGS10	49.66145	2.318836	0.046693	0.003499
LAMA3	5.523618	0.259117	0.046911	0.001144
PCDH15	0.039484	0.001854	0.04695	0.01284
ALS2CL	1.47324	0.069306	0.047043	0.000472
ZNF518A	4.823025	0.22704	0.047074	0.002623
LIMS2	0.611765	0.028816	0.047102	0.007245
KCND3	0.019927	0.000941	0.047201	0.056553

GPR179	0.042622	0.002012	0.047218	0.001509
LRRC75A	1.204927	0.056951	0.047265	0.036514
TOX	0.322072	0.015237	0.047309	0.003818
FKBP5	6.296321	0.297892	0.047312	0.000274
CXorf57	21.9684	1.042408	0.04745	0.001813
SNCG	9.055624	0.432498	0.04776	0.034683
TAC3	0.400525	0.019164	0.047848	0.0273
MYB	2.412756	0.115635	0.047926	0.002937
CYS1	0.993528	0.047671	0.047982	0.019308
ZNF648	0.031564	0.001516	0.048019	0.096897
ZBTB10	8.878064	0.426456	0.048035	4.51E-07
KLHL14	0.41182	0.019792	0.04806	0.000879
MTF2	13.23964	0.63816	0.048201	5.24E-05
NT5C1A	0.614297	0.029632	0.048237	0.00083
SEMA3G	0.731447	0.035294	0.048252	0.038772
DAB1	0.341759	0.016524	0.048351	0.017071
SLC2A12	1.653035	0.08011	0.048462	0.013315
ZNF229	0.184357	0.008935	0.048464	0.000663
ZNF781	0.108319	0.005257	0.048529	0.006533
SAGE1	0.283662	0.013797	0.04864	0.01147
ZNF431	3.274178	0.160214	0.048933	0.000213
RAB39A	4.136229	0.202481	0.048953	0.001518
HSD11B2	1.066949	0.052235	0.048958	0.087543
PROM2	0.202845	0.009943	0.049017	0.005983
ZNF843	0.158368	0.007769	0.049059	0.090039
NPC1L1	0.426006	0.020918	0.049103	0.001442
FAXC	1.169243	0.057525	0.049199	0.0055
EPHB3	2.221611	0.109474	0.049277	0.006539
RNF157	4.578698	0.225906	0.049339	0.000441
ALDH5A1	5.939587	0.293101	0.049347	0.012831
SPATA18	1.782177	0.088182	0.04948	0.023398
COL11A2	0.654895	0.032503	0.049631	0.020409
ANGPTL1	0.091181	0.004529	0.04967	0.046142
SYNM	1.747291	0.086981	0.04978	0.00394
CNFN	5.839376	0.290817	0.049803	0.006502
TFAP2E	0.927585	0.046441	0.050067	0.094314
CCDC192	0.094211	0.004717	0.050071	0.004149
OPLAH	0.152909	0.007684	0.050251	0.049633
ADAMTS1	5.561853	0.279708	0.05029	0.007499
SLC6A11	0.387166	0.019536	0.050458	0.009183
VIPR2	0.093796	0.004736	0.050491	0.008793
RUBCNL	1.650869	0.083396	0.050517	0.00707
INHBE	10.02059	0.506634	0.050559	0.087688
PIPOX	0.125133	0.006331	0.05059	0.176854
SLAMF9	0.801609	0.040608	0.050658	0.048144
MIA	30.74257	1.558059	0.050681	0.022492
C2orf15	1.007167	0.051202	0.050837	0.071657
NPAS1	8.918084	0.456201	0.051155	0.005936
C2orf48	2.198922	0.112882	0.051335	6.67E-06
RNF183	0.052499	0.002701	0.051439	0.018124
HOXD13	15.14445	0.779249	0.051454	0.012363
FGF10	0.132331	0.006834	0.051642	0.062031
SNCA	5.757108	0.298154	0.051789	0.001509
FUT9	0.017086	0.000886	0.051859	0.026471
GPC2	7.603589	0.394323	0.05186	0.004769
TPH1	0.17036	0.008848	0.051939	0.266083
ZNF347	0.153833	0.007992	0.051949	0.036505
MSH5	40.23034	2.091669	0.051992	0.007036

ZNF708	1.387525	0.07215	0.051999	0.002328
ABCA3	9.461868	0.493061	0.05211	0.000265
STK32B	0.692737	0.036109	0.052125	0.009773
CTH	34.45274	1.797573	0.052175	0.05352
GOLGA6L1	1.973003	0.10295	0.052179	0.000423
NKAIN1	0.898699	0.047411	0.052755	0.085869
KIAA0040	2.021268	0.106636	0.052757	0.008987
LIPG	0.170667	0.009015	0.05282	0.080918
CPNE9	0.23728	0.012558	0.052927	0.082805
L3MBTL4	0.082515	0.004373	0.052995	0.012492
ABCA10	0.047594	0.002537	0.053298	0.005313
DKKL1	0.524544	0.02806	0.053494	0.000888
SPAG17	0.187135	0.010013	0.053509	0.002968
VPREB3	1.068879	0.057241	0.053552	0.018151
MAOB	0.409957	0.021959	0.053564	0.00771
PCSK4	3.128352	0.168312	0.053802	7.07E-05
SLITRK3	1.734621	0.093412	0.053851	0.025599
HLA-DOB	1.786256	0.096468	0.054006	0.001086
STK26	22.69884	1.226902	0.054051	0.001172
COL19A1	0.884777	0.047831	0.05406	0.015957
TBC1D30	0.775494	0.041925	0.054062	0.011867
CDHR2	0.069783	0.003774	0.054086	0.077089
TUBB8P12	0.191662	0.010374	0.054126	0.032485
ZFHX2	0.284692	0.015546	0.054608	0.031477
ACAD11	62.18585	3.400937	0.05469	0.000807
HOXB7	7.032861	0.384944	0.054735	0.002939
ZNF544	0.151146	0.008273	0.054736	0.053728
MACROD2	0.395156	0.021638	0.054759	0.004052
CNGA4	0.075302	0.004133	0.054882	0.398758
H1F0	650.5944	35.72786	0.054916	0.002248
MAPK13	0.359282	0.019741	0.054947	0.038539
ACTR3C	0.044872	0.002467	0.054988	0.000431
KLF15	2.351793	0.129685	0.055143	0.00909
AGAP9	19.67481	1.086134	0.055204	0.002429
LIX1	0.146615	0.008097	0.055224	0.014467
DHRS2	8.688906	0.480309	0.055278	0.018859
SCG3	0.354349	0.019614	0.055352	0.0149
GREB1L	2.393921	0.132821	0.055483	0.002304
FGG	0.430956	0.023952	0.055579	0.020791
CKM	0.344361	0.019162	0.055646	0.000892
DNAJC12	21.02202	1.171637	0.055734	0.001995
RAB40A	1.319731	0.073621	0.055785	0.002941
BACH2	0.690319	0.038621	0.055947	0.000256
KCNJ5	0.092393	0.005174	0.055999	0.127665
ANKRD1	5.878842	0.329583	0.056063	0.177387
ACVR2B	4.663721	0.262012	0.056181	0.001211
HAL	0.063969	0.003602	0.056316	0.081587
LOC11226	0.131074	0.007389	0.056371	0.271974
NHLH2	0.258078	0.01455	0.056378	0.005241
KBTBD11	1.905132	0.10818	0.056784	0.002447
LOC10798	0.218116	0.012401	0.056857	0.146369
NEDD9	0.880959	0.050158	0.056936	0.010697
LOC10013	0.682913	0.038979	0.057078	0.003198
CNGA1	0.066643	0.003807	0.057119	0.008513
DCLK1	0.552345	0.03163	0.057265	0.008851
LOC10798	1.540144	0.088461	0.057437	0.029617
SAPCD1	41.60404	2.392418	0.057504	0.009865
PLEKHB1	8.089594	0.46548	0.057541	1.24E-05

RRAGD	6.781043	0.390193	0.057542	0.000146
SHF	1.602931	0.09224	0.057544	0.007754
KIAA0895	4.909055	0.283519	0.057754	0.00164
MRO	0.05075	0.002931	0.057756	0.304639
KLK7	0.396432	0.022994	0.058002	2.02E-05
TBC1D3D	0.06551	0.003809	0.058145	0.039906
GFAP	0.190669	0.011094	0.058182	0.057227
LOC10798	2.431798	0.141846	0.05833	0.001211
CHSY3	0.098604	0.005752	0.058334	0.017563
TEX14	0.428223	0.024991	0.05836	0.000492
NOVA2	4.025661	0.235455	0.058488	0.000412
LOC39093	1.129246	0.06605	0.05849	0.096154
NFIA	1.514503	0.088835	0.058656	4.52E-07
PDE1A	0.309363	0.018177	0.058757	0.037778
PCDH1	1.324525	0.077843	0.05877	0.000165
LOC10192	0.8745	0.051496	0.058886	0.00148
TMEM139	0.314205	0.018542	0.059012	0.003704
BMP5	0.074405	0.004406	0.05921	0.018974
CTNNA3	0.02075	0.001229	0.059213	0.086519
ELAVL2	2.436605	0.144415	0.059269	0.003779
PCDH19	0.055547	0.003292	0.05927	0.016066
COQ8A	42.97439	2.568416	0.059766	0.000824
TSHZ2	0.922721	0.055255	0.059883	0.002419
C2	2.083817	0.124831	0.059905	0.006343
SESN3	5.618156	0.337694	0.060108	0.000108
PROX1	1.449071	0.087136	0.060132	5.39E-05
APOBEC3I	1.152997	0.069436	0.060222	0.003759
RHBDL1	4.879599	0.294098	0.060271	0.078005
PM20D1	0.117899	0.00715	0.060648	0.011101
AKNA	3.390258	0.205633	0.060654	0.011366
DOCK8	0.459989	0.027936	0.060732	0.015267
CAPN9	0.058475	0.003559	0.060856	0.000713
KCNQ5	0.811848	0.049428	0.060883	0.013622
SHISA3	0.989295	0.060249	0.060901	0.002126
SLC16A4	0.961924	0.058658	0.06098	0.000233
PRRX2	1.123364	0.068582	0.061051	0.164454
KCNJ1	0.180861	0.011045	0.06107	0.037054
WNT6	0.93639	0.057218	0.061105	0.002899
SLC25A23	18.65787	1.140127	0.061107	9.26E-05
PCDH17	0.346439	0.021176	0.061125	0.056523
PAIP2B	4.13865	0.25345	0.06124	6.63E-05
ADAMTSL	0.69598	0.042657	0.061291	0.000498
MARCKSL	144.2313	8.843262	0.061313	0.00328
MAP3K21	12.1314	0.745918	0.061487	9.39E-05
6-Sep	22.64414	1.394267	0.061573	2.29E-06
BCAN	0.126477	0.007789	0.061586	0.013578
ENPEP	0.26691	0.016441	0.061597	0.019822
MPP7	1.190323	0.073429	0.061688	0.015927
S1PR1	0.693965	0.042872	0.061779	0.012931
YOD1	13.20693	0.819429	0.062045	0.025765
COL4A2	27.06374	1.680583	0.062097	9.77E-06
GPR63	2.103504	0.130855	0.062208	6.50E-05
PLK5	0.694282	0.043191	0.062209	0.030833
MAEL	0.098439	0.006124	0.062211	0.083495
CYP27A1	1.02683	0.064078	0.062404	0.017368
TRIM10	0.124945	0.007799	0.062419	0.097786
MAP7D2	10.20459	0.637275	0.06245	0.001788
DYSF	0.3686	0.023053	0.062542	0.052833

ISM2	0.824564	0.051575	0.062548	0.000433
CILP2	2.801937	0.175365	0.062587	0.015558
LONRF2	2.436848	0.152541	0.062598	0.000648
RASEF	8.454481	0.529257	0.062601	0.007891
CCL5	0.402055	0.025177	0.062621	0.098447
PMEL	3.914858	0.245649	0.062748	0.001487
BCO2	0.685771	0.043059	0.062789	0.007517
GPRC5B	4.521033	0.284481	0.062924	9.18E-05
PLCXD2	0.719983	0.045497	0.063192	0.003735
ZNF525	0.04271	0.0027	0.063228	0.011346
FAHD2B	8.386223	0.530351	0.063241	0.000176
ZNF620	2.661663	0.169038	0.063508	0.006038
FLT3	0.12449	0.007915	0.06358	0.076079
XG	0.066892	0.004255	0.063604	0.300463
ANKRD20.	0.058678	0.003736	0.063667	0.132328
SFRP1	2.20437	0.140751	0.063851	2.51E-06
GLT8D2	2.367976	0.151296	0.063892	0.015087
CYP2J2	1.881762	0.12036	0.063961	0.039927
PHF24	0.070147	0.004495	0.064085	0.011569
ALDH3A1	0.119047	0.007667	0.064401	0.0115
GOLGA8N	0.862385	0.055615	0.064489	0.008547
ZNF703	97.67491	6.309586	0.064598	8.69E-05
CLEC4A	0.838177	0.054269	0.064746	0.032269
SPARCL1	0.079254	0.005137	0.064822	0.059371
LTB4R2	2.411218	0.15632	0.06483	0.026376
GNGT1	1.739199	0.112794	0.064854	0.014729
C1QTNF1:	4.567425	0.296236	0.064858	0.164839
GHR	0.742063	0.048135	0.064866	0.031986
TUB	10.90236	0.708018	0.064942	0.005176
SNTG2	0.139741	0.009084	0.065003	0.000259
ZGRF1	19.97462	1.301204	0.065143	0.007921
SLC4A9	0.186576	0.012181	0.065286	0.013595
BCHE	6.710966	0.438298	0.065311	0.00858
ARHGAP4	0.438004	0.028616	0.065332	0.103506
CCDC81	0.273184	0.017858	0.065368	0.010232
MCIDAS	0.357132	0.02339	0.065493	0.093139
CBSL	2.248106	0.147442	0.065585	0.003133
RXRG	0.829209	0.054394	0.065598	0.006124
FGD6	1.42028	0.093384	0.065751	0.000772
WIF1	0.108795	0.00717	0.065899	0.010433
NCR3LG1	2.714843	0.17901	0.065937	0.029617
TRPM6	0.09783	0.006451	0.065941	0.005294
CYP2E1	6.736463	0.444327	0.065959	0.041513
RAPGEF4	1.674394	0.110994	0.066289	0.004464
DUSP26	0.245398	0.01628	0.06634	0.108747
CCNJL	0.802044	0.053355	0.066524	0.009357
GRIN3B	1.24991	0.083194	0.06656	0.020578
KIT	5.265097	0.350477	0.066566	0.001102
RENBP	2.377584	0.15854	0.066681	0.002083
FBLN5	4.817914	0.321266	0.066682	0.006061
CHADL	0.217939	0.014554	0.066782	0.048821
FGD4	0.413403	0.027656	0.066899	0.000566
ADAM20	0.129411	0.008662	0.066937	0.00859
FMO1	0.081432	0.005458	0.067027	0.015709
KCNB1	0.059484	0.003994	0.067149	0.005339
ZBBX	0.777764	0.052275	0.067212	0.056027
POSTN	0.231895	0.015645	0.067466	0.164472
MEP1B	0.105128	0.007114	0.067669	0.020786

KIAA1614	0.058714	0.003975	0.067702	0.048593
SYPL2	4.976687	0.336944	0.067705	0.006117
PLCXD1	2.041774	0.138354	0.067762	0.009582
PRKN	0.317991	0.021552	0.067776	1.76E-05
MAPK8IP2	7.854989	0.532396	0.067778	0.001625
C9orf139	0.052058	0.003531	0.067822	0.190899
ASNS	467.258	31.77024	0.067993	0.005014
ZNF618	0.987505	0.067154	0.068003	0.000114
LOC10537	0.512203	0.034899	0.068136	0.023684
SYT7	0.972447	0.066278	0.068156	0.000283
SLC5A1	0.089796	0.006134	0.068314	0.001491
CD72	4.619028	0.316242	0.068465	0.016985
ARMC12	0.565471	0.038717	0.068469	0.003587
PDLIM4	7.439608	0.51193	0.068811	1.59E-05
ATP8A1	3.814792	0.263665	0.069117	0.000246
GAB2	5.587179	0.386518	0.069179	0.005892
ANO1	0.085642	0.005928	0.069221	5.32E-05
ABHD1	1.762359	0.122608	0.06957	0.006489
GVQW2	1.651508	0.115623	0.070011	0.002696
IGSF3	7.160453	0.502187	0.070133	1.86E-05
ACAN	0.30864	0.021699	0.070305	0.031161
LRAT	0.325136	0.022862	0.070316	0.000138
PROSER2	3.505744	0.247936	0.070723	0.000583
GALNT4	0.042018	0.002977	0.070845	0.024569
RAB19	0.100038	0.007116	0.071128	0.004073
SP8	0.936026	0.066599	0.071151	0.000564
PROB1	3.123187	0.222686	0.071301	0.004066
PRSS12	7.319564	0.522698	0.071411	0.003485
AVPR1A	0.028717	0.002051	0.071414	0.00323
ATAD5	15.50107	1.107358	0.071438	0.005573
EXO5	1.262576	0.090233	0.071468	0.000229
EXO1	65.9263	4.716864	0.071548	0.000513
RGCC	5.821229	0.417154	0.071661	0.000814
CCDC88B	2.456331	0.176127	0.071703	0.00883
PIK3R5	0.438119	0.031438	0.071758	0.013802
RPP14	0.063858	0.004588	0.071854	0.150377
SLC16A10	1.452498	0.104496	0.071942	0.002667
ANKRD36	1.762987	0.127023	0.07205	0.000399
PSTPIP2	6.049605	0.436146	0.072095	0.004258
FOXP2	2.41602	0.174283	0.072136	0.010861
LOC10798	0.207507	0.014988	0.072227	0.140416
C8orf49	0.232323	0.016785	0.072248	0.130861
LIMD2	6.705336	0.485012	0.072332	7.01E-05
GPRIN3	2.472874	0.179625	0.072638	0.00018
NKPD1	0.227384	0.016528	0.072686	0.01498
CYP7B1	0.065632	0.004773	0.072726	0.020845
IRAK3	0.054713	0.003986	0.072853	0.069918
AMT	8.924771	0.650349	0.07287	0.030587
GSDMB	17.66935	1.287596	0.072872	0.061482
TLL1	0.107977	0.007878	0.072965	0.077716
ST6GALNA	0.906335	0.066208	0.07305	0.011971
ZNF718	0.034867	0.002548	0.073067	0.085046
KLHL15	13.17792	0.964553	0.073195	0.009627
VCX	0.233267	0.01708	0.073221	0.03329
AGAP2	3.552263	0.260579	0.073356	0.001887
GPX7	0.554438	0.040685	0.073381	0.017284
NME9	0.054756	0.004024	0.073492	0.011444
ZNF280C	7.223656	0.531245	0.073542	0.001697

BEX4	92.99035	6.845678	0.073617	1.75E-05
MAPK8IP3	13.3518	0.983467	0.073658	0.004615
CYP3A5	0.836455	0.061684	0.073744	0.001119
C15orf48	0.939721	0.06932	0.073766	0.095763
INHBA	6.04445	0.445918	0.073773	0.034241
LTB4R	6.562166	0.484223	0.07379	0.003224
CRYBA4	0.239572	0.017683	0.073812	0.03162
NPIPB9	0.404342	0.029883	0.073904	0.030683
PHKA1	12.35072	0.9139	0.073996	0.000323
SLC25A6	7.882069	0.585086	0.07423	0.012381
ZNF415	0.265261	0.019738	0.074411	0.034383
CSRP2	252.8357	18.82397	0.074451	0.008587
ATCAY	0.085658	0.006378	0.074459	0.001708
CCDC33	0.11044	0.008229	0.074515	0.007839
LYPD6B	1.586278	0.118219	0.074526	0.097286
LSAMP	0.18781	0.014001	0.07455	0.022026
INPP5J	4.45817	0.332937	0.07468	0.000126
HIC2	6.884368	0.514359	0.074714	1.12E-06
TLE2	9.239713	0.690996	0.074785	6.02E-05
ZKSCAN8	21.23439	1.594924	0.07511	0.011486
TBC1D7-L	0.826462	0.062256	0.075328	0.00051
TRIM7	0.938058	0.070694	0.075362	0.009912
SLC4A5	0.506045	0.038163	0.075414	0.00457
ESRRB	1.127938	0.08511	0.075457	0.037336
NEU3	3.019995	0.228463	0.07565	0.002065
ZNF354C	0.104448	0.00791	0.075736	0.062829
NMU	12.50722	0.949174	0.07589	0.012555
GPCPD1	5.416081	0.41173	0.07602	0.00045
SYDE2	1.892088	0.143899	0.076053	0.000791
SCRG1	1.262341	0.096079	0.076112	0.065014
DNPH1	113.5694	8.648077	0.076148	0.00086
FRMD1	0.412676	0.031483	0.076291	0.000454
ZNF383	0.915985	0.069951	0.076367	1.66E-05
MOG	0.05514	0.004228	0.076686	0.059908
REC8	1.942999	0.149086	0.07673	0.104284
EDNRB	0.550904	0.042338	0.076852	0.014612
RND1	3.671675	0.283143	0.077116	0.01906
PLAT	56.22346	4.340683	0.077204	0.001572
F12	4.021667	0.310711	0.077259	0.022222
SPTSSB	0.144805	0.011194	0.077303	0.001745
KIAA1958	2.357252	0.182289	0.077331	0.000952
NXPH3	0.647917	0.050151	0.077403	0.010102
ELF3	0.751923	0.05838	0.077641	0.102068
IL4R	2.679544	0.208071	0.077652	4.69E-06
KDM7A	8.759499	0.681031	0.077748	0.006421
PKD1L2	0.232429	0.01809	0.077832	0.013256
CHST4	0.178844	0.013923	0.077847	0.143258
SCTR	0.025947	0.00202	0.077855	0.007723
NTF4	0.082047	0.006396	0.077952	0.112658
COL23A1	1.929309	0.150465	0.077989	0.016813
TMPRSS5	0.236127	0.018427	0.07804	0.036204
RGS9	0.934523	0.07303	0.078147	0.00195
NECTIN1	3.332046	0.260422	0.078157	0.000188
ARMC4	1.398297	0.109463	0.078283	0.021725
MELTF	8.349824	0.655799	0.078541	3.43E-07
KRT3	0.082698	0.006507	0.078686	0.290578
FEV	0.108588	0.008547	0.07871	0.126571
PADI2	1.261873	0.099337	0.078722	0.025654

DNAAF1	0.429052	0.033792	0.07876	0.040699
PLEKHH2	5.58528	0.440423	0.078854	0.003222
DNMT3B	10.36833	0.819492	0.079038	0.001251
IL18	2.485429	0.196794	0.079179	0.032734
FAM174B	0.543655	0.043092	0.079263	0.002098
FLJ45513	0.300118	0.023811	0.079337	0.090662
GPR4	0.09403	0.00749	0.079661	0.06246
ASIC3	5.839507	0.465446	0.079706	0.011044
PLEKHG3	2.744568	0.218902	0.079758	0.000696
CD7	0.303204	0.024202	0.079821	0.271842
RGPD3	0.138361	0.011072	0.080023	0.0274
LZTS1	1.113983	0.089323	0.080184	0.00024
AZGP1	2.256471	0.180982	0.080206	0.026208
EMILIN1	5.025811	0.403948	0.080375	0.011107
CARNS1	0.029831	0.002401	0.080478	0.083722
PKDCC	5.981959	0.481703	0.080526	0.000354
RAP1GAP	1.896793	0.152918	0.080619	0.013861
LRIT3	0.263172	0.021287	0.080888	0.008076
S1PR5	2.985899	0.242093	0.081079	0.002592
TRAF5	3.499428	0.284654	0.081343	2.71E-05
ISL2	18.67207	1.520185	0.081415	0.00015
CNKSR3	4.759517	0.387886	0.081497	0.000392
BAIAP3	1.113465	0.091058	0.081779	0.005237
SUSD2	1.262147	0.103487	0.081993	0.002497
NR5A2	0.506426	0.041595	0.082134	0.007583
PIK3C2B	2.180997	0.179387	0.08225	0.004661
CRISPLD1	2.819064	0.232097	0.082331	0.006416
HPCA	1.656055	0.136491	0.08242	0.015161
SLCO5A1	1.497901	0.123491	0.082443	8.66E-06
ZBTB37	2.315851	0.191126	0.08253	0.00609
PKP2	3.86719	0.31971	0.082672	0.000682
MEX3A	7.846243	0.648924	0.082705	0.00015
TAF4B	8.206416	0.680103	0.082875	0.004948
ST8SIA4	0.812479	0.067406	0.082963	0.008991
ZNF8	3.9358	0.32696	0.083073	0.001782
ANO4	0.183242	0.015226	0.083092	0.015102
TRIM6	3.584042	0.299299	0.083509	0.003213
ACKR1	0.181397	0.015152	0.083529	0.085845
SDR9C7	0.108869	0.009099	0.083577	0.188274
YPEL1	3.859595	0.322738	0.08362	7.74E-05
RSPH1	0.26993	0.022575	0.083633	0.308761
ADAMTSL	1.049927	0.087813	0.083637	0.006075
ALG1L	0.425289	0.035589	0.083682	0.024348
PDE4A	1.822531	0.152514	0.083682	0.000131
MSMP	1.151692	0.096429	0.083728	0.141047
SOX11	0.319256	0.026806	0.083964	0.037657
CACNA1D	0.249526	0.020954	0.083975	0.00144
CDC25A	67.42755	5.664561	0.08401	3.76E-05
SH3BGRL2	6.210272	0.521828	0.084027	0.000157
CSAG1	3.769802	0.316869	0.084055	0.026697
SLC9B1	0.573199	0.048222	0.084128	0.030594
HES2	0.366881	0.030894	0.084207	0.055082
FAM84B	16.79363	1.414762	0.084244	0.034328
PRKCZ	3.069314	0.258688	0.084282	0.001713
HSD17B8	22.5635	1.908152	0.084568	5.23E-05
HOXA2	1.517978	0.128507	0.084657	0.000978
SLC8A1	0.806571	0.068324	0.084709	0.044483
LRRC43	0.136437	0.011621	0.085172	0.000986

CCKBR	0.085727	0.007302	0.085172	0.000986
LRRC8B	1.623064	0.138306	0.085213	4.41E-05
STPG2	0.051325	0.004385	0.085431	0.195133
DBNDD2	42.24006	3.618062	0.085655	2.36E-06
NR5A1	0.433256	0.037133	0.085708	0.040336
LY6G6C	0.211704	0.018229	0.086106	0.177929
NPRL3	12.59169	1.084919	0.086161	2.42E-05
DMKN	46.34992	3.998124	0.08626	0.008655
C5orf38	0.036139	0.003117	0.086263	0.167588
BEX2	43.51397	3.754054	0.086272	0.001745
LONRF3	5.246091	0.452664	0.086286	0.000258
MESP2	0.825498	0.071487	0.086599	0.057529
VWA2	0.026639	0.002307	0.086607	0.17473
KLHL40	0.07664	0.006639	0.086626	0.212773
HTR1D	0.331602	0.028726	0.086628	5.89E-05
COLQ	1.001468	0.086761	0.086634	0.066608
GSG1L	0.10439	0.00905	0.086696	0.034929
TMPRSS2	0.168884	0.014649	0.086742	0.045485
FAM229A	21.36817	1.85451	0.086788	0.036467
ASIC4	0.604199	0.052586	0.087034	0.108659
CTXND1	0.027322	0.002379	0.087067	0.073964
ITIH4	1.266668	0.110327	0.0871	0.035591
SLC15A2	0.598418	0.052222	0.087267	0.040866
ARHGAP3	0.174659	0.015288	0.08753	0.00305
WFDC2	2.609181	0.228606	0.087616	0.01344
GRHL1	2.407479	0.211085	0.087679	0.00309
INSL3	0.209005	0.018351	0.087804	0.002118
EXOC6	3.497983	0.307506	0.087909	0.000227
SOCS2	2.913599	0.2571	0.088241	0.000378
ZBTB46	1.624462	0.143497	0.088335	0.002244
NTRK1	0.385736	0.034205	0.088676	0.062344
TSPAN11	0.810226	0.071883	0.088719	0.022473
PRDM7	0.089642	0.007966	0.088866	0.048568
BGN	3.320865	0.295327	0.088931	0.020654
PLCG2	1.680135	0.149483	0.088971	0.003495
RGPD5	0.362556	0.032336	0.08919	0.124054
SYTL1	0.971054	0.086703	0.089288	0.000661
FTCD	0.398895	0.035618	0.089292	0.038983
GDPD1	4.796061	0.429262	0.089503	0.006298
SNCAIP	0.751916	0.067406	0.089646	0.00274
NTS	1.737616	0.155911	0.089727	0.004464
PIM1	35.80052	3.212294	0.089728	0.044013
HSPA4L	14.41352	1.293914	0.089771	0.00119
ARL17A	1.682929	0.151258	0.089878	0.000303
E2F2	10.30411	0.929042	0.090162	0.000247
PCED1A	24.8333	2.240777	0.090233	0.000323
SCN9A	0.619459	0.055995	0.090394	0.034557
RFLNB	7.410436	0.671704	0.090643	0.000181
CDH26	0.155502	0.014098	0.090659	0.01333
ZNF418	0.105107	0.009539	0.090755	0.002403
B3GNT8	0.118462	0.010812	0.091267	0.005128
TLCD2	2.593733	0.236931	0.091347	0.002679
RASSF6	0.037128	0.003393	0.091396	0.025807
GPR35	0.48003	0.043919	0.091493	0.152984
IL32	8.837859	0.808602	0.091493	0.006773
ERBB2	9.214155	0.843373	0.09153	0.000176
ZNF43	0.043404	0.003974	0.091569	0.065383
TSPOAP1	0.03866	0.003544	0.091669	0.007451

KCNH4	0.0801	0.007347	0.091718	0.067807
LEFTY1	0.219929	0.020174	0.091729	0.000173
TRIM73	0.26698	0.024491	0.091735	0.080628
ZNF726	0.59496	0.054602	0.091774	0.000153
CEACAM1	3.485655	0.320216	0.091867	0.000888
MDM4	11.30512	1.03887	0.091894	0.00023
YJEFN3	5.920768	0.544973	0.092044	0.020661
TEX45	0.993321	0.091754	0.092371	0.005368
WNT3	1.694699	0.156643	0.092431	1.25E-05
MOBP	0.047574	0.004402	0.092537	0.256765
CCDC114	0.282251	0.026132	0.092585	0.000267
HSD17B6	5.153938	0.477696	0.092686	0.001773
SLC12A7	4.166507	0.386515	0.092767	0.000237
GRIK2	0.132057	0.012287	0.093047	0.005457
CYB5R2	2.045054	0.190476	0.09314	0.026286
TAS2R31	0.523351	0.048752	0.093153	0.132761
CCNB3	0.712076	0.066449	0.093318	0.070595
SLC16A14	1.081648	0.100953	0.093333	7.52E-06
TP53AIP1	0.018089	0.001692	0.093511	0.091607
ATG16L2	5.839111	0.54623	0.093547	0.02074
CAPN6	0.047652	0.004467	0.093747	0.091737
C1QTNF6	2.432022	0.228107	0.093793	0.000339
FAM178B	0.124707	0.0117	0.093818	0.021277
GAS1	3.891721	0.365261	0.093856	0.003713
CELF3	0.109213	0.01025	0.093857	0.001943
RDM1	10.37354	0.974074	0.0939	0.010418
VWA3A	0.078693	0.007393	0.093942	0.157125
TAT	0.061965	0.005825	0.094006	0.061649
REM2	0.542086	0.05099	0.094062	0.089451
CRIP3	0.227072	0.021393	0.094213	0.237636
ZNF441	1.690851	0.159456	0.094305	0.001447
VSTM2L	1.32513	0.124969	0.094307	0.011693
TAS2R19	0.528977	0.049902	0.094338	0.006165
SLC18A2	0.043954	0.004148	0.094364	0.060734
ABCA5	2.496341	0.235705	0.09442	0.000271
INSC	1.445107	0.13646	0.094429	0.045508
ERO1B	8.767618	0.828134	0.094454	0.000683
SLC6A13	0.073728	0.00697	0.094532	0.141684
AGMAT	0.272637	0.025821	0.094707	0.072615
BMP6	5.790156	0.54846	0.094723	0.005543
CREBZF	22.24817	2.108243	0.09476	0.001125
LOC10798	1.374765	0.130418	0.094865	0.009396
MYO5C	1.995703	0.189344	0.094876	0.012369
SOWAHB	0.13834	0.013126	0.094879	0.15815
TTC34	0.029945	0.002844	0.094989	0.080008
ZNF273	1.496443	0.142369	0.095138	0.000224
HIC1	0.485758	0.046251	0.095215	0.010333
TBX19	1.523136	0.145342	0.095423	0.008331
CYP2C18	0.065975	0.00631	0.095646	0.153362
ARL5B	14.82849	1.421109	0.095836	0.003013
ZNF485	0.749395	0.071837	0.095861	0.034684
C1QL4	6.199166	0.594643	0.095923	0.001965
DQX1	0.431675	0.041498	0.096133	0.06198
RIPPLY3	0.072033	0.006925	0.096141	0.150052
JPH4	0.03793	0.003647	0.096141	0.150052
HHIPL1	0.06741	0.006484	0.096188	0.009357
LOC10798	1.021026	0.098213	0.096191	0.014384
SPRY1	3.749953	0.361654	0.096442	0.009939

PKD2L1	0.071544	0.0069	0.096444	0.001866
LOC10106	0.185228	0.017889	0.09658	0.159695
ARHGAP1	31.50338	3.042767	0.096585	5.29E-05
EML5	1.209586	0.117165	0.096863	0.005637
ADCY10	0.35045	0.033973	0.09694	0.030297
UNC5B	16.10942	1.563817	0.097075	0.003098
RDH10	8.800126	0.854901	0.097146	0.000132
KRT8	36.96114	3.590649	0.097147	0.000365
OGT	74.08505	7.201523	0.097206	0.027003
PEAR1	0.078968	0.007678	0.097228	0.049122
MDFI	0.053204	0.00518	0.097367	0.159165
ECHDC2	0.069767	0.006807	0.097563	0.058467
LYN	2.854272	0.279466	0.097912	0.000386
FGF21	2.267679	0.222414	0.09808	0.082941
SYT3	1.828603	0.179364	0.098088	0.004436
ZNF322	6.147701	0.603075	0.098098	0.000587
PLXNC1	0.231446	0.022775	0.098405	0.004812
CSAG2	0.34469	0.033949	0.098492	0.083101
AOAH	0.148702	0.014648	0.098509	0.007111
UPK2	0.18194	0.017926	0.098527	0.083638
ZSCAN12	3.544246	0.349226	0.098533	0.00011
IGFBP5	20.05084	1.977475	0.098623	0.002836
C6orf48	506.3707	49.96728	0.098677	0.004355
LYNX1	0.033485	0.003307	0.098746	0.080788
HOXD9	21.68888	2.142244	0.098772	0.019431
LGI4	1.777086	0.176669	0.099415	0.054222
AQP1	0.501818	0.049914	0.099467	0.01056
FAM8A1	26.66134	2.652545	0.09949	0.003102
VCAM1	0.100515	0.010028	0.099766	0.026778
LOC40092	3.782809	0.377652	0.099834	0.000111
HVCN1	0.373454	0.037308	0.099899	0.043306
SINHCAF	39.94043	3.998112	0.100102	0.000102
WHRN	4.678568	0.469563	0.100365	0.000271
IP6K3	0.103049	0.010346	0.100398	0.001567
PIK3AP1	0.357871	0.035991	0.100569	2.91E-05
FAM69C	0.13132	0.013229	0.100737	0.047086
LOC10192	6.779127	0.683634	0.100844	0.00354
DHDH	1.655908	0.167122	0.100925	0.007591
DRD4	5.253023	0.53048	0.100986	0.041975
PRKAA2	6.900997	0.69712	0.101017	0.000769
NFATC4	7.935144	0.801744	0.101037	0.000213
ULK2	2.458853	0.248924	0.101236	0.000156
RYR1	0.114629	0.011611	0.101295	0.000935
GAGE2A	0.900533	0.091419	0.101516	0.00261
GRM2	0.037134	0.00377	0.101532	0.054733
FAM149A	1.130358	0.11481	0.101569	0.035967
CDT1	156.3949	15.88495	0.10157	0.005788
ARVCF	3.165346	0.321595	0.101599	0.007013
FMNL1	1.363316	0.138559	0.101634	0.000318
TXNDC16	7.499108	0.763995	0.101878	1.89E-06
DLX6	2.832208	0.288851	0.101988	0.016011
ADAT2	5.649285	0.576848	0.10211	2.34E-05
SLC8A2	0.242473	0.024777	0.102184	0.050791
CADM3	4.012273	0.410034	0.102195	0.000287
CACNA1G	0.41074	0.042028	0.102322	0.01472
SEMA3D	0.974056	0.099739	0.102395	0.001302
BTN2A2	18.31642	1.875942	0.102419	1.19E-06
SMIM24	0.880295	0.090193	0.102457	0.073935

ZIK1	0.109383	0.011222	0.10259	0.008322
LOC10272	0.532947	0.054687	0.102612	0.048824
ALDH1L2	17.35697	1.781054	0.102613	0.02495
SCML2	7.50914	0.772345	0.102854	0.001163
JAK3	1.411648	0.145197	0.102857	0.000986
PAWR	7.406602	0.762305	0.102922	8.57E-06
TPK1	0.378911	0.039006	0.102942	0.001221
DCAF4L1	0.369269	0.038024	0.10297	0.056953
CSTA	2.664319	0.274572	0.103055	0.022482
SLC13A5	0.088142	0.009087	0.103099	0.001878
MARVELD	0.058972	0.00608	0.103099	0.001878
NKTR	17.89645	1.84626	0.103163	0.012297
GREB1	1.873702	0.19339	0.103213	0.005758
PPP1R14C	11.20602	1.157933	0.103331	5.02E-05
CYP4X1	0.213442	0.02206	0.103354	0.025871
LOC10798	0.799045	0.082584	0.103354	2.71E-05
RAPGEF5	1.073911	0.111161	0.10351	0.00119
FAM78A	2.244113	0.232335	0.103531	0.009568
RBM44	0.385576	0.03997	0.103662	0.051221
GABBR1	12.27263	1.276786	0.104035	0.002505
EPPK1	0.320277	0.033327	0.104058	0.008114
ZNF600	0.113827	0.011865	0.104238	0.050805
RASL11B	0.808662	0.084393	0.104361	0.103155
ACTA1	0.210387	0.021971	0.104431	0.162634
CDC7	45.21441	4.723966	0.104479	0.000579
PLS1	7.575128	0.791453	0.10448	0.001209
DENND2C	2.484154	0.259872	0.104612	3.42E-06
SLC10A4	0.442737	0.046357	0.104706	0.068633
ZNF577	2.432075	0.254835	0.104781	0.025029
KLHL35	0.164523	0.017279	0.105022	0.048336
SC5D	14.32071	1.504247	0.10504	0.000592
ATG9B	0.246881	0.025956	0.105137	0.000623
PTGIS	0.687089	0.072267	0.105179	0.003449
RGPD6	0.074747	0.007881	0.10544	0.059752
DNHD1	2.795538	0.2948	0.105454	0.051124
RLN1	0.098092	0.010356	0.105578	0.032498
CTXN2	0.049	0.005184	0.105794	0.066934
LIN7A	3.755993	0.397419	0.105809	0.000524
DCHS2	0.096754	0.010247	0.10591	0.017767
ZNF562	5.218473	0.552854	0.105942	0.000764
CPAMD8	0.222553	0.023587	0.105982	0.000126
ADAMTS1	128.7549	13.64635	0.105987	0.025481
SPOCK3	1.397085	0.14837	0.1062	0.039962
WDR27	3.178595	0.338034	0.106347	0.002177
ST20-MTH	0.066125	0.007038	0.106429	0.006167
ZDHHC11	0.8242	0.087734	0.106447	0.047312
NEK3	4.962898	0.528426	0.106475	0.02119
NYAP1	1.919072	0.204824	0.106731	0.000836
CRB1	0.130472	0.013944	0.106876	0.032797
LOC10537	0.096218	0.010295	0.106993	0.009159
KLF9	21.24214	2.273503	0.107028	0.000103
C5orf63	1.573101	0.168993	0.107426	0.00168
RASSF5	2.423563	0.260554	0.107509	0.016277
CELSR2	4.184662	0.450642	0.107689	0.002699
FAM131B	2.17648	0.234457	0.107723	0.00137
GRASP	0.401973	0.043411	0.107996	0.03957
TMEM225	1.095435	0.118335	0.108026	0.021839
GOLGA6L4	0.153903	0.016649	0.10818	0.021095

MST1	6.006937	0.649845	0.108182	0.013648
FAM171A:	7.794557	0.843502	0.108217	2.99E-05
LOC11226	0.521857	0.056559	0.10838	0.006163
TXLNB	0.481156	0.052166	0.108419	0.051839
TBC1D3E	0.234256	0.025441	0.108602	0.211949
DAAM2	1.658609	0.180174	0.10863	0.002959
TNNC1	1.088857	0.118316	0.108661	0.012652
DOCK4	1.530066	0.166326	0.108705	0.001138
TMSB15B	0.412265	0.044915	0.108946	0.200319
TCTE3	1.380273	0.150477	0.10902	0.015718
ARL10	3.928563	0.429187	0.109248	0.043326
DLX5	1.503207	0.164473	0.109415	0.021798
N4BP2	1.799727	0.196918	0.109415	0.005232
GPRASP2	0.038471	0.004212	0.109487	0.137684
ZNF385B	0.303636	0.033273	0.109582	0.001878
HMCN1	1.756106	0.192784	0.109779	0.081092
HCRTR1	0.089979	0.009878	0.109783	0.001837
CATSPER2	1.952899	0.214476	0.109824	0.000867
GPR21	0.017287	0.001901	0.109956	0.02906
TCF21	0.041097	0.004519	0.109956	0.02906
STON1	3.062575	0.337172	0.110094	0.000267
CRABP2	88.64638	9.761016	0.110112	0.00551
COL2A1	0.025545	0.002813	0.110139	0.037963
SLC43A1	8.924503	0.983244	0.110173	0.012975
CABP4	0.021631	0.00239	0.110482	0.180301
RGPD4	0.290358	0.032094	0.110533	0.004138
CFAP46	0.035838	0.003963	0.110571	0.280459
RHOF	5.366444	0.593405	0.110577	0.009338
TULP2	0.497813	0.055076	0.110636	0.005641
ZC3HAV1I	2.203819	0.24532	0.111316	0.008857
CORO2A	1.808015	0.201391	0.111388	0.004531
REL	1.709434	0.190495	0.111437	0.011166
CLCNKA	0.170456	0.018998	0.111454	0.002723
SLC39A5	0.138953	0.015488	0.111465	0.029426
EGFL8	10.49558	1.170039	0.111479	0.001794
LRRC75B	8.193869	0.915122	0.111684	0.000227
C19orf38	0.89717	0.100299	0.111794	0.052363
SEMA6C	3.340878	0.373493	0.111795	0.000137
ASTN1	0.059301	0.006639	0.111948	0.021508
KCNK15	0.126924	0.014228	0.1121	0.055339
LRRC70	0.780169	0.087615	0.112302	0.019547
ABCC6	0.6372	0.07159	0.112351	0.003314
C11orf52	0.506868	0.056949	0.112355	0.127054
INHA	1.120141	0.126317	0.112769	0.063854
HSBP1L1	2.124607	0.240102	0.11301	0.002468
IBA57	2.217029	0.250552	0.113013	0.009129
RPH3AL	0.364051	0.041156	0.113051	0.012976
SIX1	15.66989	1.771797	0.11307	0.00013
ADAMTS2	2.346307	0.265454	0.113137	0.002728
LOC44029	0.065926	0.007459	0.113145	0.10116
LRFN1	7.210178	0.817199	0.11334	0.000261
WFIKKN1	0.219698	0.024967	0.113643	0.100049
EXOC3L4	0.024776	0.002816	0.11365	0.343553
SAMD5	1.278197	0.145453	0.113796	0.00114
PRSS27	1.813681	0.206485	0.113849	0.000501
BBC3	18.05196	2.058238	0.114017	0.000805
EYA1	1.945878	0.2226	0.114395	0.002321
PCK2	43.27098	4.959835	0.114623	0.019605

ELFN2	1.039129	0.119131	0.114645	0.007276
PHYKPL	6.254352	0.71833	0.114853	0.002784
MDC1	27.2837	3.136856	0.114972	0.002981
CDON	3.709105	0.426543	0.114999	2.07E-05
OVOL1	0.126769	0.014581	0.115022	0.072958
OXCT2	0.700597	0.080742	0.115248	0.022949
GDAP1L1	1.050838	0.121329	0.115459	0.178579
CREB5	7.961445	0.92198	0.115806	0.002659
KIAA1671	2.211097	0.256211	0.115875	0.007791
VWA7	0.205667	0.023882	0.116119	0.00682
RNF169	9.947421	1.157667	0.116379	0.000444
FBXO41	1.789807	0.208439	0.116459	0.000177
UBD	1.130371	0.131849	0.116642	0.022678
GALNT8	0.196906	0.022994	0.116777	0.107259
AMBN	0.278506	0.032527	0.116792	0.09716
MYEOV	1.035313	0.120995	0.116868	0.036285
SAP25	2.120575	0.248056	0.116976	0.202853
PELI1	3.079165	0.360346	0.117027	0.000252
NANP	7.22655	0.84603	0.117072	0.00063
PAPSS2	11.96596	1.40096	0.117079	0.002163
CD80	0.053351	0.006248	0.117108	0.025228
FAM53A	0.422894	0.049623	0.117341	0.01937
TLX1	0.296185	0.034867	0.117719	0.024835
CPE	41.09952	4.839265	0.117745	0.000268
C2orf92	0.842916	0.099253	0.117749	0.018116
GAA	28.83989	3.407007	0.118135	1.57E-07
PPARGC1I	1.298732	0.153509	0.118199	7.42E-05
DHRS4L1	0.724066	0.085642	0.118279	0.025483
MYO7A	0.17303	0.020467	0.118289	0.001875
ERC2	1.319293	0.15609	0.118314	0.002413
MYCBPAP	0.11751	0.013905	0.118329	0.23179
PAM16	1.132029	0.133967	0.118342	0.003122
ZNF274	12.3075	1.457983	0.118463	0.010618
ATP1A3	10.51914	1.248543	0.118693	0.006757
KIAA1107	1.134344	0.135172	0.119163	0.000129
CRYGS	2.840688	0.338974	0.119328	0.001058
ZFPM2	0.408115	0.048828	0.119644	0.003117
NR4A1	5.022988	0.601006	0.119651	0.011269
ANKRD23	2.021773	0.242094	0.119743	0.047415
ANKRD46	7.514418	0.900292	0.119809	0.000549
CHAC1	12.49909	1.498364	0.119878	0.054183
DERL3	2.962654	0.355883	0.120123	0.000837
IQCA1L	0.051195	0.006163	0.120376	0.100386
CD96	0.344366	0.041469	0.12042	0.09453
TMEM173	1.251624	0.150942	0.120597	0.029688
ODF3L2	0.407818	0.049209	0.120665	0.000793
STK31	0.856524	0.103382	0.1207	0.030845
SORCS2	0.387815	0.046824	0.120738	0.001882
CHD7	5.219422	0.631003	0.120895	0.001917
DSG2	31.43795	3.801499	0.120921	0.00159
MTR	16.99221	2.059248	0.121188	0.0006
SPATA45	0.261875	0.031739	0.121197	0.012305
HECA	11.60346	1.407936	0.121338	0.003062
SMAD9	2.970773	0.360847	0.121466	0.002702
CXADR	2.941865	0.357807	0.121626	0.000181
RUNX1T1	0.17099	0.020799	0.121636	0.085239
TSSK3	0.442872	0.053898	0.121702	8.66E-05
IGF1R	7.80598	0.950838	0.121809	0.000706

PCDHA2	0.065889	0.008043	0.122068	0.023857
SV2C	0.020239	0.002474	0.122258	0.280883
RMND5A	17.56432	2.147803	0.122282	0.000521
KMT5C	10.44424	1.278354	0.122398	0.000954
KRTAP21-	0.298026	0.036499	0.12247	0.33264
PLXND1	6.717865	0.823193	0.122538	0.000145
C11orf21	0.046407	0.005697	0.122767	0.217693
SMIM17	0.182803	0.022447	0.122791	0.016396
SNAI1	4.042472	0.496526	0.122827	0.009217
LOC10798	0.055029	0.006761	0.122856	0.074311
SNX32	0.910388	0.112105	0.12314	0.002053
SENP7	6.015101	0.742071	0.123368	0.000839
RUNDC3A	0.395956	0.048853	0.123381	0.007009
SLC22A17	6.212693	0.766623	0.123396	0.021289
GSTO2	2.106609	0.260028	0.123435	0.001421
TCHH	0.038244	0.004722	0.123479	0.206426
RNF138	33.69934	4.162745	0.123526	0.00026
ANKRD20,	0.382374	0.047283	0.123655	0.00991
SLITRK6	0.643719	0.079602	0.123659	0.124359
CPNE5	0.1976	0.024481	0.123891	0.05318
ACE2	0.246629	0.030568	0.123942	0.022975
EPB41	11.19476	1.387758	0.123965	0.000637
PEX6	2.642144	0.327808	0.124069	0.006258
PXT1	0.061368	0.007618	0.124144	0.172933
SCARF1	0.150533	0.01876	0.124624	0.025315
EXD3	4.171083	0.520508	0.12479	0.001596
KIAA1257	0.087383	0.010905	0.124798	0.065798
LRRC71	0.082631	0.010316	0.124846	0.050194
CREG2	0.572313	0.071511	0.124951	0.001314
FOXJ1	0.44959	0.056196	0.124994	0.001617
WDR97	0.117637	0.014713	0.125073	0.11165
CDCA7	54.58845	6.834001	0.125191	2.50E-05
MYOM1	0.130163	0.01632	0.125385	0.003759
ANKLE1	0.89378	0.112201	0.125535	0.036896
COL4A4	0.283899	0.035644	0.125552	0.003946
E2F3	7.598586	0.955328	0.125724	0.00111
NANOS1	0.650441	0.081888	0.125896	0.013191
PARP1	265.8971	33.49336	0.125964	0.001201
SYP	10.863	1.369704	0.126089	0.009407
NUP133	54.11184	6.823095	0.126092	0.000229
MCAM	7.623206	0.961254	0.126096	0.000507
KDM1B	13.68868	1.726087	0.126096	1.56E-05
FGF20	0.651472	0.082173	0.126135	0.0205
TAS2R4	1.160633	0.146552	0.126269	0.031331
PRKAB2	20.7625	2.632774	0.126804	0.00158
MLIP	0.011429	0.001451	0.126997	0.203345
MBP	0.280795	0.03567	0.127032	0.000418
NRSN2	11.19525	1.422442	0.127058	0.000271
DZIP3	15.96304	2.029795	0.127156	0.014062
SORBS1	3.268211	0.415608	0.127167	0.000166
MTMR1	7.496576	0.953616	0.127207	0.00126
HIST3H2B	0.578318	0.073582	0.127235	0.081841
PPP1R12B	2.472303	0.315897	0.127774	0.0046
LOC10192	0.472164	0.060444	0.128014	0.0106
GVQW3	1.06304	0.136107	0.128036	0.000104
KHDC4	79.93996	10.24883	0.128207	0.010773
NOXRED1	0.62275	0.079922	0.128338	0.013435
ADGRV1	1.733013	0.222442	0.128355	0.004235

RALGAPA1	2.28401	0.293662	0.128573	6.69E-06
ZNF302	10.75982	1.384647	0.128687	0.008496
ZNF449	8.728822	1.123476	0.128709	0.002349
TMOD1	15.77458	2.033957	0.128939	0.007429
MUC4	0.007726	0.000996	0.128954	0.335301
BCORL1	8.423115	1.087862	0.129152	0.000151
RAB6D	0.554031	0.071564	0.129169	0.007389
FAM184A	8.383734	1.084151	0.129316	0.016466
GLYATL1	0.032951	0.004265	0.129425	0.175785
PDZD7	1.873832	0.242658	0.129498	0.000705
NR3C2	1.407516	0.182455	0.129629	0.002516
YDJC	28.80964	3.736125	0.129683	0.007639
SCML1	17.08193	2.21612	0.129735	1.56E-06
PITPNM3	0.751108	0.097482	0.129784	0.010056
PTGER1	0.353089	0.045849	0.129852	0.096153
VTCN1	0.036004	0.004675	0.129859	0.340028
BTBD3	22.10707	2.871216	0.129878	0.004146
TMEM143	8.166592	1.060742	0.129888	1.51E-05
ARHGAP4	0.746788	0.097079	0.129995	0.006048
FHL1	89.24206	11.60168	0.130002	7.26E-05
ZNF430	4.686297	0.610133	0.130195	0.000609
TRPC3	0.847918	0.11059	0.130425	0.000225
HLA-DQA	11.55973	1.509363	0.130571	0.025507
TREX2	0.902498	0.117896	0.130634	0.019301
SGK2	0.71823	0.093828	0.130637	0.029925
ADCYAP1I	1.495754	0.195423	0.130652	0.014517
GRIP2	0.606211	0.079256	0.13074	0.000911
PDK4	2.472128	0.323445	0.130837	2.59E-05
MZB1	0.296817	0.038838	0.13085	0.224486
TMEM178	3.694408	0.48416	0.131052	0.000353
BST2	14.55487	1.909375	0.131185	0.054229
RNF122	12.94531	1.698453	0.131202	0.00086
MOV10L1	0.035991	0.004727	0.131329	0.204207
ATP2A1	2.077118	0.273006	0.131435	0.009706
THEGL	0.110347	0.014512	0.131511	0.042695
PMP2	0.666134	0.087604	0.131511	0.021546
CXCL6	0.147181	0.019361	0.131544	0.02085
ZNF772	1.471527	0.19368	0.131618	0.022674
ACSBG1	0.05837	0.007699	0.131893	0.104514
DLX4	4.163254	0.549608	0.132014	0.000115
C2orf88	0.420574	0.055537	0.13205	0.007287
DES	0.54196	0.071846	0.132567	0.004432
WFDC1	0.166937	0.022168	0.132793	0.093081
ARHGEF19	0.361507	0.048021	0.132835	0.0003
ZNF138	10.38006	1.379876	0.132935	0.000854
TCAP	2.80227	0.372571	0.132953	0.182795
ADGRL1	12.99902	1.72911	0.133018	0.000219
FBLN2	1.8269	0.243012	0.133019	0.133676
ANK3	1.526942	0.203335	0.133165	0.002644
KLK14	0.497487	0.06627	0.13321	0.009144
UNC80	0.014913	0.001987	0.133214	0.119058
LOC10272	0.315636	0.042085	0.133334	0.009651
ACSS3	9.157181	1.221983	0.133445	0.000953
U2AF1	3.833895	0.511899	0.133519	0.054387
PPM1N	10.18738	1.361243	0.13362	5.90E-06
MEF2C	1.645379	0.21988	0.133635	0.006123
ANO9	0.097316	0.013005	0.133641	0.00039
MSS51	2.503149	0.33493	0.133803	0.014746

PLAGL1	11.21318	1.502003	0.13395	0.00825
LRRC3C	0.179519	0.024111	0.134311	0.049483
NUTM2D	0.109218	0.014692	0.134521	0.023056
ACER2	0.902154	0.121532	0.134713	0.001057
BMF	5.183298	0.698583	0.134776	0.000989
FAM76B	4.918161	0.663063	0.134819	0.006474
LRP5L	0.913442	0.12327	0.134951	0.001248
HHAT	2.06673	0.278927	0.134961	5.42E-05
TNFRSF9	2.314522	0.313	0.135233	0.034007
CPEB3	0.372297	0.050456	0.135528	0.00221
CSDC2	3.078286	0.417244	0.135544	7.37E-06
TXLNG	14.63383	1.984781	0.13563	0.001481
DPEP1	0.074981	0.01017	0.13564	0.383341
PET117	0.621588	0.084321	0.135654	0.034406
MYEF2	5.900713	0.80051	0.135663	0.001387
LOC10536	0.195944	0.026591	0.135705	0.005448
SLC23A2	11.37939	1.544841	0.135758	0.01969
SERINC2	9.47433	1.286924	0.135833	0.000381
NUP153	30.72331	4.173813	0.135852	0.001212
UTS2	0.561989	0.076424	0.135989	0.273553
SMIM10L2	1.728155	0.23533	0.136174	0.025682
KMT2A	11.37164	1.549402	0.136251	0.0073
RIOK1	30.73751	4.189053	0.136285	0.005329
CHTF18	25.64295	3.494761	0.136285	0.000485
LIN9	6.831668	0.931578	0.136362	0.000864
FAM227A	1.766617	0.240952	0.136392	0.001117
ZNF185	2.251029	0.307148	0.136448	0.017935
FGF17	0.209632	0.028638	0.13661	0.058343
CDH18	0.07518	0.01029	0.136867	0.024684
PLEKHD1	0.082385	0.011293	0.137072	0.018319
LSMEM1	0.937197	0.128525	0.137138	0.018905
EXTL1	1.73329	0.237729	0.137155	0.077205
TMTC2	1.549581	0.213137	0.137545	9.26E-05
TLR9	0.18155	0.025075	0.138118	0.145823
KLHL24	4.854286	0.671485	0.138328	0.015332
NT5M	3.518157	0.487626	0.138603	0.00455
RTKN2	2.753917	0.381845	0.138655	0.001043
TNFRSF11	2.294803	0.318802	0.138923	0.000908
DCHS1	1.294263	0.180183	0.139217	0.000279
CLDN10	0.034816	0.004852	0.139358	0.226641
SLC26A10	1.515651	0.211393	0.139473	0.004523
KLK6	0.121705	0.016976	0.139488	0.194897
PITX3	0.847877	0.118331	0.139561	0.015694
CDS1	6.265444	0.875493	0.139734	2.80E-05
ZBTB39	5.278089	0.737943	0.139813	0.000111
SKOR1	0.78678	0.110021	0.139837	3.62E-05
LOC15005	3.146292	0.440084	0.139874	0.064355
PTGDS	1.578882	0.220927	0.139926	0.104736
SAT1	336.0287	47.04359	0.139999	0.093803
DTL	43.46795	6.08896	0.140079	0.001911
ZNF460	1.749497	0.245138	0.140119	0.029514
UPF3B	76.72627	10.75395	0.14016	5.29E-06
ZNF488	0.711728	0.099872	0.140323	0.03135
LOC10798	0.125907	0.017683	0.140441	5.62E-05
ST3GAL5	2.506667	0.352281	0.140538	0.000173
PTCD1	0.02073	0.002917	0.140726	0.062663
NUTM2B	0.034535	0.004878	0.14126	0.174128
GPR87	0.074746	0.010559	0.14126	0.174128

FOLH1	0.103353	0.014603	0.141297	0.101523
HMGN5	21.95116	3.106808	0.141533	0.012872
KCNA1	0.014209	0.002012	0.14158	0.216646
MYO15A	0.108031	0.015303	0.141655	0.000527
NHLH1	0.363683	0.051631	0.141967	0.039871
HIST1H3E	0.985633	0.140033	0.142075	0.026948
PLCB2	0.162334	0.023067	0.142097	0.087179
PROC	0.04967	0.007059	0.142121	0.003913
NPY1R	0.057117	0.008125	0.142257	0.01264
NDRG4	16.84096	2.395831	0.142262	0.009655
RSPH14	0.631187	0.089827	0.142315	0.0096
ZNF426	2.104301	0.29965	0.142399	0.008486
CCDC180	1.823243	0.259644	0.142408	0.013823
TECTA	0.248025	0.035325	0.142425	0.001106
PTPN13	14.92765	2.126154	0.142431	0.009082
CFAP53	1.543464	0.220033	0.142558	0.010645
CGREF1	0.257553	0.036729	0.142607	0.067825
ITGB2	0.100848	0.014382	0.142609	0.12111
LYG2	0.01104	0.001575	0.142666	0.215947
MSANTD1	0.072516	0.010349	0.142711	0.025873
KCNE4	0.760265	0.108518	0.142738	0.035337
LOC10192	0.367888	0.052517	0.142754	0.036898
CYP2D7	0.63854	0.091304	0.142988	0.030816
PRSS57	0.208208	0.029795	0.143104	0.096605
C8G	0.511024	0.073248	0.143337	0.041224
AP1S3	3.051601	0.43764	0.143413	0.000222
GPC5	0.571265	0.08197	0.143489	0.00199
TPO	0.027044	0.003882	0.143544	0.051216
ANKRD36	1.457807	0.20936	0.143613	0.001001
C3orf35	0.415913	0.059793	0.143764	0.026173
BPI	0.058402	0.008399	0.143821	0.167736
RFX4	0.025512	0.003669	0.143821	0.167736
KIAA0408	0.028429	0.004094	0.144008	0.256979
ATP6AP1L	2.485756	0.358108	0.144064	0.005514
KCND1	0.777592	0.112028	0.14407	0.091217
MEIOB	0.059133	0.008524	0.144153	0.333786
LOC10050	0.050642	0.007305	0.144246	0.071459
RPA4	0.146309	0.021175	0.144727	0.064328
OVOL3	0.141941	0.020543	0.144727	0.064328
SLC18A3	0.045769	0.006636	0.144996	0.222875
FAM135A	3.040357	0.441002	0.14505	8.24E-05
GARNL3	1.952723	0.283318	0.145089	0.000784
MAGEC3	0.05863	0.008518	0.145283	0.0056
SIAE	6.456466	0.938391	0.145341	0.002978
CLDN15	7.370283	1.071442	0.145373	0.00332
SYT13	0.375118	0.054545	0.145407	0.021925
USP21	36.77535	5.357457	0.145681	0.013041
SERPINE3	0.017294	0.002521	0.145752	0.177559
NHLRC2	4.893827	0.713553	0.145807	0.004934
CMTM5	0.053674	0.007827	0.145833	0.231945
ATP10B	0.08173	0.011948	0.146189	0.039275
POU2F1	4.005475	0.585941	0.146285	0.000269
AP1M2	0.39329	0.05755	0.14633	0.012873
SLC4A11	1.217932	0.178259	0.146362	0.007234
LST1	0.61777	0.090482	0.146466	0.083999
RNF32	0.44257	0.064834	0.146494	0.075101
MAGEB6	0.397476	0.058265	0.146587	0.020946
CSMD3	0.015967	0.002343	0.146717	0.032302

UGT2B4	0.409541	0.060113	0.146781	0.123394
KLHL23	0.083581	0.01227	0.146809	0.03691
CCDC183	3.779516	0.554893	0.146816	0.02222
LYSMD1	17.96185	2.638882	0.146916	3.66E-06
TOM1L1	13.03514	1.916343	0.147014	0.00207
FAM71A	0.097005	0.014262	0.147024	0.006431
PHF6	23.72689	3.501595	0.147579	0.00012
IL18BP	2.653412	0.391785	0.147653	0.032114
ARPC4-TT	0.166447	0.024625	0.147947	0.044915
B3GNT7	0.830431	0.123043	0.148167	0.025917
ASGR1	2.630526	0.390084	0.148291	0.000456
TAF1D	97.56662	14.47591	0.14837	0.005834
SLC26A11	5.993605	0.890203	0.148525	0.000179
ZNF516	4.156299	0.617751	0.14863	6.03E-05
IL18R1	1.072788	0.159465	0.148646	0.007454
EIF4E3	8.001758	1.190917	0.148832	7.81E-05
SCN8A	0.643388	0.095815	0.148923	0.026034
SNX31	0.033111	0.004936	0.149075	0.174444
C3orf80	0.940977	0.140409	0.149217	0.018618
AFDN	19.85373	2.962563	0.149219	0.003205
NR2E3	0.153829	0.022974	0.149349	0.004891
ZNF253	0.180192	0.026912	0.149349	0.004891
PTPRF	26.85529	4.014026	0.149469	0.00016
CSRNP3	0.683609	0.102266	0.149597	0.000457
C1orf198	16.4891	2.467058	0.149617	0.000733
IGSF9B	0.744711	0.11151	0.149736	0.045138
C10orf95	0.152174	0.022795	0.149795	0.099921
CACNA2D	2.462324	0.368939	0.149834	0.000171
PYGL	132.0575	19.78896	0.149851	0.001296
TMEM217	0.105577	0.015823	0.149873	0.004895
NAALAD2	0.907158	0.136073	0.149999	0.000412
TRPC5OS	0.036195	0.00544	0.150286	0.337487
NFRKB	10.92647	1.643421	0.150407	0.002124
TMC4	0.21917	0.032982	0.150487	0.072933
MAGEA12	1.308198	0.197139	0.150695	0.026255
DYNC2H1	5.523279	0.833266	0.150864	0.007938
RAB40AL	0.425288	0.064203	0.150964	0.140439
PARP2	88.14234	13.30691	0.150971	0.00166
PLA2G6	5.007767	0.756984	0.151162	0.003079
CCDC85A	0.132325	0.020006	0.15119	0.117839
DUSP8	6.549291	0.991181	0.151342	0.005801
ATL1	1.507696	0.228305	0.151427	0.018353
CCDC154	0.146412	0.022198	0.151613	0.236969
5-Sep	16.31616	2.474158	0.151638	0.005911
BTBD8	0.602691	0.091562	0.151922	0.006091
ZNF24	44.00786	6.688366	0.151981	0.000345
RASSF9	0.291648	0.044335	0.152014	0.070329
RNF44	10.908	1.661274	0.152299	0.000273
HCN3	4.530002	0.690097	0.152339	0.003783
MMP25	0.120218	0.018334	0.152506	0.020807
HAPLN3	11.42114	1.742525	0.15257	0.000669
ZAR1L	0.190685	0.02912	0.152713	0.178667
PMFBP1	0.341207	0.052135	0.152796	0.019033
RAC3	78.58958	12.01485	0.152881	0.00124
DEPDC4	1.698705	0.260248	0.153204	0.005341
RASL11A	11.30766	1.732654	0.153228	0.004255
PAN2	23.46004	3.60696	0.153749	0.005121
MED12L	1.160825	0.178823	0.154048	0.002522

MIB1	12.53647	1.931307	0.154055	0.000291
ABCA8	0.293485	0.045257	0.154206	0.040735
GADD45G	0.979359	0.151085	0.15427	0.102652
RPRD1A	39.63169	6.114242	0.154277	0.00065
DUOX2	0.05026	0.007759	0.154386	0.149178
NINJ2	0.361803	0.055895	0.154489	0.028897
PIK3IP1	3.401122	0.526213	0.154717	0.003662
SDE2	15.52427	2.403698	0.154835	8.97E-05
CXorf58	0.126926	0.019657	0.154869	0.00996
GAGE12G	2.071174	0.320804	0.15489	0.04178
NATD1	5.259293	0.81522	0.155006	0.001739
STAT4	1.759848	0.273007	0.155131	0.034959
PPM1L	2.246227	0.349083	0.155408	5.59E-05
SLC7A8	2.833729	0.440544	0.155464	0.001529
SLCO2B1	0.283447	0.044086	0.155536	0.051407
TMPRSS9	0.05129	0.007978	0.155548	0.008098
FAM193B	29.71046	4.631829	0.155899	0.024695
C4orf36	0.596996	0.093111	0.155966	0.008913
C11orf96	46.43804	7.254643	0.156222	0.053733
SNRNP48	18.36884	2.871005	0.156298	0.000827
CALCRL	0.250732	0.039228	0.156455	0.019368
TMEM56	3.632058	0.568887	0.156629	1.02E-05
LOC10798	0.079052	0.012391	0.156745	0.029772
GRIK5	0.678474	0.106574	0.15708	0.020548
FAM166B	0.322356	0.050662	0.157162	0.016043
C1orf52	8.50345	1.336467	0.157168	0.004858
MYSM1	11.33409	1.783751	0.157379	0.006019
RAP1GAP2	4.967897	0.78227	0.157465	9.99E-05
TMEM131	5.858979	0.922596	0.157467	6.05E-05
HIST1H2A	0.458169	0.072303	0.157808	0.169089
LOC10798	1.750172	0.27664	0.158065	0.010517
NEUROG2	0.4094	0.064794	0.158265	0.006593
ZNF74	9.346005	1.48253	0.158627	0.000992
KLF11	6.782677	1.077041	0.158793	0.001853
CARD8	3.476431	0.55245	0.158913	0.000524
DGKK	0.027629	0.004391	0.158941	0.044174
CEP290	10.63856	1.691531	0.159	9.66E-07
CSPG5	4.046303	0.64387	0.159125	0.013083
SYTL5	1.084643	0.17291	0.159416	0.005178
KHDC1	1.271521	0.203029	0.159674	0.061145
KIF21A	5.444747	0.870539	0.159886	0.001126
DUSP22	5.218807	0.835274	0.160051	0.000528
NOS1AP	0.700643	0.112147	0.160063	0.00089
LTB	0.230734	0.036949	0.160137	0.022599
TFAP2A	13.32357	2.134024	0.160169	0.000127
DIRAS1	15.45159	2.478311	0.160392	1.15E-05
LOC10193	1.313699	0.210777	0.160445	0.01887
RGS5	2.047595	0.328708	0.160533	0.049574
TICRR	12.99689	2.087553	0.160619	0.000191
RAVER2	14.61272	2.349046	0.160754	0.000851
LMLN2	0.079727	0.012841	0.161068	0.001588
HOXC6	6.557392	1.059365	0.161553	0.000504
RPL36A	3.716507	0.600531	0.161585	5.12E-05
CTBS	8.559372	1.383811	0.161672	0.000687
DCP2	11.93953	1.930319	0.161675	0.000118
AK7	1.988007	0.321465	0.161702	0.013225
FREM1	0.195452	0.031616	0.161756	0.002164
TAF5	13.87453	2.245385	0.161835	0.000276

RNF130	5.746681	0.930486	0.161917	0.000621
NOXO1	0.070563	0.011429	0.161964	0.213366
ZNF721	11.12067	1.801411	0.161988	5.06E-05
CPT1B	13.36925	2.166359	0.16204	0.021274
KHK	5.889328	0.954793	0.162123	0.011321
RAD51AP1	43.28396	7.020815	0.162204	8.88E-05
DMRTA1	0.312244	0.050814	0.162738	0.022948
ZNF512	21.07432	3.432764	0.162888	0.000994
KYAT3	38.73413	6.311111	0.162934	0.00051
SMCO4	4.167178	0.679038	0.162949	1.64E-05
LRRC69	0.674157	0.109993	0.163156	0.007409
DNAH1	0.773452	0.126229	0.163201	0.041776
LUC7L	25.17577	4.11699	0.16353	0.01191
HIGD2B	0.225201	0.036877	0.163753	0.096225
P2RX5	0.128949	0.021162	0.164109	0.066499
CALML6	0.443738	0.07292	0.164332	0.151615
NR2C1	10.16811	1.671083	0.164346	6.37E-06
CRB2	0.049266	0.008109	0.164601	0.115875
KIAA1211	2.23348	0.36787	0.164707	0.00055
ENHO	10.76712	1.77645	0.164988	0.012422
DNAH11	0.520834	0.085991	0.165103	0.019065
TFEB	2.282257	0.376933	0.165158	3.58E-07
DYDC2	0.344747	0.056951	0.165196	0.124998
SDCCAG8	4.294764	0.709827	0.165277	0.000139
THAP9	3.483172	0.575849	0.165323	3.84E-05
MBTD1	5.51786	0.914675	0.165766	0.006692
DISP3	0.040491	0.006729	0.166181	0.001589
HKR1	5.032534	0.836914	0.166301	0.000131
RLBP1	0.027779	0.004627	0.166558	0.329591
RHOJ	0.242824	0.040449	0.166579	0.065329
AGTPBP1	17.32456	2.8878	0.166688	0.000514
GSTA4	38.70857	6.45412	0.166736	0.00021
DOPEY1	3.517937	0.587071	0.166879	0.003507
AGAP5	2.800616	0.46748	0.16692	0.023227
XK	0.35978	0.060084	0.167003	0.058356
PURG	2.688682	0.449308	0.167111	0.002255
AGT	0.303232	0.050692	0.167171	0.054216
SLC7A5	80.9495	13.5427	0.167298	0.00506
PLAG1	4.601557	0.770242	0.167387	0.001671
ZNF92	9.714714	1.630589	0.167847	0.001674
PGM2L1	1.930994	0.324543	0.168071	0.000682
LOC10192	5.772603	0.970262	0.16808	7.56E-05
DCP1A	20.19602	3.398212	0.168261	0.00196
TNNI3	22.32052	3.758978	0.168409	0.002228
MTHFD2	236.4853	39.84591	0.168492	0.000376
KLHL6	0.012006	0.002023	0.168516	0.214049
HNF4G	0.379337	0.064009	0.168738	0.002845
TRIB3	20.60648	3.477523	0.168759	0.00556
CRYAB	5.909287	0.997452	0.168794	0.252209
HES7	2.440961	0.412469	0.168978	0.001101
ZNF121	6.761306	1.142674	0.169002	0.002163
KPNA5	4.829597	0.81675	0.169114	1.62E-05
ALDH7A1	28.9276	4.893313	0.169157	0.000566
LOC10013	0.663956	0.112368	0.16924	0.007367
CD4	0.027065	0.004583	0.169326	0.458767
SLC7A7	0.884685	0.149808	0.169335	0.011023
PAEP	0.089292	0.015122	0.169356	0.205316
IFI27	2.929149	0.496736	0.169584	0.013149

LNX2	5.124545	0.870102	0.169791	0.001078
CFAP70	0.986429	0.167601	0.169907	0.038477
IQCF1	0.119641	0.020355	0.17013	0.035822
RIMBP2	0.0186	0.003164	0.17013	0.035822
CYFIP2	16.68974	2.842494	0.170314	0.001863
AGAP4	3.396031	0.578699	0.170405	0.007994
CISH	0.566333	0.096507	0.170407	0.002585
HELLS	17.04245	2.905212	0.170469	0.000416
ABCD2	0.025676	0.004384	0.170751	0.129576
RAB37	0.178942	0.030565	0.170812	0.012357
ZNF157	0.628199	0.107313	0.170827	0.046786
MEIS1	5.353006	0.916467	0.171206	9.54E-06
CTAGE6	0.036221	0.006204	0.171293	0.33262
PLEK	0.033808	0.005791	0.171293	0.33262
PRCD	0.011282	0.001934	0.171381	0.125741
CHRM3	0.103583	0.017766	0.171514	0.000881
CSAD	6.735426	1.156794	0.171748	0.011948
LOC10192	0.136184	0.023392	0.171765	0.067426
DCLK3	0.017155	0.002947	0.171776	0.031044
VCX3A	0.093962	0.01614	0.171776	0.031044
CALHM4	0.017663	0.003034	0.171776	0.031044
CPZ	0.412147	0.070806	0.171797	0.271634
EPHB4	18.85124	3.239301	0.171835	5.74E-07
FAM46C	2.649594	0.455444	0.171892	0.000227
RHBDL3	2.156407	0.370836	0.17197	0.000497
FNDC11	0.088539	0.015244	0.172174	0.122456
HOXA3	1.733397	0.298993	0.17249	0.003825
TRAP1	153.1584	26.42173	0.172512	0.00261
HNF4A	0.01692	0.00292	0.172573	0.32028
SLC4A1	0.018319	0.003161	0.172573	0.32028
GOLGA8B	6.765304	1.167541	0.172578	0.02665
MRE11	14.7521	2.548856	0.172779	6.41E-06
CCDC181	6.44958	1.114595	0.172817	0.017804
LCOR	3.848874	0.666193	0.173088	1.33E-05
ZNF578	0.027603	0.00478	0.173155	0.08987
EMSY	8.246036	1.427881	0.17316	0.008135
FAM171B	4.983846	0.863069	0.173173	0.014534
TRIM51	0.059323	0.010278	0.173256	0.332624
PCDHGB3	0.040068	0.006944	0.173307	0.217177
PCDHA4	0.017454	0.003025	0.173307	0.217177
SLC24A4	0.244706	0.042415	0.17333	0.008742
DIRAS2	0.064262	0.011141	0.173361	0.078837
PPP1R15B	24.20653	4.197119	0.173388	0.002776
LOC11226	0.209037	0.03625	0.173413	0.065955
SH3TC1	0.044486	0.007716	0.173451	0.072087
RBAK-RBA	1.440493	0.249971	0.173532	0.059502
AKAP14	0.206793	0.035889	0.173551	0.004991
MMP9	0.604338	0.104979	0.173709	0.032127
CREB3L3	0.105932	0.018403	0.173724	0.195305
LENG8	59.3942	10.3284	0.173896	0.021676
PLCB4	1.840243	0.320553	0.17419	0.017557
TRIM9	3.477938	0.606078	0.174263	7.62E-05
GOLGA6L:	0.114125	0.019892	0.174298	0.147111
BAIAP2L2	0.287765	0.05016	0.174307	0.005593
ADAMTS9	1.652719	0.288122	0.174332	0.000165
C18orf65	0.729477	0.127181	0.174345	0.039138
MB	0.255363	0.044536	0.174402	0.002607
BCL2L11	3.399931	0.59305	0.17443	0.000412

PSD	0.638587	0.111468	0.174554	0.001342
ANKRD65	0.020803	0.003632	0.174568	0.038778
EXOC3L2	0.046837	0.008181	0.174676	0.46183
HCRT	0.159342	0.027833	0.174676	0.46183
NR6A1	2.58978	0.452588	0.174759	0.009222
PAK6	0.040115	0.007015	0.174862	0.134403
LOC10798	0.061763	0.010802	0.174893	0.217119
SPATA1	0.249182	0.043582	0.174902	0.025312
HEPACAM	0.036616	0.006407	0.174967	0.037745
IQGAP3	31.67539	5.545764	0.175081	0.01228
ESR2	0.256601	0.044949	0.175171	0.044032
TRPM1	0.014045	0.002462	0.175288	0.143628
KHDC1L	0.148316	0.02601	0.175368	0.208348
DISC1	0.67586	0.118536	0.175385	0.000689
TET3	2.364301	0.415136	0.175585	0.0131
KRBA1	6.542251	1.149994	0.17578	3.72E-06
ATAD2B	1.314491	0.231068	0.175785	0.004277
ANKRD36	0.764487	0.134397	0.175801	0.016533
BTAF1	15.62198	2.74736	0.175865	0.0055
ARRDC4	77.47611	13.62875	0.175909	0.003686
TPPP3	5.076278	0.893528	0.17602	0.006062
PXDNL	0.037208	0.00655	0.17604	0.209515
ENGASE	8.457851	1.490486	0.176225	0.001358
ZNF354B	5.116805	0.902445	0.176369	0.006076
CYP27C1	1.150983	0.203139	0.176491	0.001588
GLB1L2	9.542857	1.685593	0.176634	8.46E-05
TEAD2	10.87641	1.922571	0.176765	0.000613
S100A1	14.28606	2.528266	0.176974	9.95E-05
PRR25	0.077152	0.013661	0.177069	0.125407
CEP295	6.525501	1.155808	0.177122	0.000728
FHDC1	0.37099	0.065815	0.177403	0.001342
C1orf35	50.08232	8.888473	0.177477	3.31E-05
LOC10798	0.151625	0.026937	0.177656	0.035062
FNBP1L	7.797666	1.385997	0.177745	3.99E-05
C11orf65	0.498908	0.088711	0.177811	0.007757
CCDC153	2.523642	0.449512	0.17812	0.085147
WFIKKN2	0.044769	0.007975	0.178138	0.226615
PDCD4	35.03214	6.251002	0.178436	0.001406
RPS2	6593.849	1177.077	0.178511	0.000159
TCP11	0.297991	0.053329	0.178962	0.028738
KIZ	15.91854	2.856402	0.179439	9.76E-05
CEP135	6.317035	1.133541	0.179442	1.59E-05
SHPRH	2.018203	0.36227	0.179501	4.98E-06
UBN2	4.099097	0.735878	0.179522	0.006306
UBAP1L	0.212028	0.0381	0.179694	0.070328
SFN	0.553864	0.099576	0.179784	0.013816
ADH1C	0.052935	0.009524	0.179915	0.325088
DDTL	0.660096	0.118918	0.180152	0.044552
DNA2	9.873844	1.778839	0.180157	7.27E-05
ZFAND1	50.45226	9.101099	0.18039	0.000166
BCL2	1.598226	0.288675	0.180622	0.006871
RAD52	8.766056	1.586177	0.180945	0.001196
FGF11	6.341232	1.148287	0.181083	0.011808
AASDH	8.206345	1.486107	0.181092	0.000246
C3orf14	8.113222	1.469926	0.181177	0.009032
MAN2C1	39.27715	7.118241	0.181231	0.015773
ANKRD33	1.970187	0.357145	0.181275	0.000489
JADE3	6.028537	1.093193	0.181336	0.000227

PYGM	0.12419	0.022522	0.181348	0.013005
L2HGDH	5.151712	0.935693	0.181628	1.26E-05
SMARCD3	21.75679	3.955085	0.181786	1.01E-05
RDH12	0.570313	0.103832	0.182061	0.001813
LOC10536	0.416907	0.07611	0.18256	0.101803
SLC37A4	24.5666	4.485184	0.182572	0.019044
GSE1	8.107754	1.481582	0.182736	0.001209
ZNF649	3.039427	0.55553	0.182774	0.013237
TTLL4	8.675757	1.58768	0.183002	0.000155
C1orf226	4.367857	0.799651	0.183076	0.000643
TMEM268	4.839375	0.887528	0.183397	0.033139
NAPB	7.125406	1.306886	0.183412	0.007429
ZNF326	9.782813	1.796819	0.183671	0.003056
ADGRG3	0.324452	0.059677	0.183931	0.008131
FCGRT	18.08788	3.3274	0.183957	0.000156
ZNF165	1.840579	0.338672	0.184003	0.004557
PAXBP1	22.17539	4.08177	0.184068	0.001995
METTL7A	10.13321	1.865989	0.184146	0.001885
PRPF39	25.70079	4.740924	0.184466	0.002034
EME2	0.888464	0.163897	0.184472	0.090664
CFAP65	0.081926	0.015115	0.184493	0.001463
MAP3K1	1.553306	0.286729	0.184593	0.000318
PER1	15.42718	2.849475	0.184705	0.017027
AGBL3	1.050005	0.194058	0.184816	0.006632
CD34	0.317547	0.058752	0.185019	0.156373
NTM	0.159453	0.029505	0.185038	0.197924
KCNJ14	1.399642	0.259241	0.18522	0.006473
LOC10537	0.614629	0.113986	0.185455	0.214034
BRD2	94.35043	17.51374	0.185624	7.82E-05
LMF1	1.079501	0.200398	0.18564	0.003503
PPP1R1B	0.399854	0.074247	0.185686	0.003973
HOXB3	1.684316	0.313779	0.186295	0.002829
CLK2	50.14636	9.348738	0.186429	2.55E-06
ZNF469	0.06726	0.012542	0.186464	0.150974
CCDC15	4.237187	0.79012	0.186473	5.52E-05
PVRIG	0.108515	0.020239	0.186509	0.258274
ODF2L	4.545686	0.848371	0.186632	0.000176
FZD5	2.700722	0.5044	0.186765	1.95E-05
ELANE	0.101931	0.019039	0.186788	0.210832
PTBP2	8.372547	1.564438	0.186853	4.36E-05
NCAN	0.094464	0.017656	0.186901	0.014725
ABCA6	0.057251	0.01071	0.187066	0.110024
EBI3	0.562575	0.105346	0.187257	0.015048
MPHOSPH	19.61885	3.674775	0.187308	0.000408
TTC28	2.077359	0.389111	0.187311	0.005465
LRRK1	0.398002	0.074555	0.187322	0.013964
AMY2B	2.766081	0.518951	0.187612	0.003768
RHPN2	7.441281	1.398097	0.187884	6.12E-07
TSPAN13	26.56732	4.994003	0.187975	2.60E-05
E2F5	14.91065	2.805296	0.18814	0.031539
ALG10B	3.216518	0.605971	0.188393	0.001852
LYPD3	1.793166	0.337894	0.188434	0.04554
EZH1	21.08923	3.974216	0.188448	0.001875
SRRM2	74.31456	14.03072	0.188802	0.003386
TRHDE	0.077714	0.014674	0.188818	0.129856
MYO18B	0.010093	0.001908	0.189013	0.129042
ZNF747	2.746218	0.519711	0.189246	0.021725
GEMIN2	32.76969	6.2047	0.189343	0.005677

RREB1	3.712507	0.703127	0.189394	0.003017
GOLGA6L!	3.026173	0.573518	0.189519	0.019863
SRGAP2B	0.899003	0.17043	0.189576	0.000428
HOXD4	2.136987	0.405181	0.189604	0.001457
IL2RB	0.183702	0.034831	0.189604	0.02015
IRX1	0.211763	0.040255	0.190095	0.108354
FANCM	4.57095	0.869559	0.190236	6.04E-05
PDE11A	0.238167	0.045316	0.190268	0.018549
ADPRHL1	1.966575	0.374959	0.190666	0.00739
MATN1	0.137502	0.026229	0.190755	0.041011
OSCAR	0.538521	0.102746	0.190794	0.00211
ADGRD1	0.113315	0.021623	0.19082	0.001674
AEBP1	0.08208	0.015676	0.190986	0.090153
DNAH17	0.104435	0.019972	0.191238	0.246212
GPR132	0.043331	0.0083	0.191539	0.130082
LSM11	5.903313	1.131859	0.191733	0.000244
RPL5	2644.089	507.0438	0.191765	4.76E-05
LARP1B	6.695759	1.285151	0.191935	0.00013
EME1	29.94904	5.748716	0.19195	0.002513
LSR	14.93244	2.866518	0.191966	0.000257
RIMS2	0.138775	0.026656	0.192084	0.004087
KANK3	1.579044	0.303317	0.192089	0.000207
WTIP	2.556464	0.491268	0.192167	0.000976
NXPH4	12.06344	2.318306	0.192176	0.000113
FBLN7	0.435488	0.083695	0.192187	0.036641
FGF8	0.385985	0.074188	0.192203	0.061028
RAB3IL1	2.311713	0.444391	0.192234	0.000186
CEP170	12.09298	2.32484	0.192247	1.09E-05
ZKSCAN4	2.803837	0.53936	0.192365	0.000983
C6orf163	0.655577	0.126306	0.192664	0.067931
CRYBB2	0.075396	0.014543	0.192886	0.205878
LOC11226	4.65626	0.898451	0.192956	0.000635
RBFOX3	0.273093	0.052705	0.192994	0.197359
SLAMF8	0.171651	0.033137	0.193051	0.020407
TOMM20	299.6462	57.86666	0.193117	2.08E-05
IL6R	3.973957	0.768063	0.193274	0.01124
SRSF11	56.4203	10.91103	0.193388	0.006325
DGKE	2.554869	0.494223	0.193444	0.002889
TAF7L	0.338695	0.065519	0.193446	0.111135
YPEL2	3.706811	0.718271	0.193771	0.000948
SYT5	0.298576	0.05786	0.193786	0.018848
RBM38	12.80071	2.482018	0.193897	1.23E-05
SYNE4	0.251319	0.048731	0.193903	0.092454
PROCA1	2.596079	0.503441	0.193924	0.004815
TFAP4	10.4783	2.033195	0.194039	0.000195
STRBP	8.183181	1.588016	0.194059	0.002695
DNASE1	2.283168	0.443155	0.194096	0.012985
DNAH12	0.041232	0.008005	0.194156	0.048978
CCDC28A	27.07024	5.261608	0.194369	0.000546
OTX1	2.932022	0.570123	0.194447	5.48E-05
C7orf31	2.327999	0.452749	0.19448	0.005898
MAGED4E	1.169793	0.227505	0.194483	0.339809
UCN	8.18715	1.595328	0.194858	0.014228
ZNF559-Z	0.052466	0.010227	0.194931	0.025293
TMEM253	0.316651	0.061748	0.195004	0.011921
KLHL28	1.806696	0.352335	0.195016	0.006979
TCEANC2	10.36377	2.022804	0.19518	0.000596
INTU	2.421748	0.472819	0.195239	0.001263

RND2	5.605603	1.094729	0.195292	0.003262
ZNF30	1.723941	0.336711	0.195315	0.001917
RBM33	23.54481	4.603848	0.195536	0.013691
THBS2	15.41566	3.015574	0.195618	0.060832
TYRP1	0.086383	0.016911	0.195766	0.155673
PHKG1	0.354852	0.069468	0.195767	0.025183
RHEBL1	11.20555	2.195355	0.195917	0.000583
USP45	4.512347	0.884049	0.195918	0.000954
IL17RD	3.237244	0.634404	0.195971	0.000461
SIGIRR	12.35399	2.42194	0.196045	0.002923
MFSD13A	7.806395	1.530827	0.196099	4.42E-05
FAM126B	2.492678	0.489406	0.196338	0.018458
RNF149	5.003244	0.9828	0.196433	0.001244
BHMG1	0.489071	0.096191	0.196682	0.134453
TARBP1	11.92086	2.346266	0.19682	0.00088
APOA1	0.446195	0.087961	0.197135	0.01847
FADS2	9.699179	1.912772	0.19721	0.018619
LOC10537	0.189695	0.03741	0.197213	0.015218
GOLGA8Q	0.089371	0.017633	0.197305	0.043702
SMC1A	47.79053	9.435499	0.197435	0.001819
RSPO2	0.281422	0.05559	0.197531	0.080117
APOBEC3I	1.318807	0.260739	0.197709	0.00094
FAM83A	0.033446	0.006614	0.197742	0.137122
EFNA3	12.805	2.532605	0.197782	0.005303
MRNIP	26.80332	5.301722	0.197801	7.80E-05
THUMPD2	11.08344	2.193353	0.197895	0.006958
CHEK1	22.39128	4.43518	0.198076	1.31E-05
BLM	21.1181	4.189279	0.198374	2.98E-05
C20orf96	20.95265	4.16283	0.198678	2.05E-05
LHX8	1.141837	0.226932	0.198743	0.020685
DEPDC7	12.19174	2.423461	0.198779	0.000232
LOC10798	0.404392	0.080391	0.198795	0.020723
NKX3-2	2.992559	0.595019	0.198833	0.007129
TIGD1	9.618152	1.912585	0.198852	0.002244
GMNN	36.68877	7.297005	0.198889	0.003845
LRIG3	6.143953	1.223841	0.199194	0.031123
AQP6	0.218224	0.043474	0.199219	0.090457
TXNIP	333.8378	66.5388	0.199315	0.003849
FLVCR1	5.107273	1.019001	0.19952	0.000122
RUNDC3B	1.953363	0.389903	0.199606	0.019286
SOCS7	7.218304	1.441034	0.199636	0.002998
TAF5L	10.63814	2.126514	0.199895	0.000897
BTK	0.158509	0.031724	0.200137	0.015594
ACVR1C	0.262554	0.052589	0.200299	0.018334
ADA	16.32055	3.269427	0.200326	0.005866
MYLK3	0.090317	0.018116	0.200585	0.069691
ZDBF2	5.644485	1.132258	0.200595	0.020557
HEATR5A	2.801362	0.562055	0.200637	0.000104
NRN1L	0.50179	0.10074	0.200761	0.257842
MCM10	24.20842	4.864233	0.200931	6.01E-05
LRRTM2	0.276604	0.055594	0.200987	0.035719
SPIN4	6.429008	1.293482	0.201195	0.014074
SGK494	3.172572	0.639126	0.201454	0.016516
SOX5	0.262235	0.052855	0.201557	0.006117
PIGL	4.16536	0.840292	0.201733	0.001616
HIST3H2A	16.22542	3.280876	0.202206	0.003734
HPGD	0.596873	0.12073	0.20227	0.078391
IRX6	0.642546	0.130033	0.202371	0.019196

GPRASP1	0.348399	0.070596	0.202631	0.01422
SLC29A4	8.20266	1.662989	0.202738	0.001158
ZSCAN30	13.48746	2.736803	0.202915	0.008204
SLC45A3	5.358451	1.087707	0.202989	0.001026
NBPF3	2.098314	0.426092	0.203064	0.002438
GABBR2	0.224672	0.04568	0.20332	0.031135
ZBTB44	5.238263	1.066188	0.203538	0.00114
ZFP14	1.759768	0.358714	0.203842	0.000195
ZAP70	0.052457	0.010698	0.20393	0.113135
LETMD1	26.63204	5.431338	0.20394	0.000619
HACE1	10.563	2.155575	0.204068	0.001835
RAB33B	4.604211	0.939605	0.204075	0.001338
CDKL1	0.510324	0.104369	0.204514	0.001056
SMARCC1	55.32476	11.3244	0.20469	0.001164
NEMP1	24.86308	5.090118	0.204726	0.004689
TMCC2	4.581443	0.938032	0.204746	0.000797
TMED6	0.422856	0.086637	0.204884	0.107601
PSAT1	240.0084	49.22619	0.205102	0.006339
SLC9A3R2	4.679701	0.960472	0.205242	0.016058
TMEM136	6.91906	1.42215	0.205541	0.000441
AKAP1	16.44019	3.380072	0.205598	0.000814
LTA4H	84.63907	17.4038	0.205624	0.001542
LCA5	3.1575	0.649321	0.205644	0.000673
NOTUM	2.251222	0.463047	0.205687	0.003809
GRIK3	0.008464	0.001742	0.205806	0.480078
AKAP17A	0.399559	0.08232	0.206027	0.06136
BPNT1	22.62462	4.662341	0.206074	0.001605
SCAPER	2.970397	0.613041	0.206384	5.11E-05
PHC1	9.109721	1.88072	0.206452	2.57E-05
GCNT2	1.206942	0.249294	0.20655	0.009608
EIF2S3B	10.52137	2.174724	0.206696	0.000374
FOXP3	0.217622	0.044998	0.206771	0.234635
DCAF16	21.88318	4.525864	0.206819	2.52E-05
IKZF5	6.031256	1.247482	0.206836	0.000135
IFT140	4.430929	0.916926	0.206938	7.52E-07
FNDC7	0.128362	0.026579	0.207063	0.156187
APAF1	5.470051	1.133328	0.207188	1.77E-05
PKN3	6.516431	1.35032	0.207218	0.001643
AGO2	4.896736	1.015115	0.207304	0.005513
LBP	1.805497	0.374601	0.207478	0.046328
CNNM3	6.639951	1.378996	0.207682	0.00092
EDRF1	13.7811	2.865784	0.20795	0.00117
C11orf54	5.952851	1.238005	0.207968	0.000244
ARFGEF3	3.181399	0.66241	0.208213	0.004372
IFRD1	34.74837	7.267151	0.209136	0.000526
GPATCH2	3.372212	0.705481	0.209204	5.24E-06
ABCB10	7.644362	1.59944	0.209231	0.001537
ZNF397	9.013215	1.88597	0.209245	3.76E-05
ZBTB34	5.113315	1.070915	0.209437	0.000685
TMEM206	16.81631	3.524262	0.209574	0.000629
GTPBP1	29.30253	6.145408	0.209723	0.007202
TMC5	0.153604	0.032237	0.209873	0.139845
PPM1J	9.909662	2.082252	0.210123	0.021521
ZNF296	1.740615	0.366584	0.210606	0.002501
HOXA1	7.770393	1.638084	0.210811	0.010005
IPO9	30.72249	6.482047	0.210987	0.003577
TMEM56-	0.102101	0.021542	0.210989	0.12163
MUSTN1	1.599407	0.337642	0.211105	0.122284

SETD5	20.5054	4.33112	0.211219	0.00266
GLRA4	0.035222	0.007442	0.211288	0.082889
CLSTN3	14.68164	3.104207	0.211435	0.00148
TTLL3	0.806568	0.170571	0.211477	0.014891
RIPPLY2	2.223903	0.470527	0.211577	0.011613
MUC5B	0.02614	0.005531	0.211587	0.091921
AFAP1L1	2.268506	0.480371	0.211757	0.001272
SLFNL1	0.044639	0.009477	0.212298	0.392618
TMEM198	4.030146	0.856275	0.212468	0.035862
TIGD7	4.813089	1.022878	0.21252	1.30E-05
TIAM1	3.169707	0.675486	0.213107	0.000855
C3orf67	1.428635	0.304511	0.213148	0.01166
DNAI2	0.024025	0.005123	0.21322	0.314955
TRIM54	0.730129	0.155726	0.213285	0.002135
ACRBP	0.241672	0.051576	0.213411	0.085
PRR3	18.66783	3.991983	0.213843	0.000868
RAB26	3.213672	0.687861	0.214042	0.008828
ANPEP	0.604601	0.129573	0.214311	0.124121
DEAF1	32.99498	7.074346	0.214407	7.11E-06
FAM222A	0.376399	0.080718	0.214449	0.023303
OTP	0.083893	0.017991	0.214449	0.069795
ENDOU	0.155207	0.033291	0.214491	0.159296
TMPO	36.70991	7.875684	0.214538	0.000198
DNAI1	0.121653	0.026105	0.214586	0.065233
GPR153	4.280509	0.919599	0.214834	1.75E-05
LOC10537	0.024166	0.005196	0.214996	0.014698
XRCC3	33.39792	7.181008	0.215014	0.004836
L3MBTL3	4.330196	0.931401	0.215094	0.000459
MYO1A	0.080865	0.017404	0.215222	0.25526
GJA5	0.106115	0.022844	0.215275	0.041586
HSPA1A	1.87487	0.403741	0.215343	0.220664
ILDR2	0.664152	0.143049	0.215387	0.000661
ARMH1	0.291891	0.062898	0.215486	0.045874
MEX3B	4.0015	0.862315	0.215498	0.003476
DHH	0.095619	0.020606	0.215498	0.255559
CP	0.486408	0.104829	0.215516	0.004806
SLC25A53	0.364629	0.078649	0.215696	0.010281
GATAD2B	15.28969	3.298371	0.215725	0.003607
ATP11C	11.27369	2.43223	0.215744	0.002745
TTC13	8.642794	1.864886	0.215774	0.000861
EVPL	0.01114	0.002404	0.215811	0.486092
XRCC2	10.75774	2.322555	0.215896	0.0637
GPR158	0.130194	0.028109	0.215901	0.006027
ZKSCAN1	14.6275	3.158467	0.215927	0.008841
HOXC8	2.382781	0.51489	0.216088	7.49E-05
EIF2S3	240.7415	52.0253	0.216104	0.000333
CATSPERG	0.261443	0.056544	0.216277	0.008835
TBC1D32	2.118551	0.458448	0.216397	7.54E-05
RBM5	44.72293	9.681298	0.216473	0.011901
ZNF107	2.501035	0.541622	0.216559	0.017333
P2RY1	4.189741	0.907525	0.216607	2.54E-05
TBC1D28	0.016149	0.003499	0.21668	0.224228
ZNF280B	3.878893	0.840747	0.216749	0.002032
LMBR1L	9.523567	2.065305	0.216863	0.001906
C10orf105	0.011862	0.002575	0.217071	0.224272
CLIC6	0.01367	0.002967	0.217071	0.224272
PAOX	0.98514	0.214061	0.21729	0.0199
CEP295NL	0.035075	0.007622	0.217295	0.086832

ESRRG	0.224652	0.048836	0.217386	0.011141
XPO5	28.81477	6.265905	0.217455	0.003519
HR	0.470585	0.102433	0.217672	0.033209
FRG2B	0.082885	0.018045	0.217706	0.322681
NOS1	0.023862	0.005198	0.217841	0.031296
PAPOLG	5.233244	1.140822	0.217995	0.000159
GATA3	2.474889	0.539865	0.218137	0.024405
FAM89A	23.27864	5.078666	0.218169	0.000701
PTPN7	0.015767	0.003443	0.218345	0.487626
KCNG2	0.026859	0.005871	0.21858	0.115549
CASTOR3	2.515551	0.54986	0.218584	0.001979
RAB3A	11.29071	2.468195	0.218604	0.029785
SLC7A11	7.646875	1.671845	0.218631	0.05053
RBM12B	8.250329	1.804017	0.21866	0.001744
UPK1B	0.107973	0.023633	0.218875	0.285061
RAB9B	1.759636	0.385291	0.21896	0.004295
ATF4	162.1077	35.5438	0.21926	0.000258
ASIC1	11.23733	2.464073	0.219276	0.00119
TROVE2	9.677301	2.124023	0.219485	4.20E-06
FXYD1	2.068551	0.454044	0.219499	0.007114
CRAMP1	6.016563	1.320892	0.219543	0.002867
LOC10537	0.03885	0.008534	0.219653	0.22651
DDX21	78.77643	17.31034	0.21974	0.002525
CMTM4	11.67396	2.56786	0.219965	0.00056
GRAMD4	7.979117	1.756209	0.220101	9.13E-06
ELK4	10.16015	2.237635	0.220236	0.079756
BBS5	4.96927	1.094735	0.220301	0.000893
DOCK6	8.031512	1.770499	0.220444	0.000239
BAZ2A	21.29876	4.696139	0.220489	0.001765
TCN1	0.047791	0.01054	0.220546	0.073678
SLC39A10	6.814865	1.504375	0.220749	0.000655
ARHGAP3	2.843229	0.62781	0.220809	0.003996
ZNF223	1.018112	0.224933	0.220931	0.02377
GAGE1	0.074293	0.016415	0.220944	0.083496
RAD54L	43.67649	9.657854	0.221122	0.000921
PWP2	1.334511	0.295242	0.221236	0.018586
UPK3B	0.061541	0.013618	0.221279	0.121846
ARID3A	9.597893	2.124742	0.221376	0.000782
PHF8	13.84666	3.067686	0.221547	0.000657
C19orf71	1.361928	0.301832	0.221621	0.040385
SOWAHD	0.468474	0.103874	0.221728	0.179564
EXOC8	10.33216	2.291741	0.221807	0.000109
HEATR5B	7.487178	1.660883	0.22183	0.001886
CLNS1A	161.4193	35.8139	0.221869	0.001388
RORB	0.411736	0.091461	0.222135	0.039899
KIF21B	2.509582	0.557529	0.22216	0.002221
LBR	30.34392	6.742322	0.222197	0.001269
SRL	0.02429	0.005397	0.222208	0.189703
DNMT3A	2.495036	0.554662	0.222306	5.71E-05
GPSM3	2.003075	0.446195	0.222755	0.004618
EPB41L5	5.094996	1.135983	0.222961	0.001656
UCHL5	17.6224	3.929245	0.222969	0.003346
STAG2	44.52178	9.927026	0.22297	0.000456
FRS3	2.256285	0.503764	0.223271	3.17E-05
ZBTB5	10.37764	2.318245	0.223389	0.009591
PTCH1	2.333221	0.521226	0.223393	0.000186
DDX11	19.74946	4.414313	0.223516	0.004239
ZNHIT6	8.982075	2.007795	0.223534	0.001517

LAPTM4B	171.0551	38.27099	0.223735	0.000233
SCGB2B2	0.134344	0.030064	0.223784	0.119055
ZNF224	4.607559	1.031538	0.22388	0.01684
CCDC177	0.035521	0.007959	0.224058	0.282259
TGFBR3	5.27305	1.181511	0.224066	0.00026
TMEM200	0.18	0.040334	0.224075	0.015787
C20orf204	0.467587	0.104802	0.224134	0.052093
ACP6	5.076126	1.137844	0.224156	0.001308
PRR14	59.32116	13.2985	0.224178	0.002045
BIN2	0.022934	0.005142	0.224219	0.321623
SIPA1L2	11.70545	2.624757	0.224234	0.003575
ERN2	0.053884	0.012088	0.224333	0.439683
SCAMP5	6.717259	1.507155	0.224371	4.14E-05
SWT1	1.949096	0.437567	0.224498	2.09E-05
CD248	0.028754	0.006457	0.224552	0.491403
GNRH1	2.145965	0.482036	0.224624	0.04909
ZNF184	7.335074	1.649225	0.224841	0.001787
GCFC2	3.854544	0.86684	0.224888	0.008359
MTA1	58.88861	13.24626	0.224938	1.04E-05
LYG1	1.469383	0.330621	0.225007	0.005383
RPS18	5009.644	1127.22	0.22501	0.000353
PROSER1	26.28081	5.920286	0.22527	0.001377
TIPRL	58.22004	13.12475	0.225434	0.000849
OPRL1	0.831684	0.187654	0.225631	0.000156
HSD17B3	0.103986	0.023465	0.225658	0.013559
KIAA0355	2.854656	0.644686	0.225837	0.013655
GFOD1	2.198813	0.496806	0.225943	7.50E-05
DCLRE1A	11.4533	2.588043	0.225965	0.020153
H1FX	323.9228	73.21669	0.226031	0.000463
KIAA1147	12.13588	2.745145	0.226201	0.000661
DHTKD1	20.9022	4.72959	0.226272	0.000429
PNN	109.0576	24.68806	0.226376	0.000738
CRISPLD2	0.256271	0.058053	0.22653	0.128654
ZNF266	12.0347	2.727612	0.226646	0.010578
ADGRL2	5.728292	1.298514	0.226684	0.008247
DDAH1	11.65743	2.643751	0.226787	0.002917
FABP7	0.485685	0.110173	0.22684	0.02651
HMBOX1	3.332348	0.756045	0.226881	0.024273
GP1BB	6.877077	1.563584	0.227362	0.008208
TOP3B	11.18082	2.542877	0.227432	0.005657
DNAH6	0.293706	0.066801	0.227442	0.102891
VSIR	0.238476	0.054302	0.227705	0.050072
FUOM	4.937547	1.124408	0.227726	0.013453
SPRTN	9.768249	2.225328	0.227812	0.000439
PRRT2	3.848445	0.878061	0.22816	0.015135
BLNK	0.09746	0.022248	0.228283	0.015797
LATS1	5.475762	1.250579	0.228384	0.003385
URB2	5.211526	1.191056	0.228543	0.007601
MCOLN2	3.722781	0.852009	0.228864	0.000196
SPATA6L	0.633241	0.144961	0.22892	0.006227
GLO1	274.2561	62.78908	0.228943	0.003318
ABAT	1.558957	0.356992	0.228994	0.000519
CABYR	6.394063	1.466024	0.229279	3.42E-05
ARF1	359.1417	82.43485	0.229533	0.002091
RAB4A	20.81008	4.777943	0.229598	0.004029
TBX6	1.91065	0.439346	0.229946	0.004592
BCOR	13.69039	3.148622	0.229988	0.001642
GRK4	1.446762	0.332887	0.230091	0.003554

BRWD3	3.36848	0.775623	0.230259	0.000123
SIX4	6.416535	1.477595	0.230279	0.00093
PITX2	2.840571	0.65446	0.230397	0.005152
PKMYT1	31.32129	7.221752	0.23057	1.82E-05
POLR1D	48.93227	11.29106	0.230749	0.004382
UNC79	0.052545	0.01213	0.230843	0.173013
MAGEA3	2.334339	0.538971	0.230888	0.010041
KNTC1	28.27142	6.527754	0.230896	0.000697
EMILIN2	4.068265	0.939633	0.230967	0.00697
RPIA	32.46572	7.505026	0.231168	6.50E-05
MYO6	9.003082	2.081227	0.231168	0.001178
CCDC17	0.293373	0.067869	0.231339	0.075004
HDHD3	8.907235	2.060602	0.23134	0.002925
ZFC3H1	15.48778	3.589108	0.231738	0.007839
SYN3	0.493112	0.114424	0.232046	0.000647
NBPF11	3.347619	0.777044	0.232118	0.001835
CAPN3	0.286304	0.066468	0.232159	0.117359
CASP8AP2	11.98945	2.784032	0.232207	0.000275
HSPA1B	78.59102	18.27065	0.232478	0.083212
CCSAP	8.380625	1.950394	0.232727	8.94E-05
VENTX	0.109227	0.025439	0.232901	0.237344
POMK	1.258628	0.293276	0.233012	0.006328
ARIH2OS	2.769731	0.6457	0.233127	0.020309
RNF180	0.040672	0.009484	0.233181	0.052905
ZNF814	0.882663	0.205884	0.233253	0.009421
CTC1	4.364452	1.018454	0.233352	0.004832
POU6F1	0.766799	0.178952	0.233376	0.001314
CLDN1	1.284686	0.299963	0.233491	0.005248
FRAT2	12.40278	2.896113	0.233505	0.0029
TAS2R14	0.671875	0.157239	0.234031	0.044827
BMPR1B	0.901816	0.211138	0.234125	0.002056
NKRF	15.52067	3.63468	0.234183	0.00132
CHCHD10	115.0568	26.94927	0.234226	0.001528
CCDC73	0.12023	0.028164	0.234251	0.15022
WSB1	31.21161	7.314143	0.23434	0.007085
KLHL11	6.730728	1.577691	0.234401	0.015653
ATP6V1G2	8.358019	1.960891	0.234612	0.000459
DDRGK1	75.18998	17.65814	0.234847	0.000793
ERMP1	10.0965	2.372971	0.235029	0.000298
NOP53	218.9389	51.4649	0.235065	0.001919
GINS3	15.61459	3.671117	0.235108	0.005666
ABCC10	5.577432	1.312064	0.235245	0.004598
FEM1B	27.57928	6.489363	0.235298	0.000115
ZBTB41	2.748167	0.647226	0.235512	0.000368
KLHDC3	67.49579	15.91168	0.235743	5.59E-05
NFYA	12.88204	3.037237	0.235773	0.000339
TXNRD3	4.777468	1.127613	0.236027	0.011415
OLIG2	2.633946	0.621916	0.236116	0.001269
REM1	0.284793	0.067246	0.236124	0.072371
ZCCHC3	19.35131	4.572454	0.236287	0.000128
TMEM170	2.885055	0.681886	0.236351	0.001885
CDNF	1.337149	0.316222	0.236489	0.030968
PLS3	187.4868	44.36138	0.236611	0.011702
TMEM176	0.109217	0.025843	0.236616	0.43082
UBE2T	181.0364	42.83831	0.236628	0.000587
PTPRH	2.461478	0.58285	0.236789	0.001288
C4orf19	0.030442	0.007211	0.236874	0.217675
PM20D2	3.445106	0.816311	0.236948	0.01175

TEKT4	0.088887	0.021067	0.237013	0.105904
NOL4L	2.975992	0.706072	0.237256	0.003529
BEX5	1.293014	0.306981	0.237415	0.188949
EDEM3	8.043439	1.91063	0.237539	0.006098
UCP2	20.03426	4.762984	0.237742	0.053739
PEX1	10.45483	2.487091	0.237889	0.013288
NUF2	61.49715	14.6351	0.23798	0.000323
CDH3	1.025326	0.244088	0.238059	0.081369
RAB2B	16.40777	3.906583	0.238094	0.001007
FKBP1B	5.422526	1.292134	0.23829	0.005215
MARK1	2.746878	0.656297	0.238925	0.001501
TSNARE1	1.190793	0.284669	0.239058	0.006456
DNAJC27	1.732949	0.415429	0.239724	0.001086
ZNF496	6.048617	1.45006	0.239734	3.02E-05
SEPSECS	2.970126	0.712345	0.239837	0.009555
SNX5	129.6367	31.10227	0.239919	0.008962
CATSPERE	0.221068	0.053053	0.239985	0.002137
MMP15	7.085659	1.701489	0.240131	0.008648
ZCCHC11	12.97627	3.117931	0.240279	0.001358
LIG3	11.02175	2.65	0.240434	0.001548
HMGN3	58.28295	14.01782	0.240513	0.001894
NRXN3	0.629907	0.151725	0.240868	0.026754
FAXDC2	2.196464	0.529146	0.240908	0.000178
LOC10013	0.106772	0.025726	0.240947	0.209192
DDX59	12.48691	3.010958	0.241129	0.000641
FNBP4	30.77612	7.424966	0.241257	0.003005
IL2RG	0.13447	0.032448	0.241305	0.025562
DBF4B	6.44101	1.554975	0.241418	0.000558
FAM122C	2.943259	0.710669	0.241457	7.17E-06
TMIE	0.177768	0.042956	0.241641	0.443881
HELB	1.116882	0.269903	0.241657	0.001443
GGA2	21.77425	5.264121	0.241759	4.23E-05
CBL	9.52026	2.304578	0.242071	0.0062
PLXNB1	1.885979	0.457064	0.242348	0.015718
TEX13B	0.106177	0.025737	0.242395	0.060304
PBX2	37.35662	9.055165	0.242398	2.36E-05
CFAP43	0.385666	0.093534	0.242527	0.013257
SH3BP2	5.484846	1.331079	0.242683	0.008876
ARID4B	11.21479	2.724557	0.242943	0.001535
FZD3	1.518885	0.369446	0.243235	1.87E-05
ECT2L	0.022539	0.005483	0.243263	0.315016
FAM43A	1.403754	0.341516	0.243288	0.004436
CDH22	0.072016	0.017525	0.24335	0.348444
PJVK	0.890233	0.216728	0.243451	0.044364
TRIM13	4.423476	1.078099	0.243722	0.003258
LRP1B	0.476386	0.116213	0.243946	0.047318
LOC10798	0.118441	0.028896	0.243967	0.016269
TRPV1	2.313946	0.564534	0.24397	0.011509
EPCAM	0.982099	0.239803	0.244174	0.037623
IMPG1	0.036917	0.009025	0.244462	0.052592
TFEC	0.016217	0.003964	0.244462	0.052592
EFCAB12	0.129726	0.031722	0.244531	0.003483
PRSS22	0.088332	0.021629	0.244864	0.161985
DOCK3	4.74971	1.163174	0.244894	0.072696
HINFP	8.141286	1.99426	0.244956	0.000409
INTS6L	5.265528	1.290666	0.245116	0.003589
ELL3	4.092552	1.00355	0.245214	0.008919
TUBGCP6	13.05809	3.203577	0.245333	0.000656

DEK	137.7131	33.78956	0.245362	0.00014
PPP1R13B	4.951456	1.216029	0.24559	0.001974
SAMD13	0.834762	0.205031	0.245616	0.059807
CCDC138	4.370234	1.073745	0.245695	0.005526
CORO6	5.528076	1.359168	0.245866	0.000685
CEBPG	49.54886	12.18569	0.245933	0.000117
C3orf20	0.109732	0.027	0.246058	0.118658
ZBTB20	2.49972	0.615216	0.246114	0.144601
GABRA2	0.01279	0.003148	0.246156	0.111028
MGME1	27.83084	6.856085	0.246348	0.002023
ZUP1	23.96961	5.908129	0.246484	0.008025
GH2	0.098539	0.024296	0.246564	0.163166
ANKRD13	8.158587	2.011888	0.246598	0.000481
ATXN7L3E	34.57577	8.528299	0.246655	0.004175
DICER1	3.585914	0.884515	0.246664	0.007468
DNM3	0.229203	0.05654	0.246683	0.000244
WNT2B	0.478871	0.118149	0.246724	0.019893
RGPD2	0.012197	0.00301	0.246747	0.224902
PHF10	39.86271	9.848097	0.24705	2.49E-05
PHLDB2	4.96201	1.225928	0.247063	0.000401
NARS2	13.24511	3.274336	0.247211	0.007644
RAB17	0.14113	0.034892	0.247232	0.059014
LTA	0.235913	0.058387	0.247495	0.064312
C4B	0.024417	0.006044	0.247551	0.021161
ADCY9	5.016098	1.242097	0.247622	0.000492
CAPS2	1.125926	0.278845	0.247659	0.000101
TUFT1	7.996118	1.982519	0.247935	0.005499
VAMP7	1.847261	0.458062	0.247968	0.380269
UBR2	6.845348	1.697452	0.247972	0.002334
RPL39	5158.301	1279.501	0.248047	0.000742
ZSCAN10	0.089836	0.022286	0.248072	0.34937
DARS2	26.89021	6.671672	0.248108	0.022384
EGF	0.491765	0.122037	0.248161	0.008736
LPAR6	0.200576	0.049799	0.248282	0.057222
TRIM11	27.11853	6.734745	0.248345	0.0006
ANKRD24	0.924679	0.2297	0.24841	0.008346
SERAC1	6.111544	1.519182	0.248576	0.000503
FMN2	2.307284	0.573897	0.248733	0.001604
TNNI1	0.021459	0.00534	0.248844	0.128253
C2CD5	12.57063	3.128588	0.248881	5.44E-05
FUZ	32.19254	8.015148	0.248975	0.021675
ENTPD8	0.207159	0.051609	0.249128	0.002682
LY6G5B	12.68717	3.164198	0.249401	0.020102
CILP	0.04008	0.010005	0.249636	0.238159
DDX19B	14.71189	3.67349	0.249695	0.010743
MISP	0.142142	0.035499	0.249746	0.097241
HAUS4	51.86669	12.95979	0.249867	0.000838
PPAT	20.47168	5.11796	0.250002	0.000511
EVL	12.71274	3.179392	0.250095	0.001203
DRC7	0.149182	0.03733	0.250232	0.024339
DMTF1	12.85539	3.216861	0.250234	0.00023
EIF1B	229.5278	57.46087	0.250344	0.002429
ISG20	0.621394	0.15559	0.250389	0.01524
FANCD2	30.21581	7.566072	0.250401	7.22E-06
RIMKLB	6.978049	1.747941	0.250491	0.001923
N4BP2L2	17.13702	4.292778	0.250497	9.06E-05
USPL1	8.441178	2.117695	0.250877	1.64E-05
SRPK1	49.84673	12.50569	0.250883	2.76E-06

NUFIP2	24.84636	6.234992	0.250942	0.03166
CHGB	0.342143	0.085898	0.251059	0.010579
ZFP90	11.44221	2.873367	0.25112	0.000481
KIAA1324l	4.571245	1.149142	0.251385	0.000664
RPS6KA5	1.792862	0.450705	0.251389	0.004468
PPCS	6.84749	1.721913	0.251466	0.011008
TSPAN12	5.254024	1.321497	0.251521	0.057945
ARL17B	0.697897	0.175544	0.251532	0.021193
PUS3	14.28877	3.596287	0.251686	4.35E-05
ITGA4	2.520939	0.634557	0.251714	0.014449
C6orf99	0.473192	0.119171	0.251846	0.035509
INSM2	0.044122	0.011114	0.251886	0.259144
METTL17	60.08722	15.13825	0.251938	0.001091
SLC19A2	9.278746	2.339245	0.252108	0.000114
HSPB2	0.22715	0.057288	0.252202	0.201026
BST1	0.137607	0.03472	0.252313	0.035321
MYL10	0.120438	0.030389	0.25232	0.293992
VPS36	10.35464	2.617349	0.252771	0.000421
THBS3	7.163109	1.810792	0.252794	0.004097
WDR17	1.679283	0.424557	0.25282	0.003441
RALGPS1	1.263641	0.319693	0.252994	0.002339
PMS2	5.833807	1.476222	0.253046	0.001631
PRIM1	57.53056	14.55938	0.253072	0.043964
IQCB1	33.32754	8.434663	0.253084	0.011302
C20orf202	0.081349	0.020594	0.253154	0.116775
SETDB2	1.282256	0.324727	0.253247	0.012521
SIDT2	10.68968	2.707281	0.253261	0.000615
PSMC3IP	77.14089	19.53881	0.253287	0.008476
CLCN5	2.240853	0.568033	0.25349	0.046891
ZBTB33	17.54476	4.448075	0.253527	2.05E-06
GPR78	0.031104	0.007888	0.253612	0.323337
CSNK1E	1.542327	0.391499	0.253836	0.004509
TMEM190	0.246374	0.062579	0.253999	0.01747
NPIPB8	0.045081	0.011453	0.254051	0.267748
HOXD1	0.591123	0.150179	0.254057	0.0069
ARHGEF37	1.408351	0.357818	0.254068	0.030993
ALPL	2.373428	0.604371	0.25464	0.026702
POLR3K	88.19603	22.46051	0.254666	0.003061
ZNF692	13.72649	3.497796	0.254821	0.001174
CHODL	0.092045	0.023462	0.254892	0.147246
LOC28371	0.052803	0.013461	0.254921	0.265322
TMED8	11.15513	2.844393	0.254985	9.70E-05
FIGNL1	6.113718	1.558924	0.254988	0.000805
SNAP47	11.55389	2.946163	0.254993	0.000355
EBPL	47.43845	12.09806	0.255027	0.010376
PRRC2C	35.25222	8.990746	0.255041	0.001456
NEB	0.011672	0.002977	0.255084	0.018469
TBC1D3F	0.155169	0.03961	0.255267	0.236368
JARID2	7.409526	1.891861	0.255328	2.31E-06
SFXN2	2.69497	0.688578	0.255505	0.041626
ICAM4	1.284444	0.328205	0.255523	0.011532
CALML4	7.398273	1.891533	0.255672	0.001964
ODR4	12.25965	3.135895	0.25579	0.000223
GKAP1	10.90255	2.789466	0.255854	3.29E-05
OPHN1	2.090271	0.534965	0.255931	9.30E-05
POLR2A	54.51635	13.97889	0.256416	0.002604
RNF2	9.03297	2.316631	0.256464	0.001127
TCP11L2	1.619038	0.415257	0.256484	9.27E-05

XRN1	6.304413	1.617244	0.256526	0.002326
CREBRF	3.339524	0.856757	0.256551	0.011818
CD55	26.50447	6.807665	0.25685	0.000582
ELMOD1	4.239458	1.08895	0.256861	0.000664
SFTPA2	0.068643	0.01764	0.256983	0.170331
ZMYM2	5.016177	1.289216	0.257012	0.000958
POLE	27.82054	7.151007	0.257041	0.00142
SPATA17	2.087926	0.536727	0.257062	0.1063
FANCE	10.12312	2.60234	0.257069	0.017952
NCAPD3	31.38173	8.067453	0.257075	9.24E-05
MED23	12.36341	3.178596	0.257097	0.001145
PYCR1	91.11483	23.43238	0.257174	0.000397
CCDC186	2.149774	0.553018	0.257245	0.000194
FAM72A	2.475244	0.637419	0.257518	0.039776
BLOC1S5	13.12245	3.381046	0.257653	0.003241
S100B	4.796536	1.235865	0.257658	0.054436
C16orf87	4.375923	1.127918	0.257755	9.75E-05
ZNF705E	0.111948	0.028864	0.257835	0.314303
C18orf21	31.77864	8.195409	0.257891	0.003754
ZBTB26	4.506568	1.16246	0.257948	0.003302
C1orf74	3.884557	1.002698	0.258124	0.01878
PKN2	10.50391	2.712539	0.258241	0.000308
TPR	35.94938	9.296695	0.258605	0.001914
CLVS2	0.176856	0.045747	0.258665	0.000133
SDR39U1	38.92358	10.06941	0.258697	3.16E-05
RPL12	3413.89	883.1646	0.258697	0.000311
ANKRD20	0.090721	0.023484	0.258859	0.334952
NHSL2	0.172208	0.044614	0.25907	0.010647
C2orf72	1.873522	0.485755	0.259274	0.001555
NPIPB13	0.242126	0.062814	0.259426	0.008273
ZPLD1	0.045446	0.011794	0.259519	0.212171
CHUK	15.90975	4.12992	0.259584	0.00021
TMEM150	1.967498	0.51077	0.259604	0.024366
ZNF608	4.158096	1.079903	0.259711	0.000831
GNRH2	0.059418	0.015433	0.259735	0.018449
CFAP69	0.977461	0.253945	0.259801	0.002193
PNISR	31.08355	8.082106	0.260012	0.012655
FUT10	1.724342	0.448879	0.260319	0.001079
YPEL4	3.555309	0.92561	0.260346	0.12247
RNPEP	47.42978	12.35182	0.260423	0.000153
ALLC	0.027598	0.007197	0.260789	0.277454
ACBD3	41.36595	10.79034	0.260851	0.013921
WDHD1	24.07395	6.280615	0.260888	2.66E-05
BRMS1L	6.639877	1.733014	0.261001	3.74E-05
SIRT5	3.642657	0.951496	0.261209	0.006707
PLEKHA8	3.838223	1.003209	0.261373	0.002413
ABCB4	0.99631	0.260409	0.261374	0.018957
PRPF4B	10.65812	2.787183	0.261508	0.00175
DBNDD1	11.99644	3.138432	0.261614	0.000648
ZKSCAN3	2.448636	0.640855	0.261719	0.012731
CCNT2	4.291037	1.123585	0.261845	0.001654
RSRC2	89.9429	23.56431	0.261992	6.13E-05
KDM6B	7.285719	1.90927	0.262057	0.002401
F11R	15.74454	4.126923	0.262118	0.001086
ARHGEF3	0.043084	0.0113	0.262283	0.263592
AGAP6	7.093172	1.860596	0.262308	0.006718
C1orf116	0.067958	0.017828	0.262339	0.101354
LHX2	2.63145	0.690663	0.262465	0.005547

RAB3GAP2	10.68575	2.805266	0.262524	0.001159
EML6	1.437261	0.377768	0.262839	0.001002
RFX3	1.288543	0.338725	0.262875	0.01181
TRRAP	14.39808	3.785457	0.262914	0.013311
ADAMTS7	3.160679	0.831184	0.262977	0.006667
HEXDC	4.032991	1.06062	0.262986	0.011814
PAXX	63.2675	16.64348	0.263065	5.00E-05
ZDHHC17	6.618531	1.742014	0.263203	0.000177
DHRS12	2.998403	0.790468	0.26363	0.014552
CADM4	12.70737	3.350716	0.263683	0.001775
BMI1	0.454571	0.119977	0.263935	0.004442
TBKBP1	6.929818	1.82954	0.26401	1.81E-05
VEZT	10.61731	2.803841	0.264082	0.000235
MYLIP	4.418269	1.166817	0.264089	0.00145
ATAD3C	0.048164	0.012722	0.26413	0.128054
TMEM81	10.16259	2.68504	0.264208	0.028175
SUGCT	1.188572	0.31444	0.264553	0.023807
C9orf72	7.939034	2.100369	0.264562	0.000449
NKAPD1	16.68121	4.416665	0.264769	0.003277
DDX39B	235.5083	62.394	0.264933	0.008471
SLC25A35	7.005466	1.856394	0.264992	0.000804
C20orf194	2.598502	0.688585	0.264993	5.27E-05
SESN2	37.50011	9.944983	0.265199	0.003812
TMEM151	0.168561	0.044704	0.265209	0.241758
KLHL31	0.086537	0.022952	0.26523	0.002926
ESCO1	14.32389	3.800145	0.265301	0.000185
TRAPPC2	13.33913	3.541027	0.265462	0.000773
LOC10192	0.099376	0.026395	0.265606	0.067551
WNT10B	1.830823	0.486445	0.265697	0.028405
TLE4	15.45501	4.108765	0.265853	0.015345
ARHGAP2	0.526927	0.1401	0.265882	0.054064
LOC10798	0.073032	0.019427	0.266008	0.047259
XRRA1	2.206792	0.587798	0.266359	0.000159
NVL	18.4282	4.912113	0.266554	0.003359
LOC28550	0.047961	0.012788	0.266643	0.221129
PRR5L	0.800364	0.21361	0.266891	0.032951
RRNAD1	16.52375	4.416809	0.267301	0.00044
SLC39A2	0.131256	0.035103	0.267437	0.369831
NBPF10	0.231529	0.061932	0.267491	0.085725
CCDC171	0.452896	0.121157	0.267516	0.024935
PRRC2B	40.39285	10.8065	0.267535	0.002326
FAM160A1	1.274459	0.341016	0.267577	0.001436
SIM1	0.735733	0.196883	0.267602	0.00713
RBM41	7.686609	2.057422	0.267663	0.00041
EMCN	0.032586	0.00873	0.267916	0.180007
TRAPPC8	12.64425	3.390125	0.268116	0.002528
UNC13D	0.124193	0.033306	0.268183	0.145688
CPSF4L	0.094448	0.02533	0.268193	0.227425
CCL27	0.656002	0.175937	0.268196	0.021795
GCNT4	0.197111	0.052874	0.268246	0.094912
FBXW10	0.073495	0.019728	0.268431	0.162761
SKP2	18.77069	5.040887	0.268551	0.004553
HYKK	4.914111	1.320174	0.26865	0.000598
RIBC1	1.172439	0.315153	0.268801	0.02876
INTS7	12.23977	3.292194	0.268975	0.003602
TSPYL2	26.5484	7.142817	0.269049	0.000863
VSIG8	0.33762	0.090845	0.269075	0.242936
CLDN9	0.120959	0.032559	0.269171	0.18284

ZNF445	6.286659	1.69492	0.269606	0.002036
JAKMIP3	0.030059	0.008111	0.269829	0.184922
PCDHGA1	0.184522	0.049791	0.26984	0.026575
ZNF343	6.461286	1.743924	0.269903	0.000302
CHORDC1	21.25764	5.737654	0.26991	0.000289
CORO7	1.604183	0.433054	0.269953	0.00089
NUDT17	2.217572	0.598691	0.269976	0.006919
FAM161A	5.143545	1.388636	0.269976	4.99E-05
NAP1L3	3.254695	0.878869	0.270031	0.085865
FANCB	3.538227	0.955914	0.270168	0.014077
FBXO24	0.221784	0.059924	0.27019	0.079647
CA11	17.32255	4.684371	0.270421	0.000504
ZC3H8	9.295964	2.514543	0.270498	0.003278
RBM15	14.93484	4.040564	0.270546	0.007973
TADA1	15.60031	4.222485	0.270667	0.000319
TAF11	26.18949	7.088865	0.270676	0.000188
TMEM63A	9.555575	2.587318	0.270765	0.012154
DSCC1	17.2104	4.663583	0.270975	0.010635
NGEF	7.812428	2.117815	0.271083	0.004434
YARS	279.2883	75.7187	0.271113	0.008889
TRIM46	1.27695	0.346307	0.271199	0.002478
HMGB2	344.1983	93.34714	0.271202	0.000638
JUN	432.2264	117.3429	0.271485	0.001891
SASS6	8.513505	2.311679	0.271531	0.001221
JMY	5.734334	1.558014	0.271699	6.33E-05
DCAF17	4.052399	1.101447	0.271801	0.000989
SPSB3	23.78351	6.468716	0.271983	0.010851
NPIPB6	0.276923	0.075324	0.272002	0.049831
CENPC	6.697702	1.821921	0.272022	0.000217
SDHAF4	30.42547	8.283335	0.27225	0.008616
ADHFE1	0.510544	0.139088	0.27243	0.046831
ITGA6	7.539461	2.055127	0.272583	0.007495
DENND2A	1.929996	0.526627	0.272864	0.005696
SIRT1	10.90319	2.97624	0.27297	9.12E-06
ZBTB11	9.797326	2.674495	0.272982	0.006998
ZC3H6	2.921935	0.797705	0.273006	0.009485
STAP2	0.729882	0.199327	0.273094	0.133322
ENPP5	1.681353	0.459417	0.273243	0.002893
MAGEA11	0.536428	0.14664	0.273363	0.007246
ASPRV1	0.38376	0.105058	0.27376	0.073095
PCDH9	2.178947	0.597178	0.274067	0.000779
C2CD3	5.074375	1.391171	0.274156	0.000212
TMEM119	4.077451	1.118061	0.274206	0.042932
SSC4D	0.818551	0.224622	0.274415	0.036322
TBC1D22E	9.46346	2.59804	0.274534	0.001229
PATZ1	10.8462	2.978838	0.274644	0.017364
PPP1R3E	3.435167	0.943945	0.274789	0.001664
ABCD4	20.90574	5.752613	0.275169	0.006292
VILL	1.070297	0.294718	0.275361	0.008348
HMG20A	27.90814	7.686518	0.275422	0.000527
BICDL1	0.446794	0.123187	0.275714	0.152783
AGTR1	1.251018	0.344934	0.275722	0.007767
AMN1	3.891308	1.073285	0.275816	0.00528
GUCY1A2	0.128401	0.035429	0.275923	0.036067
MAML3	0.92476	0.255196	0.275959	0.002727
PAGE5	0.552267	0.152425	0.275998	0.063684
GMDS	6.792509	1.875033	0.276044	0.009269
OCA2	0.346222	0.095743	0.276537	0.107587

FSTL3	8.32963	2.303773	0.276576	0.061432
PTAR1	10.33584	2.858984	0.276609	0.028407
ARHGAP1	5.468709	1.513514	0.276759	0.000709
RCBTB1	7.569305	2.095085	0.276787	0.000105
GAL3ST4	1.429632	0.396184	0.277123	0.027655
ATP8A2	0.095266	0.026407	0.277197	0.004812
MSX1	12.29529	3.408379	0.27721	0.000458
GINS1	20.82079	5.772615	0.277252	1.95E-05
COBLL1	1.477633	0.409747	0.277299	0.011475
CNNM4	7.723683	2.141865	0.277311	0.005105
SETDB1	19.64233	5.447358	0.277328	1.37E-05
CEP44	3.610073	1.001341	0.277374	0.000895
PANK3	14.61177	4.053797	0.277434	0.010171
SNORC	0.66916	0.185857	0.277747	0.059661
TSC2	18.93865	5.262258	0.277858	0.01177
FAM234B	11.27735	3.135622	0.278046	0.00185
KMT2D	5.354123	1.488928	0.27809	0.017306
SEC14L5	0.062675	0.017434	0.278164	0.041549
SYTL4	3.245605	0.903018	0.278228	0.008223
PGAP1	3.351156	0.932565	0.278281	0.002577
PLEKHM3	1.77354	0.493665	0.27835	0.00087
LRP6	5.487441	1.528106	0.278473	0.003911
CEP57	18.3341	5.106221	0.27851	0.000558
GRAMD1C	3.011999	0.839378	0.278678	0.012082
HK3	0.016006	0.004461	0.278715	0.329009
TRABD	35.94154	10.02314	0.278873	0.000116
EIF4EBP1	416.9192	116.2864	0.278918	0.004659
TMEM52	1.28706	0.359178	0.279069	0.128668
KMT2C	5.817952	1.623778	0.279098	0.020715
DDX4	0.035711	0.009972	0.279238	0.287599
PASK	5.216131	1.45672	0.279272	0.000112
HECTD4	6.023854	1.682622	0.279327	0.010918
PABPN1	7.395985	2.067028	0.27948	0.00471
FHL3	11.28277	3.154734	0.279606	0.000804
NFXL1	6.908339	1.931682	0.279616	0.002327
LOC10192	0.01352	0.003782	0.279763	0.331292
VSIG4	0.025928	0.007254	0.279763	0.331292
EPRS	99.25489	27.77906	0.279876	0.003562
CCDC117	28.44317	7.9631	0.279965	0.000134
ZMYM1	7.278104	2.038271	0.280055	0.001489
SPOCK2	0.40229	0.1127	0.280147	0.006669
JMJD4	12.09241	3.387973	0.280174	0.027475
TPD52	16.84623	4.722335	0.28032	0.001025
TGDS	29.39936	8.242029	0.280347	0.007835
TMEM9	69.85308	19.5887	0.280427	7.29E-05
TRPA1	0.0503	0.014109	0.280502	0.176099
COA7	11.09995	3.11426	0.280565	0.011575
SCT	0.118729	0.033354	0.28092	0.27845
TMEM135	3.000271	0.843043	0.280989	0.005135
KANK1	2.945329	0.827916	0.281095	0.000228
ERN1	3.142638	0.883398	0.281101	0.004954
RAPGEF3	4.303951	1.210536	0.281261	0.150542
ANKS1A	3.756517	1.056579	0.281266	0.000875
TMEM170	7.578931	2.133395	0.28149	0.001068
CYP46A1	0.299424	0.084316	0.281594	0.036726
DXO	23.09575	6.506851	0.281734	0.000444
ATE1	7.296816	2.057777	0.28201	0.004505
VASH1	8.542143	2.410434	0.282181	0.002814

TFR2	0.0178	0.005023	0.28221	0.527683
CEACAM1	0.016203	0.004573	0.28221	0.527683
TRMT10A	3.80178	1.073217	0.282293	0.021982
ZNF169	1.576654	0.445115	0.282316	0.003295
OSGEP	24.05912	6.797936	0.282551	0.0091
USP37	5.386619	1.523807	0.282888	0.000406
CLK1	40.51803	11.46294	0.28291	0.011619
E4F1	12.91882	3.654897	0.282913	0.001089
POLDIP3	77.89832	22.04383	0.282982	0.000494
RPS3	1049.173	296.9529	0.283035	0.001028
AKAP5	0.654794	0.185397	0.283138	0.088226
GALR3	0.09804	0.027761	0.28316	0.322063
PTK6	0.022373	0.006335	0.28316	0.322063
RALGDS	15.17264	4.298151	0.283283	0.000962
RCCD1	16.20288	4.59586	0.283645	0.004046
LUC7L3	36.28934	10.30189	0.283882	0.007216
RBM46	0.01377	0.003914	0.284242	0.065848
ZSCAN4	0.017558	0.004991	0.284242	0.065848
IL9R	0.008832	0.002511	0.284242	0.065848
CTSZ	0.07743	0.022022	0.284407	0.43364
GLUL	31.70521	9.023447	0.284605	0.000367
CCDC173	2.104404	0.599159	0.284717	0.051882
ZNF670	2.088212	0.594735	0.284806	0.000125
TEKT2	0.396256	0.112876	0.284856	0.155471
MRPL48	55.0002	15.66819	0.284875	0.000339
TRAPPC6/	13.38856	3.814783	0.284929	0.011906
SLC17A9	0.043681	0.012446	0.284937	0.435042
IFITM2	22.12255	6.304813	0.284995	0.001088
AGER	2.485974	0.709024	0.28521	0.03262
DENND6A	11.99268	3.420713	0.285233	0.000466
ACSM1	0.010429	0.002976	0.285332	0.322711
FAM151A	0.027362	0.007807	0.285332	0.322711
PCSK2	0.022282	0.006358	0.285332	0.322711
GAN	1.330026	0.379717	0.285496	0.017283
BRINP2	0.047458	0.013552	0.285554	0.215343
RHOBTB3	20.69799	5.911367	0.285601	0.000426
ADAM33	0.021732	0.006209	0.285714	0.303877
GDPD3	4.094768	1.170332	0.285812	0.12525
KIF14	5.463692	1.56177	0.285845	0.000761
LAMA1	4.906059	1.40268	0.285908	0.003542
PRRC2A	88.02202	25.17229	0.285977	4.56E-05
RPS11	3402.822	973.1726	0.28599	0.000478
ISG20L2	27.81817	7.95763	0.286059	0.001473
MIEF1	2.978475	0.852062	0.286073	0.001961
CPB1	0.040031	0.011452	0.286082	0.530191
CLEC18A	0.010479	0.002998	0.286082	0.530191
CSNK2A3	10.15749	2.905897	0.286084	0.001107
RPS4X	5204.383	1489.623	0.286225	0.000288
THSD7A	0.029436	0.008425	0.286233	0.118834
ARG2	25.84625	7.398205	0.286239	0.001219
ZNF644	6.510808	1.864483	0.286367	0.002222
ZNF318	6.229737	1.784283	0.286414	0.001549
NUP43	27.0812	7.758732	0.286499	2.62E-05
OTUD1	10.8315	3.103219	0.2865	0.03833
PAN3	3.41442	0.978342	0.286532	0.0026
RPS6KA6	1.695963	0.486011	0.286569	0.001821
ATM	5.372686	1.539948	0.286625	0.002028
C19orf18	0.062973	0.018051	0.286639	0.335965

GFRA3	0.02984	0.008553	0.286639	0.335965
MUC7	0.020772	0.005954	0.286639	0.335965
LOC10013	0.121376	0.034822	0.286896	0.242617
PTPRS	16.18785	4.645139	0.286952	1.32E-05
C2orf68	9.009076	2.585657	0.287006	0.002908
CCNJ	4.369966	1.254595	0.287095	0.003136
ATP6V1B1	1.422833	0.408663	0.287218	0.040695
SLC22A15	1.236652	0.355211	0.287236	0.007098
ABHD18	2.547684	0.732596	0.287554	3.06E-05
NPIPA2	0.158372	0.045542	0.28756	0.401877
IDO1	0.029728	0.008553	0.287717	0.338277
FOXR2	0.020583	0.005922	0.287717	0.338278
P2RX1	0.028518	0.008206	0.287733	0.090617
SFTPD	0.040738	0.011722	0.287749	0.531274
CCDC85C	4.318486	1.243926	0.288047	0.011348
LVRN	0.161642	0.046562	0.288054	0.249894
GDF6	0.278611	0.080269	0.288103	0.126785
RAB40B	3.686237	1.062156	0.288141	0.010293
ZNF362	8.560942	2.467072	0.288178	0.005443
CRY1	10.93975	3.15787	0.28866	0.004703
WDR93	0.083154	0.024003	0.288661	0.133504
BBS7	13.02393	3.76061	0.288746	0.001843
PTPRT	0.00718	0.002074	0.288834	0.087791
TRPM5	0.012104	0.003497	0.288866	0.333817
NCR3	0.044019	0.012716	0.288866	0.333817
ESPL1	12.19239	3.52387	0.289022	0.001057
YBX3	212.7876	61.64469	0.289701	2.89E-05
MPDZ	5.284437	1.531296	0.289775	0.001558
COG2	19.72193	5.716499	0.289855	0.001725
CACNA1E	0.012813	0.003715	0.289968	0.33362
VIT	0.035729	0.010361	0.289986	0.268079
AHSG	0.03231	0.00937	0.289992	0.332176
CCR6	0.030041	0.008712	0.289992	0.332176
ZBED4	8.097796	2.348466	0.290013	0.002271
GP6	0.04952	0.014363	0.29005	0.239967
TEX22	0.495742	0.143846	0.290162	0.101745
CWF19L2	8.475233	2.460876	0.290361	0.00236
IQCH	2.060776	0.598397	0.290374	0.054583
SERPINB9	0.012589	0.003658	0.290573	0.339458
RNF214	13.27195	3.856568	0.29058	0.0066
GLIPR1L2	0.112613	0.032739	0.290719	0.068363
MRPS17	49.77892	14.48365	0.29096	0.017639
ASCL5	0.161522	0.047004	0.291004	0.218719
PEX11G	1.500204	0.436654	0.291063	0.086696
CXorf40B	11.86101	3.452764	0.291102	0.003632
NECAB1	0.010732	0.003124	0.291126	0.533472
CYP17A1	0.029499	0.008588	0.291126	0.533472
ADGRD2	0.015138	0.004407	0.291126	0.533472
LOC10272	0.005615	0.001635	0.291126	0.533472
FAM135B	0.005476	0.001594	0.291126	0.533472
CCER2	0.285678	0.083183	0.291177	0.200829
LPAR5	0.057738	0.016812	0.291183	0.046493
GRIA2	0.019324	0.005627	0.29121	0.329282
RSAD2	0.014801	0.00431	0.29121	0.329282
PNP	59.02922	17.19348	0.291271	0.000485
CCNT1	12.91249	3.762672	0.291398	8.30E-05
WDR26	43.95143	12.81368	0.291542	0.00095
KLC4	10.4026	3.033292	0.29159	0.008738

GTF2H2	7.233573	2.109293	0.291598	0.002875
USP30	7.199816	2.09952	0.291607	0.000253
CD22	1.643489	0.479525	0.291773	0.129508
PLCE1	1.509425	0.440556	0.29187	0.001725
LOC10537	0.102004	0.029797	0.292119	0.149763
CPN1	0.032176	0.009406	0.292323	0.073064
IFT74	5.828712	1.704161	0.292374	0.022182
GPSM1	10.11513	2.958169	0.29245	4.32E-05
GAB1	0.900343	0.26335	0.292499	0.003929
RSF1	6.861974	2.00732	0.292528	0.001341
GGCT	88.06867	25.76353	0.292539	0.002479
ZNF234	1.420543	0.415637	0.29259	0.00023
DESI2	26.88554	7.866481	0.292592	2.89E-06
HTT	12.4836	3.654488	0.292743	0.002815
PPP1R27	0.143099	0.04191	0.292871	0.289624
KCNMB4	1.624301	0.475769	0.292907	0.014064
BAMBI	12.99386	3.806458	0.292943	0.005704
UBE2K	17.58256	5.151016	0.292962	0.000233
CEP97	5.455448	1.598438	0.292998	0.002643
SIK2	5.146749	1.509789	0.293348	0.009972
PPFIA3	5.811526	1.705005	0.293383	0.000837
LRMP	0.036584	0.010735	0.293444	0.330028
PHF7	3.170277	0.930588	0.293535	0.052413
EIF2D	26.6667	7.828258	0.293559	2.21E-05
DTX1	0.182513	0.053588	0.29361	0.058251
GTF2H4	23.38989	6.86947	0.293694	0.001184
FLYWCH1	11.10079	3.260408	0.29371	0.001482
DAB2IP	3.963828	1.164348	0.293743	0.001158
CPO	0.033218	0.009758	0.293746	0.101088
ZNF519	0.958446	0.281562	0.29377	0.000136
INPP4A	2.980943	0.875926	0.293842	7.15E-05
MINOS1-I	0.153162	0.045044	0.294096	0.370407
ZNF69	0.053011	0.015591	0.2941	0.155805
SMIM13	9.003461	2.650507	0.294388	0.000194
CFAP126	0.215924	0.06357	0.29441	0.162303
GPRC5A	9.51065	2.802035	0.294621	0.07609
GABARAP	54.42167	16.03872	0.294712	0.010113
LYST	3.055159	0.900572	0.294771	0.018321
ZNF75D	2.266814	0.668229	0.294788	0.023302
VARS2	15.19259	4.478779	0.2948	0.000142
PRUNE1	14.19909	4.188049	0.294952	5.89E-05
BNC1	0.201086	0.059329	0.295045	0.048856
BICRA	2.801863	0.826714	0.295059	2.74E-05
LOC10798	0.17292	0.051038	0.295153	0.032434
NISCH	19.53845	5.768492	0.295238	0.00445
ELF2	10.3579	3.058772	0.295308	0.000656
GTF2I	10.02931	2.965644	0.295698	0.008495
GABRG2	0.055539	0.016427	0.295769	0.162545
RCOR3	5.140307	1.520427	0.295785	0.001254
DYNC2LI1	17.88517	5.290285	0.295792	0.000558
DLG3	5.654727	1.672669	0.2958	0.017239
LOC10537	0.036961	0.010938	0.295929	0.536608
MPST	47.64941	14.10111	0.295935	0.001431
AURKC	1.834416	0.543068	0.296044	0.013452
CCDC146	1.149878	0.340675	0.29627	0.049844
CTRB1	0.064572	0.019135	0.296334	0.076813
GPR143	0.030021	0.008896	0.296334	0.076813
EHHADH	1.984289	0.588022	0.296339	0.047094

MSH2	14.1906	4.207144	0.296474	0.00229
TUBE1	27.79709	8.242374	0.296519	0.001246
RBM26	21.49141	6.373894	0.296579	4.52E-05
HDAC11	3.221444	0.955686	0.296664	0.015333
LOC73018	8.074949	2.39563	0.296674	0.010672
KCNF1	0.120592	0.035778	0.296682	0.479393
NOCT	5.501056	1.632204	0.296707	0.002339
SESN1	8.22941	2.442721	0.296828	0.000525
B3GALNT2	8.336559	2.474812	0.296862	0.000108
POMT1	11.78774	3.499577	0.296883	0.001161
RAB6C	0.270491	0.080363	0.297102	0.031657
MORN4	17.76234	5.278144	0.297154	2.72E-06
TCEANC	1.41999	0.421963	0.297159	0.015808
C20orf197	0.00351	0.001044	0.29747	0.333705
LOC10798	0.012737	0.003789	0.29747	0.333705
ABHD10	40.25259	11.97473	0.29749	0.000436
LMNTD2	1.509283	0.4507	0.298618	2.77E-05
INSR	5.398014	1.611961	0.298621	0.000392
CHRDL1	2.002947	0.598451	0.298785	0.022462
TRMT1L	7.825793	2.338813	0.29886	0.022377
FAM86B2	0.534868	0.159858	0.298874	0.143674
NELFA	18.10175	5.412523	0.299005	0.000429
TRMT11	26.56272	7.943641	0.299052	1.44E-05
SLC22A5	6.365045	1.903776	0.299099	0.001069
PUS10	1.581789	0.473295	0.299215	0.000511
PLIN1	0.526669	0.157589	0.299218	0.032904
RBBP4	75.03393	22.45579	0.299275	1.24E-05
PRDM9	0.014921	0.004467	0.299403	0.538884
DDX25	0.023168	0.006937	0.299403	0.538884
CYTH1	9.540592	2.857971	0.299559	0.005056
TLE3	14.38916	4.310828	0.299589	1.93E-05
FIGN	0.927788	0.278059	0.299701	0.000475
MTERF2	2.012586	0.603493	0.299859	0.003438
LGI1	0.703608	0.211031	0.299927	0.000327
LUZP2	0.005905	0.001771	0.29999	0.53927
MYORG	2.101601	0.63056	0.300038	0.004798
GTF3C1	29.44554	8.836577	0.300099	0.000716
MACROD1	12.51862	3.758454	0.300229	0.004608
CCNG1	151.6018	45.54137	0.300401	0.007265
CAPN14	0.127312	0.03827	0.300599	0.069128
ATXN1	3.249976	0.978296	0.301016	0.012006
HSF2	34.65722	10.4353	0.3011	9.25E-06
MRPS9	60.37479	18.18018	0.301122	0.013243
POLQ	8.15558	2.455995	0.301143	0.002098
NWD1	0.006226	0.001875	0.301155	0.344921
C1orf131	21.99051	6.626024	0.301313	0.014931
HAUS5	17.78685	5.362489	0.301486	0.001056
SAV1	9.049917	2.728645	0.301511	0.002402
GALNT16	6.987762	2.106893	0.301512	0.016547
IQSEC1	6.321408	1.906418	0.301581	0.001135
ZHX2	1.978396	0.596684	0.3016	0.001375
SPICE1	11.0174	3.323676	0.301675	0.000516
PPM1D	6.67419	2.015401	0.301969	0.000246
GDPD5	2.402036	0.725449	0.302014	0.031944
KATNAL2	0.46238	0.13971	0.302155	0.004784
MED13L	8.77281	2.650849	0.302166	0.007754
SCN1A	0.006295	0.001903	0.302329	0.343383
CA5B	2.820202	0.852672	0.302344	0.000576

UHRF2	19.49274	5.899208	0.302636	0.001042
TTLL7	2.624567	0.794977	0.302898	0.001862
BAZ1A	11.37448	3.449099	0.303231	0.002287
ZNF852	2.385624	0.723819	0.303409	0.001969
CST1	0.072584	0.02203	0.303512	0.541585
TTR	0.05938	0.018023	0.303512	0.541585
FAM199X	19.72168	5.986856	0.303567	0.003052
ZNF821	4.125834	1.25258	0.303595	0.002321
VCPKMT	1.522708	0.462337	0.303628	0.008785
ZCCHC8	11.27415	3.425554	0.303841	0.007668
RAB3IP	4.115454	1.250609	0.303881	0.000396
TRIM69	0.140213	0.042616	0.303935	0.236612
PRSS53	4.884094	1.484809	0.304009	0.102088
CDKAL1	4.701953	1.42981	0.304089	0.000403
CNTRL	4.801449	1.461592	0.304407	0.014744
MAST1	5.813585	1.771628	0.304739	0.002128
ZHX1-C8c	0.526613	0.160595	0.304958	0.166065
ZNF839	4.491567	1.369953	0.305006	0.020443
TRUB1	10.27026	3.136205	0.305368	0.017476
TTK	31.8132	9.71641	0.305421	0.00032
KCTD3	22.5364	6.883411	0.305435	0.025673
ZNF621	7.611128	2.325857	0.305586	0.005423
GLMN	7.617446	2.329389	0.305797	0.009569
RPL18	1500.505	458.8804	0.305817	0.001928
CHML	12.52955	3.832485	0.305876	0.006509
PTK7	35.27473	10.79045	0.305898	0.000525
REPS1	24.25482	7.422449	0.306019	0.020466
ZNF133	2.705293	0.828208	0.306144	0.000412
KLHL8	6.823733	2.090809	0.306403	0.000265
SCAI	2.378594	0.728858	0.306424	0.004851
ADARB1	2.115583	0.64871	0.306634	0.003606
HAUS3	19.31818	5.924122	0.30666	0.001427
GNB3	2.710978	0.831632	0.306765	0.045773
PPA1	231.2102	70.93317	0.306791	0.000463
DGKD	6.042167	1.854518	0.306929	0.000805
GPA33	0.0737	0.022629	0.307038	0.342262
KLHL13	2.662421	0.818488	0.307423	0.008788
MBOAT1	1.387475	0.426571	0.307444	0.002989
MST1L	0.193503	0.059513	0.307556	0.054388
TIAF1	0.09992	0.030751	0.307756	0.158051
BNIPL	0.349701	0.107664	0.307876	0.079206
ZNF84	8.5269	2.625308	0.307885	0.007528
MCM8	10.03228	3.089276	0.307934	2.24E-05
ZNF782	0.645652	0.198879	0.308028	0.003274
EFNB1	7.388237	2.275793	0.308029	0.001223
CHRM4	0.206552	0.063625	0.308036	0.029663
CORT	0.216547	0.066714	0.308081	0.208986
AKAP8L	55.52509	17.10731	0.308101	0.001842
SHMT2	142.2702	43.84358	0.308171	2.34E-05
TVP23C	0.88096	0.271511	0.308199	0.006878
HPD	0.218799	0.067448	0.308264	0.369745
ELP2	28.59648	8.81976	0.308421	0.000236
FAM118A	8.176417	2.521942	0.308441	0.003032
TCOF1	68.09707	21.01623	0.308622	0.002003
TRIM48	0.261782	0.080794	0.308631	0.161015
MASTL	20.70069	6.389462	0.308659	0.000275
RIF1	8.897228	2.746864	0.308733	0.000357
AARS	259.9662	80.32203	0.308971	0.000247

ZSCAN9	7.206245	2.226609	0.308983	0.002955
KAT6A	6.251536	1.932112	0.309062	0.004512
PPP2R3B	3.134248	0.969422	0.3093	0.188974
ZNF337	5.079929	1.571617	0.309378	0.003945
C3orf52	8.946016	2.768618	0.309481	0.0016
CMTR1	28.24237	8.745945	0.309675	0.003237
MINDY3	9.147593	2.833082	0.309708	1.81E-05
ZRANB2	101.9458	31.58265	0.309798	0.001184
MAML1	15.39342	4.769229	0.309823	0.006471
ITGB4	0.278313	0.086254	0.309919	0.029297
DCDC2	0.265487	0.082329	0.310107	0.075381
MRPL55	51.03803	15.83493	0.310257	3.16E-05
RBM25	52.70049	16.36911	0.310606	0.000808
CCDC150	3.072608	0.955033	0.310822	0.001187
LMTK3	1.423672	0.442581	0.310873	0.008952
SP1	27.46836	8.541618	0.310962	0.004335
PRR22	0.569083	0.176986	0.311002	0.023425
RFC5	38.60074	12.00541	0.311015	7.60E-06
THOC3	38.72018	12.04326	0.311033	0.011343
BRWD1	3.543758	1.102328	0.311062	0.003513
EED	14.19928	4.418282	0.311162	1.03E-05
TAS2R20	1.104601	0.343743	0.311192	0.087872
NBL1	0.063343	0.019718	0.311292	0.095612
FAM221A	1.862376	0.579909	0.311381	0.08084
MED28	27.34924	8.5198	0.311519	0.004917
MOCS1	4.861322	1.514544	0.31155	0.010473
FCHSD2	6.266765	1.952539	0.311571	0.000186
MIF4GD	10.1591	3.16548	0.311591	0.006181
ARIH1	36.3546	11.33426	0.31177	0.00032
CDC14C	0.14394	0.044946	0.312257	0.065285
CBY1	70.7716	22.10749	0.312378	0.017471
B3GNT4	0.613663	0.191826	0.312592	0.12598
MLST8	20.66699	6.46157	0.312652	0.001966
GPR155	2.428613	0.760386	0.313095	0.001446
GATAD1	15.55183	4.870978	0.313209	8.43E-05
SRGAP3	0.79134	0.24786	0.313216	0.011933
ZSCAN31	0.230901	0.072341	0.3133	0.022668
ATP9A	6.1508	1.928437	0.313526	0.016795
CASC1	0.428924	0.134517	0.313616	0.253835
LRRC24	0.981213	0.307828	0.313722	0.027178
C19orf81	7.403716	2.323265	0.313797	0.000195
ZNRD1	38.23646	11.99968	0.313828	0.004677
MRPS26	76.51464	24.01562	0.31387	0.000889
SNRPB	445.0383	139.7241	0.31396	2.99E-05
EEPD1	0.683348	0.21461	0.314057	0.000714
CKAP2	39.93178	12.54276	0.314105	0.000457
MRPL2	45.41771	14.26722	0.314133	7.77E-05
POLR2J3	1.433862	0.450654	0.314294	0.006747
ACACA	9.628864	3.026826	0.314349	0.003535
TAF8	6.500861	2.043626	0.314362	3.62E-05
SLC4A3	1.870254	0.588025	0.314409	0.000943
FGFBP3	1.306331	0.410739	0.314422	0.056088
GFRA2	0.039791	0.012517	0.31458	0.054134
FAM133B	5.490168	1.727528	0.314658	0.00102
COL27A1	3.257584	1.025053	0.314667	0.001091
SPAG9	29.1815	9.183417	0.3147	0.008151
U2SURP	34.56091	10.88607	0.314982	0.001244
NPIPB15	1.492685	0.470203	0.315005	0.044934

DIP2A	5.683059	1.791171	0.315177	0.004761
RHOBTB1	1.973929	0.62215	0.315184	0.000621
ACTL6B	0.100811	0.031791	0.315351	0.464544
SF3B6	511.0852	161.2045	0.315416	7.56E-05
SEMA4D	1.89559	0.598764	0.315872	0.001134
BAK1	17.6807	5.587441	0.316019	0.014404
CYB5RL	4.046969	1.279391	0.316136	0.019917
ARHGEF9	2.90946	0.919807	0.316143	0.005991
NPAT	3.867384	1.223726	0.316422	0.001195
NFKBIL1	30.53504	9.66412	0.316493	0.001797
ABCC5	4.276316	1.354235	0.316683	0.000317
RPS29	59.78325	18.93437	0.316717	0.00147
CEP95	8.329996	2.638262	0.316718	0.002452
C12orf73	8.540608	2.706616	0.316911	1.98E-05
CUTC	40.78125	12.92423	0.316916	0.003206
ZBED3	1.368747	0.433809	0.316939	0.025852
MCM6	74.88824	23.74161	0.317027	0.000366
BDP1	4.967991	1.575365	0.317103	0.003654
CCDC74B	6.6606	2.112959	0.317233	0.001022
ZNF891	1.673499	0.530896	0.317237	0.005709
ALG10	2.993235	0.949595	0.317247	0.000442
PRKDC	57.90834	18.37739	0.317353	0.003494
ID2	80.46024	25.55636	0.317627	0.193947
VHL	46.03075	14.62095	0.317634	1.97E-06
HAPLN1	0.120986	0.038436	0.31769	0.057795
ACTR3B	4.123779	1.310163	0.317709	0.000343
DDHD1	2.603982	0.82731	0.31771	0.011952
MON2	5.56917	1.769388	0.317711	0.006119
IARS2	70.69594	22.46247	0.317734	0.007756
CAGE1	0.044258	0.014067	0.317835	0.314104
PLXDC1	1.018031	0.323574	0.317843	0.024247
PRR14L	5.760309	1.831359	0.317927	0.000312
GON4L	9.532315	3.030628	0.317932	0.000727
YPEL3	33.25474	10.57975	0.318143	0.04746
PCNA	323.7306	103.147	0.31862	0.000401
C2CD2L	5.002102	1.595095	0.318885	0.006707
NOA1	25.05507	7.99656	0.319159	0.025911
LOC10272	0.53368	0.170343	0.319186	0.009593
RNPC3	11.40431	3.640636	0.319233	0.000138
GSTM5	0.308121	0.098397	0.319345	0.288444
FCHO1	4.060004	1.296874	0.319427	0.016164
SLC43A2	1.122091	0.358489	0.319483	0.008779
ZNF518B	1.973494	0.630647	0.319558	0.048479
PRKAR2B	5.76899	1.843841	0.319612	0.006165
TIA1	8.256804	2.639648	0.319694	0.002887
RPS6KC1	4.959895	1.58605	0.319775	0.000448
ZNF624	1.224881	0.391746	0.319824	1.39E-05
DHX57	15.93964	5.098445	0.31986	0.001124
SLC24A1	1.553484	0.497069	0.319971	0.017108
PHIP	5.662106	1.812476	0.320106	0.000463
MRPL42	30.5413	9.776981	0.320123	0.010274
MCF2L2	0.290391	0.092974	0.320168	0.028913
TBC1D4	9.546171	3.057408	0.320276	0.004958
PFKFB2	1.484523	0.475774	0.32049	0.001019
PMS1	9.644331	3.090987	0.320498	0.002621
CREBBP	5.260549	1.686165	0.32053	0.044498
SDHAF3	15.21829	4.88006	0.320671	0.023913
RFESD	2.66629	0.855128	0.320718	0.008646

ZNF10	2.137216	0.68573	0.320852	0.007331
FOXK1	10.47431	3.361961	0.320972	0.001144
RNF144B	1.058944	0.340213	0.321276	0.015343
LPGAT1	7.323655	2.353799	0.321397	0.001653
BPTF	8.347808	2.683201	0.321426	0.001945
ZNF610	0.087448	0.028116	0.321514	0.172367
NCBP3	9.06766	2.916844	0.321676	0.002625
ADCY7	2.556773	0.822463	0.32168	0.000432
IREB2	14.97492	4.817833	0.321727	0.002126
ZNF34	2.661729	0.856962	0.321957	4.05E-05
ARHGAP2	0.91491	0.29491	0.322338	0.000721
ZNF32	11.49644	3.706539	0.322407	0.057498
DFFA	26.79251	8.640005	0.322478	0.008449
ITPRIPL1	2.823905	0.911047	0.32262	0.012706
ZNF76	11.61276	3.74862	0.322802	0.00084
ACVR1B	7.400686	2.389234	0.32284	4.61E-05
MOAP1	22.32543	7.20756	0.322841	0.000715
KALRN	0.272612	0.088062	0.323032	0.000923
EIF3E	385.0715	124.4564	0.323203	0.001989
BRD1	4.588918	1.484347	0.323463	0.000233
TRIML2	0.589405	0.190676	0.323505	0.176713
DLAT	13.85039	4.482634	0.323647	0.01993
DTNBP1	8.027026	2.598979	0.323779	0.006256
WDR48	17.99856	5.838289	0.324375	1.64E-05
H2AFJ	4.475019	1.451909	0.324448	0.000187
KHDRBS3	7.056632	2.289829	0.324493	0.001479
LTC4S	0.451431	0.146519	0.324565	0.01731
SMARCD1	50.52535	16.39896	0.324569	0.002932
TMEM229	0.881502	0.286141	0.324606	1.73E-05
FZD4	2.525479	0.819854	0.324633	0.005241
PDE1C	0.18671	0.060617	0.324659	0.134662
CSNK2A1	45.69077	14.83634	0.324712	0.000142
GLA	134.6617	43.73869	0.324804	0.112457
ZNF514	6.540408	2.12532	0.324952	0.004999
CIDEC	0.105049	0.034145	0.325042	0.482758
LCP1	14.201	4.618212	0.325203	0.002848
PCLO	0.698129	0.227163	0.325388	0.052415
IRAK1BP1	1.989139	0.64732	0.325427	0.087616
RHOT1	11.11426	3.61765	0.325496	2.47E-05
SPEN	11.09331	3.614078	0.325789	0.000436
TCERG1	34.02617	11.08624	0.325815	0.000353
ORC1	22.52896	7.340889	0.325842	0.001525
NBEAL2	3.121365	1.017312	0.325919	0.001358
PNPLA7	0.661159	0.215509	0.325956	0.002894
ZNF587	4.561718	1.487437	0.326069	0.003906
MGRN1	3.550082	1.158414	0.326306	0.000132
CAMK1G	0.114191	0.037263	0.326322	0.050721
USP9X	32.43833	10.58701	0.326373	0.000166
TET2	1.234587	0.403005	0.326429	0.002376
RPL10A	1508.98	492.7799	0.326565	7.96E-05
OXA1L	105.5959	34.52523	0.326956	0.000401
PCDHGB4	0.033145	0.010837	0.32697	0.160367
OLFM1	0.178713	0.058442	0.327014	0.214695
RPUSD3	54.2862	17.76405	0.32723	0.011271
PLCL2	1.399073	0.457871	0.327267	0.006889
EIF1	1368.464	447.9626	0.327347	0.006048
SLC25A36	34.48016	11.28877	0.327399	0.007743
FSD1	25.1137	8.224265	0.327481	0.002087

FBXO5	41.60586	13.62909	0.327576	0.010004
GLI1	2.351556	0.770484	0.327649	0.001699
LIG1	29.55709	9.684475	0.327653	9.91E-07
C3orf33	2.941474	0.964313	0.327833	0.001961
NCKAP5	0.10387	0.034055	0.327859	0.02673
USP42	4.824013	1.58222	0.327988	0.003428
ADAMTS3	0.904801	0.29687	0.328105	0.039454
TOP1MT	21.93923	7.199453	0.328154	0.001856
DNAJA3	44.93454	14.75708	0.328413	8.00E-05
NAF1	10.09621	3.318984	0.328736	0.003861
E2F8	5.236403	1.721405	0.328738	0.000205
GADD45A	152.8027	50.24279	0.328808	0.03174
CUL5	7.99101	2.627675	0.328829	0.000828
TMEM38A	5.357429	1.762688	0.329017	0.006811
TRIM74	0.977392	0.321676	0.329117	0.001915
NT5DC4	0.01526	0.005025	0.32929	0.558721
CST7	0.104883	0.034537	0.32929	0.558721
SLF1	3.007611	0.990437	0.32931	0.002908
PUM1	27.12067	8.93212	0.329347	0.00487
LRRC39	1.539462	0.507106	0.329405	0.028801
POLN	0.779666	0.256869	0.329461	0.043962
TBC1D3L	1.836361	0.605038	0.329477	0.064428
RPL13A	3267.974	1077.255	0.32964	0.001237
FLI1	0.146286	0.048225	0.329665	0.107102
POLD1	43.43845	14.32031	0.329669	0.000195
ZNF439	0.330643	0.109012	0.329696	0.06398
JCAD	1.546422	0.509951	0.329762	0.000106
SECISBP2	23.42279	7.725724	0.329838	0.000172
CDYL	12.78199	4.216141	0.32985	8.84E-05
HEATR1	21.7314	7.168263	0.329857	0.00416
CNTLN	2.347795	0.774516	0.329891	0.008025
CDK2	81.20298	26.80296	0.330074	5.80E-05
TMCO6	4.757424	1.570839	0.330187	0.038526
TIMELESS	29.68289	9.803685	0.330281	0.005177
PGBD1	5.974684	1.973907	0.330378	0.012193
MYBL2	114.9764	38.00621	0.330557	0.002081
HOPX	0.568896	0.18807	0.330589	0.061014
PKD1L1	1.496975	0.494903	0.330602	0.015069
CHD8	15.41988	5.099762	0.330726	0.000292
ZNF641	1.460492	0.483059	0.330751	0.007952
AKR7A3	0.636414	0.21084	0.331294	0.044259
PPOX	6.971301	2.309582	0.331299	0.000105
MCM4	98.51362	32.64118	0.331337	0.000233
ATXN2L	71.54125	23.72537	0.331632	0.006096
METTL3	43.98475	14.60224	0.331984	3.94E-05
TTC39B	1.66246	0.552057	0.332072	0.000271
TTBK2	4.198066	1.395975	0.332528	0.016115
STARD9	1.297643	0.431631	0.332627	0.018839
EIF4EBP2	18.29443	6.085779	0.332658	0.008262
EPHA4	3.218475	1.070701	0.332673	0.005949
GPR180	11.48914	3.824445	0.332875	0.000104
RINL	0.404121	0.13459	0.333044	0.026707
POM121	7.090613	2.361962	0.333111	0.001935
TEX12	0.084284	0.028076	0.333116	0.453974
PDCD11	20.16361	6.722274	0.333386	0.00433
ABRACL	22.79827	7.603409	0.333508	0.000956
GMCL1	10.95554	3.654778	0.333601	8.82E-05
LMTK2	5.146412	1.717474	0.333723	0.002725

POGZ	6.901345	2.303541	0.333781	0.000837
GZMM	0.259941	0.086785	0.333865	0.024419
DIS3	32.61205	10.902	0.334294	0.00051
PODXL	13.14768	4.395733	0.334335	0.002063
SERBP1	215.5797	72.09565	0.334427	0.003572
TFDP2	12.51372	4.18615	0.334525	0.003313
ASMTL	3.307341	1.106484	0.334554	0.116053
ANGEL1	12.63815	4.229648	0.334673	0.001686
PPT2	16.29326	5.453733	0.334723	0.004499
TMEM184	0.050153	0.01679	0.33477	0.198675
OTUB2	2.861813	0.958313	0.334862	0.002712
KDM6A	7.739352	2.592259	0.334945	0.00066
TNRC6B	3.219786	1.078508	0.334962	0.002563
NIPSNAP2	50.71182	16.99751	0.335179	0.00213
RAD54L2	5.750448	1.928572	0.335378	0.002576
ZNF783	5.557931	1.865353	0.33562	0.05364
MAP3K9	2.055657	0.689992	0.335655	0.009834
ALG6	12.68255	4.258684	0.335791	0.013707
AXIN2	2.003849	0.673165	0.335936	0.000181
KDM5B	12.91772	4.339562	0.335939	0.001655
ATAD1	12.86591	4.32328	0.336026	0.000952
STK19	11.85888	3.985269	0.336058	0.000335
FIBIN	1.041462	0.349997	0.336063	0.324876
CDS2	10.44591	3.514224	0.336421	0.000454
MICU3	0.691795	0.232926	0.336698	0.002432
NPIPB11	0.399458	0.134524	0.336767	0.002653
STAB1	0.046096	0.01553	0.336905	0.261396
KDM3B	13.56787	4.571554	0.33694	0.004099
RFC1	37.06096	12.48762	0.336948	0.001168
LRRN1	0.015268	0.005146	0.337032	0.3551
RAD9A	13.37347	4.508209	0.337101	0.003047
ZFHX4	1.116093	0.376493	0.337331	0.008577
ETV3	7.942987	2.679911	0.337393	0.007122
SDCBP2	1.395105	0.471332	0.337847	0.047768
CEP83	1.4989	0.5064	0.337848	0.033213
DRICH1	0.15226	0.051448	0.337895	0.051118
HOXB4	7.258769	2.452923	0.337926	0.014313
DR1	25.7505	8.706566	0.338113	0.001418
GLCCI1	2.987885	1.01035	0.338149	0.007377
BRCA1	17.07314	5.776107	0.338315	0.004935
MRS2	11.19635	3.787917	0.338317	0.000745
GART	60.38689	20.43046	0.338326	0.004915
AKAP9	4.607707	1.559101	0.338368	0.011965
FNIP2	1.724678	0.583685	0.338431	0.000667
GTF2IRD1	9.921198	3.358134	0.338481	7.51E-06
ABCB7	25.57697	8.658932	0.338544	0.004104
SMIM11B	0.023814	0.008065	0.338652	0.565022
SLC25A41	0.054625	0.018502	0.338712	0.348387
DCAF8	37.4482	12.68628	0.338769	0.001764
AEBP2	5.160782	1.748918	0.338886	0.000582
STRN3	8.969623	3.040783	0.339009	0.003081
CDK16	69.47105	23.55365	0.339043	7.97E-07
TCF7L2	3.305776	1.120828	0.339052	0.00304
UBXN7	11.1472	3.780502	0.339144	0.009395
ANK2	1.782338	0.604626	0.339232	0.019675
BUB1B-PA	0.024391	0.00828	0.339485	0.172951
NLRC3	0.025875	0.008784	0.339485	0.172951
PPP1R26	6.161847	2.092322	0.339561	0.005222

ACKR2	0.178628	0.060682	0.339713	0.183053
POLR3B	3.76025	1.278623	0.340037	0.077556
CCDC18	3.04122	1.034263	0.340082	0.000181
AGO4	4.018196	1.366833	0.340161	0.005543
TMEM229	0.03696	0.012576	0.340261	0.17227
TAS1R3	0.026869	0.009143	0.340281	0.305771
HSD17B11	25.50295	8.680572	0.340375	0.014953
FUBP1	26.86266	9.145572	0.340457	0.00051
GAD1	4.262947	1.451432	0.340476	0.001756
DYNC1I1	0.604052	0.2057	0.340534	0.040838
ARID2	4.118881	1.402918	0.340607	0.005734
DFFB	2.854517	0.972432	0.340664	0.000181
ZNF333	1.361501	0.464128	0.340894	0.05803
ACADM	26.61856	9.075413	0.340943	0.010605
CEP350	6.269177	2.138065	0.341044	0.003003
DDX50	20.32982	6.9336	0.341056	0.00559
ARRB2	48.8134	16.65108	0.341117	0.000585
RPL3	3624.962	1236.852	0.341204	0.000413
AMOTL1	9.017373	3.08164	0.341745	0.000129
NPM3	76.26268	26.06725	0.341809	0.003496
FAM219B	23.66854	8.094853	0.342009	0.012662
FBXO10	3.30526	1.131027	0.34219	0.021363
COL21A1	0.160954	0.05509	0.342271	0.070336
COMTD1	11.50439	3.939408	0.342427	0.002291
PTGR2	14.46897	4.954618	0.342431	0.02608
PROM1	0.08737	0.029943	0.34272	0.253701
KCNIP2	0.750766	0.257377	0.34282	0.177395
PCNX2	4.452488	1.526942	0.342941	0.000664
MACC1	0.031112	0.01067	0.342957	0.141395
KIAA1755	0.535882	0.183785	0.342958	0.022814
SYNDIG1L	0.185463	0.063614	0.342999	0.118853
CCDC93	8.088849	2.776259	0.343221	0.007812
DNAH3	0.019043	0.006536	0.343238	0.283331
RASGRF2	0.611382	0.20987	0.343271	0.000199
ZXDC	7.78351	2.67194	0.343282	0.011526
ZNF227	7.061793	2.424308	0.343299	0.00028
HELZ	4.275745	1.467993	0.34333	0.005557
TANC1	5.417281	1.860651	0.343466	0.004468
MTBP	5.433236	1.866585	0.343549	0.005202
GABPA	8.219927	2.824795	0.343652	2.21E-05
MIA2	3.33092	1.14511	0.343782	0.007645
CYP2D6	0.11528	0.039684	0.344238	0.226629
PLCB1	2.43854	0.839613	0.34431	0.037019
CPSF1	30.68016	10.56437	0.344339	0.000197
STAT5B	11.55823	3.982336	0.344545	0.008333
TTC14	9.676381	3.334254	0.344577	0.025592
EXOSC1	22.73127	7.835799	0.344715	0.004805
ENO3	3.244591	1.118698	0.344789	0.039175
PRTFDC1	10.9057	3.760268	0.344798	0.030029
CEP68	3.594185	1.239356	0.344823	0.000142
OSBPL1A	10.3044	3.555692	0.345065	0.009453
INPP1	6.477403	2.23514	0.345067	0.000536
ADNP2	10.22307	3.530187	0.345316	0.000214
SNRNP200	68.08866	23.51449	0.345351	0.006531
GSTM2	20.65015	7.135265	0.345531	0.002117
ZNF573	0.628325	0.217108	0.345535	0.055346
TCTE1	0.388935	0.134469	0.345737	0.070504
MPP3	3.214892	1.111595	0.345764	2.89E-06

TRAPPC6E	12.8192	4.433072	0.345815	0.000109
CACNB3	5.440302	1.881482	0.345841	0.004793
CTDSPL2	10.97029	3.796271	0.34605	0.001596
MTMR3	8.313854	2.878763	0.346261	0.006676
POC1B	10.09943	3.497114	0.346269	4.64E-06
HESX1	0.734938	0.254675	0.346526	0.137367
LOC10099	0.027467	0.009519	0.346562	0.336439
PAX3	3.813164	1.321692	0.346613	0.000837
WDR90	4.643826	1.610351	0.346772	0.002154
PSIP1	54.65763	18.9541	0.346779	6.96E-05
KIAA1328	1.279228	0.44375	0.346888	0.001162
HPX	0.375588	0.13032	0.346976	0.262584
C4orf33	8.900848	3.088677	0.347009	0.003074
CLASP2	14.53132	5.042767	0.347027	0.012801
EIF4B	236.1047	81.95328	0.347106	0.003615
ZNF346	4.928791	1.711707	0.347287	0.011126
GTPBP3	24.43087	8.484705	0.347294	0.000679
LRIG2	4.217265	1.465106	0.347407	0.000236
CAAP1	7.808986	2.713359	0.347466	0.002445
SCNN1B	0.033277	0.011568	0.347633	0.204021
NCAM1	7.810738	2.716878	0.347839	0.000107
ANKRD49	7.228021	2.514301	0.347855	0.003343
PELI2	1.197636	0.416686	0.347923	0.000581
C7orf57	0.264781	0.092124	0.347925	0.069975
SMC5	11.5723	4.026365	0.347931	0.008534
C5orf22	11.9376	4.154104	0.347985	7.33E-05
CAD	20.04207	6.974862	0.348011	0.00152
SUDS3	19.9722	6.957213	0.348345	0.002184
NEK9	27.21265	9.481879	0.348436	0.01104
FAT2	0.170428	0.059392	0.348486	0.041933
LRPPRC	41.23132	14.37509	0.348645	0.001968
CABP7	0.157974	0.055078	0.348654	0.015551
RHBDD3	12.19428	4.253335	0.348798	0.002394
TTLL9	0.053514	0.018676	0.348995	0.212345
ATP6V0A4	0.070754	0.024707	0.349197	0.067105
NOTCH3	13.52257	4.722142	0.349204	0.009124
RBMXL1	5.552105	1.939353	0.349301	0.000288
EZH2	24.72783	8.638284	0.349334	1.51E-06
KCNIP1	0.019909	0.006955	0.349352	0.572271
ACTR6	21.82483	7.634243	0.349796	0.023675
DNASE1L2	0.660321	0.231025	0.349868	0.149987
TMEM177	13.12132	4.59082	0.349875	0.075028
CPLX2	0.015776	0.005522	0.350052	0.423873
TATDN3	12.82463	4.491085	0.350192	0.009499
FOXO4	8.788013	3.07884	0.350345	0.000206
ADGRB2	12.01869	4.211106	0.35038	0.007741
ATF7	6.893294	2.415323	0.350387	0.005701
GTF2B	27.91643	9.784294	0.350485	0.000844
ANKRD20A	0.016137	0.005657	0.350576	0.300344
CYTH3	8.590644	3.012927	0.350722	0.000471
TGM1	0.504258	0.176859	0.35073	0.076953
INTS4	11.65164	4.087107	0.350775	0.000153
SLC37A3	25.32265	8.887058	0.350953	0.000138
MCEE	17.08278	5.996577	0.351031	0.015021
SYNGAP1	5.380738	1.889308	0.351124	0.00044
RFC3	10.71912	3.765616	0.351299	0.013186
TBC1D3G	0.072157	0.025357	0.35141	0.042856
SLC30A4	1.286456	0.45214	0.351462	0.000959

DENND4C	4.100072	1.441042	0.351468	0.005062
GAS2L3	3.338701	1.173487	0.35148	0.000691
ZNF330	35.88289	12.61899	0.351671	0.002166
UPK3BL2	0.047809	0.016813	0.351672	0.202092
LOC10798	0.035059	0.01233	0.351696	0.459903
SHTN1	4.498434	1.582289	0.351742	0.004038
ZNF200	4.474294	1.573938	0.351773	0.000318
ARNT	23.54317	8.285738	0.351938	0.000528
ZNF503	59.58778	20.98633	0.352192	0.019122
PRELID3A	3.200304	1.127309	0.352251	0.000158
CHKB	25.04837	8.824321	0.352291	8.61E-05
DNAJC5B	0.109156	0.038459	0.35233	0.236232
CCDC66	8.387854	2.956764	0.352505	0.000307
STPG1	6.426475	2.265834	0.352578	0.005567
MTRNR2L	0.824493	0.290754	0.352646	0.082424
LOC10028	11.3522	4.003628	0.352674	0.000161
GLT1D1	0.049941	0.017617	0.352751	0.243381
SOX30	0.083196	0.029353	0.352812	0.102807
TYMSOS	7.128608	2.515387	0.352858	0.093029
MKS1	14.18183	5.005829	0.352975	0.005815
PCGF6	12.68481	4.4796	0.353147	0.009246
PIP5K1A	50.23269	17.74188	0.353194	0.000277
SLC3A1	0.042781	0.015111	0.35322	0.1093
NEK2	23.98725	8.473296	0.353242	0.000828
KBTBD8	1.729708	0.611107	0.353301	0.00674
PHGDH	126.1935	44.59204	0.353362	0.005676
ATXN7L2	3.137545	1.109017	0.353467	0.000563
OTUD3	5.50226	1.945576	0.353596	0.013872
TM7SF2	9.014423	3.187873	0.353641	0.021336
LOC10192	0.960103	0.33968	0.353795	0.052745
AASDHPP	15.25928	5.399715	0.353864	0.039282
ATG4C	13.30793	4.709745	0.353905	0.002494
POLR1B	10.86737	3.846288	0.35393	0.009773
MGEA5	46.14513	16.3362	0.354018	0.006189
GGPS1	36.39259	12.88415	0.354032	0.010748
WDCP	10.3427	3.662791	0.354143	0.00119
ZNF581	29.29475	10.37533	0.35417	0.018932
KCP	0.194717	0.068967	0.35419	0.119946
PDE10A	1.286609	0.456106	0.354503	0.000323
DAPL1	0.342058	0.121437	0.355019	0.153134
EFHB	0.422846	0.150178	0.355161	0.002905
CARD11	0.030165	0.010714	0.355163	0.503479
SCAF4	12.14387	4.316867	0.355477	0.01368
TBC1D16	6.986958	2.483854	0.355499	0.017565
CADM1	18.69932	6.650303	0.355644	0.003192
ZNF56	0.298673	0.10625	0.355738	0.083748
COPA	92.72676	33.00236	0.35591	0.006109
ALPPL2	0.037211	0.013245	0.355941	0.469136
ZSCAN26	7.485367	2.664843	0.356007	0.031082
RLIM	14.4377	5.140461	0.356044	0.004762
HPS4	8.41477	2.999664	0.356476	0.000957
CHRNB2	0.021624	0.007709	0.356489	0.145817
C17orf75	7.930592	2.827349	0.356512	0.006281
LTBP4	26.73185	9.531244	0.35655	0.004762
ZNF219	4.018309	1.433693	0.35679	0.000248
FKTN	3.02209	1.078303	0.356807	0.048108
AKT3	19.71349	7.036044	0.356915	0.000253
PHF3	9.906615	3.536625	0.356996	1.81E-06

BPHL	8.865582	3.165376	0.357041	0.048371
RNPS1	85.29687	30.46409	0.357154	0.005959
RBBP5	12.09246	4.32171	0.357389	0.000176
S100PBP	17.59653	6.290531	0.357487	1.06E-05
PYY	0.654921	0.23415	0.357525	0.344977
ZFAND4	1.743495	0.623571	0.357656	0.015156
CTAGE1	0.042924	0.015362	0.357898	0.42979
RBSN	12.38879	4.434864	0.357974	0.002137
ATP5MGL	0.281993	0.10107	0.358415	0.143809
KANSL3	16.93932	6.073329	0.358534	0.000807
SGSM3	30.52999	10.94914	0.358635	0.002621
GNAZ	2.059022	0.738482	0.358657	0.0017
MAPK12	26.16752	9.3874	0.358742	0.016017
PRDM11	0.130554	0.046844	0.358806	0.074919
SEMA4C	8.29418	2.976092	0.358817	0.001208
TESK2	3.116583	1.118327	0.358831	0.039866
LOC11226	0.147683	0.053032	0.359096	0.083386
PEX19	36.98225	13.28149	0.359131	0.000354
RPL26	2211.593	794.347	0.359174	0.003458
FAM53B	6.460386	2.320768	0.359231	0.000667
SLC17A8	0.023083	0.008293	0.359284	0.579042
USP43	1.120883	0.402718	0.359286	0.021819
IFNGR1	19.38354	6.968599	0.359511	0.001186
HNRNPA1	6.819677	2.452308	0.359593	0.000709
TLE6	1.576171	0.566869	0.359649	0.002415
NUDT11	9.340391	3.359921	0.359719	0.029047
PLEKHA1	6.380199	2.295109	0.359724	0.002117
CTPS2	7.598894	2.733982	0.359787	0.006382
MED12	7.613191	2.741239	0.360064	0.010271
HINT3	23.72515	8.543441	0.360101	0.000871
BCAS3	5.3353	1.921537	0.360155	0.008808
DDIT4	248.0066	89.41939	0.360553	0.012598
TAZ	20.44752	7.375853	0.360721	0.005346
ECI1	64.3922	23.23138	0.360779	4.51E-05
EP300	8.938831	3.225114	0.360798	0.00854
ICOSLG	0.059699	0.021564	0.361214	0.125728
MLLT10	5.750653	2.078852	0.361498	0.00063
MATN4	0.198451	0.071825	0.361928	0.082619
FAM186B	0.091533	0.033133	0.361981	0.094769
THOC2	28.3219	10.25295	0.362015	0.002889
LRRC37A	0.457343	0.165669	0.362242	0.003684
LOC10798	0.087036	0.031528	0.362244	0.097715
POLR3A	16.19475	5.869105	0.362408	0.000658
WASF1	21.85495	7.925976	0.362663	0.005991
KBTBD6	5.741181	2.082428	0.362718	0.001068
ZNF554	1.544103	0.560445	0.362958	0.005022
CCDC174	15.11248	5.488226	0.363159	0.001701
ZNF536	0.803318	0.291801	0.363245	0.000703
GRPEL2	17.17474	6.241555	0.363415	0.000131
PTHLH	0.85529	0.311029	0.363654	0.002767
GNL3	164.2727	59.74197	0.363675	0.016658
LRRC8D	12.25548	4.45724	0.363694	0.026945
DAP3	95.19857	34.62694	0.363734	0.000315
TEP1	2.084098	0.758086	0.363748	0.008519
ZNF548	3.93809	1.433328	0.363965	0.000612
DCAF12	23.1522	8.427249	0.363993	0.003258
RPS10	6.396636	2.328352	0.363996	0.001354
NUDT3	2.054885	0.748295	0.364154	0.038283

HIST1H4B	0.257535	0.093798	0.364214	0.582417
LPAR2	2.145405	0.781432	0.364235	0.011532
ZNF146	79.09956	28.82361	0.364397	1.42E-05
CHRNB1	6.081688	2.216972	0.364532	0.00176
LRRC40	19.08157	6.956397	0.364561	0.00394
AMH	6.623667	2.416989	0.364902	0.017584
RPL31	868.2559	316.8861	0.364969	0.001685
TNRC6C	0.890958	0.325223	0.365026	0.010962
RALGAPA:	2.33545	0.852585	0.365062	0.004072
TP53BP2	20.33839	7.430823	0.36536	0.004625
SLC22A18	0.046779	0.017093	0.365399	0.217288
RASA4B	0.771617	0.282042	0.365521	0.059514
ZNF182	7.141523	2.610603	0.365553	0.000307
TGFB3	1.394332	0.509826	0.365642	2.39E-05
POLG2	12.72868	4.658032	0.365948	0.000993
NUP205	63.50516	23.24097	0.36597	4.71E-05
HUWE1	30.01914	10.99688	0.366329	0.00114
DENND1B	0.527181	0.193155	0.366392	0.007202
NAA25	16.37514	6.000205	0.366422	0.001547
XPO4	2.364628	0.866495	0.36644	0.004471
HDAC5	14.07943	5.160867	0.366554	0.013533
SYCE3	0.868271	0.318361	0.366661	0.096832
BBS4	20.46231	7.50534	0.366788	0.001277
WASHC1	2.127154	0.780287	0.366822	0.002076
RPL29	1992.72	731.0121	0.366841	0.002261
FAM208B	4.549941	1.669361	0.366897	0.002018
NBEAL1	2.384729	0.875383	0.367079	0.000496
TRABD2B	0.37296	0.136973	0.367258	0.008988
CA14	0.281393	0.103355	0.367299	0.093983
ARID3C	0.597867	0.21969	0.367456	0.067799
TYW5	3.717146	1.366064	0.367503	0.002304
C11orf71	3.428425	1.261409	0.367927	0.104165
MINDY4	2.452363	0.902534	0.368026	0.03447
DENND6B	1.598037	0.588146	0.368043	0.011409
LZTS3	6.87561	2.530551	0.368047	0.001682
NME3	35.2287	12.9682	0.368115	0.004035
RNF219	26.35419	9.701656	0.368126	0.001008
DOT1L	13.96701	5.141777	0.368137	0.006813
SUPT16H	76.95747	28.33891	0.368241	0.002308
TEX13D	0.032009	0.011789	0.368302	0.41385
ABI3BP	2.202449	0.811616	0.368506	0.065196
ARHGEF2(	4.915206	1.811889	0.368629	0.000158
ALDH2	40.8894	15.08329	0.36888	0.000817
ZNF250	2.171529	0.801076	0.3689	0.005053
NCOA7	5.329918	1.966428	0.368942	0.006743
EFCAB7	5.672369	2.092827	0.368951	0.00584
STX16	59.55666	21.97357	0.368952	0.004579
UVRAG	3.079702	1.137466	0.369343	0.00031
A1BG	6.977214	2.577981	0.369486	0.003809
TSSK6	1.748391	0.646038	0.369504	0.10436
MARS	152.853	56.48862	0.369562	0.021355
ZNF768	45.89579	16.97071	0.369766	0.003017
ABHD16B	0.074732	0.027642	0.369881	0.158065
CCDC14	13.27733	4.912213	0.36997	0.006665
KCNK9	0.115611	0.042776	0.370004	0.089806
UQCC2	64.84161	24.0053	0.370214	0.000669
MFSD9	3.022861	1.119179	0.370238	0.005531
ITFG2	5.953757	2.204503	0.370271	0.00279

DGKH	2.077961	0.769945	0.370529	0.00482
NEIL3	18.01253	6.674312	0.370537	0.009907
RPS25	2196.766	814.085	0.370583	0.005666
ZDHHC21	1.46265	0.54245	0.370868	0.006816
LOC10012	0.502364	0.186316	0.370879	0.002083
ZFP62	7.495504	2.781083	0.371034	0.009489
FRA10AC1	15.0317	5.577386	0.371042	0.002809
GADD45B	28.97359	10.75277	0.371123	0.185442
RPL32	1500.535	556.9157	0.371145	0.001404
FAM117A	5.013103	1.860912	0.37121	8.73E-05
PLA2G4A	12.97564	4.816806	0.371219	0.055576
HOXA4	8.480844	3.148872	0.371292	0.006143
SP7	0.037685	0.013996	0.371388	0.385769
ARHGAP2	1.125957	0.418202	0.371419	0.022841
LOC10537	0.255236	0.094809	0.371457	0.282526
ARV1	37.53111	13.94601	0.371585	0.005385
TAF3	5.298516	1.97008	0.371817	0.001955
YEATS4	32.04922	11.91682	0.371829	0.038897
EGR3	0.267397	0.099438	0.371875	0.100494
RASGRF1	0.0385	0.01433	0.372211	0.382514
METAP2	58.98992	21.96174	0.372296	0.021367
KLHDC2	21.09795	7.861093	0.3726	0.016376
RPS6	2748.927	1024.252	0.372601	1.13E-05
FBXO21	21.91396	8.170656	0.372852	0.000305
VSIG1	0.037641	0.014042	0.373054	0.435246
ZRANB3	2.152838	0.803366	0.373166	0.028968
LRRC73	0.891726	0.332885	0.373303	0.147098
PLPP7	0.964906	0.360432	0.373541	0.106288
RACK1	3951.277	1476.011	0.373553	0.000922
SLX4	2.315032	0.865052	0.373667	0.005278
PRPS1	111.9737	41.8455	0.373708	0.003825
RAMP1	5.934034	2.217831	0.373748	0.011425
RPAP2	4.021154	1.503361	0.373863	0.014584
CHD2	17.58428	6.576641	0.374007	0.006191
TTF2	8.8997	3.329486	0.374112	0.005952
FOXG1	11.04916	4.134175	0.374162	0.001885
CDK6	6.15539	2.305002	0.374469	0.036134
PIWIL2	0.085076	0.031863	0.374525	0.139838
RTL10	5.151564	1.929493	0.374545	0.000288
PAX6	3.335062	1.249575	0.374678	0.000565
WRNIP1	31.80318	11.92268	0.37489	0.001021
ZC3H11A	39.2466	14.7255	0.375204	9.56E-05
COQ10A	14.99562	5.627759	0.375293	0.026727
GGA3	15.32039	5.750241	0.375332	0.004205
SNRPA	152.6458	57.29537	0.375349	0.000414
SREBF2	37.15748	13.96946	0.375953	0.001346
NUP155	25.18079	9.468526	0.376022	0.000778
RPS6KB1	12.45895	4.684937	0.37603	2.18E-06
HNRNPC	277.3181	104.2899	0.376066	0.00012
MCM7	158.5819	59.63893	0.376076	0.000395
FNDC3A	7.111306	2.674419	0.37608	0.001575
CXorf40A	4.085377	1.536744	0.376157	0.012919
DENND3	2.110042	0.794593	0.376577	0.00653
KANSL2	18.91382	7.124421	0.376678	0.000917
OCRL	20.40966	7.688642	0.376716	0.001213
PDZD8	7.182686	2.706552	0.376816	0.000732
NUDT12	8.27706	3.120906	0.377055	0.012044
PRR29	0.286972	0.108313	0.377433	0.129541

SNRPC	173.6455	65.54442	0.377461	0.00134
CNNM2	4.598984	1.736214	0.377521	0.003884
GPATCH2	3.307263	1.24861	0.377536	0.027028
PABPC1L	12.34908	4.662282	0.377541	0.034663
EP400	12.18439	4.604282	0.377884	0.007189
PARD6G	2.017084	0.762266	0.377905	0.004349
ZNF277	16.98898	6.422567	0.378043	0.025017
JDP2	2.167349	0.819565	0.378142	0.000657
ITGB8	1.133536	0.429138	0.378583	0.126284
TBC1D12	3.095555	1.172077	0.378632	0.000305
TTC33	5.153497	1.952355	0.378841	0.001775
ATP8B2	16.07896	6.091377	0.378842	0.006996
SUPT7L	18.29205	6.929891	0.378847	0.006746
DDX55	14.47347	5.486041	0.379041	0.000303
ANAPC1	6.815618	2.583467	0.379051	0.002317
MIS18BP1	5.179718	1.964043	0.37918	0.000106
MXD1	3.113398	1.181621	0.379528	0.132084
ELOVL4	7.572846	2.874574	0.37959	0.003035
RUFY3	6.542178	2.483984	0.379688	9.02E-06
THSD1	1.278469	0.485504	0.379755	0.001416
CELF6	0.14342	0.054481	0.379871	0.229717
NIT1	17.72508	6.739009	0.380196	2.25E-05
KITLG	4.272292	1.624886	0.380331	7.40E-05
NSUN6	3.01172	1.145538	0.38036	0.024023
XPR1	7.287468	2.772311	0.380422	0.000919
CDC23	40.76301	15.5142	0.380595	1.08E-05
BEND7	2.965311	1.128817	0.380674	0.000675
KLHL30	0.127474	0.048558	0.380925	0.099211
RPL8	2744.419	1045.466	0.380943	0.000582
LEKR1	0.151411	0.057684	0.380973	0.190024
CCZ1	12.31462	4.695296	0.381278	0.000857
FOXD2	1.135152	0.43287	0.381333	0.000511
CDC6	70.4367	26.86737	0.38144	2.31E-05
NUP160	19.02784	7.261081	0.381603	0.023906
MGA	4.400116	1.680427	0.381905	0.015013
RMI2	53.53287	20.4473	0.381958	8.18E-06
POLD3	16.9225	6.464667	0.382016	3.43E-05
INPPL1	27.01968	10.327	0.382203	0.002623
CHD1	12.66815	4.842241	0.382237	0.000214
ZNF281	8.974827	3.431285	0.382323	0.016046
WWC2	3.996231	1.528027	0.382367	0.000182
MRPS25	20.96061	8.017529	0.382505	0.000391
PAQR8	6.254209	2.392278	0.382507	0.019541
NF1	7.420107	2.838504	0.382542	0.006462
EML4	19.71323	7.541589	0.382565	0.001602
SUGP2	37.47664	14.33731	0.382567	0.00462
EEF1G	2756.079	1054.922	0.382762	0.002209
ZNF3	7.768325	2.974999	0.382965	0.005564
BLMH	39.09115	14.97116	0.382981	0.000656
BBS10	7.290036	2.793019	0.383128	0.017421
WDR44	8.92405	3.41926	0.383151	0.000916
SLC10A7	1.10544	0.423555	0.383155	0.012427
CFI	8.142229	3.120635	0.383265	0.008317
LOC10272	0.027853	0.010677	0.38334	0.207838
TIGD4	0.66754	0.255945	0.383415	0.009237
ARMCX5	6.945336	2.66506	0.383719	0.041873
SRPK2	8.991023	3.450158	0.383734	0.000214
ZNF211	4.39825	1.689887	0.384218	0.006428

YY1AP1	39.87282	15.32096	0.384246	0.000513
STX11	0.236924	0.091044	0.384276	0.043525
ITSN2	3.190477	1.226139	0.384312	0.014566
RIMBP3C	0.029438	0.01132	0.384529	0.13681
LOC10537	0.043032	0.016559	0.384808	0.375421
AXIN1	9.222022	3.550559	0.385009	4.05E-06
NT5DC3	3.156617	1.215548	0.385079	0.000558
TRIM39	3.61412	1.392101	0.385184	0.012917
FASN	26.16475	10.07938	0.385228	0.005333
FGFBP2	0.805425	0.310283	0.385242	0.15599
ALG11	1.82454	0.702939	0.385269	0.002917
ZNF550	2.11857	0.81629	0.385302	0.00098
MESP1	5.853308	2.256941	0.385584	0.009726
NLE1	8.724965	3.365615	0.385745	0.065451
GALT	23.41668	9.04249	0.386156	0.000993
ATAT1	5.319033	2.054034	0.386167	0.003389
RBM6	44.48911	17.18665	0.386311	0.021596
RPL7L1	62.00983	23.95891	0.386373	0.005761
TRIM23	5.303289	2.050371	0.386623	0.002463
CABIN1	9.12266	3.527415	0.386665	0.000432
PHETA2	0.191627	0.074105	0.386714	0.088819
SENP6	8.804705	3.405778	0.386813	0.007172
SMAD5	15.11126	5.846705	0.386911	0.004415
RSBN1L	6.749786	2.611959	0.386969	0.00269
PLBD1	0.067182	0.025998	0.386984	0.451311
PKD1	5.74534	2.224479	0.38718	0.040898
SLC25A4	6.60399	2.557484	0.387263	0.000324
CASQ1	0.086217	0.033391	0.387287	0.145323
BCL6	7.906299	3.062154	0.387306	0.066093
TMTC3	6.379887	2.472321	0.387518	0.005406
GPR156	0.168172	0.065188	0.387626	0.020679
FITM1	0.169895	0.065864	0.387676	0.057064
USP1	55.87274	21.67352	0.387909	0.034573
LOC64421	1.792419	0.695322	0.387924	0.031248
RAB24	38.6884	15.00962	0.387962	0.000152
MDN1	5.87477	2.279835	0.388072	0.020247
SCYL2	8.411026	3.264578	0.388131	0.010017
DDI2	1.03976	0.403748	0.388308	0.024126
INTS2	4.319436	1.678082	0.388496	0.006114
MTRNR2L	2.170869	0.843591	0.388596	0.010256
PIBF1	18.2641	7.100147	0.388749	0.008306
CCNA1	0.093287	0.036285	0.388966	0.3028
ZSCAN2	5.498473	2.139465	0.389102	0.005367
EYA3	12.90943	5.02394	0.389168	0.000796
CPLANE1	2.277206	0.886634	0.389352	0.00305
PIKFYVE	4.443062	1.730309	0.389441	0.015844
KAT2A	34.14309	13.30251	0.389611	0.004563
DIEXF	5.111683	1.991593	0.389616	0.009896
ZBTB18	5.377731	2.095691	0.389698	0.000304
FRAT1	2.019406	0.78708	0.389758	0.164871
NOP56	127.0853	49.54424	0.38985	0.003703
VWA8	4.71714	1.839581	0.389978	0.0137
TBC1D2B	10.6576	4.156407	0.389995	0.011472
RASA4	0.912479	0.355952	0.390093	0.201193
ADAM22	2.515464	0.981394	0.390144	0.000546
CAMSAP2	7.507053	2.92972	0.390262	0.007497
CA3	0.334589	0.1306	0.39033	0.029884
SYT15	0.023309	0.009102	0.390491	0.042261

ARMCX3	20.30391	7.928576	0.390495	0.017503
CASTOR2	4.124059	1.61118	0.390678	0.001815
TBP	22.8746	8.939449	0.390803	2.35E-05
RNF43	1.370403	0.535671	0.390886	0.00692
DEPP1	52.64489	20.57871	0.390897	0.032622
NUCKS1	141.627	55.37763	0.391011	0.018418
USP24	13.34874	5.221436	0.391156	0.005263
GLI4	13.44736	5.260795	0.391214	0.000136
ZNF674	1.180169	0.461699	0.391214	0.052353
NOS3	0.073132	0.028614	0.391257	0.093493
WRN	6.148287	2.40583	0.391301	0.002346
ZCCHC14	5.049273	1.976027	0.391349	0.008256
ZNF236	1.437604	0.562713	0.391424	0.029334
ZNF263	11.10211	4.345705	0.391431	0.000106
ZBTB43	6.084136	2.385018	0.392006	0.084255
ACAT1	35.3626	13.87335	0.392317	0.031769
NBPF9	5.311058	2.083917	0.392373	0.004298
PCMTD2	12.76756	5.009724	0.392379	0.01797
DPF2	32.8216	12.8796	0.392412	0.00288
LUC7L2	4.217586	1.655039	0.392414	0.00144
PRMT3	13.10305	5.141955	0.392424	0.030942
ADAR	28.53597	11.20194	0.392555	0.000709
BTBD16	0.110304	0.0433	0.392556	0.175041
KLHL17	9.131484	3.584813	0.392577	0.021611
BAG4	8.746631	3.434256	0.392638	0.000595
USP36	12.05366	4.734441	0.39278	0.036743
CCDC112	11.35718	4.46272	0.392943	0.008644
LRRC1	2.197115	0.863715	0.393113	0.001636
SLX4IP	0.955437	0.375727	0.393251	0.002658
DPH7	7.546679	2.968185	0.39331	0.003978
MAGI3	2.080705	0.819148	0.393688	0.012191
SH3BP1	11.35136	4.470337	0.393815	0.002081
LSM2	91.22287	35.92749	0.393843	0.000314
TAF1A	5.111312	2.013159	0.393863	0.00109
ZNF598	23.81794	9.382165	0.393912	0.000225
LBH	6.183535	2.436512	0.394032	0.008767
ZNF862	2.489823	0.981094	0.394042	0.001208
PIM3	58.88074	23.20807	0.394154	0.018611
MSL2	11.2909	4.455298	0.394592	0.001774
SRRM5	0.655033	0.258484	0.394611	0.048696
IP6K2	39.27074	15.5012	0.394726	0.006055
AKAP3	0.389545	0.153769	0.39474	0.167258
ESF1	9.711179	3.833423	0.394743	0.005082
TRIM29	0.120866	0.047732	0.394917	0.252812
TEKT5	0.129594	0.051182	0.394939	0.078011
MBIP	20.10479	7.940615	0.394961	0.007993
C19orf48	82.0604	32.41586	0.395024	0.028373
CEP164	6.719013	2.654483	0.39507	0.003494
RHOT2	21.31212	8.41988	0.395075	0.000282
TOP2A	144.9893	57.29511	0.395168	0.000293
RFC2	92.39153	36.54775	0.395575	0.002479
FBXL4	4.5837	1.813632	0.39567	0.000625
CPSF4	14.51913	5.745427	0.395714	0.001011
CNOT1	51.92619	20.54899	0.395735	0.008828
ARL8A	24.67934	9.770707	0.395906	1.97E-05
LRRC37B	2.786282	1.103636	0.396096	0.000452
BRAF	5.407437	2.14199	0.396119	0.008688
TMEM241	3.763942	1.491241	0.396191	0.001806

XBP1	216.8124	85.90203	0.396204	0.007888
ALPK3	0.079805	0.031623	0.396256	0.071158
PCBD1	28.55248	11.31726	0.396367	0.000651
PNPT1	15.7081	6.226448	0.396385	0.017577
C16orf89	0.074149	0.0294	0.3965	0.510771
FAM19A2	0.152566	0.060503	0.396569	0.126106
NES	11.41917	4.529176	0.396629	0.025474
SLC45A4	0.604997	0.240033	0.39675	0.00115
BRPF3	3.612221	1.433326	0.396799	0.017048
CHEK2	23.86343	9.472115	0.39693	0.023565
FAM20A	0.045422	0.018035	0.397053	0.110879
NT5C3A	20.26324	8.047667	0.397156	0.014676
GPR135	0.761135	0.302415	0.397321	0.008454
SHROOM:	0.917677	0.364615	0.397324	0.005185
TLE1	24.8423	9.873012	0.397427	0.008657
TRIM27	30.38507	12.0783	0.397508	0.006797
MDM1	3.460789	1.375857	0.397556	0.000503
CCNL1	19.32823	7.685203	0.397615	0.03158
CBX5	35.5059	14.12068	0.3977	0.060591
POGK	9.39263	3.736847	0.397849	0.000278
ANGPT2	0.045365	0.01805	0.39787	0.122066
PIGZ	0.544568	0.216671	0.397878	0.023956
HAUS6	17.29916	6.883627	0.397917	0.003496
EEF2	1951.542	776.6764	0.397981	0.005901
CSPP1	3.06383	1.219471	0.398022	0.00151
ELP1	25.02196	9.959693	0.398038	0.000456
PIGF	28.04543	11.17205	0.398355	0.007358
TMEM68	7.204069	2.870291	0.398426	0.001959
LHX9	0.100594	0.040089	0.398525	0.075804
PPCDC	5.503409	2.193702	0.398608	0.100741
BOLA1	21.43846	8.547004	0.398676	0.013668
POLL	10.23659	4.082261	0.398791	0.011092
MOB1B	5.007816	1.997103	0.398797	0.007172
LAG3	0.224863	0.089682	0.398829	0.082089
FANCI	64.59981	25.7743	0.398984	0.000207
ZNF286B	0.960474	0.383281	0.399054	0.008718
L3MBTL1	2.738779	1.092965	0.39907	0.014182
SLC35E2B	7.530453	3.005337	0.399091	0.000593
C18orf54	4.105131	1.638726	0.39919	0.001888
TSC22D2	6.382858	2.548103	0.39921	0.00029
HEATR6	7.809313	3.117853	0.399248	1.53E-05
YIPF4	20.6298	8.23697	0.399275	0.001432
MKL1	9.126814	3.644511	0.399319	0.001593
PIGQ	14.42162	5.75983	0.399389	0.033036
C22orf39	19.75381	7.889644	0.399399	6.14E-05
ALOX12B	0.11291	0.045114	0.399555	0.130639
MND1	37.30534	14.91057	0.39969	0.006745
FGFR1OP2	23.75794	9.499185	0.399832	2.77E-05
SOGA3	1.240026	0.495811	0.399839	0.003793
DIAPH3	5.965121	2.385973	0.399987	0.003671
KLRG1	0.676576	0.270631	0.400001	0.136445
SNRPE	107.7038	43.12692	0.400422	0.002484
NSMCE4A	34.24066	13.71993	0.400691	0.019332
ZNF740	17.73424	7.106797	0.400739	0.001235
ATP6V0A1	18.7168	7.501453	0.400787	0.00011
PSMG3	41.42812	16.60652	0.400851	0.002587
HNRNPH3	163.6069	65.58748	0.400884	0.011412
LIPT2	1.418072	0.568636	0.400993	0.141221

LRRCC1	4.725688	1.895621	0.401131	0.059174
AGPAT4	7.503077	3.010987	0.4013	0.003286
CCAR1	33.63078	13.49863	0.401377	0.018619
PLAC8	0.473179	0.189964	0.401462	0.239945
SPRR2D	1.069856	0.429595	0.401545	0.305656
ANP32C	3.065313	1.231241	0.401669	0.117314
DNAAF2	5.278338	2.121245	0.401878	0.013071
EML2	22.34537	8.981639	0.401946	0.005356
FOXD4L1	0.138298	0.055601	0.402039	0.202399
ZNF396	0.991848	0.398806	0.402083	0.139824
CLEC2D	3.471697	1.396209	0.402169	0.001139
GMPR2	27.4377	11.04814	0.402663	0.004349
METTL22	3.677722	1.481457	0.402819	0.000884
UNC13B	6.672822	2.688138	0.402849	0.000502
ARHGEF12	13.16108	5.305164	0.403095	0.002412
PPFIBP2	0.620212	0.250109	0.403264	0.099534
TDG	12.08234	4.872888	0.403307	0.000594
C8orf44	2.519276	1.016136	0.403344	0.011209
CCR10	2.247815	0.907272	0.403624	0.008807
HIP1R	9.863276	3.983209	0.403842	0.006076
LINC0221(	3.059643	1.235673	0.403862	0.002035
TMEM94	12.89166	5.207395	0.403935	0.00289
KIAA1024	0.51907	0.2098	0.404185	0.014052
JHY	0.985251	0.398278	0.40424	0.003723
TTC23L	0.115633	0.046745	0.404248	0.166066
ZNF543	2.034956	0.823024	0.404443	0.128947
CHRNA5	11.03355	4.463022	0.404496	0.017992
CDK17	4.722307	1.910731	0.404618	0.007105
HNRNPH1	116.6695	47.20705	0.404622	0.037076
MORC3	7.578956	3.066718	0.404636	0.002376
COX15	10.95601	4.435053	0.404806	0.003609
ZC3H11B	0.869916	0.352205	0.404872	0.036354
HIST1H3H	1.27845	0.517974	0.405158	0.036736
RPP25	4.017445	1.62776	0.405173	0.051011
NEURL4	9.833763	3.985016	0.405238	0.000271
WARS2	9.8677	4.00007	0.40537	0.016642
CALHM6	0.231722	0.093937	0.405388	0.065656
WRAP73	16.34742	6.62762	0.405423	2.60E-06
RRP1B	22.78273	9.245559	0.405814	1.98E-05
PDPK1	2.074115	0.841943	0.405929	0.009533
EOMES	0.57328	0.232778	0.406046	0.060664
MED4	20.67478	8.399069	0.406247	0.000176
TWNK	8.834535	3.589064	0.406254	0.007268
WDR31	1.852162	0.752492	0.406278	0.157352
SFI1	10.53409	4.28117	0.406411	8.79E-06
CCDC82	3.380362	1.374755	0.406689	0.012104
CEP85L	1.479656	0.602056	0.406889	0.000535
MAN2A2	14.66563	5.970365	0.407099	0.00133
MYH3	0.505544	0.205882	0.407249	0.025808
NUPL2	14.37289	5.853947	0.407291	0.002042
ZNF484	1.87516	0.763917	0.407387	0.003991
HLTF	33.91687	13.81814	0.407412	0.024575
ZNF283	1.024317	0.417339	0.407431	0.010356
ZMAT1	0.080879	0.032957	0.407479	0.263061
KMT2B	8.14434	3.318893	0.407509	0.007659
KRTCAP3	0.675725	0.275369	0.407516	0.220163
DBT	4.029659	1.642743	0.407663	0.00255
ACBD4	3.177433	1.29557	0.407741	0.003606

FSD1L	1.255076	0.511805	0.407788	0.028781
CCDC77	18.84114	7.683503	0.407805	0.000845
SATB1	2.487714	1.0151	0.408045	0.01767
RXRB	23.84459	9.731182	0.408109	0.000117
RNF38	5.321122	2.172019	0.408188	0.000526
DNMT1	59.20661	24.17152	0.408257	0.003321
THOC6	16.83166	6.873805	0.408385	0.000827
PSPC1	5.232923	2.137451	0.408462	0.004741
KIAA0319	0.193806	0.079201	0.408664	0.048419
C19orf73	1.94239	0.793935	0.408741	0.158621
TSHZ1	5.750496	2.351144	0.408859	0.042669
NBPF8	5.973083	2.443155	0.409028	0.000694
PPIL4	35.31732	14.44782	0.409086	1.13E-05
CAMKMT	0.65749	0.268998	0.409129	0.020212
LOC10798	0.229777	0.094046	0.409293	0.164343
IL12A	2.173942	0.890523	0.409635	0.024081
TMED4	50.46774	20.67344	0.409637	0.000277
CENPT	23.22478	9.516027	0.409736	0.007248
ATP5MC2	112.3635	46.04527	0.409788	3.53E-05
DENND4B	15.64987	6.416014	0.409972	0.001182
CCDC40	0.741846	0.304281	0.410168	0.002895
PYCR2	78.69649	32.28105	0.410197	0.003441
STK38L	4.554278	1.86818	0.410203	0.000902
HIVEP1	1.739023	0.713388	0.410223	0.005769
H3F3C	2.010165	0.824665	0.410248	0.016482
SMYD4	8.076517	3.313574	0.410273	0.00219
RPS3A	1442.837	592.2096	0.410448	0.00051
PLD1	3.731438	1.532081	0.410587	0.000293
TDRD3	7.003345	2.875837	0.410638	0.024382
FBL	512.6089	210.5775	0.410796	0.00112
LEMD3	5.732941	2.355204	0.41082	0.007001
ATRX	7.437662	3.055752	0.410848	0.035472
ULBP1	5.022114	2.063427	0.410868	0.040141
ATP5S	1.12541	0.462527	0.410985	0.020575
PCOLCE2	11.95777	4.915274	0.411053	0.002962
CDK5RAP3	48.29943	19.86104	0.411207	0.021238
FAR2	3.412761	1.403368	0.411212	0.043229
EXOC2	6.896022	2.837514	0.411471	0.001203
RPP40	7.459098	3.069232	0.411475	0.019625
GTF2H3	25.52074	10.50206	0.411511	0.052838
CKS1B	265.9017	109.4418	0.411587	0.000796
FOXD4L6	0.014318	0.005893	0.411612	0.615282
CSRP3	0.026651	0.01097	0.411612	0.615282
H2BFS	0.084819	0.034912	0.411612	0.615282
TRIM31	0.018876	0.00777	0.411612	0.615282
NCF4	0.017045	0.007016	0.411612	0.615282
GRXCR2	0.018421	0.007582	0.411612	0.615282
TSPEAR	0.009684	0.003986	0.411612	0.615282
CDC73	9.438271	3.885113	0.411634	0.004073
BTRC	5.898146	2.428003	0.411655	0.015013
TIRAP	3.468818	1.428496	0.411811	0.021142
LYPD1	10.12003	4.171055	0.412158	0.049449
PDE3A	0.016927	0.006977	0.412164	0.438332
NONO	305.2642	125.8728	0.41234	1.28E-06
GJB2	1.629268	0.671957	0.412429	0.024042
ACVR2A	2.808681	1.159136	0.412698	0.000223
ABHD4	9.82831	4.056554	0.412742	0.003791
TBCEL	3.957351	1.63344	0.412761	0.003981

USP35	3.251448	1.342138	0.412781	0.005031
CCDC121	0.954154	0.39409	0.413026	0.214748
USP33	25.47019	10.52308	0.413153	0.0033
TM2D3	32.28578	13.34181	0.413241	0.000288
NLN	10.31246	4.261737	0.413261	0.019726
LIAS	14.54665	6.015068	0.413502	0.000372
FBRSL1	9.635449	3.985781	0.413658	2.39E-05
ASCC1	10.11189	4.183552	0.413726	4.35E-06
CCDC84	10.0066	4.140256	0.413752	0.031605
RB1CC1	10.68546	4.421745	0.413809	0.001592
ZNF316	6.401899	2.649269	0.413825	0.000409
LOC44115	0.643117	0.266145	0.413837	0.024118
NEDD1	11.16675	4.622198	0.413925	0.002368
ABT1	18.88422	7.818639	0.41403	0.001331
RPL4	3329.785	1378.846	0.414095	0.004122
SULT1C2	0.806291	0.333952	0.414184	0.044563
DDX10	18.65806	7.734555	0.414542	0.04329
ABCA7	2.762401	1.145499	0.414675	2.57E-05
PRDM10	1.591701	0.660101	0.414714	0.004411
SCFD1	31.17042	12.92709	0.414723	0.000783
FASTKD1	6.135032	2.544963	0.414825	0.000361
CDK8	6.900566	2.862708	0.414851	0.025818
PARD3B	0.489296	0.203035	0.414953	0.03727
SETD6	7.853311	3.258936	0.414976	0.004225
ACBD7	2.81799	1.169463	0.414999	0.002738
IMMP2L	0.18643	0.077369	0.415003	0.056604
CBLN1	0.291243	0.120869	0.415012	0.104955
SRP9	334.3863	138.83	0.415178	0.000236
STX3	9.692736	4.025538	0.415315	0.013741
CDADC1	2.726783	1.132552	0.415344	0.007486
CNST	3.71632	1.54376	0.4154	0.000349
KCNE3	0.034037	0.014145	0.41559	0.54244
RSL24D1	199.2341	82.82673	0.415726	0.000725
MMS22L	4.979147	2.070705	0.415875	4.17E-05
GDF9	0.31368	0.130489	0.415994	0.101139
COL4A6	2.746704	1.143449	0.416299	0.009347
MYH11	0.050885	0.021193	0.416502	0.259278
MED20	12.94285	5.392464	0.416636	0.003638
TFAM	14.23414	5.931143	0.416684	0.009308
TMEM91	6.366617	2.654118	0.416881	0.015947
SNAPC3	13.94765	5.818129	0.41714	2.09E-05
ZNF559	4.343199	1.811968	0.417197	0.018076
UNG	42.46454	17.71701	0.417219	0.014573
G2E3	4.369695	1.823613	0.417332	0.000538
ZNF555	1.53081	0.639168	0.417536	0.002091
WDR88	0.022776	0.009511	0.417608	0.503358
P4HA3	0.390404	0.163047	0.417638	0.128229
KRIT1	6.881243	2.875465	0.41787	0.007915
B4GALT3	27.40221	11.45157	0.417907	0.000129
PHB2	283.5956	118.5226	0.417928	2.42E-05
SUZ12	19.61886	8.203288	0.418133	0.000462
SMARCA5	28.27175	11.8216	0.418142	7.43E-05
KMT5B	7.614351	3.184267	0.418193	0.008782
PIH1D1	47.92267	20.05129	0.418409	0.01205
ZBED1	0.091026	0.038095	0.418508	0.005155
TLK2	9.512518	3.981566	0.418561	0.000835
NOD1	2.269408	0.94989	0.418563	0.002632
NPIPB12	0.616574	0.258089	0.418585	0.012748

STK35	6.289876	2.633406	0.418674	0.017043
HCFC2	2.624195	1.098695	0.418679	0.016852
TMEM120	9.073612	3.799065	0.418694	3.55E-05
DLG2	0.062234	0.026057	0.418698	0.034489
SYNE2	2.776162	1.162603	0.418781	0.008663
SLC6A20	0.018487	0.007743	0.418858	0.432043
DEFB124	0.131008	0.054874	0.418858	0.432043
PAIP2	77.17286	32.32803	0.418904	0.000687
TAF1B	11.72336	4.913179	0.419093	0.010597
CCBE1	0.979846	0.410708	0.419156	0.042222
NOLC1	59.60968	24.98581	0.419157	0.000132
PTCD3	14.04197	5.886668	0.419219	0.011207
ANKS3	6.450309	2.705099	0.419375	0.000661
PRKCH	2.316452	0.971637	0.41945	0.011085
LYPLAL1	4.687202	1.966831	0.419617	0.109825
ZMYND12	0.064681	0.027143	0.419645	0.467219
SLC7A1	32.44897	13.62765	0.419972	0.075189
AMMECR1	7.129162	2.994062	0.419974	0.006055
RINT1	14.75349	6.196694	0.420016	0.00155
MICB	3.578993	1.503643	0.42013	0.004851
BRSK1	13.9542	5.865426	0.420334	0.00965
SF3A2	103.2276	43.40323	0.420461	0.001514
SCX	2.523074	1.060981	0.420511	0.148583
C1orf109	9.072847	3.816116	0.420608	0.029081
CMYA5	0.060163	0.025305	0.420613	0.212067
RPS8	3062.923	1288.478	0.420669	0.001039
CACFD1	2.406634	1.012549	0.420733	0.007667
SPATA9	0.119796	0.050429	0.420961	0.063034
FANCA	15.87607	6.684862	0.421065	0.00029
CUL7	13.97173	5.884301	0.421158	0.000991
GRAMD2B	1.144891	0.482195	0.421171	0.000707
CDC45	52.99498	22.32528	0.421272	0.009253
ZBTB49	2.071575	0.872779	0.421312	0.002408
PPEF2	0.009658	0.00407	0.42138	0.426408
PLA1A	0.019307	0.008136	0.42138	0.426408
CARD14	0.007333	0.00309	0.42138	0.426408
CSF2RB	0.007667	0.003231	0.42138	0.426408
MEFV	0.06076	0.025609	0.421483	0.514901
SLIT3	0.921862	0.388643	0.421585	0.076903
DYM	8.617981	3.634441	0.421728	2.11E-06
MED13	7.311749	3.083569	0.421728	0.025587
ALG13	5.677086	2.394358	0.421758	0.012467
EPHA5	0.383361	0.161717	0.421841	0.00899
C20orf196	0.881282	0.371814	0.421901	0.020297
RBBP7	202.1423	85.30403	0.422	0.001866
BTN2A1	14.36381	6.061557	0.422002	0.001998
N4BP2L1	0.441462	0.186332	0.42208	0.127805
ZMYM3	16.29643	6.882445	0.422328	0.005311
ZNF500	1.972067	0.833284	0.422543	0.006567
TSACC	0.520933	0.220206	0.422715	0.082855
ATL2	10.33462	4.369816	0.422833	0.001182
LOC10798	0.150413	0.063656	0.423208	0.482651
CHAT	0.014302	0.006054	0.423315	0.623494
HYAL1	0.188289	0.079733	0.423459	0.119615
DTNB	3.430693	1.452822	0.423478	0.000935
BRIP1	3.058932	1.295541	0.423527	0.01231
C6orf136	10.54753	4.468584	0.423662	0.000415
CHRNA2	0.009183	0.003891	0.42378	0.429289

OLFML1	0.010615	0.004499	0.42378	0.429289
RHAG	0.018414	0.007804	0.42378	0.429289
HEPHL1	0.006368	0.002699	0.42378	0.429289
ITIH1	0.011639	0.004932	0.42378	0.429289
TMEM72	0.006268	0.002656	0.42378	0.429289
AASS	6.775703	2.872116	0.423885	0.001052
KIF3A	6.805108	2.884836	0.423922	0.001153
SMC3	52.61154	22.3069	0.423992	0.004467
POLR3F	11.74353	4.980001	0.424063	0.00063
TRIM4	14.73061	6.247592	0.424123	0.000118
RAB11FIP2	3.77269	1.600159	0.424143	0.000298
RBM23	14.80593	6.28332	0.424379	0.001581
ZNF143	9.848528	4.18022	0.424451	0.062088
FABP6	0.98913	0.420129	0.424746	0.129904
DKC1	78.88086	33.50728	0.424783	0.000386
LARS	99.72014	42.37483	0.424938	0.036007
DYRK2	5.381678	2.288396	0.42522	0.021331
NRL	0.17638	0.075017	0.425315	0.080086
FILIP1	0.236963	0.100818	0.425458	0.067596
GPR161	3.533797	1.503894	0.425574	0.002588
PTPRA	23.32094	9.92531	0.425596	0.000361
CAPN15	6.409023	2.727774	0.425615	7.91E-05
C5orf51	1.015306	0.432144	0.425629	0.092182
MGAM2	0.032792	0.013961	0.425754	0.450846
NSD1	6.381625	2.718024	0.425914	0.022784
PIDD1	8.260432	3.518685	0.425969	0.002772
BAZ2B	1.449914	0.61762	0.42597	0.02047
DMXL2	5.430866	2.314289	0.426136	0.027176
PPARGC1A	0.781808	0.333226	0.426224	0.007296
VPS13A	5.066561	2.159821	0.426289	0.02579
KIF27	0.765479	0.326324	0.4263	0.001467
EAF1	13.24413	5.647458	0.426412	0.000455
MAD2L1	117.8943	50.2753	0.426444	0.006618
SARS	333.981	142.4556	0.426538	0.006771
HHLA3	16.29864	6.954473	0.42669	0.024877
BFSP1	1.366037	0.582962	0.426754	0.078432
CASC3	42.51962	18.14635	0.426776	0.006993
TRIB1	14.15646	6.04217	0.426814	0.006518
CCDC78	0.440685	0.188094	0.426821	0.261832
FAM78B	0.385689	0.164632	0.426851	0.08929
FRMD5	2.411715	1.029832	0.427012	0.015845
HBS1L	29.78821	12.72258	0.427101	0.000183
TBC1D15	13.67289	5.842621	0.427314	0.000685
ZNF664	76.86367	32.85305	0.42742	0.001106
NPIPB3	1.616673	0.691041	0.427446	0.104822
SRRM1	18.74959	8.01649	0.427555	0.036804
C8orf33	14.55851	6.226161	0.427665	0.001783
ZNF195	8.528194	3.647518	0.427701	0.000614
TELO2	14.11617	6.037505	0.427701	0.001295
NUP107	22.7971	9.751944	0.427771	0.003275
AMER1	1.319536	0.564482	0.427788	0.065324
HASPIN	7.321324	3.1322	0.427819	0.086055
RPS24	791.9701	339.0142	0.428064	0.002115
RPL7	1552.729	664.7418	0.428112	0.005863
PARD6B	1.530826	0.655461	0.428175	0.000612
ATG2B	4.721056	2.022795	0.428462	0.056123
RAB11FIP3	7.854976	3.366332	0.42856	3.05E-05
NPPB	0.108895	0.04667	0.428584	0.172748

IFT27	16.32325	6.998736	0.428759	0.012229
FAM129C	0.035667	0.015294	0.428793	0.351919
MRI1	15.46986	6.634832	0.428888	0.004384
CT62	0.035815	0.015361	0.428898	0.30853
KRAS	11.75014	5.040529	0.428976	2.26E-05
ELAVL3	0.029551	0.012678	0.429016	0.567955
UBXN10	0.005965	0.00256	0.429123	0.627582
GRK1	0.008528	0.00366	0.429123	0.627582
RASL12	0.011087	0.004758	0.429123	0.627582
GSDMA	0.015886	0.006817	0.429123	0.627582
KLHL33	0.00655	0.002811	0.429123	0.627582
UBQLN4	24.46053	10.49918	0.429229	0.000258
ISLR2	0.062517	0.026844	0.429386	0.15415
TBC1D31	6.391438	2.745198	0.429512	0.001234
DNAJC6	4.242202	1.822283	0.429561	0.024224
MAZ	194.7483	83.67002	0.429632	0.000395
P2RX3	0.076007	0.032671	0.429842	0.223575
GCC2	6.51792	2.801881	0.429874	0.007205
GPT	0.041202	0.017714	0.42993	0.422307
MFGE8	29.94114	12.87552	0.430028	0.008767
SLC18B1	14.60755	6.28177	0.430036	0.003794
CIRBP	113.9795	49.02133	0.430089	0.005555
SBF1	32.96314	14.17765	0.430106	0.010422
AHSP	0.129865	0.05586	0.430138	0.504733
ARHGAP5	4.474722	1.924928	0.430178	0.001855
CCT4	290.3967	124.9553	0.430292	0.003952
PIK3C2A	9.258149	3.986063	0.430546	0.012638
HDX	1.30459	0.561704	0.43056	0.007144
PREPL	18.93885	8.15808	0.430759	0.007145
NUBPL	1.511354	0.651113	0.430814	0.067029
PDIA5	42.65367	18.37598	0.430818	0.012932
PRPF8	81.15463	34.97191	0.430929	0.009113
MKRN2	34.27315	14.77023	0.430956	0.001892
TNXB	0.895948	0.386144	0.43099	0.125381
MANEAL	7.274366	3.1355	0.431034	0.009028
LIN54	4.864482	2.097087	0.431102	0.024298
DNAJC18	7.21811	3.112188	0.431164	0.004482
PTPDC1	2.459921	1.060729	0.431205	0.001583
ETNK1	4.602467	1.985277	0.431351	0.003437
CCDC126	2.238367	0.965561	0.431369	0.00437
NAT9	16.5176	7.125352	0.431379	0.003064
GTF3C4	10.52106	4.539887	0.431505	0.00229
INO80D	1.345248	0.580488	0.43151	0.042808
ACAP2	6.556704	2.829959	0.431613	0.005329
SRMS	0.01549	0.006686	0.431623	0.629343
OR13A1	0.0059	0.002547	0.431623	0.629343
AOC1	0.01269	0.005477	0.431623	0.629343
MYOG	0.024271	0.010476	0.431623	0.629343
BIRC7	0.027199	0.01174	0.431623	0.629343
LIN28A	0.009246	0.003991	0.431623	0.629343
RBM39	86.16999	37.19689	0.431669	0.002122
GDAP1	5.039205	2.175377	0.431691	0.010638
ZMYM5	3.950911	1.705889	0.431771	0.017349
BRCA2	2.59858	1.123146	0.432215	0.116089
GJD3	0.113553	0.049088	0.432297	0.294373
POLH	5.326839	2.302791	0.4323	0.004011
ZNF350	1.718098	0.742738	0.432302	0.00256
C19orf54	12.24962	5.296245	0.43236	0.000438

PLA2G12A	13.32991	5.763597	0.432381	0.000285
LRRC37A2	1.0003	0.432651	0.432521	0.006716
PHF21A	9.963823	4.310103	0.432575	0.003008
FMOD	0.057965	0.025075	0.432581	0.140018
LEPR	2.818145	1.219337	0.432673	0.02174
NUP58	14.2404	6.162008	0.432713	0.001097
CBX1	91.60318	39.67249	0.433091	0.000204
KIAA1551	2.816234	1.21998	0.433196	0.018015
FAM160B1	3.41883	1.481518	0.433341	0.007954
MTA3	7.602538	3.294506	0.433343	0.000528
LOC10798	0.005654	0.00245	0.43336	0.44026
LCE1B	0.033461	0.014501	0.43336	0.44026
LOC64517	0.015749	0.006825	0.43336	0.44026
DCX	0.015462	0.006702	0.43346	0.199428
RBMS2	4.404379	1.90919	0.433475	0.001638
UBR4	17.12548	7.423605	0.433483	0.086122
INPP5F	4.28794	1.859918	0.433756	0.006473
CRKL	45.07914	19.5551	0.433795	0.000877
RPL36	1707.024	740.6021	0.433856	0.002723
NPAS3	0.034604	0.015022	0.434106	0.207608
PLVAP	0.032111	0.013941	0.434142	0.440977
MYCT1	0.007268	0.003155	0.434142	0.440977
ENPP6	0.009398	0.00408	0.434142	0.440977
CLDN12	16.08067	6.981837	0.434176	0.005262
ARL6IP6	12.51549	5.433971	0.43418	0.005443
PHLPP1	3.180516	1.381156	0.434255	0.000509
IL4I1	0.255437	0.110937	0.434304	0.033154
PAAF1	21.76275	9.453072	0.434369	0.000183
TTC38	6.474788	2.813781	0.434575	2.50E-05
TTC21B	1.555487	0.676123	0.43467	0.00022
TRIM66	2.501531	1.087357	0.434677	0.021622
ARID5A	4.309181	1.873116	0.43468	0.028738
LOC10798	0.777973	0.338175	0.434688	0.061252
OBSL1	6.999185	3.04265	0.434715	0.016947
PHACTR2	2.02477	0.880244	0.434738	0.004927
STAC3	2.007297	0.872848	0.434837	0.006213
KCNA7	0.025334	0.01102	0.434988	0.477098
TMEM182	1.348169	0.586447	0.434996	0.011946
HSD17B7	10.64751	4.63241	0.43507	2.59E-05
UBR5	15.31082	6.662939	0.435178	0.003489
ANP32E	59.46852	25.88684	0.435303	0.00028
SYCE2	0.814187	0.354458	0.435351	0.049308
RALGPS2	6.073193	2.644054	0.435365	0.000206
UBQLNL	0.064744	0.028207	0.43567	0.032065
KHSRP	102.6843	44.74191	0.435723	0.003347
CCDC142	2.750085	1.198379	0.435761	0.043619
TAS2R50	0.037896	0.016516	0.435828	0.443239
OCSTAMP	0.019101	0.008325	0.435828	0.443239
CXCL12	0.007048	0.003072	0.435828	0.443239
FRG2	0.052968	0.023085	0.435837	0.555285
RPL38	1878.23	818.7588	0.43592	0.000541
SLC25A15	10.02727	4.371163	0.435927	0.029012
C4orf46	14.14206	6.165603	0.435976	0.001133
C2orf50	0.040551	0.01768	0.435997	0.407694
CBR4	8.547012	3.727352	0.4361	0.056905
ARFGAP3	44.15571	19.25914	0.436164	0.009043
GAS2L2	0.037864	0.016515	0.436166	0.226274
SF3B1	71.74259	31.29411	0.4362	5.37E-05

GFI1B	0.024841	0.010837	0.436253	0.456474
GTF3C3	19.07635	8.324119	0.436358	0.008658
GJB1	0.080022	0.034927	0.436467	0.364045
TIPIN	14.00615	6.113779	0.436507	0.037503
TROAP	23.27457	10.15977	0.436518	0.000388
TACSTD2	0.017749	0.007751	0.43669	0.632917
PPP1R3A	0.016858	0.007362	0.43669	0.632917
PIK3R6	0.008328	0.003637	0.43669	0.632917
OR5B12	0.034892	0.015237	0.43669	0.632917
MCEMP1	0.028116	0.012278	0.43669	0.632917
PLPPR1	0.027486	0.012003	0.43669	0.632917
WNT10A	0.02872	0.012542	0.43669	0.632917
AMPD1	0.015279	0.006672	0.43669	0.632917
GCM1	0.045054	0.019676	0.436728	0.197627
LEF1	9.743716	4.264269	0.437643	0.004458
MEF2B	0.103669	0.045372	0.437665	0.286235
NEMF	7.584181	3.319984	0.437751	0.011797
TNFSF4	0.130039	0.056928	0.437774	0.057642
ALPK1	2.752486	1.205357	0.437916	0.031213
LGALS4	0.062519	0.02738	0.437949	0.212521
RCC2	49.27135	21.58095	0.438002	0.000124
GLI3	2.777512	1.217056	0.438182	0.021367
BIRC6	4.467089	1.957544	0.438215	0.016214
MED14	14.593	6.395116	0.438232	0.000184
STXBP4	1.535965	0.673136	0.43825	0.002355
ENTPD1	0.025001	0.01096	0.438399	0.289093
S1PR2	3.405232	1.493518	0.438595	0.012939
IMPG2	0.018093	0.007937	0.43867	0.158114
C11orf1	12.39939	5.444537	0.439097	0.008432
TBC1D24	2.171574	0.953741	0.439193	0.014297
CCDC62	0.282834	0.124219	0.439195	0.126117
TEDC2	15.20199	6.676817	0.439207	0.000932
NYAP2	0.002697	0.001185	0.439307	0.447177
CIDEB	0.019056	0.008371	0.439307	0.447177
SLC23A1	0.012512	0.005497	0.439307	0.447177
SLC9C2	0.008536	0.00375	0.439307	0.447177
BHMT	0.015245	0.006697	0.439307	0.447177
ECHDC3	0.022987	0.010098	0.439307	0.447177
FAM163B	0.022096	0.009707	0.439319	0.152472
ZNF136	2.678994	1.177014	0.439349	0.016637
DGKA	5.708105	2.508155	0.439402	0.001039
FABP3	0.116664	0.051271	0.439479	0.208656
TTC5	9.104912	4.001518	0.43949	0.062865
LTN1	6.769389	2.975686	0.43958	0.01082
CTHRC1	3.513084	1.544461	0.439631	0.064155
DHX33	8.747828	3.846477	0.439706	0.011966
AUNIP	9.686846	4.260163	0.439788	0.022836
NIPSNAP1	105.983	46.61082	0.439795	0.027633
DCUN1D4	8.586292	3.776896	0.439875	9.54E-06
EFR3B	1.993171	0.876861	0.439933	0.003568
WDR24	5.693718	2.505448	0.440037	0.003356
TSEN54	16.10148	7.085458	0.44005	0.001295
IRF2BP2	34.64811	15.25218	0.440202	2.38E-05
ZSWIM9	7.277725	3.20466	0.440338	0.001873
SPN	0.024812	0.010927	0.440405	0.621695
THAP12	19.58553	8.628602	0.44056	0.00083
MED30	16.45773	7.254783	0.440813	0.002404
TRMT13	4.072736	1.796078	0.441	0.014637

TRAPPC9	2.173861	0.958727	0.441025	0.000566
POU5F2	0.057829	0.025508	0.441092	0.326807
GPATCH8	3.994603	1.762459	0.44121	0.005078
SMG1	9.037958	3.989564	0.441423	0.005829
TESPA1	0.013879	0.006128	0.441573	0.462363
KHNYN	11.37559	5.023202	0.441577	0.000602
VTA1	30.13006	13.30584	0.441614	0.003782
KRTAP5-8	0.032034	0.014153	0.441809	0.450203
SKA3	13.21307	5.839707	0.441965	0.00298
ANKRD10	9.62855	4.256751	0.442097	0.046696
SLC2A3	37.0428	16.38642	0.442364	0.021631
ASB16	0.069075	0.030565	0.44249	0.370433
CALR3	0.114359	0.050603	0.44249	0.370433
ANKRD26	0.888706	0.393336	0.442594	0.000417
KCNE2	0.186521	0.082555	0.442605	0.211939
LOC10537	0.096879	0.042879	0.442608	0.543415
ZFYVE16	3.700674	1.63808	0.442644	0.013138
HAX1	230.0158	101.8158	0.442647	0.009309
5-Mar	13.49014	5.971813	0.44268	0.002598
DHX16	17.06125	7.554367	0.442779	0.000965
METTL27	1.548469	0.685639	0.442785	0.052582
KCNRG	0.114112	0.050536	0.442866	0.371886
TGS1	8.622074	3.81854	0.44288	0.003883
ZNF696	2.507294	1.110834	0.443041	0.003443
PTGFR	0.096152	0.042607	0.44312	0.119428
TMEM40	0.044491	0.019725	0.443342	0.21796
TNKS2	9.432642	4.182298	0.443386	0.00235
NPDC1	39.63787	17.57599	0.443414	0.004584
DOK4	8.136646	3.608354	0.443469	0.006369
TNKS	6.991737	3.101475	0.443592	0.002164
ZNF841	1.816049	0.805805	0.443713	0.010306
CRTC3	7.428455	3.296305	0.44374	0.001314
DRAXIN	0.13321	0.059112	0.443753	0.341064
VIL1	0.013589	0.006032	0.443894	0.638006
MPV17L	0.014245	0.006323	0.443894	0.638006
C2orf66	0.277379	0.12314	0.443942	0.077545
ENAH	17.50079	7.771163	0.444046	0.000587
PHF2	7.001339	3.108935	0.444049	0.002729
KDM4D	1.252544	0.556366	0.444189	0.217366
PABPC3	0.471544	0.209474	0.44423	0.05586
RPL22	313.6605	139.3409	0.444241	0.003209
ZNF749	3.069064	1.363484	0.444267	0.026993
TNPO2	29.76901	13.22633	0.444299	0.003386
CCDC113	3.077425	1.367373	0.444324	0.005142
PABPC1	488.775	217.2518	0.444482	0.001288
KREMEN1	7.712772	3.428439	0.444515	0.000468
GJC2	0.559219	0.248659	0.444654	0.037515
LOC10798	0.217342	0.096653	0.444704	0.393796
OTUD7B	6.35262	2.826632	0.444955	0.008127
NPIPA1	6.830739	3.039395	0.444958	0.001999
GARS	225.309	100.2576	0.444978	0.020203
TMED5	17.83514	7.940265	0.445203	0.000159
ZC3H7A	25.69519	11.4433	0.445348	0.003194
SRGAP2	7.82307	3.485644	0.44556	1.06E-05
ATAD2	24.26726	10.81281	0.445572	0.000722
ZSCAN32	3.872172	1.725889	0.445716	0.017726
PARS2	8.504024	3.79094	0.445782	0.016233
RPH3A	0.014033	0.006257	0.44586	0.336656

NCKAP1L	0.123101	0.054886	0.445862	0.164866
CCT3	368.4447	164.3856	0.446161	0.007243
GGA1	18.93045	8.44835	0.446284	0.002848
PTPRU	16.88494	7.537111	0.446381	0.058177
HMGN4	46.92719	20.95209	0.446481	0.000459
LOC10099	0.02915	0.013015	0.446484	0.455371
SMIM11A	0.56846	0.253827	0.446518	0.105796
SMN2	16.2676	7.265453	0.446621	0.044194
TSNAX	28.27642	12.63086	0.446692	0.041261
CLIC2	0.175949	0.078598	0.446707	0.182059
DHFR2	3.044262	1.360734	0.446983	0.127352
STRN4	48.88269	21.86	0.447193	0.003196
ABI2	24.81327	11.09695	0.447218	0.004311
CCDC167	85.21129	38.1197	0.447355	0.011047
ZNF507	4.720059	2.11191	0.447433	0.003284
KLHL12	26.70683	11.94979	0.447443	0.012994
ZKSCAN2	2.142001	0.958609	0.44753	0.049325
SLC5A11	0.081901	0.036666	0.447686	0.444895
MED1	14.8617	6.655424	0.447824	0.068719
PILRB	15.25988	6.836568	0.448009	0.007108
GK	5.43417	2.434778	0.44805	0.007734
CNN3	38.47444	17.24131	0.448124	0.009204
CEP85	10.40853	4.664316	0.448125	0.00481
SCNM1	0.314089	0.14083	0.448376	0.140301
ALKBH8	4.012506	1.799198	0.448398	0.071458
PPM1M	14.53028	6.51787	0.448572	0.022068
HIST1H2B	2.531201	1.136219	0.448885	0.0402
POLK	3.206012	1.439453	0.448986	9.59E-05
ANKZF1	21.13091	9.487817	0.449002	0.008924
ZNF638	17.73503	7.96414	0.449063	0.002714
PIGH	29.14192	13.08774	0.449103	0.000417
RLN3	0.030444	0.013672	0.449105	0.641693
CAPN11	0.025664	0.011526	0.449105	0.641693
CTSE	0.016396	0.007363	0.449105	0.641693
GPR37L1	0.0054	0.002425	0.449105	0.641693
DPT	0.021282	0.009558	0.449105	0.641693
C17orf51	6.328724	2.842454	0.449135	0.012167
ZNF142	3.842082	1.725978	0.44923	0.003552
ST3GAL6	4.212609	1.892865	0.449333	0.011179
C2orf49	4.194187	1.884974	0.449425	0.00146
ZNF623	5.290229	2.37847	0.449597	0.132123
FCHO2	2.572577	1.156755	0.449648	0.008447
ZNF594	0.869959	0.391204	0.44968	0.260826
KDELR1	230.6416	103.738	0.44978	0.002366
NEO1	12.99685	5.845767	0.449783	0.009556
STX19	0.019655	0.008845	0.449985	0.642317
ARSH	0.044059	0.019826	0.449985	0.642317
HIST1H4A	0.100021	0.045008	0.449985	0.642317
FAM189B	30.04884	13.52906	0.450236	0.048511
GAREM1	2.366661	1.065649	0.450275	0.029447
RABEP1	15.37677	6.924424	0.450317	0.006773
ASH1L	4.902419	2.207737	0.450336	0.04889
PITX1	11.74658	5.290327	0.450372	0.012408
BRD3	10.30568	4.641785	0.45041	0.004661
UBE4A	11.98924	5.401209	0.450505	0.025249
HES6	13.61497	6.135615	0.450652	0.051859
TOR2A	7.960889	3.58825	0.450735	0.000589
COL1A1	24.60954	11.10668	0.451316	0.032533

AHCTF1	8.94613	4.041874	0.451801	2.29E-05
CDHR4	0.134426	0.060742	0.451864	0.203284
POLR3E	11.01647	4.980358	0.452083	0.001143
RPL34	455.3706	205.8759	0.452106	0.001454
ZNF652	3.232928	1.461898	0.45219	0.00085
ZNF774	1.327629	0.600581	0.452371	0.050626
TP53INP1	3.724197	1.685024	0.452453	0.044283
HPN	0.02708	0.012257	0.452611	0.46255
FLJ20712	0.013326	0.006031	0.452611	0.46255
UGT3A1	0.006579	0.002978	0.452611	0.46255
CKAP5	84.83522	38.40269	0.452674	0.017026
TTC17	16.08278	7.280692	0.452701	0.002088
KIDINS22(	5.372921	2.433965	0.453006	0.018499
ABHD14B	30.1375	13.653	0.453024	0.015307
THAP5	11.10057	5.028992	0.453039	0.025021
R3HDM2	16.50763	7.479394	0.453087	0.005686
PRC1	154.2398	69.90653	0.453233	0.000128
NTHL1	18.25844	8.27756	0.453355	0.016158
BARD1	5.114327	2.31891	0.453414	0.00047
NOL11	45.28073	20.54654	0.453759	0.008821
ZNF19	1.724285	0.782436	0.453774	0.106882
NBPF12	2.116761	0.960938	0.453966	0.024619
NET1	8.980315	4.077693	0.45407	4.65E-05
MRPL10	49.10134	22.30559	0.454277	0.002815
NCOA1	4.641651	2.109333	0.454436	0.0259
WDR33	8.653789	3.933861	0.454582	0.000488
NUBP2	20.24157	9.203847	0.4547	2.21E-05
NDUFAF5	6.625383	3.012567	0.454701	0.004813
DZIP1	10.1004	4.59449	0.454882	0.003164
SH3GL2	1.253353	0.570151	0.4549	0.008228
WAPL	11.4257	5.197919	0.454932	0.000291
STX17	6.34664	2.887858	0.455022	0.009474
ANKRA2	9.91455	4.512789	0.455168	0.00377
PEX26	15.93447	7.253989	0.455239	0.001417
CABP5	0.016971	0.007726	0.455267	0.646059
DCDC2B	0.016374	0.007455	0.455267	0.646059
KDM4C	1.925056	0.876489	0.455306	0.00079
TTLL1	9.696407	4.41488	0.455311	0.005297
NEURL2	0.371046	0.169125	0.455806	0.411169
ZBTB8A	2.770004	1.26275	0.455866	0.006506
FCGR2A	0.822477	0.375005	0.455946	0.021073
PRH1-TAS	0.230865	0.105265	0.45596	0.081126
CRLF3	9.312374	4.247429	0.456106	3.67E-05
MCM3	167.8766	76.57494	0.456138	8.05E-05
SIDT1	0.147378	0.067249	0.456302	0.354562
NAP1L1	152.919	69.78612	0.45636	1.88E-05
ZNF486	0.102981	0.047027	0.456651	0.399081
STRN	4.348584	1.985899	0.456677	0.003464
RIOK3	19.60655	8.955701	0.456771	0.010999
ATF7IP	5.804203	2.65123	0.456778	0.001519
TMEM65	10.47459	4.787013	0.457012	2.44E-05
SPECC1L	16.62053	7.5978	0.457133	0.008275
8-Sep	22.40723	10.24543	0.457238	0.001414
CEP120	7.447817	3.407359	0.457498	0.039679
COA6	29.2927	13.40701	0.457691	0.06706
CNOT6L	4.047453	1.852809	0.457772	5.11E-05
SNRPD1	127.614	58.42408	0.457819	0.012572
SYT1	3.720938	1.70361	0.457844	0.001121

ZSCAN16	1.79515	0.822355	0.458098	0.001757
TNFSF10	0.200787	0.092006	0.458227	0.176248
TUBD1	5.705621	2.615217	0.458358	0.011304
MAU2	12.37793	5.673794	0.45838	0.01316
PCCB	50.9838	23.37216	0.458423	0.013166
MRPS14	26.60986	12.19881	0.458432	0.002313
WEE1	19.63807	9.002809	0.458436	0.001066
PYGO2	24.86212	11.40025	0.458539	3.97E-05
GCA	1.69015	0.775373	0.45876	0.007881
ING4	26.1114	11.97983	0.458797	0.005687
ZNF282	14.00131	6.424663	0.458861	0.006942
TRMT2A	21.59749	9.91064	0.458879	0.004735
CCDC57	1.858787	0.853133	0.458973	0.0295
CLASRP	26.96347	12.38076	0.459168	0.004426
RPF1	43.65014	20.04575	0.459237	0.01489
AKAP6	0.241739	0.111043	0.45935	0.104896
ARIH2	27.60905	12.68373	0.459405	0.000114
CCDC134	4.6166	2.121049	0.45944	0.009049
MINDY1	2.886333	1.326139	0.459455	0.018874
LOC10099	0.244999	0.112568	0.459463	0.175517
DUSP16	6.399853	2.941057	0.459551	0.029036
ZNF367	10.30198	4.73933	0.460041	0.05055
SYNJ1	2.309878	1.062787	0.460105	0.021507
CCDC74A	9.215993	4.241379	0.460219	0.000879
CTNNBIP1	6.553754	3.016383	0.460253	0.001164
GTPBP10	5.522603	2.54199	0.460288	0.031861
CAB39L	1.83167	0.843982	0.460772	0.015301
LINS1	3.253114	1.499148	0.460835	0.001358
RNF152	0.83337	0.384052	0.460841	0.027863
TIMM44	42.01139	19.3727	0.46113	1.28E-05
YTHDC1	18.62269	8.591494	0.461346	0.003675
REV3L	2.967308	1.369647	0.461579	0.003032
PDPR	6.331089	2.923697	0.4618	0.001947
EIF3D	371.7732	171.7176	0.461888	5.22E-05
MDH1B	1.121195	0.517877	0.461897	0.178449
SLK	12.14281	5.609121	0.46193	0.00043
SFSWAP	13.81278	6.382663	0.462084	3.33E-05
DRAM2	51.0255	23.5856	0.462232	1.94E-05
ADSSL1	5.944647	2.748169	0.462293	0.024963
HDAC10	11.27267	5.214685	0.462595	0.024589
GINS2	81.13159	37.55923	0.462942	0.009806
LCORL	2.399044	1.110876	0.46305	0.005578
CRTC1	3.152334	1.459728	0.463063	0.010785
WDFY1	18.59614	8.611957	0.463105	0.010887
TTC21A	1.456705	0.67497	0.463354	0.002578
CNIH4	63.56349	29.45591	0.463409	0.00395
FBXO22	24.90509	11.54253	0.463461	0.00271
INTS12	20.41208	9.464524	0.463673	0.014696
OR1F1	0.332965	0.154477	0.463943	0.021494
CDK15	0.09529	0.044224	0.464098	0.014022
PIAS1	8.791514	4.081476	0.464252	7.84E-05
OFD1	4.796102	2.227012	0.464338	0.00338
PPIG	18.63843	8.659755	0.464618	0.000331
NSD3	10.82623	5.030255	0.464636	0.002641
AURKB	63.59377	29.55554	0.464755	0.002441
EIF3L	318.2309	148.06	0.46526	0.001552
MAGEA6	2.064697	0.960824	0.465358	0.066066
APOBEC3I	1.095158	0.509958	0.465648	0.005046

PPIL1	39.50297	18.39593	0.465685	0.04982
ACOT13	11.7833	5.487958	0.46574	0.050769
LRCH4	12.5928	5.865908	0.465814	0.036978
ZNF71	2.403609	1.120256	0.466073	9.63E-05
PTP4A1	38.07865	17.75973	0.466396	1.90E-05
ZNF713	0.442933	0.206588	0.466409	0.002841
WDR43	24.79016	11.56396	0.466474	0.011637
GRK6	26.40933	12.32001	0.466502	0.002166
ENTPD5	3.642386	1.699334	0.466544	0.001403
CDC14A	1.448706	0.676039	0.46665	0.001883
RFWD3	23.41203	10.92793	0.466766	0.017362
MORC4	20.4143	9.529039	0.466782	0.006139
ATXN2	13.7064	6.398799	0.466848	0.000264
SMG5	43.46788	20.29706	0.466944	0.000377
DOCK11	5.510654	2.574194	0.46713	0.005181
HMGN1	92.25144	43.1009	0.467211	3.27E-05
PBX4	2.026628	0.946919	0.467239	0.001956
NPM1	1530.363	715.1241	0.46729	0.004124
NGDN	26.67338	12.4715	0.467564	0.000379
ZNF12	7.241467	3.385852	0.467564	0.003519
PGM3	17.23461	8.058709	0.467589	0.028375
TNFSF9	8.744503	4.090775	0.467811	0.010886
LDHB	671.3591	314.0787	0.467825	0.011833
METTL14	10.92005	5.108699	0.467827	1.22E-05
SNRPA1	147.5557	69.03954	0.467888	0.011623
GLB1L3	0.226759	0.106127	0.468016	0.257127
CLSPN	9.746624	4.563412	0.468204	0.011669
ANKRD31	0.133501	0.062514	0.46827	0.359769
LMNB1	45.23976	21.19535	0.468511	0.009972
VRK2	3.076376	1.441377	0.468531	0.065846
YTHDC2	8.157205	3.822212	0.468569	0.044365
SEZ6L	0.031278	0.01466	0.468696	0.36654
TCEA3	0.759959	0.356239	0.46876	0.02921
WDR59	12.77046	5.988851	0.468961	0.013113
TIMM17B	97.00698	45.49567	0.468994	1.05E-05
CDC14B	5.600635	2.627453	0.469135	0.001655
C19orf44	3.903105	1.831388	0.469213	0.00603
CUL4B	38.51395	18.07269	0.469251	0.012479
NR2C2	7.463005	3.502811	0.469357	0.017418
CAPRIN2	7.846693	3.68317	0.469391	0.031318
TMEM161	26.65588	12.52063	0.469713	0.002604
TRAPPC1(	2.980475	1.400481	0.469885	0.038377
NBPF20	0.909486	0.427403	0.469939	0.027873
FERMT2	29.13077	13.6964	0.47017	0.011138
NDST2	5.095257	2.395878	0.470217	0.00178
JMJD1C	4.107808	1.932413	0.470424	0.040766
NDUFS2	71.46263	33.62106	0.470471	4.35E-05
FARP1	9.832554	4.626621	0.470541	0.002282
SAMD8	5.045508	2.374573	0.470631	0.006307
MRPL30	31.09864	14.64118	0.470798	0.003212
PDIK1L	2.184932	1.028982	0.470945	0.031085
ORC2	13.1601	6.197811	0.470955	0.017242
LOC10798	1.610906	0.758664	0.470955	0.041091
KCTD7	4.024828	1.896156	0.471115	0.024729
MANSC1	4.449806	2.097939	0.471467	0.050055
POLE2	13.62648	6.426094	0.471589	0.038014
ZNF205	9.496656	4.478861	0.471625	0.000884
SCNN1D	2.474138	1.167051	0.4717	0.033611

NUB1	58.63571	27.66042	0.471733	0.001755
ZW10	15.90242	7.502294	0.471771	0.024694
RPL24	2741.554	1294.358	0.472126	0.000212
ROBO1	26.10526	12.32758	0.472226	0.001258
EIF4ENIF1	8.358212	3.947345	0.472271	0.007099
BACH1	5.724455	2.703816	0.472327	0.01127
CS	114.3968	54.04131	0.472402	4.36E-06
HAGH	37.43962	17.68698	0.472413	0.000654
DDX17	125.8387	59.45289	0.472453	0.013933
NUAK1	2.704014	1.277553	0.472465	0.008353
RAB3GAP1	22.73497	10.74544	0.472639	0.000948
FBXW7	2.714281	1.28298	0.472678	0.008611
NDFIP2	18.75996	8.868036	0.472711	0.000211
SH3PXD2A	5.566982	2.632321	0.472845	0.006368
TJAP1	13.14952	6.219468	0.472981	0.016309
ZNF764	5.465223	2.585857	0.473148	0.027384
AAMP	137.3314	64.9853	0.473201	0.001537
RPAIN	24.11284	11.41095	0.473231	1.73E-05
MCM2	93.03794	44.03043	0.473252	0.00341
CCP110	8.610697	4.076129	0.47338	0.001296
KATNA1	19.18045	9.081494	0.473477	0.009241
SUN1	28.1468	13.33243	0.473675	0.00067
TPRG1	0.043918	0.020811	0.473862	0.265604
SMNDC1	29.90557	14.18277	0.474252	0.000884
NAA15	24.86595	11.79426	0.474314	0.005199
ZNF689	7.346709	3.484892	0.474347	0.034813
SOX13	5.552472	2.635234	0.474606	0.001778
TSGA10	0.902088	0.428183	0.474658	0.087311
DRG1	132.3919	62.84409	0.474682	0.002352
DUS1L	38.76665	18.40544	0.474775	0.000981
EZR	52.6514	24.99893	0.474801	0.018079
GK5	2.892287	1.373998	0.475056	0.000575
UBE2V1	1.044624	0.496375	0.475171	0.008339
HACD3	74.04595	35.19261	0.475281	0.000314
TAF2	8.879399	4.223818	0.475687	0.024459
GFM2	18.26213	8.688042	0.475741	0.008408
MIS18A	21.36422	10.16386	0.475742	0.014989
C16orf91	20.22927	9.624813	0.475786	0.00442
NTPCR	59.00269	28.07817	0.475879	0.005019
COIL	15.30952	7.291924	0.4763	0.011336
ORC6	52.65304	25.08078	0.476341	0.00207
MAPRE3	18.32107	8.727332	0.476355	0.011089
ZXDB	2.29495	1.093396	0.476436	0.025917
SLC1A4	11.45177	5.456763	0.4765	0.056726
TMEM80	10.73407	5.115369	0.476555	0.007846
MDM2	13.44624	6.411026	0.476789	0.033602
FOXP4	6.44045	3.07178	0.476951	0.00378
DDIT3	39.66077	18.91695	0.476969	0.106982
DSTYK	5.526344	2.635986	0.476986	0.000253
TAF1	6.432774	3.068824	0.477061	0.000613
ROBO3	4.626479	2.207536	0.477152	1.74E-05
DVL2	29.48604	14.07286	0.477272	2.92E-05
TDRKH	7.741531	3.696448	0.477483	0.001617
SMARCD2	41.05865	19.60516	0.477491	0.00082
RPL21	2358.189	1126.063	0.477512	0.004928
NKAP	45.42725	21.70242	0.47774	0.043177
WNK1	16.72954	7.994418	0.477862	0.005619
C6orf106	28.99098	13.85921	0.478052	0.000545

KDM5C	38.4078	18.3628	0.478101	0.003311
ROCK1	15.22507	7.279321	0.478114	5.35E-05
FASTKD5	8.079896	3.863333	0.478141	0.014738
GPHN	5.463372	2.612286	0.478145	0.022346
C15orf61	11.94702	5.713739	0.478256	0.000338
TRAF6	2.728329	1.305093	0.478349	0.018788
NPIPB5	4.672632	2.235307	0.478383	0.049499
CHTOP	85.86541	41.08943	0.478533	0.002282
RAB29	14.80735	7.086201	0.47856	0.012542
MAP2	0.917215	0.439022	0.478647	0.118026
ANKRD17	10.24132	4.902047	0.478654	0.021513
ZNF354A	7.942276	3.802388	0.478753	0.036762
TNFRSF19	3.972839	1.902565	0.478893	0.05479
MYCBP2	8.195475	3.925425	0.478975	0.005552
SERINC4	0.162386	0.077821	0.479233	0.046971
ENPP4	3.55051	1.701763	0.479301	0.000631
PMPCB	25.32734	12.14214	0.479408	0.001373
BTG1	24.43987	11.7182	0.47947	0.000232
KIFC1	33.64387	16.13449	0.479567	0.004625
ZSWIM6	4.517779	2.167344	0.479737	0.008011
CNTD2	0.184111	0.088351	0.479879	0.330675
SMCO2	0.112159	0.053832	0.479961	0.070857
ARRB1	2.027941	0.973394	0.479991	0.112796
PRDM15	0.986669	0.47361	0.480009	6.74E-05
SMYD3	3.572353	1.714887	0.480044	0.043484
UBALD1	18.22508	8.749539	0.480082	0.016211
STARD10	11.19869	5.37727	0.480169	0.001418
ORC4	10.06524	4.833373	0.480204	0.009721
AKAP13	4.010177	1.925837	0.480237	0.007052
LEAP2	1.257144	0.603815	0.480307	0.065173
BYSL	21.90515	10.5233	0.480403	0.033131
MAP6D1	2.851209	1.369898	0.480462	0.047643
FAM168A	11.96231	5.749758	0.480656	0.005003
MMS19	18.04812	8.676494	0.480742	0.004428
KLF5	7.152473	3.438701	0.480771	0.000477
TMEM169	0.715754	0.344146	0.480816	0.000512
PAK1	15.88253	7.637983	0.480905	0.003101
SSH2	1.90587	0.916809	0.481045	0.00023
NPRL2	29.95892	14.41905	0.481294	0.002825
RNLS	0.01123	0.005406	0.481425	0.418137
XYLB	1.423453	0.685808	0.481792	0.061573
RNF217	2.153252	1.037742	0.481942	0.009374
SAXO2	0.830835	0.400449	0.481984	0.124907
TRIM52	4.65092	2.241907	0.482035	0.050779
ZDHHC23	2.707397	1.305221	0.482094	0.006017
MED24	35.04952	16.89921	0.482152	0.005662
ASNSD1	36.8839	17.7876	0.482259	0.068276
KRR1	17.07452	8.237868	0.482465	0.034226
ACAD10	9.600324	4.635161	0.482813	0.03079
VWCE	1.213147	0.585947	0.482998	0.179715
HOMEZ	2.973851	1.436563	0.483065	0.165678
USP54	1.943804	0.938999	0.483073	0.000381
PGAM2	0.197232	0.095292	0.483145	0.409798
CDK5R1	2.273087	1.098324	0.483186	0.003201
CHN1	9.385196	4.537095	0.483431	4.16E-05
MFAP3L	1.479332	0.715175	0.483445	0.01578
RPL27	1764.605	853.4744	0.483663	0.00123
PUSL1	30.79393	14.8942	0.483673	0.015998

PTPN4	1.521972	0.736194	0.483711	0.012669
ABHD3	5.354995	2.590709	0.483793	0.00292
PRSS35	1.215153	0.587884	0.483794	0.311126
RNF8	8.018528	3.879319	0.483794	0.000277
SRP54	50.01973	24.2048	0.483905	0.002824
ZNF684	2.069711	1.001814	0.484036	0.001152
MRPS21	92.25687	44.70933	0.484618	0.00218
PRKAB1	22.94513	11.12033	0.484649	0.000191
ZNF615	2.094373	1.01508	0.48467	0.077675
EPS8	6.313959	3.060243	0.484679	0.0063
UPF1	30.23172	14.65349	0.484706	0.0064
PRMT5	59.37884	28.79228	0.484891	0.035656
TTC23	10.11405	4.905074	0.484976	0.006822
C21orf91	2.441943	1.184516	0.485071	0.038938
SELENOI	8.721793	4.23141	0.485154	0.013897
FAN1	6.37953	3.095247	0.485184	0.013683
ARHGAP3	2.282049	1.107243	0.485197	0.00898
D2HGDH	3.352303	1.626667	0.485238	0.084545
RABGGTB	117.6673	57.10118	0.485276	0.024052
GFER	15.52972	7.53666	0.485306	0.029041
MRPS18B	54.18163	26.30333	0.485466	0.025935
ARHGAP1	0.045409	0.02209	0.486468	0.130663
NME4	72.88475	35.47537	0.486732	0.011656
FAM57B	0.777165	0.378299	0.486768	0.001602
EAF2	1.70759	0.831543	0.486969	0.15563
SARS2	13.90748	6.772906	0.486997	0.032865
ABRAXAS1	8.340644	4.06237	0.487057	0.020402
TONSL	11.9867	5.838287	0.487064	0.039821
NAIP	0.170119	0.082863	0.487088	0.083341
TRPV3	0.160316	0.078089	0.487093	0.000678
DTD2	5.544037	2.70077	0.487149	0.072864
FAM193A	6.91715	3.370295	0.487238	0.007126
SNAPC4	4.294003	2.092629	0.487338	9.50E-05
TMEM183	30.93062	15.07477	0.487374	0.000713
RPL14	1379.981	673.075	0.487742	0.010391
SPPL2B	7.326158	3.574272	0.487878	0.002128
TSPAN6	19.25429	9.397318	0.488064	0.038279
SPTBN4	0.622992	0.304071	0.488081	0.055639
DNAAF3	7.199221	3.513946	0.488101	0.019093
CGB1	0.944992	0.461353	0.488208	0.438067
GMFG	0.658704	0.321647	0.488303	0.149135
CCHCR1	17.064	8.332441	0.488305	0.009407
C4A	0.124407	0.06075	0.488317	0.156547
KLF10	46.11773	22.52756	0.488479	0.015859
SEC61A2	7.312026	3.571928	0.488501	0.00698
PPP1R3F	1.058723	0.517244	0.488555	0.013457
PCBP2	264.4752	129.2482	0.488697	0.001086
DGCR8	18.37932	8.98225	0.488715	0.0227
PHKA2	7.150793	3.49556	0.488835	0.002923
SCN11A	0.051144	0.025003	0.48887	0.272714
ZNF286A	4.289591	2.098997	0.489323	0.00395
ZIC2	12.76595	6.246745	0.489329	0.025365
SELENOO	20.13755	9.855775	0.489423	0.000846
MDH2	180.4938	88.34465	0.489461	0.004928
RMND5B	12.65774	6.195862	0.489492	0.001301
CCDC191	1.723061	0.843435	0.489498	0.03006
MSANTD2	0.701098	0.343204	0.489524	0.019076
PUS7	8.551216	4.187369	0.489681	0.036164

MEF2A	5.98634	2.93165	0.489723	0.012106
ADAM17	12.45059	6.099038	0.48986	0.000516
SCMH1	12.25833	6.00613	0.489963	0.054318
FAM229B	17.76875	8.711973	0.490298	0.00386
ZFP1	4.113179	2.016745	0.490313	0.000513
DPH5	5.019436	2.461745	0.490443	0.032674
ANKRD50	2.88146	1.413302	0.490481	0.000761
FAM168B	41.44719	20.33454	0.490613	0.001576
RNF187	75.22959	36.92147	0.490784	0.000305
KANSL1	3.74574	1.838482	0.490819	0.000446
ME3	0.285539	0.140211	0.491039	0.254435
RBAK	3.719513	1.827303	0.491275	0.000965
METTL8	2.501849	1.229379	0.491388	0.060434
OSR2	2.082478	1.023314	0.491393	0.033565
SEC24B	10.73586	5.276853	0.491516	0.002859
PPM1B	4.861358	2.39054	0.491743	0.001859
RDX	16.42712	8.081839	0.491982	0.004645
ULK1	20.87545	10.27296	0.492107	0.105026
RICTOR	3.70068	1.821502	0.492207	0.06407
KDM4A	20.4856	10.08335	0.492217	0.003843
ARCN1	80.21297	39.48671	0.492273	0.000849
CFAP298	3.639885	1.791863	0.492286	0.002941
NOP58	86.19916	42.44173	0.492368	0.06174
LARP4B	7.060655	3.476652	0.492398	0.008104
WDR11	16.65662	8.204184	0.492548	0.000997
KSR2	0.094177	0.046392	0.492605	0.096697
ALDH18A1	51.12376	25.19285	0.492782	0.000342
PLEKHH3	12.25898	6.041105	0.49279	0.022025
NCOA2	1.188766	0.585849	0.492821	0.021998
CELSR1	4.196492	2.069493	0.493148	0.020045
ANG	2.227239	1.098409	0.493171	0.071088
RAD54B	5.025779	2.479667	0.493389	0.001446
LIN52	4.85789	2.397197	0.493465	0.003108
HSPA12A	2.478554	1.223118	0.493481	0.00022
C14orf93	2.450889	1.209566	0.493521	0.062293
MORF4L2	506.2304	249.8365	0.493523	0.002317
RCHY1	5.843375	2.884991	0.49372	0.030833
MROH6	0.675176	0.333406	0.493806	0.003206
SGSM2	7.439535	3.676564	0.494193	0.006695
XRN2	34.77731	17.18821	0.494236	0.002602
ADPRM	11.08452	5.480469	0.494425	0.009035
ADCK1	4.301114	2.127542	0.494649	0.145294
C6orf52	0.849956	0.420506	0.494738	0.387098
RNF41	13.91329	6.887692	0.495044	0.001419
ATXN7	4.597414	2.276263	0.495118	0.019256
NRGN	33.41313	16.54719	0.49523	0.003572
DCUN1D5	6.140399	3.04099	0.495243	0.048131
RPS7	1981.725	981.8124	0.495433	0.000734
ASAP3	3.007224	1.490406	0.495609	0.003172
LOC10798	0.127205	0.063045	0.495618	0.20729
SLC33A1	10.24295	5.079328	0.495885	0.001467
RAB15	4.597038	2.27969	0.495904	0.000581
MAPRE2	20.26631	10.0508	0.495936	0.003215
NDUFA4L2	1.848505	0.916775	0.495955	0.144304
PRPF40A	21.18012	10.50578	0.496021	0.00067
ATP1B1	29.24175	14.5048	0.496031	0.017704
DNAJC4	20.4164	10.12905	0.496123	0.001714
H3F3A	231.0503	114.631	0.49613	8.80E-05

RPS17	3822.537	1896.701	0.496189	0.00189
PPP2R5D	29.44595	14.61403	0.4963	0.000764
PGGT1B	3.075174	1.526748	0.496475	0.000205
MAP3K2	4.852039	2.409249	0.496544	0.009096
FBXW4	15.18935	7.546627	0.496837	0.034886
AGK	7.122342	3.540286	0.497068	0.00786
ATP6V0A2	6.441749	3.203074	0.497237	0.004817
BOD1	63.65623	31.66286	0.497404	0.001435
GOT1	60.82913	30.27324	0.497677	0.014868
FMR1	16.20178	8.063401	0.497686	0.004712
ARL13B	7.277796	3.622279	0.497716	0.030213
PTPRO	0.007251	0.003609	0.497737	0.191746
DND1	0.863491	0.429879	0.497838	0.198096
MTURN	10.51258	5.233855	0.497866	0.03833
LETM2	0.8857	0.440968	0.497875	0.063465
TIMM8A	12.51407	6.233556	0.498124	0.09452
SESTD1	4.936551	2.45994	0.498312	0.000833
RANBP2	11.15308	5.559223	0.498447	0.003263
APOOL	7.439869	3.708927	0.49852	0.00633
POLR1E	26.00065	12.9646	0.498626	0.016234
LIPT1	2.874011	1.433246	0.498692	0.059295
PRDM4	11.00819	5.492005	0.498902	0.004929
SERHL2	0.898254	0.448148	0.498911	0.221694
BAG6	57.09729	28.48929	0.49896	0.008551
DZIP1L	0.999714	0.498869	0.499012	0.004051
NDUFB8	279.4014	139.4756	0.499194	0.00542
MAMDC2	4.159926	2.076709	0.499218	0.001546
TERF1	13.37555	6.678482	0.499305	0.005696
PWWP2A	3.097052	1.546534	0.499357	0.000149
AHCY	120.3893	60.128	0.499446	0.004715
LSM12	15.9958	7.989831	0.499495	0.003015
NOM1	12.57384	6.28187	0.499598	0.004062
PDGFA	4.2321	2.114525	0.49964	0.041893
CRY2	7.403009	3.700751	0.499898	0.02029
KIAA0513	1.692209	0.846118	0.500008	0.040567
PHACTR1	0.697089	0.348557	0.500018	0.070135
SIKE1	18.18338	9.097022	0.500293	8.75E-05
ATG4A	13.82063	6.914707	0.500318	0.000486
RFPL4B	0.120127	0.060127	0.500527	0.233049
MTCL1	2.639645	1.321472	0.500625	0.014227
PISD	12.51321	6.264613	0.50064	0.006084
UTP14C	4.561783	2.284519	0.500795	0.026322
PCGF3	7.968765	3.991498	0.500893	0.009212
HERC5	7.925002	3.971881	0.501184	0.050873
TOMM6	193.1888	96.86	0.501375	0.002138
B4GALT6	1.139415	0.571341	0.501433	0.042474
ZNF497	0.708295	0.355354	0.501702	0.120962
HERPUD1	74.82559	37.54167	0.501722	0.006485
NAA60	6.542206	3.283835	0.501946	0.001114
PYGB	49.70752	24.95897	0.502117	0.087502
SFXN4	8.486734	4.262119	0.502209	0.172335
ALG9	6.894269	3.462599	0.502243	0.002632
MIER3	5.436712	2.730877	0.502303	0.007567
UNK	5.014547	2.519018	0.502342	0.012493
GCH1	7.484735	3.761656	0.502577	0.003665
RPL23A	2181.445	1096.373	0.50259	0.00767
CEP162	2.175645	1.093575	0.502644	0.029626
MPI	42.06423	21.14913	0.502782	0.018594

FOXO3	4.597265	2.311778	0.502859	0.010353
ZNF791	8.620202	4.335111	0.502901	0.021731
ADCY6	9.917946	4.989569	0.503085	0.012555
PLEKHA5	2.426304	1.220673	0.5031	0.010973
DLX2	12.62975	6.357241	0.503355	0.302438
DZANK1	0.800525	0.403019	0.503443	0.025466
SEC23B	29.199	14.70237	0.503523	0.000373
SUCLA2	29.3979	14.80491	0.503604	0.006369
DCAF7	25.63844	12.91294	0.503656	0.002236
MOSPD2	10.71576	5.397743	0.50372	0.002629
CDK1	70.90064	35.71772	0.503772	0.000472
GATC	21.37097	10.7685	0.503884	0.000603
PDXP	50.02509	25.20939	0.503935	0.001828
PTPRB	0.072333	0.036451	0.503938	0.199983
CLTCL1	4.507179	2.272124	0.504112	0.002553
RNFT1	8.630483	4.350862	0.504127	0.00898
MARS2	3.209327	1.618179	0.504211	0.099787
NR4A2	1.061918	0.535447	0.504226	0.053899
PPIL6	0.585981	0.295522	0.50432	0.058761
USP19	22.32435	11.25952	0.50436	0.008451
PRMT9	3.01001	1.518204	0.504385	0.024156
TIMM9	53.28533	26.88018	0.504457	0.004207
RANBP9	21.99658	11.09989	0.504619	0.016857
TP53	18.48247	9.32699	0.50464	0.041275
SYNGR2	21.81019	11.008	0.504718	0.002547
AP3M2	12.13056	6.122663	0.504731	0.000518
NSUN3	2.469902	1.246677	0.504748	0.006335
AGO3	1.29673	0.654579	0.504792	0.011031
KAT7	22.56693	11.39224	0.50482	0.000781
TRIM2	4.292485	2.167884	0.505042	0.003486
KMT5A	39.85587	20.13164	0.505111	0.003338
CPNE8	2.76347	1.396264	0.505257	0.002786
ZNF592	8.390416	4.239862	0.505322	0.000494
RNASEL	2.762881	1.396533	0.505463	0.004502
TEX11	0.023143	0.0117	0.505547	0.539555
SRBD1	6.485817	3.27899	0.505563	0.058125
KIF2A	17.49362	8.845153	0.505622	0.001086
SYMPK	31.65092	16.00573	0.505696	1.62E-05
C17orf100	2.582044	1.305769	0.505711	0.176397
GAPVD1	7.744642	3.916693	0.505729	0.008594
RECQL4	15.28877	7.732768	0.505781	0.023874
PRRT1	2.320556	1.1739	0.50587	0.012178
TMEM234	5.212487	2.637291	0.505956	0.000991
DUSP12	31.577	15.98339	0.506172	0.001138
SLC16A5	0.028553	0.014458	0.506345	0.495951
VAX2	0.629057	0.318663	0.506572	0.087158
SERF1B	0.894538	0.453258	0.506695	0.192661
CD46	37.54052	19.02348	0.506745	0.000587
CHST5	0.180751	0.09161	0.506832	0.135533
BCL9	6.134859	3.110062	0.506949	0.004292
CEMIP2	4.001441	2.028881	0.507038	0.042736
RPL10	826.7669	419.2696	0.507119	0.001921
COL9A2	1.203175	0.610163	0.507127	0.042602
NIPBL	6.684598	3.390289	0.507179	0.00148
NKX6-1	7.562916	3.836098	0.507225	0.011479
DPAGT1	24.30778	12.33495	0.507449	0.016063
PHTF2	7.14833	3.627685	0.507487	0.000492
PSPH	14.1753	7.195804	0.50763	0.116593

ZNF213	3.664511	1.860956	0.507832	0.02615
IFT46	7.638729	3.879288	0.507845	0.079883
PPP2R5A	11.99413	6.095516	0.508208	0.014763
HOOK2	7.971499	4.052941	0.508429	0.00504
ZNF579	8.255687	4.198479	0.508556	0.019099
EIF3H	295.5545	150.3072	0.50856	0.000244
EPS15L1	9.551725	4.858273	0.508628	1.78E-05
MAPK14	9.645704	4.907933	0.508821	0.000317
SLC35A1	16.95995	8.630324	0.508865	3.53E-05
PRCC	48.43935	24.66021	0.509095	0.000872
RPSA	2909.758	1481.353	0.509098	0.000708
PLK4	13.46334	6.854717	0.509139	0.000778
HSF2BP	0.069084	0.035185	0.509307	0.12845
NCOR1	10.62266	5.411635	0.509443	0.007949
C12orf66	2.347909	1.19617	0.509462	0.032338
NUP50	21.36513	10.8858	0.509512	0.012552
MLLT6	15.78695	8.052779	0.510091	0.012256
GIGYF1	7.991713	4.077577	0.510226	0.004464
ZNF655	3.736808	1.906881	0.510297	0.065173
POU5F1	0.027663	0.014117	0.510301	0.662896
CTU1	11.32947	5.782433	0.510389	0.00102
CCDC163	5.820596	2.971281	0.510477	0.021517
ESYT3	0.027623	0.014103	0.51057	0.106142
LHPP	2.790603	1.425675	0.510884	0.008364
E2F6	5.485286	2.802936	0.510992	0.027752
TOP3A	9.023243	4.613209	0.511258	0.000526
CD70	6.158626	3.148762	0.511277	0.057325
RPL30	1968.114	1006.291	0.511297	0.001546
RNF113A	31.27302	15.99051	0.51132	0.000483
CBX8	7.068379	3.615195	0.51146	0.025213
ARAP1	10.65888	5.452122	0.51151	8.86E-05
REV1	4.138518	2.117231	0.511592	0.002334
KANSL1L	0.573885	0.29364	0.511671	0.005402
NAXE	33.72937	17.25837	0.511672	0.001726
ZRSR2	6.429067	3.289808	0.511709	0.037976
EEF1E1	63.89463	32.71201	0.511968	0.032842
ZSCAN21	4.681006	2.396738	0.512013	0.000326
MOSPD1	14.37404	7.360575	0.512074	0.023771
SSPN	1.162586	0.595393	0.512128	0.009296
REEP6	11.10446	5.68821	0.512246	0.016659
RPS15A	2751.238	1409.721	0.512395	0.001971
STIL	9.937392	5.093031	0.512512	0.000559
TSNAXIP1	0.506746	0.259855	0.512791	0.18618
LOC10798	1.466466	0.752665	0.513251	0.035927
CAMLG	45.25851	23.25344	0.513792	0.05083
GMCL2	0.032908	0.01691	0.513845	0.572586
SNRNP70	164.7584	84.66472	0.513872	0.006309
ZNF746	9.918934	5.09723	0.513889	7.57E-05
ALMS1	2.857343	1.468539	0.513953	0.00354
DPH1	13.65083	7.016522	0.514	0.001231
ARID4A	3.254318	1.673775	0.514324	0.014464
RPL17	16.28401	8.376262	0.514386	0.019739
GPBP1	37.29317	19.18764	0.514508	0.001528
POM121C	12.54523	6.455283	0.514561	0.007009
USP39	44.97258	23.15326	0.514831	0.000193
UFL1	11.28255	5.808719	0.514841	0.00359
GXYLT1	6.265855	3.228183	0.515202	0.002356
NPIPB4	0.460408	0.237297	0.515406	0.204679

SBNO1	6.987588	3.601539	0.51542	0.000202
UBA2	88.54662	45.64886	0.515535	0.001196
OTULINL	2.040913	1.052279	0.515592	0.009998
IGHMBP2	4.160863	2.145539	0.515648	0.005767
POLA1	7.9032	4.077213	0.515894	0.000904
RAD18	13.73731	7.092991	0.51633	0.000923
KCTD1	3.115498	1.609115	0.516487	0.020094
SAXO1	0.028595	0.01477	0.516521	0.495523
TASP1	2.353811	1.215887	0.516561	0.034802
DISP2	0.149715	0.077359	0.516707	0.063692
POLR3C	8.200313	4.237713	0.516775	0.147112
NPM2	0.068399	0.035353	0.516865	0.491563
UBE2I	49.80786	25.74495	0.516885	0.004546
ZNF75A	1.855128	0.959707	0.517327	0.247298
EMD	125.4562	64.90312	0.517337	0.002005
PLEKHG6	0.024744	0.01281	0.517719	0.612048
FRZB	0.229516	0.118835	0.517764	0.398611
XIAP	9.528218	4.935032	0.517939	0.001382
GGT5	0.451147	0.233695	0.518001	0.17553
SFT2D3	3.362401	1.742335	0.518182	0.268102
PLAGL2	10.97211	5.68598	0.518221	0.027623
C14orf28	1.692212	0.877198	0.518373	0.016723
EGFR	7.729916	4.00709	0.518387	0.074627
MFSD8	4.289612	2.223935	0.518447	0.003432
KMT2E	4.724295	2.450681	0.51874	0.007431
GCN1	31.36195	16.28521	0.519266	0.001186
DGAT1	10.49847	5.452714	0.519382	0.004767
CAPS	4.680337	2.431068	0.519421	0.014734
CENPU	44.32938	23.03088	0.51954	0.028124
MDP1	1.132844	0.588609	0.519585	0.021487
DGKQ	5.379571	2.795228	0.519601	0.001993
TRIM33	6.07516	3.157066	0.519668	0.001575
SETSIP	0.488654	0.253942	0.519676	0.098884
ZCCHC10	9.1476	4.754433	0.519747	0.007164
BOD1L1	6.725537	3.497116	0.519976	0.068945
ZNF773	2.639752	1.372912	0.520091	0.001388
LPO	0.132686	0.069015	0.520132	0.108347
MPC1	9.666645	5.02999	0.520345	0.003594
COX19	5.080121	2.644184	0.520496	0.001169
KYAT1	7.880696	4.104338	0.520809	0.216801
OSBPL7	2.233719	1.163394	0.520833	0.005641
LANCL1	27.40121	14.27453	0.520945	0.004037
WDFY3	3.041281	1.584698	0.521063	0.062722
EVI5	2.344253	1.221543	0.52108	0.002977
TRAF4	9.295574	4.845514	0.521271	0.015274
RAB30	0.69617	0.362898	0.521278	0.018442
KNOP1	15.89586	8.28659	0.521305	0.00055
LDHD	1.844247	0.961417	0.521306	0.013292
RWDD2A	3.832462	1.998215	0.521392	0.030745
TMEM144	1.358584	0.708498	0.521498	0.114332
INTS8	7.626291	3.978137	0.521635	0.015471
RAB6A	36.16541	18.86912	0.521745	0.000808
VARS	31.79415	16.58983	0.521789	0.047395
PHOSPHC	2.156248	1.125222	0.521843	0.056044
TOMM70	44.03822	22.98322	0.521893	0.011798
SSX2IP	10.61891	5.543241	0.522016	0.000939
NSL1	3.362924	1.75583	0.522114	0.000154
CLYBL	1.663696	0.868792	0.522206	0.004897

CCNF	10.71706	5.596819	0.522235	0.002038
R3HCC1L	7.312289	3.823063	0.522827	0.005491
ZNF48	6.133313	3.209291	0.523256	0.025587
RBMX2	43.39347	22.7069	0.523279	0.008447
TMEM175	6.817611	3.568375	0.523405	0.02105
CSGALNA	9.152549	4.791167	0.523479	0.0482
PBRM1	9.458319	4.951505	0.523508	0.005911
KLHL25	3.332708	1.744779	0.523532	0.002067
PCTP	9.662434	5.059477	0.523623	0.003039
CELF1	17.44244	9.134145	0.523674	0.012378
VWA5B1	0.007783	0.004077	0.523886	0.393251
TRIM24	15.26681	7.998247	0.523898	0.00664
CD83	4.440972	2.326838	0.523948	0.071793
ULK3	16.92512	8.868465	0.523982	0.02118
CHIC1	2.539069	1.33045	0.523991	0.005575
TNS1	0.366568	0.192079	0.523993	0.000595
RFNG	27.3742	14.34586	0.524065	0.001455
QRICH2	0.188712	0.0989	0.524078	0.055198
CC2D1A	21.47537	11.26136	0.524385	0.008532
CMTM7	11.95287	6.271659	0.524699	0.00243
CNOT6	11.33545	5.947981	0.524724	0.001936
NSUN4	5.676604	2.978753	0.524742	0.004269
RNF144A	1.068244	0.56063	0.524814	0.008357
LMCD1	4.514518	2.369338	0.524826	0.065474
DDHD2	13.41752	7.04347	0.524946	0.004486
SLC7A9	0.042407	0.022268	0.525097	0.53667
MPP6	3.929726	2.065181	0.525528	0.011752
CSRNP2	8.331427	4.381581	0.52591	0.002307
MTMR11	4.951298	2.604114	0.525946	0.182455
RGS9BP	0.312386	0.164319	0.526014	0.093889
CCDC59	30.93044	16.26985	0.526014	0.028717
SLC17A5	8.949088	4.707578	0.52604	0.023703
KIF11	31.18534	16.40742	0.526126	0.00816
PLXDC2	0.827072	0.435197	0.52619	0.060059
PHLDB3	3.32896	1.752993	0.526589	0.004448
PURA	7.133381	3.756408	0.526596	0.004401
KIAA1191	76.92658	40.54861	0.527108	0.006511
HECTD1	9.532922	5.025021	0.527123	0.019324
STX12	26.93719	14.20951	0.527505	0.008493
DONSON	26.3286	13.88914	0.52753	0.025465
CROCC2	0.012691	0.006695	0.527532	0.574067
HSF4	3.15927	1.666783	0.527585	0.044596
ILF2	322.6749	170.2413	0.527594	9.84E-05
JPT2	48.84755	25.7723	0.527607	0.000384
PPP2R2D	7.514318	3.9656	0.527739	0.061233
F2	0.045197	0.023853	0.527755	0.577367
PPP2R3C	23.4345	12.37122	0.527906	0.002478
IMPACT	13.24563	6.994307	0.528046	0.059219
KHDRBS1	129.8803	68.5853	0.528066	3.32E-05
NUSAP1	81.75251	43.17476	0.528115	0.00053
KIAA2026	1.456243	0.769123	0.528156	0.011007
CASP2	17.64926	9.322427	0.528205	0.00027
WDSUB1	4.996034	2.638955	0.52821	0.000536
PERP	36.8166	19.44785	0.528236	0.023066
LITAF	20.07634	10.61835	0.528899	0.003012
WASF3	3.719448	1.967461	0.528966	0.000876
EGFL6	0.062418	0.033017	0.528972	0.496169
ZNF33A	5.046374	2.669783	0.52905	0.00266

IMPDH2	367.4126	194.4207	0.529162	0.009326
TMEM8B	2.880944	1.524659	0.529222	0.007838
GPATCH1	11.36178	6.013705	0.529293	0.004322
CEP72	6.24467	3.305939	0.529402	0.003563
LOC11226	0.205068	0.108607	0.529613	0.075324
LYPLA1	21.14453	11.19893	0.529637	0.002427
SPATA31A	0.022206	0.011762	0.529703	0.272108
DIP2B	5.524561	2.927933	0.529985	0.022242
SOS1	5.525981	2.928747	0.529996	0.022551
SCAF8	12.6763	6.71843	0.529999	0.019396
ZNF212	9.276741	4.918296	0.530175	0.009982
TRPC1	2.384135	1.264086	0.530207	0.150509
ZFP64	6.595082	3.497331	0.530294	0.001369
DPY19L4	5.089782	2.699247	0.530327	0.002418
CLDN4	0.442357	0.234644	0.530441	0.381626
NUDT15	18.66783	9.904769	0.53058	0.000596
PELP1	31.53117	16.73187	0.530646	0.000647
MTIF2	16.54294	8.780235	0.530754	0.060061
TTC19	17.46946	9.273158	0.530821	0.023314
HPRT1	121.9858	64.76693	0.530938	0.023584
ZNF669	2.506878	1.331112	0.530984	0.043325
MAP2K7	19.6032	10.4098	0.531026	0.026081
CREB3L4	5.001417	2.656403	0.53113	0.153972
DNAJB4	6.418024	3.40883	0.531134	0.009807
NECAP1	19.93701	10.59024	0.531185	0.001912
NDUFV3	20.45861	10.86929	0.531282	0.002467
CENPJ	5.218554	2.773435	0.531457	0.001087
NTN4	1.266478	0.673122	0.531491	0.009614
TMEM183	2.197932	1.168186	0.531493	0.030066
BRD8	23.98854	12.75724	0.531806	0.001764
SLC36A4	5.486769	2.917903	0.531807	0.001732
ZNF546	0.558947	0.297254	0.531812	0.190462
ETFDH	17.00479	9.044042	0.531852	0.001364
SETD1A	11.76495	6.260193	0.532105	0.001846
FBXO11	16.63332	8.855542	0.532398	5.05E-05
PPM1K	3.728446	1.985369	0.532492	0.007931
BUD13	11.3408	6.039042	0.532506	0.010739
KAT8	19.85766	10.57687	0.532634	0.002633
C4orf48	10.58339	5.637307	0.532656	0.009871
PDCD2L	19.70696	10.49764	0.532687	0.109162
SSR2	393.9452	210.0266	0.533137	0.000337
VIPAS39	17.16111	9.150273	0.533198	0.008318
SGPL1	9.254341	4.934414	0.5332	0.007505
ANGEL2	6.639143	3.541475	0.533423	0.001828
TMEM242	8.355854	4.457447	0.533452	0.001324
SMIM26	110.9077	59.18633	0.533654	0.000698
SGTB	6.771179	3.613525	0.533663	0.00061
PHF12	9.291641	4.960412	0.533857	0.006131
MAP7D3	12.12491	6.47443	0.533978	0.004753
MSH6	37.88045	20.23792	0.534258	0.000426
OXNAD1	1.446149	0.772653	0.534283	0.008473
ACOT11	0.25174	0.134555	0.534502	0.536469
RNF115	10.80405	5.775854	0.534601	0.008715
UBR3	4.664754	2.494978	0.534857	0.041776
GIGYF2	11.46026	6.131725	0.535042	0.010546
CAPN7	17.33605	9.277582	0.535161	0.002956
PPWD1	25.20913	13.49148	0.535182	0.005891
GBF1	20.90061	11.18694	0.535245	0.023172

NTSR1	0.057266	0.030653	0.535277	0.304005
SLC6A9	3.835661	2.053837	0.535458	0.119543
LDLRAD3	6.934668	3.713288	0.535467	0.036596
TEAD3	6.41923	3.437477	0.535497	0.009849
TSTD2	9.590733	5.137727	0.535697	0.017496
NDUFAF3	80.34913	43.05444	0.535842	0.003509
CEP78	8.125407	4.354121	0.535865	4.48E-05
NFAT5	4.432614	2.376203	0.536073	0.057835
DEPTOR	1.391259	0.74592	0.536148	0.044356
SCARB1	27.57133	14.78283	0.536167	0.001108
CTIF	11.80382	6.329364	0.536213	0.02262
RIC1	3.454788	1.853115	0.536391	0.004682
UBE2O	13.07255	7.012153	0.536403	0.002475
TBX18	2.488494	1.334867	0.536416	0.000446
FBXL20	3.5848	1.923099	0.536459	0.001264
TEAD4	11.74611	6.303119	0.536613	0.006894
INPP5B	6.700139	3.596307	0.536751	0.02374
RGS22	0.025366	0.013619	0.536897	0.540613
ADCY3	11.59391	6.224844	0.536907	0.004414
FAM216A	25.43764	13.65959	0.536983	0.049774
PRPF3	44.13915	23.7029	0.537004	0.005934
BOC	2.587812	1.38973	0.537029	0.026012
USP11	55.342	29.73053	0.537215	0.031335
MECP2	10.10148	5.427392	0.537287	0.003099
ZNF251	4.038265	2.170658	0.537523	0.026769
NEUROD1	0.030698	0.016505	0.537663	0.63366
EXOSC2	33.20742	17.86747	0.538057	0.0452
SMCHD1	6.268452	3.373705	0.538204	0.026464
JRK	2.113793	1.137862	0.538303	0.000755
PAICS	180.3213	97.07782	0.53836	0.015575
NFYB	13.51118	7.275329	0.538467	0.046917
SMAP1	9.754286	5.254385	0.538675	0.034365
QKI	3.970418	2.139172	0.538777	0.016964
CNOT9	27.33749	14.74075	0.539214	0.000319
RPUSD4	11.94454	6.441542	0.539287	0.095357
RECQL5	5.934178	3.200368	0.539311	0.023879
FOXK2	22.68702	12.23837	0.539444	0.005464
CCT6B	1.159202	0.625422	0.539528	0.338672
CRYZL1	22.33105	12.0493	0.539576	0.004311
CEBPZOS	23.54997	12.70945	0.53968	0.00034
SIK3	3.239085	1.748793	0.539903	0.006611
TRIM65	14.99084	8.094625	0.539971	0.042923
NAA35	7.248433	3.914766	0.540084	0.008372
DNAJB9	10.68933	5.774526	0.540214	0.021612
GALNT11	18.75516	10.13251	0.540252	0.003417
PGAP2	5.990649	3.237146	0.540367	0.007696
KLF12	5.836179	3.154159	0.540449	0.014302
SCYL3	3.447925	1.86348	0.540464	0.126032
BCAT2	20.42709	11.04022	0.54047	0.010688
IQUB	0.242636	0.131194	0.540703	0.035967
RIC8B	3.14366	1.699859	0.540726	0.000614
COPZ1	190.4868	103.0638	0.541055	0.000492
DMPK	13.95337	7.55073	0.54114	0.083153
RASD2	0.40612	0.219877	0.541409	0.296463
ATXN1L	6.484144	3.510888	0.541457	0.042189
ADAMTS1	0.349284	0.189138	0.541503	0.095025
ZSCAN20	1.464507	0.793064	0.541523	0.014874
FBXL14	1.519276	0.823082	0.54176	0.088889

ATP5MG	208.7515	113.0937	0.541762	0.000131
PRKD3	14.33915	7.76901	0.541804	0.004463
TMEM263	26.61154	14.4203	0.541881	0.038726
ZDHHC9	17.14587	9.291855	0.54193	0.000151
HDAC2	48.27156	26.16306	0.541997	0.034597
RPL19	2018.156	1094.03	0.542094	0.002343
RPL37A	4021.355	2181.857	0.542568	0.008437
STAU2	7.736205	4.197974	0.54264	0.006965
C1QBP	267.8699	145.3633	0.542664	0.053213
ZNF517	0.651262	0.353465	0.542739	0.06773
ZNF691	5.073257	2.753771	0.542801	0.051956
BORA	16.93262	9.193441	0.542943	0.018664
KIF15	14.07992	7.645109	0.54298	0.005803
KCTD20	13.7017	7.440315	0.543021	0.016341
NUP54	15.8053	8.583745	0.543093	0.002346
PDP2	4.981282	2.705656	0.543165	0.029217
ZNF614	2.69529	1.464164	0.543231	0.035592
CSNK2A2	24.27699	13.18962	0.543297	0.002601
CHD6	5.071297	2.755653	0.543382	0.038386
EEF1A1	3019.247	1641.047	0.543528	0.002739
ZNF226	4.91837	2.673475	0.543569	0.001239
ISLR	0.361178	0.196328	0.543576	0.349445
RETSAT	17.0418	9.263749	0.54359	1.88E-06
AGPAT2	19.98218	10.86316	0.543642	0.004265
CEBPZ	39.10139	21.26797	0.543919	0.019676
NOG	2.197267	1.195402	0.544041	0.213279
SREK1	10.17515	5.536711	0.544141	0.018984
MPZ	1.561943	0.849932	0.544151	0.175422
SPCS2	27.10123	14.74741	0.54416	0.004815
ATG4B	16.04326	8.731977	0.544277	0.051565
TMEM39A	14.83732	8.076248	0.54432	0.025887
LRRC34	2.69137	1.465057	0.544354	0.163082
NT5C3B	62.28177	33.92941	0.544773	0.000314
ZNF417	1.415494	0.771124	0.544774	0.146254
TRAK1	6.321109	3.443844	0.544816	0.002557
AS3MT	7.920496	4.317099	0.545054	0.008073
POLG	24.27564	13.23682	0.545272	0.020652
IARS	143.7685	78.4641	0.545767	0.034298
RPS19	1152.912	629.2232	0.545769	0.003543
TBC1D3I	0.052166	0.028476	0.545865	0.428893
CALCOCC	49.95444	27.27744	0.546046	0.013315
ITGB3BP	28.34485	15.48445	0.546288	0.009137
MYO1D	11.62817	6.352374	0.546292	0.008403
TMEM129	15.30632	8.3662	0.546585	0.005578
TMEM97	26.29457	14.37801	0.546805	0.110327
ZSWIM7	13.7943	7.544669	0.546941	0.002888
C1GALT1(	2.256408	1.234603	0.547154	0.143698
STXBP5L	0.200255	0.109571	0.54716	0.049936
SPATA13	1.429914	0.782639	0.547333	0.037283
DNAJC2	29.45292	16.12187	0.547378	0.030465
CXorf38	11.0624	6.055395	0.547385	0.022173
THAP6	3.566108	1.952968	0.547647	0.069809
CASKIN2	4.608696	2.525661	0.548021	0.011944
COA5	24.32534	13.33436	0.548167	2.52E-05
DHFR	46.91818	25.71982	0.548184	0.003846
RSAD1	25.6574	14.0872	0.54905	0.007897
RAD21	92.52812	50.80856	0.549115	0.005763
PCNP	91.23161	50.10509	0.549208	0.000913

FAM117B	2.000991	1.098972	0.549214	0.026923
THEM4	6.402585	3.516617	0.54925	0.044442
ELMOD3	7.291566	4.00609	0.549414	0.011456
NEK7	6.288378	3.455769	0.549549	0.000752
DNAH10	0.071055	0.039049	0.549562	0.087302
FAM53C	22.195	12.19949	0.54965	0.012654
SKAP2	9.934179	5.462973	0.549917	0.008363
AP3M1	21.97447	12.08777	0.550082	0.007288
OSBPL10	4.95188	2.724816	0.550259	0.001047
CCDC28B	6.226109	3.426611	0.550362	0.029533
SMARCAD	8.059546	4.436564	0.550473	0.045195
CYP24A1	0.186642	0.102759	0.550566	0.446623
RABL2A	2.262185	1.245561	0.550601	0.042677
KCNK7	0.095662	0.052688	0.550774	0.532465
RBM12	15.31052	8.43354	0.550833	0.002587
TMEM126	22.777	12.55057	0.551019	0.027867
SENP1	7.343648	4.046526	0.551024	0.004966
LMBRD2	2.476199	1.365234	0.551343	0.034336
MXI1	17.12335	9.441436	0.551378	0.001397
RNF19B	7.889044	4.351696	0.551613	0.015954
SLC2A13	0.833699	0.459944	0.55169	0.000115
USF1	28.49628	15.72134	0.551698	0.003368
SLC25A42	5.561623	3.069196	0.551853	0.006595
DDX42	34.91803	19.27731	0.552073	0.001968
FBXO46	8.129322	4.489334	0.55224	0.004976
PPP4R2	21.02367	11.61253	0.552355	0.0025
DDX52	8.542647	4.719594	0.552474	0.034208
CLPB	11.16615	6.169495	0.552518	0.007696
RSBN1	2.070847	1.144199	0.552527	0.000124
PDCD2	23.10553	12.76653	0.552531	0.014264
PPRC1	12.39768	6.856252	0.553027	0.005763
SUMO2	366.309	202.6678	0.55327	0.00241
TBC1D7	0.114573	0.063395	0.553312	0.357718
NAA16	4.041221	2.237322	0.553625	0.002765
MARK3	36.13585	20.01515	0.553886	5.17E-05
CALCOCC	23.01369	12.74728	0.5539	0.036668
ERCC2	16.26733	9.010596	0.553908	0.002393
TMF1	12.12282	6.716704	0.554055	0.05612
GET4	26.96782	14.9491	0.554331	0.006136
6-Mar	14.75934	8.181679	0.554339	0.002416
ZNF619	1.528633	0.847558	0.554455	0.036708
WDR63	1.471264	0.815753	0.554457	0.126217
TLNRD1	12.16859	6.747276	0.554483	0.03453
PSME4	18.85715	10.45816	0.554599	0.00452
SORT1	11.39783	6.323841	0.554829	0.032652
GPR137B	2.211137	1.226819	0.554836	0.00719
NSD2	11.75448	6.523298	0.554963	0.014944
GRAMD1A	18.75676	10.40959	0.554978	0.001394
CCNL2	19.52157	10.8363	0.555094	0.074991
EDC3	21.15032	11.74072	0.555108	0.007655
PRICKLE4	1.41651	0.786441	0.555196	0.017916
RANBP10	5.403407	3.000322	0.555265	0.025132
CFAP206	0.252259	0.140125	0.555482	0.351268
ZZEF1	4.065137	2.260043	0.555957	0.032718
PANK2	4.369082	2.429187	0.555995	0.000339
PAPD7	9.580602	5.33101	0.556438	0.045031
ISOC1	22.33212	12.42695	0.556461	0.08364
HTATSF1	76.69562	42.69514	0.556683	0.015481

TERF2	21.0496	11.72041	0.5568	0.010957
EIF3A	69.60598	38.75968	0.556844	0.001002
SMN1	17.7441	9.881458	0.556887	0.000332
UBE2G2	31.95911	17.80033	0.556972	0.000466
BEST1	1.572559	0.876066	0.557096	0.332233
HMGXB4	11.469	6.39098	0.55724	0.004796
RNF123	18.11972	10.09999	0.557403	0.000429
SNX27	8.257202	4.606962	0.557933	3.96E-05
EIF4A2	215.7037	120.3608	0.557991	0.011402
PJA1	19.43614	10.84615	0.558041	0.00161
CHM	3.830401	2.137959	0.558155	0.011309
MCM3AP	19.34005	10.79497	0.558166	0.034345
PTBP1	133.7205	74.6417	0.558192	0.000378
GULP1	1.094513	0.611005	0.558243	0.041661
HCLS1	0.128812	0.071927	0.558385	0.403607
GEN1	4.90906	2.741433	0.558444	0.067268
RTN2	7.705833	4.304821	0.558644	0.012946
AAAS	47.98446	26.80652	0.55865	0.037543
CDCA4	41.54785	23.21402	0.55873	0.008511
AFTPH	10.77937	6.024176	0.558861	0.002308
ATOH1	0.195385	0.109196	0.558876	0.397133
ARHGAP4	0.702084	0.392409	0.558921	0.004525
C6orf62	30.9648	17.32389	0.559471	6.87E-06
CFAP97D1	0.023721	0.013272	0.559527	0.616463
ILF3	72.00491	40.29108	0.55956	0.019965
KLHDC8B	6.217155	3.479064	0.559591	0.005333
PDS5B	5.370699	3.005708	0.559649	0.003915
C11orf95	4.694762	2.627581	0.559683	0.042271
TAB1	15.87198	8.884647	0.559769	0.017694
ZNF805	2.123733	1.189296	0.560003	0.02746
RGP1	7.378609	4.132054	0.560004	0.03097
FOXRED2	12.65364	7.088135	0.560166	0.089388
SLC25A19	4.8053	2.693192	0.560463	0.044103
KCNH8	0.009367	0.00525	0.56049	0.693922
GGNBP2	37.60789	21.07935	0.560503	0.000438
RBM34	45.29335	25.38836	0.560532	0.030577
HNRNPA1	807.211	452.4793	0.560547	0.113893
RPL9	1748.792	980.2875	0.560551	0.000827
MIER1	8.314104	4.660761	0.560585	0.000385
SFRP4	0.227904	0.127804	0.560779	0.040692
OSGEPL1	5.552749	3.115037	0.56099	0.091226
IFT172	5.738139	3.21907	0.560995	0.027877
MARK2	12.29946	6.900555	0.561045	0.00326
RPL13	610.1774	342.3408	0.561051	0.00921
PPP1R12A	8.258857	4.633973	0.561091	0.000823
MED9	15.46957	8.683284	0.561314	0.002479
DDIAS	7.2572	4.074521	0.561445	0.007634
C1orf112	7.977173	4.481669	0.561812	0.057038
SH2D6	0.124068	0.069706	0.561838	0.184371
ABCD3	15.33215	8.614302	0.561845	0.006496
GAMT	21.36895	12.00752	0.561914	0.029142
ZZZ3	10.3154	5.796775	0.561954	0.014723
TBCC	12.30757	6.920528	0.562298	0.046155
BCL10	6.662134	3.746408	0.562343	0.018649
TRA2A	38.00393	21.3759	0.562466	0.005704
BZW2	39.40095	22.16169	0.562466	0.0732
GTPBP4	58.73938	33.04955	0.562647	0.004868
GDI1	197.9964	111.4025	0.562649	0.038697

CYTIP	0.121626	0.068441	0.562715	0.363114
PFKM	26.49691	14.91514	0.562901	0.002841
HIPK1	8.389794	4.725238	0.563213	0.027772
RHBDD1	3.457695	1.947651	0.56328	0.052845
MCCC2	19.47462	10.97043	0.563319	0.043657
RPS27A	914.4551	515.3024	0.563508	9.41E-05
CARF	0.822216	0.463343	0.563529	0.277744
FAM185A	0.672226	0.378876	0.563614	0.067021
EID2B	7.378143	4.158792	0.563664	0.063107
UBAP2L	45.63666	25.73581	0.563929	0.003743
PLD6	2.781649	1.568761	0.563968	0.055354
MYO9A	4.129207	2.329128	0.564062	0.034312
TMEM69	38.65454	21.81066	0.564246	0.031172
NCAPG	40.37256	22.7826	0.564309	0.014526
BCLAF1	23.01233	12.98859	0.564419	0.001243
RPL23	2437.936	1376.099	0.564452	0.002611
FANCC	2.732776	1.542553	0.564464	0.065073
NBDY	9.642034	5.443016	0.564509	0.001466
DCPS	5.272773	2.976837	0.564568	0.024377
STAM	8.605989	4.858852	0.56459	0.014898
UNC119B	34.36195	19.40128	0.564615	0.000842
LSM8	7.794811	4.401336	0.564649	0.001499
SNX30	5.117264	2.890407	0.564835	0.000457
OMA1	13.91704	7.860838	0.564835	0.14657
FKBP4	50.30654	28.41711	0.564879	2.04E-07
MTX3	9.535646	5.38649	0.564879	0.002804
MTO1	8.151404	4.607214	0.565205	0.054366
NDNF	1.293793	0.731316	0.56525	0.107016
OARD1	19.55341	11.05544	0.565397	0.025176
GMEB2	9.754703	5.517232	0.565597	0.055145
CHAF1A	35.61069	20.1519	0.565895	5.17E-05
SDHD	54.32584	30.74483	0.565934	0.005801
DCTPP1	77.08192	43.6255	0.565963	0.124915
TRPM4	5.848508	3.310233	0.565996	0.014652
WFDC5	0.037338	0.021145	0.56632	0.617533
SLC9A3	1.919015	1.086998	0.566435	0.022426
MYBBP1A	15.82055	8.963819	0.566593	0.077575
DHX30	34.51068	19.55659	0.566682	0.005647
LIMK2	5.607915	3.177982	0.566696	6.60E-05
TYW1B	0.432782	0.245271	0.566731	0.083196
SRSF8	4.906454	2.780739	0.566751	0.063957
KCTD13	6.459317	3.66123	0.566814	0.037317
POLB	24.72015	14.01449	0.566926	0.009788
PECR	3.019126	1.71173	0.566962	0.005762
TIGD3	0.410398	0.232776	0.567196	0.30373
TRIT1	16.5628	9.404978	0.567837	0.00036
CUL9	2.430958	1.380391	0.567838	0.07648
ZNRF3	2.272014	1.290254	0.56789	0.005736
REXO5	7.275537	4.132419	0.567988	0.064432
DBP	5.017105	2.850978	0.568252	0.134676
PPIL2	12.51595	7.112837	0.568302	0.009964
MYH7B	0.069307	0.039388	0.568317	0.083413
FRRS1	2.446136	1.390422	0.568415	0.050562
SPEM1	0.057536	0.032708	0.568484	0.490293
FBXL12	14.92434	8.486245	0.568618	0.026442
USP25	7.010368	3.986335	0.568634	0.030725
NDUFS1	37.7219	21.45496	0.568767	0.004042
WDR37	5.47462	3.115064	0.569001	0.026643

CHERP	22.20775	12.63661	0.569018	0.043438
MEGF9	4.061526	2.311296	0.569071	0.027736
WDR12	13.81434	7.863323	0.569215	0.12455
CPSF6	18.67336	10.63042	0.569283	0.003127
PARP15	0.00682	0.003882	0.569288	0.545041
TYW1	4.483628	2.552628	0.569322	0.014299
ZSCAN29	4.553627	2.594373	0.569738	0.021093
ELMO3	0.774106	0.441082	0.569796	0.483393
KIF16B	2.111466	1.203575	0.570019	0.006787
EIF2AK3	6.118145	3.487688	0.570057	0.00705
SFT2D2	3.520507	2.006956	0.570076	0.138999
SMC4	64.6342	36.85058	0.570141	0.001615
POT1	15.14784	8.639927	0.570373	0.014853
ZNF630	1.163544	0.66382	0.570516	0.046394
CYP3A43	0.016943	0.009669	0.570664	0.621594
C17orf58	12.22971	6.981326	0.57085	0.068959
SLMAP	8.339575	4.760811	0.57087	0.001734
TIAL1	21.09082	12.0415	0.570936	0.006566
UCK2	22.37917	12.77729	0.570946	0.034078
IL13	0.03661	0.02092	0.571429	0.548433
MAP3K14	2.107083	1.204164	0.571484	0.151461
CENPE	12.02371	6.873642	0.571674	0.052639
ADRB2	0.633116	0.361946	0.57169	0.140945
GLYR1	14.55766	8.32474	0.571846	0.006422
KIFAP3	14.77971	8.455762	0.572119	0.011594
TFCP2	14.13764	8.089321	0.572183	0.051668
MTHFD1	84.36466	48.30206	0.572539	0.039132
DGCR6	18.62679	10.66609	0.572621	0.018831
PPFIA4	3.798166	2.175877	0.572876	0.032095
SNX6	20.43624	11.70932	0.572968	0.010285
ALB	0.048689	0.027902	0.573072	0.500355
GEMIN8	3.944038	2.260426	0.573125	0.00179
PFKFB1	0.147327	0.084463	0.573307	0.355428
VPS26B	17.38153	9.967736	0.573467	0.003583
UBN1	8.804975	5.050249	0.573568	0.000129
XPOT	44.86702	25.74067	0.57371	0.004296
KCTD2	11.16408	6.405457	0.573756	0.086381
ARHGEF25	10.39132	5.966598	0.574191	0.015162
BMS1	15.80158	9.075345	0.574332	0.000313
LOC10798	0.600889	0.345146	0.574392	0.317687
PGAM5	32.19778	18.49936	0.574554	0.045419
TST	11.84451	6.80534	0.574557	0.037961
UTS2B	0.061532	0.035357	0.574614	0.374482
MAP2K6	0.885855	0.509178	0.574787	0.142768
LDB1	25.08356	14.41819	0.574806	0.000505
LRTOMT	1.060084	0.609539	0.574992	0.116702
ZNF707	3.388973	1.948744	0.575025	0.046499
NICN1	9.038367	5.198052	0.57511	0.027946
MAMSTR	1.660277	0.955205	0.575329	0.225523
REELD1	0.135586	0.078036	0.575544	0.495181
TMEM249	0.171382	0.09864	0.575558	0.396705
LACTB2	10.26905	5.911587	0.57567	0.163335
FBXO48	1.71472	0.987137	0.575684	0.079876
KIAA1522	13.89855	8.001966	0.575741	0.009626
POLA2	23.89598	13.75849	0.575766	0.01643
IL23A	0.993238	0.571908	0.575802	0.3155
ATF2	10.99853	6.333228	0.575825	0.000685
RAD51C	19.02845	10.95805	0.575877	0.078843

PRPF38B	24.50723	14.11371	0.5759	0.000793
NDUFA12	135.367	77.96256	0.575935	0.012901
LOC10536	0.01972	0.011358	0.575966	0.368594
GTF3C2	19.18522	11.05218	0.576078	0.001735
NUDT4	4.400146	2.535047	0.576128	0.001327
SNRPF	128.7965	74.2227	0.576279	0.014607
NEPRO	14.19556	8.183045	0.576451	0.013481
NKIRAS2	23.53804	13.56854	0.576451	0.002509
GSTCD	3.592883	2.07135	0.576515	0.038049
ARGLU1	58.07846	33.4856	0.576558	0.04601
STMN2	0.072747	0.041943	0.576565	0.216665
NFATC2	0.769602	0.443728	0.576569	0.351874
TOMM7	249.3885	143.7954	0.576592	0.00787
CNOT10	21.7365	12.53369	0.57662	0.092981
ZCCHC2	2.796643	1.613456	0.576926	0.016466
RC3H1	3.009812	1.736693	0.57701	0.015491
MFNG	0.515901	0.297706	0.577061	0.241793
ARHGAP2	0.032496	0.018752	0.577063	0.478206
WDR91	6.189889	3.572706	0.577184	0.006404
FUBP3	14.81138	8.548968	0.577189	0.002656
SMC6	12.09902	6.98463	0.577289	0.002443
LOC10099	0.029022	0.016756	0.57738	0.581021
COL4A5	5.303504	3.063242	0.577588	0.046751
ANP32A	176.2627	101.8178	0.577648	0.011128
RSPH3	3.114717	1.799354	0.577694	0.048299
DIP2C	3.67189	2.121597	0.577794	0.006379
RFX5	24.47748	14.14891	0.578038	0.001007
ME2	13.8652	8.014822	0.578053	0.021183
ALDH16A1	5.32778	3.080163	0.578133	0.001789
TWISTNB	13.45506	7.781176	0.578308	0.026018
ZDHHC8	13.34218	7.715975	0.578314	0.003332
AIFM1	34.05673	19.69712	0.578362	0.042428
NACA2	3.465652	2.004649	0.578434	0.006848
WDR5	28.36133	16.40657	0.578484	0.000547
KCNH6	0.010952	0.006339	0.578776	0.543937
HDAC6	10.42661	6.035276	0.578834	0.027042
TADA2A	6.399548	3.705838	0.579078	0.095404
SLC4A10	0.016138	0.009345	0.579082	0.464941
PACSIN2	22.10063	12.80667	0.579471	0.017087
ZNF404	3.25596	1.887019	0.579559	0.007549
KDM5A	6.38856	3.70365	0.579732	0.006248
DUSP19	1.108986	0.643021	0.579828	0.188011
TMOD2	3.297149	1.912183	0.57995	0.089701
YTHDF2	50.254	29.15512	0.580155	0.02368
SMG7	17.89395	10.38325	0.580266	0.006247
NEK4	5.399194	3.13384	0.580427	0.012534
SMARCA1	39.39886	22.86891	0.580446	0.002323
LOC10798	0.031095	0.018053	0.580576	0.223001
OSBPL11	6.588164	3.825724	0.580697	0.008175
LCAT	4.588259	2.664538	0.58073	0.231532
RRP12	16.54468	9.612111	0.580979	0.081992
PPFIA2	0.121983	0.070877	0.581045	0.167223
POLR2B	51.35914	29.84476	0.581099	0.004686
COG3	12.75451	7.413871	0.581274	0.002294
DYRK4	16.97244	9.866468	0.581323	0.000322
PPTC7	10.00338	5.815637	0.581367	0.014035
EPC1	4.483339	2.606511	0.581377	0.003529
MVB12B	1.706291	0.992302	0.581555	0.02868

KIAA1109	3.527206	2.051429	0.581602	0.084354
CPLANE2	1.559764	0.907388	0.581747	0.271862
HILPDA	42.4366	24.69658	0.581964	0.118215
DGKB	0.005273	0.00307	0.582253	0.73635
LKAAEAR1	0.054781	0.031896	0.582253	0.73635
GPIHBP1	0.02259	0.013153	0.582253	0.73635
AQP10	0.029802	0.017352	0.582253	0.73635
ANKS6	6.051475	3.524908	0.582487	0.002214
ZC3H3	5.315065	3.09615	0.582524	0.008159
CDC25C	15.40471	8.97445	0.582578	0.018986
TMEM67	3.540831	2.06288	0.582598	0.134365
MMGT1	19.20492	11.18908	0.582615	0.009733
ATXN7L1	0.401246	0.233818	0.58273	0.126301
NPIPA7	1.106898	0.645106	0.582805	0.133055
NMD3	25.79897	15.03807	0.582894	0.026814
KRT4	0.052412	0.030558	0.583044	0.649223
VAV3	1.562904	0.911299	0.58308	0.093983
BLOC1S2	23.9318	13.95965	0.58331	0.005305
TUBG2	6.226209	3.632384	0.583402	0.020685
SHH	0.048606	0.02836	0.583467	0.60028
LBHD1	1.600513	0.934063	0.583603	0.298509
ZHX1	7.83494	4.572506	0.583605	0.017268
RBM28	9.953617	5.812852	0.583994	0.026278
SLC25A25	7.83754	4.57796	0.584107	0.068076
ZNF81	0.847923	0.49528	0.58411	0.179305
RHCE	0.774413	0.45245	0.584249	0.059702
ARHGAP3	2.330549	1.361861	0.584352	0.035958
EXOSC9	61.70985	36.0652	0.584432	0.017336
C8orf89	0.062848	0.036744	0.584646	0.516852
HSDL1	16.54648	9.674935	0.584713	0.000276
MCM9	2.477434	1.448999	0.584879	0.003959
TTC37	25.41508	14.86664	0.584954	0.005179
HIVEP2	2.524593	1.476808	0.584969	0.080729
MTFR1	11.35638	6.64313	0.584969	0.033973
MYO1F	0.335766	0.196434	0.585031	0.171275
OSBPL8	6.103261	3.57107	0.585108	0.010836
AHCYL2	4.899212	2.867167	0.58523	0.014292
FLAD1	15.76735	9.228322	0.58528	0.054604
RGL2	9.150067	5.355625	0.58531	0.000294
TSEN2	5.455545	3.193784	0.58542	0.165922
AMIGO1	1.106848	0.648373	0.585783	0.17716
PDE7A	6.788342	3.976972	0.585853	0.010617
ADGRF1	0.009144	0.005358	0.585944	0.558451
SPATS2	11.71097	6.862575	0.585996	0.001571
DDX6	18.95896	11.11172	0.586093	0.018925
SQLE	22.77595	13.35785	0.586489	0.031802
STRA6	0.957427	0.56171	0.586687	0.543637
CCDC51	17.65255	10.35705	0.586717	0.009333
SERPINF2	0.075706	0.044421	0.586759	0.621229
GLIS2	4.03009	2.364912	0.586814	0.046261
ARMCX5-	0.136058	0.079949	0.58761	0.24403
TSEN15	24.19406	14.21977	0.587738	0.00693
ST8SIA1	0.109474	0.064349	0.587805	0.187967
FOXJ3	12.2576	7.205177	0.587813	0.009113
DIAPH2	4.367628	2.567427	0.587831	0.027881
GNA13	9.216938	5.418067	0.587838	0.008395
ANAPC10	6.733527	3.958442	0.587871	0.005041
DOK6	0.180418	0.106071	0.587917	0.176181

SLC3A2	119.248	70.12669	0.588074	0.103249
SYNRG	6.482316	3.812456	0.588132	0.014304
RB1	10.91746	6.421352	0.588173	0.038954
GEMIN4	9.165931	5.391336	0.588193	0.013347
PARP11	2.279768	1.340957	0.588199	0.032458
SOS2	4.03494	2.373373	0.588205	0.062629
RBCK1	15.08122	8.870972	0.588213	0.003616
CEP131	8.822406	5.189831	0.588256	0.011228
C22orf46	3.693487	2.173215	0.588391	0.291559
CTBP2	10.50969	6.184208	0.588429	0.000752
CNTROB	14.50244	8.535815	0.588578	0.024082
STK38	12.13505	7.143324	0.588652	0.022396
C9orf50	0.042898	0.025252	0.588661	0.637658
SNRK	3.93136	2.315168	0.588897	0.001747
NACA	134.7847	79.39589	0.589057	0.005535
STT3A	74.14111	43.68275	0.589184	0.008655
CALM3	206.8742	121.9236	0.589361	0.01655
ZNF776	2.010294	1.184822	0.589377	0.275801
SHC4	0.37036	0.218378	0.589638	0.290508
GXYLT2	6.673494	3.935419	0.589709	0.003458
RAB5B	4.691433	2.766892	0.589775	0.034074
XPO7	27.44039	16.18514	0.589829	0.000305
LOC10192	0.033231	0.019607	0.590021	0.562713
FAM228A	0.288276	0.170105	0.590078	0.044644
SRP72	52.33637	30.88299	0.590087	0.013778
SMARCC2	27.27987	16.09798	0.590105	0.002272
ADM5	1.377691	0.813105	0.590194	0.335948
XKRX	0.148777	0.087831	0.590352	0.457212
WDR75	19.97505	11.79401	0.590437	0.008678
IFFO1	11.43182	6.751992	0.590631	0.005113
FAM107B	6.521861	3.853536	0.590864	0.018128
TSC1	6.228519	3.680402	0.590895	0.058905
CD36	0.02869	0.016953	0.590913	0.470395
PTK2B	0.99806	0.589767	0.590913	0.104733
ING5	3.379583	1.997695	0.591107	4.25E-05
SPATA24	3.395395	2.007122	0.591131	0.034236
OLA1	50.4214	29.80826	0.591183	0.000323
RPS27	1877.647	1110.054	0.591194	0.020718
PPP1CC	94.70165	55.992	0.591246	0.125613
SETD7	14.07728	8.323327	0.59126	0.162733
ASB3	0.441415	0.261063	0.591422	0.115598
GCHFR	10.45791	6.185465	0.591463	0.064141
4-Sep	2.421281	1.43213	0.591476	0.055504
PTMA	367.833	217.6885	0.591813	0.046581
TBC1D20	14.93543	8.842407	0.592043	0.024516
ZNF800	1.324117	0.784016	0.592105	0.092218
GATA6	7.630387	4.518083	0.592117	0.008806
HOOK3	2.970686	1.759225	0.592195	0.019886
DIS3L2	5.624955	3.33119	0.592216	0.001927
BRPF1	9.683318	5.734937	0.592249	0.002423
FBXW11	13.47986	7.983844	0.592279	0.0017
ORC5	10.08053	5.972898	0.592519	0.182207
SUCO	9.65724	5.722794	0.592591	0.001621
NAA10	159.2785	94.39242	0.592625	0.001595
PAX1	0.010557	0.006257	0.592668	0.529863
MUTYH	7.215255	4.277884	0.592894	0.055674
ING3	4.801885	2.847244	0.592943	0.082905
TCF20	7.290817	4.323258	0.592973	0.001703

TOMM40L	8.763132	5.198601	0.593236	0.148054
NME2	0.82293	0.488288	0.593353	0.128589
SOCS4	9.025784	5.355893	0.593399	0.007027
ZNF174	3.855827	2.288107	0.593415	0.003484
ABHD17B	4.732124	2.808281	0.59345	0.019993
ALAS1	35.45232	21.04024	0.59348	0.026627
CAPN10	9.243917	5.486866	0.593565	0.010725
HS6ST1	7.238602	4.296897	0.593609	0.005383
LOC10798	0.352164	0.209177	0.593978	0.346695
DIXDC1	2.21398	1.315086	0.593992	0.018016
RTTN	3.045895	1.809409	0.594048	0.000423
MTERF4	4.051161	2.407094	0.594174	0.013778
USP22	53.98185	32.07486	0.594178	0.000266
THTPA	5.206622	3.094578	0.594354	0.113333
ACP1	92.68628	55.09904	0.594468	0.020287
RIPK4	6.132892	3.645882	0.59448	0.016482
GSR	24.96352	14.84297	0.594586	0.004238
LRGUK	0.064376	0.038281	0.594654	0.312302
FATE1	0.127505	0.075828	0.594705	0.718568
CCNI	120.7622	71.84547	0.594933	0.030024
LRRK2	0.548866	0.326562	0.594976	0.100636
RPL41	2575.093	1532.133	0.594982	0.003011
MTPAP	4.066652	2.420402	0.595183	0.022462
TAOK1	7.571176	4.507736	0.595381	0.020685
PTBP3	8.885067	5.290124	0.595395	0.006243
C17orf53	5.45008	3.245051	0.595413	0.006136
ROGDI	9.140565	5.442488	0.595421	0.006469
SLC26A6	12.00205	7.149938	0.595727	0.14166
SMARCE1	101.9747	60.75805	0.595815	0.002499
DPM3	48.51992	28.91194	0.595878	0.006332
PPP1R15A	71.12653	42.39299	0.596022	0.106736
HIST1H3B	1.747933	1.041991	0.596127	0.201396
ERMARD	6.184864	3.687281	0.596178	0.194638
CRLS1	10.01659	5.973675	0.596378	0.02304
KIF26B	0.151607	0.090456	0.596646	0.150268
TRAPPC11	7.577016	4.521	0.596673	0.00167
PLRG1	39.2514	23.42775	0.596864	0.024706
SF3A1	38.66832	23.08138	0.596907	0.008383
AADAT	4.651146	2.776605	0.596972	0.000691
SCLT1	2.681206	1.600686	0.597002	0.106702
HDAC1	90.65774	54.13487	0.597135	0.005148
PTGES3	110.639	66.07518	0.597214	0.004233
AMZ1	0.030445	0.018183	0.597242	0.295334
SIN3B	13.07651	7.810443	0.597288	0.052816
NDUFAF7	6.557439	3.917098	0.597352	0.003446
RITA1	31.80206	19.00171	0.597499	0.018199
UBA6	15.08386	9.013319	0.597547	0.028832
ZNF384	12.19159	7.285373	0.597574	0.019029
KIF4B	0.287757	0.171969	0.59762	0.026155
SRCAP	14.99507	8.961516	0.597631	0.014584
SUPT20H	18.79822	11.23715	0.597777	0.042925
EXOC1	11.96663	7.155837	0.597983	0.000963
TAF1C	9.328859	5.579155	0.598053	0.008185
APBB3	6.466424	3.86992	0.598464	0.147602
EI24	58.96022	35.28855	0.598515	0.041992
USP10	28.13679	16.8418	0.598568	0.001575
MEF2D	6.231495	3.73046	0.598646	0.00698
SLC38A1	21.68956	12.98448	0.598651	0.136499

ACADSB	3.332808	1.995202	0.598655	0.004648
IGIP	4.973079	2.977238	0.598671	0.007672
GOLPH3L	12.68263	7.594207	0.598788	0.027759
UGT2A1	0.016144	0.009667	0.598806	0.748028
LMBRD1	11.34596	6.79579	0.598961	0.002337
PRELID3B	53.89995	32.29647	0.599193	0.004579
PPIE	12.42061	7.44342	0.59928	0.001347
C18orf25	6.338409	3.798924	0.59935	0.02422
ARFGAP1	49.96355	29.94741	0.599385	0.256554
DHX40	29.51685	17.69267	0.599409	0.028235
TSPYL1	25.23531	15.12763	0.599463	0.010101
ALKBH2	23.4753	14.0775	0.599673	0.054141
FAM43B	1.725914	1.035035	0.599702	0.076849
SEC23A	26.60346	15.955	0.599734	0.021263
HSPA14	14.41279	8.64598	0.599882	0.020375
ZNF419	5.74661	3.44738	0.599898	0.101668
EFNA4	5.10534	3.062982	0.599957	0.028379
SUGT1	5.911905	3.548127	0.600166	0.049091
RSC1A1	1.82547	1.095719	0.600239	0.011392
COPS7B	12.21498	7.339988	0.600901	0.002211
C2orf69	6.834464	4.107541	0.601004	0.078662
MMP7	0.824425	0.495693	0.601259	0.302096
MARF1	9.296247	5.590211	0.601341	0.031728
LPCAT4	38.89045	23.38906	0.601409	0.202274
BUB3	83.33776	50.127	0.601492	0.000497
ATXN10	66.1478	39.78761	0.601495	0.024116
DDX51	7.587358	4.564274	0.601563	0.001382
ATP7A	1.846595	1.110929	0.60161	0.066265
C17orf80	8.150905	4.903706	0.601615	0.06722
FAR1	18.24941	10.97918	0.601618	0.028788
TCEA1	51.32214	30.8826	0.60174	0.039216
CASP7	8.540194	5.139496	0.601801	0.001009
METTL18	5.578926	3.358446	0.601988	0.06471
CHCHD6	2.443053	1.47081	0.602038	0.053004
ATIC	66.54171	40.06103	0.602044	0.079456
CYGB	0.110485	0.066531	0.602169	0.115611
RFXAP	4.836847	2.912949	0.602241	0.052668
PHC3	3.554113	2.140929	0.602381	0.086423
TOPBP1	24.11197	14.53112	0.602652	0.000976
VIRMA	13.59374	8.19248	0.602666	0.002905
NDUFB10	193.0469	116.3649	0.602781	0.01321
CDC42BP/	10.30004	6.208697	0.602784	0.064832
SEMA3A	1.5319	0.923498	0.602845	0.026787
FUCA2	41.48101	25.00797	0.602878	0.017964
TMEM238	0.083329	0.050241	0.602915	0.723135
VPS54	8.544735	5.152016	0.602946	0.001763
FAM200A	5.40388	3.25833	0.602961	0.098891
LOC10272	0.111938	0.067497	0.602989	0.418768
GALNT13	2.413487	1.455383	0.603021	0.164407
NENF	110.9188	66.88666	0.603023	0.036707
C12orf29	9.69709	5.848507	0.60312	0.011349
TOP2B	48.76088	29.41055	0.603159	0.111586
EPS15	14.01409	8.454992	0.603321	0.007015
IDE	7.38256	4.454338	0.60336	0.013265
EHMT2	15.11761	9.123161	0.603479	6.65E-05
PEX7	4.553643	2.748074	0.603489	0.291508
PRKD1	2.654862	1.602376	0.603563	0.006078
RAD51D	5.021784	3.031175	0.603605	0.119047

RPS13	1628.994	983.3388	0.603648	0.005508
NEK1	4.147172	2.503586	0.603685	0.129481
ZNF627	5.005132	3.022488	0.603878	0.033437
AP2B1	75.7605	45.75422	0.603932	0.010662
SF3B2	254.6644	153.805	0.603951	0.031631
ABCE1	41.02989	24.78046	0.603961	0.016298
IL17RE	0.309697	0.187053	0.603986	0.175442
ACAD8	3.987486	2.408415	0.603993	0.042768
ACACB	1.868623	1.128823	0.604094	0.039522
MORC2	12.43113	7.511432	0.604244	0.208284
GID4	6.464724	3.906417	0.604267	0.002717
CDC42SE1	31.71491	19.16511	0.604293	0.011466
MLF1	20.92476	12.64606	0.604359	0.146036
RFC4	73.73855	44.57192	0.604459	0.031498
CFH	1.18554	0.716696	0.604531	0.051372
PLCG1	33.07035	19.994	0.60459	0.013637
CPM	1.50833	0.911985	0.604632	0.046011
RBM4	10.22283	6.182227	0.604747	0.0104
ALG8	26.80148	16.21526	0.605013	0.155627
WDR38	0.263038	0.159337	0.605755	0.163717
SFMBT1	3.382529	2.049176	0.605812	0.012152
LYPD5	0.032272	0.019556	0.605987	0.564073
AAMDC	11.47941	6.958095	0.606137	0.046265
SMARCA4	38.56592	23.37812	0.606186	0.001692
USP6NL	1.876939	1.137778	0.606188	0.003792
CDH15	0.292997	0.177643	0.606299	0.383823
DCTN4	31.47303	19.08218	0.606303	0.020182
ZADH2	6.47736	3.929577	0.606663	0.003171
SETD4	3.774162	2.290044	0.606769	0.028245
MTHFS	0.076802	0.046607	0.606855	0.381006
ARL14	0.434347	0.263587	0.606858	0.490174
CEP192	3.810895	2.312707	0.606867	0.01305
DAGLA	1.930356	1.171566	0.606917	0.01859
CNBP	156.1313	94.77619	0.607029	0.035045
TBC1D9	9.272821	5.62923	0.607068	0.002362
IQCC	6.457867	3.921303	0.607213	0.01944
RPS14	2210.167	1342.201	0.607285	0.013761
IPO8	14.80603	8.992941	0.607384	0.028855
IPO4	15.5405	9.439636	0.607422	0.110502
ZNF609	7.734712	4.698913	0.60751	0.078536
TEX9	1.820469	1.106014	0.607543	0.064506
GNL1	17.46835	10.61288	0.607549	0.007364
TWIST2	5.350651	3.25099	0.607588	0.046419
FKBP3	46.2788	28.11964	0.607614	0.014281
TERF2IP	75.22398	45.72085	0.607796	0.036233
TTLL11	0.469751	0.285553	0.607881	0.177212
ZC3H15	85.09926	51.73199	0.607902	0.002365
GPRC5C	1.571426	0.955438	0.608007	0.056463
RPL7A	3046.334	1852.317	0.608048	0.015557
KLHL18	8.264933	5.027584	0.608303	0.005352
CSRNP1	16.12695	9.813406	0.60851	0.064747
ACSF2	17.47729	10.63574	0.608546	0.017283
ST20	3.708701	2.257256	0.608638	0.084347
MTMR6	5.665759	3.451045	0.609105	0.056228
SLC6A15	8.05607	4.907207	0.609132	0.074141
LRSAM1	8.656135	5.272968	0.60916	0.026509
NCKIPSD	13.81538	8.416155	0.609187	0.019677
TMEM164	2.48368	1.51329	0.609293	0.004138

KEL	0.070367	0.042883	0.609426	0.461599
PAPD4	8.691966	5.298958	0.609639	0.000264
FARSB	37.94437	23.1325	0.609642	0.044582
MFSD3	10.46657	6.383726	0.609916	0.08984
SRF	19.74752	12.04612	0.610007	0.015133
RCAN3	2.650577	1.617388	0.610202	0.000348
FBLIM1	4.345931	2.652602	0.610364	0.016346
PIGC	23.19511	14.16522	0.610699	0.055127
DHRS13	11.52519	7.039679	0.610808	0.122962
SCRIB	21.72417	13.27172	0.61092	0.006876
RAP2C	10.77641	6.584982	0.611056	0.000184
PAXIP1	8.528898	5.212059	0.611106	0.007362
TMEM128	13.4657	8.229297	0.61113	0.031347
GPHA2	0.070891	0.043325	0.611157	0.490024
BCL2L13	17.13048	10.4708	0.611238	0.036607
THAP3	6.610192	4.04056	0.611262	0.063761
RBFOX2	17.77814	10.86895	0.611366	0.033764
ANKRD61	0.170319	0.104135	0.611409	0.254423
TMEM18	6.349715	3.883526	0.611606	0.005578
RAB40C	7.057843	4.317148	0.611681	0.003977
EXOSC8	79.23917	48.48705	0.611908	0.016433
PSD2	0.071366	0.043671	0.611928	0.269709
AARS2	9.138491	5.593878	0.612123	0.074965
RNF5	57.97077	35.48897	0.612187	0.08754
RNF216	9.691488	5.933637	0.612252	0.016421
IVNS1ABP	14.90624	9.131513	0.612597	0.039175
CDPF1	6.18006	3.786318	0.612667	0.061535
GPALPP1	12.00495	7.355161	0.612678	0.00039
NPL	2.508434	1.537082	0.612765	0.184548
QTRT2	10.57495	6.483901	0.613138	0.084494
CLASP1	5.867846	3.598114	0.613192	0.057052
TADA2B	4.486719	2.751722	0.613304	0.004079
ICA1L	0.944367	0.579216	0.613338	0.075933
IER5	52.57471	32.24941	0.613402	0.015166
THG1L	7.567522	4.642501	0.613477	0.109072
PFDN6	117.2751	71.9491	0.613507	0.001111
MAPK11	8.329037	5.110068	0.613524	0.014239
GEMIN5	10.1208	6.21236	0.613821	0.03004
MAGI1	2.683274	1.647106	0.613842	0.003508
ZFR	30.50599	18.73247	0.614059	0.009884
DLG4	8.335532	5.11862	0.614072	0.108498
CHD4	48.35087	29.70029	0.614266	5.40E-05
RNMT	10.24733	6.296305	0.614434	0.010167
CBFA2T2	4.395949	2.701695	0.614587	0.05869
CLK4	11.72958	7.210683	0.614744	0.063445
FDXR	7.906789	4.86284	0.615021	0.0281
PAFAH1B2	14.6256	8.997493	0.615188	0.005023
DCTN2	86.23593	53.05315	0.615209	0.001172
MTFR2	2.903712	1.786637	0.615294	0.039365
FSTL4	0.169288	0.104185	0.615429	0.133915
BMPR1A	4.024426	2.47684	0.615452	0.01253
KCNG1	4.433	2.728354	0.615464	0.025218
CDK10	18.10756	11.14564	0.615524	0.007006
BRI3BP	5.327021	3.279171	0.615573	0.038261
GTPBP8	17.16029	10.56528	0.615682	0.109974
PANK1	1.169508	0.720094	0.615724	0.054164
CNBD2	0.336273	0.207096	0.615857	0.10432
SEPHS2	49.12265	30.25493	0.615906	0.136268

DBR1	9.792352	6.031691	0.615959	0.000471
NHEJ1	6.938748	4.273999	0.615961	0.001711
CEP70	7.662601	4.720152	0.615999	0.015733
MAVS	9.171582	5.650193	0.616054	0.013347
CRTC2	26.03729	16.05021	0.616431	0.054641
SRSF5	141.8231	87.42724	0.616453	0.075193
ZNF2	1.976473	1.218516	0.61651	0.007912
TMC7	1.742964	1.074804	0.616653	0.000886
ASB13	9.470245	5.841133	0.616788	0.085196
SNRPB2	57.89559	35.71578	0.6169	0.006901
PPP5C	49.99172	30.84691	0.61704	0.004206
SEC24A	7.018016	4.330785	0.617095	0.089333
NTNG2	0.902782	0.557185	0.617186	0.009818
GOT2	90.10785	55.61484	0.617203	0.000443
PPP2R2A	22.18665	13.69524	0.617274	0.005363
PARP6	19.51238	12.04515	0.617308	0.105789
RRP15	3.815513	2.355931	0.617461	0.053199
STK32C	5.413295	3.342648	0.617489	0.057305
MARK4	12.59398	7.776717	0.617495	0.038611
SLC35A3	4.609099	2.84727	0.61775	0.043427
PRKAR2A	18.88867	11.66919	0.617788	0.018264
CCDC65	0.102285	0.063194	0.617823	0.428273
ANKMY1	1.078976	0.666849	0.618039	0.003143
MGARP	13.76825	8.509647	0.618063	0.079444
THBS4	2.456263	1.518585	0.61825	0.053383
THAP2	0.94246	0.58268	0.618254	0.177117
HBP1	16.24497	10.04569	0.618388	0.03841
IGBP1	50.31351	31.11624	0.618447	0.043796
ZBTB21	5.458088	3.375788	0.618493	0.024411
SPINDOC	22.78936	14.1042	0.618894	0.014248
PAQR4	22.33393	13.82239	0.618897	0.014777
RTRAF	206.7226	127.9725	0.619054	0.013558
FLYWCH2	21.03399	13.02242	0.619113	0.003682
MTCH1	62.9537	38.99079	0.619357	0.056326
RPL18A	2012.194	1246.304	0.619376	0.010951
CENPF	20.00869	12.3929	0.619376	0.140916
RPS9	860.1414	532.8287	0.619466	0.059507
FANCG	29.38312	18.20257	0.619491	5.25E-05
ZNF398	5.246806	3.252882	0.619974	0.012434
PUS7L	2.726549	1.69064	0.620066	0.079494
RPL27A	398.8537	247.3391	0.620125	0.008968
ARHGAP2	3.272243	2.029815	0.620313	0.093101
TMEM181	7.586509	4.706295	0.62035	0.003973
PRDM5	1.149643	0.713705	0.620806	0.26576
GZF1	3.197402	1.985453	0.620958	0.110216
TTC7B	13.90369	8.633806	0.620972	0.035773
OSBP	22.7779	14.14707	0.621087	0.002818
SGSH	7.646955	4.750269	0.621197	0.000822
TRAF7	28.09769	17.45487	0.621221	0.006512
LIME1	4.622148	2.872982	0.621569	0.207191
NARFL	12.69668	7.892888	0.62165	0.017496
GPBP1L1	26.82622	16.68036	0.621793	0.001719
CEP104	5.276095	3.280667	0.621798	0.000396
PJA2	28.21621	17.54842	0.621927	0.025086
DNAAF5	16.68176	10.37556	0.62197	0.04076
AK2	81.74041	50.84401	0.622018	0.012068
CENPO	14.98944	9.325559	0.622142	5.50E-05
HECTD2	1.475358	0.917883	0.622143	0.000753

TRPM2	0.080487	0.050083	0.62224	0.312871
RPRD1B	11.95267	7.439175	0.622386	0.02174
TARS2	28.7023	17.86827	0.622538	0.017599
ZC3H10	4.207365	2.620717	0.622888	0.102731
BSDC1	26.45609	16.4838	0.623063	0.024243
A4GALT	2.154704	1.342561	0.623084	0.099403
SLITRK5	5.501207	3.427847	0.623108	0.062919
UBE2D1	8.533554	5.321648	0.623615	0.006871
DAAM1	3.74875	2.337863	0.623638	0.003478
INTS14	25.06242	15.63074	0.623672	0.140667
AEN	23.01929	14.35898	0.62378	0.017406
CYTH2	11.84151	7.387115	0.623832	0.004034
FZD7	5.759428	3.594497	0.624107	0.029189
ZNF586	4.267442	2.663464	0.624136	0.016865
CDCA5	42.44846	26.49599	0.624192	0.003904
ZFP36L2	8.502645	5.307833	0.624257	0.008105
JRKL	2.630256	1.642258	0.624372	0.112825
AGBL5	25.87854	16.15814	0.624384	0.015793
UBOX5	2.291619	1.431268	0.624566	0.005873
CARD9	0.187146	0.116904	0.62467	0.233048
RBMX	108.664	67.88025	0.62468	0.08947
MED22	21.18621	13.23541	0.624719	0.007904
TSPYL4	13.30307	8.312084	0.624825	0.002659
GMEB1	6.469295	4.04332	0.625002	0.028276
FAM241A	7.215642	4.510478	0.625097	0.031866
TRMT44	1.850934	1.157026	0.625104	0.014429
MSL1	20.67105	12.93131	0.625576	0.014499
CACUL1	9.894386	6.189812	0.625588	0.000539
CPS1	6.143467	3.843445	0.625615	0.132506
DHX8	14.24205	8.910178	0.625625	0.009209
HERC1	4.038429	2.526715	0.625668	0.017384
TMEM50B	9.2946	5.815622	0.625699	0.006721
RANBP3L	0.086255	0.053977	0.625785	0.585797
PRR4	4.207549	2.633842	0.62598	0.235303
TAF15	205.0719	128.3999	0.626122	0.090931
EPHX3	0.038252	0.023952	0.626174	0.742523
DTD1	20.23599	12.67798	0.626506	0.029197
ZNF699	0.954271	0.598022	0.62668	0.040804
ATP23	7.750945	4.859122	0.626907	0.069456
PCM1	11.22142	7.03598	0.627014	0.025924
TRAPPC4	41.89303	26.26751	0.627014	0.001735
GPR137C	1.669541	1.047194	0.627235	0.227093
PICK1	13.74282	8.621773	0.627366	0.00598
ASPM	9.353132	5.87039	0.627639	0.192687
CDC5L	21.88775	13.74219	0.627848	0.00058
SWSAP1	1.143632	0.718148	0.627954	0.2203
ACTBL2	0.093712	0.058865	0.628152	0.575254
RNF166	4.041157	2.538622	0.628192	0.008941
KIAA1549	1.971249	1.238551	0.628308	0.016893
TMEM117	1.199808	0.753851	0.628309	0.204946
ZFYVE27	8.110168	5.096531	0.628412	0.053022
TBL1X	13.61975	8.561615	0.628618	0.128834
CACNB1	1.578988	0.992646	0.62866	0.010465
UVSSA	0.929472	0.584457	0.628805	0.011722
ATP5MPL	208.3849	131.0536	0.628902	0.000779
CASP10	1.929831	1.213897	0.629017	0.042132
SPRY3	0.014263	0.008971	0.629017	0.60319
CCNA2	44.02072	27.69874	0.62922	0.009457

HMGCS1	11.50305	7.241052	0.62949	0.347689
DDX46	27.1187	17.071	0.629492	0.000598
ACCS	2.714324	1.708687	0.629507	0.266787
ITGB1BP2	0.405686	0.255401	0.629552	0.226658
KCNK13	0.102861	0.064765	0.629632	0.567882
VPS72	64.08384	40.35344	0.629698	0.00096
GFPT1	14.12651	8.895592	0.629709	0.08173
ZNF275	4.38112	2.759311	0.629819	0.05727
MAP4K5	6.868662	4.326145	0.629838	0.018091
PNKP	31.50556	19.84774	0.629976	0.003744
MIGA1	2.666223	1.680082	0.630136	0.002154
PIP4P1	18.42885	11.61291	0.630149	0.02867
SLC6A4	0.019889	0.012535	0.630254	0.476457
LARP4	7.346926	4.63052	0.630266	0.027301
ZNF222	2.149593	1.355027	0.630364	0.005559
XPO1	50.63708	31.92269	0.630421	0.033951
ZNF473	5.310861	3.348197	0.630443	0.102871
RBM48	1.744544	1.0999	0.63048	0.025359
GNPAT	40.05239	25.25493	0.630547	0.006235
HIST1H4J	0.216654	0.136617	0.630578	0.65327
QRICH1	27.32411	17.23527	0.630772	0.012669
AFAP1	4.901313	3.092098	0.630871	0.046673
ABL2	6.429899	4.056519	0.630884	0.009617
RIT1	10.59076	6.681798	0.630908	0.01115
CRYL1	9.222848	5.821089	0.63116	0.015479
CEP57L1	5.09975	3.218844	0.631177	0.01462
MRPL28	87.21492	55.05181	0.63122	0.013628
ZNF284	0.947071	0.597848	0.63126	0.000219
C12orf76	6.773856	4.277029	0.631402	0.007624
ZNF572	0.365362	0.230722	0.631488	0.378918
SLC45A2	0.024099	0.015232	0.63207	0.671796
ICAM5	13.64991	8.628334	0.632117	0.171446
RPL37	937.7925	592.9222	0.632253	0.009845
NQO2	15.39459	9.733578	0.632273	0.03603
VPS16	8.13419	5.143214	0.632296	0.007591
HIST2H3D	0.1683	0.106424	0.632348	0.655871
QARS	112.9006	71.40879	0.632493	0.0269
KCTD5	18.20797	11.51701	0.632525	0.007515
YY2	2.283094	1.444219	0.632571	0.054332
ZNF549	1.349444	0.854103	0.632929	0.059121
ARL15	6.683148	4.23	0.632935	0.045423
ARMT1	18.70628	11.84155	0.633025	0.062166
TPMT	15.72477	9.955922	0.633136	0.000144
RPS23	579.775	367.1182	0.633208	0.003572
METTL16	10.14665	6.426968	0.633408	0.01975
UBA1	78.78092	49.9042	0.633455	0.005383
THY1	0.019124	0.012116	0.633517	0.655791
LOC10798	0.37717	0.238982	0.633617	0.556179
PUM2	6.617756	4.193193	0.633628	0.00238
TOB2	6.710444	4.25411	0.633954	0.010268
CARNMT1	3.237884	2.052874	0.634017	0.002023
OXLD1	13.57461	8.610189	0.634286	0.075123
RFT1	5.684587	3.607718	0.634649	0.001746
EPB41L2	33.0682	20.9877	0.634679	0.002406
GNG5	189.2964	120.2188	0.635082	0.003291
ANKHD1	0.8955	0.568762	0.635134	0.00069
NUTM2E	0.158629	0.100762	0.635207	0.054364
TRIM45	2.190217	1.391254	0.635213	0.022774

CEP112	1.521423	0.96645	0.635228	0.142113
PREB	44.18822	28.07981	0.635459	0.003162
FBN2	7.691449	4.888759	0.63561	0.148552
TMEM209	16.64799	10.58209	0.635637	0.061884
FAM50A	204.2768	129.8468	0.635641	0.014485
APEX1	112.9756	71.84344	0.63592	0.071005
PIGA	5.304696	3.373937	0.636028	0.009174
SYN1	0.392205	0.24948	0.636096	0.202053
PRPF19	134.8874	85.81082	0.636166	0.067177
PRPSAP2	16.66237	10.60081	0.636212	0.088361
ZNF780A	1.835071	1.167722	0.636336	0.063129
NOMO3	4.736468	3.014348	0.636413	0.186646
AWAT2	0.035774	0.02277	0.636498	0.660325
ZYG11B	5.942225	3.782352	0.636521	0.001335
SSR1	26.5427	16.89557	0.636543	0.000845
TOR1AIP2	4.945956	3.148808	0.636643	0.000237
RAB38	0.181457	0.115542	0.63675	0.421408
FBXL19	20.82419	13.27306	0.637387	0.023692
CXorf56	19.86012	12.66176	0.637547	0.008213
PICALM	30.168	19.23472	0.637587	0.037347
HSPA13	24.41279	15.57059	0.637804	0.014908
NEFH	0.020485	0.013071	0.63806	0.590348
SRSF10	14.03141	8.956581	0.638324	0.00084
PPY	0.203336	0.129824	0.638471	0.60033
FABP5	26.96202	17.2149	0.638487	0.25469
RPLP1	3528.054	2252.639	0.638493	0.009989
FAM49B	13.33489	8.521246	0.639019	0.014757
MTHFD1L	8.697304	5.557823	0.639028	0.04758
IL1A	3.150501	2.013879	0.639225	0.311183
ARHGEF1	4.496788	2.874905	0.639324	0.055154
ACBD6	50.78036	32.46771	0.639375	0.13419
RAD51	11.86102	7.585171	0.639504	0.000132
DENND5B	2.036925	1.302786	0.639585	0.023101
CLUAP1	7.318614	4.681819	0.639714	0.04164
POLR2D	26.60602	17.03261	0.640179	0.016773
SLC44A2	24.00943	15.37479	0.640365	0.019755
PSORS1C1	0.228411	0.146267	0.640368	0.375661
BDH2	14.61524	9.360269	0.640446	0.034746
NDUFA7	184.2738	118.0503	0.640624	0.001835
PFDN2	234.3559	150.197	0.640893	6.22E-05
SLC29A3	1.305352	0.83671	0.640984	0.220418
KIF22	73.5876	47.17112	0.64102	0.002231
ZFP92	0.011271	0.007225	0.641034	0.593307
GABRR2	0.049857	0.031961	0.641052	0.349888
RABGAP1	12.55633	8.050616	0.64116	0.027427
KIAA0930	16.18112	10.37863	0.641404	0.040732
AQP11	0.414936	0.266173	0.64148	0.283944
WNK3	0.748003	0.479836	0.641489	0.009154
UHRF1BP1	3.825722	2.454394	0.641551	0.024591
TAL2	0.115538	0.07414	0.641695	0.594751
PIGS	48.32948	31.01285	0.641696	0.015647
CLINT1	26.79284	17.19753	0.64187	0.007594
TMEM41B	9.469656	6.080319	0.642084	0.06985
LOC10192	0.033402	0.02145	0.64218	0.517085
KPNA7	0.037992	0.024407	0.642422	0.612435
NTN5	0.142593	0.091647	0.642719	0.399109
LRRC63	0.174839	0.112375	0.642733	0.358147
RBBP6	12.14477	7.806245	0.642766	0.008955

ZCCHC7	4.241017	2.726409	0.642867	0.048538
SCD	80.75227	51.94408	0.643252	0.159091
PXYLP1	4.354008	2.801484	0.643426	0.024114
NME6	3.52553	2.26913	0.643628	0.08245
FAM217B	3.78206	2.436134	0.644129	0.010473
ZNF7	4.639163	2.988543	0.644199	0.01843
ATRN	8.004933	5.15816	0.644373	0.013628
SPIRE2	2.731904	1.760466	0.64441	0.023134
MLXIP	9.435842	6.080682	0.644424	0.000349
PPP3CA	8.807509	5.675855	0.644434	0.213262
SIRT4	0.743184	0.479194	0.644785	0.068038
ZBTB6	4.276576	2.757747	0.644849	0.125617
NOP9	8.956243	5.775797	0.644891	0.028306
WAS	0.471543	0.304119	0.644944	0.388309
SLC25A30	6.083797	3.925756	0.645281	0.029473
HOMER1	3.273848	2.11367	0.645622	0.024102
ARHGAP2	1.449772	0.936196	0.645754	0.017644
SFT2D1	55.83996	36.08168	0.646162	0.003924
PIGP	18.66584	12.06228	0.646222	0.128098
ERCC3	26.25385	16.96736	0.646281	0.02833
CLPX	12.42205	8.030631	0.646482	0.01216
NDC1	24.38965	15.76887	0.646539	0.037391
OTUD4	6.57899	4.25395	0.646596	0.010783
TTLL12	21.97481	14.20889	0.646599	0.002872
INPP5E	8.221988	5.319127	0.646939	0.007318
SRSF4	79.27795	51.32089	0.647354	0.003591
ST13	167.6361	108.5404	0.647476	0.024332
PWWP2B	4.772058	3.090272	0.647576	0.021255
EPO	0.056469	0.036568	0.647578	0.471092
VPS52	11.34985	7.350204	0.647603	0.135201
IST1	41.67341	26.99343	0.647737	0.015623
METAP1D	2.043935	1.324127	0.647832	0.203916
LOC10798	0.211633	0.137111	0.647869	0.352696
NIPAL2	0.592834	0.384096	0.647899	0.37414
IDH3B	51.26774	33.21751	0.647922	0.01593
TRAIP	14.3733	9.314207	0.648021	0.003514
THOC5	28.32607	18.35845	0.648112	0.026732
CYP1A1	1.438182	0.932277	0.648233	0.551469
CCDC110	0.504864	0.327357	0.648406	0.448268
PEAK1	3.3018	2.141178	0.648488	0.040363
HPSE	2.669989	1.731992	0.648689	0.048092
FCGBP	0.126319	0.081985	0.649028	0.380737
TMEM38B	13.02139	8.454679	0.649291	0.059195
PCMT1	83.06539	53.9413	0.649384	0.002296
EMC9	12.48693	8.114324	0.649825	0.018936
ZNF22	23.46191	15.24626	0.64983	0.207177
CLK3	10.42865	6.778108	0.64995	0.085558
CDK12	5.163447	3.356069	0.649967	0.027202
WDR47	7.357111	4.783889	0.65024	0.02425
SELENOK	71.58495	46.55269	0.650314	0.000502
RYBP	13.14207	8.547181	0.650368	0.001799
CATSPERZ	0.301388	0.196107	0.65068	0.632547
UIMC1	18.5936	12.10008	0.650766	0.048572
CCDC178	0.049659	0.03232	0.650841	0.454219
COX6A1	660.0833	429.7775	0.651096	0.003898
TNFAIP1	21.97534	14.3082	0.651103	0.1262
BBX	9.428562	6.140677	0.651285	0.006204
SCAP	29.26582	19.06183	0.651334	0.021913

DMXL1	2.475746	1.612571	0.651348	0.052327
LOC10050	1.595454	1.039291	0.651408	0.266062
C22orf23	0.994285	0.647749	0.651472	0.21627
TNPO1	22.98288	14.97282	0.651477	0.042684
BCR	12.60678	8.216081	0.651719	0.017306
KAT6B	2.229875	1.453552	0.651854	0.038709
TUBGCP4	7.658036	4.992092	0.651876	0.026087
MCMDC2	0.043638	0.028457	0.652102	0.57924
TRAPPC13	13.27036	8.655963	0.652278	0.0333
LOC10537	0.050227	0.032772	0.652471	0.474389
STAG3	0.301301	0.19659	0.652472	0.141939
ZNF443	1.335196	0.871225	0.652507	0.101178
SGO1	6.749927	4.405553	0.652682	0.026137
MRPS5	29.64213	19.35067	0.65281	0.011264
SAFB	52.96967	34.57968	0.65282	0.01399
RPSAP58	35.62571	23.25816	0.652848	0.013087
CMC4	17.61841	11.50452	0.652983	0.025635
LSM14A	25.38245	16.57441	0.652987	0.00092
TTC26	7.682188	5.017305	0.653109	0.149115
RELT	6.677138	4.361203	0.653155	0.08198
CA13	1.447783	0.94565	0.653171	0.020564
VGLL2	0.251785	0.164522	0.653423	0.526738
ELMOD2	5.513971	3.603266	0.653479	0.00905
UAP1	33.1639	21.67329	0.65352	0.014818
ETFA	151.0742	98.78354	0.653874	0.043499
HIST1H2A	0.320733	0.209825	0.654204	0.564604
CAMSAP1	6.025066	3.94208	0.65428	0.002127
C14orf119	34.04481	22.28194	0.654489	0.000242
FANCL	31.80534	20.8178	0.654538	0.005887
MAPK9	16.05805	10.51199	0.654624	0.00646
TCF19	29.88141	19.56144	0.654636	0.004461
PMF1	16.38029	10.72503	0.654752	0.047517
LOC10798	0.120622	0.078988	0.654837	0.51028
TUT1	13.83054	9.058514	0.654965	0.016491
GLUD1	48.30433	31.63795	0.654971	0.000775
RPL39L	38.22227	25.0348	0.654979	0.029461
KIAA0232	5.459039	3.576013	0.655063	0.059338
POLR1C	11.82109	7.745005	0.655185	0.103003
NCL	423.4568	277.4584	0.655222	0.099058
METTL6	5.307418	3.477786	0.655269	0.077118
PLPPR5	0.01454	0.009531	0.655471	0.674789
LRBA	5.674857	3.720544	0.655619	0.025801
HDDC2	66.38068	43.52822	0.655736	0.134492
TMEM106	6.118953	4.012493	0.655748	0.017035
SLC29A1	23.32677	15.30295	0.656025	0.12561
C21orf58	3.946829	2.590438	0.656334	0.090767
AKIRIN2	42.5324	27.91872	0.656411	0.083412
DPP9-AS1	0.024232	0.015911	0.656616	0.763835
HDGFL1	0.033922	0.022276	0.656675	0.231517
TSPAN9	5.138717	3.374513	0.656684	0.011758
ATPAF1	30.69328	20.15614	0.656696	0.004112
ABCF1	37.72467	24.77413	0.656709	0.002825
FLOT2	53.29244	35.00441	0.656836	0.023503
ZFAND3	24.3612	16.00201	0.656865	0.049793
AJUBA	4.765815	3.130713	0.65691	0.173274
TMX1	14.45075	9.493427	0.656951	0.016683
CDKL5	0.959142	0.630187	0.657033	0.016258
BCS1L	8.230669	5.409345	0.657218	0.085559

USH2A	0.003699	0.002432	0.657436	0.672966
NOB1	59.32	39.00427	0.657523	0.197884
HADHA	128.4286	84.44972	0.657562	5.55E-05
DDN	0.126344	0.083087	0.657621	0.466418
MZT2A	28.15569	18.51604	0.65763	0.026101
NUDCD3	20.82861	13.69842	0.657673	0.015058
ZBED8	7.740368	5.090974	0.657717	0.042857
HAUS7	27.32636	17.97934	0.657949	0.170013
ASB7	5.39426	3.549908	0.65809	0.002814
PSMD10	72.9447	48.02974	0.65844	0.000692
INTS13	22.19519	14.61546	0.658497	0.306631
HS2ST1	8.143029	5.362318	0.658516	0.156139
PNLDC1	0.025063	0.016506	0.658567	0.223096
LRRC28	2.164172	1.425335	0.658605	0.030289
BRD4	11.26864	7.423857	0.658807	0.009717
FUK	3.22344	2.124501	0.659079	0.00607
SLC9B2	1.537452	1.013529	0.659227	0.364193
IL3RA	0.042105	0.02776	0.659307	0.676864
CMTM1	0.911088	0.600777	0.659407	0.067032
IFNGR2	20.74486	13.68588	0.659724	0.003972
LGMN	42.62203	28.12092	0.659774	0.031578
FGFR1OP	13.84403	9.136627	0.659969	0.010997
CCDC43	25.93195	17.12379	0.660336	0.00544
TMEM106	125.8469	83.10409	0.660359	0.007946
TNFAIP6	2.237804	1.477898	0.660423	0.399805
SYAP1	39.08747	25.81608	0.660469	0.000155
SUMF2	84.16881	55.60382	0.660623	0.006032
UQCRH	497.4574	328.6332	0.660626	0.003051
GOLM1	59.38357	39.23206	0.660655	0.010829
MAST3	3.242601	2.142371	0.660695	0.006978
RNF40	34.9526	23.0945	0.660738	0.045101
IRS2	3.338536	2.20634	0.660871	0.028736
HIKESHI	34.412	22.74238	0.660885	0.026691
USP46	3.753543	2.480961	0.660965	0.021332
HNRNPH2	0.093438	0.06176	0.660975	0.559198
BRF1	6.841751	4.524631	0.661326	0.007902
IFNAR2	10.87765	7.195543	0.661498	0.087555
MYOCD	0.019819	0.013111	0.661552	0.580498
TXLNA	25.35186	16.77212	0.661574	0.014274
GUSB	37.46357	24.78656	0.661618	0.032373
ZNF202	1.960486	1.297099	0.661621	0.030438
PAQR3	8.846123	5.852967	0.661642	0.105238
ARPIN	9.831177	6.505079	0.661679	0.344918
FMO5	0.934445	0.618512	0.661903	0.265028
ELAVL1	30.42138	20.1377	0.661959	0.000493
ZNF225	1.200372	0.794745	0.662082	0.136556
ADCK2	21.63684	14.32557	0.662092	0.102277
NIF3L1	20.15183	13.34265	0.662106	0.060789
SLC7A6OS	9.407698	6.229257	0.662145	0.00386
RNF213	5.791899	3.835556	0.662228	0.113948
TRIM58	0.058601	0.03882	0.662447	0.604132
HERC3	1.976321	1.310039	0.662867	0.048663
MINK1	16.34545	10.83492	0.662871	0.008443
CEP152	2.552433	1.692342	0.663031	0.048665
NUTM2A	0.093339	0.061889	0.663055	0.516637
VPS37D	2.898419	1.921904	0.663087	0.031806
SELENOF	113.5553	75.32431	0.663327	0.08819
TMEM74	0.796529	0.528401	0.663379	0.037584

ARSG	1.121026	0.743676	0.663389	0.166242
HELZ2	4.022242	2.669976	0.663803	0.075436
EEF1B2	491.1409	326.0772	0.663918	4.74E-05
FAM76A	3.600271	2.39032	0.663928	6.99E-05
MAFG	15.1747	10.07496	0.663931	0.308528
RWDD3	6.883521	4.571479	0.664119	0.038685
NOP2	26.76676	17.77681	0.664138	0.044362
ACY1	0.248812	0.165287	0.664303	0.476846
SAAL1	46.10564	30.63362	0.664422	0.055079
MAN2B1	33.67752	22.37859	0.664497	0.00609
RPLP0	3151.195	2094.46	0.664656	0.006266
ZMYM4	13.07624	8.691392	0.664671	0.065486
KRBA2	0.667942	0.443997	0.664723	0.235668
SOX12	14.30103	9.506233	0.664724	0.050596
IWS1	23.20401	15.42449	0.664734	0.002957
GPD1L	14.06473	9.353024	0.664999	0.060651
WDR36	8.196545	5.450748	0.665006	0.040462
FSCN2	0.248476	0.165305	0.665276	0.436448
MRPS34	118.3708	78.75218	0.6653	0.071627
ABHD17C	10.07441	6.703817	0.66543	0.006933
RAB28	19.2083	12.78247	0.665466	0.085324
TBC1D1	6.976748	4.642831	0.665472	0.026936
RPL15	281.1655	187.1812	0.665733	0.017794
SCRN3	5.799448	3.861306	0.665806	0.280001
MRPL44	21.5893	14.37774	0.665966	0.120088
CYP27B1	0.820455	0.54651	0.666107	0.124102
SMARCB1	68.68851	45.77655	0.666437	0.043631
RNF170	4.656038	3.103067	0.666461	0.230402
KBTBD3	0.600985	0.400587	0.666551	0.038177
C2orf76	2.281536	1.520769	0.666555	0.084572
STK3	3.625669	2.417818	0.666861	0.001964
FBXO15	0.081107	0.054088	0.666871	0.211098
SEC14L1	12.38908	8.263962	0.667036	0.006053
SLC26A2	7.086709	4.728666	0.667258	0.150434
LETM1	15.43099	10.29713	0.667302	0.086016
PRDX2	179.1119	119.529	0.667343	0.028795
FBXO28	10.12531	6.757058	0.667344	0.017016
TEFM	4.612322	3.078385	0.667426	0.072098
AP4M1	8.310058	5.550124	0.66788	0.028999
PPP1R3D	1.901365	1.269894	0.667886	0.147115
MKNK2	30.77596	20.5582	0.667995	0.215908
TRIM37	7.867616	5.257487	0.668244	0.011766
MEOX1	0.082963	0.055442	0.668273	0.565063
FAM222B	2.879374	1.924266	0.668293	0.01445
GPR162	1.137451	0.760277	0.668405	0.081219
CWF19L1	19.87963	13.29139	0.668593	0.005853
GMPR	3.450555	2.307146	0.66863	0.080776
KIN	2.829282	1.891921	0.668693	0.014098
PCDHA1	0.115031	0.076952	0.668967	0.275411
RNF208	6.299475	4.214226	0.66898	0.008213
PLXNB2	30.89184	20.67024	0.669117	0.000539
METTL2A	10.59344	7.089416	0.669227	0.022285
SF1	48.58058	32.51617	0.669325	0.017403
ZNF221	0.262921	0.176119	0.669858	0.237604
MEI1	0.126311	0.084627	0.669989	0.488491
HCFC1	12.05286	8.075541	0.67001	0.048168
RFX2	3.961304	2.654777	0.670178	0.015671
STK25	30.10372	20.18192	0.670413	0.010366

ATXN3	3.516465	2.358113	0.670592	0.011966
MBNL2	7.387326	4.954118	0.670624	0.029872
GCNA	0.645177	0.432876	0.670942	0.250714
TOX4	16.60323	11.14398	0.671194	0.002494
TNN	0.033288	0.022346	0.671312	0.393971
PGM2	8.532866	5.729477	0.67146	0.0534
BUB1	33.98666	22.82927	0.671713	0.0215
MSANTD4	7.088515	4.761487	0.671719	0.069915
EDARADD	2.868468	1.92681	0.671721	0.109708
SRSF1	75.46982	50.69959	0.671786	0.021386
ALS2	3.782272	2.540933	0.671801	0.000534
EHMT1	4.788536	3.217041	0.671821	0.001175
GPR89A	4.596548	3.088197	0.671851	0.071225
GOLGA4	22.29333	14.97892	0.671901	0.019323
DROSHA	13.85549	9.311118	0.672017	0.039135
MADD	12.88212	8.65708	0.672023	0.00533
ATP6V1E2	1.203665	0.808956	0.672077	0.334197
WDR41	19.48491	13.09577	0.672098	0.021221
TMEM187	6.996944	4.703757	0.672259	0.297211
RAPGEF6	2.449308	1.646688	0.672308	0.063126
DTNA	8.739646	5.876086	0.672348	0.014187
GBA2	17.53651	11.79408	0.672544	0.001374
SLC25A5	497.6781	334.7513	0.672626	0.060361
EDA2R	4.864245	3.272669	0.672801	0.025989
IAH1	33.33446	22.42864	0.672837	0.002935
OSBPL9	24.4817	16.47379	0.672902	0.003333
MRPS31	29.29272	19.71354	0.672984	0.055732
ABCC1	10.82248	7.284644	0.673103	0.038156
POLR1A	5.056933	3.403956	0.673127	0.156014
ZNF341	2.370097	1.595694	0.673261	0.476179
SBDS	78.25761	52.68968	0.673285	0.010257
KIAA0586	4.472319	3.013177	0.673739	0.031761
SS18L1	4.22066	2.844196	0.673875	0.011711
RAB39B	5.261355	3.545576	0.67389	0.051509
SLC41A1	13.02255	8.776514	0.673948	0.012542
NBPF26	0.626955	0.422646	0.674125	0.146154
BTF3L4	23.2271	15.65805	0.674128	0.026151
PA2G4	154.6668	104.2674	0.674142	0.038946
TUBGCP3	8.65014	5.831756	0.674181	0.009588
KIF18B	10.59436	7.143622	0.674285	0.000737
FNTA	37.17949	25.07503	0.674432	0.066947
RBM7	5.960269	4.021011	0.674636	0.077074
ZNF57	3.484912	2.351303	0.67471	0.023372
URB1	3.78263	2.552271	0.674735	0.011383
LRMDA	2.502776	1.689107	0.674893	0.350564
PIK3R4	10.25884	6.925067	0.675034	0.020973
MYNN	4.455445	3.007638	0.675048	0.001017
PDP1	10.42334	7.03673	0.675093	0.095756
XRCC6	443.6287	299.5986	0.675336	0.010144
RASA2	3.893125	2.629658	0.675462	0.056858
NASP	120.8125	81.63406	0.675709	0.01782
NR2F6	30.97931	20.93607	0.675808	0.030909
SLC5A2	0.025216	0.017042	0.675853	0.636787
MATN3	0.955796	0.646234	0.676121	0.197599
EDC4	17.12708	11.58071	0.676164	0.076652
TMEM192	6.559187	4.436726	0.676414	0.076829
RPL35	2770.37	1873.97	0.676433	0.016516
LOC11226	1.171069	0.792367	0.676619	0.380248

DIO1	0.039523	0.026747	0.676738	0.778217
CACNA2D	1.250888	0.846558	0.676766	0.222482
VCPIP1	2.267548	1.534645	0.676786	0.002255
CNOT3	27.50347	18.61643	0.676875	0.003473
LRRC66	0.100513	0.068038	0.676905	0.307792
CTDSP2	19.51696	13.21182	0.676941	0.033276
WDR19	5.722891	3.875152	0.677132	0.00333
STK32A	0.297631	0.201587	0.677304	0.392758
CRNKL1	9.932269	6.72893	0.677482	0.073515
FAM149B1	4.430727	3.001919	0.677523	0.068668
ZDHHC4	31.83433	21.57199	0.677633	0.007049
NDUFA1	410.7448	278.3725	0.677726	0.004483
DYRK3	3.944323	2.673565	0.677826	0.150975
SEMA3B	5.982704	4.055673	0.6779	0.234288
GRIP1	0.707113	0.47949	0.678095	0.214441
NADK2	7.381233	5.005242	0.678104	0.084241
ZNF280D	2.317054	1.571508	0.678235	0.015943
CDC40	8.388116	5.689372	0.678266	0.049148
PEF1	41.19047	27.94143	0.678347	0.001059
SNAPC5	11.02524	7.479803	0.678425	0.057527
MBD6	8.847306	6.00376	0.678598	0.02359
CLDN23	0.135083	0.091668	0.678609	0.415089
HMMR	40.50524	27.49055	0.678691	0.269909
CETN3	46.02602	31.24612	0.678879	0.124222
TBC1D14	8.694363	5.905326	0.679213	0.004704
XKR9	0.026534	0.018024	0.679288	0.494626
CBX6	21.30028	14.47031	0.679348	0.136839
CAMK1	31.59409	21.47854	0.679828	0.093181
NUDT21	43.63195	29.67139	0.680038	0.003565
FAM110B	0.581476	0.395481	0.680132	0.159304
FECH	4.558986	3.100744	0.680139	0.139086
CCNG2	5.393585	3.668421	0.680145	0.00212
ZNF215	2.584199	1.757907	0.680252	0.07905
TPTE	0.09633	0.065539	0.680359	0.390864
LOC10798	0.037606	0.025587	0.680403	0.712635
SERPINB1	13.03437	8.869968	0.680506	0.01708
GIP	0.710162	0.483314	0.680569	0.284064
C1orf162	0.806779	0.549131	0.680646	0.228424
TIMM17A	54.63452	37.20007	0.680889	0.026281
PRRC1	18.61567	12.67592	0.680927	0.095178
EPOP	5.790644	3.943187	0.680958	0.220796
PROSER3	5.069725	3.452538	0.681011	0.011839
EAPP	19.25529	13.11443	0.681082	0.027631
RHBDD2	29.50803	20.09956	0.681156	0.098211
PTGS1	0.096577	0.065804	0.681368	0.530136
TBL3	17.54379	11.95507	0.681442	0.001067
GRIPAP1	13.10128	8.930136	0.681623	0.082317
SLC25A34	0.092896	0.063321	0.681626	0.625307
KLHL20	3.937725	2.685549	0.682005	0.023004
METAP1	15.88954	10.83967	0.682189	0.007936
LOC10192	0.994759	0.678667	0.682243	0.459246
SEC23IP	9.722116	6.634129	0.682375	0.043428
LARS2	11.54126	7.878917	0.682674	0.09588
TRDMT1	0.892243	0.609271	0.682853	0.000207
RPAP3	14.94887	10.2094	0.682954	0.042166
RP9	16.00075	10.92802	0.682969	0.172579
KCTD6	7.231255	4.939402	0.683063	0.030868
GPT2	22.50003	15.36958	0.683092	0.010093

RNF19A	5.016872	3.427466	0.683188	0.022483
CTBP1	12.09443	8.263801	0.683273	0.000136
TMX4	10.61122	7.253156	0.683537	0.040316
SENP8	1.326749	0.906946	0.683585	0.277147
PWP1	30.07497	20.5607	0.683648	0.06548
C5orf34	4.561234	3.118303	0.683653	0.098575
SNN	5.703622	3.89975	0.683732	0.077342
FNBP1	5.96925	4.081411	0.683739	0.013032
C12orf60	0.848852	0.580393	0.683739	0.019804
SAP130	13.12477	8.976937	0.683969	0.022116
ANKRD27	13.9981	9.577001	0.684164	0.036563
BCL2L15	0.067623	0.046266	0.684171	0.329474
C1QTNF3	0.333483	0.228187	0.684255	0.162243
FAM136A	40.52078	27.72723	0.684272	0.027601
AIFM3	0.038584	0.026402	0.684274	0.788065
GTF2H2C	6.635656	4.541048	0.68434	0.156255
MAP3K10	10.33777	7.074658	0.68435	0.000889
PSMG4	2.981243	2.040348	0.684395	0.201483
EXOSC7	31.23008	21.3761	0.684472	0.018716
CYP2R1	1.403058	0.960587	0.684638	0.209283
PDCL	8.811899	6.033233	0.684669	0.013282
GPR89B	2.182583	1.494426	0.684705	0.265163
ACTR2	73.7891	50.53868	0.684907	0.026381
MIEF2	5.386076	3.689874	0.685076	0.024442
TIMM23B	4.571207	3.132145	0.68519	0.046281
PGF	7.510315	5.146648	0.685277	0.378185
VBP1	59.03139	40.46737	0.685523	0.071953
ERICH2	2.16709	1.486148	0.685781	0.106581
KCNK6	0.063826	0.043793	0.686131	0.044691
NLGN2	27.46382	18.84811	0.686289	0.034705
AMBRA1	5.998949	4.117107	0.686305	0.029827
PLAA	9.05266	6.214365	0.686468	0.010293
APPL1	15.32608	10.52095	0.686474	0.003819
ZBTB40	3.487544	2.394484	0.686582	0.234235
N4BP1	7.60047	5.219403	0.686721	0.002747
PXK	9.76375	6.706172	0.686844	0.129843
ERCC6L	9.403151	6.459943	0.686998	0.030477
PPP6C	29.17003	20.03983	0.687001	0.007952
CAVIN4	1.408491	0.96768	0.687033	0.199667
RRM2B	8.057027	5.535495	0.687039	0.13121
UBP1	29.79124	20.4694	0.687095	0.017111
KIAA0319l	13.52479	9.30096	0.687697	0.013029
SETD2	10.68975	7.352306	0.68779	0.129055
HDHD5	21.50318	14.79315	0.687951	0.182851
ACADS	10.29261	7.082214	0.688087	0.161317
ADSS	19.26948	13.26261	0.688271	0.136767
WDR76	16.04308	11.04879	0.688695	0.024454
ARFGEF1	6.181344	4.257573	0.688778	0.066587
FBXO16	5.207012	3.588499	0.689167	0.038659
7-Mar	20.75845	14.30711	0.689218	0.005627
WDR62	12.11607	8.352448	0.689369	0.07816
SLC9A6	6.71464	4.629381	0.689446	0.011579
UNKL	3.16789	2.184242	0.689494	0.010367
EIF3C	11.80707	8.141719	0.689563	0.059271
MCU	10.04075	6.92451	0.689641	0.007072
SREK1IP1	6.319306	4.358336	0.689686	0.030148
DISP1	2.347339	1.61899	0.689713	0.004296
ZNF276	2.23537	1.541778	0.689719	0.105346

ZFYVE28	0.507152	0.349855	0.689843	0.069779
AGPS	8.193647	5.652516	0.689866	0.010863
RANBP6	8.019861	5.533343	0.689955	0.318717
SPTLC2	8.842936	6.101312	0.689964	0.029962
U2AF1L4	15.80934	10.90859	0.69001	0.120016
SPTBN5	0.016291	0.011242	0.690086	0.720133
LIN7B	6.7873	4.683854	0.690091	0.278484
AUH	2.848219	1.965719	0.690157	0.007213
BCAM	10.50591	7.25279	0.690354	0.256435
POLR3D	21.77696	15.03515	0.690416	0.015896
CSNK2B	168.2208	116.1569	0.690503	0.009558
RGS2	17.91524	12.37344	0.690665	0.217108
ANO3	0.008463	0.005845	0.690678	0.738042
BBS2	24.74399	17.09161	0.690738	0.001966
RILPL1	7.68546	5.3089	0.690772	0.010312
HLA-DPB1	7.886761	5.44807	0.690787	0.223349
CHD1L	16.79903	11.60562	0.69085	0.106025
COX7A2	131.5975	90.92229	0.690912	0.010061
HSD17B14	7.880228	5.44656	0.691168	0.003787
FAM110A	2.905398	2.009742	0.691727	0.12756
ROCK2	5.436557	3.761224	0.691839	0.056246
CRNDE	17.44237	12.0675	0.691849	0.022381
CMBL	6.401506	4.429179	0.691896	0.095148
WAC	24.27431	16.79607	0.691928	0.00113
KCNAB2	2.584284	1.788342	0.692007	0.01502
BRICD5	1.498857	1.037481	0.692182	0.40561
MYL6B	93.63292	64.83786	0.692469	0.00893
SMIM8	5.837783	4.042515	0.692474	0.113848
DHX34	5.892768	4.081032	0.692549	0.052399
DNMBP	4.671167	3.235017	0.69255	0.0161
ZC3H13	9.276339	6.42446	0.692564	0.024209
COX18	4.575822	3.169643	0.692694	0.184336
PLEKHF2	3.752519	2.599474	0.692728	0.026621
SNRNP40	68.49553	47.4545	0.692812	0.03201
CIR1	32.04094	22.20868	0.693134	0.078454
LAMA5	10.31529	7.150815	0.693225	0.177807
LPCAT2	2.844094	1.971815	0.693302	0.050996
FBXL6	7.301206	5.062169	0.693333	0.044248
ANP32B	280.5382	194.5292	0.693414	0.010339
UQCRC2	122.3421	84.83804	0.693449	0.00201
LOC10537	0.04155	0.028816	0.693518	0.440049
GORASP2	52.47344	36.39354	0.693561	9.14E-05
FOXN2	2.437557	1.690604	0.693565	2.69E-05
CARMIL1	2.169975	1.505078	0.693593	0.114671
GATD1	15.81614	10.96997	0.693593	0.019413
BTBD2	64.4101	44.68549	0.693765	0.028803
PTPRD	1.159316	0.80453	0.693969	0.134837
FAM72B	3.590845	2.492301	0.694071	0.100453
NUDCD1	9.75133	6.771962	0.694465	0.073263
SIN3A	12.95069	8.994289	0.694503	0.000901
DYNC1LI1	38.74036	26.91704	0.694806	0.038967
FAM47E	0.248944	0.172971	0.694821	0.421285
TDP2	25.75893	17.8988	0.694858	0.201538
ABCA2	9.570655	6.650736	0.694909	0.018307
DNTTIP2	37.63904	26.16115	0.695054	0.071392
COPS4	39.03408	27.13088	0.695056	0.046088
AFF4	13.25395	9.213891	0.695181	0.076117
SLC16A1	43.43868	30.19996	0.695232	0.081761

PKP4	9.167255	6.374005	0.695301	0.137522
SMURF1	6.724107	4.67583	0.695383	0.085757
PPP1R1C	0.29964	0.208451	0.695673	0.577167
PRPSAP1	22.17037	15.43363	0.696138	0.016076
MATR3	63.79666	44.41357	0.696174	0.014627
AGAP3	29.62483	20.62706	0.696276	0.001903
C1orf43	153.6966	107.0157	0.696279	0.046192
PANK4	8.263832	5.754962	0.696404	0.008814
BAZ1B	27.26568	18.99392	0.696624	0.004276
LSP1	0.19938	0.138938	0.696849	0.432552
C15orf39	7.95312	5.542175	0.696855	0.02584
NCAPD2	53.25052	37.11261	0.696943	0.007944
ALDH9A1	43.91123	30.60481	0.69697	0.061207
UBR1	4.768243	3.324116	0.697137	0.013462
COX7A2L	82.57085	57.56919	0.69721	0.001171
YY1	28.01564	19.53702	0.697361	0.010008
NOP14	22.99455	16.04149	0.697621	0.217807
IMPA1	5.81777	4.05883	0.697661	0.024207
IFT80	10.06872	7.025372	0.697742	0.114366
RNF146	3.272933	2.28427	0.697928	0.041591
ZNF451	3.458884	2.414449	0.698043	0.007142
GRAMD1E	0.593018	0.41413	0.698342	0.002496
RLF	7.575382	5.290423	0.69837	0.035399
KDM2B	6.71346	4.688707	0.698404	0.012719
FOXRED1	11.76586	8.218354	0.698492	0.026868
YBEY	3.06715	2.142753	0.698614	0.101066
INTS1	16.21668	11.33103	0.698727	0.004453
HIGD2A	144.993	101.3323	0.698877	0.023634
CIAO1	27.18659	19.00162	0.698933	0.013934
PLPP6	5.833222	4.077187	0.69896	0.263913
TMEM185	7.160943	5.005382	0.698984	0.029079
DHRS11	12.5579	8.77822	0.69902	0.200462
ZMYM6	4.380094	3.061827	0.699032	0.031644
LOC10105	0.05179	0.036203	0.699038	0.690282
ATP6V1A	23.56337	16.47311	0.699098	0.043181
UBB	794.8068	555.6961	0.699159	0.019348
DLD	36.45883	25.49724	0.699343	0.139933
ABRAXAS2	11.52583	8.060887	0.699376	0.029663
RAPSN	0.065381	0.045728	0.699409	0.758835
CCDC151	4.258236	2.978391	0.699442	0.132892
LOC11226	0.074401	0.052058	0.699696	0.582257
TEX29	0.157663	0.110321	0.699729	0.48254
FRS2	2.607323	1.824549	0.699779	0.029914
YIPF6	9.556439	6.687751	0.699816	0.0031
AHI1	4.570436	3.198523	0.699829	0.023535
UBAP2	11.12667	7.788305	0.699967	0.013248
NRM	13.57815	9.504394	0.699977	0.052949
SNX4	18.20387	12.74346	0.700042	0.023335
CENPQ	25.69863	17.99359	0.700177	0.018095
C20orf27	30.54402	21.38897	0.700267	0.164145
PEX5L	0.007418	0.005195	0.700381	0.690405
TCP1	189.6075	132.8299	0.700552	0.046754
RPL6	687.3454	481.6282	0.700708	0.006316
LRP4	3.621992	2.538	0.700719	0.031482
STIMATE	6.922737	4.852458	0.700945	0.008116
EIF3M	207.4667	145.4528	0.70109	0.067484
SCRN1	36.0752	25.29637	0.701212	0.110932
HSPB1	310.6352	217.8246	0.701223	0.310494

RPF2	22.7575	15.96234	0.70141	0.148664
WISP2	0.08574	0.060143	0.701463	0.660166
ELP5	26.44817	18.55293	0.701483	0.009844
SLC25A16	3.106069	2.179235	0.701606	0.180764
DIS3L	4.177526	2.93138	0.701702	0.233616
KAZN	0.753553	0.528801	0.701744	0.052337
SRP68	54.65654	38.37392	0.702092	0.004745
DDX18	27.37377	19.22295	0.70224	0.110188
KIAA0391	9.908241	6.961218	0.702569	0.158845
CD99	14.31026	10.05536	0.702668	0.50872
ZNF394	6.242167	4.386193	0.702672	0.056399
FAM86B1	0.707868	0.497452	0.702747	0.435152
TTC32	8.846973	6.217696	0.702805	0.122617
MGAT5	12.58131	8.842205	0.702805	0.071076
LYSMD3	8.169412	5.743227	0.703016	0.004298
PSMF1	12.61905	8.873538	0.703186	0.001995
BNIP1	22.46907	15.80573	0.703444	0.036906
MTM1	1.566325	1.101969	0.703538	0.021012
CBX4	9.936816	6.992414	0.703688	0.02521
1-Sep	5.281736	3.716942	0.703735	0.204985
USO1	36.65016	25.79828	0.703906	0.00194
LAS1L	30.70727	21.61984	0.704063	0.054418
CCDC140	0.189263	0.133274	0.704171	0.689963
UBE2E2	2.31537	1.630521	0.704216	0.002723
CPT2	9.492324	6.685965	0.704355	0.172559
ITPKC	6.211461	4.377557	0.704755	0.077797
SLC50A1	37.29139	26.28214	0.704778	0.00383
PDCD7	14.12255	9.953276	0.704779	0.004324
AFF1	3.3649	2.371656	0.704822	0.013035
LZTR1	12.00297	8.460457	0.704864	0.136013
ACYP1	38.66172	27.27081	0.70537	0.058389
HDDC3	25.51283	17.99825	0.705459	0.411554
VPS13D	3.142118	2.216791	0.705508	0.132913
COX6C	161.8791	114.2184	0.705578	0.034229
USP38	2.842594	2.005684	0.705582	0.086123
AMACR	3.680456	2.597589	0.705779	0.239927
LIMCH1	1.560819	1.101807	0.705916	0.059803
IGF2BP2	12.87653	9.091903	0.706083	0.043433
PCDHA7	0.017683	0.012486	0.706127	0.625389
KCNJ9	0.010476	0.007399	0.706301	0.751488
SUPT5H	45.03604	31.81579	0.706452	0.059956
RPRD2	3.752655	2.651288	0.70651	0.095917
HS1BP3	3.914736	2.766057	0.706575	0.097316
RABIF	7.853279	5.549214	0.706611	0.011845
C6orf120	10.95403	7.740646	0.706648	0.325715
UBE2Q1	29.90837	21.14125	0.706868	0.020069
IFFO2	4.542446	3.210963	0.70688	0.221484
RCAN1	8.064361	5.701616	0.707014	0.331545
MREG	2.086989	1.475832	0.707159	0.072046
VAT1	152.3819	107.7661	0.707211	0.128501
F2RL1	6.45112	4.562875	0.7073	0.001465
MAP4K3	6.088507	4.307531	0.707486	0.008263
GUCY1B1	6.14841	4.350459	0.707575	0.097202
UTRN	2.529759	1.790321	0.707704	0.160809
CNOT11	29.03895	20.55169	0.707729	0.031814
PIK3R3	0.078358	0.055457	0.707732	0.528454
PDZD9	0.042198	0.029867	0.707777	0.625519
MXD4	11.03024	7.809302	0.70799	0.08905

PKD2L2	0.042739	0.030262	0.708055	0.721113
MRPS30	26.56843	18.81321	0.708104	0.143645
ASCC3	4.016758	2.845111	0.70831	0.141021
TEX46	0.364439	0.258171	0.708406	0.530197
CDH2	18.81002	13.32572	0.708437	0.062366
NFATC2IP	17.63622	12.50269	0.708921	0.001612
HMGB1	74.16774	52.5842	0.70899	0.010802
NAT6	9.425443	6.685455	0.709299	0.086801
SON	21.65586	15.36083	0.709315	0.030801
STOM	18.60546	13.19889	0.709409	0.053138
HIBADH	30.44508	21.60076	0.709499	0.109347
APRT	206.6674	146.6877	0.709777	0.073817
UBL3	6.770384	4.807313	0.71005	0.106349
SUPT3H	1.639315	1.164125	0.710129	0.046478
ARID1B	4.506649	3.202086	0.710525	0.043242
PPP1R3B	2.991317	2.125728	0.710633	0.288607
ZNF264	2.085348	1.481998	0.710672	0.040153
ZNF765-Z	0.081144	0.057671	0.71072	0.436648
ROPN1B	0.345553	0.245632	0.710836	0.090907
PRRG1	2.587322	1.839286	0.710884	0.084006
RNF31	7.212034	5.127543	0.710971	0.005959
TARS	137.2141	97.59005	0.711224	0.04374
VAMP4	6.335815	4.506404	0.711259	0.002294
RBM22	40.81627	29.03161	0.711275	0.003092
MIS12	8.395915	5.972123	0.711313	0.200237
RC3H2	7.501063	5.336947	0.711492	0.038466
SLC41A2	1.212777	0.862889	0.711498	0.041899
GOLGA3	14.32403	10.19229	0.711552	0.331994
KIF1B	5.140745	3.657953	0.711561	0.070505
ADNP	14.71038	10.46807	0.711611	0.023738
C11orf74	20.50515	14.59237	0.711644	0.252805
THOC1	12.69377	9.034962	0.711764	0.016923
DYRK1A	3.693148	2.629571	0.712014	0.019268
CHAC2	5.722163	4.074411	0.71204	0.188623
XAB2	35.24806	25.09926	0.712075	0.024689
AGL	4.055482	2.888526	0.712252	0.129802
USP53	4.762957	3.392488	0.712265	0.017317
RRN3	16.80257	11.96814	0.71228	0.038754
MCMBP	26.87542	19.14735	0.712448	0.028132
TCF3	19.10465	13.6115	0.712471	0.015838
COG8	10.71672	7.636031	0.712534	0.091363
FAHD2A	4.283373	3.052888	0.71273	0.00689
FBXO17	24.19053	17.24628	0.712935	0.054227
TCP11L1	8.823061	6.2919	0.71312	5.44E-05
GOSR1	8.609963	6.140898	0.713232	0.041662
HSPA4	102.609	73.1996	0.713383	0.047017
NUP35	10.85018	7.740777	0.713424	0.093991
SLC2A11	2.808189	2.003662	0.713507	0.134217
ICE2	4.95297	3.534573	0.713627	0.122994
FAM13B	4.257341	3.038474	0.713702	0.301957
SART3	19.27933	13.76099	0.713769	0.015076
MTIF3	7.975085	5.694303	0.714012	0.011661
PPP2R1B	3.257184	2.326137	0.714156	0.204053
DHRS7	37.29652	26.63857	0.714237	0.050895
KPNB1	111.0932	79.3896	0.714622	0.010749
OXR1	5.550308	3.967266	0.714783	0.0038
NARS	94.15555	67.32965	0.71509	0.027115
NFU1	26.6811	19.08385	0.715257	0.062368

SLC25A29	8.24317	5.896315	0.715297	0.037218
ANKFY1	8.344332	5.968794	0.715311	0.072012
SLC44A3	0.902454	0.64561	0.715394	0.248517
WDR73	8.965468	6.414216	0.715436	0.154642
SARNP	159.9833	114.4732	0.715532	0.013321
DUOX1	0.016208	0.011598	0.715564	0.666825
CCT8	174.3701	124.7976	0.715705	0.187178
UBQLN2	15.79331	11.3041	0.715753	0.029787
STX6	11.22007	8.031262	0.715795	0.009734
PARP8	2.240832	1.604024	0.715816	0.00479
DOCK9	2.864529	2.050825	0.715938	0.152056
ZNF70	1.645841	1.178388	0.715979	0.302688
RPA2	69.08172	49.47332	0.716157	0.12172
MCTS1	13.9205	9.969465	0.716171	0.063617
NBPF1	4.582394	3.281786	0.716173	0.029042
ZEB1	4.85754	3.479333	0.716275	0.033256
FBXO31	6.163928	4.41515	0.716288	0.000769
TMCC3	2.148049	1.53868	0.716315	0.037037
KIF6	0.105513	0.075608	0.716572	0.491196
DCAF4	4.577959	3.280445	0.716574	0.086034
FBXL18	3.799816	2.723246	0.716678	0.052114
CDKN2AIF	4.971729	3.56318	0.716688	0.005648
PDE12	5.976991	4.284826	0.716887	0.053741
ZC4H2	8.66855	6.216302	0.71711	0.073761
EIF2A	32.78752	23.52323	0.717445	0.013136
C2orf73	0.031102	0.022319	0.717627	0.712652
USP18	8.881383	6.373646	0.717641	0.229113
DGCR2	17.86834	12.82421	0.717706	0.071015
EIF2AK1	47.62505	34.18396	0.717773	0.001602
LPCAT1	21.56043	15.47639	0.717814	0.20504
SLC35G1	1.317126	0.945517	0.717864	0.192669
SPDYE18	0.092613	0.066488	0.717914	0.62825
LOC10272	0.115271	0.082769	0.71804	0.435406
NCAPH	13.39003	9.614748	0.718053	0.039294
SLC25A28	6.123782	4.398134	0.718205	0.037602
CCDC190	0.012349	0.008873	0.718532	0.795743
MSH3	6.733433	4.839418	0.718715	0.034436
NOC3L	11.10032	7.97867	0.718779	0.062686
KLHL7	7.526185	5.410049	0.71883	0.001238
RHNO1	22.89844	16.46021	0.718835	0.196355
SLBP	55.72489	40.06335	0.718949	0.081603
MAP3K7	17.67879	12.71302	0.719112	0.008999
IPMK	2.390822	1.719299	0.719125	0.003354
ZNF26	3.287557	2.36469	0.719285	0.106215
MXD3	11.15063	8.021013	0.719333	0.232478
MSRB2	14.5506	10.46676	0.719335	0.144076
BRD7	26.06705	18.75132	0.719349	0.004412
KATNAL1	2.997291	2.156272	0.719407	0.097831
USP32	7.095637	5.105065	0.719465	0.001595
MIB2	4.247002	3.055709	0.719498	0.209384
PEX5	7.638092	5.495811	0.719527	0.02764
GLOD4	27.78491	19.99245	0.719543	0.104969
RETREG3	13.11627	9.440377	0.719745	0.051748
SZT2	3.165861	2.27936	0.719981	0.23948
EXOSC6	14.10789	10.15805	0.720027	0.124538
NRF1	6.585608	4.744924	0.720499	0.00249
TAF4	5.666773	4.083062	0.720527	0.004454
WDR82	36.30231	26.15952	0.720602	0.065261

GJC1	8.333055	6.004909	0.720613	0.062303
HNRNPLL	12.78042	9.21138	0.720741	0.039038
PMPCA	29.19355	21.04238	0.720789	0.000227
CBLL1	3.320347	2.393516	0.720863	0.013965
FLOT1	86.5616	62.40088	0.720884	0.001593
ATG4D	18.41575	13.27654	0.720934	0.065552
ZNF444	7.76906	5.60178	0.721037	0.006614
TMEM33	10.29229	7.422854	0.721205	0.034237
EIF1AD	16.08056	11.59901	0.721306	0.083983
SSBP3	17.69955	12.76752	0.721347	0.134299
COPS3	68.64689	49.51993	0.721372	0.011773
PHF5A	68.88796	49.70773	0.721574	0.025064
MRFAP1L1	62.72999	45.27483	0.721741	0.199173
TMEM255	4.697803	3.39107	0.721842	0.104252
SPIN3	2.105944	1.520672	0.722086	0.040035
SLC38A9	4.874391	3.520042	0.72215	0.127702
BRF2	8.142194	5.882036	0.722414	0.073063
ETV7	0.046902	0.033884	0.722433	0.660639
C3orf38	14.73755	10.65039	0.722671	0.096631
NUMA1	19.44296	14.06415	0.723354	0.076619
DDB2	41.8065	30.24543	0.723462	0.105193
HSPD1	282.8123	204.6519	0.723632	0.214991
SH3GLB1	16.40217	11.87516	0.723999	0.00026
ENOPH1	28.15279	20.38291	0.72401	0.070292
CAND1	29.90067	21.65433	0.724209	0.006783
GMNC	0.118176	0.085646	0.724732	0.614941
FUT4	0.796456	0.577244	0.724766	0.357468
MAP3K3	7.265013	5.265759	0.724811	0.003627
UBTD2	13.67242	9.910592	0.72486	0.173581
NUDT2	38.80851	28.13608	0.724998	0.078713
SCO1	5.907779	4.284224	0.725184	0.177169
C17orf49	86.93811	63.04681	0.725192	0.043122
EXOG	2.87558	2.086412	0.725562	0.015122
FBXO45	7.677344	5.570476	0.725573	0.005645
ALG12	15.2185	11.04557	0.725799	0.002798
IFT81	12.34036	8.95689	0.725821	0.145301
AGPAT5	8.939938	6.489435	0.725893	0.008115
SLC25A26	4.382174	3.181126	0.725924	0.090594
COG5	6.159495	4.471955	0.726026	0.002559
PCGF2	13.11746	9.525095	0.726139	0.006606
COX10	7.273483	5.282567	0.726278	0.029499
PRKD2	7.721832	5.608525	0.72632	0.034553
R3HDM1	11.6754	8.480161	0.726327	0.05908
MEAF6	15.70995	11.41335	0.726505	0.003962
NUDT13	2.429803	1.765483	0.726595	0.314011
LOC10537	0.099952	0.072637	0.726722	0.802411
CDK3	2.098653	1.525248	0.726775	0.047236
SERGEF	19.86391	14.43668	0.72678	0.146422
RAPGEF2	3.086865	2.244267	0.727038	0.058711
H3F3B	210.8688	153.3116	0.727048	0.056336
FAM206A	15.58637	11.33206	0.727049	0.126563
PHACTR4	9.736543	7.080105	0.727168	0.025392
NR1D2	13.30071	9.677536	0.727595	0.005441
MBOAT2	9.928166	7.224756	0.727703	0.026468
RDH13	2.396934	1.745184	0.72809	0.017914
ERAL1	57.24434	41.69375	0.728347	0.069021
HYOU1	61.05519	44.47514	0.728442	0.105261
ASAH2	1.106471	0.806145	0.728573	0.298642

MTREX	28.81802	20.99721	0.728614	0.242959
RPA1	33.30961	24.27116	0.728653	0.038465
ANKRD42	1.909674	1.391561	0.72869	0.08914
LEMD2	8.226383	5.994779	0.728726	0.034249
WDR35	4.167424	3.03701	0.72875	0.183508
C11orf80	5.904254	4.303009	0.728798	0.060689
C8orf76	2.119228	1.544877	0.728981	0.062514
FAM122B	18.80009	13.70507	0.72899	0.006282
OTUD5	33.80942	24.65393	0.729203	0.133198
RMND1	18.3604	13.39073	0.729327	0.255687
CBX3	81.08456	59.14842	0.729466	0.093355
TACC2	1.142819	0.8338	0.729599	0.181144
ZNF697	5.396873	3.937741	0.729634	0.143823
PPP1R8	36.11107	26.35246	0.729761	0.076426
USP20	4.5242	3.301983	0.729849	0.013675
AP1B1	41.9406	30.61155	0.729879	0.044509
CEP170B	7.007663	5.115342	0.729964	0.146988
SLC30A5	19.29177	14.08251	0.729975	0.00925
ZNF385A	8.202334	5.989148	0.730176	0.002357
GIN1	2.586372	1.889088	0.730401	0.24876
ALKBH1	11.10327	8.110239	0.730437	0.096012
ANKRD7	1.244765	0.909445	0.730616	0.314566
TRIM36	1.796037	1.312313	0.730672	0.007229
FAM13A	2.630423	1.921996	0.730679	0.086511
SEC63	27.67333	20.22076	0.730695	0.014471
SNX13	2.275219	1.662525	0.73071	0.007566
GNB1L	9.09781	6.649512	0.730892	0.017187
EEF2K	6.188009	4.523007	0.730931	0.051336
CLTB	37.60026	27.48866	0.731076	0.008736
KDM1A	35.91041	26.26482	0.731398	0.006694
COG1	13.0003	9.509319	0.731469	0.046417
CEPT1	14.81126	10.83454	0.731507	0.052731
UBA5	15.26913	11.1728	0.731725	0.039236
TARSL2	5.75272	4.209456	0.731733	0.01585
IPO7	51.20997	37.47553	0.731801	0.009573
EIF3F	231.7723	169.6224	0.731849	0.027163
STK11IP	4.748705	3.475666	0.731919	0.07389
GLCE	3.249685	2.379275	0.732155	0.012091
NAT16	0.695643	0.509328	0.732169	0.601196
SLC12A6	2.358882	1.727469	0.732325	0.173869
UQCRB	69.91219	51.19871	0.732329	0.001222
HMGB3	32.1805	23.57249	0.732509	0.089449
LYRM7	4.451913	3.261306	0.732563	0.098906
GAR1	36.18104	26.50564	0.732584	0.170354
ZBTB25	1.966384	1.440573	0.7326	0.201004
STAT3	17.64282	12.92748	0.732733	0.083386
HYI	5.86461	4.298324	0.732926	0.103373
ZSWIM8	10.14135	7.435473	0.733183	0.280927
MBTPS2	10.37526	7.608078	0.733291	0.15488
CLIP4	2.944459	2.159193	0.733307	0.092876
SPATA7	4.240568	3.109796	0.733344	0.012409
REST	4.480207	3.285716	0.733385	0.005766
FAF2	28.53885	20.93616	0.733602	0.069864
RPS16	1338.656	982.8165	0.734181	0.050354
TMEM230	39.9572	29.34145	0.734322	0.018677
USP34	10.57709	7.768757	0.734489	0.086347
ANGPTL6	0.186526	0.137086	0.734941	0.504876
SYVN1	18.65699	13.71599	0.735166	0.174466

ASXL2	1.828736	1.344589	0.735256	0.123607
MECR	13.81344	10.15659	0.735269	0.233405
RBM27	7.687602	5.654183	0.735494	0.012766
UMAD1	9.278715	6.824562	0.735507	0.005882
ZNF77	6.229835	4.582365	0.735552	0.052994
NHLRC3	6.107415	4.493142	0.735686	0.142849
CIT	8.44421	6.212713	0.735736	0.082767
SKA2	27.30036	20.08728	0.735788	0.023506
MYL5	3.042435	2.238701	0.735825	0.157787
NCK1	7.24051	5.327905	0.735847	0.00898
ZFX	1.969861	1.449634	0.735907	0.170914
TCF12	7.288064	5.364621	0.736083	0.184722
AP3B2	0.93657	0.689458	0.736152	0.361805
THYN1	38.34664	28.22967	0.73617	0.065886
AAGAB	38.69247	28.4859	0.736213	0.029641
ZFP37	2.502098	1.842206	0.736265	0.013967
ZNF629	8.111884	5.974734	0.736541	0.28968
CSNK1G3	8.782557	6.469633	0.736646	0.001234
SMC2	11.94248	8.797459	0.736653	0.122028
ZKSCAN5	5.717893	4.212211	0.736672	0.101324
TMLHE	3.763485	2.773417	0.736928	0.205387
TMEM254	7.437245	5.480981	0.736964	0.250881
POC5	11.49421	8.470898	0.736971	0.178422
VPS13B	1.296558	0.955894	0.737255	0.094193
PDSS1	3.678431	2.712584	0.73743	0.283032
PLPBP	8.646771	6.376726	0.737469	0.002539
CENPK	9.88977	7.294767	0.737607	0.006908
GTF2A1	5.037883	3.716574	0.737725	0.030992
STK11	26.34093	19.43745	0.737918	0.001297
RUFY2	3.071051	2.266876	0.738143	0.080476
ATP5F1C	232.2847	171.4834	0.738247	0.078907
NDUFA10	13.10256	9.67346	0.738288	0.016536
MKLN1	9.316688	6.880611	0.738525	0.06421
REPIN1	20.18991	14.91591	0.738781	0.003258
YEATS2	11.16146	8.24843	0.73901	0.082219
KIF5C	8.743964	6.46221	0.739048	0.200987
INO80C	7.969507	5.890217	0.739094	0.09953
LOC10272	0.470118	0.347539	0.739258	0.113909
ELOB	139.7421	103.3059	0.739261	0.00965
SLC19A1	2.920775	2.159789	0.739457	0.436994
AFMID	13.8666	10.25408	0.73948	0.103145
SLC25A37	10.96752	8.110492	0.739501	0.181618
NCSTN	29.53935	21.85473	0.739851	0.10246
ERCC4	2.992724	2.214762	0.740049	0.284646
C6orf89	12.77149	9.451835	0.740073	0.003944
ASXL1	14.34314	10.61511	0.740083	0.058317
FUNDC2	36.14412	26.75151	0.740135	0.018142
CLN8	2.874316	2.12743	0.740152	0.283796
TNFRSF6B	1.858845	1.376571	0.740552	0.077963
TMEM86B	3.998275	2.961203	0.74062	0.492084
FBXO33	3.379275	2.503686	0.740895	0.018614
INHBC	0.033762	0.025016	0.740954	0.753007
NPFF	1.228957	0.910699	0.741034	0.624739
UQCRHL	31.48365	23.33609	0.741213	0.005614
KDM3A	13.32972	9.880244	0.741219	0.04176
ELP4	11.79932	8.746429	0.741266	0.18153
DCLK2	3.39267	2.515263	0.741382	0.143884
ACIN1	30.64729	22.72703	0.741568	0.008889

HEBP2	67.08772	49.75349	0.741619	0.02107
TMEM123	38.48613	28.54302	0.741644	0.081165
SCAMP1	11.49616	8.526798	0.741708	0.057839
SH3PXD2E	6.741583	5.000851	0.741792	0.090822
MRPL21	88.74633	65.83751	0.741862	0.027027
TMEM42	26.15344	19.40402	0.74193	0.103738
SIAH1	2.320665	1.721858	0.741967	0.021271
LRRC49	6.789793	5.038186	0.742024	0.015568
TMSB15A	14.14185	10.49767	0.742312	0.47429
TNFRSF4	0.034157	0.025357	0.742386	0.803795
MKRN1	35.69202	26.50057	0.742479	0.019535
CIP2A	10.54758	7.831995	0.742539	0.008441
LMO4	10.95613	8.135391	0.742542	0.176957
DDX19A	24.1418	17.93126	0.742748	0.001865
CFAP300	1.28277	0.952784	0.742755	0.514617
NXNL2	0.225846	0.167791	0.742943	0.260834
RGS4	0.320678	0.238293	0.743093	0.371121
SMCR8	3.924814	2.917026	0.743226	0.043206
SEC16A	13.96025	10.37894	0.743464	0.19437
FDPS	92.60108	68.85052	0.743517	0.17623
PARP16	5.33907	3.969908	0.743558	0.250314
C10orf88	4.929053	3.66606	0.743766	0.132875
ZNF106	7.532597	5.603154	0.743854	0.003024
FAM102B	4.94808	3.680798	0.743884	0.085576
SLC39A6	25.91849	19.28141	0.743925	0.039711
LCLAT1	4.1428	3.082804	0.744135	0.197603
PRKRIP1	16.25514	12.10453	0.744658	0.04356
VDAC3	90.23357	67.19646	0.744695	0.002189
COL4A3BI	6.020709	4.483864	0.74474	0.003695
MRPL45	42.12924	31.37611	0.744758	0.20548
CAMK2G	11.20106	8.343207	0.744859	0.076137
VDAC2	116.6902	86.92982	0.744963	7.79E-06
RFX1	3.491136	2.601353	0.745131	0.044837
PRDX3	124.0304	92.4372	0.745279	0.130162
GCSH	22.62636	16.86303	0.745283	0.312513
SUPV3L1	14.64784	10.92033	0.745525	0.065701
SSB	94.99708	70.86884	0.746011	0.11511
ATP5F1B	480.2646	358.2832	0.746012	0.049556
EPHA1	0.036185	0.026998	0.746123	0.567986
STXBP5	1.834654	1.368937	0.746155	0.010185
DNAJC22	1.612431	1.203328	0.746282	0.215523
KIF20B	10.0558	7.504504	0.746286	0.155564
LNPK	9.359524	6.986117	0.746418	0.056717
FOXN3	6.90036	5.15056	0.746419	0.061087
ODF3L1	0.265838	0.198445	0.746488	0.565584
PAPD5	1.944189	1.451362	0.746513	0.014335
MAGT1	37.63397	28.09771	0.746605	0.012281
USP16	21.09467	15.75241	0.746748	0.032328
NUMBL	13.54158	10.11378	0.746868	0.008751
SLC48A1	3.134193	2.340906	0.746893	0.001871
SNX16	2.039283	1.523401	0.747028	0.090164
TMPPE	0.443634	0.331459	0.747145	0.476576
SYNJ2BP	8.189397	6.118841	0.747166	0.020022
PNPLA8	9.395461	7.022314	0.747416	0.050746
CDK11A	5.053061	3.77677	0.747422	0.176869
PLD3	92.05331	68.81058	0.747508	0.161157
CCNE2	4.119929	3.079749	0.747525	0.137697
CRBN	12.54849	9.38716	0.748071	0.00533

SNRNP35	14.39367	10.77359	0.748495	0.054584
MPLKIP	45.01044	33.69016	0.748496	0.026112
MOCOS	6.714798	5.026797	0.748615	0.171723
RAB9A	31.27715	23.41931	0.748767	0.112221
WDR3	24.49612	18.34439	0.748869	0.275754
RUVBL2	73.59796	55.12799	0.749042	0.218119
FBXO3	7.768855	5.819596	0.749093	0.001239
CDR2	22.8214	17.09549	0.749099	0.114978
UBE2N	33.874	25.37643	0.749142	0.004506
ZNF789	1.23126	0.922894	0.749552	0.0883
UBE2Q2	3.966123	2.973212	0.749652	0.000475
NDOR1	6.973966	5.230222	0.749964	0.107981
C9orf64	8.913709	6.685894	0.750069	0.328535
HNRNPA3	111.5009	83.64082	0.750136	0.010895
RCL1	10.44543	7.837166	0.750296	0.312067
COA1	7.370046	5.529834	0.750312	0.029553
CHCHD4	22.30436	16.73762	0.750419	0.233664
ARPC1A	115.5301	86.70066	0.75046	0.02201
DCAF10	6.405725	4.808728	0.750692	0.007452
TTI1	18.5228	13.9082	0.750869	0.000402
PHF14	2.179461	1.636703	0.750967	0.175173
MYLK4	0.10021	0.075256	0.750981	0.371158
PRDM12	0.227106	0.170574	0.751079	0.573644
CDHR3	0.143171	0.107535	0.751092	0.456834
ECT2	19.94073	14.98484	0.751469	0.017078
YPEL5	27.02728	20.31638	0.751699	0.028048
COX7C	943.6198	709.3877	0.751773	0.128667
ZNF777	8.121734	6.106663	0.751892	0.359969
LMNTD1	0.073722	0.055433	0.751917	0.748405
STYXL1	16.45045	12.36943	0.75192	0.351052
PPIL3	16.87072	12.69094	0.752247	0.098806
SUFU	3.042888	2.289028	0.752255	0.047664
NPIPA3	0.051584	0.038829	0.75274	0.403846
TTC4	34.67276	26.10167	0.752801	0.040982
MYO19	12.15667	9.152807	0.752904	0.015304
SPAAR	0.097159	0.073157	0.752963	0.719832
SF3A3	70.50952	53.09798	0.753061	0.064379
PER2	1.506513	1.13541	0.753668	0.20177
SBF2	4.355006	3.283278	0.753909	0.07735
ZNF16	3.268902	2.465111	0.75411	0.111842
NELFE	70.62332	53.25984	0.75414	0.016398
PPM1F	11.83665	8.928536	0.754313	0.000118
NOMO2	13.96777	10.53723	0.754396	0.042338
ATP11B	3.477512	2.624811	0.754796	0.048932
XPC	15.83845	11.95526	0.754826	0.21681
EIF2B3	24.91237	18.80474	0.754835	0.236006
COQ5	31.99132	24.15511	0.755052	0.298566
DCUN1D2	3.177185	2.399591	0.755257	0.006682
HARS2	20.70218	15.63656	0.75531	0.002444
EIF4A3	70.53167	53.28878	0.75553	0.018201
TMEM168	2.782355	2.102658	0.755712	0.086251
SYBU	1.107475	0.837325	0.756066	0.313604
H2AFX	62.97819	47.61683	0.756084	0.037563
UBE3D	1.045588	0.790623	0.756151	0.335497
HLA-DMB	6.331181	4.787507	0.756179	0.324299
STOML2	164.5754	124.4519	0.7562	0.21112
DUSP11	15.07726	11.40303	0.756307	0.118571
CRELD1	15.95664	12.07374	0.756659	0.065949

CDC34	70.90662	53.66816	0.756885	0.079535
BBS1	8.911296	6.745003	0.756905	0.093266
ZFHX3	0.94981	0.719065	0.757061	0.200617
IL1RAP	1.737134	1.315138	0.757073	0.176332
VPS33A	9.416747	7.129983	0.75716	0.157239
EEF1AKM1	4.812574	3.645584	0.757512	0.024913
ACER3	3.263399	2.472694	0.757705	0.020875
MED17	5.278411	3.999665	0.75774	0.220074
PMEPA1	10.73823	8.138946	0.757941	0.503697
SH3TC2	0.041258	0.031278	0.758095	0.732074
BAP1	38.98042	29.55829	0.758286	0.01158
CENPH	76.35976	57.92005	0.758515	0.002222
HERPUD2	6.509045	4.937347	0.758536	0.051276
NGRN	117.311	88.9896	0.758578	0.014592
DCAF15	19.30702	14.64657	0.758614	0.035684
TRMT10C	32.09727	24.35753	0.758866	0.470569
KRBOX4	5.833654	4.427813	0.759012	0.034858
RABGAP1I	3.010446	2.285366	0.759145	0.402539
C1orf21	6.182273	4.693355	0.759163	0.043215
EIF4H	188.1951	142.946	0.759563	0.006458
MAMDC4	0.808079	0.613942	0.759756	0.409821
PDE5A	2.886278	2.192881	0.759761	0.189839
NUAK2	1.071958	0.814501	0.759826	0.352062
VWA1	1.535053	1.166878	0.760155	0.291881
CDRT1	0.04627	0.035182	0.760348	0.811456
RRP7A	18.93679	14.39964	0.760406	0.300324
EXOSC5	34.14667	25.97499	0.760689	0.248754
MFSD14A	18.11518	13.78172	0.760783	0.011669
HS6ST3	0.476922	0.362885	0.760889	0.351938
VMA21	22.41913	17.06643	0.761244	0.015087
HSD17B12	23.77034	18.09677	0.761317	0.143151
ZNF830	11.96954	9.112997	0.761349	0.223118
NEU4	0.116792	0.08894	0.761522	0.5345
ADAM19	2.460858	1.874054	0.761545	0.000814
C1orf174	5.574252	4.246179	0.761749	0.015114
ALKBH4	8.096455	6.169259	0.76197	0.053699
XPA	7.874228	6.000278	0.762015	0.005535
VAMP2	31.64169	24.11297	0.762063	0.007524
RASD1	0.762446	0.581231	0.762324	0.597323
ITCH	9.141202	6.969624	0.762441	0.104942
UBXN2A	6.63573	5.059457	0.762457	0.098832
MSI2	3.398456	2.593046	0.763007	0.045176
RBM17	28.09558	21.4444	0.763266	0.012778
ESS2	6.172577	4.714711	0.763816	0.2041
THUMPD1	9.386652	7.169847	0.763834	0.021524
ADAT3	2.369682	1.810077	0.763848	0.199423
RSL1D1	57.5399	43.95627	0.763927	0.17149
DLST	49.58525	37.88817	0.764102	0.007721
SERPINI1	4.547968	3.475375	0.76416	0.139774
CREB1	3.249945	2.483862	0.764278	0.038139
PKIA	6.329017	4.839291	0.76462	0.445145
KBTBD2	12.27669	9.390853	0.764934	0.092808
CLDN14	0.065711	0.050277	0.765117	0.75503
RAD1	11.84092	9.060535	0.765189	0.079309
TYW3	15.58755	11.92854	0.765261	0.216
GNPNAT1	16.23176	12.42199	0.765289	0.071112
CAMKK2	9.471367	7.250217	0.765488	0.071244
EIF4E	6.452147	4.93918	0.76551	0.043524

NFATC3	7.598574	5.816885	0.765523	0.026614
AP1G1	17.27861	13.22974	0.765671	0.040528
HDGFL3	14.94737	11.44648	0.765785	0.014591
ARHGEF6	9.622327	7.369604	0.765886	0.060679
PDRG1	42.1648	32.29508	0.765925	0.2
KIF5A	4.146901	3.176338	0.765955	0.012815
CPD	14.70876	11.26654	0.765975	0.036409
ADD3	13.93945	10.67954	0.766138	0.149001
DCDC1	0.083157	0.063724	0.766309	0.373554
GLRB	3.512493	2.692084	0.766431	0.070174
MAPK8	4.678627	3.58618	0.766503	0.024701
ERI2	5.82784	4.467804	0.766631	0.244577
SSR4	131.6633	100.9831	0.76698	0.001985
HMBS	20.01786	15.35676	0.767153	0.316513
9-Mar	11.09144	8.509993	0.767258	0.016056
BLZF1	6.688082	5.132634	0.76743	0.118473
EPC2	2.891582	2.219414	0.767543	0.294066
GAK	12.88129	9.889372	0.767732	0.037445
ZC3H12B	0.366388	0.2813	0.767765	0.540035
AP1S2	12.59817	9.672448	0.767766	0.041565
CCDC47	44.81029	34.41093	0.767925	0.023904
GTF3C5	38.74406	29.76231	0.768177	0.090689
CTSV	4.848281	3.72466	0.768243	0.401952
LOC10798	0.976993	0.750872	0.768554	0.473299
TAX1BP1	41.90502	32.20888	0.768616	0.025616
MRPL32	52.7643	40.55979	0.768697	0.018183
RPS15	1190.85	916.1936	0.769361	0.073912
IFT20	41.27645	31.75901	0.769422	0.341303
FBXO34	6.048919	4.655499	0.769641	0.018423
RAPGEFL1	1.928951	1.484747	0.769717	0.106746
SAE1	80.53033	61.99406	0.769823	0.008665
SERINC1	85.33671	65.71049	0.770014	0.01336
TMEM218	3.737708	2.878572	0.770143	0.349033
SAYSD1	2.623004	2.020091	0.770144	0.343188
ZNF700	5.204952	4.009824	0.770386	0.229755
ZNF329	1.745059	1.344411	0.77041	0.207216
LSMEM2	0.061031	0.047026	0.770526	0.651351
LOC10798	0.742781	0.572724	0.771054	0.46036
SIRT3	5.977086	4.609435	0.771184	0.173174
HSCB	11.26412	8.687214	0.771229	0.128545
RCOR1	7.538997	5.814548	0.771263	0.079489
FBF1	5.092753	3.927884	0.771269	0.194637
XPNPEP3	3.814785	2.942318	0.771293	0.013993
TKT	174.5738	134.6706	0.771425	0.043389
TXNDC5	121.3075	93.58254	0.771449	0.100793
FDX1	9.752359	7.524272	0.771533	0.215593
FAM120B	5.322204	4.106773	0.77163	0.032475
TRIM26	9.053028	6.987139	0.771801	0.261791
TOGARAM	1.274576	0.983842	0.771897	0.283712
LARP1	35.96384	27.76122	0.77192	0.125098
C11orf98	0.262019	0.202296	0.772065	0.555412
SLC30A1	3.437686	2.654701	0.772235	0.056961
PQBP1	51.06042	39.43593	0.772339	0.035212
LPIN1	5.278146	4.076639	0.772362	0.114063
DDIT4L	1.881214	1.4532	0.77248	0.345876
ATP6V1B2	29.53542	22.81907	0.7726	0.087062
YTHDF1	23.79476	18.38508	0.772653	0.187482
CWC25	10.35526	8.002011	0.772749	0.211397

JAM3	21.32941	16.48678	0.77296	0.036562
DDX3X	50.79504	39.27486	0.773203	0.015408
FRY	0.495868	0.383407	0.773205	0.367953
AGAP1	3.310269	2.560088	0.773378	0.019569
RNF10	55.69399	43.0734	0.773394	0.031093
RALA	20.18886	15.61478	0.773435	0.000229
RPLP2	1874.599	1450.328	0.773674	0.089097
ARL14EP	10.45964	8.096815	0.774101	0.10373
NDFIP1	32.38029	25.07251	0.774314	0.018455
ZNF552	1.587007	1.22896	0.774388	0.060957
LARP7	17.22428	13.33857	0.774405	0.184718
NXT1	22.08255	17.103	0.774503	0.174421
RNFT2	4.72346	3.658819	0.774606	0.146798
FBRS	25.48805	19.74368	0.774625	0.095744
CDCA8	31.46208	24.37588	0.77477	0.021139
UHMK1	11.51905	8.926502	0.774934	0.232943
VPS13C	2.206036	1.710631	0.775432	0.355701
PIAS4	9.506601	7.372177	0.77548	0.094004
RRM1	95.2165	73.84212	0.775518	0.125866
PLD2	8.781132	6.810113	0.775539	0.06899
METTL4	5.380156	4.173059	0.775639	0.243004
INVS	4.437744	3.443266	0.775905	0.05917
ZWINT	83.82737	65.0688	0.776224	0.01009
CLMN	2.254694	1.750328	0.776304	0.256829
SPATA21	0.139902	0.108609	0.77632	0.528834
ZNF160	2.015824	1.56531	0.776511	0.002039
RCC1	56.19133	43.64683	0.776754	0.048156
MICU2	24.90877	19.3496	0.776819	0.241889
DMWD	11.86576	9.219538	0.776987	0.029883
PPP1R35	32.24518	25.05711	0.777081	0.001662
ACO2	51.76013	40.23702	0.777375	0.086602
HNRNPUL	92.99588	72.30458	0.777503	0.017952
BANP	2.586953	2.011383	0.77751	0.095712
TOR3A	26.52143	20.62142	0.777538	0.063933
DDX5	124.5636	96.87866	0.777745	0.188233
RPS6KA2	7.062676	5.493281	0.77779	0.289157
HNRNPAC	82.53787	64.21516	0.778008	0.075756
STX18	9.480491	7.379484	0.778386	0.019428
RPS5	1231.927	959.1754	0.778597	0.025341
TCFL5	9.623262	7.494365	0.778776	0.06327
SPAG7	41.31342	32.17897	0.778899	0.002055
METTL5	66.05309	51.45829	0.779044	0.199133
POP7	59.95913	46.71288	0.779079	0.354262
API5	29.19077	22.74384	0.779145	0.057628
RAD9B	0.132981	0.103619	0.779195	0.462335
ZSCAN22	1.339621	1.044129	0.779421	0.040056
PAK1IP1	13.72143	10.69742	0.779614	0.505088
ZNF428	40.86344	31.85891	0.779643	0.024551
FER1L6	0.188222	0.14677	0.77977	0.66489
JAG1	2.849446	2.222193	0.779869	0.053543
PRX	1.312661	1.023808	0.779949	0.42495
LRRIQ1	0.46229	0.360566	0.779957	0.319342
TNRC6A	8.812234	6.874477	0.780106	0.232038
PDXDC1	16.62165	12.96827	0.780204	0.134602
HAVCR2	0.146046	0.113946	0.780204	0.684096
FAAP100	5.763725	4.497053	0.780234	0.101942
QSER1	7.572025	5.908283	0.780278	0.23214
SUV39H2	7.598645	5.931748	0.780632	0.18506

IL11RA	7.793216	6.084626	0.780759	0.070383
SMU1	17.21948	13.44598	0.780859	0.003581
UBE2M	171.5948	134.1256	0.781641	0.025393
MIER2	6.825761	5.336772	0.781857	0.27396
TRAF2	8.823239	6.898522	0.781858	0.068992
SS18	20.80472	16.26866	0.78197	0.002353
ASH2L	17.35158	13.56947	0.782031	0.229485
ROS1	0.103776	0.081162	0.782092	0.406318
NPEPPS	22.89062	17.9027	0.782098	0.017048
RASAL2	1.559447	1.220202	0.782458	0.244534
OSBPL6	3.680808	2.880802	0.782655	0.073909
GRWD1	16.69332	13.06632	0.782727	0.334051
METTL25	0.158959	0.124428	0.782765	0.148153
FAM133A	0.297288	0.232712	0.782781	0.420367
RRP8	4.001072	3.132293	0.782863	0.000686
USP15	4.985544	3.904354	0.783135	0.015209
KIAA0895l	6.722073	5.265486	0.783313	0.114261
BCL2L2	11.45743	8.975059	0.78334	0.040345
NUP214	8.657162	6.781568	0.783348	0.188289
TULP3	11.54745	9.047333	0.783492	0.060512
POLD2	98.16395	76.91582	0.783545	0.173261
TAMM41	3.087858	2.419518	0.783559	0.41003
STK33	1.32476	1.038229	0.783712	0.361086
YLPM1	9.912492	7.770087	0.783868	0.008973
TTC39C	2.433943	1.908078	0.783945	0.189173
PPFIA1	9.340522	7.324999	0.784217	0.108757
CCDC97	7.900296	6.196483	0.784336	0.24603
SPOP	15.84136	12.42523	0.784353	0.266207
CRADD	1.919709	1.5058	0.78439	0.291276
TTC31	9.316616	7.309668	0.784584	0.070352
MCPH1	0.983974	0.772181	0.784758	0.097123
ST6GALNA	15.05259	11.81446	0.784879	0.003422
ITGAE	4.896235	3.843145	0.784918	0.007465
SPA17	13.32523	10.46311	0.78521	0.081444
NR1H3	3.794366	2.979387	0.785213	0.124135
ELF4	10.70705	8.407328	0.785214	0.027218
LAMA2	0.163715	0.128566	0.785303	0.286769
CD14	0.252139	0.19803	0.785403	0.674064
PPP1R42	0.254226	0.199674	0.785419	0.424246
WTAP	21.54241	16.92037	0.785445	0.000633
KLHL29	3.041986	2.389529	0.785516	0.122355
SGCE	11.48054	9.018893	0.785581	0.013273
DHRS9	0.137423	0.107974	0.785705	0.80947
CDK5RAP2	20.46354	16.07947	0.785762	0.002477
ECI2	62.22417	48.89397	0.785771	0.354308
CDK5RAP1	15.11774	11.87968	0.78581	0.043532
RHOQ	35.24965	27.70232	0.785889	0.359477
MAK16	7.555744	5.939428	0.786081	0.32399
PAG1	2.042419	1.605805	0.786227	0.311576
HAND2	1.51224	1.189181	0.786371	0.465486
ANKRD54	12.19774	9.593698	0.786514	0.108929
REG4	0.073753	0.058009	0.78654	0.812197
CDK13	4.895002	3.850296	0.786577	0.007695
CSNK1D	22.18766	17.45982	0.786916	0.010165
NOL7	46.25884	36.40234	0.786927	0.085871
NCKAP5L	5.928566	4.665735	0.786992	0.050415
ARHGEF1(	5.171582	4.070149	0.787022	0.209961
SUV39H1	10.30938	8.113916	0.787042	0.004949

NIN	3.633535	2.86008	0.787134	0.190406
PTRHD1	40.63057	31.98988	0.787335	0.122094
EIF3K	304.2933	239.5869	0.787355	0.011823
ELOVL5	32.84147	25.86172	0.787471	0.015355
PITPNB	43.8931	34.56863	0.787564	0.024385
GPR107	19.76716	15.56868	0.787603	0.144608
GAS8	5.820852	4.584675	0.78763	0.005465
NUP85	28.67537	22.58925	0.787758	0.004114
MZF1	3.427575	2.701299	0.788108	0.209423
MCRIP1	56.10201	44.21641	0.788143	0.079605
SCAF11	7.45896	5.882791	0.788688	0.049418
METTL21A	4.000532	3.156667	0.789062	0.0586
CWC22	11.87256	9.36918	0.789146	0.203387
USP7	26.83016	21.17921	0.789381	0.05667
RAB11A	37.92511	29.9451	0.789585	0.005241
SLC39A7	105.4437	83.25751	0.789592	0.23289
PHLPP2	2.866225	2.264182	0.789953	0.227798
RCE1	14.98257	11.83589	0.789978	0.176461
EXOC6B	4.388081	3.467285	0.79016	0.188969
ZBTB3	1.484425	1.17309	0.790266	0.06782
KAT5	26.92923	21.28623	0.790451	0.090871
UBXN2B	7.959637	6.295544	0.790933	0.003874
MCUR1	8.499575	6.724394	0.791145	0.018903
SMAD1	3.797658	3.00493	0.791259	0.252051
GTPBP2	32.69959	25.87714	0.79136	0.255612
TAF7	57.50761	45.51306	0.791427	0.24193
TYMP	0.516433	0.408755	0.791497	0.410584
ZRANB1	10.73054	8.494572	0.791626	0.081855
SLC35F5	3.956514	3.132246	0.791668	0.038735
MBLAC1	1.171008	0.927373	0.791944	0.29541
RBL2	12.64442	10.01773	0.792265	0.01834
RPS6KB2	40.7903	32.32754	0.79253	0.06758
PTDSS2	12.82121	10.16369	0.792724	0.040097
AZIN1	24.04802	19.0674	0.792889	0.024237
NOL10	10.3789	8.230169	0.792971	0.200096
MPP1	33.29981	26.40729	0.793016	0.019574
STAMBP	12.77398	10.1327	0.793229	0.0009
FAM208A	11.90788	9.446761	0.79332	0.452947
DHX36	18.5949	14.75221	0.793347	0.224034
PARG	9.345363	7.414914	0.793432	0.018105
IBTK	9.305269	7.383143	0.793437	0.082138
SULT1A3	0.163221	0.129509	0.793459	0.713131
GNL3L	4.271411	3.39036	0.793733	0.240531
MTFP1	22.93189	18.20261	0.793768	0.323922
SPAG1	2.389821	1.898515	0.794417	0.121285
HADH	37.1419	29.50783	0.794462	0.190511
BRIX1	77.02803	61.20165	0.794537	0.190183
ADIPOR2	24.06393	19.12031	0.794563	0.025466
CC2D2B	0.019307	0.015346	0.794862	0.590856
RACGAP1	33.38009	26.53667	0.794985	0.007536
FKBP14	8.490985	6.750505	0.79502	0.078307
ITSN1	2.200482	1.749506	0.795056	0.092988
KRTCAP2	186.9922	148.7238	0.795348	0.015182
NCAPH2	27.58551	21.94227	0.795427	0.001919
FGD1	5.795083	4.61013	0.795524	0.03514
NCOR2	19.37451	15.41482	0.795624	0.06178
GOLGB1	10.14421	8.071812	0.795706	0.128143
DNM1L	24.65642	19.62068	0.795764	0.029857

KLHL22	3.454908	2.749348	0.79578	0.094097
GSTP1	237.8956	189.3797	0.796063	0.219394
POLR2M	0.205873	0.163935	0.796293	0.382965
MRPL12	116.3419	92.65006	0.79636	0.226801
C12orf49	12.95772	10.32361	0.796715	0.092919
HLF	0.459659	0.366221	0.796723	0.632008
BICRAL	1.300009	1.035845	0.796798	0.315013
PCF11	3.970338	3.164863	0.797127	0.227816
CDC42BPE	17.46492	13.92324	0.797212	0.01649
RGS17	3.990513	3.181348	0.797228	0.22039
KLF3	4.045951	3.225588	0.797239	0.086057
FAM209B	0.185773	0.148144	0.797446	0.698842
SLC4A1AF	19.65263	15.67296	0.797499	0.098602
MAGED2	115.1376	91.82927	0.797561	0.178848
ULBP2	23.62515	18.84747	0.797771	0.266886
UBFD1	18.68168	14.91866	0.798572	0.109155
HIRA	14.92007	11.91754	0.798759	0.002492
CHKA	4.633583	3.701423	0.798825	0.02021
NDUFAF4	14.36865	11.47909	0.798899	0.319518
NOSIP	25.0408	20.00514	0.798902	0.049031
CNOT8	20.14727	16.09726	0.79898	0.045856
COX5A	250.1529	199.9073	0.79914	0.163891
RXRA	4.612271	3.686772	0.79934	0.058025
CCDC122	0.97001	0.775407	0.79938	0.608289
COG6	5.986718	4.786805	0.799571	0.047031
CNOT4	4.847502	3.876031	0.799593	0.160845
NOC4L	27.32287	21.84847	0.799641	0.029948
TLN2	1.516174	1.21248	0.799697	0.246207
GRB10	7.463323	5.968927	0.799768	0.208421
LOC10099	5.913167	4.729885	0.79989	0.417374
CPSF7	30.31847	24.25377	0.799967	0.089859
NOD2	0.103855	0.083088	0.80004	0.590063
MRPS10	29.26147	23.42751	0.800627	0.236674
ZXDA	0.504953	0.404299	0.800666	0.234367
CEP126	1.108121	0.887382	0.800799	0.024921
IDH3A	32.34734	25.9268	0.801512	0.03826
SAT2	38.94256	31.21712	0.801619	0.150059
USF3	0.91135	0.730648	0.801721	0.124298
FBXL15	5.946142	4.767746	0.801822	0.058376
PDS5A	13.69891	10.98415	0.801826	0.048679
RPS28	1036.568	831.5103	0.802177	0.114733
SPRYD7	6.192273	4.967583	0.802223	0.149888
TCEAL8	88.60242	71.08119	0.802249	0.013532
GORASP1	11.19173	8.978676	0.80226	0.024304
TATDN1	17.57085	14.09987	0.802458	0.052127
RBBP8	24.24422	19.4568	0.802533	0.252031
FUNDC1	27.05805	21.72059	0.80274	0.308723
SMIM10	9.350811	7.510705	0.803214	0.41382
YIPF2	26.74287	21.48034	0.803217	0.431308
MRPL15	30.13265	24.20863	0.803402	0.363502
DSN1	48.17192	38.70342	0.803444	0.018895
TMEM100	2.132488	1.713922	0.803719	0.489505
CANX	207.3947	166.7219	0.803887	0.03449
RPL35A	1432.389	1151.653	0.804009	0.070298
NIFK	39.10906	31.45778	0.80436	0.269974
MARCKS	43.77086	35.2182	0.804604	0.097476
EEA1	2.424978	1.951654	0.804813	0.084609
MIA3	10.83866	8.723395	0.804841	0.003199

SLC25A44	5.706405	4.593538	0.804979	0.110673
SHISA5	61.0684	49.15947	0.80499	0.177151
PPM1A	3.901746	3.140958	0.805013	0.113281
GRK3	4.739589	3.815963	0.805125	0.130255
ZNF580	27.74827	22.34375	0.80523	0.055745
TMA16	13.95537	11.23763	0.805255	0.303324
PRKCI	10.84133	8.73216	0.805451	0.036976
C1D	12.82897	10.33495	0.805594	0.121876
COQ3	17.61922	14.20893	0.806445	0.322216
SARM1	4.405558	3.553118	0.806508	0.401474
ZNF521	2.758937	2.225571	0.806677	0.059783
CHAF1B	11.17176	9.015788	0.807016	0.148526
ETFBKMT	0.25613	0.206712	0.807061	0.544183
NDUFV1	135.3511	109.2727	0.807328	0.051824
GPR19	0.305978	0.247142	0.807711	0.547716
WIZ	8.019959	6.478656	0.807817	0.077315
CELSR3	2.454739	1.98306	0.80785	0.479344
KAT14	2.047465	1.654221	0.807936	0.497779
GFM1	16.05233	12.96943	0.807947	0.164148
TGFBRAP1	3.359068	2.714001	0.807962	0.168835
NPHP3	4.526245	3.657115	0.80798	0.215896
IFT122	4.278241	3.457244	0.808099	0.010218
SKIV2L	12.44065	10.0536	0.808125	0.109219
FAM204A	8.87063	7.170715	0.808366	0.109961
L3MBTL2	13.24134	10.70394	0.808372	0.312841
CCNDBP1	7.38762	5.973598	0.808596	0.17903
HLA-DRA	76.00341	61.46532	0.808718	0.608678
ORMDL1	21.53028	17.42153	0.809164	0.100577
LOC1005C	0.433689	0.351038	0.809423	0.689202
TSR1	22.96481	18.59104	0.809545	0.119188
ACSL3	18.28842	14.80651	0.809611	0.185236
VPS28	56.01622	45.35689	0.80971	0.137584
BOP1	30.93542	25.05037	0.809763	0.445117
FMC1	0.370387	0.300093	0.810214	0.59475
MDH1	154.4433	125.1371	0.810247	0.080145
ABCB9	1.653052	1.339556	0.810353	0.18419
KATNB1	13.1448	10.65362	0.810481	0.383366
INCENP	13.56318	10.99339	0.810532	0.057604
PPIP5K1	2.267795	1.83912	0.810973	0.059472
IDI1	10.01835	8.125191	0.811031	0.341502
THAP4	25.05411	20.32144	0.811102	0.111053
DALRD3	15.24482	12.36528	0.811113	0.088122
WDR4	3.665136	2.972986	0.811153	0.451088
NR2F2	14.71354	11.93625	0.811243	0.511666
SPTLC1	20.76458	16.85298	0.811622	0.057265
ARHGAP1	15.41852	12.51606	0.811755	0.134467
CGGBP1	27.31068	22.16985	0.811765	0.110521
UBQLN1	52.42172	42.55897	0.811858	0.157259
FXN	1.9455	1.579621	0.811936	0.493028
C9orf85	7.092365	5.758929	0.81199	0.244043
BTN3A3	7.585269	6.159784	0.812072	0.371478
MOV10	10.86143	8.820997	0.81214	0.20924
ZNF532	3.062764	2.488172	0.812394	0.344978
MAP2K5	4.036485	3.280006	0.81259	0.106214
ZNF442	0.145796	0.118481	0.812649	0.67283
HEMK1	3.721878	3.025582	0.812918	0.223844
MECOM	1.579465	1.284308	0.813128	0.5385
NADK	13.57887	11.04231	0.813198	9.82E-05

CD79A	0.207618	0.16886	0.813319	0.644346
ANKRD11	11.2888	9.183202	0.813479	0.391307
DMAC1	14.7455	11.9992	0.813753	0.377548
DYNLT1	211.1097	171.8254	0.813915	0.049458
EARS2	9.933304	8.085542	0.813983	0.139631
EIF1AX	34.32362	27.94009	0.814019	0.126191
MZT2B	150.7576	122.7231	0.814043	0.06169
COX11	7.515869	6.118466	0.814073	0.11732
DCAF11	10.24765	8.346464	0.814476	0.125103
NMT1	36.60771	29.81612	0.814476	0.016054
YRDC	18.68432	15.22436	0.81482	0.118325
MTTP	0.082847	0.067505	0.814821	0.73371
UACA	14.92733	12.16321	0.814829	0.322459
ATF6	10.09222	8.224154	0.8149	0.084603
SEMA6D	0.553279	0.450895	0.81495	0.077643
ZNF799	1.607572	1.310517	0.815215	0.216744
ITM2C	92.15683	75.13734	0.81532	0.09227
SNRNP27	30.26956	24.68058	0.81536	0.024876
TMEM260	2.623404	2.139607	0.815584	0.104038
MAP3K4	7.816317	6.376293	0.815767	0.082139
ENPP1	8.772543	7.156522	0.815786	0.136014
SNRPD3	56.92178	46.43859	0.815832	0.17569
ZFP41	1.668851	1.361672	0.815934	0.566202
CFAP44	0.661783	0.539998	0.815974	0.692616
NPHP4	1.452792	1.185607	0.816089	0.010958
ORMDL2	33.72188	27.53857	0.816638	0.04802
SDHAF1	16.76446	13.69281	0.816776	0.209426
TRIAP1	47.11422	38.48483	0.816841	0.125897
CD2BP2	41.9886	34.30953	0.817115	0.223243
TMEM259	72.56038	59.29267	0.817149	0.199309
CTXN1	18.39281	15.03077	0.817209	0.415619
TM2D1	33.1583	27.10463	0.817431	0.176125
SNX22	1.327168	1.084906	0.817459	0.377939
OTULIN	2.819383	2.30476	0.81747	0.173422
BLOC1S4	11.14051	9.107257	0.81749	0.386924
ZNF687	7.215267	5.902764	0.818094	0.090524
GGT7	27.70478	22.66742	0.818178	0.127924
PPP5D1	0.184395	0.150928	0.818501	0.543287
JUNB	61.43987	50.29818	0.818657	0.258805
IKBKB	6.070037	4.970598	0.818874	0.152316
LRRC14	5.957157	4.880197	0.819216	0.03167
C5orf24	8.281704	6.784505	0.819216	0.096897
ATR	5.755665	4.716173	0.819397	0.191235
SMIM27	7.777693	6.374059	0.819531	0.096645
C16orf46	1.125763	0.922754	0.81967	0.299541
ACOX1	3.520055	2.885982	0.819868	0.066424
G3BP2	30.08831	24.67232	0.819997	0.057101
PPP6R2	13.15725	10.79508	0.820466	0.000937
MYC	18.52429	15.19954	0.820519	0.641007
HNRNPL	132.7742	108.9641	0.820672	0.102978
EXTL3	13.82063	11.34282	0.820717	0.233293
MBD4	14.82373	12.16761	0.82082	0.180993
DAZAP1	42.48395	34.87766	0.820961	0.047045
ASPHD2	3.88383	3.189346	0.821186	0.077207
PCLAF	40.12817	32.95325	0.8212	0.111249
MTDH	17.44206	14.32472	0.821275	0.232686
TYMS	64.46217	52.97864	0.821856	0.156845
BHLHE40	67.34208	55.3466	0.821872	0.275194

KLC1	56.29242	46.27064	0.821969	0.14441
ZC3HAV1	7.926933	6.516041	0.822013	0.130318
NKIRAS1	6.909967	5.682306	0.822335	0.210164
C3orf62	1.517453	1.247954	0.8224	0.400094
LOC11226	0.597529	0.491482	0.822524	0.563561
BUB1B	25.07859	20.63043	0.822631	0.287536
SUPT6H	26.54167	21.84047	0.822875	0.258357
RBMS3	1.105776	0.910191	0.823124	0.480332
ENPP2	9.382388	7.723607	0.823203	0.154904
CD8B	0.003663	0.003015	0.823225	0.898036
PIRT	0.005628	0.004634	0.823225	0.898036
GPAT2	0.005966	0.004911	0.823225	0.898036
FAM92B	0.010246	0.008435	0.823225	0.898036
CBLC	0.010278	0.008461	0.823225	0.898036
GLP1R	0.002651	0.002183	0.823225	0.898036
PDE6H	0.018491	0.015222	0.823225	0.898036
LOC10105	0.009044	0.007445	0.823225	0.898036
DPEP2NB	0.032925	0.027105	0.823225	0.898036
TMEM262	0.038102	0.031367	0.823225	0.898036
TOMM20L	0.028069	0.023107	0.823225	0.898036
BFSP2	0.004246	0.003495	0.823225	0.898036
C22orf24	0.012208	0.01005	0.823225	0.898036
TAS2R13	0.023834	0.019621	0.823225	0.898036
MBOAT4	0.007857	0.006468	0.823225	0.898036
CPA1	0.012945	0.010657	0.823225	0.898036
PRND	0.004831	0.003977	0.823225	0.898036
KIR2DL4	0.012355	0.010171	0.823225	0.898036
LOC10537	0.00733	0.006034	0.823225	0.898036
TLR7	0.003908	0.003217	0.823225	0.898036
KBTBD12	0.004142	0.00341	0.823225	0.898036
SNRNP25	27.56469	22.69673	0.823399	0.148464
TMEM92	0.472236	0.388902	0.823533	0.577666
POPDC2	0.673573	0.554756	0.823602	0.698065
C12orf65	8.283229	6.822672	0.823673	0.194147
HMGCR	11.98877	9.878506	0.82398	0.311733
TMEM101	11.36822	9.370199	0.824245	0.285621
HIPK3	2.318918	1.911577	0.82434	0.082267
SP4	0.621615	0.51256	0.824561	0.152777
RAF1	34.91891	28.79898	0.824739	0.040151
MFAP3	6.817868	5.626667	0.825282	0.316052
ERICH1	0.795091	0.65639	0.825554	0.154074
CASP3	15.74968	13.00412	0.825675	0.092403
STK36	3.924014	3.24067	0.825856	0.188998
ALDH6A1	5.361318	4.42789	0.825896	0.240117
FLCN	5.68628	4.696781	0.825985	0.24302
TBRG1	5.029997	4.155893	0.826222	0.165063
SLC5A3	5.369711	4.437344	0.826366	0.519135
LIFR	2.797594	2.312349	0.826549	0.141945
PKN1	65.42506	54.08283	0.826638	0.135019
MTOR	8.441837	6.978861	0.826699	0.278941
CCT2	126.9274	104.9457	0.826817	0.17397
GOLT1B	24.99489	20.66773	0.826878	0.455262
ABHD16A	7.160864	5.921443	0.826917	0.203456
PTK2	17.01645	14.07126	0.826921	0.040725
C7orf26	7.664402	6.339389	0.827121	0.082162
C15orf40	6.618793	5.475187	0.827218	0.254491
VPS51	32.55011	26.92708	0.82725	0.03693
HADHB	48.36132	40.00818	0.827276	0.051349

SNRPG	101.3447	83.84907	0.827365	0.019066
TCAF1	10.82466	8.957235	0.827485	0.018218
NUDT5	42.65495	35.30104	0.827595	0.079351
LATS2	2.185203	1.809227	0.827944	0.188263
CCNK	29.79397	24.66824	0.827961	0.164212
MZT1	32.65478	27.04165	0.828107	0.242975
GPATCH1	4.741237	3.926683	0.828198	0.081013
DENR	67.84476	56.20263	0.8284	0.216818
KLHDC4	4.176966	3.460377	0.828443	0.229248
RNF139	10.80797	8.954889	0.828545	0.092976
RNASE4	3.459861	2.867343	0.828745	0.558597
MRPL37	164.1298	136.034	0.82882	0.12448
CENPL	5.537695	4.591574	0.829149	0.232057
DCAF1	6.319706	5.241376	0.82937	0.006731
AHCYL1	47.44423	39.35198	0.829437	0.016749
DPY30	20.07673	16.65247	0.829441	0.156162
RBM18	16.24576	13.47839	0.829655	0.22623
MPHOSPH	5.976223	4.959688	0.829903	0.010814
GOLGA7	25.99468	21.5774	0.83007	0.021846
CLUH	11.61973	9.645461	0.830094	0.170427
RUNDC1	4.403553	3.655436	0.830111	0.116759
TMEM79	4.252433	3.530043	0.830123	0.206568
ELP3	10.79086	8.959075	0.830246	0.059625
C16orf86	0.954508	0.792632	0.830409	0.474744
POLR3G	5.965327	4.953957	0.830459	0.301104
CFL2	31.50953	26.16977	0.830535	0.010445
PPP1R2	15.14095	12.5768	0.830648	0.019774
MSTO1	5.652808	4.696368	0.830803	0.124897
SPC24	25.48645	21.17475	0.830824	0.089556
CEBPD	12.80846	10.64515	0.831103	0.345111
PPFIBP1	8.897454	7.395763	0.831222	0.241536
TULP4	1.77514	1.47592	0.831438	0.271883
CAMK2D	7.07383	5.882447	0.831579	0.132514
RBM10	23.52656	19.56519	0.831621	0.027311
ATP5IF1	86.1423	71.64181	0.831668	0.047447
ANK1	1.06054	0.882045	0.831694	0.370253
VRK3	12.62831	10.50355	0.831746	0.061906
NGLY1	16.23891	13.50822	0.831843	0.142409
CARMIL2	3.395333	2.826121	0.832354	0.635981
MCRIP2	13.53951	11.27032	0.832402	0.351651
SLC5A6	9.16369	7.627989	0.832415	0.358796
PCMTD1	4.413364	3.674908	0.832677	0.337207
RING1	34.08254	28.38144	0.832727	0.343751
NUP62	26.04421	21.69038	0.83283	0.115103
SLC7A6	7.225414	6.017717	0.832854	0.362653
PRDM2	2.244725	1.869698	0.832929	0.194651
STRIP1	12.76083	10.62969	0.832993	0.104445
KLLN	0.178618	0.14884	0.833283	0.784955
C5orf30	5.173864	4.311395	0.833303	0.076476
TTC9C	16.42454	13.68704	0.833329	0.175717
RAB21	5.070328	4.225964	0.83347	0.153234
PDHX	14.23614	11.86628	0.833532	0.225227
THRA	7.882049	6.569988	0.833538	0.058114
BMPR2	3.266013	2.723865	0.834003	0.211532
CLDN7	0.805409	0.671757	0.834058	0.636261
ZNF770	10.33981	8.624924	0.834148	0.122801
SNU13	77.21315	64.4437	0.834621	0.046682
SLC35B4	13.74298	11.47054	0.834647	0.350178

NRDE2	3.554338	2.966978	0.834748	0.095448
SUMO1	135.8112	113.3805	0.834839	0.00022
ZNF124	0.414416	0.345994	0.834896	0.349012
NSMCE2	7.709668	6.43721	0.834953	0.379034
FAM131C	2.653057	2.215878	0.835217	0.595119
STYX	3.718551	3.106037	0.835282	0.251507
MKRN3	1.722312	1.438814	0.835396	0.399386
LONRF1	4.75726	3.975998	0.835775	0.140019
NFKB2	13.7979	11.53611	0.836078	0.241215
ARL9	0.02881	0.02409	0.836146	0.880326
RNF215	19.86226	16.61221	0.836371	0.208554
TMEM265	4.884979	4.086638	0.836572	0.315275
CTNND1	26.65321	22.29928	0.836645	0.118891
PDHB	99.92682	83.63204	0.836933	0.477644
PSMD4	234.0137	195.8549	0.836938	0.152961
AMDHD1	0.319957	0.267844	0.837124	0.723921
TSPAN19	0.780502	0.653381	0.837129	0.296667
UBXN4	24.17331	20.23687	0.837157	0.036488
AK3	25.75473	21.56658	0.837384	0.045459
MID1	6.817996	5.709389	0.8374	0.06978
ZCCHC9	25.82046	21.62276	0.837427	0.101625
BRAP	7.768103	6.505566	0.837472	0.326426
FAM220A	11.75857	9.849629	0.837655	0.221285
HSF1	37.56648	31.47836	0.837937	0.144984
VKORC1L1	10.38535	8.706102	0.838306	0.166397
APBB1	13.85118	11.61349	0.838448	0.130148
MID1IP1	37.79665	31.69399	0.83854	0.395085
SLC25A3	233.3249	195.7289	0.838869	0.157146
MGP	0.644809	0.540923	0.83889	0.785792
MPHOSPH	19.33073	16.21663	0.838904	0.175469
SF3B3	33.29037	27.9319	0.839038	0.250175
REXO1	7.228394	6.065121	0.839069	0.18658
TAPT1	5.918765	4.968024	0.839368	0.184033
GNL2	54.65984	45.88036	0.83938	0.004527
FAM160B2	6.48893	5.446765	0.839393	0.161978
CUX1	5.594581	4.697292	0.839615	0.12244
ZNHIT3	16.63604	13.96851	0.839654	0.16711
B9D1	5.017355	4.213381	0.839761	0.472885
GRK2	22.54992	18.93764	0.83981	0.015216
DENND4A	2.372024	1.992553	0.840023	0.53745
ICK	1.710295	1.436797	0.840087	0.474464
NAPG	6.316188	5.306623	0.840162	0.277664
SPAST	3.271157	2.748426	0.8402	0.268951
ARSA	7.375221	6.196869	0.840228	0.137941
COPB1	76.25071	64.07671	0.840343	0.189557
KIF24	1.961461	1.648345	0.840366	0.159836
ZNF45	3.027463	2.544979	0.840631	0.477435
ANKS1B	0.027915	0.023467	0.840677	0.470866
EDIL3	9.709811	8.163599	0.840758	0.376641
ARMCX6	6.602894	5.553163	0.84102	0.222247
C17orf67	1.381638	1.162028	0.841051	0.391942
GPAT4	20.92947	17.60401	0.841111	0.112475
DEPDC5	2.039131	1.71527	0.841177	0.137499
STARD3	14.40416	12.11885	0.841343	0.134765
ANKH	6.991945	5.882799	0.841368	0.11827
ZNF786	2.279929	1.918358	0.841411	0.489008
LYAR	19.1514	16.11488	0.841447	0.539616
OGFOD1	29.56549	24.88227	0.841599	0.303568

BORCS5	1.378013	1.160143	0.841896	0.108813
SPACA9	5.791941	4.878318	0.84226	0.211505
STK4	9.26549	7.805079	0.842382	0.216924
BCAT1	11.06898	9.325547	0.842494	0.556696
OSBPL5	3.768095	3.174835	0.842557	0.09731
FAM91A1	7.853954	6.618152	0.842652	0.361765
PDHA1	45.9996	38.76431	0.84271	0.157573
MC4R	0.026504	0.022336	0.842761	0.880321
LYZ	0.02514	0.021187	0.842761	0.880321
HEXA	36.91852	31.11685	0.842852	0.013629
TMEM256	118.9315	100.2421	0.842855	0.211153
CMAS	55.98273	47.19077	0.842952	0.269721
SMOX	9.51903	8.024347	0.842979	0.580267
LTV1	29.53306	24.89721	0.843028	0.404589
IMMT	49.32858	41.59782	0.84328	0.306681
TMCO1	25.83909	21.79279	0.843404	0.084323
CIPC	5.482929	4.624654	0.843464	0.158126
HLA-DQB	19.29895	16.27844	0.843489	0.573663
NAP1L2	0.926509	0.781518	0.843508	0.706444
DHX15	61.05829	51.53635	0.844052	0.145683
UTP6	47.27096	39.91057	0.844294	0.045901
SEC22C	7.858397	6.634873	0.844304	0.117882
TRAM1	32.68333	27.60324	0.844566	0.100285
GCLC	8.314796	7.023719	0.844725	0.223568
BTN3A1	5.52348	4.666551	0.844857	0.132855
ESD	84.80717	71.66468	0.845031	0.254112
AZI2	18.61237	15.73274	0.845284	0.092043
SCLY	8.474045	7.164271	0.845437	0.414085
NUTF2	61.49865	51.99869	0.845526	0.021017
PTPN23	13.09313	11.07073	0.845537	0.232507
CCDC25	17.05299	14.42039	0.845622	0.291924
DUS4L	6.113765	5.170069	0.845644	0.58814
PRICKLE3	5.155829	4.360094	0.845663	0.325939
FGF18	0.359375	0.303944	0.845759	0.689306
ZNF292	1.386283	1.172585	0.845848	0.548029
DHRS1	8.881544	7.512957	0.845907	0.249387
IPPK	5.043064	4.267643	0.84624	0.330725
BUD31	36.97329	31.29118	0.846318	0.056155
PROS1	15.253	12.90914	0.846334	0.34015
STAB2	0.002198	0.001861	0.846629	0.912459
RGL4	0.00635	0.005376	0.846629	0.912459
TRIM34	0.007785	0.006591	0.846629	0.912459
ACRV1	0.016603	0.014056	0.846629	0.912459
CT55	0.01661	0.014062	0.846629	0.912459
14-Sep	0.00504	0.004267	0.846629	0.912459
OR2AE1	0.02007	0.016992	0.846629	0.912459
SMCO3	0.004091	0.003463	0.846629	0.912459
GRIK1	0.002958	0.002505	0.846629	0.912459
OGN	0.005124	0.004338	0.846629	0.912459
SCN7A	0.00254	0.002151	0.846629	0.912459
CKMT2	0.002647	0.002241	0.846629	0.912459
GPD2	5.325263	4.508529	0.84663	0.336106
POLR3GL	35.44642	30.01009	0.846632	0.208694
MORN3	0.920377	0.779432	0.846861	0.5778
KPNA6	15.86236	13.43345	0.846876	0.128948
NOTCH1	2.565161	2.172724	0.847013	0.203657
LOC11226	0.876849	0.742833	0.847161	0.569097
CX3CR1	0.015697	0.013299	0.847244	0.851031

KDM2A	9.660268	8.184648	0.847249	0.145959
UBE2D3	45.81999	38.82233	0.847279	0.060211
SVBP	21.33677	18.07972	0.84735	0.439487
VAMP1	3.858234	3.269324	0.847363	0.553975
LMOD2	0.016154	0.013691	0.847561	0.884434
DUSP27	0.009105	0.007717	0.847561	0.884434
RNASET2	17.00126	14.41105	0.847646	0.606903
VPS11	6.927441	5.872542	0.847722	0.047269
HAUS1	45.72984	38.78348	0.8481	0.506663
DEDD	11.55494	9.80026	0.848144	0.430509
RFPL4AL1	0.097124	0.082396	0.848355	0.841777
CCT7	254.669	216.1485	0.848743	0.170262
PLSCR4	0.695224	0.590075	0.848756	0.022665
LAMB2	31.54768	26.78217	0.848943	0.317941
BAHD1	7.151333	6.07145	0.848996	0.227738
COPG2	21.408	18.17586	0.849022	0.44798
CARS	46.62044	39.58271	0.849042	0.066706
KLF6	18.89082	16.03924	0.84905	0.478956
SLC31A1	21.16581	17.97257	0.849132	0.184011
AKR1B15	1.068139	0.907186	0.849314	0.682469
NUPR1	43.6456	37.0773	0.849508	0.705113
SPR	12.13569	10.31122	0.849661	0.561804
ARSK	5.268466	4.478349	0.850029	0.151318
EEF1AKM1	5.795076	4.927761	0.850336	0.395969
CLCN7	10.73091	9.128317	0.850656	0.342486
PSTK	3.266801	2.779252	0.850756	0.31499
IRGQ	4.531658	3.857683	0.851274	0.526171
FAM219A	23.1278	19.69013	0.851362	0.25891
NAT10	24.16204	20.58428	0.851926	0.419067
IL5	0.016987	0.014473	0.851994	0.856672
SGF29	7.61869	6.492186	0.852139	0.166618
SAFB2	9.214995	7.852579	0.852152	0.447512
OR5B21	0.041953	0.035763	0.852438	0.89858
MBLAC2	1.096716	0.935126	0.85266	0.673762
FBXL3	15.61176	13.31166	0.852669	0.179057
SLC12A5	0.421038	0.359015	0.85269	0.24736
L1TD1	0.014164	0.012078	0.852725	0.871295
SFXN1	45.69009	38.96587	0.85283	0.061349
UBE2D2	56.39904	48.12385	0.853274	0.047168
ANAPC16	23.26695	19.85417	0.853321	0.423895
LRCH1	3.267847	2.788817	0.853411	0.287086
TEX37	0.080459	0.068679	0.853585	0.854888
JADE1	6.48293	5.535374	0.853838	0.211166
SPPL3	14.63404	12.49564	0.853875	0.070896
INPP5A	2.70694	2.311508	0.853919	0.175942
ZBTB8OS	18.71558	15.98599	0.854154	0.139717
CHST10	4.899579	4.185074	0.85417	0.454475
CTCF	15.66078	13.37844	0.854264	0.235254
NEURL3	0.236402	0.201979	0.854386	0.837201
STARD7	43.30827	37.00911	0.854551	0.234962
CTF1	5.968453	5.101202	0.854694	0.446121
AKR1D1	0.042191	0.036061	0.854712	0.845298
EFL1	16.34451	13.97166	0.854823	0.038857
DDR1	8.763701	7.492003	0.85489	0.045495
TIMM8B	42.59567	36.4198	0.855012	0.355821
C2orf74	24.09521	20.60396	0.855106	0.474827
MSR1	0.010249	0.008765	0.855198	0.860262
SLU7	23.88668	20.42813	0.85521	0.295497

PLCD3	8.63118	7.381752	0.855242	0.165567
ACTR1A	82.86199	70.87121	0.855292	0.186414
CALN1	0.005437	0.004651	0.855331	0.878661
PPP4R3A	19.16861	16.39653	0.855384	0.093152
CYCS	50.10425	42.8632	0.85548	0.136142
STX1B	1.186573	1.015439	0.855774	0.459392
AKAP10	7.314076	6.259586	0.855827	0.212744
PIGK	24.04394	20.57892	0.855888	0.364108
PPIP5K2	3.553148	3.041174	0.85591	0.328711
MISP3	2.704556	2.315085	0.855995	0.543096
RSPH10B2	0.016141	0.013817	0.855995	0.895195
DTX3	8.97949	7.686433	0.855999	0.236169
NDUFB11	156.6313	134.1358	0.856379	0.009152
HSD17B1	0.62089	0.531974	0.856793	0.451668
RNF20	19.32227	16.55654	0.856863	0.01195
ANKRD13	7.569363	6.486879	0.856991	0.212442
PXMP2	38.6731	33.14808	0.857135	0.26342
PI4KB	25.98676	22.27684	0.857238	0.081929
SLC25A11	47.97538	41.14695	0.857668	0.457105
HYDIN	0.011872	0.010186	0.857969	0.823065
PCDHGA5	0.057708	0.049522	0.858138	0.80679
ZNF561	4.647375	3.988291	0.858182	0.444385
ART3	0.004365	0.003746	0.858247	0.919517
GPR26	0.001893	0.001625	0.858247	0.919517
LOC10537	0.014875	0.012766	0.858247	0.919517
AMBP	0.013604	0.011676	0.858247	0.919517
TRAF3IP3	0.005164	0.004432	0.858247	0.919517
C16orf47	0.005658	0.004856	0.858247	0.919518
GTDC1	0.973663	0.835935	0.858546	0.393595
CYP51A1	22.12393	19.00284	0.858927	0.489427
TRNP1	2.816507	2.419522	0.85905	0.65195
EIF2B4	21.15203	18.17396	0.859206	0.191608
HMX3	0.211032	0.18135	0.859347	0.829719
XKR6	0.431113	0.370571	0.859568	0.203253
AKR1A1	49.20663	42.30273	0.859696	0.109588
NUP93	37.44506	32.1936	0.859756	0.147477
MBD5	1.165685	1.002244	0.85979	0.039257
STAT2	9.300221	8.000562	0.860255	0.117607
TRNAU1A	15.26993	13.13704	0.860321	0.227195
FBXO27	3.72947	3.209213	0.860501	0.348055
MAGI2	0.114266	0.098336	0.860589	0.644809
DNAJC1	19.93383	17.15666	0.860681	0.262399
CCL7	0.046763	0.040249	0.860687	0.843188
BTF3	570.6874	491.2439	0.860793	0.18099
AGA	10.95027	9.425938	0.860795	0.455204
WDR78	0.744989	0.641428	0.86099	0.531022
ATP5ME	310.9474	267.8213	0.861307	0.082529
UBE2G1	14.06631	12.11848	0.861525	0.1644
MTERF1	5.344108	4.604849	0.861668	0.237547
DCAF5	7.163375	6.172521	0.861678	0.169435
FAM207A	8.095164	6.977068	0.861881	0.453898
PCYOX1L	8.648457	7.454195	0.86191	0.335129
COX14	28.01714	24.15173	0.862034	0.265385
QPCT	14.85231	12.80473	0.862137	0.273011
SGTA	64.2564	55.40915	0.862313	0.290974
SPATC1L	5.618452	4.845443	0.862416	0.091677
SLC10A5	0.173468	0.14962	0.862521	0.753657
TXNL4B	6.429183	5.546534	0.862712	0.305401

AIMP1	14.2019	12.25257	0.862741	0.363334
APLP1	20.57784	17.75438	0.862791	0.377092
MTHFSD	3.561308	3.072752	0.862816	0.218031
TOB1	5.03993	4.34908	0.862925	0.235813
LOC10798	0.002686	0.002318	0.863246	0.922534
GRIN3A	0.001671	0.001442	0.863246	0.922534
VSX2	0.006212	0.005362	0.863246	0.922534
C6orf58	0.01549	0.013372	0.863246	0.922534
CYP3A4	0.006663	0.005752	0.863246	0.922534
JSRP1	0.01162	0.010031	0.863246	0.922534
DDX53	0.008738	0.007543	0.863246	0.922534
TNNT2	0.036077	0.031144	0.863246	0.922534
ETV3L	0.009335	0.008058	0.863246	0.922534
PDCL2	0.018365	0.015854	0.863246	0.922534
TMEM89	0.056205	0.048519	0.863246	0.922534
TAS2R30	0.038758	0.033458	0.863246	0.922534
LOC10537	0.0148	0.012776	0.863246	0.922534
SPANXN1	0.030349	0.026199	0.863246	0.922534
PRF1	0.014608	0.012611	0.863246	0.922534
CNDP1	0.008464	0.007307	0.863246	0.922534
LMAN1L	0.010717	0.009251	0.863246	0.922534
C6	0.004139	0.003573	0.863246	0.922534
EDN3	0.006812	0.005881	0.863246	0.922534
ERCC6	1.249987	1.079343	0.863483	0.164855
ATP6V1G1	58.79011	50.77586	0.86368	0.362098
ANKRD39	12.73381	10.99859	0.863731	0.210655
SPAG6	0.029459	0.025455	0.864086	0.857334
SLA2	0.017382	0.01502	0.864107	0.865312
TYK2	18.77333	16.22238	0.864119	0.120428
STK16	10.61059	9.16946	0.86418	0.261636
WDR60	6.88118	5.947548	0.864321	0.257812
OAT	20.18726	17.44854	0.864334	0.324115
ACAA2	27.88553	24.10396	0.864389	0.43606
WRB	17.09015	14.77321	0.864428	0.405878
LRRC8C	2.52131	2.180007	0.864633	0.043839
SH2B1	10.11836	8.749919	0.864756	0.293146
SPATA2L	6.570388	5.682648	0.864888	0.153814
RSPH4A	0.067234	0.058155	0.864966	0.652304
WDR89	4.175017	3.612167	0.865186	0.460826
PPP6R3	10.79515	9.344066	0.86558	0.416799
MTERF3	18.47504	15.9968	0.86586	0.206913
TEPSIN	3.422925	2.964651	0.866116	0.392601
GALNT1	21.47593	18.60787	0.866452	0.530341
GNAS	147.0743	127.4415	0.866511	0.141445
PLA2G4C	0.720012	0.623917	0.866536	0.768687
NECAB2	1.853766	1.606441	0.866582	0.538756
SH2D1A	0.015142	0.013124	0.86672	0.900489
TRIM41	13.9755	12.11333	0.866755	0.285845
ZNF513	6.622179	5.740089	0.866798	0.008199
JAZF1	5.42613	4.703977	0.866912	0.326954
RIMS3	9.302031	8.064511	0.866962	0.622746
WRAP53	7.669769	6.650281	0.867077	0.171987
HNF1A	0.013061	0.011325	0.867099	0.871595
JTB	86.84975	75.33687	0.867439	0.202614
DIDO1	7.3777	6.400195	0.867506	0.212516
KRT86	0.054916	0.047645	0.867593	0.802611
REXO4	18.61141	16.14802	0.867641	0.461267
B3GALT2	0.01624	0.014093	0.867785	0.872485

RPL11	1241.959	1077.992	0.867977	0.29305
PSRC1	22.18486	19.25866	0.868099	0.027381
PKIB	4.022091	3.491605	0.868107	0.66732
SLC28A1	0.010556	0.009164	0.868134	0.869261
CCDC187	0.007542	0.006548	0.868283	0.90176
TDRD9	0.00701	0.006087	0.868283	0.90176
IDI2	0.02722	0.023635	0.868283	0.90176
DTX2	2.292489	1.990688	0.868353	0.099896
COQ8B	11.38632	9.8897	0.86856	0.626447
DPP8	6.526619	5.670855	0.868881	0.357138
ANO6	9.435158	8.199433	0.86903	0.165294
TUFM	134.7535	117.1093	0.869064	0.280784
PHF1	19.02134	16.53205	0.869132	0.705916
TPGS2	20.04152	17.41963	0.869177	0.279558
NAE1	62.67162	54.4844	0.869363	0.402696
TRIM68	5.225583	4.543985	0.869565	0.153896
SP2	2.600077	2.261095	0.869626	0.414902
LAIR1	0.01691	0.014711	0.869975	0.906793
MEGF8	7.885486	6.862893	0.87032	0.606582
ISPD	0.029693	0.025851	0.870607	0.236325
MAPKAPK	3.902318	3.398361	0.870857	0.208694
MOGS	25.74431	22.42687	0.871139	0.334006
SRGAP2C	3.922429	3.417259	0.87121	0.156494
LOC10798	0.062473	0.054429	0.871246	0.845161
ADIPOR1	71.68305	62.46499	0.871405	0.242202
RGPD1	0.004512	0.003932	0.871427	0.913935
UBALD2	33.88877	29.53781	0.871611	0.380233
AVPI1	8.897383	7.75659	0.871783	0.23339
HS3ST3B1	2.574816	2.244968	0.871895	0.484849
PFAS	7.285895	6.354121	0.872113	0.629842
SGK3	0.012214	0.010654	0.872219	0.874786
DAXX	40.2002	35.06741	0.872319	0.159166
RAET1L	0.052777	0.046044	0.872427	0.917064
TMEM237	9.767801	8.523879	0.872651	0.085507
SRC	9.770502	8.526808	0.872709	0.069524
RHOA	412.6904	360.3209	0.873102	0.419343
SMG8	7.773028	6.787952	0.87327	0.410133
SV2B	0.001273	0.001112	0.873379	0.928609
C19orf67	0.006579	0.005746	0.873379	0.928609
FANCD2C	0.021344	0.018642	0.873379	0.928609
LOC10272	0.015355	0.013411	0.873379	0.928609
LOC11226	0.027383	0.023916	0.873379	0.928609
LOC10537	0.015743	0.01375	0.873379	0.928609
SPINK7	0.033433	0.029199	0.873379	0.928609
APOA5	0.019035	0.016625	0.873379	0.928609
CA5A	0.006107	0.005334	0.873379	0.928609
HEMGN	0.007174	0.006266	0.873379	0.928609
CYP2C19	0.004447	0.003884	0.873379	0.928609
HTR3E	0.007288	0.006365	0.873379	0.928609
NBPF7	0.004919	0.004296	0.873379	0.928609
FAM71D	0.006525	0.005699	0.873379	0.928609
PIK3CG	0.00227	0.001983	0.873379	0.928609
SCML4	0.001378	0.001203	0.873379	0.928609
LOC10798	0.116284	0.101577	0.873532	0.813444
TEX49	0.035908	0.031369	0.873602	0.849188
HIST1H2A	0.863363	0.754536	0.87395	0.854146
MED8	42.98474	37.56706	0.873963	0.292257
KLKB1	0.044947	0.039283	0.873986	0.814177

ZNRF1	3.658006	3.198677	0.874432	0.022591
DCAKD	13.68454	11.96929	0.874658	0.372114
FAM177A1	9.918152	8.676916	0.874852	0.325345
TTC27	8.314846	7.274945	0.874934	0.663926
ARFGAP2	19.89128	17.4082	0.875167	0.298719
UCHL3	22.15246	19.38711	0.875167	0.18259
FKBPL	8.421753	7.370971	0.87523	0.344648
LOC11226	0.021797	0.019084	0.875517	0.914709
HIST1H2A	0.078004	0.068294	0.875517	0.914709
SLC6A3	0.009458	0.008281	0.875517	0.914709
LOC11226	0.056266	0.049266	0.8756	0.852027
NAT2	0.085012	0.074438	0.875613	0.780448
ELF1	3.940779	3.450696	0.875638	0.56043
SLC9A9	0.88724	0.777112	0.875876	0.622787
TLK1	6.711533	5.878731	0.875915	0.148234
ANKRD40	13.45721	11.78828	0.875983	0.14254
ACTR8	4.821197	4.224204	0.876173	0.249176
HCK	0.024632	0.021584	0.876246	0.837715
PIK3R2	27.7222	24.29335	0.876314	0.254635
SLC11A2	8.766807	7.683445	0.876425	0.266508
ARHGAP1	2.545347	2.231483	0.876691	0.587573
PPP4R4	3.1107	2.727337	0.87676	0.424543
DBF4	11.33042	9.935847	0.876918	0.588432
TFB1M	5.458154	4.787075	0.87705	0.564519
HLA-F	4.430056	3.885802	0.877145	0.408959
CERS6	5.331205	4.677039	0.877295	0.308812
HSPA6	0.341043	0.299242	0.87743	0.668819
LOC10798	0.117495	0.103104	0.877517	0.923698
C2orf42	2.970198	2.606866	0.877674	0.303618
TRIM56	2.749618	2.413409	0.877725	0.475874
SCN2A	0.172833	0.151706	0.877765	0.481461
NLK	5.470492	4.80262	0.877914	0.091809
ZNF248	1.646519	1.445979	0.878204	0.0278
EEFSEC	5.819143	5.110435	0.878211	0.537268
UPF2	7.872878	6.91447	0.878265	0.032914
MKKS	18.28553	16.06126	0.878359	0.398305
WDR45B	30.00669	26.35884	0.878432	0.262809
CCDC194	0.028742	0.025253	0.878613	0.910232
GYS2	0.010184	0.008951	0.878887	0.881997
GATD3A	2.689458	2.364507	0.879176	0.751127
SDHC	37.22043	32.7235	0.879181	0.320486
GLI2	1.217307	1.07031	0.879244	0.284904
SUCLG1	86.67785	76.2143	0.879282	0.090251
C5	0.493571	0.434001	0.879308	0.764673
GLUD2	0.17648	0.155221	0.879542	0.851253
RBM8A	31.93359	28.09136	0.879681	0.069496
BCLAF3	1.325423	1.166044	0.879752	0.220348
GLS	5.379734	4.733571	0.879889	0.28101
TMEM14C	91.01588	80.10584	0.88013	0.152669
AAED1	8.097878	7.128852	0.880336	0.474526
ITM2B	109.0814	96.03217	0.880372	0.051214
ZNF230	2.282827	2.009825	0.88041	0.444922
PNRC2	32.38189	28.5133	0.880532	0.067881
DET1	4.948969	4.357845	0.880556	0.652492
TARDBP	47.28764	41.63974	0.880563	0.295814
POMZP3	4.281006	3.769718	0.880568	0.650218
CD164L2	0.022597	0.0199	0.880626	0.86653
LYRM4	5.484984	4.830496	0.880676	0.190121

HINT2	47.78564	42.0893	0.880794	0.575551
IHH	0.027268	0.024023	0.88098	0.825813
WBP11	46.45215	40.92432	0.880999	0.296545
RANBP1	78.87948	69.49393	0.881014	0.087333
WWC1	4.891251	4.309627	0.881089	0.607987
C8orf59	44.17123	38.92328	0.881191	0.459028
FARSA	75.32109	66.38304	0.881334	0.467738
TNPO3	42.42544	37.39726	0.881482	0.023783
FAM122A	5.810847	5.122723	0.881579	0.602838
SH3BP5	12.70224	11.19823	0.881595	0.251057
DWORF	0.046802	0.041261	0.881608	0.918956
DPYS	0.00445	0.003923	0.881608	0.918956
DOC2B	0.013428	0.011838	0.881608	0.918956
SLC14A2	0.006636	0.00585	0.881608	0.918956
C22orf15	0.019071	0.016813	0.881623	0.884448
SLC25A40	4.713708	4.156056	0.881696	0.275051
PPP1R9B	21.06414	18.57353	0.881761	0.050268
RNF168	8.397226	7.405377	0.881884	0.542645
ZCCHC6	4.27308	3.769531	0.882158	0.474813
SNRPD2	466.1332	411.4278	0.88264	0.262618
TBRG4	38.39523	33.89326	0.882746	0.244803
CDC123	80.85392	71.37765	0.882798	0.491805
CASD1	1.541796	1.361255	0.882902	0.573502
SLC15A4	8.116092	7.167707	0.883148	0.462265
CFDP1	68.52914	60.52712	0.883232	0.406895
HNRNPD	157.0438	138.7388	0.88344	0.35407
MICU1	26.50166	23.41467	0.883517	0.208896
DDAH2	18.35297	16.21754	0.883647	0.674793
PLEKHJ1	18.5645	16.40585	0.883721	0.45498
PACS2	9.396369	8.30407	0.883753	0.25992
PTPN3	3.393842	2.999853	0.88391	0.00121
PGP	13.56632	11.99225	0.883972	0.315884
WWP1	8.764214	7.747721	0.884018	0.171108
YME1L1	31.40786	27.77261	0.884257	0.054885
GALM	8.256803	7.30301	0.884484	0.319421
TAOK2	10.01528	8.858457	0.884494	0.533522
WDR46	16.45734	14.55772	0.884573	0.521564
EWSR1	46.23617	40.90356	0.884666	0.485218
THRAP3	26.84582	23.75789	0.884975	0.412262
HGD	0.044629	0.039496	0.884991	0.833788
PGS1	4.087195	3.617159	0.884998	0.191541
PAPSS1	24.21826	21.43317	0.885001	0.341263
METTL2B	14.25233	12.61358	0.885019	0.23946
VGLL4	12.16047	10.76235	0.885027	0.515545
SSBP4	17.43517	15.43206	0.885111	0.316842
SPIDR	7.434721	6.583229	0.885471	0.23778
COX17	155.8968	138.0703	0.885652	0.321362
MIP	0.012416	0.010998	0.885794	0.921864
SLC25A47	0.019965	0.017685	0.885794	0.921864
LEP	0.010495	0.009297	0.885794	0.921864
AMD1	45.86041	40.62626	0.885868	0.333104
YES1	11.78844	10.44342	0.885904	0.120914
MAPK3	74.05625	65.61236	0.88598	0.092013
AGPAT1	32.62323	28.90398	0.885994	0.130802
LMO1	0.410191	0.363427	0.885996	0.764517
PTPN9	14.3653	12.73057	0.886203	0.092509
ANO7	0.378165	0.335143	0.886233	0.660498
CREM	2.920306	2.588634	0.886426	0.46661

FBXO2	3.395297	3.009759	0.886449	0.752643
NEK8	0.680826	0.603618	0.886597	0.501393
LRRC20	8.420965	7.466329	0.886636	0.425184
SRPRB	38.0418	33.73009	0.886659	0.478832
CREB3L2	16.31002	14.46269	0.886736	0.226207
GRK7	0.015916	0.014115	0.886841	0.907068
HDAC8	9.39132	8.329453	0.886931	0.440493
TCEAL1	18.24441	16.18184	0.886947	0.350852
MCAT	10.9926	9.750097	0.886969	0.566938
BTLA	0.012766	0.011325	0.887099	0.855076
RPS6KA1	9.543213	8.466118	0.887135	0.464214
KIF2C	44.5658	39.53626	0.887144	0.167773
LZTFL1	7.73166	6.859627	0.887213	0.453491
ARL6	2.965726	2.631402	0.887271	0.231246
NBPF19	1.444132	1.281392	0.88731	0.52058
MOSMO	4.43805	3.93821	0.887374	0.470264
FAM118B	3.913723	3.47411	0.887674	0.369817
TRMT1	11.16401	9.910226	0.887694	0.630551
C4orf54	0.001717	0.001524	0.887788	0.937157
LAX1	0.003496	0.003103	0.887788	0.937157
PCP4L1	0.010943	0.009715	0.887788	0.937157
C1orf189	0.026351	0.023394	0.887788	0.937157
ZNF878	0.014931	0.013255	0.887788	0.937157
ZNF197	0.014579	0.012947	0.888088	0.856993
CYSLTR2	0.011015	0.009785	0.888385	0.891134
BEX3	211.2096	187.6396	0.888405	0.072864
DPH6	1.748676	1.553573	0.888428	0.677674
NAT14	38.96684	34.6223	0.888507	0.438686
MRPS22	44.0109	39.10608	0.888554	0.549149
TBC1D3B	0.02464	0.021905	0.889002	0.90668
CYP4V2	1.707417	1.517952	0.889034	0.714417
CD302	0.037414	0.033264	0.889066	0.895695
NIPA1	5.547336	4.932795	0.889219	0.268983
MED11	15.83744	14.08338	0.889246	0.525142
BCKDHB	1.924445	1.711566	0.889382	0.628687
JMJD7	1.958151	1.74159	0.889405	0.606997
BCL2L12	14.7307	13.10718	0.889787	0.537057
FPGT	2.286468	2.03449	0.889796	0.379797
BORCS6	4.656616	4.144397	0.890002	0.308629
ANAPC7	16.36201	14.56299	0.890049	0.24541
ATP6V0E2	34.13573	30.38635	0.890163	0.250233
CUL3	12.16867	10.83516	0.890414	0.061508
COQ7	7.183732	6.396736	0.890447	0.472027
AP2A2	21.14925	18.83984	0.890804	0.286443
EFHC1	7.962129	7.093571	0.890914	0.132302
OSBPL3	3.45747	3.080411	0.890944	0.331275
RNF185	21.3672	19.03708	0.890949	0.33112
TBC1D3H	0.014018	0.012493	0.891235	0.923437
FAM72C	1.501291	1.338032	0.891254	0.449447
LCK	0.036949	0.032939	0.891487	0.886124
IPO5	63.7237	56.82032	0.891667	0.346021
DEPDC1B	5.233542	4.667498	0.891843	0.464628
MED25	15.93484	14.21237	0.891906	0.101271
CST2	0.052991	0.047266	0.891957	0.926152
SLC25A17	24.17242	21.56113	0.891972	0.553753
ZNF823	3.149038	2.809076	0.892043	0.134405
HAO2	0.018197	0.01624	0.892411	0.923816
PRPF18	2.548303	2.274573	0.892583	0.208186

STRADA	9.056682	8.085312	0.892746	0.07494
NUS1	19.376	17.29844	0.892777	0.350346
MED15	15.87491	14.18456	0.89352	0.269088
EPHX1	27.86707	24.90354	0.893654	0.643676
TACO1	23.902	21.36035	0.893664	0.540436
SFR1	5.082491	4.542427	0.89374	0.569589
DDX31	3.547813	3.171386	0.893899	0.327829
MIOS	4.56921	4.084972	0.894021	0.664368
HIBCH	13.24357	11.84329	0.894267	0.625285
BTBD9	0.86711	0.775612	0.89448	0.531687
ZNF324B	1.792999	1.603893	0.894531	0.494984
MRM3	14.82903	13.2697	0.894846	0.695238
SMUG1	8.498283	7.605077	0.894896	0.543658
GUK1	91.02879	81.46944	0.894985	0.285266
FNIP1	3.953053	3.538004	0.895005	0.503488
SLC36A1	0.819945	0.73393	0.895097	0.52249
MTSS1L	15.31144	13.70555	0.895118	0.496252
CWC15	29.74246	26.62341	0.895131	0.490691
BRAT1	11.81566	10.57763	0.895221	0.024669
CCDC6	9.34233	8.364131	0.895294	0.351085
BBS9	2.169019	1.94219	0.895423	0.152751
WHAMM	4.298327	3.849075	0.895482	0.513463
INPP4B	0.568881	0.509476	0.895575	0.467002
TRAM1L1	2.15186	1.927759	0.895857	0.759737
INTS3	18.95798	16.98459	0.895907	0.497523
SRPRA	34.30386	30.73391	0.895932	0.589741
DEDD2	17.60172	15.77426	0.896177	0.588788
OGFRL1	4.619201	4.139654	0.896184	0.534666
RYR3	0.005877	0.005269	0.896396	0.91621
NFKBIB	23.54475	21.10641	0.896438	0.509036
ZMAT5	35.4606	31.79784	0.896709	0.122208
MTRR	6.607425	5.926748	0.896983	0.278718
SAMD4B	9.484697	8.508847	0.897113	0.61173
PRCP	21.2869	19.09756	0.897151	0.379457
LIX1L	15.68155	14.0737	0.897469	0.121043
GGCX	6.460861	5.799823	0.897686	0.398202
COP1	4.543786	4.078991	0.897708	0.623046
NMRAL1	7.045394	6.325232	0.897783	0.480557
DEPDC1	18.70944	16.80125	0.898009	0.571605
PBK	42.16398	37.86628	0.898072	0.231313
MEDAG	0.005248	0.004714	0.898209	0.943272
LOC10798	0.01055	0.009476	0.898209	0.943272
APOB	0.001302	0.00117	0.898209	0.943272
PCDHGA1	0.00382	0.003432	0.898209	0.943272
MFSD6L	0.016418	0.014747	0.898209	0.943272
CA4	0.013393	0.012029	0.898209	0.943272
HNRNPCL	0.015716	0.014116	0.898209	0.943272
F7	0.004549	0.004086	0.898209	0.943272
PLD4	0.0171	0.015359	0.898209	0.943272
PPIAL4C	0.023424	0.02104	0.898209	0.943272
RNASE13	0.011442	0.010278	0.898209	0.943272
NUTM2F	0.00718	0.006449	0.898209	0.943272
SSRP1	127.9019	114.8857	0.898234	0.299716
RAC1	173.8055	156.1835	0.89861	0.182137
RIPK2	17.90997	16.10038	0.898962	0.392149
UBE2R2	28.48421	25.61121	0.899137	0.019616
DBN1	24.60795	22.12788	0.899216	0.206541
TMEM99	7.656507	6.884989	0.899234	0.700107

CDK4	140.6138	126.4456	0.89924	0.361568
NUDCD2	5.175628	4.654213	0.899256	0.594329
ARRDC1	3.473458	3.123718	0.899311	0.451112
KNL1	4.686484	4.214748	0.899341	0.472869
CBLB	2.731018	2.456953	0.899647	0.116946
JUND	105.1559	94.60915	0.899704	0.546102
ACTR1B	33.0361	29.72425	0.899751	0.311686
TBC1D23	18.2825	16.45022	0.899779	0.059517
MAGOH	89.13608	80.20523	0.899806	0.425053
DNAJB14	3.394679	3.054688	0.899846	0.440398
LRRC3B	0.001569	0.001412	0.89997	0.9443
RFPL3	0.008192	0.007372	0.89997	0.9443
RIPPLY1	0.015686	0.014117	0.89997	0.9443
CALHM3	0.011261	0.010135	0.89997	0.9443
HIST1H1A	0.023821	0.021438	0.89997	0.9443
TCEAL2	0.016897	0.015207	0.89997	0.9443
DCANP1	0.005934	0.005341	0.899971	0.9443
CCNC	28.26662	25.44064	0.900024	0.25056
SLC35B3	4.935728	4.442477	0.900065	0.382283
PTPN11	42.52032	38.27434	0.900142	0.437223
MRFAP1	170.3285	153.343	0.900278	0.077971
GMPS	21.41748	19.28626	0.900492	0.470329
SUGP1	9.168861	8.259188	0.900787	0.348274
CDCA7L	26.9685	24.29848	0.900995	0.649009
PDCD6IP	28.11201	25.33749	0.901305	0.058355
INO80B	21.48554	19.36611	0.901355	0.478384
KLHL2	3.543107	3.19554	0.901903	0.393776
CARM1	36.20802	32.6622	0.902071	0.478413
ATG14	4.428903	3.996357	0.902335	0.592897
RPS12	2390.192	2156.755	0.902336	0.243852
CAPRIN1	51.99002	46.91396	0.902365	0.122443
LRWD1	16.64181	15.01712	0.902373	0.638226
ZNF512B	4.032989	3.639735	0.902491	0.547801
DARS	35.75153	32.27639	0.902798	0.54699
PPID	29.81683	26.91896	0.902811	0.289346
OTUD6B	4.605955	4.159252	0.903016	0.400779
REEP2	40.08509	36.2033	0.903161	0.21192
DDB1	91.60698	82.74828	0.903297	0.151367
UBR7	26.43515	23.88078	0.903372	0.177409
BIRC5	82.18627	74.25031	0.903439	0.211555
TRIM28	125.0236	112.9705	0.903594	0.458019
FH	36.21136	32.72392	0.903692	0.554631
SET	93.89912	84.87622	0.903909	0.143089
ERH	329.1264	297.5732	0.90413	0.415567
BANK1	0.010078	0.009115	0.904372	0.934754
BCCIP	36.88139	33.35948	0.904507	0.601699
SEC11A	128.8711	116.6252	0.904976	0.220909
MBD1	7.923087	7.172119	0.905218	0.424394
RNF26	24.67171	22.33576	0.905319	0.434452
STXBP6	2.214556	2.005007	0.905376	0.79386
TRIM14	1.999672	1.810537	0.905417	0.413962
TMUB1	58.63087	53.08852	0.905471	0.219433
ADSL	27.4222	24.83447	0.905634	0.673286
HMCES	29.7647	26.95734	0.905682	0.399038
KPNA3	12.75322	11.55313	0.905899	0.02026
CDC27	17.78773	16.11704	0.906076	0.35643
DPY19L3	5.334458	4.83417	0.906216	0.579872
FGGY	0.886996	0.804011	0.906443	0.394501

NUP98	13.36864	12.11884	0.906513	0.36252
OAZ3	0.708795	0.642774	0.906854	0.736894
NBAS	9.68697	8.785113	0.9069	0.264991
TDRD7	4.349295	3.944556	0.906941	0.578974
CUBN	0.105669	0.09584	0.906984	0.752831
EGLN3	3.564056	3.232706	0.90703	0.78534
PIGM	2.490125	2.259298	0.907303	0.760908
UBA3	42.23775	38.32285	0.907313	0.269224
NXF1	14.89141	13.51265	0.907412	0.59994
ALG14	2.582925	2.343975	0.907489	0.688088
LRRFIP2	15.98425	14.50591	0.907512	0.146048
SERINC5	2.839014	2.576746	0.90762	0.067953
WWOX	2.506326	2.274805	0.907626	0.572705
SS18L2	13.12261	11.91254	0.907787	0.402157
MNT	3.736747	3.392775	0.907949	0.465073
RPAP1	10.32957	9.379514	0.908026	0.555446
NAXD	11.7837	10.70177	0.908184	0.408367
NAA30	7.206343	6.544951	0.908221	0.300897
C16orf95	6.971679	6.332137	0.908266	0.608064
MINDY2	2.785037	2.529587	0.908278	0.526221
AKT2	15.95303	14.49076	0.908339	0.206167
LCA5L	0.232517	0.211217	0.908396	0.835898
LRRIQ3	1.382901	1.256353	0.908491	0.728096
CTTNBP2N	5.848811	5.314192	0.908594	0.404164
DUS2	9.186485	8.347324	0.908653	0.564137
BCAS4	4.967568	4.515228	0.908941	0.331813
PI4K2A	14.62137	13.29097	0.90901	0.417438
DCTN1	51.41422	46.73753	0.909039	0.328109
SH3D19	3.400489	3.091504	0.909135	0.747501
MVB12A	33.29537	30.27306	0.909227	0.226466
PTS	37.77489	34.36416	0.909709	0.545947
RPL22L1	113.8877	103.6232	0.909872	0.688038
SIK1	0.134152	0.122069	0.909926	0.838394
MIF	1385.285	1261.087	0.910345	0.503595
SIK1B	0.386152	0.351563	0.910426	0.651044
PCDHA8	0.003462	0.003153	0.910535	0.950432
SH2D1B	0.007254	0.006605	0.910535	0.950432
TTC24	0.005634	0.00513	0.910535	0.950432
FUT2	0.005934	0.005403	0.910535	0.950432
PENK	0.013651	0.01243	0.910535	0.950432
CLEC19A	0.007373	0.006713	0.910535	0.950432
OXT	0.028289	0.025758	0.910535	0.950432
FOXD4L3	0.016581	0.015097	0.910535	0.950432
LRRC14B	0.014399	0.013111	0.910535	0.950432
DMP1	0.005045	0.004593	0.910535	0.950432
MYL1	0.015976	0.014546	0.910535	0.950432
FAM231BP	0.004529	0.004124	0.910535	0.950432
NR1I3	0.010098	0.009194	0.910535	0.950432
TAF1L	0.002958	0.002694	0.910535	0.950432
ZNF181	1.311938	1.194595	0.910557	0.718068
TBCE	22.41785	20.41394	0.910611	0.58635
UBE2E1	48.32198	44.00763	0.910717	0.507203
PRDX6	210.1857	191.4462	0.910843	0.043576
SNX25	2.638083	2.403145	0.910944	0.313095
CASK	4.549564	4.144583	0.910985	0.556277
ERCC5	0.233821	0.213163	0.911651	0.715845
PUS1	11.41904	10.4117	0.911785	0.743374
MRPS35	30.60534	27.90581	0.911795	0.23003

DHPS	63.24807	57.68909	0.912108	0.088625
MAFK	10.69516	9.755357	0.912128	0.800604
RNF103	4.183828	3.816985	0.912319	0.541329
ZMYND19	18.96293	17.30297	0.912463	0.214066
B3GNT5	1.957906	1.786948	0.912684	0.291817
TXN2	58.07064	53.00307	0.912734	0.021327
LRRC47	15.32034	13.98463	0.912815	0.092282
SLC39A4	4.496506	4.105818	0.913113	0.410454
ARID1A	7.260016	6.63205	0.913504	0.356501
PPP2R5C	26.82849	24.51159	0.91364	0.155786
CENPI	7.235315	6.610767	0.913681	0.209402
MEN1	16.18393	14.78901	0.913808	0.587783
RASSF1	11.13585	10.17763	0.913952	0.711993
RTKN	6.238207	5.702679	0.914154	0.517772
ZNF44	0.945017	0.86416	0.914439	0.661896
MCM5	53.43335	48.86222	0.914452	0.406307
MPP2	5.834184	5.335827	0.91458	0.36099
HNRNPK	233.2846	213.3802	0.914678	0.413152
DECR2	8.844765	8.090463	0.914718	0.368106
SPDL1	17.65436	16.14906	0.914735	0.764652
CFAP20	32.2196	29.4801	0.914974	0.113221
MRPL39	13.86982	12.69168	0.915057	0.568145
FAM200B	4.066259	3.722207	0.915389	0.03882
AVL9	6.242393	5.714373	0.915414	0.454108
CACYBP	47.35496	43.35517	0.915536	0.672183
URI1	23.77543	21.7727	0.915765	0.432953
NCOA4	27.43902	25.1445	0.916377	0.71861
SLC30A9	25.84315	23.69536	0.916891	0.604947
ZNF317	8.221776	7.538711	0.91692	0.54771
AKAP8	6.757152	6.197254	0.91714	0.35842
LRIF1	5.301399	4.86262	0.917233	0.760695
SLX1A	1.328326	1.218729	0.917493	0.738912
ETFRF1	5.32939	4.890668	0.917679	0.703104
TMOD4	0.203213	0.186562	0.91806	0.899686
GIT1	17.2819	15.86764	0.918165	0.433134
SATB2	4.307012	3.954985	0.918266	0.620905
UFC1	50.43135	46.31834	0.918443	0.485227
SAP18	49.64674	45.59816	0.918452	0.273451
SEPHS1	17.30854	15.89935	0.918584	0.322453
SLC41A3	21.86778	20.0885	0.918634	0.212171
FBXO30	2.223863	2.043322	0.918816	0.638812
SLC25A39	82.07917	75.42512	0.918931	0.412338
IPO11	9.602299	8.824691	0.919019	0.638251
KIAA1143	10.44438	9.598972	0.919056	0.60802
RFTN2	0.250302	0.230049	0.919085	0.861636
ZSCAN25	1.701424	1.563768	0.919094	0.722956
TCTN1	8.525317	7.835861	0.919128	0.614816
TAB3	3.200168	2.941742	0.919246	0.35391
IPP	2.709312	2.490566	0.919262	0.576512
RNF14	9.858	9.063169	0.919372	0.469963
ANO8	3.444274	3.166626	0.919389	0.469267
LMBR1	10.16824	9.351605	0.919688	0.294191
SPAG5	37.22382	34.23558	0.919722	0.15523
PFDN5	335.4445	308.6754	0.920198	0.545807
SENP2	14.4654	13.32638	0.921259	0.424839
DCP1B	5.211143	4.800916	0.921279	0.61361
PTPN14	4.44403	4.094281	0.921299	0.557833
NDC80	34.69439	31.97492	0.921617	0.576414

BIVM	5.978835	5.510545	0.921675	0.487868
RAD51B	0.909262	0.838226	0.921876	0.640919
MOB1A	27.22804	25.10167	0.921905	0.43652
TACC3	47.68129	43.96061	0.921968	0.226174
SCG2	1.059163	0.976609	0.922058	0.81104
CHCHD2	410.4091	378.4426	0.922111	0.054475
HAT1	29.11728	26.85062	0.922154	0.455633
BTG3	38.17784	35.2201	0.922527	0.488028
TFB2M	6.482225	5.983389	0.923046	0.68428
IP6K1	11.19904	10.33774	0.923091	0.342984
RMI1	4.739498	4.375075	0.923109	0.68843
FRYL	2.546185	2.350467	0.923133	0.533635
SKIDA1	0.19677	0.18166	0.92321	0.712304
ARNT2	6.08257	5.615533	0.923217	0.752335
U2AF2	70.98226	65.53838	0.923306	0.410064
FAM3B	0.107225	0.099014	0.923418	0.918127
PALLD	10.74597	9.923579	0.92347	0.692568
LSM7	134.7613	124.4772	0.923687	0.212099
APLP2	143.6216	132.666	0.923719	0.075243
C1orf54	20.6086	19.03736	0.923758	0.425148
RPS20	914.5506	845.1642	0.924131	0.408024
RBKS	1.433242	1.324629	0.924218	0.797199
CSTF2	15.24231	14.09518	0.924741	0.562047
YAF2	1.494043	1.381862	0.924915	0.082943
WIPI2	18.43186	17.0486	0.924953	0.51119
NAA50	36.95252	34.19888	0.925482	0.397041
ZNF189	2.078208	1.923694	0.92565	0.825056
PLK3	4.887799	4.525087	0.925792	0.82619
PRPF38A	20.78996	19.24908	0.925883	0.311317
AP1AR	3.848031	3.564624	0.92635	0.520331
CAP2	4.495573	4.164702	0.926401	0.649688
GPN3	29.46739	27.29897	0.926413	0.62457
RAD17	14.26356	13.22029	0.926857	0.452397
EGLN1	9.822694	9.104504	0.926885	0.755239
BTBD1	38.7489	35.917	0.926916	0.579851
DDX20	11.73609	10.87845	0.926922	0.631018
SDAD1	13.94791	12.9306	0.927063	0.752066
LSM6	22.10537	20.49995	0.927374	0.649425
TBCA	87.53738	81.1802	0.927378	0.065718
FAM166A	0.406082	0.376596	0.92739	0.866149
RAB5A	33.62313	31.19675	0.927836	0.164651
WASF2	33.85799	31.41622	0.927882	0.585857
SSFA2	8.989975	8.341955	0.927917	0.488162
MMP16	0.921914	0.855484	0.927943	0.767288
APTX	5.967548	5.539593	0.928286	0.614792
HDAC4	0.40681	0.377827	0.928754	0.518476
WDR45	29.01224	26.94571	0.92877	0.720062
F2R	7.455015	6.924406	0.928825	0.672473
LOC10012	0.437732	0.406587	0.928849	0.816723
ZCRB1	45.67858	42.42954	0.928872	0.232014
SPG7	12.223	11.35661	0.929118	0.352253
RPA3	45.09069	41.90833	0.929423	0.636723
TMEM47	8.552992	7.949924	0.92949	0.751866
HNRNPU	84.65216	78.70533	0.92975	0.589281
VMAC	1.121708	1.043387	0.930177	0.691569
SETX	4.611386	4.290109	0.93033	0.555882
AGO1	2.558328	2.380907	0.93065	0.646269
KCNAB3	0.475599	0.442653	0.930727	0.84434

MAP9	2.998586	2.791227	0.930848	0.600499
PSMC6	25.61449	23.8467	0.930985	0.60116
EIF2B1	45.29854	42.17385	0.93102	0.203558
PNRC1	25.06288	23.33471	0.931046	0.38968
NME1	58.19726	54.20627	0.931423	0.719854
TPM2	14.83144	13.81468	0.931446	0.704951
PFN4	0.301256	0.280618	0.931493	0.881225
COX7B	462.6483	431.0185	0.931633	0.32193
EIF3I	250.6745	233.5449	0.931666	0.180088
HPF1	48.54527	45.24688	0.932055	0.375428
FBXO7	79.22811	73.84526	0.932059	0.566101
ATP5PO	302.8756	282.349	0.932227	0.454938
HERC2	4.720144	4.400477	0.932276	0.71253
LDLRAD2	0.067668	0.063087	0.9323	0.878703
RUSC1	9.709293	9.0559	0.932704	0.574411
ORC3	16.63233	15.51913	0.93307	0.69276
APOBEC3(	0.297134	0.277283	0.933191	0.77093
MEX3C	10.7161	10.0037	0.933521	0.502666
CMSS1	55.25367	51.59155	0.933722	0.769305
ANKLE2	27.96269	26.11354	0.933871	0.314163
ENOSF1	6.111473	5.70826	0.934024	0.724705
FKBP11	8.607051	8.041502	0.934292	0.713228
OSTC	122.2034	114.1744	0.934298	0.057761
NXPE3	4.852951	4.53425	0.934328	0.397885
ATP5MC3	53.87717	50.35434	0.934614	0.50033
FRK	0.115473	0.107925	0.934638	0.859456
PPP4R3B	9.786814	9.147218	0.934647	0.662533
SMG6	3.006629	2.810613	0.934805	0.670927
SSH3	8.418132	7.870236	0.934915	0.45487
CCDC12	5.83594	5.456508	0.934984	0.001826
DTWD2	1.152811	1.077868	0.934991	0.800364
ENSA	46.92185	43.87987	0.935169	0.632783
VPS50	2.063754	1.930141	0.935257	0.698943
VPS25	85.10133	79.59205	0.935262	0.211067
TMEM258	288.648	269.9996	0.935394	0.233792
RPE	16.98281	15.89184	0.93576	0.608193
APPBP2	5.655401	5.292578	0.935845	0.804246
NUFIP1	5.551361	5.196524	0.936081	0.717074
TMTC1	1.783253	1.669365	0.936135	0.551757
METTL23	13.90284	13.01784	0.936344	0.695452
SLC22A23	3.65	3.418235	0.936503	0.551698
STXBP3	22.65374	21.21928	0.936679	0.499068
MAP2K2	91.47113	85.6909	0.936808	0.448031
SLC39A1	50.23084	47.05987	0.936872	0.421537
GUF1	7.235095	6.780351	0.937147	0.734735
YIPF3	54.43821	51.01694	0.937153	0.537628
FAU	527.5398	494.4062	0.937192	0.346382
ZNF720	2.885749	2.705066	0.937388	0.524286
DIMT1	10.36862	9.72012	0.937455	0.683367
FOXJ2	5.813555	5.451478	0.937719	0.621528
SEC31A	46.90751	44.00978	0.938225	0.671918
SCAMP4	35.0061	32.84552	0.93828	0.705212
LBX2	1.671772	1.568947	0.938493	0.809395
POFUT2	15.55962	14.61215	0.939108	0.867646
CCDC69	2.946369	2.767049	0.939139	0.778511
PRKRA	24.319	22.84048	0.939203	0.605054
SLC35F2	11.83875	11.12222	0.939476	0.775021
C19orf57	1.168293	1.097657	0.939539	0.414328

USP51	0.27809	0.261287	0.939576	0.85624
ARMH3	5.187679	4.874534	0.939637	0.491406
ASAH2B	3.24707	3.051885	0.939889	0.798624
UBE2W	3.496581	3.287255	0.940134	0.361598
TKFC	4.335481	4.07607	0.940166	0.825986
BTBD7	2.49515	2.346071	0.940252	0.513058
RAPH1	4.974054	4.680945	0.941072	0.640124
COMMD2	12.13661	11.42214	0.941131	0.576938
PLD5	0.023432	0.022061	0.941467	0.880763
IFI27L1	12.01032	11.30751	0.941483	0.255389
RFFL	7.280383	6.855298	0.941612	0.523453
RELL2	3.866051	3.641865	0.942012	0.341727
STRAP	91.1832	85.90415	0.942105	0.518547
SP3	9.381754	8.839821	0.942235	0.490423
HNRNPAE	199.7537	188.2354	0.942338	0.310118
LOC73026	1.927865	1.816867	0.942424	0.651788
GNE	10.67628	10.06407	0.942657	0.713753
RASSF3	6.028197	5.683769	0.942864	0.705244
GNAI1	8.543123	8.055596	0.942933	0.622428
FAM90A1	0.265695	0.250539	0.942957	0.820283
TRMT5	5.792602	5.462219	0.942965	0.711751
MTMR2	8.596574	8.109243	0.943311	0.766164
FAM214A	2.215275	2.090243	0.943559	0.806397
RRAGB	3.890542	3.672428	0.943938	0.7572
PYGO1	0.846336	0.799088	0.944174	0.700243
ZNF766	3.189552	3.011922	0.944309	0.488708
FAM104B	6.125905	5.785929	0.944502	0.666927
ZNF414	9.993734	9.440585	0.94465	0.674395
TRAF1	1.675322	1.582829	0.944791	0.439707
STARD4	2.848525	2.691829	0.94499	0.81038
MRC2	23.13903	21.86623	0.944993	0.732485
APBA3	8.579554	8.109741	0.94524	0.56279
ATP6V0D2	1.082894	1.023801	0.945431	0.921055
SIL1	17.79179	16.82531	0.945679	0.543168
ZNF646	3.904182	3.693281	0.945981	0.724565
POLRMT	23.50323	22.24626	0.946519	0.362384
GFOD2	3.631294	3.437914	0.946746	0.78844
SND1	64.37381	60.9493	0.946803	0.569329
DNAJC7	65.48245	62.0192	0.947112	0.819647
FMNL2	5.67345	5.37354	0.947138	0.410911
ITGAX	0.47872	0.453419	0.947148	0.888417
HDHD2	6.882387	6.519473	0.947269	0.687093
ARMC10	9.26948	8.781526	0.947359	0.42937
PITHD1	38.90007	36.85957	0.947545	0.614637
LSM4	154.7311	146.6284	0.947634	0.501197
GPR75	0.192942	0.182954	0.948235	0.929704
MRPS27	36.6781	34.78951	0.948509	0.813753
CPSF3	29.01005	27.51852	0.948586	0.802784
ARL5A	6.970758	6.612791	0.948647	0.626121
PLXNA1	8.550356	8.113615	0.948921	0.743902
KPNA2	160.1943	152.0319	0.949047	0.218845
ZNF335	5.51914	5.238006	0.949062	0.760026
PCNT	4.285766	4.067721	0.949124	0.658215
ZNF131	6.005771	5.700303	0.949137	0.677516
MAPK1IP1	17.64004	16.75367	0.949753	0.626597
PIP5KL1	1.724006	1.63794	0.950078	0.777083
GID8	17.61314	16.73464	0.950122	0.111828
ELAC2	20.48245	19.46102	0.950131	0.591002

KIAA0753	2.86853	2.72592	0.950284	0.669328
ZNF480	1.765794	1.678563	0.950599	0.810192
TIMM29	12.01053	11.42209	0.951006	0.524009
RAP1A	6.88833	6.551488	0.9511	0.424561
RNF220	17.41357	16.56357	0.951188	0.435332
SRSF9	204.7478	194.754	0.95119	0.413964
GATAD2A	17.05019	16.22097	0.951366	0.396296
MCRS1	27.45052	26.11689	0.951417	0.663643
MELK	36.90223	35.10944	0.951418	0.495234
MOXD1	19.43476	18.4933	0.951558	0.797317
TRAF3	4.876463	4.641243	0.951764	0.728573
NLGN1	0.348451	0.331738	0.952037	0.859869
RTEL1	4.283055	4.078014	0.952127	0.725294
SRP19	25.09033	23.89412	0.952324	0.492615
GABPB1	4.175616	3.977445	0.952541	0.75446
PPME1	23.49278	22.38192	0.952715	0.314177
CRYM	1.483897	1.414011	0.952903	0.908314
KIF4A	21.56261	20.55245	0.953152	0.785499
RGS12	1.462003	1.393695	0.953277	0.581988
DDX47	34.69617	33.08352	0.953521	0.225331
VEZF1	12.90395	12.30508	0.953591	0.766378
DESI1	20.12947	19.1956	0.953607	0.754314
CAB39	8.03353	7.660907	0.953617	0.705852
PCDHGB6	0.040746	0.038874	0.954048	0.915064
ZNF491	0.130369	0.124385	0.9541	0.921643
ETAA1	8.141109	7.767468	0.954104	0.7883
HIF1AN	11.02437	10.52553	0.954751	0.729179
ATP9B	0.578239	0.552174	0.954925	0.539892
MINPP1	9.285123	8.868171	0.955095	0.746211
KIAA1586	4.804558	4.589171	0.95517	0.727784
SLC39A9	19.73154	18.85072	0.95536	0.646134
ZBTB42	2.128071	2.033076	0.955361	0.781957
LOC10272	0.422227	0.403411	0.955435	0.632988
SERPINH1	78.1609	74.69617	0.955672	0.738403
GRB2	39.427	37.68927	0.955925	0.502368
TSPAN31	18.22747	17.42868	0.956177	0.445802
ADAM15	21.373	20.43916	0.956308	0.59396
EYA4	3.825018	3.658212	0.956391	0.769139
MKL2	1.44749	1.38445	0.956449	0.794265
SKA1	12.61896	12.07033	0.956524	0.55971
SMIM30	53.02336	50.73162	0.956779	0.602092
SSBP2	3.240902	3.100854	0.956788	0.675019
AKIRIN1	39.3745	37.68132	0.956998	0.446578
DIABLO	29.3973	28.13767	0.957151	0.578981
RTN3	45.72479	43.77841	0.957433	0.627795
ERFE	10.53115	10.08935	0.958048	0.542769
ZNF710	3.237711	3.102153	0.958132	0.682293
LMAN1	70.87817	67.91094	0.958136	0.743227
PNKD	12.7109	12.179	0.958154	0.54226
DNAJC19	17.17597	16.46506	0.958611	0.756952
B3GNT2	5.781895	5.542623	0.958617	0.583563
MMADHC	94.19538	90.33246	0.95899	0.737887
CIAPIN1	32.04337	30.73684	0.959226	0.733284
CEP128	1.51136	1.450185	0.959523	0.596462
AK6	23.94157	22.97824	0.959764	0.548164
IDH3G	41.65572	39.98427	0.959875	0.519272
HSPG2	4.600183	4.416801	0.960136	0.878478
POMGNT2	15.77312	15.14507	0.960182	0.345896

GLRX2	13.47224	12.9448	0.96085	0.774438
ZBED5	16.44784	15.80416	0.960865	0.716038
SDHA	31.74182	30.4996	0.960865	0.525954
PAFAH1B1	20.14305	19.35514	0.960885	0.645666
RPL28	82.22706	79.01613	0.96095	0.788846
FAM234A	23.38474	22.473	0.961011	0.595895
QTRT1	19.85819	19.08972	0.961302	0.760651
DCLRE1B	9.761574	9.385063	0.961429	0.638757
JMJD8	19.05462	18.32116	0.961507	0.870745
RAI1	3.585298	3.44774	0.961633	0.815467
GSPT1	33.48777	32.21334	0.961944	0.687755
HNRNPF	45.00808	43.30951	0.962261	0.767224
NT5C	21.75046	20.93439	0.962481	0.70356
EHBP1	6.553426	6.30877	0.962667	0.771827
BACE1	6.399174	6.160724	0.962737	0.834486
ZMIZ2	8.625679	8.307376	0.963098	0.453693
UST	3.577977	3.446381	0.96322	0.656638
ASB8	8.192167	7.891144	0.963255	0.642143
ZNF626	0.024739	0.023833	0.963375	0.948213
ERCC6L2	1.355834	1.306192	0.963386	0.60507
ADGRF3	0.022791	0.021959	0.963479	0.958762
SH2D5	1.043634	1.005529	0.963488	0.913083
PI4KA	16.37518	15.78446	0.963926	0.76936
TOPORS	8.152646	7.86005	0.96411	0.64937
BEND3	0.665234	0.641414	0.964193	0.801842
NCDN	16.29927	15.71617	0.964225	0.795842
MRPL19	7.485167	7.218203	0.964334	0.466586
SERP1	58.67354	56.58551	0.964413	0.720093
IFT88	1.822599	1.757946	0.964527	0.880587
C17orf82	0.099949	0.096408	0.964571	0.955645
PINX1	8.069534	7.789272	0.965269	0.858929
CHD9	2.762846	2.667577	0.965518	0.781628
PCNX1	4.595296	4.438317	0.965839	0.836915
CPNE3	17.15114	16.56564	0.965862	0.693465
SETD1B	2.361507	2.281532	0.966134	0.87639
ENDOV	2.769032	2.675417	0.966192	0.800949
EPM2A	0.766168	0.74031	0.96625	0.826111
XPNPEP1	31.35075	30.30043	0.966497	0.79797
SCCPDH	21.95351	21.22327	0.966737	0.690105
MYO1B	14.55255	14.0701	0.966848	0.683756
RMDN1	7.588553	7.338174	0.967006	0.698238
CDK19	2.918979	2.822761	0.967037	0.880091
WARS	48.92362	47.31167	0.967052	0.903593
MVD	24.73915	23.93098	0.967332	0.847527
MAP2K4	8.312435	8.041133	0.967362	0.627957
FBXW8	4.139862	4.005052	0.967436	0.723935
MED6	17.98696	17.41282	0.96808	0.834422
TMEM160	24.25958	23.48678	0.968144	0.688839
SELENOW	64.96576	62.89846	0.968179	0.686152
LURAP1	1.687792	1.634284	0.968297	0.741771
C9orf152	0.056707	0.054917	0.968436	0.952729
NFX1	9.0985	8.811791	0.968488	0.43485
ANAPC5	73.00899	70.71647	0.968599	0.817378
PPIH	17.30178	16.75913	0.968636	0.49739
SLC12A4	12.67236	12.27794	0.968876	0.849469
NAA40	7.605105	7.368594	0.968901	0.828291
ENTPD6	8.340996	8.082676	0.96903	0.78862
TMEM161	2.02607	1.963361	0.969049	0.834118

DOPEY2	2.387409	2.314502	0.969462	0.873361
ARL6IP1	109.185	105.8519	0.969473	0.590484
CAMK2A	0.238635	0.231351	0.969478	0.877392
UTP11	43.70504	42.37704	0.969615	0.791079
FARP2	2.01706	1.955974	0.969715	0.760615
MRPL53	63.79083	61.87024	0.969892	0.718402
WDR83	13.49312	13.09091	0.970191	0.675481
ZC2HC1A	0.975284	0.946568	0.970557	0.825426
APOBR	0.075158	0.07296	0.970758	0.962001
PRSS36	0.16657	0.161736	0.97098	0.909308
PPP1R21	6.070609	5.894462	0.970984	0.868964
CCDC71	11.93087	11.58994	0.971425	0.906759
USP48	12.89445	12.5262	0.971441	0.619844
MANBA	6.958405	6.763522	0.971993	0.767886
MPND	7.833022	7.613691	0.971999	0.895795
HSPA5	378.5608	367.9802	0.97205	0.789264
LAPTM5	0.267673	0.260285	0.972399	0.960988
FN3KRP	23.19691	22.55945	0.97252	0.61276
C1GALT1	3.421981	3.32924	0.972898	0.765383
FAM171A1	10.56929	10.28592	0.973189	0.679099
OPA1	12.82078	12.48097	0.973496	0.157777
PRR13	82.33006	80.16968	0.97376	0.812701
PRKCE	0.442116	0.430529	0.973791	0.821386
MPP5	7.721512	7.520707	0.973994	0.655252
P2RX7	0.656603	0.639558	0.974041	0.870007
GEMIN6	35.22456	34.31566	0.974197	0.881851
UBL7	41.49397	40.42474	0.974232	0.82727
ZNF775	6.293754	6.131672	0.974247	0.853809
UPP1	13.69541	13.34557	0.974455	0.818017
BVES	3.975963	3.875549	0.974745	0.748085
IMP4	13.43562	13.09788	0.974862	0.765118
MFSD4B	1.988046	1.938078	0.974866	0.804083
C19orf47	1.927423	1.879658	0.975218	0.746033
MS4A15	0.171399	0.167163	0.975286	0.956286
CORO1A	1.715651	1.673289	0.975309	0.942444
SMAD4	5.815018	5.671752	0.975363	0.553221
CFAP97	3.571776	3.48378	0.975363	0.861366
FBXW2	10.0063	9.76369	0.975755	0.649671
SPG11	5.177994	5.053941	0.976042	0.871531
MADCAM	0.085624	0.08359	0.976243	0.981928
RDH14	0.991413	0.967868	0.976251	0.932753
ATP6AP1	72.27193	70.56609	0.976397	0.850851
UTP23	4.955714	4.838748	0.976398	0.804614
DCXR	55.80714	54.5138	0.976825	0.795779
NUDT14	6.598245	6.447679	0.977181	0.910432
ZGLP1	0.714279	0.698251	0.97756	0.922063
KIFC2	6.317846	6.17652	0.977631	0.844917
TYSND1	3.432561	3.35592	0.977672	0.949688
GRM8	0.046112	0.04509	0.977834	0.887094
LSM5	30.96144	30.28133	0.978034	0.851706
CLCC1	12.3646	12.09327	0.978056	0.830111
DAGLB	10.82609	10.58874	0.978076	0.821489
LYSMD4	1.397276	1.366912	0.978269	0.800748
STAT5A	0.163932	0.160424	0.978603	0.922837
SIRT2	16.4989	16.14653	0.978642	0.771129
EMC2	13.25803	12.97516	0.978664	0.841294
DNAJC28	1.362378	1.333353	0.978695	0.947043
PLSCR1	7.569049	7.40824	0.978754	0.897066

LRRC58	8.427589	8.249201	0.978833	0.80887
PSMA4	190.0468	186.0309	0.978869	0.894966
METTL15	3.086734	3.022301	0.979126	0.869961
MTA2	44.83433	43.90197	0.979204	0.867333
ZNF37A	1.317412	1.290413	0.979506	0.889046
SIGMAR1	83.74659	82.03581	0.979572	0.901028
CSTF3	19.42366	19.03137	0.979803	0.83901
MSMO1	26.66905	26.13791	0.980084	0.967227
UBE2Q2L	0.330842	0.324566	0.98103	0.967611
ACSL1	11.10898	10.89875	0.981076	0.855896
NBR1	17.8258	17.49449	0.981414	0.875141
STEAP2	1.263239	1.239837	0.981475	0.829467
NBPF14	1.107126	1.087278	0.982072	0.883544
SMPD4	13.3739	13.13431	0.982085	0.871411
GPR157	1.426434	1.401022	0.982184	0.956801
HSDL2	22.33634	21.94392	0.982431	0.91529
SHQ1	4.446746	4.370903	0.982944	0.919728
KIAA0100	24.45655	24.04379	0.983123	0.76502
FKBP10	124.0994	122.0179	0.983227	0.823776
ZDHHC24	3.516387	3.457721	0.983316	0.809638
SPTSSA	11.93359	11.73513	0.983369	0.872027
C16orf72	12.38133	12.1764	0.983448	0.854474
BPGM	14.71425	14.47115	0.983479	0.892875
UQCR10	145.7943	143.3883	0.983497	0.899675
ZSWIM5	0.55512	0.545966	0.98351	0.957251
C19orf25	8.082622	7.95197	0.983835	0.943067
ATP5F1A	183.0265	180.0728	0.983862	0.811227
PPP1R2B	0.35933	0.353577	0.983989	0.958688
DOK7	0.102544	0.100923	0.984187	0.985358
EIF3B	81.45664	80.18116	0.984342	0.869953
RAB3D	5.518547	5.433212	0.984537	0.92898
TTI2	5.447891	5.36382	0.984568	0.904581
C11orf58	35.25403	34.71203	0.984626	0.934072
TRIM16L	3.816271	3.760277	0.985328	0.92984
C9orf163	0.160423	0.158078	0.985382	0.975907
SDCCAG3	26.08362	25.70674	0.985551	0.902912
DHRS4L2	4.341263	4.278595	0.985565	0.934284
RNF111	3.395311	3.347106	0.985803	0.934109
HEBP1	36.20025	35.69499	0.986043	0.895465
UMPS	7.020891	6.926846	0.986605	0.934669
LSM3	217.5303	214.7557	0.987245	0.751376
NRAS	30.38332	29.99671	0.987276	0.840335
LOC11226	0.352235	0.34785	0.987551	0.933825
TTC7A	3.280412	3.239793	0.987617	0.831212
CCDC9	13.10253	12.94132	0.987696	0.936021
CYB5R1	24.04393	23.75368	0.987928	0.906497
FAM69A	5.120463	5.060371	0.988264	0.930887
HACL1	16.7165	16.52071	0.988288	0.966227
COQ6	6.160069	6.088558	0.988391	0.901659
KLHL36	4.513973	4.461719	0.988424	0.89142
EFCAB8	1.870963	1.851064	0.989365	0.964482
PCGF1	18.22268	18.02941	0.989394	0.923098
ERO1A	25.18953	24.93093	0.989734	0.924039
MASP1	0.082516	0.081688	0.989963	0.976446
MAGEE1	1.654203	1.637697	0.990022	0.937207
RNF34	19.53488	19.34169	0.99011	0.8267
DOCK1	6.062498	6.004283	0.990398	0.935424
RAP2A	14.57698	14.43724	0.990414	0.90614

CHPT1	34.10635	33.78011	0.990435	0.93519
CHCHD7	17.04599	16.88602	0.990616	0.910607
ZPR1	25.38929	25.15208	0.990657	0.924192
ECPAS	18.43495	18.2771	0.991437	0.902496
TRAF3IP1	4.344866	4.307713	0.991449	0.95325
LPCAT3	13.86013	13.74451	0.991658	0.969515
ZNF433	0.546708	0.542223	0.991795	0.94587
MPPE1	2.714906	2.692765	0.991844	0.951035
HNRNPDL	81.93916	81.29021	0.99208	0.937016
PGBD4	1.049737	1.041465	0.99212	0.979798
ST3GAL3	3.41607	3.390672	0.992565	0.939801
ARHGEF39	3.120081	3.097163	0.992655	0.982746
METTL26	56.9852	56.57586	0.992817	0.92844
LLGL1	16.45008	16.33965	0.993287	0.935181
ANKDD1B	0.044933	0.044648	0.993649	0.987157
LRRFIP1	12.10607	12.0304	0.993749	0.965038
HARBI1	2.337262	2.322801	0.993813	0.975494
HDGFL2	56.69924	56.34861	0.993816	0.936244
SLC9A5	3.213424	3.193557	0.993817	0.934601
UTP18	24.19403	24.0477	0.993952	0.957553
SCUBE3	0.345723	0.343668	0.994056	0.97694
TMEM214	35.62751	35.4192	0.994153	0.976687
DDX49	21.89465	21.77385	0.994483	0.972054
CTNNB1	76.78808	76.44049	0.995473	0.963665
SLC30A6	4.736612	4.715514	0.995546	0.98223
MRPL24	91.46125	91.05601	0.995569	0.984368
LOC10537	0.693144	0.69011	0.995622	0.988622
HAUS2	10.18666	10.14567	0.995976	0.96072
FBXO42	5.036455	5.017349	0.996206	0.973832
TSPO	92.79131	92.44843	0.996305	0.980748
DMGDH	0.193715	0.193054	0.996589	0.994131
NCAPG2	25.42251	25.33677	0.996628	0.945581
PTAFR	0.05	0.049851	0.997033	0.997491
CCDC90B	7.694369	7.674687	0.997442	0.989186
ELP6	8.832416	8.810295	0.997495	0.993308
NME7	13.84346	13.81203	0.997729	0.994123
FYCO1	4.719785	4.709207	0.997759	0.979805
SCAMP2	29.2466	29.21283	0.998845	0.990078
LONP2	7.902686	7.896181	0.999177	0.983637
APH1A	46.97263	46.93584	0.999217	0.993496
CLCN3	10.25147	10.24391	0.999263	0.996747
ADO	12.89275	12.88456	0.999364	0.991638
ZNF148	2.776659	2.775276	0.999502	0.993788
RBM4B	7.380081	7.376539	0.99952	0.99672
SAMHD1	8.796046	8.792194	0.999562	0.997729
GDF11	3.699272	3.697905	0.99963	0.998396
RUFY1	12.56454	12.56041	0.999671	0.996857
SIVA1	45.48275	45.47691	0.999872	0.998785
MFAP2	16.45204	16.45028	0.999893	0.998266
CCDC189	3.669601	3.672533	1.000799	0.994766
GMPPA	48.90233	48.9532	1.00104	0.993055
LMNB2	66.28702	66.41289	1.001899	0.986567
SRRD	11.84722	11.86978	1.001904	0.991056
FXR1	22.72788	22.77645	1.002137	0.987946
JAGN1	28.78467	28.85572	1.002468	0.992236
PILRA	0.512152	0.513703	1.003028	0.994563
RUVBL1	42.41785	42.54723	1.00305	0.990973
MYPOP	6.293227	6.313963	1.003295	0.981028

POLI	1.651752	1.657936	1.003744	0.974932
GTF2F1	59.7202	59.94712	1.0038	0.975539
BCDIN3D	1.846043	1.853254	1.003906	0.990391
HIST1H2B	0.487815	0.489747	1.003961	0.992399
ALDH3A2	15.30186	15.36794	1.004319	0.981738
PCIF1	20.30524	20.39385	1.004364	0.872525
APOO	19.71588	19.80242	1.004389	0.977563
HTRA2	12.98991	13.04703	1.004397	0.974562
MUM1	9.79224	9.839743	1.004851	0.964094
SERTAD2	6.424472	6.456302	1.004955	0.93033
MAST4	0.363338	0.36515	1.004987	0.970913
C9orf24	0.135596	0.136358	1.005616	0.990957
ITPA	7.64377	7.687951	1.00578	0.965458
EMC8	22.94732	23.08836	1.006146	0.939105
CCDC157	0.928715	0.93468	1.006422	0.979887
SNTB2	4.736251	4.766704	1.00643	0.959089
AIG1	5.834769	5.873532	1.006643	0.971533
NDUFA3	107.3941	108.1222	1.00678	0.948536
MAPK1	22.97043	23.13362	1.007104	0.942525
TRIP10	46.03526	46.38942	1.007693	0.962863
RAD50	11.89058	11.9824	1.007722	0.972644
KAZALD1	2.357199	2.375583	1.007799	0.943645
RWDD1	61.3006	61.79444	1.008056	0.927804
MANEA	2.995315	3.020077	1.008267	0.881223
NUCB2	34.9702	35.26543	1.008442	0.915985
AKAP11	5.06083	5.104519	1.008633	0.955373
MAGEF1	33.07251	33.36415	1.008818	0.941111
SNW1	53.3305	53.80337	1.008867	0.805073
UBAC2	10.45731	10.55237	1.00909	0.946017
GDAP2	2.586456	2.610119	1.009149	0.933259
VRK1	34.76809	35.08697	1.009172	0.962811
RHBDF1	7.502924	7.572573	1.009283	0.957118
IL16	0.086684	0.087504	1.009464	0.991007
HDAC3	45.88388	46.33797	1.009896	0.913997
GPS1	28.64698	28.93166	1.009938	0.830992
DHX35	4.887132	4.937092	1.010223	0.889044
GLIPR2	6.04082	6.103512	1.010378	0.942721
MRPL9	54.3523	54.92469	1.010531	0.939666
ABCA13	0.040953	0.041392	1.010716	0.984328
RAB12	13.30138	13.44479	1.010782	0.855928
NSMF	11.4255	11.54907	1.010815	0.91706
HACD2	6.336582	6.408532	1.011355	0.770295
YTHDF3	7.240802	7.323191	1.011378	0.916541
NDE1	5.451861	5.515509	1.011675	0.882865
EIF2AK2	6.099854	6.172905	1.011976	0.905093
CLPP	61.29748	62.04421	1.012182	0.83529
DELE1	5.64811	5.71727	1.012245	0.91118
AMZ2	33.25111	33.66926	1.012575	0.950462
SACM1L	13.20342	13.37047	1.012652	0.908083
CDV3	43.07472	43.63506	1.013009	0.686978
ZNHIT1	56.30134	57.03817	1.013087	0.780223
HEATR3	6.540437	6.627049	1.013243	0.949604
SEC24C	29.77246	30.17926	1.013664	0.931164
RAD23A	120.477	122.1335	1.01375	0.896917
MARVELD	10.96633	11.12084	1.01409	0.94482
SKIL	3.667317	3.719187	1.014144	0.949659
TNK2	6.234931	6.325285	1.014492	0.913139
BABAM2	30.88986	31.3463	1.014776	0.948855

CASQ2	0.041901	0.042533	1.015086	0.972224
ZNF207	47.23539	47.95193	1.01517	0.82757
TTC3	13.23249	13.43432	1.015253	0.921307
ALKBH3	29.59619	30.05464	1.01549	0.937866
FAM35A	7.321723	7.435332	1.015517	0.873291
OCIAD1	41.64275	42.29576	1.015681	0.887452
THRB	0.936532	0.951266	1.015733	0.953687
INSIG1	68.74837	69.83233	1.015767	0.978836
LRRC56	0.105099	0.106758	1.015784	0.924345
LOC10192	0.088087	0.089523	1.016305	0.947045
DNAJC8	149.2386	151.6803	1.016361	0.85119
ZDHHC20	9.078037	9.231002	1.01685	0.677736
CCT6A	112.1492	114.0485	1.016936	0.896791
RPS19BP1	73.47924	74.72837	1.017	0.90518
ZNF304	2.563639	2.608122	1.017352	0.930601
YARS2	14.02998	14.27572	1.017515	0.892291
PPM1G	103.1685	104.9888	1.017644	0.862892
PHF20L1	3.124311	3.179959	1.017811	0.854074
SLC1A5	66.55617	67.75263	1.017977	0.834097
FXR2	25.02508	25.48237	1.018273	0.915047
HJURP	22.26355	22.67246	1.018367	0.840119
INTS9	5.455959	5.556859	1.018493	0.885893
FIP1L1	13.45461	13.70568	1.01866	0.638373
CLEC16A	2.656883	2.708342	1.019368	0.845293
CHMP4A	21.88513	22.3092	1.019377	0.922359
LOC72802	0.130097	0.132619	1.019388	0.98302
RNASEH2/	106.7347	108.8378	1.019704	0.799479
TOR1AIP1	9.350368	9.536908	1.01995	0.818304
PIH1D2	0.602284	0.614458	1.020213	0.957076
ESYT2	19.91108	20.32057	1.020566	0.828334
ZNF844	0.0643	0.06564	1.020845	0.966898
ZNF410	27.335	27.91897	1.021363	0.853853
NEU1	21.22557	21.68206	1.021507	0.943307
FOXP1	2.958034	3.022307	1.021728	0.831984
GRINA	73.26549	74.85967	1.021759	0.747939
PGRMC1	122.2817	124.9497	1.021819	0.833276
CDKN1B	14.22826	14.5405	1.021945	0.877338
PRKAG1	34.78145	35.54894	1.022066	0.910693
OSBPL2	8.084471	8.263043	1.022088	0.854292
CAMK1D	1.276	1.304892	1.022642	0.857288
ITPR2	1.59155	1.627936	1.022862	0.764767
FLT3LG	2.824833	2.889689	1.022959	0.864333
CHCHD5	8.516896	8.713465	1.02308	0.808729
BAG2	4.667831	4.776008	1.023175	0.877528
WDR6	33.557	34.33898	1.023303	0.867237
NEMP2	1.883768	1.927821	1.023386	0.91138
LAMTOR2	87.68112	89.74118	1.023495	0.839535
PRIM2	6.209106	6.356109	1.023675	0.830786
TRMU	13.46777	13.78714	1.023714	0.891331
GABARAP	350.4252	358.786	1.023859	0.828927
CNOT2	10.65004	10.91715	1.025081	0.797055
UHRF1	10.38017	10.64495	1.025508	0.786459
IMPDH1	20.8369	21.37298	1.025728	0.833682
CAMKK1	2.777879	2.849426	1.025756	0.847672
CDC25B	16.28354	16.70384	1.025811	0.745407
HLCS	1.97118	2.022143	1.025854	0.918875
DOCK7	10.41552	10.68531	1.025902	0.83883
RANBP3	21.76183	22.33406	1.026295	0.628335

LRRC4	0.073613	0.075558	1.026419	0.974972
ALX1	3.188437	3.273115	1.026558	0.821786
KLF4	4.163099	4.273846	1.026602	0.910461
TARBP2	13.07126	13.41927	1.026624	0.864086
TMEM231	6.978032	7.166886	1.027064	0.76374
LRP11	13.02196	13.37476	1.027093	0.790597
TMEM60	26.89949	27.63322	1.027277	0.626095
MTMR9	2.42599	2.492416	1.027381	0.837802
ITPR1	3.195834	3.283491	1.027429	0.901174
CTTNBP2	0.690418	0.709634	1.027832	0.850654
UBA52	180.5877	185.6569	1.02807	0.790341
ARHGEF7	5.187281	5.333295	1.028148	0.774248
ATG16L1	8.689583	8.934485	1.028183	0.784832
TBCK	2.713235	2.791358	1.028793	0.891399
EPG5	2.143542	2.205316	1.028818	0.864245
GSDMC	0.023263	0.023943	1.029194	0.961564
PREP	8.043901	8.281218	1.029503	0.871551
SMDT1	20.40068	21.00315	1.029532	0.809675
TEF	4.888247	5.034049	1.029827	0.646698
MBTPS1	24.28927	25.01667	1.029947	0.528093
DAD1	232.2917	239.3134	1.030228	0.486296
PTEN	8.528932	8.78684	1.030239	0.652608
TUBG1	61.24639	63.0985	1.03024	0.747159
RERE	4.23272	4.360733	1.030244	0.829574
TVP23B	9.548887	9.837812	1.030257	0.833203
ULBP3	3.728958	3.842907	1.030558	0.818675
B4GAT1	29.56761	30.47658	1.030742	0.859987
MCC	1.568224	1.616483	1.030773	0.80152
HEXIM1	7.928045	8.173919	1.031013	0.603694
MRPS16	34.53776	35.60962	1.031034	0.819708
KIAA1468	1.965886	2.027297	1.031238	0.813289
HSPA9	148.5635	153.21	1.031276	0.837407
HLA-C	121.8781	125.6979	1.031342	0.74532
FADS1	19.28613	19.89284	1.031458	0.605445
TMEM70	14.12843	14.57538	1.031634	0.837853
FBXO9	9.691226	9.998844	1.031742	0.752097
HNRNPR	30.23114	31.19168	1.031773	0.684343
ZNF425	1.001719	1.033558	1.031784	0.934586
CDK20	5.640112	5.819477	1.031802	0.615822
MITD1	15.87051	16.37577	1.031836	0.662911
TMED2	159.6897	164.8584	1.032367	0.765658
AFG3L2	23.39033	24.15242	1.032582	0.813011
CPSF2	19.05479	19.67726	1.032667	0.899933
ABCA12	0.020071	0.020729	1.032783	0.949301
ACTRT3	0.157908	0.163152	1.033212	0.952848
CCNH	19.91057	20.57339	1.03329	0.792332
TLCD1	2.039739	2.107892	1.033412	0.906025
SLC39A8	6.235206	6.444052	1.033495	0.816581
PALM2	0.514207	0.531477	1.033587	0.878524
UBXN8	5.300247	5.479213	1.033766	0.770831
CCDC30	0.204633	0.21159	1.033996	0.907806
UTP14A	13.49606	13.96121	1.034465	0.847186
TSN	27.92551	28.89045	1.034554	0.802076
FAM3A	22.32884	23.10057	1.034562	0.576627
TNFRSF10	24.4756	25.32383	1.034656	0.81742
FAM209A	0.115093	0.119108	1.034882	0.978978
LOXL4	0.845584	0.875187	1.035009	0.942472
PRADC1	17.89087	18.51789	1.035046	0.731742

TRIM5	6.560182	6.790757	1.035148	0.582137
PTCD2	6.148418	6.364718	1.03518	0.92412
ZC3H18	18.6893	19.35058	1.035382	0.583089
C22orf34	0.004691	0.004858	1.035541	0.967005
PIR	9.797606	10.14648	1.035608	0.794246
TTL	15.0518	15.58825	1.03564	0.831857
PIN4	20.27872	21.00241	1.035687	0.677353
RIDA	27.86374	28.88069	1.036497	0.856019
HAGHL	4.224057	4.378629	1.036593	0.887787
ETF1	58.08135	60.21809	1.036789	0.750734
TMEM11	26.02303	26.99023	1.037167	0.763509
SLC25A38	14.5944	15.13866	1.037292	0.848352
LAMTOR4	63.65697	66.03844	1.037411	0.513275
COX16	1.385173	1.437251	1.037596	0.841039
PURB	7.761222	8.054274	1.037759	0.782628
SPTY2D1	6.292799	6.532132	1.038033	0.762528
RAB14	24.38504	25.3126	1.038038	0.57889
DHRS4	8.785373	9.119737	1.038059	0.815302
TMEM41A	8.286259	8.603581	1.038295	0.732813
SMAD6	1.999893	2.076817	1.038464	0.939726
NPB	0.316805	0.329079	1.038743	0.969693
C1RL	3.867501	4.018359	1.039006	0.83602
ZBTB48	3.8548	4.005183	1.039012	0.776641
ACD	20.04312	20.82877	1.039198	0.524802
ZNF778	2.028293	2.107841	1.039219	0.828255
TCAIM	3.757877	3.905579	1.039305	0.828771
ZNF587B	1.426594	1.482954	1.039507	0.837934
EPS8L2	0.838555	0.871958	1.039834	0.91623
LRRC57	5.274298	5.486086	1.040155	0.794973
GPAA1	45.67652	47.51758	1.040307	0.758711
ODC1	64.10851	66.69651	1.040369	0.820964
TRIP12	10.37087	10.78979	1.040395	0.711095
DDX1	63.67436	66.25616	1.040547	0.772124
RAD23B	64.56065	67.21315	1.041085	0.684488
KXD1	40.33724	42.01045	1.041481	0.728124
ESCO2	3.81725	3.975853	1.041549	0.818919
ZNF18	1.310308	1.364851	1.041626	0.867837
DDT	55.17621	57.47777	1.041713	0.709869
DCTN5	11.20616	11.67611	1.041937	0.640521
SERF1A	0.130992	0.136513	1.042144	0.851965
ADAMTS4	0.371735	0.387479	1.042353	0.899776
SENP3	35.90336	37.42419	1.042359	0.715723
ST7L	2.961637	3.089519	1.043179	0.513832
C9orf40	13.75413	14.34832	1.043201	0.692817
DNAJB6	33.31544	34.75642	1.043253	0.474414
VPS8	5.113754	5.336302	1.043519	0.700112
USP14	22.04815	23.02039	1.044096	0.572628
XRCC1	35.33061	36.88964	1.044127	0.721867
SLC25A51	7.78117	8.125625	1.044268	0.638882
ZC3H7B	18.38342	19.20322	1.044594	0.789508
PDLIM1	90.25893	94.29558	1.044723	0.631276
TEAD1	9.474313	9.898307	1.044752	0.802676
P4HA1	63.40431	66.24891	1.044864	0.637919
PLEKHA6	0.20329	0.212447	1.045044	0.734529
CHSY1	11.88152	12.41816	1.045166	0.6744
TBC1D10E	25.31305	26.45714	1.045198	0.321742
TAF9B	15.21237	15.90071	1.045248	0.840259
SAR1A	43.05987	45.01407	1.045383	0.781599

CINP	53.40141	55.82837	1.045447	0.628732
GALK1	21.49806	22.47639	1.045508	0.868401
CCDC58	42.34623	44.27619	1.045576	0.808559
C12orf4	4.945748	5.171699	1.045686	0.854811
WDFY2	2.651657	2.772917	1.04573	0.832526
CRCP	11.61096	12.14279	1.045804	0.822473
WBP1	49.76312	52.0656	1.046269	0.502832
ZC3HC1	8.807	9.216478	1.046495	0.659602
GDI2	62.01102	64.9128	1.046795	0.57676
ZNF17	2.682876	2.809755	1.047292	0.773233
FAM173A	12.49485	13.0858	1.047296	0.431817
PLPP5	5.226286	5.475204	1.047628	0.86912
CAMTA2	11.16145	11.69317	1.047638	0.202933
PEX11B	16.2989	17.07781	1.047789	0.659672
DENND5A	25.40417	26.61895	1.047818	0.572346
MED26	4.018512	4.210842	1.047861	0.664601
INTS10	19.39356	20.32225	1.047886	0.633064
C9orf147	0.098084	0.102807	1.048143	0.889444
PFKFB3	15.01214	15.74459	1.04879	0.618814
KRI1	17.19865	18.04714	1.049335	0.691472
RNF128	3.350403	3.517692	1.049931	0.824752
DTX4	0.129423	0.13589	1.049971	0.904564
SLC25A24	4.506816	4.732487	1.050073	0.727128
CCDC7	0.147043	0.154431	1.050245	0.858425
MFSD14C	3.885198	4.080425	1.050249	0.537153
FAM227B	0.284005	0.298341	1.05048	0.760083
UBC	638.2345	670.5618	1.050651	0.732701
COG7	9.995009	10.50202	1.050727	0.599905
COX4I1	357.9338	376.3276	1.051389	0.294917
ATXN7L3	10.73209	11.28413	1.051438	0.271337
ZNF639	7.695624	8.091967	1.051502	0.39436
UQCR11	106.9569	112.4735	1.051577	0.684843
SMAP2	22.31137	23.46218	1.051579	0.69466
RAB35	21.4434	22.55109	1.051656	0.504494
DNAL4	16.88293	17.75625	1.051728	0.753408
RPP38	9.608355	10.11071	1.052283	0.798376
SNX24	5.69656	5.998721	1.053043	0.562072
HLA-E	39.14477	41.2232	1.053096	0.693098
ABCB6	7.891715	8.311017	1.053132	0.718496
CKS2	336.911	354.8666	1.053295	0.73518
TMBIM6	175.077	184.414	1.053331	0.676785
PSMB5	91.18962	96.05635	1.053369	0.605384
LY6E	78.31516	82.50942	1.053556	0.714258
RPS6KA3	11.44764	12.06493	1.053923	0.425393
ARHGEF2	11.82419	12.4634	1.05406	0.754624
TPCN2	1.251751	1.319515	1.054135	0.774533
MRPL22	16.6824	17.58619	1.054176	0.736011
MORF4L1	177.5751	187.1956	1.054177	0.338544
ATP8B3	0.724542	0.76393	1.054362	0.824367
GPRIN1	8.93713	9.423404	1.054411	0.666937
SGMS2	1.84841	1.949522	1.054703	0.293514
GRB14	2.199513	2.320007	1.054782	0.935812
MTX2	28.03654	29.5757	1.054899	0.612647
CTDSPL	4.377667	4.618272	1.054962	0.705085
PRPF4	26.0329	27.47614	1.055439	0.146005
PALB2	4.467664	4.715565	1.055488	0.719646
AACS	8.632305	9.123763	1.056932	0.701762
EIF3G	200.7816	212.2255	1.056997	0.329532

PRPS2	24.0732	25.44722	1.057076	0.70262
CCNYL1	6.642792	7.022723	1.057195	0.692627
DGUOK	66.13915	69.92852	1.057294	0.377028
ATP2A2	30.28205	32.02554	1.057575	0.532981
C12orf43	9.586112	10.13887	1.057662	0.716255
VPS37B	8.287119	8.766605	1.057859	0.567653
SUCLG2	24.81	26.2462	1.057888	0.744084
MRPL40	30.9095	32.71638	1.058457	0.682839
SLC6A8	26.32158	27.87021	1.058835	0.75544
HERC6	2.299745	2.435813	1.059166	0.773862
MRRF	8.036368	8.512673	1.059269	0.597716
KBTBD7	0.918548	0.973003	1.059285	0.851683
CXCL1	3.420901	3.625692	1.059865	0.897146
PTPMT1	15.91141	16.86427	1.059885	0.354116
SKI	4.5922	4.868607	1.060191	0.52186
FAM213A	5.971192	6.331784	1.060389	0.578085
GABARAP	104.9596	111.3259	1.060655	0.679737
CXXC1	13.67338	14.50347	1.060709	0.726196
FOXM1	32.41008	34.38196	1.060841	0.638612
TSTD3	3.027935	3.212191	1.060852	0.739915
ZNF155	1.53619	1.630015	1.061076	0.804774
GSTZ1	8.230267	8.736301	1.061485	0.558154
ZNF462	0.934914	0.992531	1.061628	0.468334
ZFPL1	28.64481	30.41178	1.061685	0.619643
MRM2	22.30091	23.67658	1.061686	0.742634
CMIP	4.415476	4.688068	1.061736	0.483222
ATPAF2	4.464946	4.740823	1.061787	0.549037
ATP5PB	133.8258	142.1141	1.061933	0.517259
VPS53	2.643246	2.807097	1.061988	0.388186
TMEM191	0.279916	0.297329	1.06221	0.816819
LOC10798	0.555574	0.590147	1.062229	0.825123
ZFAT	0.358544	0.38102	1.062686	0.663393
CERS5	11.92158	12.6725	1.062988	0.375532
ZNF235	0.640781	0.681372	1.063345	0.790694
POR	15.12928	16.09489	1.063824	0.512643
NDRG3	26.47132	28.16521	1.06399	0.418155
SNX2	14.22461	15.13484	1.06399	0.737671
TRMT12	4.445153	4.72963	1.063997	0.826794
RABGGTA	6.402127	6.811862	1.064	0.636924
STARD6	0.139525	0.148464	1.064068	0.899672
PXDC1	8.240727	8.769286	1.06414	0.518301
RNGTT	6.455567	6.870729	1.064311	0.445005
F10	0.181284	0.192947	1.064339	0.892752
RAB10	34.07419	36.27055	1.064458	0.696298
MAT2A	44.46777	47.34004	1.064592	0.65559
CKLF	0.870304	0.926598	1.064682	0.544967
TRMT61B	5.857419	6.238112	1.064993	0.746062
WDYHV1	2.763156	2.943031	1.065098	0.766075
APMAP	44.41859	47.31486	1.065204	0.277563
NEDD8	82.61735	88.01791	1.065368	0.343171
PCCA	2.783297	2.96617	1.065704	0.717791
SMYD5	9.001977	9.59444	1.065815	0.215864
GOLGA1	5.79123	6.173136	1.065945	0.632995
ARF3	38.47274	41.01487	1.066076	0.637286
DVL3	10.38861	11.07627	1.066194	0.571967
ZDHHC6	9.606734	10.24423	1.066359	0.572218
WDR7	1.675787	1.787272	1.066527	0.613184
TMEM108	0.93408	0.996224	1.066529	0.765745

DYNLL2	35.29631	37.66125	1.067002	0.504261
TINF2	11.84501	12.64186	1.067273	0.520292
WASL	8.912616	9.512807	1.067342	0.531786
PRRG4	1.160894	1.239252	1.067498	0.791467
NPLOC4	29.9901	32.02111	1.067723	0.675012
PRKACA	37.29349	39.83138	1.068052	0.551746
VPS41	7.043429	7.523974	1.068226	0.635508
ICE1	6.974206	7.450364	1.068274	0.265806
CSDE1	219.4422	234.4559	1.068418	0.226391
SNIP1	4.948207	5.287397	1.068548	0.537208
SPAG16	1.707015	1.824041	1.068556	0.698567
MAD1L1	5.666766	6.058867	1.069193	0.51776
NHP2	187.6785	200.674	1.069243	0.704084
FTO	3.755612	4.015895	1.069305	0.542831
RCN2	70.723	75.63429	1.069444	0.484773
LAGE3	41.8251	44.74616	1.06984	0.520021
NCKAP1	29.81733	31.9004	1.069861	0.419199
KARS	114.7641	122.8162	1.070161	0.216205
AKAP7	1.291853	1.382591	1.070239	0.686223
ISY1	5.100897	5.459252	1.070253	0.377478
HIGD1A	49.70433	53.20187	1.070367	0.490991
NTMT1	18.53127	19.83531	1.07037	0.413936
STX2	8.067216	8.636495	1.070567	0.589413
RNF126	41.93693	44.91494	1.071012	0.588442
VEGFD	0.036752	0.039376	1.071377	0.92893
GHITM	75.93157	81.36095	1.071504	0.421806
RANGAP1	41.02795	43.97143	1.071743	0.511345
ATG2A	4.593217	4.922835	1.071762	0.50568
ADAL	4.553725	4.881697	1.072023	0.575027
FDXACB1	0.984514	1.055457	1.072058	0.842609
RBM43	1.433821	1.537224	1.072117	0.738683
MRPL52	38.62305	41.41323	1.072241	0.365047
PDPN	10.08059	10.80938	1.072297	0.516897
SHMT1	14.69941	15.76226	1.072305	0.720166
DLG5	4.943692	5.303747	1.072831	0.375549
CDIPT	49.83682	53.50615	1.073627	0.46592
ENOX1	0.639162	0.68632	1.073781	0.804534
DDX41	32.72279	35.14084	1.073895	0.352136
B3GLCT	3.113355	3.343452	1.073906	0.668437
DRG2	17.0514	18.31834	1.074301	0.634501
ZBTB9	3.067156	3.295343	1.074397	0.765929
GSK3A	55.64929	59.79341	1.074468	0.455355
CCPG1	11.02907	11.85078	1.074504	0.745803
COA4	11.87252	12.76235	1.074948	0.421512
PLEKHG2	3.302884	3.550508	1.074972	0.07611
TRA2B	34.37994	36.95843	1.075	0.352894
SLC4A7	4.841752	5.205629	1.075154	0.612228
PIGG	5.61344	6.036887	1.075435	0.477163
TMEM251	11.60024	12.47865	1.075724	0.414932
VPS33B	7.495932	8.063805	1.075757	0.329087
SENP5	7.529837	8.101523	1.075923	0.651902
MRPL1	17.67663	19.0208	1.076042	0.655517
NRN1	24.40729	26.26374	1.076061	0.798527
PRKCD	12.24574	13.18235	1.076484	0.196443
SCAMP3	55.17542	59.39699	1.076512	0.636513
UFSP2	15.7714	16.97995	1.076629	0.4894
CRIPAK	1.785675	1.922696	1.076733	0.564867
HSH2D	0.346889	0.373631	1.07709	0.826349

JOSD1	12.25778	13.20316	1.077125	0.651782
HSPB7	0.02749	0.029624	1.077621	0.941042
COASY	24.51338	26.41931	1.077751	0.417259
UBAC1	30.54408	32.92208	1.077855	0.194612
GTF2F2	19.09481	20.58231	1.0779	0.564605
TRAFD1	13.55327	14.6101	1.077976	0.315169
NCOA3	5.080971	5.477447	1.078031	0.675279
TANC2	1.811903	1.953909	1.078374	0.479675
NDUFC2	3.920775	4.228403	1.078461	0.575036
ZDHHC13	10.2121	11.02207	1.079314	0.505819
TAB2	17.65115	19.06473	1.080084	0.539492
CRYBG3	2.400783	2.593598	1.080314	0.581432
DCK	10.03863	10.84599	1.080426	0.415312
MOB2	15.065	16.27824	1.080534	0.38264
UNC119	16.03327	17.33057	1.080913	0.389068
POLR2J	36.98742	39.98544	1.081055	0.326097
C16orf58	35.26403	38.12562	1.081148	0.217587
DLGAP1	0.015865	0.017153	1.081217	0.935073
ODF3B	0.48143	0.520536	1.081227	0.879203
TMUB2	12.90997	13.96119	1.081427	0.422022
ANKRD28	6.550558	7.089909	1.082337	0.568198
RTCB	71.50949	77.41735	1.082616	0.625522
FCHSD1	5.163474	5.593707	1.083323	0.630372
QRSL1	7.602172	8.240508	1.083968	0.700718
TJP1	6.226402	6.749685	1.084043	0.590175
ZNF232	2.30765	2.502088	1.084258	0.523652
NUDT19	5.254702	5.698018	1.084366	0.605841
KCTD21	0.90602	0.982608	1.084533	0.510028
CCDC91	6.001351	6.509444	1.084663	0.620372
SHARPIN	20.4878	22.23359	1.085211	0.299697
PDE4D	0.948275	1.029237	1.085378	0.641788
SLC27A3	1.226043	1.330854	1.085487	0.732166
KLC2	19.32526	20.98099	1.085677	0.568825
FASTKD2	5.291596	5.745604	1.085798	0.634416
UBIAD1	2.720579	2.95431	1.085912	0.677502
PRELID1	182.1884	197.8561	1.085997	0.416145
NAGA	16.01544	17.39292	1.086009	0.501442
PIM2	7.88905	8.568606	1.086139	0.613204
ATG9A	16.95797	18.42257	1.086367	0.291698
RIOX2	6.965137	7.566964	1.086406	0.769858
ENTPD7	2.027943	2.203267	1.086454	0.709156
CRK	19.19909	20.86146	1.086586	0.267161
RPL36AL	246.4139	267.8732	1.087087	0.549974
SEC22A	6.42338	6.984555	1.087365	0.243121
NECAB3	9.488998	10.31852	1.087419	0.656999
IRF3	32.26865	35.09514	1.087593	0.267943
RIPK1	3.461856	3.765103	1.087597	0.647447
TM9SF3	29.86895	32.48932	1.087729	0.298885
UNC50	24.88746	27.07515	1.087903	0.611175
ASB6	7.700988	8.37802	1.087915	0.668528
ZBTB2	3.911534	4.25618	1.08811	0.742568
USP27X	2.083953	2.267842	1.08824	0.73759
FBXO44	7.054144	7.684323	1.089335	0.275495
PSMD11	32.8587	35.79931	1.089493	0.488046
EXD1	0.01964	0.021399	1.089527	0.878523
MIIP	8.831046	9.622191	1.089587	0.211564
LONP1	66.3757	72.33477	1.089778	0.375699
ERLIN2	9.445298	10.29366	1.089818	0.646981

TMEM30A	24.89706	27.13429	1.089859	0.362182
DENND1A	3.042184	3.316804	1.090271	0.551392
LIN7C	6.689272	7.293802	1.090373	0.482006
MAN1A2	3.860972	4.211232	1.090718	0.249957
MTG2	5.008989	5.464125	1.090864	0.585571
LRP2BP	0.077571	0.08462	1.090877	0.66354
CSNK1A1	49.31076	53.82127	1.091471	0.159206
DNAJC17	4.679486	5.107661	1.091501	0.565078
COX5B	280.7964	306.5084	1.091568	0.353216
FEM1A	9.093895	9.928825	1.091812	0.686392
BAG1	5.694205	6.217006	1.091813	0.530168
CFAP61	0.083601	0.091282	1.091887	0.846386
YWHAH	99.83383	109.0073	1.091888	0.216961
PAPOLA	21.87316	23.89015	1.092213	0.044382
STIM1	13.56489	14.8167	1.092283	0.215624
TRIM63	0.035584	0.038872	1.092397	0.917833
CTU2	10.54956	11.52485	1.092449	0.436193
ARMC8	4.640643	5.071299	1.092801	0.48678
NDUFS3	86.09017	94.08147	1.092825	0.551173
TSPAN3	62.04459	67.80473	1.092839	0.42681
TMEM98	20.54125	22.45349	1.093093	0.480904
QSOX2	16.29011	17.81322	1.093499	0.013606
DNAJA2	28.65377	31.33718	1.093649	0.41515
KDM4B	5.442943	5.954935	1.094065	0.437095
TMOD3	9.11074	9.969403	1.094247	0.293649
ZNF41	1.086107	1.188622	1.094388	0.728227
KIF7	12.28789	13.44771	1.094388	0.580019
NAA20	35.95633	39.35059	1.094399	0.626367
IKZF4	0.807101	0.88335	1.094473	0.699894
FAM114A1	4.18777	4.583786	1.094565	0.492573
TNFSF13	0.058012	0.063505	1.094683	0.943462
RAB43	0.218246	0.238927	1.094758	0.201265
HIP1	9.404068	10.2952	1.09476	0.40699
GLRX3	32.92812	36.05716	1.095026	0.317411
NIPSNAP3	15.1752	16.63329	1.096084	0.507615
TMEM186	13.59399	14.90658	1.096556	0.4882
KTI12	11.19779	12.28363	1.096969	0.429882
NSA2	115.3598	126.5473	1.096979	0.067811
CENPA	14.16388	15.54122	1.097243	0.633383
RAB1A	69.72756	76.51489	1.097341	0.135456
RNF167	43.77997	48.05586	1.097668	0.247417
HNRNPA2	301.0485	330.4541	1.097678	0.334457
ARHGEF18	4.510734	4.952685	1.097978	0.43855
MRPL11	38.02782	41.76677	1.098321	0.303584
MORN1	0.724634	0.795907	1.098358	0.661163
GIT2	6.575009	7.221831	1.098376	0.222626
MCUB	16.47205	18.09496	1.098525	0.507256
ATF6B	17.78275	19.53481	1.098526	0.38317
GTSE1	10.88654	11.96003	1.098608	0.3326
PDIA6	147.8307	162.4563	1.098935	0.021467
PHKB	8.321901	9.14865	1.099346	0.512628
FOPNL	21.04718	23.14207	1.099533	0.24494
PTPN12	17.02224	18.72847	1.100235	0.077914
KDELC2	9.902084	10.89728	1.100504	0.690556
JPT1	33.56403	36.94111	1.100616	0.578689
NPIPA5	1.483099	1.632649	1.100836	0.620229
TMEM184	7.207923	7.940603	1.101649	0.179903
TMEM141	40.49238	44.61493	1.10181	0.718927

TRIM35	4.050297	4.463029	1.101902	0.453717
REEP3	1.867646	2.057989	1.101916	0.297165
AFF3	0.908378	1.000966	1.101926	0.578378
IFI16	14.34323	15.80644	1.102014	0.522738
VSNL1	0.269078	0.296542	1.102067	0.884854
GALNT6	3.194153	3.521046	1.102341	0.528398
UCK1	17.88069	19.71189	1.102413	0.581348
ISCA2	31.09039	34.28714	1.102821	0.427623
INSIG2	10.99394	12.12663	1.103028	0.591042
ECD	16.95245	18.70041	1.10311	0.566268
TNRC18	4.438158	4.89646	1.103264	0.433024
RNF13	10.59908	11.69451	1.103351	0.527356
RPS21	249.0398	274.8602	1.10368	0.311081
PRAF2	63.5251	70.13467	1.104047	0.36206
RAPGEF1	4.502314	4.973494	1.104653	0.162123
CENPW	38.12532	42.12227	1.104837	0.386264
ZFAND2B	17.46167	19.29879	1.105208	0.414257
CHURC1	6.323483	6.989027	1.10525	0.583791
GEMIN7	9.940392	10.98804	1.105393	0.460259
PEX2	8.115859	8.974401	1.105786	0.275366
ERP29	147.8593	163.6327	1.106678	0.119487
PRKCSH	183.5887	203.2016	1.106831	0.287147
IGSF8	11.32889	12.54046	1.106946	0.643157
OGFOD2	6.750495	7.474992	1.107325	0.113215
DLGAP5	31.59217	34.99098	1.107584	0.274886
RBM14	9.851271	10.91215	1.10769	0.671784
SEH1L	7.438659	8.243206	1.108158	0.413765
7-Sep	26.09143	28.9178	1.108326	0.197716
ZNF446	3.447895	3.821644	1.108399	0.620422
MKNK1	1.710578	1.896079	1.108443	0.163943
CMC1	3.992063	4.425014	1.108453	0.392732
CSNK1G2	20.95607	23.23341	1.108672	0.188835
SCD5	7.203705	7.987583	1.108816	0.563951
ZNF827	0.420259	0.46609	1.109054	0.599818
ANLN	26.38197	29.25916	1.109059	0.43214
LAMP2	15.30531	16.97663	1.109198	0.136626
ZNF217	5.525887	6.129616	1.109255	0.48634
TMEM88	0.189428	0.210132	1.109301	0.668885
ABCA9	0.008092	0.008977	1.109307	0.933702
BICD2	9.465125	10.50249	1.109599	0.510054
MRPL49	36.2079	40.18483	1.109836	0.383979
MTHFR	1.181932	1.311866	1.109934	0.643289
LRRC45	8.339663	9.259862	1.11034	0.616975
2-Sep	79.72579	88.53473	1.110491	0.222355
SLC25A45	0.640532	0.711445	1.11071	0.824839
KIF13A	3.844013	4.269821	1.110772	0.217612
PPARA	2.20538	2.449856	1.110855	0.406899
CTSC	6.803977	7.55852	1.110897	0.515213
MNAT1	12.11756	13.4662	1.111296	0.448645
NPEPL1	6.055408	6.73402	1.112067	0.790693
ATP13A1	15.41503	17.14545	1.112256	0.537281
SEM1	34.66572	38.55852	1.112295	0.33117
ICMT	27.59893	30.69887	1.112321	0.000591
MRPS33	83.13999	92.51565	1.11277	0.412527
KIAA1841	1.951149	2.171263	1.112812	0.388776
TGM3	0.027717	0.030847	1.112919	0.880215
H2AFZ	563.4014	627.1213	1.113098	0.130511
EXOSC10	34.00569	37.87165	1.113686	0.295467

KAT2B	4.996731	5.564935	1.113715	0.446533
MGAT2	10.31593	11.48919	1.113733	0.651736
ITGAV	10.17315	11.33056	1.113771	0.573275
OAF	17.765	19.78628	1.113779	0.181496
C15orf41	2.426705	2.70321	1.113942	0.586235
LAMC1	52.88605	58.91636	1.114025	0.214989
HGH1	6.964295	7.76193	1.114532	0.685311
NDUFA4	84.5928	94.28217	1.114541	0.263678
CCS	21.56318	24.03541	1.114651	0.378399
LRP12	3.874969	4.319403	1.114694	0.408884
RNASEH1	12.39594	13.81795	1.114716	0.184646
HAUS8	14.13985	15.76574	1.114987	0.162355
FAM103A1	15.63354	17.43225	1.115055	0.119302
CMTR2	3.790312	4.226712	1.115136	0.624276
SUSD6	2.579948	2.878181	1.115596	0.344069
NOL8	6.758903	7.540304	1.115611	0.425866
MTMR12	4.905736	5.473812	1.115798	0.159334
RECK	2.899603	3.235494	1.11584	0.142748
NCK2	7.934389	8.854752	1.115997	0.098471
THUMPD3	14.40961	16.08535	1.116294	0.44201
GTF2E1	5.073432	5.664131	1.11643	0.559131
FUCA1	5.840525	6.523088	1.116867	0.548886
PPP4C	121.6208	135.8407	1.11692	0.106985
C2orf27A	1.813788	2.026643	1.117354	0.457373
ABI1	8.721675	9.74738	1.117604	0.369036
ANAPC4	7.354425	8.220342	1.117741	0.337599
TM9SF4	37.21468	41.5973	1.117766	0.124963
CLN5	16.40969	18.34518	1.117948	0.328043
HLX	2.829329	3.16358	1.118138	0.182427
CHCHD3	55.42231	61.98251	1.118367	0.221453
RP2	3.245031	3.629709	1.118544	0.07102
RBMS1	12.22	13.67147	1.118778	0.344046
SYNPO2	0.091008	0.10183	1.118913	0.669774
NDUFAB1	111.9892	125.3319	1.119143	0.391066
UQCRC1	182.7986	204.5986	1.119257	0.031107
ADIRF	0.110095	0.123226	1.119274	0.905835
CDC42EP1	38.90395	43.56086	1.119703	0.53163
CYB5R4	9.237804	10.3448	1.119833	0.491405
KATNBL1	5.76004	6.450455	1.119863	0.138595
PIK3R1	1.74325	1.95233	1.119937	0.409299
RTN4RL2	9.510364	10.65204	1.120046	0.59062
COPS5	44.72712	50.11041	1.120358	0.36884
TBK1	9.25537	10.36991	1.12042	0.200095
POLM	3.617931	4.054733	1.120732	0.371826
RFX7	1.399814	1.568848	1.120755	0.281753
CFAP58	0.240724	0.269895	1.121178	0.738033
PGAP3	3.571798	4.004672	1.121192	0.490194
ARFGEF2	4.227029	4.739429	1.12122	0.53479
GRSF1	12.6804	14.2177	1.121235	0.285724
FCER1G	0.538997	0.604387	1.121318	0.885711
SLC1A3	4.534398	5.087684	1.12202	0.62895
LOC10798	0.615156	0.690395	1.122308	0.789146
HSD17B4	46.51525	52.20631	1.122348	0.160338
LOC10537	0.392294	0.440446	1.122745	0.778382
ACSF3	1.139775	1.279813	1.122864	0.512506
CCDC127	15.79426	17.73824	1.123081	0.030416
ATN1	21.69763	24.36924	1.123129	0.380747
LOC10536	0.135217	0.151904	1.123411	0.867997

TIMM50	17.6539	19.84068	1.123869	0.287597
DHRS7B	6.489827	7.297717	1.124486	0.365658
FEM1C	4.143036	4.658893	1.124512	0.199152
TAF6	21.22178	23.86819	1.124703	0.012901
ARFIP2	29.65269	33.35852	1.124974	0.154561
VMO1	0.346921	0.39029	1.125012	0.824933
SOAT1	7.253274	8.161346	1.125195	0.260333
ACTA2	11.58198	13.03318	1.125298	0.744941
SMG9	7.209312	8.113332	1.125396	0.484657
PEMT	15.7363	17.7102	1.125436	0.132886
SLC35E2A	1.872418	2.107914	1.125771	0.204081
IRF2BP1	14.81298	16.67883	1.12596	0.479604
GNB4	6.567064	7.395083	1.126087	0.204224
NREP	10.22104	11.51389	1.12649	0.737896
ASAP1	6.769447	7.626529	1.12661	0.344364
POLE3	46.10673	51.9456	1.126638	0.228162
CMTM6	28.67552	32.30775	1.126667	0.10973
IRAK4	2.572021	2.898402	1.126897	0.618368
ATP2A3	1.19029	1.341505	1.127041	0.481827
IDH2	59.84067	67.46505	1.127411	0.56703
SPATA5	0.569476	0.64208	1.127492	0.234402
SEC13	96.53684	108.8805	1.127865	0.505774
MICAL3	3.938756	4.442403	1.12787	0.439112
KIAA0754	0.643425	0.726179	1.128616	0.798045
ZNRF2	2.222138	2.508208	1.128737	0.42785
TUSC2	22.96967	25.93336	1.129026	0.21223
SZRD1	57.13719	64.51108	1.129056	0.383056
CLIP1	12.0946	13.6576	1.129231	0.260018
POMT2	5.862494	6.621037	1.129389	0.311221
SAMD10	1.618305	1.827824	1.129468	0.736852
STON2	1.185672	1.339181	1.12947	0.64704
SIRT6	15.51744	17.53931	1.130297	0.33765
INO80E	21.38942	24.17797	1.13037	0.142911
ZNF564	2.16643	2.44958	1.130699	0.635596
ANKIB1	3.967614	4.486704	1.130832	0.344958
NBEA	1.131929	1.280212	1.131	0.671952
HMGXB3	15.6793	17.73361	1.131021	0.319866
PUF60	19.04278	21.53851	1.131059	0.13382
NSUN2	21.77421	24.62849	1.131085	0.208599
MRPS2	33.61865	38.02904	1.131189	0.439773
CD109	7.141404	8.079153	1.131312	0.59756
PDE2A	0.28679	0.324484	1.131432	0.770073
PDZK1	0.07124	0.080604	1.131432	0.740924
DHCR7	19.70475	22.30228	1.131823	0.619361
GPI	34.84556	39.44027	1.131859	0.370237
PSMA6	52.33107	59.25641	1.132337	0.243092
SPCS1	167.0917	189.2342	1.132517	0.066217
LAMTOR3	6.461543	7.318421	1.132612	0.01651
OVCH1	0.004614	0.005226	1.132655	0.920942
ANKAR	0.293286	0.332271	1.132923	0.495732
PIGW	2.971887	3.367261	1.133038	0.447039
RTL8A	59.04368	66.91876	1.133377	0.440968
RAP1B	34.11931	38.67852	1.133626	0.145175
PDE8A	7.780156	8.82097	1.133778	0.288322
CFP	0.08414	0.095426	1.134126	0.886129
PSEN2	8.177321	9.276875	1.134464	0.540219
TSFM	13.15916	14.92954	1.134535	0.445126
TRIM38	2.198346	2.49422	1.134589	0.117644

RCC1L	10.71587	12.16035	1.134798	0.403694
TMEM14B	33.15497	37.63748	1.135199	0.605286
NMB	11.33932	12.88489	1.136301	0.168863
FKBP7	3.682553	4.184777	1.136379	0.127066
GALNT2	24.60345	27.96094	1.136464	0.158095
HIRIP3	20.82357	23.66601	1.136501	0.561082
ERLIN1	24.04615	27.33183	1.136641	0.190289
PARPBP	5.489025	6.240188	1.136848	0.338243
ARF5	127.8211	145.3248	1.136939	0.068621
SLC12A2	1.662874	1.890795	1.137065	0.369328
TIMM13	29.07549	33.06227	1.137118	0.490297
PDE6D	19.546	22.22654	1.13714	0.452161
HMOX2	9.801943	11.15079	1.13761	0.487642
AZIN2	1.887689	2.148396	1.138109	0.34314
CDH24	3.170872	3.61035	1.138598	0.257494
ILKAP	8.534374	9.717532	1.138634	0.205147
TNFRSF25	2.328034	2.650866	1.138671	0.654345
SMARCA2	8.48527	9.662759	1.138769	0.237131
PEX11A	4.890513	5.569705	1.138879	0.506906
ATF1	3.017357	3.436673	1.138968	0.377531
MMAA	0.614548	0.700003	1.139052	0.616094
PSEN1	10.69359	12.18198	1.139185	0.010872
FBXL5	13.87377	15.80558	1.139242	0.287702
EEF2KMT	4.247197	4.838717	1.139273	0.617585
SNAP29	14.43239	16.44259	1.139284	0.204208
C1orf50	9.634345	10.97698	1.139359	0.29715
DEXI	13.01568	14.83365	1.139675	0.354695
PRDX5	300.0355	341.9662	1.139753	0.128928
CSNK1G1	2.594583	2.957297	1.139797	0.245285
ANKRD37	5.490021	6.257695	1.139831	0.58047
RARS2	11.99702	13.67672	1.14001	0.072167
ZFAND6	17.86856	20.37516	1.14028	0.093287
BET1	28.58017	32.59841	1.140595	0.198301
KIF5B	20.05932	22.88125	1.140679	0.184493
UGGT1	8.810061	10.05374	1.141166	0.264712
TFG	72.56581	82.81867	1.14129	0.079287
USP40	4.851809	5.537477	1.141322	0.545751
PCNX3	7.244192	8.269282	1.141505	0.426958
ATP6V1F	216.6318	247.3386	1.141747	0.248623
DPH3	16.62719	18.99366	1.142325	0.014026
COMMD3	8.803518	10.05673	1.142353	0.113392
IL34	0.156127	0.178426	1.142826	0.787425
ENDOD1	5.022569	5.740532	1.142947	0.640332
FAM160A:	7.419881	8.480724	1.142973	0.141016
SPATA6	1.288471	1.472919	1.143152	0.197895
THAP1	4.723713	5.400412	1.143256	0.372916
CRIP2	11.77722	13.46621	1.143412	0.322487
CTRL	0.521797	0.596772	1.143687	0.593668
RSRP1	7.950734	9.093177	1.14369	0.601485
GTF3A	81.11135	92.7672	1.143702	0.375128
SUMO3	43.36952	49.62089	1.144142	0.038586
SSSCA1	26.96954	30.8627	1.144354	0.225516
NANS	41.38735	47.36641	1.144466	0.085444
ICAM1	8.401331	9.615844	1.144562	0.468634
MEIS2	7.711008	8.82685	1.144708	0.24523
EMG1	36.85136	42.19435	1.144988	0.518707
EBAG9	5.938163	6.799257	1.14501	0.449726
GDPGP1	1.178726	1.34984	1.145169	0.418837

CLCN2	1.322915	1.514963	1.14517	0.328471
ADD1	30.77488	35.24562	1.145272	0.177585
TECPR1	4.023568	4.6086	1.145401	0.250232
YAE1D1	3.739219	4.283553	1.145574	0.487605
TTYH3	20.96345	24.02374	1.145982	0.249307
MAPKAPK	15.77881	18.08233	1.145989	0.15833
RYK	19.87402	22.77554	1.145996	0.074925
POLR2K	68.94057	79.00763	1.146025	0.077902
ZDHHC3	6.372669	7.30335	1.146043	0.012916
CISD1	14.8855	17.0604	1.146109	0.321974
FGFRL1	7.813078	8.956333	1.146326	0.07669
R3HDM4	24.38492	27.95739	1.146503	0.335196
ZBTB7B	5.652493	6.481831	1.146721	0.10807
CLGN	13.48705	15.46888	1.146943	0.553349
PRMT1	127.512	146.3109	1.147429	0.222327
CHMP2B	21.77058	24.996	1.148155	0.357243
SLC35G2	3.25631	3.739182	1.148288	0.613976
GLT8D1	31.43129	36.09411	1.14835	0.12016
HIST1H4E	0.79686	0.915126	1.148415	0.638893
USP12	11.88633	13.65217	1.148561	0.222237
RARS	78.79188	90.52186	1.148873	0.062338
NAP1L4	79.26876	91.07386	1.148925	0.042798
C5orf47	0.017634	0.020263	1.149059	0.852167
FKBP1A	162.0224	186.1794	1.149097	0.037603
CDAN1	2.817792	3.240474	1.150005	0.066773
UBL4A	22.46696	25.84602	1.150401	0.154465
RNF6	4.253333	4.893371	1.150479	0.464519
VSIG2	0.039305	0.04522	1.150491	0.88281
MEIS3	11.74958	13.519	1.150595	0.254266
IZUMO4	0.770143	0.886171	1.150658	0.277525
BAG5	10.00361	11.51685	1.15127	0.04138
PRKAR1A	24.21383	27.87697	1.151283	0.140218
PCSK5	1.001684	1.153299	1.15136	0.355343
TMA7	335.6178	386.4348	1.151413	0.077087
PBLD	1.17083	1.34817	1.151465	0.202288
BCL11B	0.512937	0.590671	1.151547	0.520393
RFK	11.9499	13.76378	1.15179	0.102484
ZNF784	2.828096	3.259457	1.152527	0.431859
FAM81A	0.537969	0.620043	1.152565	0.722622
WDR70	14.89099	17.16769	1.152891	0.189986
PRR12	4.752276	5.479467	1.15302	0.112826
RELN	0.004883	0.005631	1.153129	0.803834
METTL13	14.05781	16.2161	1.153529	0.164935
HSPB8	17.11102	19.74089	1.153695	0.560083
LMO7	2.208424	2.54816	1.153836	0.308406
ERI1	3.477919	4.013108	1.153882	0.101161
SNTA1	16.43431	18.96613	1.154057	0.337629
CBARP	2.826533	3.26346	1.15458	0.386614
CNN1	0.717158	0.828019	1.154583	0.74224
RBMXL2	0.050413	0.05821	1.15466	0.891108
8-Mar	3.500461	4.042197	1.154761	0.010657
CPT1A	12.20015	14.08892	1.154815	0.13497
MUS81	11.49274	13.27328	1.154928	0.147369
ARF6	21.23924	24.53288	1.155073	0.360989
MAB21L1	0.750233	0.866808	1.155386	0.683911
PRG4	0.010994	0.012703	1.155463	0.910073
LOC10537	0.878488	1.015498	1.155962	0.665025
POLR2I	75.13681	86.89514	1.156492	0.116549

SLC24A3	0.014584	0.016869	1.156733	0.856201
ARHGAP2	6.030604	6.976612	1.156868	0.137735
YWHAG	54.93649	63.55855	1.156946	0.09819
AGFG1	7.674463	8.882358	1.157391	0.01665
LIPA	21.03005	24.34028	1.157405	0.211324
SAP30BP	19.84596	22.9713	1.15748	0.038923
AP3D1	40.48416	46.86822	1.157693	0.005617
PPT1	60.94286	70.59137	1.158321	0.154421
SMARCAL	10.75779	12.46162	1.158381	0.33268
CCNE1	9.621562	11.15516	1.159392	0.51701
COL28A1	0.021342	0.024755	1.15988	0.32606
NDUFA6	76.87749	89.16902	1.159885	0.050301
MMP8	0.017161	0.019907	1.159967	0.907628
FEN1	81.44485	94.49849	1.160276	0.132985
PAFAH1B3	22.4762	26.08299	1.160471	0.429034
PPP1CB	27.63751	32.07387	1.16052	0.220377
DDX56	21.3344	24.76002	1.160568	0.21351
CNPY3	14.83739	17.2272	1.161067	0.128104
EIPR1	9.957275	11.5614	1.161101	0.24845
STARD5	4.614963	5.358447	1.161103	0.132127
IFIH1	0.881056	1.023145	1.161272	0.207442
TPRKB	36.2536	42.11095	1.161566	0.43584
ALDH1B1	9.343429	10.85682	1.161974	0.644782
FZR1	11.84428	13.77218	1.162771	0.102567
EFTUD2	32.65961	37.97623	1.162789	0.214607
PNPO	9.138465	10.62786	1.162981	0.486018
COPB2	60.08018	69.87926	1.1631	0.271238
CCDC130	10.7856	12.54575	1.163195	0.270258
ERBIN	6.747566	7.854634	1.164069	0.246493
RMDN3	10.14726	11.81711	1.164561	0.185155
CUEDC2	55.85758	65.09605	1.165393	0.043452
SMPDL3A	2.132518	2.485397	1.165475	0.270828
PTRH2	12.77982	14.90445	1.166248	0.00733
GRB7	0.379055	0.442106	1.166336	0.529626
KLHL42	3.073455	3.585307	1.16654	0.422276
RPS26	198.1716	231.2051	1.166691	0.003482
FBXW9	5.600047	6.533874	1.166753	0.438755
ZNF530	1.81061	2.112899	1.166954	0.566462
TMEM8A	12.6342	14.74573	1.167128	0.088144
RXYLT1	5.603381	6.540803	1.167296	0.643763
TM7SF3	9.346469	10.91125	1.167419	0.223409
WDR92	5.492929	6.413204	1.167538	0.471007
USP8	7.208157	8.416092	1.167579	0.261037
PSMB1	249.5528	291.6327	1.168622	0.069302
PUDP	6.030724	7.048472	1.16876	0.559753
PTGES2	22.91249	26.77948	1.168772	0.130778
BRD9	5.506916	6.436384	1.168782	0.007622
KLF16	17.38476	20.32292	1.169008	0.229664
SLC26A1	0.051023	0.059647	1.169028	0.816616
LRRC23	8.040503	9.400873	1.16919	0.142453
TSHR	0.011142	0.013027	1.169233	0.608565
MRPL16	40.53041	47.39537	1.169378	0.188017
PCSK7	4.738184	5.540865	1.169407	0.082508
RAD21L1	0.087438	0.102351	1.170549	0.686628
TRERF1	0.557312	0.652448	1.170705	0.428645
CHMP3	0.11871	0.138985	1.170796	0.510955
NUP188	17.04197	19.95479	1.17092	0.106785
MMP17	4.721692	5.528852	1.170947	0.276891

ANAPC11	54.30255	63.58876	1.171009	0.133448
TMEM179	56.41385	66.06786	1.171128	0.133584
RPP25L	14.73047	17.2549	1.171375	0.453072
ARFIP1	4.814711	5.643379	1.172112	0.419304
LOC10536	0.277289	0.325032	1.172179	0.80795
AUP1	67.63588	79.29494	1.17238	0.174522
UBE2V2	28.32686	33.22256	1.172829	0.244015
MAP3K13	0.87803	1.0299	1.172966	0.144581
NELFCD	35.86103	42.06944	1.173124	0.027102
UBE2A	58.04658	68.12708	1.173662	0.233842
10-Sep	10.42486	12.23641	1.173772	0.057566
MRPL4	22.11987	25.96649	1.173899	0.228088
RABL6	38.8228	45.58843	1.17427	0.006415
PTPN1	26.90212	31.59424	1.174415	0.29142
PEX3	7.893132	9.271274	1.1746	0.376955
CCDC124	127.6966	150.0316	1.174907	0.066645
ZNF616	1.788919	2.102496	1.175289	0.506677
ALKBH5	43.00806	50.5477	1.175307	0.178761
CNIH1	62.68401	73.68046	1.175427	0.248239
COPS2	27.7831	32.67232	1.175978	0.257197
MFN1	10.5559	12.41789	1.176393	0.035771
GPR150	0.026818	0.031551	1.176456	0.840676
KIF18A	10.87956	12.79957	1.176479	0.135198
MAP3K12	3.241781	3.81578	1.177063	0.278651
SLC20A2	2.088596	2.458661	1.177183	0.170649
SCP2	17.7421	20.89395	1.177648	0.109531
VNN2	0.017139	0.020186	1.177802	0.86212
YWHAZ	143.9544	169.5508	1.17781	0.00539
PSMG1	51.14981	60.26997	1.178303	0.435933
AFG1L	0.675188	0.795792	1.178623	0.488562
BATF2	0.57519	0.678008	1.178755	0.632888
SAMM50	39.69796	46.80242	1.178963	0.418663
MYLPF	0.119427	0.140824	1.179164	0.769725
TRMT2B	3.789794	4.471561	1.179896	0.556021
IL13RA2	1.856379	2.190341	1.1799	0.654104
DMAP1	14.13031	16.67265	1.179921	0.113823
CDC37L1	5.587441	6.594914	1.18031	0.120517
LCMT1	31.18633	36.81128	1.180366	0.196072
ATP6V0C	86.51591	102.1416	1.18061	0.193635
KIAA0556	2.986707	3.527386	1.181029	0.061401
PIK3C2G	0.004113	0.004857	1.181059	0.883669
SLC25A12	7.14597	8.440083	1.181097	0.324767
LRRC41	23.19731	27.39866	1.181114	0.187029
WDPCP	0.276796	0.326996	1.181364	0.322866
PNMA2	6.127372	7.239206	1.181454	0.394142
THADA	4.730065	5.588397	1.181463	0.29559
RNF227	0.921596	1.089309	1.181981	0.403104
PRPF40B	1.871604	2.21246	1.18212	0.37629
POLR2G	87.44653	103.4073	1.18252	0.111793
CATSPER3	0.344449	0.407443	1.182883	0.536755
CENPM	11.94402	14.13006	1.183024	0.364869
DNAJC25	9.724231	11.50404	1.183029	0.4094
E2F1	44.2489	52.35411	1.183173	0.07905
RSPRY1	7.926163	9.378579	1.183243	0.025217
NAB1	7.987425	9.451228	1.183263	0.123814
LOC73009	5.534949	6.549951	1.183381	0.118681
LOC10028	1.173481	1.388907	1.183579	0.563937
APIP	28.18855	33.36613	1.183677	0.075981

ZC3H14	6.045641	7.156934	1.183817	0.089124
EIF2S2	121.0274	143.3089	1.184103	0.230065
TMTC4	3.610786	4.276139	1.184268	0.470837
CHST3	2.640464	3.127044	1.184278	0.508792
WDR13	13.83472	16.3862	1.184426	0.05017
RNASEH2(	24.69678	29.25897	1.184728	0.10141
ZNF267	4.44554	5.266936	1.184769	0.484403
LOC10272	0.015218	0.01803	1.184812	0.83888
MR1	2.019379	2.392776	1.184907	0.534904
NFE2L2	20.29978	24.05701	1.185087	0.233054
MTX1	14.74996	17.48164	1.185199	0.220643
PHKG2	6.972981	8.267606	1.185663	0.193136
KLF7	1.996421	2.367398	1.185821	0.423757
IL37	0.064522	0.076513	1.185846	0.603958
DNALI1	0.402436	0.47723	1.185855	0.456663
CFAP73	0.199554	0.236729	1.186293	0.578342
UBLCP1	16.81103	19.94483	1.186413	0.112785
CYHR1	8.026068	9.523023	1.186512	0.139922
KCNN3	0.438652	0.520477	1.186537	0.771742
TPP2	9.505045	11.27893	1.186625	0.139939
GPAM	3.879096	4.603644	1.186783	0.686047
PDK2	7.65015	9.080806	1.18701	0.202377
SRSF2	55.5424	65.93281	1.187072	0.10163
SCN3A	0.027822	0.033043	1.187679	0.687919
ABHD14A	4.855731	5.768838	1.188047	0.541703
VPS45	18.42611	21.8912	1.188053	0.161226
ANKRD52	12.49322	14.84864	1.188535	0.379106
SGPP1	6.016991	7.153451	1.188875	0.029176
NUDT16	4.105533	4.882657	1.189287	0.018323
CISD2	7.471565	8.887184	1.189468	0.000203
SLC25A13	9.170807	10.90894	1.189529	0.449994
CYREN	7.121156	8.471459	1.189618	0.287445
ATP6V1H	13.98438	16.63725	1.189702	0.188726
GPSM2	8.169099	9.72012	1.189864	0.269083
CBWD6	1.651895	1.965932	1.190107	0.222244
SH3BGRL	29.15603	34.70148	1.190199	0.186021
RABGEF1	7.770139	9.249355	1.190372	0.129379
SHOC2	8.794276	10.46986	1.190531	0.221669
INTS6	1.787568	2.128572	1.190764	0.045403
ZBTB22	4.856153	5.782551	1.190768	0.390171
TIFA	3.508087	4.178283	1.191043	0.227433
PRDX4	138.9302	165.5135	1.191343	0.181461
ELMSAN1	3.489313	4.15779	1.191578	0.216991
HP1BP3	15.53791	18.51879	1.191846	0.113874
HSD11B1L	5.81083	6.926643	1.192023	0.578572
PMM1	37.49479	44.69931	1.192147	0.049281
FYB1	0.014929	0.017806	1.192696	0.866578
GLRX5	56.79073	67.73777	1.192761	0.087983
INCA1	0.248234	0.296091	1.192789	0.667216
CDC37	161.2895	192.4611	1.193265	0.073484
GNA15	0.107905	0.128798	1.19363	0.771502
PRIMPOL	5.244199	6.259753	1.193653	0.368921
YWHAE	419.2144	500.6842	1.194339	0.052834
DYNC1I2	22.19703	26.51489	1.194524	0.013727
WDR5B	2.306037	2.754823	1.194613	0.579643
ZWILCH	20.40254	24.37399	1.194654	0.039434
JMJD6	9.822707	11.73496	1.194677	0.375256
MAGEL2	0.097711	0.116736	1.1947	0.830777

HSPBAP1	1.873666	2.238702	1.194825	0.505468
GPR37	0.676554	0.808826	1.195508	0.290139
SMIM20	29.43162	35.18683	1.195545	0.190832
CYSTM1	60.35937	72.16631	1.195611	0.151998
CSE1L	94.41174	112.8798	1.195612	0.300531
IMMP1L	9.910563	11.85203	1.195898	0.119012
RIIAD1	0.122663	0.146692	1.1959	0.885522
LIN37	12.62901	15.10379	1.19596	0.367938
RABL2B	3.179563	3.803939	1.196371	0.24359
RFXANK	23.17232	27.72277	1.196374	0.172968
COMMD6	55.04321	65.85334	1.196394	0.149404
HINT1	360.0774	430.8146	1.19645	0.051832
ZNF180	1.454028	1.739685	1.196459	0.243518
GLMP	15.74233	18.84773	1.197265	0.501548
MFHAS1	3.67921	4.40586	1.197501	0.266328
FTSJ3	19.30677	23.1214	1.19758	0.009955
MAD2L2	29.73263	35.60929	1.19765	0.222452
UCKL1	6.278305	7.519227	1.197652	0.015306
MMACHC	3.032907	3.632727	1.197771	0.609467
TMCC1	1.579785	1.892291	1.197815	0.00394
ZBTB24	2.224847	2.665042	1.197854	0.216335
PPAN	0.96732	1.158725	1.197872	0.205489
BBIP1	10.06638	12.05826	1.197874	0.065221
GINM1	39.2757	47.0494	1.197926	0.018399
DOLPP1	11.6639	13.97416	1.198069	0.296785
XPO6	23.2895	27.9243	1.199008	0.04617
PSMD12	20.76839	24.90974	1.199407	0.113626
HDLBP	105.8362	126.9461	1.199459	0.055875
TTF1	7.660397	9.188775	1.199517	0.075978
PTPN6	0.035725	0.042859	1.199664	0.882007
ABHD17A	10.31201	12.37702	1.200253	0.299053
RBBP9	5.135078	6.166608	1.200879	0.222777
PSAP	243.3798	292.3109	1.201048	0.145597
INAFM1	19.3699	23.27515	1.201614	0.459537
TXNL1	36.96165	44.42298	1.201867	0.08765
USP47	6.81825	8.19594	1.202059	0.120117
NDUFS5	361.8302	435.0065	1.202239	0.06767
UFM1	21.1835	25.47094	1.202395	0.198945
NSMCE3	15.26267	18.36427	1.203215	0.024548
CD276	40.37709	48.58532	1.203289	0.264157
APOLD1	1.219681	1.467681	1.203332	0.455211
LRFN4	5.946797	7.156041	1.203344	0.214387
GABPB2	2.29935	2.767439	1.203574	0.361236
NECTIN2	35.08902	42.24257	1.203869	0.134853
HOMER2	4.575859	5.508788	1.203881	0.022439
ADAM23	3.809673	4.586576	1.203929	0.011379
UBXN11	6.985375	8.410316	1.203989	0.471218
GOLPH3	16.35521	19.69922	1.204461	0.043879
CERKL	0.452473	0.545162	1.204851	0.56705
PLCD1	4.004426	4.824925	1.204898	0.217474
LOC10537	0.092923	0.111976	1.20504	0.740286
ZACN	0.101681	0.122561	1.205349	0.469308
TMEM205	42.71266	51.49122	1.205526	0.114494
TMEM219	80.77822	97.40641	1.20585	0.025685
RBPJ	20.86651	25.16608	1.206051	0.045976
CLHC1	1.076674	1.29866	1.206178	0.326836
TRAPPC2E	6.691001	8.081371	1.207797	0.34416
GATD3B	1.638508	1.979348	1.208019	0.28812

LOC10798	0.514108	0.621438	1.20877	0.685546
RPP30	7.629776	9.224375	1.208997	0.142974
HPS5	5.039816	6.093986	1.209168	0.098935
IQCE	2.68608	3.247943	1.209176	0.080752
SLC44A1	5.678814	6.868787	1.209546	0.006324
ILVBL	15.36394	18.58411	1.209593	0.097435
NSRP1	16.20505	19.60204	1.209625	0.405231
PPP3R1	19.35749	23.41647	1.209685	0.099169
OAZ2	56.77529	68.6975	1.209989	0.033268
TAF12	16.95037	20.52071	1.210635	0.000824
NDEL1	11.82492	14.31988	1.210992	0.227509
OSM	0.088204	0.10685	1.211406	0.866698
MCF2L	0.988932	1.198006	1.211414	0.055826
TMEM39B	6.07756	7.362976	1.211502	0.056903
FYTTD1	13.74681	16.6591	1.211852	0.018798
RCBTB2	0.531321	0.644107	1.212275	0.216302
TBCCD1	3.452351	4.1862	1.212565	0.25605
VPS29	56.61633	68.65567	1.212648	0.032252
NOVA1	1.383179	1.677458	1.212755	0.136888
MRPL43	15.91542	19.30221	1.212799	0.072108
UROS	9.544728	11.58074	1.213313	0.309584
CHMP5	53.13512	64.50412	1.213964	0.192356
CROCC	1.495028	1.814959	1.213997	0.023611
UGT8	2.925901	3.552188	1.214049	0.192072
PRPF6	45.52975	55.27746	1.214096	0.329712
ARL16	16.37294	19.88117	1.21427	0.122926
NFYC	8.745285	10.61948	1.214309	0.279033
TMEM63B	11.91781	14.47335	1.214431	0.013515
RALGAPB	8.742812	10.62595	1.215392	0.080408
HDGF	125.3764	152.4901	1.216259	0.030664
DYNC1LI2	20.91565	25.43885	1.216259	0.218092
NUP88	18.24479	22.19827	1.216691	0.243563
KNSTRN	32.70185	39.79476	1.216896	0.203697
PCOTH	1.527001	1.858331	1.216981	0.712363
CMC2	41.12937	50.06098	1.217159	0.170202
ELN	0.106829	0.130035	1.217222	0.641138
SOWAHC	1.914791	2.33114	1.217439	0.423295
DNAJC24	10.07563	12.2674	1.217531	0.097514
ARL8B	29.49741	35.92196	1.217801	0.071947
SPIN2B	2.615797	3.185994	1.217982	0.221081
DDX23	28.06072	34.17809	1.218005	0.097642
HNRNPUL	61.51833	74.93067	1.218022	0.023931
ZBTB12	3.05362	3.71941	1.218033	0.49696
OS9	75.94693	92.53002	1.218351	0.002207
CAV2	4.289048	5.226187	1.218496	0.060077
TPX2	87.11273	106.1514	1.218552	0.015537
KDM8	0.847882	1.033423	1.218828	0.488463
ZFAND5	12.35054	15.05571	1.219033	0.102104
ATMIN	9.354658	11.40907	1.219614	0.118254
REEP4	16.71698	20.3937	1.219939	0.269538
TMEM126	31.35558	38.25193	1.21994	0.005823
WDR18	27.25878	33.26561	1.220363	0.13737
CNPY4	18.61803	22.7291	1.220811	0.101548
APBB2	2.534503	3.094207	1.220834	0.118105
C8orf88	10.98105	13.40932	1.221133	0.428926
MFF	32.27717	39.41775	1.221227	0.079175
IFRD2	36.47274	44.54831	1.221414	0.114668
PTPN18	8.371925	10.22886	1.221805	0.231266

SLF2	2.485905	3.038108	1.222134	0.134235
FAM92A	4.857437	5.936644	1.222176	0.021883
CTSF	14.82557	18.12004	1.222215	0.135317
CSK	24.10331	29.46647	1.222507	0.05723
ANAPC13	47.01019	57.47791	1.222669	0.029071
AK8	0.160168	0.195884	1.222992	0.622738
NDUFS7	22.83326	27.92913	1.223178	0.044219
NUDT9	5.504314	6.734401	1.223477	0.169927
SCARB2	46.58287	56.99514	1.223522	0.040754
PRAG1	0.454528	0.556143	1.223563	0.369086
DSTN	76.97686	94.20651	1.223829	0.021732
CHMP1B	13.7873	16.87684	1.224086	0.172008
TNFAIP8	4.785351	5.858177	1.22419	0.12792
PHF20	9.132888	11.18291	1.224466	0.173817
MFAP1	33.26444	40.73132	1.22447	0.081912
ZNF706	8.946543	10.95613	1.224621	0.134761
ZDHHC18	7.692309	9.421196	1.224755	0.040553
CHRAC1	12.27835	15.03923	1.224858	0.013511
CCDC137	15.20931	18.63313	1.225114	0.089348
SLC9A3R1	11.91154	14.59652	1.22541	0.275503
ISCU	22.13191	27.12441	1.225579	0.214656
CLCN6	2.559821	3.137442	1.225649	0.41522
SPEF1	0.359441	0.44056	1.225682	0.680364
GMPPB	14.11561	17.30891	1.226224	0.342639
TXNL4A	52.13509	63.93485	1.22633	0.091076
APOBEC3(	10.35511	12.7029	1.226727	0.245181
BAAT	0.048813	0.059888	1.22688	0.794487
STK24	21.39336	26.26319	1.227633	0.136328
IL1R2	0.071203	0.087416	1.227705	0.841956
UBAP1	19.19826	23.57052	1.227743	0.180348
HSP90B1	660.2959	810.7353	1.227836	0.000731
ZFYVE26	1.925028	2.363671	1.227863	0.082966
COG4	21.21765	26.05859	1.228156	0.018741
SLC25A20	14.73512	18.10783	1.228889	0.038453
ANKRD13	9.842057	12.10114	1.229534	0.173261
TMEM236	0.017926	0.022048	1.229913	0.869
AREL1	9.601072	11.80869	1.229935	0.071773
ENTPD3	0.01736	0.021353	1.229949	0.734919
MED29	14.06801	17.31045	1.230483	0.133008
FBXL8	1.533039	1.88652	1.230575	0.421485
BOLA3	54.88479	67.55659	1.23088	0.412002
ASTE1	1.857237	2.286684	1.231229	0.255783
CWC27	4.843682	5.965757	1.231657	0.255811
SURF6	14.83839	18.2795	1.231906	0.058768
SEL1L3	4.86139	5.993937	1.232968	0.336053
STAMBPL1	2.740148	3.378557	1.232983	0.15932
MYO18A	5.051339	6.228235	1.232987	0.046539
CNEP1R1	7.119947	8.77911	1.23303	0.079636
TOMM22	75.30188	92.85089	1.233049	0.141501
ARPC5L	18.46521	22.77551	1.233428	0.103252
UBE3B	5.989123	7.391383	1.234135	0.200019
WIPF2	5.91378	7.298714	1.234188	0.008819
DHODH	3.065199	3.783081	1.234204	0.22292
PDSS2	4.302673	5.311844	1.234545	0.147672
KIF23	26.07272	32.1887	1.234574	0.008919
MAP1LC3I	5.214766	6.438117	1.234594	0.347523
EEF1D	36.05773	44.52329	1.234778	0.111068
ENTPD4	10.94366	13.51377	1.234849	0.240426

SLC25A46	10.53496	13.01079	1.235011	0.098207
PIP4K2C	7.12767	8.805437	1.235388	0.093162
LRCH3	2.790702	3.447685	1.235419	0.220183
UBE2H	30.79733	38.04905	1.235466	0.034142
SNX11	5.837048	7.211525	1.235475	0.040411
FAM151B	0.735963	0.909302	1.235527	0.090321
WDTC1	11.69861	14.45406	1.235536	0.028343
DHX9	39.66834	49.02036	1.235755	0.020095
PGRMC2	23.3439	28.85059	1.235894	0.142725
PBX3	8.020925	9.91315	1.235911	0.003682
STIM2	3.234637	3.99886	1.236262	0.310534
NDUFA9	39.34995	48.65886	1.236567	0.241657
FAM120C	1.736549	2.147788	1.236814	0.026926
SLC25A10	9.259799	11.45387	1.236945	0.492885
ERLEC1	11.72029	14.49899	1.237084	0.226953
EIF5	31.46373	38.9249	1.237136	0.486243
CHD3	12.91278	15.97574	1.237203	0.000494
NUDT8	3.783861	4.681443	1.237213	0.607486
STX7	8.194239	10.14065	1.237534	0.096168
TNS3	1.520256	1.881385	1.237545	0.224091
CDKL3	0.691919	0.856312	1.23759	0.293663
ZSWIM1	4.001239	4.951978	1.237611	0.099508
ZMAT3	5.418614	6.707923	1.237941	0.109868
ATG13	12.40607	15.36243	1.2383	0.329679
ANKRD44	0.510014	0.631559	1.238317	0.268462
11-Sep	21.32985	26.41399	1.238358	0.059531
PSD3	2.482224	3.073908	1.238369	0.183826
DHX38	15.65024	19.38098	1.238382	0.092349
C12orf57	69.13056	85.62172	1.238551	0.146459
ASAP2	4.094819	5.072994	1.238881	0.137008
ESRRA	10.36544	12.85211	1.2399	0.072606
SAMD11	8.102148	10.04657	1.239988	0.761548
TMEM107	8.033428	9.963919	1.240307	0.188969
NAB2	14.61663	18.13134	1.24046	0.063219
BCAS1	0.326829	0.405436	1.240514	0.710027
FZD2	4.27021	5.297569	1.240587	0.210163
TRPS1	1.591546	1.974776	1.240791	0.127123
CFAP57	0.054582	0.067749	1.24123	0.735609
ORMDL3	9.048072	11.23284	1.241462	0.14561
PSMB4	351.6976	436.627	1.241484	0.031732
MAP4K2	3.044771	3.7834	1.242589	0.133349
PTOV1	25.69829	31.95286	1.243384	0.061673
DEF8	10.13217	12.60709	1.244263	0.013292
PAIP1	23.43685	29.16393	1.244362	0.142685
CYP26B1	5.044061	6.277655	1.244564	0.572065
STT3B	28.84157	35.90454	1.244889	0.047429
GCDH	5.070624	6.313744	1.245161	0.355676
ZNF787	10.17037	12.66491	1.245276	0.082016
RTCA	20.05877	24.97946	1.245313	0.226937
IL15RA	1.417573	1.765373	1.245348	0.220665
PIP4K2B	12.22898	15.22948	1.24536	0.022233
GTF2H5	5.467526	6.809983	1.245533	0.392321
SRSF7	43.06383	53.64058	1.245606	0.085885
DAZL	0.038164	0.047556	1.246092	0.828536
MOB4	0.009885	0.012322	1.246539	0.850698
HGS	29.2226	36.43098	1.246672	0.133522
CTR9	11.88284	14.8298	1.248001	0.033549
BET1L	21.90866	27.34271	1.248032	0.045196

EML3	10.43494	13.02618	1.248323	0.002998
LOC11226	0.033453	0.04176	1.248336	0.787309
TMED3	24.02268	29.99104	1.248447	0.247222
P3H4	17.94263	22.40687	1.248806	0.00493
LGR4	9.459939	11.82881	1.25041	0.032113
PAK4	20.2589	25.3327	1.250448	0.047284
UBE2S	180.6388	225.8796	1.250449	0.099403
MYBL1	3.11023	3.891971	1.251345	0.256918
AIP	41.64783	52.1206	1.25146	0.050995
VPS39	10.75933	13.4683	1.251779	0.163999
SOD1	241.4223	302.2885	1.252115	0.246703
NAPEPLD	1.282694	1.606188	1.252199	0.023992
ELOVL6	6.042414	7.56636	1.252208	0.166456
MOGAT1	0.035599	0.044582	1.252348	0.847249
IER2	38.57056	48.30496	1.252379	0.087875
FAM71F1	0.037268	0.046678	1.252491	0.816068
SRSF6	33.12072	41.48535	1.25255	0.049515
MRPL3	72.4417	90.81652	1.25365	0.383039
SDC2	20.00371	25.08094	1.253815	0.245712
NUP37	39.87882	50.00167	1.25384	0.242648
DHX37	7.588836	9.5155	1.253881	0.271728
OTUB1	47.5419	59.64386	1.254554	0.00709
KLHL3	0.737441	0.925187	1.254591	0.25848
NARF	9.237609	11.59594	1.255297	0.126819
NFE2	0.335214	0.420867	1.255517	0.58451
PHAX	14.10574	17.71352	1.255767	0.045615
SMURF2	4.535842	5.6967	1.25593	0.07808
RELA	28.49712	35.80567	1.256466	0.051026
PPA2	35.88988	45.11659	1.257084	0.239264
PLAC8L1	0.098485	0.123812	1.257166	0.496654
CDK9	18.84226	23.68992	1.257276	0.033209
APP	128.8227	161.969	1.257302	0.008639
DGCR6L	42.78456	53.80069	1.257479	0.145878
DUSP3	24.96669	31.39602	1.257517	0.015651
RPGR	2.984554	3.753671	1.257699	0.14619
PATL2	0.082355	0.103582	1.257741	0.646269
NDUFB4	89.44864	112.5127	1.257847	0.00308
GPKOW	21.02342	26.46695	1.258927	0.047063
WIPF1	6.715072	8.454248	1.258996	0.036808
GLB1L	2.646306	3.332739	1.259393	0.422351
TSR2	14.89032	18.76555	1.260252	0.167717
ENY2	21.6928	27.36817	1.261625	0.0574
HABP4	11.37091	14.36074	1.262937	0.064618
TMEM131	6.151078	7.768432	1.262938	0.190329
RRP36	37.12807	46.89349	1.26302	0.155623
MRPL50	39.61229	50.04445	1.263356	0.125124
SEMA3C	6.082177	7.68397	1.263358	0.378595
SPIN1	16.33582	20.64369	1.263707	0.096926
PNPLA1	0.006241	0.007889	1.264141	0.855424
SERPINB5	0.014079	0.017798	1.264141	0.855424
MASP2	0.014184	0.01793	1.264141	0.855424
STRADB	18.92946	23.93252	1.2643	0.333288
SRD5A1	4.951069	6.261669	1.264711	0.054871
PIGN	3.903979	4.937835	1.264821	0.026629
GYPC	15.75608	19.93116	1.264983	0.168707
ERC1	3.784808	4.788456	1.265178	0.154212
LMLN	1.632615	2.065681	1.26526	0.389636
DNM2	34.63232	43.82513	1.26544	0.00476

ZNF654	1.938816	2.454021	1.265732	0.020166
EXOC4	11.70937	14.82818	1.266352	0.113391
ZNF101	1.836755	2.326952	1.266882	0.222515
KMO	0.013029	0.016508	1.267033	0.799857
PIGX	9.11541	11.55243	1.267351	0.172794
ZNF576	7.317553	9.275219	1.26753	0.064366
TMEM245	10.80732	13.6993	1.267595	0.004761
NT5DC1	10.07016	12.76915	1.268018	0.19418
PIAS2	1.318614	1.672228	1.268171	0.114278
PITPNM2	1.851592	2.348457	1.268345	0.192622
TIGAR	2.264739	2.872803	1.268492	0.213492
PITPNA	19.67134	24.96724	1.269219	0.010498
CHST12	6.238417	7.918391	1.269295	0.114546
SEC11C	41.32061	52.45019	1.269347	0.079986
MFSD11	2.804135	3.559423	1.269348	0.239975
CPOX	8.224039	10.4394	1.269376	0.272902
ATRIP	5.58709	7.092465	1.269438	0.213662
SLC35A5	7.179541	9.115405	1.269636	0.050699
STX8	28.4506	36.12722	1.269823	0.117526
ZFYVE1	4.839062	6.14478	1.269829	0.219916
ROPN1	0.028314	0.035955	1.269859	0.839145
KIF20A	23.45791	29.79486	1.270142	0.118093
NCBP1	11.75355	14.9302	1.270272	0.11655
CAPZA2	31.82793	40.43566	1.270446	0.087263
KCTD12	6.784385	8.620124	1.270583	0.391727
SELENOM	72.1293	91.64644	1.270585	0.174181
LAMB1	28.02148	35.60597	1.270667	0.093465
FAM228B	1.98482	2.523153	1.271225	0.284945
SMYD1	0.008554	0.010876	1.271341	0.852272
CREG1	46.5977	59.25936	1.271723	0.1287
SHPK	4.322089	5.498384	1.272159	0.147607
CCDC88A	3.997683	5.087058	1.272502	0.20602
ARAF	12.6527	16.10184	1.272601	0.051053
ZNF324	2.336772	2.974341	1.272842	0.145235
EIF5B	51.74584	65.88466	1.273236	0.170661
COQ9	27.47409	34.98784	1.273485	0.167561
PRMT7	4.397878	5.602043	1.273806	0.074956
GORAB	3.795735	4.83568	1.273977	0.036942
GRHPR	30.00135	38.2211	1.273979	0.139729
R3HCC1	15.33023	19.53112	1.274027	0.017303
FAM45A	5.484814	6.989125	1.274268	0.014728
PLOD3	38.27847	48.78173	1.274391	0.063066
KLHL26	2.556748	3.25838	1.274423	0.071384
SLC37A2	2.312245	2.947075	1.274551	0.381209
SUN2	21.51394	27.43373	1.27516	0.087324
DUS3L	7.81414	9.964988	1.275251	0.061141
BMP2K	2.722087	3.471552	1.275327	0.058534
CDC42SE2	6.34174	8.089661	1.275622	0.056749
PIK3C3	4.7377	6.043999	1.275724	0.126671
RRAGA	37.37108	47.67928	1.275834	0.014631
DNAJC21	7.413224	9.458593	1.275908	0.013149
SUMO4	0.055396	0.070691	1.276121	0.77063
MCCC1	6.407231	8.176595	1.276151	0.068904
ZDHHC7	18.86798	24.08376	1.276435	0.005714
WASHC4	4.898125	6.252836	1.276577	0.121881
ANXA11	15.36556	19.61694	1.276683	0.178092
CLDND1	57.63967	73.58796	1.276689	0.098321
SLC25A14	4.228458	5.403198	1.277818	0.263955

KPNA1	11.21412	14.33238	1.278066	0.046492
ZNF408	3.795328	4.850852	1.278111	0.438378
LRPAP1	10.97725	14.0313	1.278216	0.019615
FOXE3	0.065759	0.08406	1.278308	0.736496
PDK3	4.051139	5.178825	1.278363	0.068191
ARL6IP4	76.55963	97.87129	1.278367	0.01729
LDAH	4.795105	6.130679	1.278529	0.109269
TBL2	12.46705	15.94367	1.278865	0.03953
UBE3A	6.781763	8.67304	1.278877	0.005471
PATL1	17.52038	22.40747	1.278937	0.030131
PRSS23	17.8031	22.77	1.278991	0.115302
MAP3K5	1.806685	2.310913	1.27909	0.105737
JAK2	1.409324	1.803042	1.279367	0.097317
TRIP11	3.693636	4.729993	1.280579	0.098678
SPTAN1	22.69706	29.07548	1.281024	0.076583
RAE1	27.56996	35.32068	1.281129	0.060232
LIMD1	6.588828	8.441152	1.281131	0.111957
UBE4B	6.692822	8.575563	1.281307	0.022409
TEX2	2.62394	3.362523	1.281479	0.008896
WDR53	4.478338	5.740051	1.281737	0.123185
APOM	1.974993	2.531846	1.281952	0.157938
PDCD5	62.49366	80.13895	1.282353	0.073844
CADPS2	1.879975	2.410866	1.282393	0.149163
LOC10192	0.595787	0.764122	1.282542	0.235525
PIP4K2A	11.1116	14.25766	1.283133	0.00563
STAM2	4.226518	5.423322	1.283166	0.053891
BCAS2	60.1346	77.16618	1.283224	0.1916
ZC3H4	1.98499	2.548495	1.283883	0.13813
NOTCH2N	0.555588	0.713654	1.284504	0.207476
HPSE2	0.013872	0.017826	1.285084	0.666998
SLC35F6	6.503117	8.359175	1.28541	0.276924
PLXNA2	1.070115	1.375648	1.285515	0.562116
SPIRE1	5.275392	6.783085	1.285797	0.314563
SNX12	23.53688	30.26375	1.285801	0.034582
BICD1	1.152436	1.482373	1.286295	0.033167
DEGS1	43.66601	56.16804	1.28631	0.002844
PLEKHG4	2.116925	2.723366	1.286473	0.451253
NCBP2	15.28173	19.66144	1.286598	0.000346
CDC26	14.45024	18.59181	1.28661	0.114718
CD200R1	0.027583	0.035491	1.286695	0.837274
FAM98B	12.83624	16.51687	1.286737	0.071581
FASTKD3	4.264345	5.487594	1.286855	0.278122
BNC2	0.337981	0.434934	1.286861	0.194468
FIS1	82.75867	106.5011	1.286888	0.035929
MPV17	12.37958	15.93703	1.287364	0.202286
USP3	3.960912	5.099672	1.287499	0.005066
ARHGEF3	2.227511	2.86835	1.287693	0.002058
RBM45	5.252133	6.765667	1.288175	0.233263
KPTN	3.704416	4.774737	1.288931	0.061349
NBN	5.889029	7.592168	1.289205	0.258955
TRNT1	3.496529	4.508161	1.289324	0.022719
ACADVL	80.48709	103.7757	1.289346	0.077753
DUSP1	60.73082	78.3132	1.289513	0.462519
SYNCRIP	20.31632	26.2058	1.289889	0.078041
TMEM147	123.6159	159.4618	1.289978	0.002393
SPTBN1	13.92374	17.96216	1.290039	0.159265
ELMO2	10.52045	13.57447	1.290294	0.10145
EIF4E2	18.31091	23.62656	1.2903	0.02787

AXDND1	0.034506	0.044539	1.290747	0.687665
RARB	10.93252	14.11167	1.290797	0.039243
ARMC6	12.42225	16.03886	1.29114	0.140245
EIF3J	37.49117	48.41899	1.291477	0.062839
APEX2	21.46457	27.72382	1.291609	0.102234
COPS6	98.54872	127.328	1.292031	0.015997
TSTA3	16.07116	20.76683	1.29218	0.019675
TMEM134	9.968486	12.88162	1.292235	0.08644
C7orf61	0.670797	0.866847	1.292265	0.284488
TOP1	29.87988	38.61689	1.292404	0.006251
ARPP19	22.40503	28.9594	1.29254	0.015883
PIK3CA	2.230098	2.882579	1.29258	0.024796
CCDC13	0.073302	0.094771	1.292889	0.64815
TMEM201	3.466757	4.482383	1.292961	0.003657
STN1	3.560228	4.604645	1.293357	0.081634
DDX54	19.86633	25.69777	1.293534	0.130655
SPEF2	0.482279	0.624013	1.293883	0.139911
ABCA4	0.056437	0.073026	1.293933	0.72403
GNAQ	8.300302	10.74053	1.293992	0.043588
ARL2	118.759	153.6853	1.294094	0.040669
ESR1	0.006069	0.007855	1.29421	0.688569
AKR7L	0.280775	0.363404	1.294289	0.481361
HSP90AB1	603.4805	781.1879	1.294471	0.040901
C17orf107	0.036511	0.047267	1.294591	0.808488
SMIM19	14.07594	18.22611	1.294842	0.346913
FAM210A	3.242509	4.19865	1.294877	0.049449
MPZL1	29.15381	37.7547	1.295018	0.141117
SFXN5	2.727033	3.532528	1.295374	0.046273
MITF	2.662443	3.448914	1.295394	0.042372
STRA8	0.01166	0.015105	1.295437	0.760676
CALCR	0.009507	0.012316	1.295437	0.760676
B4GALT4	14.41991	18.68138	1.295527	0.005975
HUS1	6.63641	8.598165	1.295605	0.019324
NDUFAF6	2.273712	2.946085	1.295716	0.179263
TAGLN2	395.8675	512.9997	1.295887	0.118221
TUBB	421.7746	546.8618	1.296574	0.097105
MAF1	45.30838	58.75437	1.296766	0.048447
TMEM267	1.400885	1.816755	1.296862	0.255824
MAPK8IP1	8.705822	11.29272	1.297145	0.036977
VPS26C	10.97535	14.23948	1.297406	0.002628
DIAPH1	18.87526	24.49286	1.297617	0.025501
TSC22D4	13.00722	16.88647	1.298238	0.041537
CCAR2	21.02264	27.30906	1.299031	0.00818
NFIB	1.122344	1.458069	1.299129	0.143464
PEX13	3.813317	4.955047	1.299406	0.115753
ORAOV1	6.138445	7.976732	1.299471	0.189729
TRIP4	11.94372	15.52135	1.299541	0.001376
LEPROTL1	9.250313	12.02365	1.29981	0.092595
PCNX4	9.392132	12.20809	1.299821	0.097511
TUBA8	0.456879	0.593898	1.299903	0.498454
GANC	1.588336	2.065154	1.3002	0.155411
SETD3	14.37658	18.69843	1.300617	0.024477
CMTM2	0.071504	0.093005	1.300704	0.55695
ALOXE3	0.790461	1.029083	1.301877	0.686217
TNF	0.244921	0.318932	1.302183	0.750608
LOC10798	0.648786	0.844911	1.302296	0.106169
ZFP36	6.464167	8.418572	1.302344	0.437696
WISP3	0.017294	0.022524	1.302424	0.838991

RABEPK	13.13857	17.11626	1.302749	0.273521
RNF121	9.468663	12.33562	1.302783	0.100403
ARHGEF4(	5.473355	7.132273	1.30309	0.020849
PRMT2	4.570707	5.957142	1.303331	0.005354
RNF141	8.253384	10.7576	1.303417	0.171509
TRPM7	3.14386	4.098345	1.303603	0.056376
RIOK2	7.55491	9.856701	1.304675	0.21807
LOC10537	0.029794	0.038878	1.304871	0.744356
ELOA	15.00888	19.58853	1.305129	0.125979
ERMAP	0.996235	1.301085	1.306003	0.294135
YIF1B	26.29327	34.34505	1.30623	0.01582
NOX5	0.02441	0.031886	1.306235	0.84443
NMRK1	3.478387	4.543894	1.306322	0.155926
GOLGA2	20.17658	26.36196	1.306562	0.203591
NPIPA8	1.285213	1.680005	1.30718	0.409508
KRT81	0.259063	0.338714	1.307459	0.779516
SLC38A2	48.45323	63.35432	1.307536	0.107441
KLHDC10	10.47655	13.69942	1.307627	0.076796
NRBP2	4.866952	6.364432	1.307683	0.159564
ERI3	48.87309	63.91284	1.307731	0.05736
ZNF658	0.407524	0.532932	1.307733	0.444777
SRRT	26.65788	34.86398	1.30783	0.18889
ARID5B	2.227177	2.913217	1.308031	0.062029
OSER1	8.853508	11.58381	1.308387	0.093135
CDCA3	15.3867	20.13189	1.308396	0.016381
SH3D21	1.736457	2.272369	1.308623	0.028844
B4GALT5	12.5377	16.40928	1.308795	0.07325
C12orf10	42.22145	55.29593	1.309664	0.039724
TMEM54	29.25863	38.32509	1.309873	0.113383
SRXN1	15.20406	19.9163	1.309933	0.107733
LOC10099	0.186352	0.244152	1.310165	0.137551
CYP2U1	1.386051	1.816391	1.310479	0.240748
KDELR2	100.199	131.383	1.31122	0.122934
CCDC22	9.299027	12.19434	1.311357	0.003485
OXSR1	18.67979	24.50244	1.311709	0.006192
HGSNAT	7.986649	10.4762	1.311714	0.085903
THNSL1	2.047257	2.686032	1.312015	0.25629
NDUFS8	146.1165	191.7493	1.312305	0.003924
RTF1	12.66252	16.62523	1.312948	0.052755
ANKRD55	0.008619	0.011319	1.313231	0.814291
HEATR4	0.008605	0.0113	1.313231	0.814291
CCDC32	4.508363	5.922066	1.313573	0.20532
MROH8	0.345641	0.454202	1.314087	0.41018
SPATA2	4.670704	6.137826	1.314111	0.135793
FAM20B	14.62112	19.21944	1.314498	0.003992
EVI5L	6.940717	9.125618	1.314795	0.034024
IQCD	0.777279	1.02203	1.314881	0.41131
FAM120A(	8.620714	11.33841	1.315252	0.288171
GSK3B	6.375976	8.386846	1.315382	0.003521
OVCA2	1.438732	1.892899	1.315672	0.27847
C7orf43	5.472535	7.202586	1.316133	0.267793
SV2A	16.35281	21.52454	1.316259	0.040427
PUM3	21.20094	27.91279	1.316582	0.158842
CNTNAP1	2.88318	3.796633	1.316821	0.2346
CCDC159	2.489737	3.279012	1.317011	0.096618
LUZP1	3.008339	3.962694	1.317236	0.09502
AMFR	23.92935	31.52062	1.317237	0.006255
MTMR10	2.585407	3.406594	1.317624	0.013576

MALSU1	35.54466	46.84211	1.317838	0.056051
PIAS3	13.37846	17.63364	1.318063	0.008624
NDUFA11	53.16191	70.07149	1.318077	0.024552
GOSR2	4.847897	6.392339	1.31858	0.189916
PRELID2	0.870983	1.148694	1.318847	0.167303
C1orf53	2.47817	3.27029	1.319639	0.330363
CD99L2	16.98634	22.41776	1.319753	0.077849
CEP76	1.944818	2.568246	1.320558	0.275718
ZFYVE9	4.082827	5.391625	1.320562	0.032972
LRTM2	0.008571	0.011321	1.320894	0.740324
NPY4R2	0.004528	0.005981	1.320894	0.740324
STAU1	34.62477	45.73719	1.320938	0.001782
CDC20	79.8358	105.4699	1.321085	0.104677
ZNF407	0.233111	0.308211	1.322164	0.078901
AKTIP	7.802618	10.31666	1.322205	0.011452
CDX1	0.020754	0.027447	1.322484	0.809723
CNTD1	0.110361	0.146016	1.323072	0.559775
TMEM184	26.20381	34.67406	1.323245	0.047081
SLC25A32	9.623023	12.73608	1.323501	0.046741
HGFAC	0.080055	0.105999	1.324086	0.782489
MIPEP	4.416633	5.848465	1.324191	0.387436
POC1A	11.53583	15.27804	1.3244	0.028156
COPS8	34.85612	46.16631	1.324482	0.017289
DNAJC9	27.74033	36.74397	1.324568	0.005503
PLK1	52.12575	69.04627	1.32461	0.127056
IDS	25.77868	34.14739	1.324637	0.096513
MRPL18	74.80253	99.1091	1.324943	0.028128
CNOT7	5.860977	7.765675	1.32498	0.001324
DUT	31.60197	41.88942	1.325532	0.024555
IDNK	0.413788	0.548529	1.325628	0.49964
MAIP1	9.695721	12.85367	1.325705	0.092555
RPL26L1	33.38264	44.26061	1.325857	0.051177
IMPA2	8.681574	11.51143	1.325961	0.317317
LURAP1L	3.533295	4.685552	1.326114	0.044011
DCSTAMP	0.027471	0.036434	1.326259	0.745677
PPP2R5E	5.760207	7.641019	1.326518	0.040207
RILPL2	7.02922	9.325209	1.326635	0.203513
PTDSS1	31.53089	41.83252	1.326715	0.113599
RALBP1	12.25723	16.27447	1.327745	0.009131
ABCF2	23.50374	31.21189	1.327954	0.146144
SAMD12	0.21541	0.286103	1.328175	0.1532
EFCAB6	0.180514	0.239759	1.328203	0.358854
CTNNA1	59.65984	79.2436	1.328257	0.0035
MRPS18C	28.60386	38.00454	1.328651	0.163889
ARMC1	12.69494	16.8691	1.328805	0.076411
EIF2B2	40.13549	53.34077	1.329018	0.011244
F8A3	0.100058	0.132987	1.329104	0.584872
PHLDB1	2.176298	2.894085	1.32982	0.040342
PRUNE2	1.075219	1.43002	1.329981	0.223823
SLC39A3	9.610521	12.783	1.330105	0.019007
ZFP69	2.125918	2.827729	1.330121	0.287186
FIG4	7.932362	10.55295	1.330366	0.064481
NR2C2AP	12.95011	17.23088	1.330558	0.178821
RARA	4.589556	6.107084	1.330648	0.039713
HCCS	7.25395	9.653515	1.330794	0.078269
PLEKHG5	2.108103	2.805968	1.331039	0.024117
GATB	9.859164	13.12418	1.331166	0.201156
PPP2R1A	88.58236	117.92	1.33119	0.002188

ANXA2R	1.821447	2.424735	1.331213	0.21178
PPDPF	261.0596	347.5314	1.331234	0.06846
LOC1009	0.007293	0.009711	1.331593	0.806612
SLC1A2	0.050273	0.066963	1.331994	0.41553
MKI67	13.83012	18.43272	1.332796	0.059582
TBPL1	15.49854	20.65797	1.332898	0.078225
PLEKHF1	2.63107	3.507236	1.333007	0.113166
MSL3	5.238944	6.984302	1.333151	0.002214
C16orf70	5.255547	7.013196	1.334437	0.027297
ATRAID	89.94617	120.0352	1.334523	0.047283
HIST1H2B	0.164273	0.219252	1.334683	0.771336
UBE3C	24.33543	32.48191	1.334758	0.015802
TSR3	33.23392	44.36092	1.334808	0.023985
ZSCAN5B	0.038509	0.051404	1.334854	0.769409
GOPC	13.5402	18.07423	1.334857	0.338203
TXNRD2	5.179232	6.914195	1.334984	0.065485
NAIF1	2.68164	3.580497	1.335189	0.092641
PGM1	42.67178	56.97948	1.335297	0.038571
TMEM167	19.63454	26.22069	1.335437	0.119418
SNPH	2.148556	2.869372	1.335488	0.084212
GANAB	168.9569	225.6647	1.335635	0.020999
ERGIC2	8.897165	11.88379	1.335682	0.115426
IGF2R	10.67108	14.25674	1.336017	0.072606
MRPL54	49.97871	66.77549	1.336079	0.006588
YAP1	10.1899	13.6155	1.336176	0.073623
PARD3	6.399875	8.55192	1.336264	0.015625
OXSM	2.511531	3.356797	1.336554	0.270493
STX5	29.83276	39.87902	1.336752	0.026786
PSMB3	227.6421	304.3789	1.337094	0.005465
EIF5AL1	3.597412	4.811105	1.33738	0.050876
THAP11	15.75702	21.07654	1.337597	0.023767
TBC1D8B	2.347013	3.140355	1.338022	0.037567
FAM198B	0.252028	0.337282	1.33827	0.167409
SLC25A1	56.2958	75.3521	1.338503	0.135653
ITPR3	9.495755	12.71712	1.339242	0.015365
RASSF8	3.88227	5.200031	1.33943	0.076916
BROX	8.224722	11.01839	1.339667	0.08807
NXT2	3.738498	5.008495	1.339708	0.075721
ERP44	19.70389	26.4001	1.339842	0.013433
CERS2	70.1744	94.04336	1.340138	0.029347
ASCC2	9.505195	12.7415	1.340478	0.025161
MED10	36.17745	48.49546	1.340489	0.065106
PCBP4	18.49055	24.7909	1.340733	0.121311
MANF	138.3473	185.4994	1.340824	0.000709
KPNA4	11.08307	14.8651	1.341244	0.035865
FBXO36	1.621865	2.175897	1.341602	0.047688
EXOC5	6.887764	9.240867	1.341635	0.058401
ABCC4	3.993478	5.358306	1.341764	0.144544
ZCCHC17	19.05714	25.57335	1.34193	0.040196
SECISBP2L	3.185351	4.275469	1.342229	0.179326
SEC24D	16.76937	22.51014	1.342336	0.178387
MRPL38	30.1101	40.41884	1.342368	0.141394
EID2	16.89636	22.68752	1.342746	0.296745
RBL1	5.227116	7.024731	1.343902	0.00544
BMP1	4.698621	6.314667	1.34394	0.093388
GTF2E2	17.09231	22.97387	1.344106	0.05255
KLRC3	0.475022	0.638538	1.344227	0.627087
RPUSD1	7.309982	9.8286	1.344545	0.261739

ZNF865	4.712637	6.336463	1.344568	0.038616
CFAP36	21.86853	29.40415	1.344588	0.084434
SHANK1	0.400742	0.539017	1.345047	0.27381
C1orf123	32.54188	43.782	1.345405	0.052489
ROPN1L	0.782059	1.052838	1.346238	0.092909
TIGD6	1.63188	2.196973	1.346284	0.243344
SMIM10L1	5.122741	6.899389	1.346816	0.153974
HELQ	1.787537	2.407675	1.346923	0.296385
ANAPC2	11.57057	15.58895	1.347294	0.068919
SLC16A12	0.059956	0.080781	1.347347	0.177888
PSKH1	8.681921	11.70043	1.347678	0.268635
NT5C2	3.663713	4.938447	1.347935	0.02786
SPCS3	24.65857	33.24212	1.348096	0.000848
ABCC2	0.388559	0.523862	1.348217	0.353798
AP4B1	7.213769	9.727345	1.348441	0.064995
ABI3	0.034734	0.046844	1.348643	0.699242
PPP3CB	9.422736	12.70812	1.348666	0.027053
CCNQ	9.722616	13.11568	1.348987	0.037955
CNP	12.52324	16.89564	1.349143	0.011947
NSFL1C	14.64161	19.76099	1.349646	0.012691
H2AFY	14.95396	20.18526	1.349827	0.005592
GDF15	16.22805	21.90645	1.349912	0.60858
EID3	0.205477	0.277486	1.350452	0.536012
ZCCHC4	1.866154	2.520725	1.35076	0.011716
APC	2.413335	3.260417	1.351001	0.181852
SF3B5	149.3319	201.8015	1.351362	0.012989
EVA1B	4.810358	6.500706	1.351398	0.214452
WDR61	20.10878	27.18244	1.35177	0.011237
DCLRE1C	1.090732	1.474695	1.352023	0.056483
UNC45A	18.33173	24.79112	1.352361	0.003271
ATP2C1	13.51814	18.28427	1.352572	2.20E-05
C7orf50	11.04929	14.94499	1.352574	0.128886
OPA3	2.999354	4.05697	1.352614	0.002021
PPP1R16A	6.88914	9.318582	1.352648	0.021454
CBX7	4.381937	5.927483	1.352708	0.01322
C6orf201	0.012981	0.01757	1.353477	0.794074
PPP3CC	4.714309	6.380889	1.353515	0.008353
PAGR1	14.28481	19.33613	1.353614	0.148528
MTUS2	0.110798	0.149983	1.353654	0.420945
HOGA1	0.225124	0.304773	1.353805	0.432501
PPIC	37.1015	50.24737	1.354322	0.001991
PCOLCE	27.50814	37.27125	1.354917	0.364071
ANKRD9	6.736306	9.12714	1.354918	0.147791
SMIM7	26.64606	36.11667	1.355422	0.055039
EHD4	9.821721	13.31642	1.355813	0.071686
SNX29	0.897441	1.217363	1.356483	0.02724
PRMT6	7.772321	10.54444	1.356666	0.424577
TRIQK	4.57353	6.204975	1.356715	0.010255
TMEM86A	2.174875	2.95112	1.356915	0.692908
TANGO6	2.766567	3.754948	1.357259	0.174977
ZNF780B	0.773213	1.049797	1.357707	0.282574
HMGN2	121.141	164.4962	1.357891	0.029619
ZNF672	6.335208	8.602825	1.357939	0.001033
STARD3NL	23.16949	31.47029	1.358264	0.012351
TRIP13	22.23205	30.19856	1.358335	0.00102
VPS37C	9.018482	12.25119	1.358454	0.011303
STK39	3.119992	4.238637	1.358541	0.051301
VAPB	12.42566	16.88851	1.359165	0.061583

FLII	29.34935	39.89241	1.359226	0.002121
MAP1LC3I	52.05892	70.76432	1.359312	0.248554
CDK5R2	0.512613	0.696876	1.359457	0.057786
SECTM1	1.326385	1.805958	1.361564	0.071599
NIP7	15.73082	21.42093	1.361717	0.093535
VPS35	23.66217	32.23664	1.362371	0.006721
NDUFC1	31.72199	43.23251	1.362856	0.049381
SLC35B1	30.63524	41.76077	1.363161	0.026923
ECSIT	20.767	28.31973	1.363689	0.080067
TMEM167	25.51685	34.8066	1.364063	0.01712
TEDC1	10.71802	14.62484	1.36451	0.020758
PCGF5	3.837425	5.236225	1.364515	0.012368
FOS	33.32648	45.48165	1.36473	0.631019
KRT222	2.276928	3.107548	1.364799	0.204389
SPC25	17.70927	24.17249	1.364962	0.022471
AP3S1	12.46133	17.00946	1.364979	0.022106
ZNF510	1.075445	1.468228	1.365228	0.182503
FDFT1	23.78958	32.49175	1.365797	0.176626
TMEM248	16.17729	22.11049	1.366761	0.013971
TRIM25	6.148668	8.404337	1.366855	0.020253
TMEM87A	7.065082	9.666455	1.368201	0.1111
ATP5PF	43.95129	60.13733	1.368272	0.02136
TOMM5	106.4797	145.7326	1.368642	0.031162
PES1	31.48345	43.11019	1.369297	0.043068
CSKMT	1.048329	1.435598	1.369415	0.221065
SPART	10.90374	14.932	1.369438	0.025228
ZNF526	3.587775	4.914261	1.369724	0.211216
PSME3	26.49331	36.29692	1.370041	0.126991
IVD	6.658523	9.123222	1.370157	0.024088
UBXN1	69.67879	95.48067	1.370297	0.024612
FAM96A	11.29222	15.48897	1.37165	0.103278
EIF2B5	17.41311	23.89307	1.372131	0.02698
DHX32	12.80876	17.57872	1.372398	0.134243
CYC1	44.78504	61.47248	1.372612	0.013841
TPT1	406.0989	557.4696	1.372743	0.012459
LRR1	13.7561	18.88737	1.373018	0.120647
SBNO2	11.50668	15.80195	1.373284	0.212122
MCL1	54.69897	75.13565	1.373621	0.001095
FBXO6	0.323478	0.444349	1.37366	0.15314
NDUFA13	326.6408	448.7191	1.373739	0.004512
C12orf45	14.77387	20.32522	1.375755	0.084908
EEF1AKMT	3.831388	5.271805	1.375952	0.220351
EDEM1	9.183907	12.63719	1.376015	0.023936
TCF4	1.477027	2.033285	1.376607	0.086179
FBXO38	5.807788	7.997596	1.377047	0.070485
SNAP23	10.60025	14.59811	1.377148	0.045744
CD101	0.075512	0.104041	1.377801	0.509981
TRPC4AP	46.8889	64.6196	1.378143	0.055777
NID2	7.509069	10.34883	1.378177	0.061617
BORCS7	4.868961	6.712911	1.378715	0.164954
ZNF268	2.158963	2.977847	1.379295	0.171547
SPRYD4	3.479269	4.799117	1.379346	0.25433
PYROXD1	2.394006	3.303656	1.37997	0.104067
SUOX	1.33204	1.838254	1.380029	0.207069
NEDD4L	5.948983	8.21177	1.380365	0.099037
NUDT1	33.89403	46.79489	1.380623	0.028036
POLR2H	25.6985	35.48616	1.380865	0.028767
SMIM29	13.75905	19.00444	1.381231	0.017278

VWF	0.014842	0.020504	1.381509	0.386138
NIPAL3	4.210939	5.817637	1.381553	0.02179
ACAP3	6.610199	9.133993	1.381803	0.086823
GCC1	6.949087	9.603	1.381908	0.077603
EDN1	0.293437	0.405704	1.382592	0.165815
LSM1	28.61556	39.57931	1.38314	0.08557
SWAP70	9.117618	12.6184	1.383958	0.009718
LTBR	1.590952	2.203544	1.385048	0.251427
TMEM243	6.284931	8.705533	1.385144	0.044719
SETMAR	1.128052	1.562761	1.385363	0.151825
ACLY	48.32511	66.9689	1.385799	0.026937
CCIN	0.096013	0.133068	1.385947	0.734625
ATP5MF	73.68515	102.1523	1.386335	0.001707
BACE2	5.294921	7.340852	1.386395	0.023464
SCYL1	31.59436	43.80439	1.386462	0.007724
STX10	32.32704	44.83079	1.386789	0.002682
RECQL	16.4923	22.87522	1.387025	0.003013
BEAN1	0.369197	0.512117	1.387111	0.332623
MTCP1	1.073221	1.488997	1.38741	0.284895
APLF	1.014084	1.407019	1.387478	0.226987
PCBP1	101.9381	141.4777	1.387879	0.001432
ATG10	0.942393	1.308799	1.388804	0.306155
CD9	20.35099	28.26477	1.388864	0.07631
SLC2A8	5.926709	8.232632	1.389073	0.062666
PPARD	3.055024	4.244928	1.389491	0.005458
ATP2B4	17.06125	23.70855	1.389614	0.087422
AQR	6.369005	8.850514	1.389623	0.017633
AGGF1	9.968153	13.85493	1.38992	0.100282
SIPA1	7.709425	10.71697	1.390112	0.030442
WDR77	23.10324	32.13596	1.390972	0.254715
NOL6	7.45052	10.36756	1.391522	0.060216
ERCC8	5.529076	7.694257	1.391599	0.183444
RTL6	9.093428	12.65493	1.391657	0.011264
CST3	45.28969	63.03565	1.391832	0.005353
FBXO8	6.188928	8.61458	1.391934	0.032126
POLR2C	47.02582	65.48571	1.392548	0.046372
DNAJB5	8.757966	12.19618	1.392582	0.120742
SRD5A3	5.570141	7.75769	1.392728	0.275926
PMM2	10.08313	14.04322	1.392745	0.021393
ARHGAP1	11.12419	15.49951	1.393315	0.018152
WWP2	5.118773	7.132254	1.393352	0.034461
RAN	219.8086	306.3073	1.393518	0.129268
SLC35D1	3.634981	5.069758	1.394714	0.101511
NAA38	21.02578	29.32638	1.394782	0.040778
GNPTAB	4.581622	6.390821	1.394882	0.004255
DMAC2	22.46132	31.33773	1.395187	0.017829
TBC1D10A	12.02696	16.78082	1.395268	0.139918
NIT2	34.78385	48.54789	1.395702	0.215255
MRPS23	35.70175	49.83833	1.395963	0.053119
CENPP	2.297217	3.207126	1.396092	0.112863
CGRRF1	5.720289	7.98739	1.396326	0.074095
ZNF836	0.971286	1.356627	1.396733	0.034375
AIMP2	13.49492	18.85257	1.397013	0.134235
C6orf226	4.998339	6.983226	1.397109	0.078592
DNAL1	2.592654	3.622293	1.397137	0.0365
TMEM116	2.236987	3.126375	1.397583	0.047119
CDK14	0.713275	0.997039	1.397832	0.035349
FER	1.249615	1.747388	1.398341	0.132454

RGS19	2.342087	3.275149	1.398389	0.012243
TAS1R2	0.022771	0.031852	1.398817	0.805707
HMG20B	56.08164	78.47337	1.39927	0.032911
RBX1	71.29791	99.80146	1.399781	0.030115
SAMD4A	2.977458	4.170771	1.400783	0.102352
PPP1R3G	0.587312	0.823486	1.402128	0.572382
MAP4	29.96332	42.02062	1.402402	0.026205
TXNDC9	15.16946	21.28167	1.402928	0.046754
GTF2IRD2	0.13034	0.182968	1.403781	0.267359
TRIR	227.4166	319.2841	1.403961	0.011355
TRIM3	4.208532	5.908749	1.403993	0.050016
BZW1	51.65293	72.52779	1.404137	0.005443
VPS4A	54.83212	77.00206	1.404324	0.003811
C8orf37	0.686275	0.963846	1.404461	0.18182
IK	78.85519	110.7688	1.404712	0.001402
UTP4	26.76788	37.61143	1.405095	0.085283
SELENOH	42.0306	59.08907	1.405859	0.017127
AHRR	6.21644	8.741994	1.40627	0.213465
BRCC3	4.440441	6.244891	1.406367	0.115765
CHTF8	30.20139	42.48654	1.406774	0.048412
TIMMDC1	47.27267	66.54264	1.407635	0.170604
HHIPL2	1.906656	2.684618	1.408024	0.499104
ALG5	36.05924	50.78138	1.408276	0.094409
TYRO3	6.181362	8.705486	1.408344	0.033645
ZNF574	3.733897	5.259534	1.408591	0.097076
CENPBD1	2.601645	3.664664	1.408595	0.320739
PPHLN1	5.615469	7.91043	1.408686	0.027737
PVR	16.60389	23.39064	1.408745	0.029175
VPS26A	12.55761	17.69291	1.408939	0.00572
AHSA1	98.88249	139.3375	1.409122	0.01974
PKD2	4.837339	6.817376	1.409323	0.064337
LOC10798	0.016902	0.023823	1.409425	0.687097
PPP2R5B	12.71135	17.92157	1.409888	0.016961
PHF13	9.344561	13.17735	1.410163	0.024343
MEA1	31.55553	44.50754	1.410451	0.020038
JAK1	26.3616	37.19046	1.410782	0.014909
ZNF490	0.9875	1.393963	1.411608	0.084167
PMVK	18.14348	25.63474	1.41289	0.088874
FAM162A	91.13364	128.8042	1.413355	0.226065
CEP250	7.619485	10.7726	1.413822	0.084594
MAT2B	17.28101	24.43771	1.414136	0.099792
CYSRT1	0.775744	1.097114	1.414274	0.555915
SPOPL	2.381508	3.368309	1.414359	0.03025
LPIN2	3.569076	5.048359	1.414472	0.012309
CEP63	4.875154	6.902191	1.415789	0.033898
MC1R	2.068429	2.928668	1.41589	0.078994
ACAD9	19.83574	28.09223	1.416243	0.043047
MLEC	30.4878	43.20277	1.417051	0.001317
IGSF10	0.111104	0.15747	1.417323	0.402159
SPATA20	7.47226	10.59355	1.417718	0.04733
DACT1	3.69507	5.238741	1.417765	0.003322
LRRC37A3	0.335107	0.475282	1.418302	0.223286
RNF25	14.05738	19.94009	1.418479	0.148619
SLC23A3	0.133415	0.189268	1.418646	0.534642
EFNA5	2.996259	4.252751	1.419354	0.008179
CCND3	12.7237	18.06351	1.419674	0.067735
SNF8	50.39223	71.55053	1.419872	0.015081
HSD17B10	76.67135	108.9114	1.420497	0.048765

TJP2	11.23884	15.96492	1.420513	0.004163
UBE2C	171.3321	243.4034	1.420653	0.000394
PCDHGB2	0.198766	0.282396	1.420747	0.454615
PLXNA3	6.697606	9.517443	1.421022	0.014379
WBP2	43.82097	62.27778	1.421187	0.010007
BANF1	106.4678	151.329	1.421359	6.45E-06
TBC1D25	4.595601	6.532517	1.421472	0.002213
PKNOX1	4.610556	6.55428	1.421581	0.002442
ELK1	18.4658	26.25835	1.421999	0.014204
THEM6	7.429444	10.56885	1.422563	0.320052
STRIP2	2.029246	2.886819	1.422607	0.09151
TMEM64	2.282296	3.248065	1.423156	0.037601
ECH1	59.03364	84.04232	1.423635	0.020428
ZFAND2A	2.309453	3.287974	1.423703	0.284057
ACAT2	18.22912	25.95427	1.423781	0.115914
TEX10	10.76134	15.3229	1.423884	0.023775
PDK1	6.025135	8.579775	1.423997	0.25306
MFSD10	21.7653	31.01795	1.42511	0.003976
MUT	9.385255	13.3773	1.425352	0.000273
OIP5	7.839767	11.1799	1.42605	0.172272
MAP1S	10.3487	14.76671	1.426914	0.070943
PGLS	33.15766	47.31772	1.427052	0.014548
TANK	6.700426	9.56209	1.427087	0.021714
RFPL1	0.017124	0.024439	1.427131	0.745652
HCFC1R1	20.04339	28.60657	1.427232	0.17361
KLHDC1	0.075033	0.107108	1.427475	0.371959
TRAPPC12	3.456023	4.935073	1.427963	0.025808
MEX3D	6.347159	9.065976	1.428352	0.066154
MAST2	8.490403	12.12938	1.428599	0.016925
C9orf47	0.060577	0.086542	1.428645	0.615012
NDUFB9	178.8492	255.5227	1.428704	0.006482
KIF3C	6.106479	8.724972	1.428806	0.002173
RAB18	10.61236	15.16686	1.429169	0.043972
PML	4.846754	6.929073	1.429632	0.009208
HLA-DPA1	27.37975	39.14523	1.429715	0.270538
APEH	31.91052	45.62411	1.429751	0.041514
SOCS3	15.48998	22.14967	1.429935	0.11939
BBS12	1.591368	2.275645	1.429993	0.16202
ALKBH6	10.42036	14.90326	1.430206	0.048764
PIF1	3.086271	4.414289	1.430299	0.128665
MRPL33	102.7264	146.9302	1.430306	0.00302
BSG	361.2703	516.7803	1.430454	0.013726
SACS	1.894202	2.710604	1.431	0.036326
SNX1	10.73701	15.37497	1.431961	0.021619
AP5B1	4.53801	6.49994	1.432332	0.264815
RPUSD2	5.02488	7.201579	1.433184	0.003588
TRPM8	0.193091	0.276941	1.43425	0.290123
MEIG1	0.403817	0.579319	1.434606	0.490914
CFAP45	2.132674	3.059759	1.434706	0.347172
EDN2	0.044388	0.063687	1.434771	0.729552
TBC1D5	5.36426	7.697282	1.43492	0.094193
ZBTB14	1.609081	2.309067	1.435022	0.048988
GTPBP6	0.188511	0.270539	1.435136	0.433913
SYNE1	0.946039	1.357812	1.43526	0.120794
LOC11226	0.191338	0.274642	1.435381	0.380009
HIST1H2A	0.422595	0.606681	1.435608	0.491137
KCTD10	14.10017	20.24525	1.435816	0.001674
BAX	50.11236	71.97026	1.436178	0.001431

ATP5MD	98.31814	141.2621	1.436785	0.005476
SORD	11.86886	17.058	1.437206	0.150692
TTC30B	0.801802	1.152869	1.437848	0.362668
CYB5R3	80.1186	115.2005	1.437874	0.00127
SLC35G5	0.046471	0.06686	1.438743	0.81461
EIF5A	123.1421	177.2258	1.439198	0.009715
LEO1	18.66479	26.86354	1.439263	0.015655
WDR20	2.181742	3.140465	1.43943	0.009231
EPSTI1	0.159262	0.229262	1.43953	0.266806
MAX	16.33457	23.51553	1.439617	0.006166
LOC11226	0.069166	0.099591	1.439893	0.665565
MYRFL	0.008713	0.012546	1.439893	0.665565
ZNF79	1.877433	2.703552	1.440025	0.165541
NSF	10.33619	14.88762	1.440338	0.014067
NDUFB1	138.6585	199.7286	1.440436	0.00244
SLC27A5	3.387899	4.881383	1.440829	0.069111
DDR2	5.854762	8.436158	1.440905	0.075408
NELFB	33.2849	47.96741	1.441116	0.005356
UFSP1	0.742467	1.070047	1.441205	0.285109
SSR3	62.71345	90.38903	1.441302	0.003245
CCZ1B	9.119582	13.14419	1.441315	0.029836
NCBP2-AS	14.93009	21.52334	1.441608	0.014325
RUBCN	2.311211	3.334936	1.442939	0.0289
RNF182	3.746043	5.408165	1.443701	0.14291
CCT5	118.9568	171.7798	1.444052	0.029984
PRR11	10.49555	15.15631	1.444069	0.021583
TMEM150	1.756091	2.537331	1.444874	0.097087
PRPF31	37.84078	54.69931	1.445512	0.004981
DDX27	30.66265	44.349	1.446353	0.0093
ANKEF1	1.084645	1.569092	1.446641	0.111473
TMED10	75.00195	108.5666	1.447517	0.018605
SDF2L1	23.76683	34.40661	1.447673	0.021307
RANGRF	11.99996	17.37692	1.448081	0.006247
PLB1	0.078672	0.113959	1.448533	0.165461
CYB561D1	2.433784	3.526833	1.449115	0.013503
SLC8B1	3.443289	4.990056	1.449212	0.025955
WWTR1	8.097866	11.73606	1.449278	0.065213
CLN6	17.3752	25.18639	1.449559	0.085165
TGM4	0.017643	0.025581	1.449958	0.787323
ACBD5	2.144271	3.109575	1.450178	0.01151
SFPQ	25.75553	37.37532	1.451157	0.00927
TXNDC11	9.436297	13.69563	1.451378	0.012609
CSPG4	6.572278	9.541789	1.451824	0.32146
MYBPHL	0.013422	0.019497	1.452542	0.723057
ARL1	27.0901	39.35652	1.452801	0.005044
CEP19	3.561821	5.177296	1.453553	0.294914
CHPF	28.54667	41.5069	1.454002	0.078999
TCEAL4	47.2159	68.6553	1.454071	0.016476
PPP1R11	28.28918	41.13601	1.454125	0.001697
PAK3	0.135498	0.197053	1.454287	0.207431
SLC25A33	6.573353	9.560455	1.454426	0.024394
CANT1	15.9871	23.26229	1.455066	0.004733
NFE2L1	55.58607	80.89149	1.455247	0.011113
H2AFV	25.42207	36.99702	1.455311	0.000145
CABP1	0.652641	0.949797	1.455314	0.189037
TRIM16	8.275461	12.04634	1.45567	0.037811
IKZF2	0.098306	0.14314	1.456064	0.3584
UBE2D4	2.832679	4.12549	1.456392	0.173712

DLX1	3.475248	5.061917	1.456563	0.409989
HOXD8	3.986193	5.807735	1.456963	0.016149
PAPPA2	0.310807	0.453058	1.457682	0.41408
GMIP	2.206151	3.216438	1.457941	0.158334
AP4S1	0.716178	1.044615	1.458597	0.178706
RAP2B	4.499776	6.564359	1.458819	0.060949
HSBP1	86.598	126.3572	1.459124	0.002104
PACRGL	1.360476	1.985225	1.459213	0.082557
FAM172A	0.782713	1.142402	1.459541	0.010842
ATP5PD	247.7522	361.6048	1.459542	0.001687
HDAC7	5.801002	8.468575	1.459847	0.0073
DUSP14	10.06105	14.68978	1.460065	0.000132
MAGOHB	14.02267	20.48177	1.460619	0.157172
GPNMB	1.93964	2.833253	1.460711	0.091035
PARN	10.57874	15.45926	1.461352	0.053131
SNX18	2.551716	3.729402	1.461527	0.02441
RAD51AP2	0.008663	0.012662	1.461614	0.767945
DDX24	56.58145	82.71414	1.46186	0.002726
HIST1H1C	31.1981	45.60867	1.461905	0.436847
DAZAP2	39.28891	57.44454	1.462106	0.025279
CUL1	19.14012	27.98682	1.462207	0.130878
GNB2	104.1687	152.3232	1.462274	0.00667
TOM1L2	5.476791	8.008725	1.462302	0.006549
TUBB2A	12.80939	18.73194	1.46236	0.149374
PSMA5	42.19898	61.71809	1.462549	0.021621
RNF114	36.02497	52.68971	1.462589	0.000483
USP31	2.009232	2.938808	1.462652	0.023368
CCDC106	9.434842	13.80307	1.462989	0.0217
PSMD14	71.1566	104.1137	1.463163	0.065662
TTC8	6.280879	9.190377	1.463231	0.029524
CYB5B	25.12158	36.75965	1.46327	0.005546
MRPS7	37.61963	55.079	1.464103	0.030603
NUDT6	5.434831	7.95728	1.464126	0.266918
LNP1	1.481499	2.171405	1.465682	0.263225
STXBP1	16.96424	24.88221	1.466745	7.38E-05
TDP1	7.178809	10.53118	1.466981	0.019511
CYBB	0.013035	0.019125	1.467219	0.781191
MTFMT	4.831687	7.089211	1.467233	0.019002
PRKACB	2.26477	3.323317	1.467398	0.170032
ZNF440	1.633271	2.396821	1.467497	0.080065
ODF2	9.83993	14.44211	1.467704	0.000976
CREBL2	8.392599	12.32079	1.468054	0.002519
CPPED1	1.985836	2.915469	1.468132	0.029431
KCMF1	6.936784	10.18417	1.46814	0.020355
GDE1	26.45431	38.84555	1.468402	0.024241
TFIP11	6.246785	9.173635	1.468537	0.023688
KIF1BP	10.43629	15.32789	1.46871	0.007326
PLEKHA4	3.458702	5.080738	1.468972	0.044012
C1orf56	3.211165	4.717276	1.469023	0.223715
INTS11	18.3794	27.00473	1.469293	0.001396
ZNF436	2.009202	2.954284	1.470377	0.111203
VTN	0.045836	0.067402	1.47051	0.743402
LYRM1	14.42451	21.23187	1.47193	0.030574
SPNS3	0.020431	0.030075	1.472036	0.715809
TCEAL9	95.44253	140.6021	1.47316	0.000201
MICAL1	7.92663	11.67965	1.47347	0.05899
SASH1	2.272547	3.348884	1.473626	0.241555
PSMD6	45.627	67.25854	1.474095	0.020621

XRCC5	97.46416	143.6736	1.474118	0.023281
CATSPERD	0.032992	0.048689	1.475748	0.634117
GTF3C6	33.27337	49.10488	1.475801	0.147683
PSMB2	44.18699	65.22992	1.476225	0.011426
ATP1A1	90.97673	134.3548	1.476804	0.000446
KDELC1	11.5731	17.09444	1.477085	0.020497
LRRC2	0.050486	0.074578	1.477213	0.554226
ARL4D	11.8739	17.54069	1.477247	0.001198
PPP2CB	30.77153	45.46135	1.477384	0.000323
TAOK3	3.902433	5.765418	1.477391	0.015937
CAMTA1	2.371743	3.504215	1.477485	0.001824
CCNB2	52.80231	78.04155	1.477995	0.011443
CMTM3	10.7676	15.91883	1.478401	0.028564
SAP30L	4.551714	6.729611	1.478479	0.012516
MRPL13	47.38383	70.06122	1.478589	0.050067
APOA4	0.037931	0.056092	1.478784	0.497508
DSE	3.762552	5.564297	1.478862	0.009153
DCUN1D3	2.885816	4.267741	1.478868	0.190146
CBWD2	6.425527	9.502756	1.478907	0.010517
PTPN2	4.993755	7.386403	1.479128	0.000304
ANKMY2	7.157529	10.58815	1.479303	0.08321
AKIP1	9.284662	13.73665	1.479499	0.013057
BLOC1S6	11.41113	16.88592	1.479777	0.008463
MROH1	1.89793	2.808518	1.479779	0.017934
SNX9	16.61306	24.58905	1.480104	0.225234
NRDC	45.61056	67.51743	1.480302	0.01893
ZFP91	17.90384	26.52051	1.481275	0.047217
HDAC9	0.346393	0.513343	1.481969	0.074747
GPS2	53.24826	78.91687	1.482055	0.007664
SPEG	1.954848	2.897521	1.482223	0.038428
VTI1A	0.692045	1.026396	1.483134	0.004484
FOXH1	0.076718	0.113795	1.483285	0.522027
MRPL57	7.382456	10.95068	1.483339	0.052119
CD2AP	7.553832	11.20612	1.483501	0.018637
ZNF599	0.735212	1.090795	1.483647	0.254237
IGF1	0.00609	0.009037	1.483794	0.723112
ADA2	0.011543	0.017131	1.484111	0.657689
MUC20	0.118723	0.176367	1.485538	0.550293
TRAPPC5	53.79115	79.9327	1.485982	0.007932
CD81	107.3595	159.5896	1.486498	0.003295
ARF4	189.2848	281.3783	1.486534	0.083776
EXT2	24.07005	35.79665	1.487186	0.000659
RBM47	0.448632	0.668351	1.489755	0.197916
USP13	2.819615	4.203135	1.490677	0.044155
ASS1	99.59925	148.4885	1.490859	0.067914
BTN3A2	7.935077	11.83246	1.491158	0.123796
TAP2	4.973716	7.417499	1.491339	8.67E-05
NIPAL4	0.134999	0.201416	1.491988	0.053982
CTPS1	17.74004	26.48287	1.492831	0.131089
ZNF134	3.163187	4.724145	1.493476	0.044477
RNF135	3.567998	5.330662	1.49402	0.058256
OTOGL	0.092605	0.138382	1.494319	0.413706
CTDNEP1	70.37878	105.1998	1.494766	0.000467
TXNRD1	41.38614	61.87614	1.495093	0.186259
TMEM232	0.030076	0.045	1.496232	0.354385
ELOC	32.25253	48.2669	1.49653	0.002765
ST6GALNA	12.44837	18.63459	1.49695	0.048353
POMP	68.25462	102.1771	1.496999	0.008656

BFAR	18.71143	28.01398	1.497159	0.088828
CASP6	6.414765	9.609201	1.497982	0.002948
DNAJC10	16.22606	24.31546	1.498544	0.018424
NEURL1B	2.452926	3.67602	1.498627	0.238384
PPP1R7	21.16334	31.7205	1.498842	0.005373
SCOC	29.04904	43.53994	1.498843	0.020383
LTBP2	1.507622	2.259752	1.498885	0.105484
MTSS1	0.691272	1.036347	1.499189	0.039737
ELOVL7	2.55566	3.831598	1.49926	0.015574
MIDN	12.93931	19.40359	1.499584	0.020237
CASP8	3.713528	5.571205	1.500246	0.02733
NLRP14	0.075477	0.113252	1.500478	0.390448
ERG28	23.01736	34.53971	1.500594	0.012212
LRRC42	26.69781	40.0681	1.500801	0.000296
SP6	1.406971	2.111751	1.50092	0.000784
P2RX4	8.347885	12.53585	1.50168	0.008278
MRPL58	28.40735	42.66762	1.501992	0.019532
SLC35A2	10.78423	16.21056	1.503172	0.076236
FKBP8	144.0498	216.5574	1.503351	0.000234
PFDN4	30.22014	45.43652	1.503518	0.020023
PACSIN3	15.34824	23.07826	1.503642	0.033292
NUCB1	47.2175	71.01138	1.503921	0.006729
LOC11226	0.409376	0.615757	1.504135	0.404249
PTPRM	4.753541	7.1515	1.504457	0.007736
LMAN2L	11.71143	17.62209	1.504691	0.080594
ANXA7	15.58699	23.45554	1.504815	0.002091
PSMC2	35.61303	53.60983	1.505343	0.009357
MMAB	4.365062	6.572034	1.505599	0.203764
ADCK5	1.117806	1.683827	1.506368	0.075174
TMED9	148.2624	223.3971	1.506768	0.016626
ZNF358	24.37405	36.72989	1.506926	0.013006
MEMO1	8.358165	12.59727	1.507181	0.001804
TMEM115	22.56999	34.01834	1.507238	0.001605
RRP1	17.24785	26.00205	1.507553	0.01134
IMPAD1	17.07473	25.74816	1.507969	0.000655
SUPT4H1	37.29426	56.24334	1.508096	0.002171
ZDHHC14	0.632069	0.953385	1.508355	0.180705
VPS35L	10.90876	16.45443	1.508368	0.015124
TBC1D8	4.025583	6.072386	1.508449	0.012239
ARL2BP	27.97413	42.20003	1.508538	0.002988
AGBL4	0.007369	0.011121	1.50901	0.20202
AKR7A2	22.11057	33.39369	1.510304	0.024834
RBM24	0.544646	0.82259	1.510321	0.137577
SREBF1	7.160494	10.81742	1.510709	0.078354
CCDC184	1.200828	1.814247	1.51083	0.290886
DYNC1H1	34.98134	52.85221	1.510869	0.04713
WDR55	5.956633	9.001292	1.511138	0.028895
MTMR14	12.89361	19.49029	1.511625	0.000601
ST3GAL4	9.865567	14.91326	1.511647	0.030014
THAP10	2.636463	3.98606	1.511897	0.047636
FAM120A	9.16099	13.85064	1.511916	0.01042
UQCRQ	78.27788	118.3526	1.511955	0.001658
PHETA1	2.558623	3.868571	1.511974	0.14362
SH3YL1	4.67467	7.070186	1.512446	0.038732
HIST1H2B	2.081144	3.148201	1.512727	0.437757
CPEB4	1.886685	2.854289	1.51286	0.227261
ATP5F1D	85.38492	129.1788	1.5129	0.009613
MYOZ3	0.019517	0.029536	1.513338	0.622239

ETV6	1.245567	1.885923	1.514108	0.021894
FMN1	0.290718	0.44026	1.514392	0.1786
C21orf2	2.920137	4.423519	1.514833	0.005614
SERINC3	41.27659	62.53276	1.514969	0.024691
BLOC1S3	5.653153	8.564998	1.515084	0.025739
POLDIP2	38.45181	58.26023	1.515149	0.062191
CLDND2	0.352655	0.534415	1.515405	0.121037
TNFRSF1A	26.65844	40.4159	1.516064	0.006197
PEX14	3.534691	5.359939	1.516381	0.022077
FRMD6	2.712921	4.113947	1.516427	0.046816
PPP1R37	10.07244	15.28437	1.517444	0.001792
POLR2F	19.48459	29.56773	1.517493	0.022473
DNAJC14	9.544514	14.48669	1.517803	0.004958
CDK11B	9.785099	14.85313	1.517934	0.017825
DERA	13.92925	21.14391	1.51795	0.058738
MON1A	5.327388	8.089262	1.518429	0.020948
ARL6IP5	49.39887	75.05903	1.519448	0.012495
ATP2B1	11.21373	17.04018	1.519582	0.012694
XXYLT1	1.257948	1.912077	1.519996	0.063225
APOPT1	14.91058	22.66657	1.520167	0.005199
AGRN	15.5646	23.66183	1.520234	0.135031
WDR74	10.79416	16.41161	1.520416	0.013491
B4GALT2	28.61086	43.52186	1.521166	0.031112
ZSWIM4	2.540283	3.864694	1.521363	0.01879
CYB5A	15.87525	24.1614	1.521955	0.005378
TEX264	21.5497	32.8013	1.522123	0.010814
ARHGEF17	0.924633	1.408243	1.52303	0.028734
COL5A2	6.207927	9.456776	1.523339	0.12624
BECN1	27.08518	41.26593	1.523561	0.005892
TSG101	45.17596	68.84888	1.524016	0.01605
CRIPT	2.754615	4.201904	1.525405	0.007265
NACAD	0.56295	0.859057	1.525991	0.049489
UCP3	0.162891	0.248619	1.526296	0.258458
TRMT112	86.5517	132.1757	1.52713	0.000655
AMN	0.019815	0.030266	1.527398	0.537584
FER1L5	0.042216	0.064517	1.528265	0.28977
THOP1	18.95945	28.98982	1.529044	0.006943
CYP20A1	1.209528	1.850709	1.530108	0.007217
ARMC5	1.273697	1.950604	1.53145	0.101175
MLX	17.49135	26.80708	1.532591	0.025057
NCOA6	4.386334	6.722571	1.532617	0.035873
PDAP1	57.87989	88.7519	1.533381	0.015579
CYB5D1	2.63066	4.033991	1.533452	0.142429
DCAF13	28.00273	42.95123	1.533823	0.001998
P3H1	31.0369	47.61848	1.534254	0.049987
SAR1B	8.665508	13.29519	1.534265	0.005405
RPN1	192.8894	295.9468	1.534282	0.001718
EXOSC3	18.5264	28.4285	1.534486	0.03425
OLFML3	0.627494	0.963039	1.534739	0.213598
SNX8	4.343105	6.66596	1.534837	0.038834
TEN1	6.451403	9.902697	1.534968	0.114519
CYFIP1	12.44326	19.10182	1.535114	0.000303
ARL4A	3.528652	5.417102	1.535176	0.069641
SCAF1	19.2725	29.5917	1.535437	0.011821
ANKRD16	2.178764	3.345451	1.535481	0.165225
ATG12	13.31172	20.44867	1.53614	0.002052
ABTB2	3.590626	5.515788	1.536163	0.099608
TP53I13	17.65177	27.12366	1.536597	0.001202

ADH5	44.72069	68.72685	1.536802	0.01744
NAV1	2.885604	4.434708	1.536839	0.02258
ZNF625	0.33255	0.511174	1.537137	0.162764
TM9SF1	16.99999	26.13371	1.537278	0.022825
MLF2	92.84338	142.7471	1.537504	0.019703
CLTC	36.32754	55.86908	1.537926	0.000399
ITGA7	6.540368	10.06563	1.539	0.099767
PDF	1.777368	2.735681	1.539176	0.275697
DCTN3	81.22917	125.0379	1.539322	0.005482
TBX3	10.296	15.84933	1.539367	0.054054
PARP4	7.098026	10.93286	1.540268	0.00903
GCGR	0.181304	0.279304	1.540529	0.175378
EIF5A2	1.880217	2.897409	1.540997	0.022814
PBXIP1	11.67612	17.99304	1.541012	0.006193
ABL1	14.80003	22.8118	1.541335	0.008902
NDUFA5	11.41952	17.61476	1.542514	0.00109
GCAT	8.293197	12.80123	1.543582	0.073754
ZDHHC16	16.51804	25.50136	1.54385	0.013564
STIP1	110.8481	171.1868	1.544337	0.001657
EXOSC4	14.5087	22.41731	1.545094	0.043527
C19orf53	96.45902	149.0444	1.545157	0.004788
E2F4	32.26399	49.86142	1.54542	0.004073
FURIN	18.5144	28.61991	1.545819	0.075344
UQCC3	9.546911	14.75892	1.545936	0.017206
GMFB	16.06242	24.83706	1.546284	0.005401
TSKU	3.603357	5.573159	1.546658	0.05081
CIZ1	24.04713	37.19469	1.546741	0.007508
IL13RA1	27.22905	42.12517	1.547067	0.012936
TFAP2C	6.354303	9.83068	1.54709	0.053506
SSBP1	85.9735	133.009	1.547093	0.083928
WLS	62.52049	96.75427	1.547561	0.02652
LOC10798	0.272163	0.421256	1.547807	0.215711
PRKAA1	4.882406	7.558513	1.548112	0.004786
COQ4	11.86267	18.3656	1.548184	0.005697
TRIM32	5.756559	8.913938	1.548484	0.025547
PHB	82.67615	128.0542	1.548865	0.034033
OGG1	3.670353	5.685806	1.549117	0.010119
FBH1	8.887064	13.77099	1.549554	0.000438
NPR1	0.017233	0.026714	1.550117	0.657425
DVL1	27.59536	42.77868	1.550213	0.004502
DCN	2.25576	3.499214	1.551236	0.29357
SPATA5L1	4.170229	6.472623	1.552102	0.026532
POLR2E	48.76603	75.69089	1.552123	2.17E-05
BCKDK	22.05312	34.23058	1.552188	0.065506
TIMM22	12.8833	20.00028	1.552419	0.018534
TMEM59	22.7087	35.26694	1.553015	0.03293
DNAJB2	10.49124	16.30017	1.553693	0.020423
MSRB3	3.611157	5.610678	1.553707	0.034117
SGO2	5.441938	8.458852	1.554382	0.011802
CCDC71L	2.809406	4.369033	1.555145	0.090853
NACC2	2.467785	3.83955	1.555869	0.006255
RAVER1	18.82851	29.30259	1.556288	0.000117
C2orf81	1.242161	1.933205	1.556324	0.140916
MED16	14.10264	21.96068	1.557203	0.002901
CCDC34	13.26839	20.66289	1.557302	0.014353
ZDHHC5	15.7268	24.49355	1.557441	0.010157
SCPEP1	24.09682	37.56048	1.558732	0.004136
MAPKBP1	1.955861	3.04892	1.558863	0.003876

YKT6	51.87189	80.86238	1.558886	0.011947
TBL1XR1	7.319577	11.4119	1.559093	0.003939
FAM83G	3.737477	5.828452	1.559462	0.191993
LOC10106	0.264361	0.412682	1.561056	0.350748
XRCC4	4.707623	7.349905	1.561277	0.017984
FZD6	4.956756	7.739792	1.561463	0.016182
ZFPM1	1.098542	1.715369	1.561496	0.045944
TUBA1A	72.05915	112.6547	1.563364	0.160808
TMEM43	31.39856	49.09314	1.563547	0.005974
SAC3D1	14.50231	22.67775	1.563734	0.018348
SPECC1	2.936943	4.594927	1.564527	0.028604
NPHP1	2.755889	4.311938	1.564627	0.028331
IFT57	23.82356	37.29067	1.565286	0.04625
ANKRD12	1.249127	1.955862	1.565783	0.05081
PRH1	0.275773	0.432024	1.56659	0.301104
B3GALT1	0.010069	0.01578	1.567178	0.560946
PTP4A2	57.72522	90.47318	1.567308	0.000859
THAP7	21.22058	33.26054	1.567372	0.031087
FAM98A	24.83487	38.93272	1.567664	0.002467
AP2S1	150.1473	235.4176	1.567911	0.002584
TGOLN2	30.85271	48.38089	1.568125	0.003006
BMT2	4.202551	6.590895	1.568308	0.005415
TP53BP1	5.628711	8.827588	1.568314	0.00091
TNFRSF8	0.168823	0.264775	1.56836	0.216886
SPRED2	6.018077	9.442426	1.56901	0.022948
UBTD1	8.686896	13.64	1.570181	0.003845
NOP16	14.25725	22.38662	1.570192	0.027935
SORBS3	10.54111	16.56172	1.571156	0.00267
CENPN	11.05314	17.36852	1.571366	0.012728
MFSD12	29.15321	45.81847	1.571644	0.040143
TCTN2	8.201783	12.89071	1.571696	0.006903
CTNNBL1	30.92064	48.61671	1.572306	0.00141
FARS2	2.09386	3.292422	1.572418	0.022343
SIPA1L1	2.56585	4.034917	1.572546	0.002769
CYR61	27.12756	42.66256	1.572665	0.386237
PHYHIP	0.149384	0.234969	1.572925	0.135659
EIF4G3	11.43066	17.98865	1.57372	0.001345
MYO10	3.058447	4.813827	1.573945	0.003029
SRFBP1	2.899829	4.56541	1.574372	0.072269
M6PR	21.98369	34.62217	1.574903	0.008765
CUL2	8.077062	12.73102	1.576194	0.008881
YIF1A	75.03164	118.2674	1.576234	0.002236
TUBGCP5	4.649365	7.333413	1.577293	0.010927
SDCBP	40.02136	63.14231	1.577715	0.009578
ZDHHC2	3.086016	4.869225	1.577835	0.004557
TTLL5	7.143664	11.27228	1.577941	0.014461
FAM98C	6.181158	9.754351	1.578078	0.056003
TECPR2	1.830872	2.890204	1.578594	0.028769
NDUFA2	80.69292	127.4194	1.579066	7.08E-05
KCNMB3	0.079305	0.125243	1.579257	0.185619
EFCAB11	2.511971	3.967109	1.579281	0.005883
GPATCH3	5.016151	7.922625	1.579423	0.013613
AATF	31.90913	50.41248	1.579876	0.010084
TMED1	13.4632	21.27791	1.580449	0.072839
MFN2	20.39239	32.23002	1.580493	0.003095
PIGV	4.284068	6.77204	1.58075	0.037408
RBFA	4.471895	7.069865	1.580955	0.006607
ATP6V1E1	91.05776	143.9693	1.581076	0.038663

GNPDA1	13.34441	21.09975	1.581168	0.004284
SLC25A22	10.28257	16.28606	1.583851	0.002153
IL1RN	1.318984	2.089118	1.583884	0.557435
CHIC2	1.164439	1.844408	1.583945	0.019765
SSNA1	58.18125	92.16216	1.584053	0.001107
KRT10	21.84608	34.61028	1.584279	0.012792
NFKBID	1.173232	1.858944	1.584464	0.187224
AAK1	1.866985	2.960105	1.5855	0.000666
FMO4	0.78513	1.244851	1.585535	0.10504
SLC30A7	1.924995	3.053012	1.585984	0.011814
SMKR1	1.475328	2.339971	1.586069	0.133324
MPC2	32.08279	50.88701	1.586116	0.066953
NMT2	6.478948	10.27682	1.586186	0.020833
ST7	5.714001	9.064598	1.586384	0.003545
EFR3A	3.757374	5.96144	1.586598	0.002607
FSCN1	54.37778	86.27973	1.586673	0.05787
TIMM10B	7.749108	12.2959	1.586751	0.00668
RILP	2.473147	3.924288	1.586759	0.146061
SPNS1	11.77385	18.69047	1.587457	0.010141
NFIL3	14.50468	23.03318	1.587983	0.026314
URGCP	6.649985	10.56314	1.588446	0.010214
RWDD4	5.75936	9.150633	1.588828	0.007676
PPIF	35.63976	56.64323	1.589327	0.006115
LMF2	23.68133	37.65713	1.590162	0.000512
UBE2J1	14.65181	23.30308	1.590458	0.000553
DERL1	17.95287	28.55462	1.590532	0.000102
TBC1D3	0.058823	0.093588	1.591008	0.610718
DPCD	13.58278	21.62537	1.592116	0.053316
TP53RK	5.966838	9.503139	1.592659	0.001374
KCTD9	7.846164	12.50226	1.593424	0.000927
GCLM	3.452794	5.503216	1.593845	0.011176
MFSD14B	15.32083	24.42341	1.594131	0.026625
USE1	18.28449	29.15152	1.59433	0.000428
MACO1	6.974222	11.1225	1.594801	0.020301
KIF13B	2.730615	4.356512	1.595432	0.017618
ISCA1	18.56096	29.62268	1.595967	0.004062
MAP7D1	48.3051	77.10265	1.59616	0.003656
ZNF23	4.762159	7.60144	1.596217	0.064501
VAV2	5.456368	8.710026	1.596305	0.003829
ANAPC15	7.596396	12.13212	1.597089	0.048427
OAZ1	603.3133	963.6636	1.597285	0.00727
COX8A	372.6909	595.4418	1.597683	0.000498
TSEN34	19.56778	31.27407	1.598243	0.003739
NETO2	6.763932	10.81206	1.598487	0.017408
FAT1	4.480998	7.164273	1.598812	0.003486
EPN2	10.04014	16.0625	1.599828	0.000515
ACOT2	4.611413	7.377577	1.599852	0.168699
NDUFB3	30.49047	48.80589	1.600693	0.007314
SORBS2	0.477883	0.765175	1.601177	0.017794
RPN2	206.4051	330.5104	1.601271	0.00143
TUSC1	4.613975	7.395383	1.602823	0.026339
ENKD1	8.472592	13.58136	1.602976	0.004468
ALG2	16.00543	25.65738	1.603042	0.023484
SART1	24.55376	39.3634	1.603152	0.012578
EDF1	239.9445	384.9783	1.604448	0.00112
TMEM17	0.426336	0.684112	1.604633	0.083103
EBP	51.46447	82.6225	1.605428	0.009535
PCID2	3.899027	6.260544	1.605668	0.001163

CCDC116	0.106542	0.171076	1.605711	0.109998
SEC61G	154.0811	247.4536	1.605995	0.015266
ACOT9	11.05206	17.76004	1.606944	0.004815
CCDC61	1.606676	2.582254	1.607203	0.159366
PTTG1IP	43.27222	69.55734	1.607436	0.001251
MTF1	1.953756	3.141081	1.607714	0.006083
CFLAR	1.540094	2.477593	1.608728	0.060171
TOE1	4.931529	7.9366	1.609359	0.007118
DPY19L1	7.847911	12.63159	1.609549	0.002187
STUB1	42.9087	69.07168	1.609736	0.01204
ABHD6	6.247206	10.06156	1.610569	0.029384
TMEM185	3.404438	5.485895	1.611395	0.009036
PTPRG	2.774877	4.471818	1.611538	0.003098
SCFD2	1.745359	2.813132	1.611778	0.045385
STMP1	38.15121	61.50832	1.612224	0.000296
CHID1	15.4205	24.86155	1.61224	0.00073
OGFOD3	5.428044	8.759362	1.613723	0.000191
ALYREF	78.90888	127.4222	1.614802	0.002817
RNPEPL1	10.76962	17.39666	1.615346	0.016784
TRMT6	6.787987	10.96595	1.615494	0.113273
FRMD8	4.87626	7.878055	1.615594	0.003693
C10orf25	0.263488	0.425715	1.615691	0.181839
INIP	5.147133	8.316406	1.615736	0.00571
RWDD2B	10.6978	17.29212	1.616419	0.121246
DNAJB12	6.924156	11.19691	1.617079	0.01895
ALKBH7	13.64521	22.06583	1.617112	0.021709
GOLGA5	15.81879	25.58703	1.617509	0.00231
HIST1H1E	0.568662	0.920205	1.618192	0.20904
TMEM19	2.884415	4.668252	1.61844	0.067969
CCNO	2.547406	4.123344	1.618644	0.052716
STKLD1	0.156761	0.253744	1.618671	0.089133
RNF4	13.62253	22.05105	1.61872	0.001426
DIRC2	3.386222	5.482167	1.618963	0.026321
GLB1	31.14252	50.4193	1.618986	0.000234
SEC62	13.23209	21.43972	1.620282	0.001414
KCTD17	11.35566	18.42377	1.62243	1.44E-05
FAM210B	12.88852	20.9269	1.623686	0.01575
PARL	18.18475	29.53381	1.624098	0.002663
LIMS1	9.090773	14.7682	1.624526	0.001467
IRF9	5.681499	9.230494	1.624658	0.013745
ZNF528	0.697149	1.132775	1.624869	0.18131
KLHDC7B	0.087576	0.14232	1.625105	0.516554
NDUFAF8	32.88526	53.47238	1.626029	0.037555
CLSTN1	30.89622	50.26291	1.62683	0.001378
UXT	77.521	126.1225	1.626946	0.001186
PDCL3	8.863883	14.42285	1.627148	0.005266
PSMA3	111.4724	181.418	1.627471	0.007358
UTP15	3.875514	6.308181	1.627702	0.033076
MAP2K1	20.27438	33.00625	1.627978	0.002328
GPC6	2.117358	3.449724	1.629259	0.017113
ACOT1	1.023578	1.668367	1.629937	0.033087
INPP5K	4.366811	7.122049	1.63095	0.009277
SULT1A4	0.202529	0.330374	1.631247	0.426562
PPP2CA	45.59492	74.37997	1.631321	0.005663
ATP5MC1	113.5185	185.2607	1.631987	0.010903
LOC10537	0.297536	0.485597	1.63206	0.270663
GRPEL1	16.56643	27.03891	1.632151	0.061359
CSTF1	16.33991	26.67573	1.632551	0.007576

ENOX2	3.393928	5.543653	1.633403	0.040505
MTRF1L	4.980764	8.137309	1.633747	0.000221
IPO13	8.635492	14.11114	1.634086	0.006178
AKAP12	15.51954	25.36182	1.634186	0.005385
TP63	0.030103	0.049199	1.634357	0.548108
GPR108	24.02435	39.27673	1.634871	0.012776
OAS1	0.131955	0.2158	1.635404	0.076307
MRPL46	28.34364	46.35749	1.635551	0.000322
ZFYVE19	8.76958	14.34368	1.635618	0.00426
FAM173B	4.4594	7.294802	1.635826	0.028428
AMMECR1	2.160436	3.534187	1.635868	0.107614
ZFP69B	1.25546	2.053973	1.636033	0.042916
PIK3CB	4.095696	6.700788	1.636056	0.000418
MPG	20.03804	32.78523	1.63615	0.01639
EFCAB5	0.039471	0.064586	1.636312	0.414362
EEF1AKM1	1.961029	3.209902	1.636846	0.126653
CTNNAL1	59.73403	97.8395	1.637919	0.005727
GLG1	21.53196	35.26824	1.637949	0.002486
PI4K2B	6.322251	10.35938	1.638559	0.034297
EXOC3	12.70923	20.82664	1.638702	1.55E-05
CBWD5	3.90089	6.394332	1.639198	0.018519
UTP3	17.73587	29.07445	1.639302	0.004608
NDUFS4	53.19775	87.2261	1.639658	0.024694
CDK7	17.5872	28.84021	1.639841	0.034114
SPSB2	2.584101	4.238935	1.640391	0.045046
SLC27A1	4.755777	7.803045	1.640751	0.055215
SH3GLB2	17.21142	28.24157	1.640862	0.002516
COPG1	69.70368	114.388	1.641061	0.009843
FLNC	22.21803	36.47709	1.641779	0.167958
VAPA	14.78591	24.28275	1.64229	0.001249
SIAH2	10.7528	17.66639	1.642958	0.014919
ECHS1	65.60905	107.797	1.643021	0.001084
CRTAP	46.70988	76.7501	1.643124	0.000204
MRPL27	49.75111	81.74962	1.643172	0.005275
DDX28	3.453979	5.676673	1.643517	0.147324
SCRN2	11.91781	19.59038	1.643791	0.001334
UBE2Z	29.63761	48.72642	1.644074	0.000589
C18orf32	0.248615	0.408817	1.644381	0.125939
MRPS36	20.76198	34.15557	1.645102	0.023739
VKORC1	161.0124	264.9239	1.645363	0.019536
EPB41L1	4.458577	7.336866	1.645562	0.018551
SGMS1	1.269401	2.089093	1.645732	0.00385
RORA	0.706783	1.163318	1.645934	0.057408
DLK1	0.002361	0.003888	1.646449	0.754048
LIPK	0.006227	0.010252	1.646449	0.754048
PLA2G4E	0.003449	0.005678	1.646449	0.754048
MS4A7	0.006483	0.010674	1.646449	0.754048
LOC11226	0.013777	0.022683	1.646449	0.754048
LGALS2	0.036948	0.060832	1.646449	0.754048
UROC1	0.005663	0.009323	1.646449	0.754048
LOC64665	0.004219	0.006946	1.646449	0.754048
HIPK2	3.64157	5.997735	1.647019	0.033243
NRBF2	12.34013	20.32825	1.647329	0.007973
SRR	4.724379	7.788527	1.648582	0.143239
RAB1B	108.5014	178.9377	1.649174	0.001548
NOC2L	46.05327	75.95752	1.64934	0.010872
NUDT16L1	1.933894	3.190846	1.649959	0.005154
STX4	22.97685	37.9119	1.650004	0.000196

CHST11	4.138172	6.82853	1.650132	0.010903
ZFYVE21	12.3277	20.34371	1.650244	0.014872
CUTA	58.39557	96.37785	1.650431	0.000511
GOLIM4	8.492947	14.02548	1.651427	0.017387
INO80	3.348454	5.532609	1.652288	0.007317
PNLIPRP3	0.148747	0.245838	1.652722	0.270808
ACTR5	4.783446	7.906543	1.652897	0.0042
PIFO	0.341373	0.564337	1.653138	0.201281
TUBGCP2	22.99029	38.0152	1.653533	0.001269
RPTOR	6.125057	10.13037	1.653922	0.017741
NDUFB5	48.58758	80.36148	1.653951	0.004006
MLLT1	15.18002	25.10749	1.653983	0.017913
EMC10	13.40127	22.16666	1.654071	0.030872
CERK	17.47589	28.92699	1.655251	0.012309
SELENOS	26.76059	44.30187	1.655489	0.002468
CD74	103.4115	171.3286	1.656766	0.291427
LIMA1	17.31913	28.69438	1.656803	0.016336
OCEL1	10.53436	17.47441	1.658801	0.016562
CHAMP1	6.244563	10.36058	1.659136	0.003121
SLC26A4	0.079171	0.131386	1.659531	0.242328
TOMM34	34.11743	56.62746	1.659781	0.008057
SLTM	6.961503	11.5627	1.660949	0.017537
RIPOR1	8.037663	13.35213	1.661196	0.000523
MAML2	0.764871	1.270648	1.661257	0.033569
EIF4A1	444.6742	738.9761	1.661837	0.020538
ITPK1	6.039516	10.03717	1.661916	0.017784
ALG3	24.40435	40.59075	1.663259	0.010305
SEZ6L2	20.89203	34.76214	1.663895	0.007669
TMEM151	0.040146	0.066801	1.663972	0.549397
HMGCL	19.22923	32.00353	1.664317	0.00875
PGPEP1	4.100553	6.827228	1.664953	0.011955
SERPINE2	80.48273	134.0039	1.665002	0.048528
CITED2	23.024	38.34179	1.665297	0.131671
TIMM23	53.47436	89.12402	1.666668	0.014977
UBE2L3	39.34559	65.57645	1.666678	0.000478
DCUN1D1	2.35846	3.932215	1.667281	0.020569
GGT1	2.940253	4.903139	1.667591	0.139894
LZIC	6.050584	10.09283	1.668076	0.004614
MED18	9.518936	15.87958	1.66821	0.005189
ZNF432	1.045346	1.744401	1.66873	0.027847
NOL9	2.750906	4.590721	1.668803	0.011795
ARHGAP1	3.917656	6.538402	1.668958	0.002718
ANO2	0.031953	0.053345	1.66947	0.236273
LAD1	0.006872	0.011474	1.669854	0.634261
IQSEC2	1.436896	2.399557	1.669958	0.004306
FAM126A	6.347579	10.6082	1.67122	0.002678
PNMA1	24.5426	41.02745	1.671683	0.00021
CSNK1A1L	0.023959	0.040066	1.672293	0.723704
PAK2	9.464243	15.82897	1.672503	4.74E-05
ZNF628	0.870039	1.455309	1.672693	0.00746
IL17RA	2.614248	4.372968	1.672744	0.000108
HIF3A	0.018196	0.030442	1.672981	0.415253
UQCRFS1	62.80252	105.1494	1.674287	0.00642
CDR2L	9.714357	16.26694	1.674526	0.003071
NOL12	8.033138	13.45365	1.674769	0.011423
AP5M1	2.168351	3.631853	1.674938	0.02029
CAPZA1	71.14543	119.1886	1.675281	0.001912
UQCC1	25.95358	43.48323	1.675424	0.004701

C3orf58	4.089734	6.852932	1.675643	0.027723
KCNN2	0.158489	0.265609	1.675878	0.256515
MON1B	6.623531	11.10077	1.675959	0.005408
ATP6V1C1	7.683413	12.88049	1.676402	0.054578
HMGA2	13.4132	22.51035	1.678224	0.003805
ZDHHC1	2.498984	4.194534	1.678496	0.019737
ARPC3	232.9577	391.0189	1.678497	4.34E-05
IFI44L	0.277316	0.465527	1.678684	0.085425
TBC1D13	7.5611	12.69679	1.679225	0.005272
SLC12A9	3.678091	6.180042	1.680231	0.025196
FKBP9	14.8259	24.92137	1.680935	0.005681
ZNF25	1.639273	2.755981	1.681222	0.010875
TMPRSS7	0.006148	0.010338	1.681471	0.629576
MAP3K8	0.670849	1.12828	1.68187	0.162102
MAOA	2.039795	3.432163	1.682602	0.002646
PRKG1	1.251809	2.106623	1.682863	0.00688
TUBA1B	874.4706	1472.022	1.683329	0.010342
HEXIM2	1.902609	3.202767	1.683355	0.006659
NRIP1	2.803775	4.719944	1.683425	0.001259
MAN1C1	0.808814	1.361689	1.683562	0.014982
STK40	23.78489	40.05484	1.684045	0.001149
DPM2	38.01791	64.02977	1.6842	0.000997
RNASEK	150.2715	253.2104	1.68502	0.02603
ABHD11	3.145592	5.300545	1.685071	0.007614
ARNTL2	3.778983	6.368378	1.68521	0.066392
ITGA10	0.16471	0.277906	1.687245	0.202081
SMIM14	4.107517	6.932571	1.687777	0.002183
NF2	6.017381	10.15796	1.688103	0.00035
HSPE1	16.25295	27.45005	1.688928	0.008964
TRIP6	34.89344	58.93882	1.689109	0.001123
INF2	13.31156	22.49608	1.689966	0.004161
TBC1D9B	23.85334	40.32303	1.690456	0.010598
FYN	23.37363	39.56521	1.692728	0.001912
GHRL	0.00712	0.012056	1.693258	0.742229
GGTLC3	0.036739	0.062208	1.693258	0.742229
C2CD4D	0.008277	0.014015	1.693258	0.742229
PIGO	3.601995	6.099646	1.693408	0.010634
ELOF1	40.13422	67.9663	1.693475	0.00122
SYPL1	21.21348	35.93833	1.694127	0.000379
CKAP2L	7.971099	13.50649	1.694432	0.003645
MRC1	0.011058	0.018738	1.694488	0.573413
F8A1	2.608815	4.420978	1.694631	0.025076
PHLDA3	11.93593	20.23729	1.695494	0.022661
AMPD2	13.9507	23.66231	1.696138	0.059
STAC2	3.314061	5.621857	1.696365	0.005061
BRK1	181.552	308.0761	1.696903	0.001983
GALK2	2.503025	4.247992	1.697143	0.001055
FEZ2	22.84022	38.77826	1.697806	0.006435
LMAN2	107.8609	183.2293	1.698755	0.000491
GJA9	0.033606	0.057099	1.699067	0.665563
MRPL34	29.20185	49.63756	1.699809	0.003752
PDCD10	16.47207	28.01108	1.70052	0.009923
VDR	1.8943	3.221584	1.700673	0.322652
TECR	52.58311	89.43913	1.70091	0.000145
TCF25	17.93507	30.53624	1.7026	0.000222
NFKB1	4.368856	7.439412	1.702829	0.009072
LYRM2	7.222055	12.30258	1.703473	0.015115
GNAI3	27.58729	47.00866	1.703997	0.001522

RRBP1	28.96848	49.37021	1.704274	0.002694
PCDHGA6	0.054002	0.092043	1.704421	0.43473
GAGE12B	0.041953	0.071525	1.704876	0.620117
PABPC4	41.90806	71.50346	1.706198	0.003349
WASHC5	6.012168	10.26559	1.707469	0.015281
CEP41	2.529877	4.320216	1.707678	0.016004
LOC10798	3.032358	5.179013	1.707916	0.004156
EFCAB13	0.347227	0.593107	1.708125	0.083096
MYCBP	13.31333	22.74149	1.708174	0.009643
TAF9	40.58169	69.32135	1.708193	0.013151
PCDHGB7	0.153517	0.262321	1.708738	0.215944
RPGRIP1L	1.216684	2.079642	1.70927	0.007515
RBM42	48.08609	82.19596	1.70935	0.005838
CNPPD1	11.85105	20.25972	1.70953	0.001756
GPN1	21.53697	36.82463	1.709833	0.005661
LSM14B	18.9783	32.45276	1.709993	0.000895
OR2B6	0.141126	0.24134	1.710107	0.556029
IFNAR1	5.408206	9.252477	1.710822	0.002633
PBDC1	20.73758	35.51777	1.712725	0.00264
PDXK	9.858448	16.8896	1.713211	0.001498
SMYD2	12.8069	21.94099	1.713216	0.007669
HK2	16.17654	27.72649	1.713994	0.016816
ALAD	9.562758	16.39052	1.713995	0.004407
DLX3	0.559796	0.959523	1.714058	0.550246
AGPAT3	4.423761	7.58305	1.714163	0.01703
GYG1	15.85363	27.18108	1.714502	0.002795
TSC22D1	10.25677	17.59157	1.715117	0.000492
TGIF1	5.250572	9.010615	1.716121	0.028769
DEFB1	0.042135	0.072324	1.716493	0.736584
SLC12A3	0.003504	0.006015	1.716493	0.736584
PSMD3	53.15318	91.25727	1.716873	0.009849
SH3GL1	50.41082	86.59822	1.71785	0.003407
PNO1	6.970873	11.97635	1.718056	0.006799
HSPH1	13.2591	22.78303	1.718293	0.061754
GPANK1	5.189615	8.917358	1.718308	0.023919
VPS37A	2.512718	4.31819	1.718533	0.001807
QDPR	15.03096	25.83918	1.719064	0.049678
CD40	1.555629	2.675073	1.719609	0.003908
CEP89	4.484608	7.711815	1.719618	0.014962
UTP20	2.711208	4.664462	1.720437	0.04302
HYPK	39.94216	68.72347	1.720575	0.007681
CCDC115	7.116186	12.24655	1.720943	0.002676
CBWD3	1.36195	2.344581	1.721489	0.00143
PTTG1	109.9435	189.3632	1.722369	0.000218
SEC61B	200.3856	345.1482	1.72242	0.000116
MYL4	0.046527	0.080168	1.723053	0.55837
DLC1	3.923198	6.760224	1.723141	0.005954
PPIA	354.9839	611.6875	1.723141	0.006859
WBP1L	3.838738	6.614733	1.723153	4.24E-05
TTPA	0.126083	0.21734	1.723781	0.068885
LOC11226	0.536581	0.925505	1.724819	0.154328
PHF19	4.63989	8.004136	1.72507	3.88E-05
TXNDC12	40.83058	70.43959	1.725167	0.002186
RNF181	23.14448	39.94244	1.725787	5.00E-05
SAG	0.005405	0.009332	1.726492	0.734198
ACCSL	0.007276	0.012561	1.726492	0.734198
SOST	0.008089	0.013965	1.726492	0.734198
TFAP2B	0.002664	0.004599	1.726492	0.734198

USP6	0.001403	0.002423	1.726492	0.734198
ZNF35	3.503448	6.048963	1.726575	0.060378
ZER1	6.477586	11.18815	1.72721	0.007575
GPATCH4	14.21048	24.55445	1.727911	0.084428
NSG2	0.061435	0.106187	1.728438	0.578691
PTGER3	0.018831	0.032549	1.728472	0.497963
CFAP54	0.060715	0.104951	1.728586	0.220822
SRM	61.61594	106.5113	1.728632	0.004374
TCEA2	5.683262	9.825758	1.728894	0.027518
RMC1	9.115073	15.75996	1.729	0.026832
PSMD7	87.74132	151.7517	1.729535	0.00025
EIF4G1	55.40707	95.83484	1.72965	0.004124
EFCAB14	10.11561	17.49792	1.729794	0.01218
SYF2	24.35406	42.1409	1.730344	0.0185
GSTK1	46.08571	79.75378	1.730553	0.005426
STK10	5.653531	9.787308	1.731185	0.000425
TPST2	5.85107	10.13673	1.732457	0.018979
COL8A2	0.042901	0.074333	1.732666	0.467527
LOC11226	0.088697	0.153775	1.733705	0.20284
LOC10798	0.031603	0.054804	1.734138	0.494856
EIF2AK4	7.824315	13.57517	1.734998	0.004462
MRPS11	7.025204	12.19096	1.735318	0.007889
SNUPN	12.32283	21.38607	1.735483	0.038907
CNPY2	29.95863	51.99316	1.735499	0.046856
EXOC7	8.168662	14.17933	1.73582	0.009206
SPOUT1	5.577784	9.68206	1.735826	0.00264
IFT22	9.08701	15.7754	1.736039	0.026294
SELE	0.009546	0.016578	1.736566	0.704445
PEX16	7.33235	12.73561	1.736907	0.009509
ZNF214	0.376934	0.654908	1.73746	0.081926
ZBTB17	5.519335	9.592548	1.73799	0.0015
PKP1	0.010539	0.018318	1.738147	0.520773
TOM1	13.5015	23.47465	1.73867	0.118399
NABP2	29.77732	51.78236	1.738987	0.01427
AGBL1	0.002246	0.003907	1.739387	0.585988
SCO2	8.834749	15.3677	1.739461	0.003693
SRI	33.29921	57.93013	1.739685	0.011889
CD164	45.49839	79.17568	1.740186	0.005685
EMC6	34.96871	60.85234	1.740194	0.004087
XIRP1	0.00743	0.01294	1.741647	0.710769
PDIA4	106.9902	186.4275	1.742473	0.000302
LCN1	0.032242	0.056194	1.742853	0.626648
PSMG2	33.34763	58.12277	1.742936	0.013342
PITRM1	17.34572	30.24792	1.743826	0.0069
ACTL6A	27.22282	47.49561	1.744699	0.000649
PTPRJ	4.037173	7.045788	1.745228	0.126515
MIGA2	3.253141	5.677576	1.74526	0.053962
CCL3L3	0.071829	0.125377	1.745484	0.632314
IRAK1	49.57417	86.53517	1.74557	0.00118
GGH	31.05547	54.21881	1.74587	0.005247
PTMS	178.3119	311.3369	1.746024	0.005098
PTPRK	5.225543	9.123944	1.746028	0.00057
FCF1	17.39591	30.37932	1.746349	0.00154
REXO2	36.2066	63.2368	1.746554	0.000485
CNTN3	0.001931	0.003373	1.746758	0.729441
MMRN1	0.003529	0.006165	1.746758	0.729441
SHE	0.001738	0.003037	1.746758	0.729441
SVOP	0.005724	0.009998	1.746758	0.729441

STOML3	0.009203	0.016076	1.746758	0.729441
FAM237A	0.016477	0.028781	1.746758	0.729441
HIST1H2B	0.042271	0.073838	1.746758	0.729441
C12orf54	0.009542	0.016668	1.746758	0.729441
SPTA1	0.002217	0.003872	1.746758	0.729441
CCR4	0.008735	0.015259	1.746758	0.729441
CYB561	3.516971	6.143558	1.746832	0.000755
ST3GAL2	5.720246	9.996728	1.747605	0.001117
PHYH	13.01535	22.75303	1.748168	0.017066
C5orf60	0.014355	0.025105	1.748909	0.581563
PEX12	1.895858	3.31572	1.748928	0.088096
FLNB	32.46128	56.78923	1.749445	0.002524
ACTN3	0.022275	0.038981	1.74993	0.600567
STYK1	0.282093	0.493855	1.750682	0.010735
SLC51B	0.013413	0.023487	1.751034	0.60226
MAN1A1	10.59149	18.54843	1.751258	9.78E-05
AP3B1	10.34592	18.11957	1.751374	0.005432
ZNF622	19.31231	33.82817	1.751637	0.003118
DPM1	54.15701	94.8707	1.751771	0.001398
SH3RF1	2.659815	4.659873	1.751954	0.007024
RIMBP3B	0.030953	0.054228	1.751977	0.420094
LOC10272	1.622638	2.843432	1.752352	0.013919
CFAP99	0.030596	0.053633	1.752966	0.688261
PSMD9	22.18858	38.91789	1.75396	0.004164
ARL3	12.41765	21.80185	1.755715	0.001466
ZEB2	3.275768	5.752513	1.756081	0.002546
ING1	3.863704	6.787323	1.756688	0.00063
DNAJC16	2.151604	3.78099	1.757289	0.002065
OST4	180.643	317.4953	1.757585	0.00025
CBWD1	8.568628	15.06414	1.758057	0.005303
IQCK	2.828383	4.97323	1.75833	0.00248
B3GAT3	21.55058	37.8964	1.758486	0.01569
SMAD3	11.91241	20.9514	1.758787	0.021911
GDPD2	0.014513	0.025531	1.759173	0.645012
LOC10193	0.044941	0.079063	1.75925	0.508624
CHIT1	0.024005	0.042262	1.760532	0.502945
CTSO	1.971995	3.472025	1.760666	0.177646
FIZ1	2.65579	4.67766	1.761306	0.021059
DGAT2	4.061465	7.159796	1.76286	0.034023
FBXO39	0.01116	0.019678	1.763216	0.597725
MDS2	0.013194	0.023264	1.763216	0.597725
NRAP	0.013445	0.023706	1.763261	0.496377
TAS2R10	0.060402	0.106506	1.763276	0.533753
RAB7A	93.02007	164.1267	1.764423	0.000998
UGP2	20.37085	35.94607	1.764584	0.002267
CTSW	0.042731	0.075407	1.764684	0.508102
VMP1	22.25708	39.29927	1.765698	0.000676
CYB5D2	2.652417	4.684207	1.766015	0.15966
HIST1H2B	2.845548	5.027926	1.766944	0.465726
INMT	0.028827	0.051008	1.769477	0.427338
ALDH4A1	1.856538	3.286713	1.770345	0.037345
CPEB2	1.051262	1.861448	1.77068	0.015949
PSMC5	69.90924	123.8276	1.771262	0.001055
PLPP2	3.439757	6.093537	1.771502	0.030337
NPC1	11.06969	19.61465	1.771924	0.036171
BCL2A1	1.623426	2.876726	1.77201	0.426598
TSPAN32	0.018965	0.03361	1.772168	0.429085
NFS1	17.13037	30.35799	1.772173	0.006869

PAF1	33.79696	59.90503	1.772497	0.001775
SF3B4	41.83848	74.23084	1.774224	0.011546
SLC35E3	2.046818	3.632153	1.774537	0.003712
OPTN	17.32086	30.74884	1.775249	0.001482
DAG1	14.3228	25.4271	1.775288	0.000358
SMIM15	12.20626	21.67071	1.775377	0.001834
FBXO40	0.003251	0.005773	1.775576	0.722854
HSFX1	0.009516	0.016896	1.775576	0.722854
ALDH1L1	0.004768	0.008466	1.775576	0.722854
WBP4	8.257626	14.66621	1.776081	0.007617
ELL	3.13146	5.564745	1.777045	0.013917
CCDC136	3.241462	5.761752	1.777516	0.005576
MAGED1	67.79862	120.568	1.778325	0.005504
CEP55	20.06367	35.68508	1.778592	0.007646
SERPINA1	0.397836	0.708653	1.781269	0.297024
G6PC3	33.13351	59.04206	1.781944	0.004757
PTPN21	1.328707	2.367913	1.782119	0.007709
RHEB	45.36683	80.85746	1.782304	0.001998
RSRC1	2.794941	4.982616	1.782727	0.001243
ADPRHL2	19.19016	34.21661	1.783029	0.012347
F11	0.002561	0.004568	1.783914	0.590053
UGT2A3	0.006138	0.010949	1.783914	0.590053
RPP21	0.031325	0.055882	1.783914	0.590053
LOC10192	0.006726	0.011998	1.783914	0.590053
CD5	0.005236	0.00934	1.783914	0.590053
LOC10537	0.006085	0.010855	1.783914	0.590053
SATL1	0.017089	0.030487	1.784004	0.640609
GLE1	12.70356	22.66329	1.784011	0.003653
BTBD10	8.020718	14.31862	1.785205	0.001207
PTTG2	0.064222	0.114691	1.785847	0.313914
FAM96B	109.3267	195.245	1.785885	0.000615
GPN2	8.524047	15.23325	1.787091	0.003279
MED21	9.889425	17.67644	1.787408	0.008845
ADORA2A	4.154682	7.426935	1.787606	0.020009
PLEKHB2	17.9319	32.0555	1.787624	0.003709
LOC33986	0.259657	0.464186	1.787685	0.075522
10-Mar	0.004344	0.007765	1.787759	0.588552
TXN	323.5756	578.482	1.78778	0.000776
TMEM189	8.802604	15.74568	1.788752	0.007198
VWA3B	0.008545	0.015289	1.789298	0.545055
BEGAIN	0.530828	0.950723	1.791019	0.031101
SNAPIN	26.59141	47.65134	1.791982	0.009548
PPIAL4A	0.056048	0.100442	1.792063	0.570039
C5orf15	25.5893	45.85777	1.792068	0.002068
CD24	6.824911	12.23287	1.792385	0.088622
CIC	5.027695	9.012514	1.792574	0.000444
MOSPD3	8.214833	14.74271	1.794645	0.032867
OSBP2	1.651101	2.965082	1.795822	0.037523
TACC1	4.429036	7.954517	1.795993	0.002206
PROX2	0.005326	0.009568	1.796299	0.612394
GABRA5	0.001997	0.003587	1.796419	0.718215
CRYBA1	0.016671	0.029948	1.796419	0.718215
CEACAM2	0.00224	0.004024	1.796419	0.718215
LAPTM4A	90.33531	162.2848	1.796472	0.002406
ADK	10.13906	18.21845	1.796858	0.012779
PQLC1	5.4342	9.76481	1.796918	0.000216
OGDH	12.13032	21.79892	1.797061	0.000225
TMEM246	4.702506	8.451335	1.797198	0.012044

SLC9A7	5.477608	9.845248	1.797363	0.002654
ASF1B	37.23892	66.95857	1.79808	0.00094
BCKDHA	26.77144	48.15155	1.798616	0.000308
PFDN1	36.50199	65.69202	1.799683	2.16E-05
RNF222	0.005726	0.010307	1.799941	0.717441
CEMP1	0.01354	0.024371	1.799941	0.717441
SULT1A2	0.00784	0.014111	1.799941	0.717441
ALDH3B2	0.00659	0.011862	1.799941	0.717441
NOMO1	28.95176	52.11215	1.799965	0.000894
N6AMT1	5.21322	9.391644	1.801506	0.017033
C15orf62	0.09035	0.162772	1.801574	0.523923
STMN1	115.6733	208.4039	1.80166	0.004406
MRPS28	29.28121	52.77218	1.802254	0.007904
COMMD1	3.185421	5.744507	1.803374	0.000972
HIST1H2B	28.23784	50.92712	1.803506	0.277718
NLRP1	0.766064	1.382099	1.804156	0.039707
GHDC	5.643727	10.18406	1.804491	0.005527
WDR54	39.13213	70.64502	1.805295	0.061833
ARRDC2	3.76034	6.791589	1.80611	0.010981
SLC35A4	18.48857	33.39879	1.806457	0.005546
C6orf203	5.880923	10.62455	1.806613	0.008771
ABCG2	0.818834	1.480341	1.807865	0.064459
FGFR1	13.33354	24.10718	1.808011	0.00233
NEK6	9.489035	17.15637	1.808021	0.079217
SYS1	4.859753	8.794056	1.809569	0.006956
ADAT1	3.034751	5.492036	1.809715	0.045582
DNM1	10.96674	19.85047	1.81006	0.007527
GNA11	14.3641	26.00023	1.810084	0.000295
FAM104A	9.452846	17.11078	1.81012	0.000668
SLC35E1	13.23337	23.96985	1.811319	0.002362
NFKBIZ	4.825988	8.742914	1.811632	0.039304
CPED1	0.490013	0.887999	1.812194	0.124527
VOPP1	4.438637	8.050811	1.813802	0.000842
NCAM2	0.03073	0.055774	1.815005	0.405735
CACTIN	6.052263	10.98597	1.815183	0.005047
DNAJC3	7.505687	13.62913	1.815841	0.029129
SPATA25	0.12865	0.233612	1.81587	0.032413
MRPL35	9.381416	17.03975	1.81633	0.007531
WASHC2C	4.788579	8.697662	1.816335	0.003283
CHST1	2.288903	4.159405	1.817204	0.098428
SEMA4F	2.020839	3.673995	1.818055	0.006022
PCED1B	0.349099	0.63481	1.818425	0.047202
COPS7A	28.01598	50.94811	1.818537	0.000454
C19orf12	2.41791	4.39714	1.818571	0.000563
NUDT7	4.443404	8.083689	1.819256	0.028728
LPP	1.805818	3.286874	1.820158	0.042163
CCDC102I	0.071976	0.131023	1.820359	0.059264
SLC16A13	1.12661	2.050867	1.820387	0.037805
STPG4	0.007713	0.014046	1.821069	0.71286
CDH20	0.001824	0.003321	1.821069	0.71286
MLYCD	2.30219	4.194278	1.821864	0.048401
MMD	8.540016	15.56052	1.822072	0.000438
B3GNTL1	0.828761	1.510658	1.822791	0.020297
NECTIN3	4.805933	8.763552	1.823486	0.001841
GSS	27.59361	50.32556	1.823812	0.000704
CHMP7	6.492727	11.84223	1.823923	0.000879
RETREG2	10.01446	18.27993	1.825354	0.006093
RNF145	15.45087	28.21283	1.82597	0.000126

ZNF584	3.551297	6.486958	1.826645	0.005264
ASAH1	13.45347	24.57537	1.826694	0.000465
PHRF1	6.782392	12.40401	1.828855	0.000858
TM9SF2	46.12777	84.38543	1.829385	0.001912
ATP6V0B	36.6307	67.0141	1.829452	0.037684
GUCD1	15.01881	27.48407	1.829976	0.00066
BATF3	5.382959	9.851428	1.830114	0.120284
IL17D	0.12818	0.234788	1.831707	0.069234
KTN1	17.49459	32.05105	1.832055	0.000568
LOC10537	0.071706	0.131441	1.833062	0.199179
CDC16	25.53295	46.81662	1.833576	0.000538
GPX4	133.1438	244.1501	1.833732	0.002139
PLEKHM2	15.54001	28.49624	1.833734	0.004081
C1orf159	1.36961	2.511566	1.833783	0.052019
MXRA5	0.011836	0.02171	1.834324	0.381824
U2AF1L5	7.962186	14.60569	1.834382	0.487696
COX6B1	233.4718	428.4879	1.835287	0.000111
GINS4	10.49303	19.26471	1.835953	0.020341
TSPAN14	4.861694	8.927556	1.836306	0.001805
RAB5C	47.66674	87.58253	1.837393	4.74E-05
FAM13C	0.106794	0.19634	1.838496	0.166313
G3BP1	25.28897	46.51713	1.839423	0.002501
ARMC9	1.3065	2.403399	1.839571	0.091165
IFT52	21.38061	39.34933	1.840421	0.002942
HYLS1	2.107493	3.879263	1.8407	0.019574
BCL7C	15.64855	28.80879	1.840987	0.005103
RAB11FIP5	9.292179	17.10842	1.841163	0.007162
TPST1	3.561111	6.556899	1.841251	0.122122
SSX1	0.109185	0.201051	1.841371	0.343967
COMT	23.90525	44.02152	1.8415	0.009959
ACOT8	21.9545	40.45114	1.842499	0.003751
SUB1	36.82345	67.85517	1.842716	0.00688
FNDC4	6.88082	12.68017	1.842828	0.003595
HIST2H2B	2.320239	4.277262	1.843457	0.226799
EXTL2	10.2662	18.92699	1.843621	0.000245
KRT17	0.710997	1.310857	1.843689	0.45834
LYPLA2	12.44466	22.94686	1.843912	0.002289
VPS4B	5.587071	10.30608	1.84463	0.001864
DHX29	10.39221	19.17359	1.844996	0.000453
AVEN	2.682135	4.949293	1.845281	0.081197
GOLGA7B	0.22172	0.40916	1.845393	0.114456
ANGPT1	1.33914	2.471629	1.845684	0.169932
CLBA1	1.862765	3.438242	1.845773	0.09572
PSMD13	67.63198	124.8349	1.845796	0.001286
ZNF860	0.038708	0.071504	1.84724	0.043778
LOC10272	0.117283	0.21673	1.847922	0.166228
TCAF2	0.773034	1.42896	1.848509	0.042182
RAP1GDS1	9.67974	17.8983	1.849047	0.015613
TATDN2	10.18664	18.83821	1.849306	0.010327
AK9	0.301317	0.557397	1.849866	0.01672
FRMD7	0.054968	0.101708	1.850308	0.17606
AP4E1	2.079865	3.849761	1.850967	0.014373
RPEL1	0.087907	0.162723	1.851084	0.278464
RASA1	5.181834	9.594007	1.851469	0.001407
MAN2A1	5.339558	9.886563	1.851569	0.004434
GNPTG	13.75967	25.49734	1.853049	0.00031
SH2B2	1.734035	3.213581	1.853239	0.005661
OSGIN2	1.74033	3.225281	1.853258	0.024819

FAM156A	0.098955	0.183469	1.85407	0.313149
IQGAP1	29.00165	53.77509	1.854208	0.003659
FAM192A	12.41209	23.0191	1.85457	0.000114
SARAF	38.36107	71.17304	1.855345	0.004503
S100A14	0.096467	0.178985	1.855397	0.361014
MED27	4.817753	8.941207	1.855887	0.000251
UBASH3B	1.864496	3.46189	1.856743	0.023475
EPN1	45.17741	83.88288	1.856744	0.00051
UPRT	1.803075	3.352323	1.859226	0.017176
FBXO32	2.553073	4.747353	1.859466	0.060851
SSH1	3.456576	6.428678	1.85984	0.005503
BABAM1	61.56468	114.5308	1.860332	0.000483
TBCB	64.73762	120.4419	1.860462	8.67E-05
NAGLU	4.722131	8.786753	1.86076	0.052744
CBFB	13.75886	25.62227	1.862238	0.032319
ANKFN1	0.04592	0.085527	1.862498	0.106964
ZNF665	0.034823	0.06491	1.864006	0.041094
DPH2	4.749648	8.856539	1.864673	0.011569
ZNF285	0.721676	1.34575	1.864755	0.014425
LRRC59	62.5022	116.6609	1.866508	0.000386
MEPCE	8.829462	16.48115	1.866609	0.023206
ATAD3B	6.116026	11.41686	1.866712	0.051094
HMGA1	213.899	399.4004	1.867239	0.000148
TIGD5	2.122986	3.96442	1.867379	0.063221
FKRP	4.106837	7.670796	1.867811	0.085519
DHCR24	20.9871	39.20089	1.867856	0.090329
CCSER2	2.534253	4.736405	1.868955	0.000546
ABCD1	2.366554	4.423124	1.869015	0.005789
CTDP1	4.284573	8.010144	1.869531	0.000586
KLF13	6.511918	12.17865	1.87021	0.001945
CSTF2T	7.501891	14.0308	1.870301	0.00387
ABCB8	6.242261	11.67704	1.870642	0.000663
MICALL2	1.60815	3.009207	1.871223	0.00545
RTBDN	0.989481	1.851998	1.871687	0.05095
LOC10272	0.628328	1.176317	1.872138	0.01512
CASP9	2.485211	4.653454	1.872458	0.001866
ZCCHC24	6.633341	12.42097	1.872506	0.019312
OGFR	28.44699	53.27451	1.872765	0.002139
SHKBP1	28.0842	52.60508	1.87312	3.10E-05
TMEM159	5.207083	9.754027	1.873223	0.001727
C9orf16	34.89562	65.37316	1.873392	0.0055
UBE2B	14.4798	27.13301	1.873853	0.001674
DDC	0.027915	0.052312	1.873976	0.683307
USP5	23.09769	43.30491	1.874859	0.001242
B3GALT4	0.264385	0.496203	1.876824	0.164072
BMP2	1.675766	3.146077	1.877396	0.101962
LSG1	7.32052	13.74736	1.877921	0.009223
C1orf122	29.05512	54.57427	1.878302	0.000375
MRPL41	73.814	138.6591	1.878494	0.003344
MAPK6	10.80146	20.31253	1.880536	0.005455
MRPL51	174.9982	329.52	1.882991	0.001364
KCNC4	2.342149	4.410718	1.883192	0.004478
MFSD6	2.29409	4.321784	1.883878	0.007874
ZNF112	1.010297	1.903848	1.884444	0.010935
FAIM	5.064216	9.544636	1.884721	0.022572
EIF2S1	27.14	51.16015	1.885046	0.0001
UEVLD	4.03884	7.613805	1.885146	0.001026
HSP90AA1	297.5548	560.9816	1.885305	0.001009

ARHGEF1	14.4217	27.19565	1.885745	0.00022
FADS3	13.98283	26.37157	1.885997	0.021346
CCDC148	0.237649	0.44852	1.887324	0.037292
ZNF557	0.898199	1.695316	1.88746	0.023579
TCTN3	16.10511	30.41255	1.888379	0.003386
TRAF3IP2	3.88358	7.33701	1.889239	0.041267
EFCAB2	0.34454	0.650928	1.88927	0.191046
TFE3	23.4725	44.35113	1.889493	0.000558
SPAG8	0.431609	0.815797	1.890129	0.00798
SERTAD3	9.390714	17.76253	1.8915	0.0153
BTBD11	1.553174	2.938468	1.891911	0.009967
FAM161B	1.169299	2.212802	1.892417	0.019805
DST	4.142728	7.839827	1.892431	0.023299
EPHB1	0.478266	0.905123	1.892512	0.038907
SEC22B	18.31626	34.67691	1.893231	0.030581
ITFG1	13.59229	25.75172	1.894583	0.003779
TAF10	71.7422	135.9494	1.894971	0.000392
ADPGK	10.71051	20.30185	1.895507	0.006998
LZTS2	10.41548	19.75599	1.89679	0.000255
IER3IP1	44.53296	84.50071	1.897487	0.000757
CTTN	39.06783	74.14801	1.89793	0.004416
PLPP1	9.009491	17.1043	1.898476	0.000966
LRRC29	0.382097	0.725528	1.898804	0.056492
ATP13A3	9.547835	18.14062	1.899972	0.029925
YWHAQ	149.9713	285.0024	1.90038	0.000431
APPL2	4.056697	7.711415	1.90091	0.000422
COA3	37.23127	70.77515	1.90096	0.002176
TNFAIP3	1.718245	3.268865	1.902444	0.149056
ANTXR1	2.857078	5.439408	1.903836	0.004834
SLC25A43	3.884778	7.400247	1.904934	0.007247
PCBD2	2.115017	4.029956	1.905401	0.003717
CES2	7.432944	14.16797	1.906105	0.002231
CCDC50	8.477541	16.15994	1.906206	0.005487
ZMAT2	55.26407	105.4114	1.907413	0.000272
MICALL1	10.89235	20.78316	1.908052	0.01001
MED31	4.948363	9.444166	1.908543	0.005443
PLTP	39.99053	76.34479	1.909072	0.012965
MFSD2B	0.504473	0.963292	1.9095	0.007804
MESD	16.41588	31.34969	1.909718	0.010302
RNF11	14.79267	28.2553	1.910088	0.000845
UGGT2	7.78559	14.89309	1.912905	0.001795
COMMD1	4.736124	9.061507	1.913275	0.005598
SNX17	27.11661	51.91581	1.914539	0.004432
ESYT1	20.31625	38.91859	1.915638	0.000422
COL1A2	44.34367	84.94647	1.915639	0.034048
OXTR	0.362414	0.69427	1.915683	0.113252
ARMC2	0.584898	1.121296	1.917078	0.025353
BNIP2	6.127145	11.75204	1.918029	0.003007
TRAPPC1	107.9596	207.337	1.920504	0.000382
SNED1	0.558837	1.073311	1.920615	0.020218
USF2	13.30944	25.57642	1.921675	0.000678
SHCBP1	8.388992	16.12751	1.922461	0.000745
9-Sep	23.45265	45.09009	1.922601	0.00061
C1orf216	7.930168	15.24746	1.922716	0.034809
DDX60	0.419277	0.806293	1.923055	0.022597
TRIM44	10.56776	20.33209	1.923973	0.002408
SLC35C2	5.145944	9.901313	1.9241	0.002063
LOC10537	0.065287	0.125638	1.924393	0.229802

NEXN	0.874829	1.683721	1.924627	0.019212
NEK10	0.183901	0.353987	1.92488	0.053548
PPP6R1	24.10326	46.40904	1.925426	0.019244
SNX15	10.69716	20.60049	1.925791	0.00037
ZNF20	0.614912	1.184441	1.926194	0.005024
LOC10012	0.035238	0.067943	1.928104	0.333178
RRM2	49.98339	96.3778	1.928197	0.001646
RHBDF2	1.710503	3.298597	1.928437	0.009894
STARD13	1.737914	3.351471	1.928444	0.024
TMEM203	23.38567	45.10751	1.928852	0.004933
PEA15	58.66081	113.1582	1.929026	0.005701
THOC7	41.90729	80.84576	1.929157	0.012179
CDKN1A	44.16282	85.21298	1.929519	0.011706
ABHD13	2.102598	4.057568	1.929787	0.001114
RRAGC	12.1795	23.50767	1.930102	0.000221
NAV2	0.830938	1.604647	1.931127	0.024808
LARGE1	2.150876	4.154003	1.931308	0.000646
ZBTB1	2.937398	5.673875	1.931599	0.021115
CLOCK	1.84602	3.568996	1.933346	0.001696
GNAT2	0.042295	0.081803	1.934098	0.349363
DCTN6	24.21989	46.84584	1.934189	0.00285
ESPNL	0.460481	0.891323	1.935635	0.006402
TPRN	4.209056	8.148337	1.935906	0.00136
NCLN	19.21956	37.20727	1.935907	0.002689
AKT1	43.02424	83.43972	1.939365	0.000657
NDUFAF2	40.24509	78.0589	1.939588	0.014194
HIST2H2A	0.140644	0.272804	1.939672	0.509313
UBTF	10.75788	20.87293	1.940245	0.008082
FKBP15	6.917392	13.42189	1.94031	0.007059
STARD8	1.178063	2.285809	1.940312	0.016082
ZNF565	0.825421	1.601861	1.940658	0.022874
DNAJB1	27.68026	53.72883	1.941052	0.013593
SURF4	48.14263	93.46252	1.941367	0.014359
MALT1	4.60358	8.949854	1.944107	0.028262
TNK1	0.017607	0.034231	1.944206	0.577008
AP2A1	23.28683	45.29614	1.945139	0.000692
TEX261	20.55235	40.01394	1.946928	0.015656
C6orf47	4.91491	9.56931	1.946996	0.004435
TMEM87B	3.625435	7.059082	1.947099	0.055469
ETFB	72.12745	140.4414	1.947129	0.000354
SURF2	13.17304	25.66091	1.947988	0.024366
ARAP3	1.962502	3.82336	1.948207	0.003338
MRPL47	37.89695	73.83467	1.948301	0.004033
ATG101	24.41672	47.58651	1.948931	0.001352
ZNF511	19.82345	38.65356	1.94989	0.003773
EGLN2	18.60942	36.29177	1.950182	0.000209
VANGL1	2.201808	4.294324	1.950363	0.015064
ANKRD29	1.954674	3.812929	1.950672	0.007665
MORN2	23.64019	46.16244	1.95271	0.001931
TRIM62	1.426697	2.786782	1.95331	0.003692
KDSR	6.589392	12.87167	1.953392	0.001244
ZNF845	1.177679	2.30064	1.953537	0.055487
NDUFS6	130.586	255.2738	1.954833	0.001486
SDF2	18.08803	35.36625	1.95523	0.008287
TK1	53.04881	103.7259	1.955292	0.000338
TMEM208	30.4344	59.51122	1.955393	0.036444
SIPA1L3	2.125278	4.156271	1.955636	0.000643
MAFB	1.857918	3.633482	1.955674	0.379068

PIN1	55.81817	109.2207	1.956723	0.000206
ORAI1	13.58283	26.58455	1.957217	0.005825
SLC39A14	18.47029	36.17212	1.958395	0.003683
TRPM3	0.014618	0.02864	1.959242	0.242179
DUSP28	0.389751	0.763899	1.959968	0.043631
NR2F1	18.01159	35.31494	1.960679	0.001662
ARL4C	16.08389	31.53758	1.960818	0.023814
CHRNA9	1.472746	2.889595	1.962046	0.213149
SGCB	12.21955	23.99826	1.963924	0.000336
AIDA	10.52252	20.68586	1.965865	0.000487
ARHGEF1(	1.382252	2.723531	1.970358	0.022693
TIMP2	25.14751	49.55546	1.970591	0.040953
PSD4	0.55623	1.096292	1.970932	0.037032
WDR25	2.332268	4.597113	1.971092	0.003754
BLOC1S1	58.53756	115.4295	1.971888	1.43E-05
CD3EAP	2.804264	5.535662	1.974016	0.005206
ATF5	6.515499	12.8767	1.976319	0.075955
IKBIP	6.576225	13.00392	1.977414	0.000995
CDC42EP4	7.770402	15.37033	1.978061	9.86E-05
PSMA2	71.74821	141.9429	1.978348	0.005213
ASPSCR1	5.902755	11.68654	1.979845	0.000246
RBM19	3.237925	6.411231	1.980043	0.009987
MEGF10	0.281454	0.557328	1.980176	0.011226
RAB2A	18.2337	36.10763	1.980269	0.001796
MAPRE1	51.96165	102.9817	1.981878	0.000892
LOC10536	0.029603	0.058707	1.983156	0.478944
XYLT2	9.536931	18.91455	1.983295	0.000349
TNFSF12	0.216906	0.430457	1.984529	0.265496
CLPTM1L	27.19627	53.98897	1.985161	0.00425
TSPAN5	8.827258	17.53063	1.985966	0.020066
TCHP	5.187445	10.30364	1.986266	4.50E-05
TRAPPC3	35.75434	71.02726	1.986535	0.000464
MIEN1	25.42663	50.52804	1.98721	0.004777
DUSP5	6.666706	13.25669	1.988491	0.072535
FOSB	2.708406	5.386259	1.98872	0.168468
GSTT2B	0.560607	1.115335	1.989514	0.040011
STK17B	3.381897	6.729658	1.989906	0.001016
PELO	11.92908	23.73839	1.989959	0.001217
DGKZ	7.655548	15.23594	1.990183	9.10E-05
ARMCX4	0.340018	0.676758	1.990361	0.099219
CHL1	0.051544	0.102695	1.992377	0.073662
BORCS8	2.895141	5.771644	1.993562	0.040951
OR7C1	0.016345	0.032593	1.994049	0.445948
PRRG2	0.11462	0.228564	1.994097	0.222415
TLDC1	3.802519	7.583938	1.994451	0.001552
PSMB6	114.8814	229.1396	1.994575	0.000783
PYCR3	2.847026	5.683932	1.996446	0.039853
MRPL36	23.17355	46.28482	1.997312	0.001217
TMEM35B	10.26462	20.51602	1.998712	0.016879
OLFML2B	0.612481	1.22517	2.00034	0.09199
MBD3	40.05741	80.17485	2.001499	0.000533
FAM19A3	0.353603	0.707759	2.001568	0.226421
BIN3	4.946607	9.902105	2.001797	0.001008
CLSTN2	0.043029	0.086214	2.00364	0.076337
ACOX3	2.17198	4.352384	2.003879	0.000337
COPE	104.1931	208.882	2.004759	0.002459
LUM	4.767942	9.560689	2.005202	0.028586
KIRREL1	6.468226	12.97171	2.005451	0.000642

ODAPH	0.058595	0.117536	2.005903	0.595761
ATP13A2	7.646289	15.34273	2.006559	0.001214
RDH11	40.36676	81.01743	2.007033	0.000942
MTRF1	0.801949	1.610078	2.007705	0.007873
ALG1	3.295595	6.618449	2.008272	0.011862
ITPRIP	4.501979	9.043131	2.008701	0.029876
USP4	7.755974	15.58325	2.009193	0.01009
HOXB2	7.238265	14.55178	2.010397	0.010526
DLGAP4	9.539303	19.18573	2.01123	0.00062
OPN3	2.868023	5.768513	2.01132	0.043331
CC2D1B	4.033255	8.112249	2.011341	0.008429
CDC42	58.54733	117.8182	2.012358	0.000401
CABLES2	4.706091	9.473157	2.012957	0.000497
ATG3	21.91877	44.13246	2.013455	0.005151
MB21D2	1.707735	3.440012	2.014371	0.002262
FAM50B	2.24778	4.529059	2.014904	0.052273
ZP3	0.253106	0.510233	2.015883	0.138495
FAM46B	0.899388	1.813061	2.015883	0.129739
SELENOT	18.4901	37.27957	2.016191	0.002403
SLC12A8	1.36116	2.74479	2.016508	0.012821
SEC61A1	114.5419	230.9837	2.016587	0.001744
LOC10013	0.132324	0.266848	2.016633	0.052149
ZGPAT	7.205622	14.53319	2.016923	0.006821
TOGARAM	0.083896	0.169261	2.017516	0.076499
AP1M1	35.26485	71.18092	2.018466	0.000251
MAPK10	0.12737	0.257241	2.019634	0.09054
SCUBE2	0.030973	0.06256	2.019844	0.141384
MRPL23	12.98043	26.22064	2.020013	0.008405
PARD6A	2.053017	4.150005	2.021418	0.062627
TPP1	23.34174	47.18591	2.021525	0.005781
DYRK1B	3.756782	7.598011	2.022478	0.014235
BCAR3	2.779776	5.623029	2.022835	0.007194
NDUFA8	45.44876	91.95711	2.023314	0.004916
DUSP7	6.802048	13.76473	2.023616	0.00443
MRGBP	17.59545	35.60865	2.023741	0.000161
MLLT11	19.55633	39.58124	2.02396	0.034691
ZG16B	0.136995	0.277325	2.02434	0.207355
SOD2	21.89464	44.33642	2.02499	0.005583
CALM2	69.31136	140.3662	2.025155	0.000287
LENG1	9.165826	18.56636	2.025607	0.019084
BCL9L	2.837202	5.747415	2.025734	0.003347
NUBP1	11.65409	23.61563	2.026382	3.59E-05
LRRC8E	3.579048	7.255512	2.027219	0.001268
RBM15B	7.959013	16.14507	2.028527	0.018246
LRP10	10.1372	20.56415	2.028583	0.001469
LRRC46	0.383197	0.777364	2.028626	0.018519
FADD	17.17059	34.83537	2.028781	0.000781
YIPF5	15.64945	31.76286	2.029647	0.00376
FITM2	4.048882	8.218977	2.029938	0.000234
CAT	14.93433	30.31673	2.030002	0.002654
MGST3	48.6073	98.72643	2.031103	0.00322
FAM131A	6.236257	12.67111	2.031846	0.012093
ACOT7	27.45313	55.81488	2.033097	0.000309
PSMA1	59.64376	121.2675	2.033197	0.004551
ARSE	0.438071	0.890807	2.033478	0.085484
LOC10537	0.153155	0.311515	2.03398	0.271654
HLA-DRB1	53.80082	109.4434	2.034232	0.10944
PTGIR	0.074681	0.151922	2.03428	0.15012

PKIG	14.94196	30.39626	2.034289	3.14E-05
GTF2A2	27.91271	56.78917	2.034527	0.00484
FUT11	3.334444	6.786555	2.035288	0.015877
DECR1	6.555373	13.34658	2.035976	0.00398
YJU2	9.374971	19.10857	2.038254	0.000843
SH3BP5L	4.402052	8.973993	2.038593	0.000943
TFPT	13.44791	27.42418	2.039289	0.000538
NDUFB6	60.99265	124.454	2.040476	0.003881
DNAH7	0.094302	0.192503	2.041339	0.050013
MTFR1L	16.62933	33.95089	2.041627	6.17E-05
PGAM1	96.30967	196.6753	2.042114	0.0097
BLVRA	30.12042	61.52024	2.042476	0.000905
ZNF765	0.772405	1.577748	2.042644	0.020626
CLN3	8.103201	16.55746	2.043323	0.000415
SIRT7	3.058987	6.251377	2.043611	0.000227
GAB3	0.265368	0.542375	2.043861	0.036487
TRMO	0.664407	1.358618	2.044859	0.014433
LOC11226	0.437538	0.895323	2.046273	0.022679
ZHX3	3.345691	6.846554	2.046379	7.22E-05
DNASE1L1	4.922001	10.07281	2.046487	6.21E-06
TRAPPC2L	11.81462	24.1962	2.047988	0.00019
SEL1L	7.293316	14.93794	2.048168	0.00411
DNAJC5	12.54405	25.69557	2.048428	1.14E-05
NDUFV2	63.48853	130.0717	2.048744	0.000995
LOC10798	0.226108	0.463254	2.048817	0.266825
BUD23	51.85765	106.3189	2.050206	0.00941
ERF	19.03371	39.02802	2.050468	0.000554
GCNT1	2.606916	5.346282	2.050807	0.042147
FPGS	12.70622	26.06259	2.051168	0.003144
ZNF319	1.433272	2.942076	2.052699	0.01145
INAFM2	5.752956	11.81015	2.052885	0.000371
SRP14	116.8394	240.1284	2.0552	0.000877
CBLN3	0.129597	0.266351	2.055231	0.022899
TFDP1	35.4548	72.8753	2.055442	0.000101
GIPC1	55.96962	115.0685	2.05591	0.001564
UBL5	150.4471	309.3855	2.05644	0.002612
SHOX2	2.524271	5.194666	2.057888	0.004147
CLVS1	0.034591	0.07125	2.059825	0.284417
ABTB1	6.361544	13.10628	2.060236	0.005801
HYAL2	8.799677	18.13258	2.060596	0.0017
DERL2	8.084146	16.65998	2.060821	0.002275
SDHB	65.20127	134.4404	2.061929	0.002686
ADRA1B	0.336295	0.693506	2.062197	0.013823
TMEM223	10.21376	21.07999	2.063882	0.004711
DTYMK	48.92476	100.9837	2.064062	3.25E-05
VSIG10	2.664038	5.499214	2.06424	0.001002
S100A13	30.03331	61.99851	2.064325	0.000105
TMED7	11.0328	22.77872	2.064636	0.00156
DNAJC13	6.336976	13.08418	2.064735	0.003538
REX1BD	13.30896	27.48092	2.064843	0.00938
SAMD1	17.76397	36.68094	2.064907	0.001827
CCNY	8.71539	17.9985	2.06514	0.000259
COTL1	118.4167	244.5738	2.065367	0.000736
NR3C1	1.977938	4.087431	2.066511	0.001404
BTD	1.794104	3.707759	2.066636	0.00085
NRP2	4.268012	8.824801	2.067661	0.009994
SLC6A6	9.173705	18.97086	2.067961	0.002759
FAM129A	3.676696	7.604462	2.068287	0.02924

KCTD18	3.591592	7.429845	2.068678	0.013994
RASGRP3	0.065333	0.135159	2.068777	0.456517
TMX3	5.864143	12.13366	2.069127	0.000937
LSM10	33.27555	68.85816	2.069332	0.000117
PET100	122.8248	254.273	2.07021	2.45E-05
ABHD2	9.658875	19.99916	2.070547	0.000967
TMEM102	1.639639	3.397785	2.072276	0.019106
ARNTL	2.760169	5.723346	2.073549	0.015525
RGL1	1.507184	3.12882	2.075938	0.001407
C11orf49	4.383046	9.099009	2.075955	0.000124
PRDX1	221.215	459.3906	2.07667	0.001891
SLC4A2	21.19675	44.01986	2.076726	0.005296
AHDC1	1.621913	3.370646	2.078191	0.003439
EHD3	3.060537	6.362016	2.078726	0.002644
PTX3	18.54821	38.57252	2.079582	0.008064
IL22RA1	0.138671	0.288394	2.079699	0.027172
IKBKG	1.915062	3.983324	2.079997	0.001618
ID3	32.02414	66.6156	2.080168	0.366815
VTI1B	47.66466	99.15163	2.080192	8.87E-05
GCK	0.205577	0.42769	2.080436	0.101508
ARHGAP1	4.88909	10.17181	2.080512	0.000892
ASF1A	11.55422	24.04097	2.08071	0.009169
SHROOM4	0.244585	0.508934	2.080803	0.082939
STOML1	2.691713	5.602119	2.081246	0.026653
ARMC7	1.816438	3.780616	2.081335	0.050031
TOX2	4.484042	9.336118	2.082077	0.016464
PODNL1	0.164944	0.343449	2.082213	0.209797
SLC52A2	14.65607	30.54262	2.083957	0.00301
VCL	34.0707	71.00961	2.084184	0.000734
POP1	1.982449	4.13205	2.084316	0.018543
TIGIT	0.004173	0.0087	2.085061	0.163395
RAB23	6.110333	12.74083	2.085128	0.001006
MTCH2	20.39456	42.53862	2.085783	0.006412
FRG1	28.33536	59.11235	2.086169	0.000833
SMAD7	3.386985	7.067377	2.086628	0.266411
RAB8A	19.99944	41.74355	2.087236	0.00016
SSC5D	1.137509	2.375045	2.087934	0.033251
C11orf24	15.01767	31.36296	2.088404	0.000808
FBXL22	0.034884	0.072854	2.088457	0.044461
PSMD8	120.1971	251.0663	2.088789	0.000847
RRS1	13.28633	27.75262	2.08881	0.009649
SLC4A4	0.280537	0.586138	2.089342	0.001332
PITPNM1	10.70132	22.36244	2.08969	0.005214
LEPROT	6.275798	13.11615	2.089958	7.91E-05
BCAP31	78.59517	164.3837	2.091524	0.00012
FTL	651.9734	1363.644	2.091564	0.020418
LNPEP	1.936858	4.051262	2.091667	0.005016
PIGU	14.4356	30.20851	2.09264	0.000426
ARHGAP9	0.022105	0.046264	2.092937	0.442221
LOC10012	0.007548	0.0158	2.093168	0.506069
MAGIX	0.181798	0.380802	2.094649	0.044164
HECW2	0.413207	0.865938	2.095651	0.004587
DCAF6	10.09236	21.16132	2.096767	0.00092
TMEM250	10.8705	22.79754	2.097194	0.005144
EXT1	20.24098	42.46048	2.097748	0.000164
SERPINB6	10.57213	22.18182	2.098141	0.000443
FUS	47.71214	100.1123	2.098257	0.007197
CD320	37.67231	79.06733	2.098818	0.002037

PFKL	21.34136	44.79528	2.098988	0.008004
SPARC	147.2666	309.1913	2.099535	0.007752
GALNT12	5.003838	10.50631	2.09965	0.00046
GALNT7	4.003907	8.408033	2.099957	0.057444
MED19	9.165202	19.24964	2.100297	0.001705
PDLIM5	5.010924	10.52445	2.100301	0.000233
ASB14	0.02358	0.049527	2.100347	0.086244
C8orf58	3.499015	7.349201	2.100363	0.01408
F2RL2	0.473467	0.99448	2.10042	0.008964
HPS3	8.296312	17.44247	2.102437	0.000443
SMPD2	3.33917	7.022376	2.10303	0.007164
ACTR3	25.84207	54.35376	2.103305	0.000151
TACR2	0.038982	0.08203	2.104313	0.218135
TBXAS1	0.3167	0.666522	2.104581	0.005818
TBC1D19	1.093689	2.301885	2.104698	0.00147
PSMD1	26.72761	56.27019	2.105321	0.000315
TMEM165	13.75009	28.96024	2.106186	0.001369
AAR2	12.25032	25.80511	2.106483	0.00256
SOX9	3.399625	7.163864	2.107251	0.01852
BLCAP	12.08309	25.46537	2.107522	3.33E-05
PEBP1	113.8419	239.9809	2.108019	0.001309
IFT43	18.41593	38.84379	2.109249	0.001393
PYM1	9.496147	20.03076	2.109356	0.002562
TM2D2	6.267578	13.22694	2.110375	0.000263
POP5	28.16206	59.45122	2.111039	0.003379
MSRB1	7.33183	15.48129	2.111518	0.003761
RAB36	0.87098	1.840306	2.112915	0.014534
FAHD1	7.554442	15.96552	2.113395	0.005093
SLC39A11	1.29088	2.729705	2.114608	0.00033
LRIG1	10.3238	21.83483	2.115	0.049154
TRABD2A	0.444395	0.940117	2.115496	0.088919
ATP6AP2	48.80515	103.2506	2.115567	0.00067
NOTCH2	12.71028	26.89895	2.116315	0.001127
ERGIC3	199.7845	422.8784	2.116672	0.000116
CFB	1.690218	3.57793	2.116845	0.141794
ENO2	55.7311	118.0497	2.118201	0.055537
TAF6L	4.019234	8.515404	2.118664	0.031751
DOHH	5.441194	11.52987	2.118996	0.014486
CTNS	2.296208	4.865712	2.119021	0.025418
NUMB	18.74091	39.71337	2.119074	0.009742
RBM3	26.30449	55.74437	2.119196	0.010061
WDR83OS	34.17734	72.44023	2.11954	0.000524
CD47	8.463336	17.94387	2.120188	0.017671
LGALS3BP	75.35107	159.8623	2.121567	0.007279
GSKIP	5.738666	12.18691	2.123648	0.004717
ARMH4	0.600332	1.276721	2.126693	0.01471
ITGB3	12.13553	25.82679	2.128197	0.093873
CLDN11	0.072934	0.155333	2.129787	0.010756
CYBC1	5.841915	12.44339	2.130019	0.006174
C1GALT1C	15.21957	32.43776	2.131319	0.001803
ZNF653	2.058693	4.388785	2.131831	0.002944
ZC2HC1C	0.19473	0.415184	2.132095	0.188776
FSTL1	39.97104	85.25362	2.132885	0.002091
DOK3	0.176042	0.375489	2.132946	0.314917
TOMM40	37.33493	79.66557	2.133808	0.003525
COLGALT1	35.8503	76.54138	2.135028	3.63E-05
PROCR	29.24822	62.44954	2.135157	0.036237
CAPN5	2.779212	5.935859	2.135807	0.029094

ASB1	8.201433	17.52397	2.136696	0.006598
NPC2	56.34232	120.3897	2.136755	0.000851
CTDSP1	8.598708	18.37673	2.13715	0.006277
NR4A3	0.325754	0.696225	2.13727	0.090556
CHCHD1	29.71382	63.53406	2.138199	0.002372
FICD	1.48377	3.174095	2.139209	0.003477
SLIRP	106.5213	228.0263	2.140664	0.002133
FNDC3B	3.049766	6.532528	2.141977	0.017176
DUSP18	0.941738	2.017639	2.142465	0.00192
UFD1	29.91267	64.14093	2.144273	0.00243
SRA1	16.82539	36.09166	2.145072	0.001134
DNAJA1	85.03937	182.474	2.14576	0.001961
SMIM4	22.56947	48.44688	2.146567	0.001672
LACC1	1.16049	2.491302	2.146767	0.147533
NEIL2	5.503076	11.81799	2.147524	0.007246
LHX6	0.9795	2.104301	2.148341	0.042201
MAMLD1	2.081394	4.471719	2.148425	0.002802
FOXD4	0.269235	0.578668	2.149305	0.035113
ZNF438	0.310964	0.668509	2.149799	0.005568
ROMO1	126.1768	271.2797	2.149997	7.83E-05
METTL9	38.96682	83.78365	2.150128	0.089958
RGL3	0.525786	1.130734	2.15056	0.018298
KIF9	1.323612	2.84653	2.150577	0.050332
AK1	8.553834	18.3957	2.15058	0.000666
P4HB	295.4021	635.6414	2.151783	0.000574
POPDC3	6.301991	13.56688	2.152793	0.000673
PPIB	487.3685	1049.971	2.154368	0.000268
MVK	4.005088	8.628679	2.154429	0.023412
ECHDC1	9.507869	20.48956	2.155011	0.009149
MRM1	0.878106	1.892969	2.15574	0.007922
SMAD2	2.674278	5.766418	2.156252	0.000468
PARVB	18.66069	40.26356	2.157667	0.001156
LIG4	1.826507	3.941655	2.158029	0.009233
SETBP1	0.174253	0.376287	2.159425	0.000106
PIGBOS1	3.927285	8.483934	2.160254	0.013558
FAM174A	5.398724	11.67101	2.161809	0.003777
NFKBIE	3.244695	7.016678	2.162508	0.03049
C15orf65	3.500959	7.573011	2.163125	0.003258
PC	3.575306	7.741788	2.16535	0.000313
ST5	2.289828	4.960259	2.166214	0.000577
EGR1	17.82724	38.62653	2.166715	0.198733
DLG1	4.525994	9.810102	2.167502	0.003571
SURF1	17.85958	38.72268	2.168174	0.004473
AMDHD2	1.705585	3.698809	2.168645	0.00489
TPRA1	6.006398	13.0347	2.170136	0.127375
EMC1	12.44135	27.00432	2.17053	0.000373
LOC10099	0.485488	1.0542	2.171422	0.000207
CPEB1	2.600414	5.648076	2.171991	0.006013
ANKDD1A	0.646129	1.405597	2.175411	0.047558
CRYBA2	0.042043	0.091505	2.176477	0.558499
BAIAP2	2.21335	4.818488	2.177012	0.022045
DCBLD1	4.935949	10.74913	2.177722	0.001913
SMS	68.20872	148.6167	2.178851	0.010521
DNAJC11	11.01081	23.99228	2.178974	0.007691
EBNA1BP2	43.50842	94.84518	2.179927	0.010334
ATP10D	1.235084	2.693849	2.181106	0.003042
ACYP2	0.596762	1.301776	2.1814	0.026415
SPPL2A	3.820194	8.334032	2.181573	0.007135

NADSYN1	8.090903	17.65719	2.182351	0.000334
PGBD2	0.776317	1.694526	2.182776	0.001344
OXCT1	12.72641	27.79116	2.183739	0.025237
GPR1	0.242129	0.529078	2.18511	0.041074
HS3ST3A1	2.010339	4.396758	2.187072	0.001905
NIPA2	10.9956	24.05559	2.187747	0.00408
ANKRD34/	0.85538	1.871851	2.188327	0.035053
EMP2	8.277227	18.11479	2.18851	1.07E-05
NAGPA	2.299665	5.032858	2.188518	0.01072
ATP2C2	0.006581	0.014407	2.189177	0.508158
RNF7	23.89474	52.31681	2.18947	0.000967
YIPF1	11.71327	25.65138	2.189941	0.002471
FAT4	0.34168	0.749034	2.192211	0.029353
C10orf143	0.29802	0.653761	2.193682	0.074304
AKT1S1	20.4175	44.79956	2.194174	0.008848
EIF3CL	5.39662	11.84296	2.194515	0.297572
NCCRP1	0.015362	0.033718	2.19484	0.476742
LRRC8A	5.837181	12.81577	2.195541	0.002072
RRP9	13.48447	29.62635	2.197072	0.013103
ZNF771	4.342565	9.542233	2.197373	0.011622
RABAC1	99.70595	219.2044	2.198508	0.000802
CPNE1	52.58664	115.6264	2.19878	0.000982
TSHZ3	2.636478	5.797894	2.199106	0.006325
TNFSF15	0.0055	0.012106	2.201019	0.331186
PRG2	0.169134	0.372466	2.202197	0.230325
MANBAL	25.68374	56.56972	2.20255	4.55E-05
AP3S2	0.050212	0.110715	2.204937	0.055693
B4GALT1	22.44888	49.50177	2.205088	0.001474
COQ10B	7.855969	17.33975	2.207207	0.038737
SLAIN2	3.792244	8.370298	2.207215	0.000958
CFAP157	0.014886	0.032865	2.207736	0.39839
EGR2	1.129251	2.494776	2.20923	0.002035
MTG1	4.133587	9.136674	2.21035	0.011314
SAMD14	1.228485	2.716434	2.211207	0.011081
SRSF3	43.12551	95.36256	2.211279	0.005707
C19orf70	26.05172	57.62425	2.211917	0.002289
ATP5F1E	538.7056	1192.165	2.213018	5.54E-05
CNTN2	0.003982	0.008812	2.213202	0.477518
ARPC5	70.55241	156.1692	2.213521	4.21E-05
CRELD2	13.69804	30.32502	2.213822	6.02E-05
DYNAP	0.014326	0.031725	2.214441	0.408833
BAG3	14.44447	32.01755	2.216595	0.007202
ZCWPW1	0.335247	0.743183	2.216819	0.040685
2-Mar	7.702651	17.07781	2.217134	0.00016
EOGT	1.577042	3.497239	2.217594	0.008887
LANCL2	1.669258	3.702352	2.217963	0.015416
RTL8C	85.61642	189.9856	2.219032	0.007095
HIPK4	0.011853	0.026307	2.219382	0.330461
PTGFRN	5.154977	11.4504	2.221232	0.002322
SELENON	27.88616	61.95564	2.221734	0.002304
C9orf78	23.94176	53.2042	2.222234	0.000625
LGSN	0.017504	0.038902	2.22252	0.146103
SPATA33	3.390827	7.538317	2.22315	0.00058
ZNF593	24.04514	53.45797	2.223234	0.000273
YBX1	176.3778	392.1695	2.223464	0.000213
CAST	23.96813	53.31076	2.224235	0.001643
DDA1	11.78708	26.22421	2.224827	0.002485
FHOD3	2.253381	5.013746	2.224988	0.00092

LOC10272	0.029023	0.06459	2.225473	0.416483
HIST1H2A	0.077355	0.172158	2.225562	0.551196
CMPK1	29.14009	64.85359	2.22558	0.00014
ITGAD	0.044611	0.099314	2.226191	0.120059
MAP3K11	9.543437	21.24573	2.226213	0.000697
SPHK2	1.743063	3.880962	2.226518	0.016004
NOL3	5.064476	11.27729	2.226744	0.002294
CHP1	16.26141	36.21729	2.227192	8.88E-05
RALY	28.37007	63.21314	2.228163	5.87E-06
EPHA2	7.718225	17.20545	2.229198	0.003091
H6PD	3.218601	7.176848	2.229804	0.002839
ZYX	62.1203	138.6069	2.231266	0.000273
SNAPC1	10.71054	23.90047	2.23149	0.001956
CDKN2AIF	15.0259	33.53845	2.232043	0.001467
RAB41	0.012703	0.028358	2.232332	0.145455
LOC10798	0.085041	0.189906	2.233105	0.272134
NAMPT	64.44126	143.9451	2.233741	0.225906
PGAM4	0.895774	2.00114	2.233979	0.027753
ACAP1	0.607183	1.356652	2.234339	0.09714
IL10RB	9.624598	21.51492	2.23541	0.000447
METRN	7.538931	16.85781	2.236101	0.027008
APOL2	5.804436	12.98207	2.236577	0.000535
TXNDC17	14.71074	32.90455	2.23677	0.005189
FBXW5	21.90601	49.03262	2.238318	0.000152
RTL8B	9.47279	21.20472	2.238487	0.007643
AMPH	1.762198	3.946744	2.239671	0.001314
NPTN	22.03052	49.37292	2.241115	0.000337
PRR7	1.622055	3.635434	2.241252	0.004716
CDK2AP2	20.38071	45.69127	2.241888	2.47E-05
UBXN6	28.13575	63.09001	2.242344	9.58E-06
MPV17L2	13.30099	29.82539	2.242344	0.009628
ZNF524	4.090612	9.17985	2.244126	0.000638
AURKA	25.85237	58.02909	2.244633	0.006803
LRRC32	0.272212	0.611146	2.245113	0.007082
AP5Z1	1.853691	4.16289	2.24573	0.027892
ABCF3	9.839633	22.1094	2.246974	0.002933
NTAN1	32.5834	73.24079	2.247795	0.0014
PSENEN	32.85782	73.94947	2.250589	0.000175
ACVR1	3.980158	8.959706	2.251093	0.00471
VCAN	10.14239	22.83728	2.251666	0.01406
LAMTOR1	26.19801	58.99919	2.252049	0.002909
BIRC2	11.44112	25.80928	2.255834	0.126148
FBXO25	4.007113	9.040023	2.255994	0.00038
IGFLR1	2.581527	5.824183	2.2561	3.62E-05
NRBP1	25.18931	56.83789	2.256429	0.001023
LOC10537	0.485916	1.096718	2.257012	0.009525
BIN1	12.13214	27.391	2.257721	1.11E-05
MRPL14	29.63415	66.90569	2.257722	0.000319
EIF6	85.93503	194.0475	2.258072	0.001352
CHMP2A	46.27445	104.5676	2.259727	0.001117
PCDHB15	0.314341	0.710514	2.260326	0.018722
IGF2BP3	8.290479	18.74427	2.260939	0.025233
BCL7B	17.01648	38.49559	2.262253	0.001284
RCN3	4.648683	10.52019	2.263047	0.006013
TMX2	40.72455	92.25452	2.265329	0.008853
ALDOC	16.90821	38.32481	2.266639	0.062396
PSMC4	50.65705	114.8916	2.268028	0.002484
EFNB2	2.359047	5.35219	2.268794	0.008552

CLTA	95.9549	217.8374	2.270207	0.003573
GNB1	71.62775	162.7421	2.272054	3.93E-05
LLPH	22.09806	50.22091	2.272639	8.23E-05
OSTF1	17.51298	39.80283	2.272761	0.001681
IL36G	0.047364	0.107696	2.273813	0.401468
PGD	38.35129	87.2044	2.273832	2.38E-05
ITGA1	1.704995	3.878031	2.274511	0.001793
ZNF547	0.453992	1.032849	2.275036	0.167852
ATAD3A	14.60122	33.23432	2.276134	0.002
GPAT3	4.622007	10.52177	2.276452	0.004311
CLP1	6.194587	14.10302	2.276669	0.007486
SH3BGR	0.269562	0.613802	2.277033	0.04793
HLA-DMA	12.29635	27.9997	2.277074	0.037499
PACS1	8.017975	18.26218	2.277655	0.001483
PHF23	12.00043	27.37079	2.280816	0.003531
ATP8B1	0.19727	0.450121	2.281752	0.202538
ZNF837	0.579203	1.322402	2.28314	0.023114
ACTC1	0.024954	0.057027	2.285249	0.478354
ABHD12	15.78234	36.07053	2.2855	0.000926
PDIA3	158.4338	362.2022	2.286143	3.08E-05
ZMYND8	3.361648	7.689496	2.287418	0.003665
GNG2	1.57853	3.613674	2.289266	0.024231
MPRIP	12.24367	28.02957	2.289311	0.009777
UCHL1	154.0603	352.6932	2.28932	0.011288
PCYT1B	0.849043	1.94437	2.290073	0.015668
SHBG	0.096769	0.221701	2.291026	0.056145
TOLLIP	8.334708	19.10036	2.291664	0.004908
FBP1	0.031079	0.07132	2.294793	0.543098
HERC4	7.69103	17.65045	2.294939	0.033976
FGF5	0.327312	0.751349	2.295515	0.000924
AIM2	2.328633	5.345804	2.295684	0.000946
SMIM12	2.389211	5.489449	2.297599	0.000262
ZC3H12C	0.513381	1.179756	2.298012	0.009695
LOC10272	0.071055	0.163404	2.29968	0.185513
CCDC86	24.56953	56.56237	2.302135	0.001861
LOC10798	0.059485	0.136971	2.302623	0.435269
TLN1	28.87314	66.48423	2.302632	0.000194
PLEKHM1	2.276803	5.243137	2.30285	0.001331
C9orf43	0.162414	0.374118	2.303486	0.16458
MED7	4.916739	11.32887	2.304143	0.00981
DDOST	117.1227	269.9069	2.30448	3.37E-05
COPS9	15.14792	34.94268	2.306764	0.001256
C5AR1	0.470001	1.084239	2.306885	0.232819
LOC72839	14.00853	32.37195	2.310874	0.002874
PLEKHA3	1.002768	2.317465	2.311068	0.003486
PCYT1A	8.040079	18.59542	2.31284	0.000272
GNLY	0.07076	0.163743	2.314051	0.433934
SMPD3	0.015345	0.035513	2.314281	0.074724
SLC35F3	0.032366	0.074929	2.315073	0.269395
PLGRKT	12.2656	28.39813	2.315266	0.005679
ARHGAP3	9.801752	22.69689	2.315595	0.001767
TADA3	25.20266	58.37117	2.316072	0.00017
MYO9B	8.035425	18.64067	2.319811	0.000497
VLDLR	7.060766	16.39135	2.321468	0.00187
ASTN2	0.678934	1.576493	2.322012	0.012433
CDC20B	0.049567	0.115111	2.322358	0.014602
MACF1	3.983136	9.251609	2.322695	0.003896
TWIST1	10.03665	23.32197	2.323681	0.009132

COQ2	3.040189	7.064822	2.32381	0.010847
PDCD6	30.9055	71.83089	2.32421	0.008058
STC2	44.21894	102.8827	2.326665	0.000946
TMEM109	43.04919	100.164	2.326733	1.06E-05
RALB	15.43705	35.94445	2.328453	0.000476
ALOX5AP	0.039772	0.09263	2.329011	0.627753
RNASE1	0.659638	1.536838	2.32982	0.15811
BIRC3	0.629453	1.46796	2.33212	0.009917
TMEM120	9.178362	21.40848	2.332495	0.001252
TWF2	32.62892	76.10822	2.332539	0.000156
OSCP1	4.05504	9.45889	2.332626	0.008815
WDR81	1.014495	2.367991	2.334159	0.022234
C6orf223	0.00886	0.020683	2.334454	0.013331
POGLUT1	2.428698	5.672005	2.335409	0.006062
UGDH	4.144636	9.688118	2.337508	0.000963
ZMPSTE24	14.75678	34.49766	2.33775	0.015938
SRGAP1	0.855897	2.001091	2.338006	0.001389
GPX8	17.95586	41.99688	2.338896	0.001976
PAFAH2	2.109621	4.935233	2.339394	0.008604
LYSMD2	1.94098	4.545302	2.341756	0.004715
GTF2H1	9.15225	21.43667	2.34223	0.008168
TMEM138	5.052619	11.85412	2.346133	0.004401
ING2	5.121947	12.04124	2.35091	0.004755
ATG5	11.23737	26.44742	2.353523	0.000555
AGFG2	1.77022	4.166757	2.353808	0.000393
COL22A1	0.368465	0.867429	2.35417	0.030007
SNX14	9.73102	22.92727	2.356102	0.000326
NSDHL	16.16987	38.13258	2.358249	0.002793
BTBD19	0.399077	0.941389	2.358914	0.031512
MBNL1	6.591411	15.5523	2.359479	0.003727
TSPAN17	14.85393	35.07886	2.361588	0.000342
EIF4G2	91.00712	214.9673	2.362093	0.000615
SLC38A5	0.201756	0.476972	2.364106	0.175771
BRD3OS	6.659972	15.77085	2.368006	0.008922
TNFRSF10	0.92885	2.200247	2.368786	0.012617
C11orf94	0.355481	0.842376	2.369679	0.126951
SIX5	3.764659	8.922585	2.370091	0.001172
MAPK7	4.18193	9.916097	2.371177	0.001811
RABL3	3.58315	8.519146	2.377558	0.001704
C11orf68	25.15958	59.83547	2.378238	0.000797
PPP1R18	10.45419	24.86594	2.378562	0.003204
CCDC89	0.31001	0.737825	2.380002	0.073476
FKBP2	43.29077	103.0701	2.380879	0.001024
GALR2	0.019417	0.046267	2.38278	0.400983
CENPX	20.38009	48.57863	2.383632	0.002149
PQLC2	1.961027	4.674784	2.383845	0.014828
TBCD	3.288499	7.849931	2.387087	0.002986
SPG21	22.95738	54.83546	2.388577	0.000119
CD53	0.02965	0.070822	2.388593	0.154301
VDAC1	125.4468	299.8753	2.390457	0.003858
SHROOM1	1.225467	2.931301	2.391987	8.74E-05
PQLC3	1.615464	3.865445	2.392776	0.020845
CROT	1.334961	3.195201	2.393478	0.000812
ACPP	1.743261	4.173824	2.394263	0.028421
NSMCE1	26.35613	63.19432	2.397708	0.005033
LAP3	17.28568	41.4578	2.39839	0.005656
PELI3	2.423204	5.81461	2.399554	0.000132
PIP5K1C	4.544713	10.91262	2.401169	0.000534

MRAS	4.30189	10.33462	2.402344	0.000987
GREM2	0.339514	0.81577	2.402755	0.46742
DAPK3	23.18326	55.7262	2.403726	0.00656
TNFRSF18	0.056469	0.135739	2.403769	0.011187
PIGB	3.449535	8.296503	2.405107	0.006825
FAM57A	8.333073	20.0434	2.405283	0.003131
CREB3	24.62622	59.26071	2.406407	0.002695
CHMP6	6.98112	16.80653	2.407426	0.00898
FAM46A	3.641737	8.770258	2.408262	0.003011
SELL	0.145433	0.350287	2.408573	0.072497
FAM3C	14.53919	35.04506	2.410386	0.000324
LOC10536	0.020113	0.048495	2.411147	0.087202
SALL1	5.984336	14.43422	2.412	0.016434
NINJ1	21.01352	50.69386	2.41244	0.051955
CAMK4	1.080559	2.607114	2.412745	0.002575
RMDN2	0.222632	0.537165	2.41279	0.046109
LRRC17	0.249542	0.602273	2.41351	0.053222
CARHSP1	21.36745	51.57148	2.413553	0.000241
HARS	24.39805	58.91673	2.414813	0.000363
LOC11226	0.050069	0.120986	2.416371	0.351212
TIGD2	2.708879	6.553004	2.419083	0.014288
GPR173	0.72701	1.759728	2.420501	0.017826
WASHC3	21.63875	52.40726	2.421918	0.001552
NPR3	0.115076	0.279372	2.42773	0.151288
RORC	0.023611	0.057322	2.427758	0.25675
ACAA1	10.01619	24.32608	2.428677	0.000104
TSSC4	3.629535	8.819233	2.429852	0.001435
MUL1	7.331584	17.81566	2.429988	0.002158
ARPC2	49.93404	121.4324	2.431856	2.17E-05
ADRM1	44.39702	108.0565	2.433867	0.002648
LOC10272	0.046379	0.112896	2.434231	0.025309
COL5A1	12.25258	29.83505	2.435001	0.038937
HCST	0.327899	0.798526	2.435279	0.06087
GNPDA2	4.089213	9.962908	2.436388	0.000342
ARPC1B	28.20745	68.78365	2.438492	0.001514
SYNE3	0.751497	1.835394	2.442316	0.006428
FASTK	26.98559	65.91124	2.44246	0.000786
BOLA2B	1.377746	3.36609	2.443186	0.129875
CXCL5	0.107055	0.261676	2.44431	0.255875
GALE	8.948097	21.89583	2.446981	0.004766
SLC27A4	5.582551	13.66492	2.447792	0.001445
TPM3	22.8899	56.0315	2.44787	0.000154
SAA1	0.784683	1.921314	2.448522	0.101057
TMEM50A	31.49282	77.1127	2.44858	0.001541
TALDO1	91.71248	224.6515	2.44952	0.001767
YWHAB	71.3915	175.0057	2.451352	0.000188
DNASE2	19.79844	48.58851	2.454159	0.00019
CLPTM1	27.36156	67.18207	2.455345	0.007
TNIP2	8.724907	21.42274	2.455355	0.000745
WDR66	0.316783	0.778058	2.456124	0.010101
NECAP2	12.39041	30.43335	2.456201	1.21E-05
FAS	0.585861	1.439199	2.456552	0.001295
DNAJB11	46.19621	113.5484	2.457959	0.001643
FBXO4	0.673627	1.65631	2.458792	0.005267
TNFAIP8L1	2.770087	6.812487	2.459304	0.023531
EMC4	59.56371	146.5274	2.460012	4.05E-05
IRF2	1.07483	2.646636	2.462377	0.000424
NOP10	145.7655	359.1322	2.463768	0.000357

DDX39A	24.16031	59.52767	2.463862	0.00034
MPHOSPH	16.86778	41.56826	2.464358	0.004008
GTF2IRD2	0.74819	1.844463	2.465233	0.01295
PANX1	3.442096	8.486958	2.465636	0.008286
ORAI2	2.619838	6.46009	2.465836	1.66E-05
MYDGF	83.53794	206.2194	2.468571	0.002812
COMMD4	31.7192	78.31472	2.469001	0.000475
RNF17	0.002305	0.005692	2.469674	0.610595
TMEM212	0.00954	0.023559	2.469674	0.610595
LOC10798	0.006309	0.015582	2.469674	0.610595
LOC10798	0.003655	0.009026	2.469674	0.610595
CCDC63	0.006568	0.016222	2.469674	0.610595
IFIT5	2.565364	6.338298	2.47072	0.001153
CDK5	18.40902	45.48906	2.47102	0.0109
DRP2	0.538904	1.3322	2.472054	0.003559
FAM24B	2.240897	5.54202	2.473125	0.005421
PITPNC1	3.773086	9.340273	2.4755	0.05208
C10orf111	0.053893	0.133438	2.475993	0.140084
CCM2	6.956959	17.23429	2.477273	0.000829
UROD	44.09269	109.3472	2.479939	0.001421
COMMD9	7.256014	18.00473	2.481352	0.00233
ATP6V1D	30.4656	75.61386	2.481942	0.006573
STBD1	0.152117	0.377559	2.482029	0.038652
DNTTIP1	30.25853	75.15413	2.483733	0.000662
MGAT5B	0.828473	2.059275	2.485627	0.004448
SLC20A1	36.07696	89.77107	2.488321	0.019823
CCDC92	7.274797	18.11627	2.490278	0.008832
MYO5A	2.824079	7.033493	2.490544	0.000132
SVIP	6.352339	15.8271	2.491538	0.009875
MCFD2	38.70484	96.44236	2.491739	4.91E-05
TAS1R1	0.004551	0.011345	2.493078	0.439638
METTL1	5.19207	12.94978	2.494147	0.001634
URM1	4.903954	12.23797	2.495532	0.001092
RIMBP3	0.074664	0.186335	2.495661	0.075949
PSME1	29.6687	74.05055	2.495915	0.003052
ACTR10	17.66736	44.12965	2.497806	0.001101
PMP22	14.16351	35.38569	2.49837	0.0002
UAP1L1	5.146828	12.85894	2.498421	6.17E-05
AP5S1	2.282699	5.703506	2.49858	0.013111
VCP	65.20279	163.0008	2.499906	0.002103
MAD2L1B	9.882629	24.71244	2.500593	0.005654
CPA2	0.113456	0.283726	2.500753	0.036724
PHTF1	4.995426	12.49798	2.501885	0.004023
SETD9	0.442902	1.108247	2.502238	0.009369
GEM	9.559264	23.92074	2.502362	0.004353
TUBA1C	134.1673	335.8556	2.503261	1.80E-06
BAD	28.38818	71.07563	2.503705	5.27E-05
DNPEP	6.450972	16.15496	2.504268	0.000248
RRAS2	16.21252	40.60543	2.504572	0.009684
MAGEC2	0.151114	0.378489	2.504665	0.007529
UPF3A	4.872392	12.20418	2.504762	2.90E-05
NDUFAF1	8.191507	20.53165	2.506455	0.000288
MAP4K4	12.41769	31.13969	2.507688	0.000232
TIMM21	12.26472	30.76392	2.508326	0.00137
MAEA	10.16947	25.53059	2.510513	0.000178
LARP6	27.92663	70.11721	2.510765	9.48E-05
INTS5	4.77694	12.00577	2.513276	0.001199
TPCN1	7.116727	17.89305	2.514225	0.004918

TOR1B	7.771968	19.54286	2.514532	0.002536
PPP1CA	74.10238	186.3506	2.514772	0.000184
ANXA4	2.837636	7.139123	2.51587	0.003745
ZNF763	0.014785	0.037205	2.516483	0.441074
EPOR	6.221721	15.66199	2.517308	0.00056
HMOX1	29.6348	74.61356	2.517769	0.12057
SPTY2D1C	1.021178	2.571176	2.517853	0.004102
PSME2	59.49103	149.8399	2.518697	0.00024
MTHFD2L	1.061664	2.676716	2.521246	0.002911
ARFRP1	7.059197	17.80183	2.521793	0.000471
SMIM37	3.944438	9.947658	2.521946	0.00018
CCNB1	58.35513	147.2413	2.523194	0.006533
RASSF7	6.540019	16.5043	2.523585	0.011197
LINC0163	0.23399	0.590545	2.523808	0.253854
VPS9D1	2.636133	6.655411	2.524687	0.001427
CPNE2	9.748209	24.61334	2.524908	0.00044
FOXL2	0.006688	0.016907	2.5281	0.201383
LOC10537	0.026221	0.066291	2.528185	0.522998
HECTD3	11.03692	27.90632	2.528451	0.000232
ADAM9	20.65788	52.23641	2.528644	0.00792
LRFN3	4.043481	10.23088	2.530217	0.000587
TRPT1	13.11676	33.19841	2.530992	0.002309
ATP1A2	0.119853	0.303367	2.531162	0.005923
PNPLA3	1.695256	4.293536	2.532676	0.002471
LRP1	13.55927	34.34556	2.532994	0.005265
ZFP36L1	15.14838	38.4032	2.535136	0.023384
DCTD	11.81936	29.98053	2.536562	8.59E-06
STMN4	0.049843	0.126479	2.537564	0.445757
PDLIM7	26.67588	67.75246	2.539839	0.000227
RAB8B	2.005826	5.100157	2.542672	0.001353
NFKBIA	24.34562	61.98979	2.54624	0.026567
LOC10272	0.035748	0.091036	2.546637	0.126576
STAT1	13.74978	35.02546	2.547347	0.000297
DUSP10	1.086341	2.767692	2.54772	0.002831
SLC22A14	0.06067	0.15459	2.548058	0.001478
RTP5	0.017063	0.043483	2.54844	0.585064
LYPD6	0.927243	2.365429	2.551034	0.001559
TMEM62	1.794975	4.579353	2.551207	9.93E-05
ASPHD1	2.841701	7.254919	2.55302	0.00091
IL20	0.022638	0.057834	2.55473	0.480707
MGAT4B	25.20058	64.39981	2.55549	0.008272
ZNF287	0.537863	1.377177	2.560458	0.002182
HIST1H4D	0.156416	0.400664	2.56153	0.238688
TTC9B	0.017023	0.043632	2.563122	0.432198
KLHL10	0.016499	0.042306	2.564221	0.319312
ABR	6.139257	15.74265	2.564259	0.00039
TMEM222	14.32279	36.75355	2.566089	0.005416
OLAH	0.037577	0.096452	2.566778	0.369619
GABRB2	0.005115	0.013129	2.566778	0.369619
DNLZ	6.396687	16.42213	2.567286	0.016547
ANXA9	0.108263	0.27804	2.568186	0.001818
QPCTL	8.442453	21.68246	2.568265	0.000188
MOCS2	9.417386	24.1898	2.568632	0.003427
COMMD5	5.373668	13.83305	2.574228	0.000291
LOC10537	0.014494	0.037317	2.57474	0.599092
RNF24	2.728059	7.025095	2.575125	0.000248
PPP4R1	8.226447	21.20072	2.577141	0.000315
FAM102A	4.916025	12.67027	2.57734	0.001731

RELB	4.86943	12.55243	2.577803	0.013679
FAM32A	33.64277	86.77768	2.579385	0.000265
EMC3	22.40259	57.7901	2.579617	0.003785
DPF1	4.175222	10.77888	2.581631	0.00166
KEAP1	11.33495	29.26754	2.582061	0.005587
PHPT1	46.87404	121.0346	2.582125	0.000933
GNS	16.6049	42.91294	2.584354	0.000308
CDA	3.789099	9.796972	2.585568	0.037351
PSPN	0.686006	1.775123	2.58762	0.015574
TAP1	6.352308	16.4386	2.587815	5.14E-05
SLFN5	2.710649	7.016298	2.58842	0.025484
CLIP2	3.559628	9.214227	2.588536	0.001509
SIGLECL1	0.008089	0.020947	2.589738	0.597532
HTR5A	0.004084	0.010577	2.589738	0.597532
C7orf71	0.003908	0.01012	2.589738	0.597532
LCT	0.002931	0.007591	2.589738	0.597532
EYS	0.009419	0.024398	2.590178	0.444517
RCSD1	0.006913	0.01791	2.590874	0.373877
PDE4B	1.613281	4.180876	2.591537	0.154598
ADAM21	0.126169	0.327036	2.592058	0.017826
CD58	1.724492	4.472666	2.593613	0.001831
MPDU1	20.16644	52.31239	2.594032	0.001171
PLEKHS1	0.043189	0.112235	2.598715	0.057263
PARK7	153.1339	398.2912	2.600934	6.33E-05
LOC10798	0.039124	0.101781	2.601483	0.031364
OLFML2A	1.098884	2.859122	2.60184	0.11467
PLA2G16	11.0448	28.7403	2.602157	0.003431
TM4SF18	0.009655	0.025131	2.602835	0.033057
CC2D2A	1.543399	4.017685	2.60314	0.000508
TLR4	0.167736	0.436646	2.603179	0.02749
IDH1	15.17925	39.52277	2.603737	0.001928
HAS2	0.75634	1.969775	2.604351	0.032091
SAMSN1	0.015278	0.03979	2.604402	0.36935
GON7	9.866357	25.69605	2.604411	0.002608
DPF3	0.410576	1.069841	2.605711	0.012242
BCL2L10	0.028895	0.0753	2.606021	0.157089
SKP1	54.51766	142.2226	2.608744	0.000178
B4GALT7	8.246585	21.52409	2.610062	0.000176
POMGNT1	17.79363	46.45416	2.610718	0.000175
GPC1	8.567902	22.36951	2.61085	0.004904
ALDOA	303.0338	791.1831	2.610874	0.000917
LOC10028	0.185311	0.483909	2.61134	0.025452
COL7A1	10.19559	26.62445	2.611369	0.018503
MMP12	0.039447	0.10306	2.612633	0.47562
PLSCR3	8.024899	20.97196	2.613361	0.001969
ETS2	3.606095	9.425363	2.613732	0.00059
FTCDNL1	0.081247	0.212369	2.613853	0.00305
FN1	131.9923	345.0202	2.613942	0.006622
SLC14A1	0.890977	2.329052	2.614041	0.190044
INSL4	0.03025	0.079082	2.61428	0.42026
PRR18	0.00688	0.017987	2.61428	0.42026
SH2D4B	0.005918	0.015473	2.614796	0.372806
MPL	0.007987	0.020884	2.614883	0.152241
ARHGAP1	11.70216	30.60331	2.615185	0.001068
PDE6A	0.01124	0.029406	2.616163	0.097643
MRPS12	14.05034	36.77165	2.617135	0.001267
LRRC25	0.014659	0.038375	2.617914	0.480139
ONECUT1	0.059826	0.156708	2.619412	0.019346

GIMAP1	0.004164	0.01091	2.620137	0.59443
CIDEA	0.011601	0.030397	2.620137	0.59443
RNF103-C	0.005452	0.014284	2.620137	0.59443
SLC13A2	0.005897	0.015452	2.620137	0.59443
MMP24OS	22.44681	58.81891	2.620368	0.000486
MMP10	0.169222	0.443812	2.622656	0.381012
CCDC169	0.012088	0.031749	2.626463	0.417108
NMBR	0.013023	0.034207	2.626551	0.380299
SGCA	0.058824	0.154518	2.626806	0.215893
DYNLRB2	0.068018	0.178767	2.628217	0.110038
CLIC4	36.28265	95.42582	2.630067	0.001003
IFIT1	0.545904	1.436747	2.631869	0.017885
FDX1L	19.04537	50.13262	2.632273	0.001377
BCAP29	5.347131	14.08494	2.634112	0.00031
LCTL	0.109625	0.288824	2.634651	0.15919
AMIGO2	1.191018	3.139178	2.63571	0.001141
EPGN	0.025983	0.068562	2.638716	0.328975
GADD45G	79.92798	211.0466	2.640459	0.000682
FAM83C	0.017778	0.046948	2.640818	0.375397
TWF1	15.22709	40.22601	2.64174	0.000289
C1R	11.93468	31.54599	2.643221	0.04254
MORC1	0.003971	0.010504	2.644968	0.415687
GLIS1	0.391521	1.035837	2.645672	0.003423
LIMS4	0.086149	0.228074	2.64743	0.069269
DYNLRB1	68.92924	182.4982	2.647617	0.000166
ATG7	2.898273	7.676176	2.648534	0.000604
GSTM4	4.342619	11.50234	2.64871	0.002651
MAGEA4	0.008561	0.022696	2.651005	0.174259
RARG	6.55161	17.37167	2.651512	0.012362
PAQR7	2.34622	6.223933	2.652749	0.000253
POFUT1	13.49148	35.79505	2.65316	0.00034
CHMP4B	77.60592	205.9286	2.653516	0.000256
GYS1	10.79837	28.65688	2.653816	0.002149
MAFF	11.00078	29.20538	2.654845	0.017157
MALRD1	0.001929	0.005127	2.657293	0.412553
PPP1R14B	113.6642	302.194	2.658657	0.000358
GLYCTK	0.386718	1.028548	2.659684	0.020774
LOXL3	8.056568	21.43229	2.660225	0.001677
CDKN2D	11.33644	30.17062	2.661385	0.00301
KDELR3	20.19782	53.77323	2.662329	0.00138
UBE2J2	6.994239	18.62322	2.662651	0.002848
ARHGAP2	0.228601	0.608693	2.662688	0.008406
SNX3	58.44505	155.6288	2.662822	0.000178
SLC5A9	0.002561	0.006821	2.663187	0.420009
SUCNR1	0.008899	0.0237	2.663187	0.420009
USP50	0.013365	0.035593	2.663187	0.420009
GNG13	0.018906	0.050355	2.663364	0.590152
ESRP2	0.01675	0.044648	2.665504	0.300085
BCL3	3.829993	10.20923	2.665601	0.001401
EPB41L4B	1.29961	3.464379	2.665708	0.045549
PPP2R3A	1.110149	2.960362	2.666634	0.001012
HM13	42.27701	112.7768	2.667569	0.008493
ARHGDIA	91.14414	243.2156	2.668472	0.000206
IRX3	5.137315	13.71355	2.669399	0.007686
IL17B	0.020998	0.056052	2.669456	0.501961
UXS1	9.007668	24.06224	2.671306	9.74E-06
CYP19A1	0.106469	0.28471	2.674109	0.22307
HSPA1L	0.236945	0.63366	2.674288	0.006969

NDRG1	49.5056	132.3936	2.674315	0.046646
C1S	7.154345	19.14083	2.675413	0.006692
SLC49A3	0.003509	0.009388	2.675547	0.412682
IL1RL2	0.003543	0.009479	2.675547	0.412682
SYCE1L	2.93177	7.844409	2.675657	0.010327
ABHD15	2.416229	6.465762	2.675973	0.001465
ABHD5	3.232876	8.657241	2.677876	4.29E-05
HPS1	6.287296	16.85081	2.680137	0.000665
TRIB2	3.331094	8.929376	2.680614	0.001065
IDUA	1.510355	4.050896	2.682082	0.001118
GSTA2	0.012931	0.034683	2.682123	0.168014
SAPCD2	5.640833	15.13114	2.68243	0.018713
GDNF	2.722742	7.304123	2.682635	0.008042
P3H3	14.34285	38.50878	2.684877	0.000938
C8orf82	6.223699	16.71304	2.685386	0.000341
CRIP1	0.429743	1.154199	2.685789	0.180377
HSD11B1	0.495816	1.332843	2.688179	0.001527
RAB3B	2.772188	7.452661	2.688368	0.000989
KRT19	0.025001	0.067211	2.688376	0.371884
SCAND1	19.39521	52.1541	2.68902	0.000597
ZNF668	2.09539	5.63661	2.690006	0.00125
PAQR5	2.868424	7.717802	2.690607	0.000318
PIANP	1.536538	4.134893	2.691045	0.010665
TBC1D17	9.295444	25.04321	2.694139	0.001137
CENPB	15.38583	41.495	2.696962	0.005927
SYCP3	0.023532	0.063472	2.697285	0.303402
TNFRSF21	7.906274	21.32847	2.697665	0.000509
ECSCR	0.035464	0.09578	2.700791	0.498264
KIAA1549I	1.031216	2.788209	2.703808	0.003431
FTSJ1	24.65386	66.73016	2.706682	0.000533
SPANXD	0.026572	0.07193	2.706953	0.408268
MRPS18A	17.09445	46.27683	2.707126	0.000592
RTN4	31.42689	85.14871	2.709422	4.16E-05
RAB11B	29.6686	80.42973	2.710938	0.000518
TGFB1	22.11591	59.98337	2.712227	0.000853
LDLR	7.992832	21.67912	2.712321	0.043232
PDE7B	0.104178	0.282775	2.714333	0.115747
STAT6	5.754098	15.62208	2.714949	0.000397
MRTO4	16.38932	44.52416	2.716658	0.000914
TBC1D22A	1.631732	4.434037	2.71738	0.000456
ORAI3	4.719541	12.82768	2.717992	0.002857
BEST4	0.042467	0.11548	2.719307	0.129173
SLC9A8	2.041617	5.554029	2.720407	0.002507
RETREG1	1.66545	4.537086	2.72424	0.001076
SSU72	55.58066	151.514	2.72602	6.08E-05
HRASLS	1.314882	3.585965	2.727215	0.002264
JKAMP	12.45322	33.97186	2.727958	0.00403
C7orf25	2.089704	5.705247	2.73017	0.010191
RGS13	0.011765	0.032136	2.731604	0.5837
SHC1	18.15473	49.6265	2.733531	4.05E-05
KBTBD4	4.429072	12.11531	2.735407	0.005106
ZMIZ1	5.158351	14.11104	2.735573	0.002601
ZBTB45	2.403016	6.573773	2.735635	0.000711
PCDHGC3	1.622788	4.439457	2.735697	0.00609
HK1	28.87397	79.0782	2.738737	4.41E-05
UBE2F	11.40187	31.24224	2.740097	3.01E-06
GSDME	7.070366	19.37665	2.740544	0.006245
DYNLT3	13.85852	38.03638	2.744621	4.57E-05

BBOF1	0.976452	2.681697	2.746369	0.001093
SEMA4B	6.954311	19.09999	2.746497	0.015952
RUSC2	3.959539	10.87726	2.747103	0.009748
MRPS15	52.8448	145.2046	2.747755	0.001523
CYB561D2	6.752567	18.55594	2.747983	0.003643
TNNT1	35.5655	97.73676	2.748078	0.00447
SDC1	11.5779	31.81888	2.748241	0.000248
ERAP1	1.509774	4.151809	2.749953	0.008242
PIP4P2	2.577994	7.092861	2.75131	0.003826
RIC8A	18.03312	49.65533	2.753563	1.07E-06
DAP	46.70318	128.7429	2.756621	9.60E-05
CFAP299	0.022384	0.061731	2.757784	0.040204
PCDHGA3	0.020495	0.056622	2.762702	0.210963
MRGPRX3	0.044733	0.123591	2.762888	0.396441
TBXA2R	0.758799	2.098355	2.765364	0.004742
NUDC	43.9955	121.6786	2.765705	8.22E-05
CHST7	3.282476	9.079259	2.765979	0.000335
HLA-A	128.4745	355.4753	2.766894	0.000863
SAP30	17.49244	48.40016	2.766919	0.006577
CRAT	8.153039	22.56986	2.768276	8.77E-05
SLC2A4RG	28.18601	78.06049	2.769477	0.008071
AK4	9.099007	25.20218	2.769772	0.02377
HNRNPM	35.80883	99.2908	2.772802	0.001031
NRIP3	6.66423	18.48054	2.773094	0.000194
LACTB	5.234979	14.51764	2.7732	0.000603
CRYZ	13.41502	37.23117	2.775336	7.53E-05
GSPT2	3.194449	8.870702	2.776911	0.015869
NDUFB2	103.5345	287.5497	2.777332	0.000189
ELL2	7.997798	22.23574	2.780233	0.004128
PIEZO1	9.296225	25.84936	2.78063	0.001326
PTPA	17.50768	48.68344	2.780691	0.000572
SERF2	56.89266	158.352	2.783347	2.26E-05
PARP9	0.956499	2.663371	2.784499	0.003952
PLBD2	12.60581	35.12117	2.78611	0.000827
ACSS2	6.906567	19.25028	2.787243	0.000187
ITGB1BP1	10.97018	30.58615	2.788118	0.000189
VASP	17.34681	48.42157	2.791382	6.84E-06
CASC4	9.477922	26.48292	2.794169	0.00145
LENG9	1.084282	3.030078	2.794548	0.015149
FAM49A	0.607732	1.69875	2.795229	0.007134
FAAP24	3.239321	9.063266	2.797891	0.008761
LRRN4CL	0.166327	0.465433	2.798303	0.123908
GRN	41.44358	115.988	2.798695	0.000995
CPTP	5.557547	15.56537	2.800763	0.000661
ADAM8	1.319881	3.697956	2.801734	0.016895
ASPH	10.28402	28.83103	2.80348	0.003498
LOC10537	0.138043	0.387285	2.80554	0.048378
NUDT18	1.439054	4.040298	2.807606	0.050107
MYADM	17.36465	48.75815	2.807897	0.001946
MDFIC	2.288634	6.426876	2.808172	0.000375
GAPDH	1816.743	5104.781	2.809854	0.002084
ZNF688	1.825554	5.129578	2.809875	0.002835
TEX30	5.359072	15.05897	2.809996	0.000878
NEDD4	3.468927	9.754319	2.811913	0.00073
RAB5IF	12.4218	34.97191	2.815365	0.005856
PFN1	218.6576	615.6702	2.815681	0.000431
SDHAF2	18.34268	51.66417	2.81661	0.003225
RTN4IP1	1.67524	4.720554	2.817838	0.003145

DHRSX	0.143981	0.40605	2.820163	0.510705
PXMP4	1.198624	3.38275	2.822195	0.000102
CNDP2	5.461066	15.41779	2.823219	0.00105
KLF8	0.027562	0.077836	2.824063	0.00775
PSMA7	190.0834	536.8953	2.824525	3.03E-05
XKR8	4.369136	12.34215	2.824849	0.00077
TIMM10	17.37121	49.0735	2.82499	0.001592
LOC10537	0.012193	0.034499	2.829392	0.330413
PPP1R12C	14.0145	39.66772	2.830477	1.27E-05
JAKMIP2	0.763001	2.160974	2.832202	0.004963
HACD1	10.22351	28.95874	2.832564	0.000236
VSTM4	0.863618	2.447476	2.83398	0.0002
SYTL3	0.850439	2.411008	2.835017	0.009025
CORO1C	28.95342	82.11652	2.83616	0.001109
ZCWPW2	0.135245	0.383655	2.836751	0.002218
LYL1	0.04966	0.141032	2.839973	0.050187
AVIL	0.027924	0.079362	2.842026	0.159438
KCNAB1	0.017385	0.04942	2.842645	0.035314
KIAA2013	13.38283	38.05796	2.84379	1.88E-05
AES	80.6108	229.2984	2.844512	8.94E-05
RTF2	47.01168	133.822	2.846569	2.69E-05
LSS	7.228196	20.57771	2.846867	0.004075
MTMR4	6.626222	18.86493	2.847011	0.034027
ITPRIPL2	3.193658	9.095769	2.848072	0.008097
CHI3L2	2.711688	7.72507	2.848805	0.003755
UNC93B1	6.297295	17.94241	2.849226	0.000835
DYNLL1	171.506	488.9525	2.850936	3.71E-05
CAPN1	18.89041	53.85708	2.851028	5.69E-06
NRROS	0.234913	0.670157	2.852784	0.125426
KIF1C	9.61831	27.44403	2.853311	4.54E-05
TESK1	8.375323	23.89804	2.853387	0.000851
PHC2	24.74231	70.60071	2.853441	0.000271
CALM1	36.50268	104.1687	2.853728	0.000923
PRKAR1B	0.778993	2.226955	2.858761	0.001041
GBA	10.64282	30.43738	2.859899	0.01788
GNG12	18.00794	51.54098	2.862126	0.000182
VEGFB	21.33745	61.12199	2.864541	0.00211
PFN2	75.85957	217.3383	2.865009	0.000114
CKAP4	45.86882	131.4148	2.865013	5.74E-06
GJA1	25.42381	72.85614	2.865665	0.010084
3-Mar	0.59572	1.707253	2.865864	0.014277
ADAM32	0.070047	0.200792	2.86653	0.040233
CUL4A	5.781018	16.58094	2.86817	9.79E-05
EVC	2.464807	7.069651	2.868237	0.000209
TTC25	0.962372	2.762026	2.870018	0.001863
TGFA	5.191846	14.91802	2.873356	0.037533
TMEM104	1.475069	4.242487	2.876128	0.003293
PYROXD2	0.499938	1.441194	2.882747	0.022741
QSOX1	21.34213	61.5925	2.885959	0.001379
LOC10537	0.143767	0.41571	2.89156	0.003773
CAP1	55.48512	160.7766	2.897652	1.58E-05
CHPF2	14.58259	42.2786	2.899251	0.007387
LAMTOR5	61.22306	177.6558	2.901779	6.20E-05
TNNC2	0.244253	0.709556	2.905006	0.10676
PCYOX1	7.909485	22.98659	2.906205	6.32E-05
ADGRA3	4.678829	13.59822	2.906329	3.42E-05
PDE4C	0.436964	1.2706	2.90779	0.000661
TTN	0.001596	0.00464	2.908091	0.027448

SNX33	3.87479	11.27143	2.908914	0.001066
DKK3	6.086766	17.70818	2.909293	0.01563
LOC10537	0.199159	0.579462	2.90955	0.072609
GRK5	4.965689	14.45051	2.910072	6.12E-05
ZNF487	0.552386	1.608537	2.91198	0.004414
HECW1	0.108413	0.315698	2.911998	0.004252
MAN1B1	10.43016	30.38147	2.912847	2.33E-05
UGCG	6.357151	18.51843	2.913007	0.057112
UBE2E3	9.39912	27.44564	2.920022	0.000145
PSMC1	36.95499	107.956	2.921284	0.000185
FAM83D	7.460819	21.80488	2.922585	6.69E-05
RND3	19.50204	57.00791	2.923178	0.020842
RAI14	12.65523	37.00383	2.923995	0.001825
PCDHGA7	0.018866	0.055183	2.925078	0.140937
NOTCH4	0.111756	0.32713	2.927178	0.007471
ANKRD13.	10.0735	29.50328	2.9288	0.006546
CIART	1.87351	5.487197	2.928833	0.004729
LRP8	3.133914	9.180633	2.929446	0.001315
DHDDS	4.501713	13.20663	2.93369	0.000973
AHR	3.529596	10.36097	2.935455	0.000669
TBX2	5.731017	16.83252	2.937091	0.002125
PLA2G15	6.484278	19.04824	2.937604	0.000383
GPX1	86.92376	255.3581	2.937725	0.000199
IMP3	27.4684	80.72794	2.938939	0.002845
EDEM2	0.223873	0.659286	2.94491	0.05254
KLF2	1.392896	4.102455	2.945269	0.004925
SAA2	0.023665	0.069777	2.948479	0.291705
MYL12B	121.7234	358.9591	2.948972	8.28E-06
TOR1A	14.44474	42.59825	2.94905	0.00259
UBE2L5	0.013834	0.040805	2.949662	0.550163
SERP2	1.609326	4.747903	2.950243	0.010086
BDH1	0.084906	0.250577	2.951241	4.64E-05
FCRLA	1.816812	5.362155	2.951409	0.218694
C4orf3	21.84792	64.51332	2.952835	0.006978
PSMB7	149.3984	441.2085	2.953235	0.000295
LHFPL2	4.043621	11.947	2.95453	0.00286
PTCHD4	0.040438	0.119539	2.956123	0.032571
ATP1B3	67.40824	199.2703	2.956171	0.006836
GAS6	11.9024	35.26569	2.962905	0.018925
NIM1K	1.018578	3.018097	2.96305	0.002639
PIGT	45.14279	133.8563	2.965175	2.08E-06
DNAAF4	1.592255	4.721443	2.965256	0.006582
MICA	7.476575	22.22798	2.973016	0.006636
POLR2J2	0.438012	1.302719	2.974159	0.012292
SLC38A6	1.656248	4.92637	2.974415	0.001216
ANKRD45	0.03763	0.111948	2.974966	0.019652
ZNHIT2	3.814105	11.3493	2.975613	0.012102
ZMYND11	2.980347	8.868694	2.975725	0.000656
RAB7B	0.344187	1.024394	2.976269	0.00427
PLK2	5.717577	17.03858	2.980035	0.043401
IRF2BPL	8.781804	26.18003	2.981167	0.005514
ZNF395	8.575881	25.59984	2.985098	0.090244
NSMAF	5.181807	15.47348	2.986117	5.03E-05
STAG1	3.12931	9.352792	2.988772	0.001816
DTX3L	1.878257	5.619286	2.991756	0.001713
MOCS3	2.00975	6.024177	2.997475	0.002877
POLR3H	6.37324	19.10478	2.997655	0.001155
ANXA6	38.8756	116.5411	2.997796	0.007537

ZBTB4	4.42467	13.28163	3.001722	6.10E-05
LOC10099	0.036721	0.110259	3.002606	0.283459
CHMP1A	16.2568	48.81718	3.002878	4.32E-06
PDE4DIP	2.612395	7.846121	3.003421	0.000297
POP4	10.14848	30.48688	3.004083	0.000195
PSMC3	84.30488	253.2647	3.004152	3.65E-05
LOC10798	0.267269	0.803192	3.005179	0.120041
PEPD	11.26383	33.90907	3.01044	0.00015
LASP1	29.75731	89.63589	3.012231	0.000307
CISD3	5.525975	16.6592	3.014708	4.59E-05
TTPAL	1.986905	5.997032	3.018278	0.00023
E2F7	4.284284	12.93695	3.019629	0.001429
NCS1	12.53031	37.87397	3.022589	0.000472
SCN1B	0.789288	2.386054	3.023046	0.007136
ACP2	7.645275	23.12336	3.024529	0.010939
ADPRH	0.421506	1.275007	3.024884	0.000986
CHRNA10	0.054922	0.166295	3.027829	0.028827
TANGO2	2.57566	7.812774	3.03331	0.000736
ZNF596	0.157919	0.479161	3.034224	0.016451
SLC39A13	11.2123	34.02688	3.034781	0.000559
HRAS	22.79294	69.20806	3.036381	1.13E-05
GM2A	9.878422	30.0142	3.03836	0.000484
WDR34	30.06976	91.42508	3.040433	0.00053
ERMN	0.044989	0.136894	3.042817	0.176247
TMEM127	4.337742	13.21308	3.046072	0.000486
C9orf153	0.057681	0.175848	3.048631	0.125489
TRIM8	12.01668	36.6618	3.050911	6.53E-06
STK17A	9.670873	29.56802	3.057431	0.000213
ACSL4	9.065447	27.72667	3.0585	0.001237
TP53INP2	5.121265	15.66417	3.058652	0.000334
TMEM199	4.36444	13.36136	3.061415	0.001488
BOLA2-SM	0.667305	2.043412	3.062184	0.02175
FZD1	2.994759	9.170899	3.062316	0.004769
FHOD1	8.39753	25.74296	3.065539	0.000139
TRMT61A	3.519105	10.79199	3.066687	0.005214
G6PD	29.85077	91.56901	3.067559	1.58E-05
CDKN3	26.0343	79.87366	3.068016	0.001906
METRNL	7.934487	24.35699	3.069763	0.006143
TENM4	0.296562	0.910701	3.070861	0.008941
DBI	48.63235	149.4477	3.073009	0.002805
FNTB	0.047724	0.146711	3.074161	0.167231
C14orf178	0.057597	0.177194	3.076448	0.327586
TIPARP	8.609668	26.48857	3.076607	0.072059
HEXB	52.51874	161.6657	3.078248	4.04E-05
CCDC24	2.052239	6.325911	3.082443	0.005256
GPR137	6.532666	20.14043	3.083034	0.000809
ETV4	4.30856	13.28403	3.083172	0.000588
PCDHB7	0.362698	1.118479	3.083774	0.006614
WFS1	4.890729	15.08936	3.085299	0.000406
PLXNB3	1.098777	3.390834	3.086006	0.006287
RER1	16.15206	49.87257	3.08769	6.45E-05
ZNF792	0.954621	2.948345	3.088497	0.002266
GSTO1	60.92988	188.3725	3.091627	0.000797
ATP6V0D1	22.04085	68.17645	3.093186	0.000782
HSPB11	44.37629	137.4815	3.098084	4.79E-05
PHLDA2	9.436459	29.23532	3.098124	9.13E-05
PLCB3	6.176817	19.14401	3.099333	0.000182
KCNIP4	0.008171	0.02537	3.105044	0.017459

GBP3	0.21259	0.660114	3.105105	0.013208
PCDHA10	0.01035	0.032142	3.105432	0.231505
IRF5	0.061573	0.191298	3.106858	0.090726
CRIM1	2.510213	7.799201	3.106987	0.000684
CNTF	0.259705	0.807102	3.107762	0.013055
TWSG1	5.013354	15.58493	3.108683	0.000465
NCOA5	4.821106	14.98873	3.108982	0.00232
TPI1	365.0868	1135.28	3.109616	0.004808
TFRC	15.09735	46.99473	3.112781	0.000117
ITPKA	2.349281	7.317022	3.114579	0.000411
FAM86C1	1.507314	4.695959	3.115448	0.007811
IFITM3	22.08893	68.82173	3.115666	0.007009
PRKAG2	2.452724	7.642861	3.11607	0.002012
NAT1	0.480957	1.499284	3.117293	0.002706
SNAPC2	11.93196	37.21254	3.118727	0.000879
RHOBTB2	1.019857	3.181649	3.119701	0.000307
NMNAT1	1.308263	4.082202	3.120323	0.001104
PLCH2	0.0166	0.051819	3.121649	0.039883
C9orf116	4.062319	12.68364	3.122266	0.004428
CDH11	0.485469	1.515863	3.122472	0.097974
IL15	0.744317	2.328256	3.128043	0.004959
GPR3	1.446164	4.52446	3.128593	0.001214
MGAT1	14.81765	46.36417	3.128983	0.000758
P4HA2	21.56897	67.56559	3.132537	0.001388
PDGFD	0.084618	0.265114	3.133056	0.087875
TMBIM4	8.325877	26.11305	3.136373	0.00131
NDUFB7	30.97724	97.17826	3.137086	0.000399
CORO1B	17.50708	54.92804	3.137475	1.65E-05
RUNX2	1.380029	4.330794	3.138191	0.001283
PCDHGA1	0.109744	0.344516	3.139258	0.042257
ATOX1	65.2529	204.9492	3.140844	0.001241
DPP9	8.161173	25.6372	3.141362	8.52E-06
FGF22	0.146071	0.459145	3.143298	0.047689
TMEM44	4.599302	14.46137	3.144254	0.000483
FAAP20	3.183146	10.01435	3.146055	2.69E-05
CNN2	20.8025	65.4922	3.148285	0.001595
CALR	453.3281	1427.841	3.149685	1.07E-05
CALB2	1.331423	4.194933	3.150714	0.026586
TMEFF2	0.377661	1.189997	3.150963	0.043899
MET	7.389395	23.28829	3.151583	9.69E-05
ABHD8	6.091319	19.20755	3.153266	0.000391
PLOD2	32.37201	102.0841	3.153468	0.003446
EBF2	1.532375	4.834132	3.154666	0.001567
GBP4	0.052539	0.166005	3.159639	0.02671
MYO1C	15.27978	48.29057	3.160424	0.000422
PCDHB4	0.053713	0.169822	3.161645	0.011632
TTC1	28.40481	89.86981	3.163894	3.93E-05
AMIGO3	1.151765	3.647577	3.166946	0.011335
NUDT4B	0.01542	0.048836	3.167013	0.326955
BNIP3L	22.6329	71.75774	3.170505	0.005061
PNPLA2	21.12009	67.02215	3.173383	0.003238
DOK1	3.258556	10.34776	3.175568	0.000325
PGK1	233.9886	743.5616	3.177768	0.01219
BID	8.51079	27.05389	3.178776	0.001274
TMEM239	0.01307	0.041603	3.183168	0.041281
ZBTB7C	0.194324	0.618892	3.18485	0.004469
SLC4A8	0.329113	1.048651	3.186288	0.002748
KCNK12	0.039801	0.126851	3.187155	0.141262

TMEM9B	9.070944	28.93526	3.189884	2.37E-05
MBD2	6.949957	22.2027	3.194653	1.29E-05
COL6A1	54.1651	173.1076	3.195926	0.010986
PLPPR2	12.9903	41.54495	3.198151	0.009155
PDLIM2	1.760594	5.635382	3.20084	0.002396
PCBP3	0.698555	2.238111	3.203916	0.00063
CCDC102/	1.23733	3.96764	3.206615	0.000588
PLPP3	5.09147	16.33114	3.207549	0.00179
LAMA4	9.037384	29.00531	3.209481	0.036281
FGR	0.017085	0.054919	3.214391	0.350891
RNF39	0.007983	0.025667	3.215434	0.048984
USB1	8.205572	26.4164	3.219325	1.65E-05
CLIC1	88.79825	285.9023	3.219684	0.00018
IRAK2	4.261336	13.72129	3.21995	0.0522
NAGK	7.233574	23.34385	3.227154	4.05E-05
DTWD1	1.437756	4.640928	3.227897	0.000554
ADAMTS6	0.368108	1.188405	3.228412	0.003363
SOCS5	3.898296	12.58678	3.22879	0.00012
CFAP161	0.026857	0.086778	3.231071	0.221175
MED14OS	0.16816	0.543801	3.233825	0.014189
RHOG	13.9993	45.34102	3.238806	0.000137
PRDM1	0.239004	0.775869	3.246261	0.045678
LOC10192	0.092838	0.301887	3.251755	0.04747
RSU1	11.68465	38.0261	3.254363	0.000111
TMEM171	8.194149	26.69616	3.257954	0.000294
CD300C	0.060568	0.197398	3.259123	0.097429
TAF13	30.60565	99.83523	3.261987	0.025254
NPY4R	0.01132	0.03697	3.265935	0.016826
FMNL3	1.414633	4.621661	3.267039	0.004371
SIRPA	3.826533	12.51079	3.269485	5.33E-05
SYNJ2	3.57179	11.67799	3.269505	0.000477
SLC38A7	1.500921	4.908519	3.270338	0.001624
TRAK2	3.494123	11.43942	3.273905	0.001067
MX1	0.070008	0.229501	3.278193	0.011534
TCTEX1D2	13.09479	42.97769	3.282045	0.000678
DNAJC30	2.251903	7.396243	3.284441	0.011965
DHX58	0.328632	1.079739	3.28556	0.000776
NR0B1	0.392361	1.289204	3.285757	0.026358
ALCAM	11.99489	39.41675	3.28613	0.008419
ACTG1	702.5802	2309.385	3.287005	0.001354
PTGDR	0.004587	0.015104	3.292899	0.541698
LRRC27	0.343667	1.133415	3.298007	0.00093
EID1	51.40771	169.7388	3.301816	0.000815
EPS8L1	1.15409	3.811246	3.302382	0.001732
CDCA2	2.831143	9.354837	3.304262	0.001354
CUEDC1	4.508429	14.90884	3.306882	0.000196
PRNP	17.13302	56.66415	3.307307	0.01087
ABCB11	0.003467	0.011497	3.316303	0.387835
MAGED4	0.471798	1.566467	3.320208	0.460524
TRIM21	4.233648	14.06491	3.322172	5.65E-05
TRUB2	7.084396	23.53684	3.32235	0.001771
CUZD1	0.115001	0.382079	3.322386	0.086702
ZBTB7A	3.484166	11.58349	3.324608	0.000232
FAH	19.09632	63.49802	3.325144	0.000209
ANKS4B	0.006713	0.022341	3.327921	0.385355
ETHE1	15.87577	52.86218	3.32974	0.000619
LHFPL6	5.708455	19.01927	3.331772	0.001643
RTL5	0.83792	2.794357	3.334874	0.004494

LOC10099	0.091307	0.305089	3.341344	0.244523
KLHL21	6.097016	20.42703	3.350332	0.011533
VSX1	0.017736	0.059427	3.350667	0.237257
FAM218A	0.008932	0.029935	3.351325	0.139374
CAPZB	27.89481	93.55001	3.35367	2.93E-06
ALS2CR12	0.029113	0.097704	3.356025	0.256399
GLTP	13.46638	45.19373	3.356042	0.000142
FANK1	0.357708	1.200868	3.357116	0.001098
DEFB134	0.021079	0.070764	3.357134	0.186087
IL20RB	0.442976	1.487154	3.35719	0.014414
PTRH1	3.46399	11.63153	3.357841	0.009507
TPRG1L	5.377207	18.06061	3.358733	0.002498
SOGA1	2.745888	9.225624	3.359796	0.000144
MRPS6	29.66854	99.76376	3.362611	7.89E-06
APOL4	0.005637	0.018956	3.362943	0.293842
RGMB	5.112526	17.20543	3.365348	0.057452
B4GALNT1	2.548021	8.583905	3.368852	0.001857
ILK	34.76514	117.2087	3.371443	1.77E-05
CIB1	33.25014	112.1157	3.371887	1.09E-06
AARD	0.337086	1.140028	3.382013	0.001469
EDNRA	0.745057	2.523165	3.38654	0.00124
GIMAP2	0.117464	0.398534	3.392817	5.60E-06
PCDHB8	0.425019	1.442432	3.393809	4.02E-05
SERPINC1	0.032591	0.110678	3.396005	0.158851
GNB5	3.494905	11.87407	3.397536	8.93E-06
AK5	0.630616	2.142813	3.397968	0.003336
ITIH3	0.006089	0.02069	3.398134	0.383274
SLC25A52	0.016463	0.055942	3.398134	0.383274
RAB13	61.48702	209.2815	3.403669	0.001707
ZNF175	3.473981	11.84026	3.408268	0.007013
ADAM10	11.99637	40.8914	3.408649	3.04E-05
SLC9A1	3.281485	11.20605	3.414932	0.000679
L3HYPDH	2.074879	7.086303	3.415284	0.000255
TMEM14A	13.89964	47.48124	3.416004	0.000193
RTL9	0.065081	0.222372	3.416827	0.015343
AP1S1	47.04871	160.7912	3.417548	2.18E-05
ERCC1	10.69825	36.57324	3.418618	1.27E-05
ZNF416	0.755334	2.585086	3.42244	0.000274
PARP3	3.366057	11.52282	3.42324	0.000367
CPNE7	3.605076	12.34888	3.425415	0.005208
PDZD11	23.8493	81.75076	3.427805	3.52E-05
SLC22A13	0.013428	0.04604	3.428687	0.10277
ZNF788P	0.128636	0.441485	3.432042	0.013277
HSPA8	321.1269	1103.637	3.436762	0.000128
PIMREG	4.678517	16.10346	3.442	0.000432
IL6ST	9.958694	34.28654	3.442875	3.32E-05
NAPA	31.20446	107.4974	3.444938	0.000323
RELL1	1.575454	5.429011	3.445998	3.23E-05
ISOC2	18.25345	62.92545	3.447318	0.000318
ST6GALNA	0.234268	0.807639	3.447497	0.080207
COMMD8	2.098326	7.240058	3.450396	0.000654
MYH13	0.003105	0.010721	3.452984	0.532481
MYT1L	0.001226	0.004234	3.452984	0.532481
ZMYND10	0.921095	3.181206	3.453723	0.002682
HSPBP1	10.94114	37.82064	3.456737	0.00017
TMC1	0.011245	0.038935	3.462357	0.243831
NRG2	0.463487	1.605382	3.463704	0.002667
TMEM53	1.269336	4.397281	3.464238	0.000414

SYDE1	6.813177	23.66016	3.472706	0.000526
MMRN2	0.221757	0.770615	3.47504	0.016553
FAM71F2	0.034823	0.121027	3.475535	0.185652
RCN1	108.3478	376.7162	3.476916	9.24E-07
LCE2A	0.031425	0.109283	3.477526	0.375907
DPP3	5.016551	17.45889	3.480257	0.000545
SLC38A10	4.335575	15.08893	3.48026	0.005147
BLVRB	46.24859	161.0241	3.481708	0.000284
CETN2	50.14585	174.6142	3.482128	0.000142
REEP5	13.59021	47.32704	3.482437	2.10E-06
CES4A	0.036405	0.12685	3.484414	0.008654
TRIO	4.259859	14.85445	3.487077	0.000985
HIST2H2B	0.105702	0.368705	3.488142	0.055759
RAB4B	8.738065	30.48934	3.489256	6.08E-06
GPR183	0.008661	0.030224	3.489709	0.373463
TNKS1BP1	13.29758	46.44189	3.492506	0.001633
PKP3	0.235358	0.822219	3.493478	0.029797
HIST1H3I	0.038549	0.134673	3.493516	0.530297
SGCZ	0.002551	0.008913	3.493516	0.530297
HCN1	0.001902	0.006644	3.493517	0.530297
CBR1	21.34929	74.85487	3.5062	3.01E-05
TLR2	0.00394	0.013847	3.514251	0.125509
MNS1	3.813596	13.40524	3.515117	0.00127
SQSTM1	61.02103	214.5098	3.515343	0.054211
MYO1E	5.578898	19.612	3.515389	0.000377
ANTXRL	0.003622	0.012743	3.518347	0.373101
IZUMO1R	0.02442	0.085917	3.518347	0.373101
S100A7	0.041886	0.14737	3.518347	0.373101
UMODL1	0.002017	0.007095	3.518347	0.373101
CSGALNA	0.632324	2.225929	3.520233	0.00016
C2orf16	0.005965	0.021013	3.522503	0.24044
TFPI	7.345445	25.87488	3.522575	0.004187
PINK1	20.0067	70.50407	3.524023	3.85E-05
CPN2	0.053254	0.187685	3.524319	0.165231
TMEM191	0.12883	0.454208	3.525652	0.00815
CXCL2	3.850557	13.57633	3.525809	0.064564
ZNF806	0.004068	0.014347	3.526611	0.379077
PPP1R10	3.542024	12.50173	3.529544	0.009825
LRRIQ4	0.007303	0.025783	3.530672	0.370665
PAGE1	0.026231	0.092613	3.530672	0.370665
DOCK5	1.703428	6.017322	3.532479	6.99E-05
MINOS1	4.681337	16.53722	3.532584	0.0004
HAS3	0.272117	0.961454	3.533232	0.043209
CBR3	4.469141	15.79669	3.534615	0.000197
NT5DC2	57.96909	204.952	3.535539	7.10E-05
CDKL4	0.005839	0.020653	3.536924	0.241878
COPRS	39.68041	140.4116	3.538562	3.42E-05
CGB7	0.48508	1.716508	3.538605	0.09132
CDYL2	0.24267	0.858963	3.539628	0.001106
TRANK1	0.050659	0.179345	3.540209	0.01782
HLA-B	82.25692	291.4056	3.542628	0.000435
MRPL17	20.73435	73.47203	3.543493	0.00125
TMEM51	2.040419	7.23568	3.546174	9.45E-05
SLC2A5	0.085943	0.304838	3.546968	0.055852
RRAS	38.35203	136.4572	3.558018	0.003073
AKAP2	0.00518	0.018451	3.561665	0.168944
BRMS1	24.54958	87.43756	3.561673	0.000209
LMO2	0.772945	2.754643	3.563826	0.020241

FEZF1	0.031532	0.112393	3.564367	0.010479
SOCS6	1.53126	5.457992	3.564379	0.000228
FERMT1	0.37524	1.337997	3.565709	0.003896
SPINK4	0.045741	0.163197	3.567828	0.277803
ZBTB47	2.233367	7.968614	3.567982	7.51E-05
PDGFRB	2.499852	8.935396	3.57437	0.001165
DENND2D	0.003852	0.01379	3.580332	0.12828
VAC14	1.58017	5.662294	3.583344	8.09E-05
SLFN11	0.377609	1.35462	3.587361	0.005713
TSPAN8	0.015299	0.054889	3.5877	0.373405
GLIS3	0.821683	2.950141	3.590365	0.000879
GCKR	0.118523	0.425547	3.590418	0.001663
CDK2AP1	46.50462	166.9954	3.590942	0.000663
VSIG10L	0.98208	3.526671	3.591022	0.000188
ERV3-1	0.326793	1.174436	3.593827	0.009657
C9orf3	0.52725	1.896306	3.596599	0.084609
KIAA1324	0.280891	1.011527	3.601137	0.028709
MBOAT7	16.92408	61.00207	3.604454	4.82E-05
MDGA1	0.711433	2.564587	3.604817	0.001191
IYD	0.002348	0.008466	3.604983	0.375564
ANKRD2	0.012089	0.043584	3.605163	0.369093
FAM89B	15.21649	54.86625	3.60571	0.009524
GBP2	2.997814	10.81592	3.607936	3.13E-05
JAM2	1.42003	5.126306	3.609999	0.007591
MORN5	0.12714	0.459157	3.611437	0.022193
KLHL4	0.263598	0.952196	3.612297	0.003947
CTSB	51.0694	184.5467	3.613645	0.000956
GGN	0.161809	0.585022	3.615519	0.008917
ERGIC1	32.01531	115.7722	3.616151	0.001628
EMC7	45.01747	162.9075	3.618762	0.001987
ADGRE5	8.567886	31.0369	3.622469	0.000283
RABEP2	2.579988	9.351677	3.624698	0.003995
NDST1	3.33881	12.12779	3.63237	0.000593
DRAM1	2.198773	7.995771	3.636469	0.001064
IRX5	2.184578	7.952362	3.640227	9.46E-05
PDGFC	5.017132	18.27583	3.642684	1.91E-05
FIBP	16.90833	61.60878	3.643693	4.51E-05
COMMD7	12.20956	44.4915	3.643987	0.000835
ACO1	13.76486	50.16947	3.644751	0.000102
ZSWIM3	0.837501	3.056592	3.649657	0.002411
CALU	73.54215	268.5173	3.651202	0.000167
LOC10798	0.222642	0.81422	3.657082	0.01005
PCDHB9	0.217858	0.798696	3.666122	0.001943
ZNFX1	3.925092	14.39806	3.668211	0.00061
TTC30A	0.561169	2.061403	3.673407	0.002636
RASSF4	2.769025	10.18042	3.676537	0.047194
IRF1	3.47724	12.80427	3.682307	0.000165
MAP6	0.514845	1.896115	3.682883	0.000105
ZDHHC12	15.05885	55.51118	3.686283	0.001061
SPRED1	1.3348	4.928493	3.692307	0.000143
PXN	14.85981	54.95646	3.698328	1.48E-05
ABCC9	0.086233	0.318951	3.698689	0.00214
SMOC1	14.75557	54.67046	3.705072	0.034263
SLC26A9	0.01533	0.056973	3.716344	0.275317
TAGLN	1.224622	4.551169	3.716389	0.097953
ENKUR	0.035391	0.131634	3.719397	0.003367
TMCO3	4.497848	16.73018	3.719596	1.34E-05
ANXA5	98.21748	365.5149	3.721485	0.000324

VPS18	2.972356	11.11718	3.740191	0.000424
DBNL	11.29183	42.24113	3.740858	0.000442
CACNA1C	0.476089	1.783199	3.745514	0.001995
BHLHE41	3.818858	14.3049	3.745858	1.41E-05
HIST1H2A	0.158877	0.595427	3.747713	0.079673
SYT14	0.055076	0.207062	3.759568	0.016256
ASL	6.390353	24.0378	3.761577	0.001737
TAX1BP3	18.27667	68.90185	3.769935	3.04E-05
BTBD6	13.90465	52.4343	3.770991	0.000125
CXCL3	1.306532	4.92924	3.772768	0.074129
ADORA2B	1.905943	7.193734	3.774369	6.81E-05
SLC28A3	0.019709	0.07442	3.775856	0.002794
ZNF556	0.092774	0.350628	3.779391	0.021223
RAB34	15.44587	58.38689	3.780098	6.77E-05
DHRS3	7.797838	29.5387	3.788062	0.246176
TRIM55	0.013121	0.049974	3.808548	0.171394
LDB3	0.013525	0.051556	3.811806	0.045638
POLD4	11.65769	44.47999	3.815507	4.72E-05
FHL2	16.5686	63.28547	3.819602	0.005816
SLCO4A1	1.937164	7.400792	3.820427	7.29E-06
DAB2	4.007115	15.33952	3.82807	0.000418
KCTD11	2.147048	8.236857	3.836363	0.002266
SFXN3	5.189657	19.91191	3.836845	0.000566
RUNX1	1.937832	7.435185	3.836857	8.70E-05
PEX10	6.298806	24.16807	3.836929	0.000355
PDGFRA	0.451047	1.731183	3.83814	0.012588
ASNA1	28.51791	109.5007	3.839718	9.11E-05
NACC1	16.14146	62.00252	3.841197	1.08E-05
MOB3C	1.232374	4.735552	3.842625	0.000273
DRAP1	141.276	542.8776	3.842673	2.89E-05
AIFM2	1.364752	5.246248	3.844104	0.007013
SPRY4	1.194896	4.593416	3.844198	0.071227
GNA12	5.953696	22.92036	3.849769	8.48E-05
MLH3	0.548104	2.111458	3.852298	0.000717
COL12A1	2.935039	11.32571	3.858794	0.00111
PFKP	25.87754	99.90816	3.860806	0.001876
MAP3K6	2.265977	8.775703	3.872813	0.002091
TBC1D2	0.899693	3.490056	3.879163	7.22E-05
NUGGC	0.007585	0.029432	3.880156	0.135159
GNG10	0.044127	0.17126	3.881043	0.080638
ADGRE2	0.036508	0.141695	3.881167	0.013099
PHEX	0.255106	0.990457	3.882529	0.008888
CYLD	3.123278	12.12622	3.882531	0.00109
SHISA4	1.876298	7.288824	3.884685	0.001279
PANO1	0.225964	0.879377	3.891659	0.02128
TCIRG1	4.826777	18.7911	3.893094	0.000597
SELPLG	0.195462	0.761701	3.89693	0.013584
KRT75	0.178297	0.694873	3.897279	0.00313
ST3GAL1	2.867638	11.17688	3.897592	0.002653
MXRA8	1.077821	4.202703	3.899259	0.010204
OR1J2	0.017496	0.06831	3.904296	0.060415
TNFRSF10	8.063696	31.48992	3.905147	0.030087
FTH1	1499.634	5872.842	3.916183	0.006573
CDH7	0.076939	0.301895	3.923852	0.010007
THEMIS2	2.131916	8.370902	3.926469	8.44E-05
DCDC2C	0.009943	0.039052	3.927511	0.25381
LOC10537	0.011671	0.04591	3.933603	0.253004
SDSL	1.914782	7.532762	3.934006	0.000798

HNMT	0.287336	1.133147	3.943626	0.001488
CAPNS1	64.51864	254.4515	3.943845	7.41E-05
FBXL17	0.351864	1.388337	3.945666	9.51E-05
PCDHGA1	0.065295	0.257835	3.948769	0.007593
TPD52L2	26.81606	106.0808	3.955868	4.60E-05
ELAC1	0.73486	2.907145	3.956054	0.013805
NFIC	6.131042	24.29187	3.962112	6.90E-05
TPGS1	5.553161	22.02113	3.965514	0.00516
MAP2K3	6.995638	27.75549	3.967543	0.00287
STX1A	7.94762	31.5395	3.968421	0.010271
MAP3K20	3.540648	14.059	3.970743	5.70E-05
ABCA1	1.360331	5.402789	3.971671	0.000416
PHOSPHC	0.415034	1.649232	3.973727	0.008074
KIF3B	6.430755	25.57364	3.976772	6.52E-06
C12orf75	34.59476	137.6911	3.980115	0.001222
ATP13A4	0.003716	0.014795	3.98124	0.027271
RNH1	24.95104	99.34028	3.981408	1.12E-05
FAM167A	1.803955	7.186333	3.983654	6.27E-05
AMOTL2	2.78171	11.08545	3.985119	0.009233
GBP5	0.019258	0.076936	3.99501	0.073592
DPP7	9.268634	37.03502	3.995736	0.000285
B9D2	1.080079	4.317991	3.997847	0.000385
BTC	0.044885	0.179458	3.998155	0.049971
HIF1A	27.95051	111.9408	4.004964	0.000221
CTSL	38.75032	155.4342	4.01117	1.66E-05
ARC	1.459249	5.8534	4.011241	0.003949
NPIPB2	0.113098	0.454178	4.015803	0.020365
UNC5D	0.011096	0.044591	4.018851	0.011314
SPAG4	5.186185	20.85728	4.021699	0.007596
MAPKAP1	15.24302	61.32947	4.023446	1.52E-06
CYSLTR1	0.063351	0.254963	4.0246	0.108371
SH3KBP1	7.63849	30.74599	4.02514	0.011064
MRPL20	30.11843	121.333	4.028531	4.03E-05
GCNT3	0.17625	0.710311	4.030144	0.163866
WIPI1	5.462836	22.06576	4.03925	0.001465
C1QL1	9.735253	39.35035	4.042047	0.017825
SPRYD3	6.832044	27.64348	4.046151	0.000455
FAM156B	0.028457	0.115194	4.047978	0.135823
CMPK2	0.009334	0.037784	4.048015	0.176365
MSN	34.9095	141.4295	4.051319	0.000301
MCOLN1	3.713014	15.06246	4.056668	0.01404
WASHC2A	3.398999	13.78911	4.056815	0.000526
ZNF883	0.535144	2.172015	4.058746	0.001811
SPRED3	0.591816	2.402897	4.06021	0.000204
VGLL3	0.916212	3.721896	4.062265	0.031826
LOC10537	0.065072	0.264658	4.067163	0.039762
CGB3	0.102951	0.419405	4.073814	0.493053
KLHL38	0.03442	0.140232	4.074147	0.062809
MMP14	42.6617	174.016	4.078976	0.014896
ULK4	0.484734	1.977755	4.080082	0.000363
SDF4	23.55074	96.42209	4.094228	0.000162
MSC	1.70483	6.992922	4.101829	0.012084
MFSD2A	4.809301	19.73105	4.102687	0.002068
VAMP3	29.13934	119.6161	4.104969	1.04E-05
GNGT2	0.0469	0.192579	4.106109	0.066557
CALB1	0.007696	0.031676	4.116124	0.502749
SEMG1	0.011503	0.047346	4.116124	0.502749
SGK1	2.533686	10.43146	4.117106	0.001748

GNA14	0.02017	0.083123	4.121201	0.291176
CCL4	0.083352	0.343576	4.121992	0.086447
SWI5	7.648816	31.58695	4.129652	0.001166
ADI1	4.902741	20.29435	4.139388	0.000441
IFI6	7.412133	30.6886	4.14032	0.001611
LOC10192	0.121636	0.503699	4.141017	0.004439
APH1B	1.483958	6.145904	4.141562	9.40E-05
GSN	13.07799	54.1841	4.143152	0.000175
MAP1B	9.914983	41.13683	4.148956	0.001529
CHST2	1.736555	7.207616	4.150525	0.034485
GATM	0.092696	0.385294	4.156548	0.173124
PLCD4	0.341259	1.418994	4.158112	0.000559
TAPBP	10.23639	42.61964	4.163542	1.10E-05
PRKCA	4.26035	17.74001	4.16398	0.002327
WNT5B	1.968079	8.20698	4.170046	0.01238
IFITM10	2.299178	9.602111	4.176323	0.114455
FLNA	66.76053	278.8703	4.177173	0.002288
MYL6	377.0811	1576.091	4.179713	0.00029
MUC16	0.000441	0.001847	4.186166	0.256625
RIN2	0.872016	3.651317	4.187212	0.000112
MFSD5	3.753035	15.72853	4.190883	0.000528
ARRDC3	4.307935	18.05587	4.191305	0.006725
SPATS1	0.017607	0.073912	4.197954	0.068077
HRASLS2	0.015325	0.064332	4.197954	0.068077
SHB	4.958446	20.82171	4.199241	3.61E-05
IL36B	2.151825	9.04266	4.202322	0.1897
RDH5	2.791127	11.75801	4.212637	0.006595
CASTOR1	2.636414	11.12243	4.218774	0.000135
MAPKAPK	14.40376	60.83175	4.223325	0.000843
ADORA1	0.762696	3.226386	4.23024	0.000778
SLC10A3	3.046768	12.89742	4.233147	1.84E-05
MCHR1	0.06903	0.292345	4.23503	0.01134
RBPMS	2.839737	12.02929	4.236058	1.00E-06
CACNG8	0.541171	2.292622	4.23641	0.001918
MCF2	0.015845	0.067158	4.238344	0.110721
TENM3	2.522557	10.69704	4.240555	0.004393
HMCN2	0.119143	0.505258	4.240763	0.00614
BMPER	1.579577	6.699758	4.24149	5.42E-05
CFL1	376.4087	1597.672	4.244515	0.000108
DRC3	0.078231	0.332126	4.245436	0.015585
WSB2	17.89738	76.12522	4.253428	0.000237
CPA6	0.006197	0.026376	4.256211	0.37459
UCN2	0.333842	1.421025	4.256575	0.035466
AKR1C3	2.121786	9.042051	4.261527	0.000226
CES1	0.139889	0.596324	4.26284	0.000267
AGTRAP	15.67388	66.82558	4.263499	0.001761
TNS2	0.829017	3.535898	4.265168	0.002513
MMP19	0.758057	3.2376	4.270922	0.000382
KRCC1	2.31213	9.883207	4.274503	0.004551
ANTXR2	1.686339	7.211532	4.276443	0.000205
NFIX	3.235468	13.84583	4.279392	0.013625
CD226	0.009784	0.041871	4.279744	0.177805
SLC22A18	2.713337	11.61761	4.28167	0.01608
DPYSL4	1.991624	8.557466	4.296728	0.00781
GALNT18	1.089403	4.682129	4.297886	0.000335
ALPK2	0.800139	3.442679	4.302599	0.001358
B3GALT6	4.23554	18.23092	4.304274	0.000769
VIM	843.6526	3646.138	4.321848	0.004084

FGF2	3.453903	14.94383	4.326649	0.042823
LOC10099	0.005018	0.021748	4.334013	0.028697
VSTM1	0.024866	0.107771	4.334014	0.028697
EML1	4.218256	18.32236	4.343587	0.001523
CPT1C	3.722809	16.18533	4.347613	0.000551
DGLUCY	1.514877	6.596218	4.354294	0.001662
SOD3	1.286637	5.604457	4.355894	0.005752
VGF	1.056479	4.604045	4.357913	0.002768
TCTA	7.196969	31.37568	4.359569	0.00498
CPLX4	0.009463	0.041308	4.365314	0.249843
TLR6	0.206932	0.903404	4.365702	0.000621
SLC35B2	16.15766	70.55845	4.366874	2.07E-05
SNTB1	1.681181	7.345671	4.369353	0.013607
EXOC3L1	0.037442	0.163712	4.372437	0.022655
TK2	2.572761	11.25108	4.373156	0.000172
TSLP	2.164193	9.473462	4.377365	0.018193
LAMC2	0.368864	1.616306	4.381847	0.001652
HIST2H2A	0.649513	2.846744	4.382892	0.019477
NR1D1	4.276019	18.7573	4.386627	0.006354
MATN2	1.415825	6.213398	4.388534	0.067698
SPHK1	3.637917	15.96614	4.388813	0.122987
TCTEX1D4	0.013471	0.059137	4.389857	0.253666
GPR176	1.815539	7.970851	4.390351	0.000111
ISM1	0.303177	1.334416	4.401437	0.045232
ENO1	450.716	1984.833	4.403733	0.003336
GCG	0.01421	0.062583	4.404051	0.359842
C14orf132	3.213924	14.18375	4.41322	7.61E-06
PARVG	0.650211	2.869883	4.413773	0.000145
AURKAIP1	23.66626	104.5777	4.418851	3.74E-05
CERCAM	8.92109	39.42488	4.41929	0.000239
APC2	0.032067	0.142208	4.434741	0.002221
ETS1	2.797499	12.40976	4.436017	0.002863
TICAM2	0.028974	0.12862	4.439176	0.163369
TINAG	0.009273	0.041183	4.441207	0.245666
ITGB5	10.15402	45.11735	4.443299	0.000195
AP2M1	117.5459	522.7057	4.446822	6.11E-06
HIST4H4	0.089262	0.39699	4.447459	0.084005
ELK3	4.876656	21.72043	4.453959	0.000648
TMEM220	0.11952	0.532597	4.456144	0.002311
PCDHB2	0.649549	2.897524	4.460827	0.001528
PLA2G2D	0.009444	0.042148	4.463128	0.366685
RNF133	0.012892	0.057541	4.463128	0.366685
LOC10798	0.004372	0.019515	4.463305	0.247086
WFDC8	0.007044	0.031441	4.463306	0.247086
ATP10A	0.002272	0.01014	4.463306	0.247086
ACTN4	49.5241	221.231	4.467139	0.000212
C6orf141	0.995973	4.452204	4.470206	0.001415
RPS6KA4	5.719486	25.59257	4.474628	0.000949
TUBAL3	0.062968	0.281961	4.477858	0.063382
TMEM215	0.006832	0.030597	4.478632	0.430401
THSD4	0.686794	3.078229	4.482025	0.003005
POTEJ	0.014214	0.063716	4.48265	0.110906
ATP6V0E1	64.24218	289.1449	4.500857	0.000857
PTPRN	4.712173	21.21826	4.502861	0.003889
BASP1	43.22688	194.6893	4.503893	5.84E-05
EPHB2	1.625393	7.323768	4.505845	0.014584
TXNDC15	6.43436	29.00582	4.507957	2.24E-06
TGFBR1	3.310647	14.92538	4.508297	0.031984

FAM187A	0.562505	2.539071	4.513861	0.018251
ZSWIM2	0.007518	0.033946	4.515518	0.364822
TRIM72	0.0065	0.029351	4.515697	0.245068
CCDC168	0.004226	0.01909	4.517585	0.001568
C4orf47	0.370308	1.672992	4.517842	0.04338
PIK3CD	2.701587	12.20948	4.519372	0.000958
PLOD1	43.04437	194.6472	4.522014	0.000985
CCDC85B	26.69615	121.1113	4.536658	5.81E-05
CARD19	16.11104	73.12048	4.538531	0.000419
LAMB3	2.017807	9.175362	4.547196	0.00086
COL8A1	2.016072	9.172278	4.549579	0.000948
BTG4	0.008607	0.039273	4.562842	0.306982
GRPR	0.014553	0.066403	4.562843	0.306982
CYP1B1	5.354901	24.52147	4.579258	0.015178
GFPT2	19.33109	88.61366	4.583998	2.44E-06
RNF175	0.358735	1.646972	4.591055	2.91E-05
SP140	0.017959	0.082498	4.593691	0.063648
SOAT2	0.044996	0.206701	4.593782	0.0265
POLE4	21.07092	96.83475	4.595659	0.000348
ZNF93	0.10857	0.499262	4.598511	0.000976
CEBPB	32.67212	150.2671	4.599244	0.023337
PLEKHA2	1.407048	6.471977	4.599683	0.004922
COL6A2	44.40407	204.36	4.60228	0.003773
ATL3	4.632036	21.32404	4.603601	1.30E-05
NPAS2	2.540793	11.70287	4.605991	4.81E-05
ARHGEF2ɛ	0.647123	2.982358	4.608638	0.005841
CAPN2	48.99738	225.9586	4.611646	3.63E-05
CSF1	3.905417	18.02417	4.615172	0.001217
RAB33A	0.283207	1.309167	4.622646	0.004005
ANO10	6.009151	27.78128	4.623163	3.88E-05
IRF7	0.494958	2.288783	4.6242	0.003169
SLC16A7	0.175534	0.812277	4.627456	0.003934
RHOC	85.43092	395.5793	4.6304	0.000103
RP1	0.018043	0.083659	4.636593	0.048955
F8	0.350428	1.626063	4.640222	0.00026
DPYD	0.458439	2.12964	4.645411	0.000281
F3	10.19278	47.4433	4.654598	0.000563
FOXF1	3.790696	17.64706	4.655362	0.005875
TNFAIP2	0.676372	3.158946	4.670423	0.000105
SARDH	0.517466	2.418598	4.673929	0.000115
HPS6	2.107926	9.852831	4.674182	0.000447
IL1R1	1.01831	4.75996	4.674374	0.001753
TMPRSS1!	2.803434	13.13075	4.683808	0.058952
RGS6	0.001238	0.0058	4.685679	0.351422
SMTN	9.199663	43.11626	4.686721	0.003208
IL17RC	3.033361	14.2257	4.68975	0.001081
CENPS-C(	0.54936	2.581742	4.699546	0.000631
MYL12A	62.93696	296.2656	4.707338	9.91E-05
FZD8	2.841104	13.38362	4.71071	0.00317
HIST1H4H	0.249402	1.176456	4.717113	0.111063
LDB2	0.081076	0.382953	4.723383	0.024987
CDC42EP2	3.306682	15.61905	4.723483	0.002377
HLA-DRB!	16.12897	76.31299	4.731423	0.019125
HTR2A	0.017306	0.081983	4.737207	0.082568
DDX58	0.689947	3.26992	4.739377	0.001835
PAM	19.09638	90.51894	4.74011	0.002493
RASA3	3.996369	18.94854	4.741439	4.05E-05
CARD6	0.377518	1.790135	4.741852	0.016553

PON2	12.90108	61.18339	4.742502	3.02E-05
S100A5	0.039436	0.187365	4.751103	0.03079
FAM213B	4.468027	21.26655	4.759719	6.49E-05
SNX7	9.814289	46.82649	4.771256	0.000452
ADAMTS1	0.086107	0.411645	4.780643	0.015493
FUT8	2.242602	10.73154	4.785307	0.000875
NMI	1.737208	8.326933	4.793286	0.000141
CYBRD1	3.552954	17.0556	4.8004	0.000239
C10orf67	0.02093	0.100675	4.810139	0.000802
FSIP1	0.078465	0.377575	4.81202	0.001626
ZNF233	0.147193	0.708753	4.815113	0.010786
ENDOG	4.424777	21.30784	4.815572	0.000269
SLC35C1	2.155561	10.42367	4.835711	0.000264
ARSB	0.499663	2.419882	4.843024	0.01099
CNIH2	2.569524	12.4512	4.845724	4.60E-05
PRRT3	0.938685	4.551863	4.84919	0.003864
CTSD	88.02042	427.0391	4.851592	3.02E-05
TNFRSF14	0.290124	1.408077	4.853359	0.001436
C5orf66	0.220507	1.071469	4.859115	0.002159
TCERG1L	0.014053	0.068288	4.859408	0.197326
PCDHB16	0.455207	2.212319	4.860024	0.001241
FAM214B	4.231994	20.57865	4.862636	0.003545
COL16A1	2.439858	11.89015	4.873296	0.001167
SNX21	2.744727	13.38018	4.874868	8.90E-05
SLN	0.077398	0.377746	4.880596	0.226059
PPBP	0.028468	0.139178	4.888926	0.421699
SPRY2	11.59618	56.75596	4.894369	0.000362
C8orf46	0.059979	0.294267	4.906133	0.000885
TACR1	0.007452	0.036568	4.907111	0.152408
IER3	47.15912	232.0867	4.921353	0.010872
CRABP1	0.072015	0.354531	4.923008	0.434052
LOC10192	0.053628	0.264177	4.926118	0.020089
RAB22A	3.362194	16.57636	4.930222	0.000217
MMP24-A	0.148702	0.73326	4.931083	0.010976
KIAA1217	0.729583	3.602681	4.937998	0.000101
SMAGP	4.445257	21.98617	4.945984	0.00262
SLC22A1	0.021152	0.104664	4.948264	0.048462
CAPG	13.98231	69.20205	4.949259	0.000776
IL12RB1	0.013667	0.067709	4.954338	0.070202
KLHL5	2.613443	12.96343	4.960288	6.33E-05
CCDC80	5.9514	29.57577	4.969547	0.013054
PORCN	5.752769	28.66353	4.982562	0.00381
ISG15	7.708235	38.47646	4.991604	0.000316
CLEC11A	13.33112	66.57405	4.993885	0.000139
TMEM132	20.32906	101.6322	4.999358	0.041466
GRIA1	0.003145	0.015755	5.009561	0.211398
PLEKHO2	2.09179	10.48312	5.011556	0.000639
HIST1H4C	0.050021	0.251166	5.021179	0.079217
BCAR1	2.513401	12.62261	5.022126	0.000219
SPATS2L	3.379086	16.97771	5.024349	0.000168
HIST1H2B	0.554328	2.786907	5.027541	0.12531
CIITA	0.262212	1.318317	5.027678	0.014365
CALD1	13.57708	68.39737	5.03771	0.004248
SEC14L2	3.052904	15.41137	5.048102	0.001978
LINC0045	0.005973	0.030201	5.056201	0.015495
XKR3	0.030393	0.153688	5.056626	0.032418
KCNK2	0.01568	0.079389	5.063041	0.057205
TUBB4B	154.175	782.7944	5.077312	5.23E-05

LIMK1	13.78962	70.03172	5.078583	0.004211
PCDHGA8	0.010164	0.051796	5.096003	0.077591
PLA2G5	0.00565	0.028829	5.10284	0.214553
KCNH1	0.006821	0.034816	5.103926	0.03974
BPIFC	0.0413	0.210798	5.104113	0.115686
ITGAM	0.015653	0.080074	5.115484	0.321565
ANGPTL2	1.060074	5.422871	5.11556	0.000142
PDZRN3	1.816337	9.316322	5.129182	0.00184
RAB27A	2.37967	12.20859	5.130372	6.03E-05
SUSD1	1.111242	5.706233	5.135005	2.76E-05
CD59	19.79469	101.8553	5.145586	3.24E-05
TNFRSF12	38.6588	199.0986	5.15015	0.006028
NPTXR	4.606223	23.79616	5.166089	0.00024
BRI3	8.22116	42.56048	5.176943	0.000617
TFF2	0.026277	0.136099	5.179476	0.472526
PNOC	0.008361	0.043307	5.179476	0.472526
SLC31A2	0.444752	2.30532	5.183387	0.005823
HIST2H3P	0.094581	0.490454	5.185527	0.123303
THAP8	1.201899	6.253552	5.20306	0.000905
NRXN2	1.425288	7.429009	5.212288	0.009888
HBEGF	4.637881	24.17552	5.212623	0.001956
FSCN3	0.009838	0.051439	5.228561	0.251035
FMO3	0.025376	0.133209	5.249365	0.004355
PCDHGA9	0.060805	0.319715	5.258058	0.004388
HSD3B7	1.938405	10.19631	5.260153	0.017099
SLC16A2	6.53447	34.45736	5.273168	0.003168
RHD	0.004527	0.02389	5.27743	0.357153
HCRTR2	0.008298	0.043791	5.27743	0.357153
MGAM	0.017949	0.094765	5.279765	0.001764
CYB561A3	6.703216	35.44032	5.287062	0.004108
ETV5	4.122341	21.82363	5.293989	0.000232
LXN	0.822855	4.356877	5.29483	0.000577
C5orf56	0.836991	4.434495	5.298138	0.001304
SPATA4	0.391695	2.077187	5.303069	0.001287
LOC10193	0.012226	0.064977	5.314586	0.247276
TIMP3	34.99547	185.9946	5.314821	0.003825
TSPAN4	12.06495	64.17184	5.318863	0.000668
ZSCAN5A	0.629018	3.350862	5.327134	0.004978
CXCL8	38.24632	204.0393	5.334873	0.213091
MAN2B2	3.253426	17.36354	5.337001	6.39E-06
SGCD	0.081448	0.434843	5.338935	0.00099
FAM114A1	3.69583	19.75366	5.34485	6.70E-05
SNAI2	8.201008	43.83613	5.345212	0.017869
CD151	109.2149	585.0306	5.356691	7.93E-06
TRIOBP	3.497091	18.74942	5.361434	0.002035
FHAD1	1.482502	7.948766	5.361725	6.79E-05
MYH9	50.02584	268.3988	5.365203	0.000405
BSCL2	14.53231	78.0239	5.368997	9.39E-05
ERRFI1	7.642356	41.03613	5.369565	0.001157
PER3	0.445496	2.393435	5.372516	6.84E-05
LDLRAP1	2.324389	12.48897	5.373014	2.57E-05
NHS	0.342108	1.840187	5.378966	0.000541
DMRT1	0.024858	0.133935	5.38798	0.240378
SH3BP4	4.099135	22.10496	5.392592	2.92E-06
TMBIM1	6.723771	36.35075	5.406304	3.33E-05
PRICKLE2	0.446007	2.411522	5.406912	6.43E-07
C3AR1	0.115997	0.627511	5.409698	0.004225
ASB5	0.003749	0.020296	5.413906	0.248202

LOXL2	73.74994	399.7995	5.421014	0.011931
PLA2G4D	0.004959	0.026909	5.426232	0.200602
APOBEC3I	4.107745	22.31555	5.432555	1.97E-05
S100A11	218.1633	1186.105	5.436775	6.93E-05
PCDHB10	0.230661	1.25459	5.439097	0.019038
PREX1	4.514175	24.58723	5.446672	0.008235
PCDHB5	0.349893	1.909909	5.458545	0.005038
FRMD4A	0.507405	2.769836	5.458829	0.000597
EMP3	37.04849	203.399	5.490076	0.00013
TMEM45A	3.917065	21.50761	5.490746	0.000208
TIE1	0.036434	0.200055	5.490903	0.063277
KCNS3	1.726203	9.482085	5.49303	0.000244
NEK11	0.210079	1.154888	5.497387	0.000125
GALNS	2.002585	11.00999	5.49789	3.47E-05
TPM4	69.15294	380.2673	5.498932	0.000331
CENPS	0.241546	1.333176	5.519352	0.001027
DMRT2	0.021725	0.119988	5.52299	5.30E-05
TRIM47	3.606074	19.92583	5.525628	0.000633
CCL20	0.310707	1.718109	5.529673	0.148561
C3orf18	1.527397	8.452681	5.534042	5.13E-05
C19orf24	13.89343	76.89293	5.53448	0.001229
CAMK2N2	5.1024	28.32535	5.551377	0.000182
TICAM1	1.637908	9.113307	5.563992	1.03E-05
STAR	0.020709	0.115252	5.565216	0.083905
ELOVL1	18.26589	101.6954	5.567504	2.55E-08
GPR55	0.011897	0.06637	5.57861	0.118806
TRPC4	0.179423	1.004036	5.595915	0.011883
UPB1	0.143818	0.805105	5.598066	0.004613
PCDHGA4	0.043038	0.240991	5.599524	0.025616
LOC10537	0.023791	0.133252	5.601032	0.149479
MXRA7	18.17595	102.0475	5.614423	1.75E-06
LOC65351	0.140734	0.790401	5.616291	0.00054
ITGB6	0.008085	0.045419	5.617403	0.017917
IL21R	0.277695	1.563741	5.631135	0.00159
NABP1	1.32879	7.486949	5.634409	0.000181
CCDC107	6.736804	38.09908	5.655363	1.91E-05
NFASC	1.439209	8.142261	5.657455	0.000716
MMP24	0.074362	0.420903	5.660207	0.004792
FXYD5	74.78361	423.9304	5.668761	0.001561
KANK2	4.863942	27.58372	5.671063	0.00047
ITGB1	57.03241	323.8167	5.677766	0.000657
DPP4	1.258521	7.149298	5.680714	0.000149
SSMEM1	0.038117	0.216757	5.686593	0.067942
ACTN1	14.07953	80.08117	5.687774	8.71E-08
PNPLA6	5.888214	33.50084	5.689473	0.000138
ECM1	7.885183	44.8745	5.69099	0.000106
COL6A3	27.7146	157.9235	5.698204	0.00514
SVIL	2.566899	14.64845	5.706671	0.002166
CNKSR2	0.025883	0.147917	5.714812	0.000414
ADM	17.28646	99.03088	5.728812	0.008026
FOSL2	3.644552	20.88689	5.730989	0.000445
LCMT2	1.266477	7.267297	5.738199	0.000683
SLC19A3	0.124652	0.716121	5.744946	0.003147
LY6K	0.16927	0.974234	5.755519	0.013221
OSTM1	3.337268	19.20943	5.756034	0.000641
PSMB10	8.685596	49.99689	5.7563	0.000309
SLC35E4	1.063877	6.12514	5.757378	5.02E-05
SLC25A18	0.049179	0.283274	5.76004	0.017679

FAM212A	0.624314	3.598265	5.76355	0.019464
MAGEH1	3.074788	17.73011	5.766286	0.00048
TNIP1	8.206724	47.34841	5.769466	5.40E-05
MOB3A	5.540557	32.00721	5.776895	0.001224
MOK	1.793313	10.37661	5.786277	0.007552
SLC11A1	0.026273	0.152166	5.791803	0.063867
CARD10	4.472884	25.94219	5.79988	0.000715
RPS27L	39.08923	227.5961	5.822477	3.75E-05
IKBKE	0.542671	3.163476	5.829449	0.000299
LPAR1	1.080711	6.302585	5.831888	0.002921
CD28	0.003405	0.019861	5.832617	0.263517
ZC3H12A	3.294851	19.28046	5.851694	0.019984
TMEM26	0.009127	0.053441	5.855447	0.094484
ZNF563	0.37775	2.212264	5.856419	0.001158
GLRX	16.68173	97.95681	5.872103	8.07E-06
NRP1	8.451849	49.63982	5.87325	0.000169
NR1H2	10.61306	62.37413	5.877112	4.04E-05
TSPAN10	0.696865	4.09695	5.879114	0.000144
PLEC	4.24393	24.95094	5.879205	0.000109
TOR4A	0.728284	4.286739	5.886084	9.77E-05
GALNT10	3.061644	18.04214	5.892957	1.26E-05
MFSD1	5.430313	32.03305	5.898932	0.001213
B2M	113.3069	672.3699	5.934057	1.28E-05
COL15A1	0.69884	4.163488	5.957716	0.012606
SPINK13	0.038327	0.228469	5.961087	0.240041
FGFBP1	0.096332	0.574276	5.961405	0.149251
TAPBPL	0.595469	3.550651	5.962782	0.000112
IL7	0.047271	0.282546	5.977195	0.013948
GSAP	0.53639	3.207687	5.980136	0.000454
CHST14	4.577253	27.38566	5.98299	0.000143
CMKLR1	0.023798	0.142456	5.985986	0.002777
NDST3	0.006976	0.041766	5.987527	0.007336
ARHGAP2	0.900425	5.398108	5.995069	9.85E-06
MME	1.193642	7.187964	6.021877	0.004947
MYO16	0.017045	0.102659	6.022817	0.275857
EFHD2	11.30968	68.12013	6.02317	0.000179
TTC29	0.055594	0.335658	6.037655	0.123135
HYAL3	0.69971	4.22493	6.038115	0.000299
HS3ST4	0.005808	0.035098	6.042722	0.456583
ANXA10	0.024495	0.148316	6.054993	0.409638
HCN2	6.758157	40.94915	6.059218	0.00274
CDC42EP3	3.936015	23.86328	6.062801	0.001094
GRAPL	0.002847	0.017272	6.067264	0.359556
LOC10192	0.252755	1.534635	6.07163	0.00475
PLEK2	0.133381	0.81123	6.082073	0.051919
HEG1	3.833935	23.46607	6.120624	0.000705
CALHM5	0.276731	1.695694	6.127587	0.000948
CLEC3B	0.496543	3.043366	6.129105	0.150933
OSGIN1	2.236875	13.73551	6.14049	0.012357
FGF12	0.161151	0.990205	6.144583	0.019573
IFI30	31.45422	193.8705	6.163579	8.72E-05
VEGFC	4.14675	25.57484	6.167442	0.000428
LDHA	377.8447	2333.173	6.174953	0.00314
CLU	13.84593	85.66216	6.186813	0.001427
FAM170B	0.013013	0.080529	6.188144	0.193637
S100A4	4.944969	30.63306	6.194794	0.000187
KCNE5	0.113244	0.70178	6.197058	0.013454
TRADD	2.702248	16.75638	6.200905	0.000336

RNF148	0.014123	0.087743	6.212795	0.10426
LTBP3	16.15519	100.5599	6.224621	0.030513
PRL	0.012137	0.075558	6.2253	0.060386
GRM7	0.001457	0.009074	6.226345	0.26715
ARSJ	0.517889	3.227834	6.232673	0.012833
SPANXB1	0.122858	0.766452	6.238512	0.078715
NUDT22	6.52712	40.7316	6.240363	7.68E-05
CREB3L1	5.641806	35.2226	6.243143	0.000431
UGT2B7	0.018996	0.118803	6.254119	0.017057
ITGA5	44.85415	280.5675	6.255107	4.04E-05
DDX60L	0.152032	0.953315	6.270495	0.000216
PRRX1	2.292203	14.38557	6.275871	0.000858
GAP43	0.444075	2.789711	6.282068	0.029893
MUC13	0.058007	0.364643	6.286153	0.336486
PFKFB4	3.63856	22.88363	6.289198	0.013978
CPQ	2.189919	13.78914	6.296641	0.008415
CNIH3	1.558436	9.841309	6.314863	0.001564
LOC10798	0.014613	0.092358	6.320251	0.006069
NAAA	0.758449	4.800481	6.329342	2.77E-06
GPR84	0.068248	0.431975	6.329499	0.29383
IER5L	2.701918	17.12495	6.338072	0.006536
OAS3	0.371696	2.361841	6.354226	0.001641
FST	0.700294	4.466166	6.377559	6.12E-06
SPSB1	2.728913	17.4155	6.381843	5.28E-06
SCARA3	0.960494	6.138413	6.390891	0.005002
IQCG	3.159669	20.21349	6.397342	0.000588
SHISAL1	1.92768	12.33287	6.397778	2.82E-05
EFEMP2	6.538546	41.91035	6.409735	0.000246
TUBB3	15.68863	100.6004	6.412312	0.000399
IL24	0.343276	2.202814	6.417032	0.196642
CYP11A1	0.038044	0.244595	6.429201	0.008624
RAB31	13.64822	87.75233	6.429582	0.014969
CNRIP1	3.193899	20.54261	6.431827	2.91E-05
MSANTD3	6.909142	44.4412	6.432231	2.19E-05
GALNT5	0.283741	1.829105	6.44639	8.47E-05
PTPRCAP	0.146571	0.946616	6.458392	0.015739
IGFBP7	18.94887	122.5286	6.466277	4.99E-06
P3H2	1.059025	6.857752	6.475535	6.07E-05
PLA2R1	0.004575	0.029658	6.482129	0.027611
FOXD1	14.17173	91.88267	6.48352	0.002266
SLC2A6	1.292542	8.385302	6.487452	0.000655
CCDC103	1.109801	7.250951	6.533562	0.000566
COX20	3.809814	24.90558	6.537217	0.000625
CD63	115.0042	752.1046	6.539802	7.73E-06
EPDR1	5.220259	34.14854	6.541542	0.000169
DUSP4	1.801107	11.78811	6.544928	0.002237
MX2	0.136841	0.89757	6.559193	0.011396
NANOS3	0.522716	3.430541	6.562919	0.034807
BCL2L1	10.34414	67.90723	6.5648	6.59E-06
TMEM200	0.913697	6.001887	6.568792	1.77E-05
CD200	0.024079	0.158437	6.580005	0.064644
ROM1	0.831864	5.475354	6.582034	4.94E-06
PCSK1	0.119	0.784073	6.588844	0.000154
MYOM3	0.02915	0.192178	6.592756	0.002226
SRPX2	3.110717	20.52794	6.599103	1.87E-05
RIN3	1.436737	9.482026	6.599696	0.000271
EHD1	11.82483	78.15407	6.609316	7.51E-06
TRAM2	3.886882	25.69249	6.61005	0.002335

PLIN3	21.4235	141.6121	6.610132	7.46E-06
SGCG	0.114456	0.756949	6.613425	0.001072
PSMD2	50.02525	331.2252	6.621161	8.43E-05
LMNA	32.79402	218.4689	6.661851	0.00191
CLIP3	2.416875	16.11935	6.669504	9.55E-05
CD163	0.004233	0.028271	6.679246	0.140777
C8orf48	0.195069	1.304025	6.684955	0.001308
ATP2B2	0.005675	0.038062	6.707029	0.002311
GPD1	0.006328	0.042634	6.737672	0.011632
AFP	0.05487	0.36991	6.741569	0.00187
CYP2C9	0.010483	0.070874	6.761076	0.013074
NKAIN3	0.011803	0.079819	6.762302	0.009789
BEND6	0.34542	2.34363	6.784864	0.012444
DPYSL2	6.452479	43.83242	6.793113	0.000271
MRGPRF	0.061792	0.420505	6.805214	0.014727
CPA5	0.021929	0.150323	6.854907	0.053909
PARP14	0.575351	3.950751	6.866675	0.000351
RNF112	0.510656	3.506751	6.867147	0.000254
ASB9	0.117693	0.808362	6.868422	0.003227
CTSK	2.884689	19.8371	6.876686	0.000141
C5AR2	0.031407	0.216817	6.903509	0.023309
CALHM2	1.420321	9.810427	6.907192	1.96E-05
RHBDL2	0.12313	0.8532	6.929265	0.000141
SAMD15	0.255826	1.772752	6.929527	0.001305
SH2D4A	0.854557	5.926348	6.934998	5.54E-05
ECE1	14.28521	99.0825	6.936022	0.000455
PARVA	1.890655	13.11645	6.937519	2.92E-06
ZNF558	0.486547	3.376181	6.939062	0.000182
CASC10	0.807412	5.608371	6.946106	0.000394
TGFB1I1	4.324335	30.04439	6.947747	1.82E-05
FLVCR2	0.180864	1.257856	6.954712	0.003668
PADI3	0.348786	2.435545	6.982926	0.000254
RIPOR3	0.418217	2.924719	6.993299	0.035523
FIBCD1	0.569876	3.990218	7.00191	0.00163
TMEM52B	0.005959	0.041854	7.024189	0.35762
TREX1	1.971413	13.86103	7.031012	0.000241
SLFN12	0.12283	0.86397	7.033888	0.001732
SHROOM:	0.296921	2.09157	7.044205	0.000377
HIVEP3	0.257198	1.8119	7.044756	0.000221
HIST1H2A	0.792903	5.593607	7.054595	0.153522
TMCO4	0.610182	4.308027	7.060236	1.37E-05
TUBA4B	0.013238	0.09348	7.061344	0.268005
LGALS3	24.5332	173.3968	7.067844	0.004651
PRR34	0.050804	0.359195	7.070206	0.005792
TMEM106	0.088897	0.62862	7.071316	0.011156
POLR2L	48.1329	341.1463	7.087592	0.000223
ACP5	1.169215	8.301737	7.100264	0.035721
C13orf46	0.007716	0.05487	7.110915	0.130761
CRHBP	0.345405	2.464539	7.13522	0.000846
DIRAS3	0.372803	2.660356	7.136088	0.000369
SPI1	0.018172	0.129738	7.139291	0.247353
SOX15	0.190268	1.359377	7.14455	0.004125
AJM1	0.257431	1.841303	7.152607	0.000186
PTN	4.530265	32.52891	7.180354	0.009094
SYTL2	0.456011	3.276237	7.184548	0.013281
GP5	0.005264	0.037826	7.185495	0.142963
SEMA5A	0.529347	3.808955	7.195567	0.004062
ZNF660	0.009535	0.06865	7.19958	0.036459

ENC1	2.287385	16.4758	7.202897	4.93E-05
PTPRR	0.050689	0.365827	7.217057	0.105043
KRBOX1	0.275748	1.991277	7.221363	0.00212
AMPD3	0.754747	5.463439	7.238771	0.000157
PHLDA1	11.13237	80.62105	7.242042	0.000516
SDC4	24.99454	181.147	7.247465	0.004622
FGF13	0.615211	4.461639	7.252212	0.000119
TXNDC2	0.044338	0.321745	7.256583	0.052509
CTGF	5.206664	37.84581	7.268726	0.06202
MYO7B	0.007891	0.057438	7.279194	0.040773
FCRLB	0.343406	2.503441	7.29003	0.019612
SFRP2	1.727767	12.59831	7.291668	0.082584
S1PR3	2.392991	17.45274	7.293276	1.69E-05
LAMP1	28.81177	210.5772	7.308722	2.80E-07
C19orf66	2.360668	17.27106	7.316177	6.49E-05
BNIP3	38.25169	280.3257	7.328451	0.01847
MMP2	94.76641	696.5693	7.350382	5.56E-05
TEK	0.031438	0.231119	7.351509	0.000267
SUMF1	1.380862	10.17562	7.369032	7.36E-06
LRRC6	0.105834	0.780048	7.370463	0.011614
LFNG	0.367504	2.709473	7.372638	0.0003
TMSB10	822.5006	6066.692	7.375912	0.0047
ADGRF2	0.014694	0.108569	7.388818	0.068837
EHF	0.046825	0.346144	7.39235	0.002214
TPBG	8.876437	65.65088	7.396085	1.03E-05
GRAP2	0.014182	0.104902	7.396984	0.006586
CAMK2N1	10.9549	81.08385	7.401605	0.016135
FAP	0.736627	5.454437	7.404614	5.72E-05
PRDM8	0.466215	3.453455	7.407426	0.000311
ARHGAP2	1.388479	10.29243	7.412734	0.003234
EHD2	8.411183	62.41801	7.420836	0.00151
C1QTNF1	3.62934	27.00853	7.441719	3.98E-05
MCTP1	0.333448	2.48413	7.449826	0.002258
LOC10537	0.00319	0.023857	7.479236	0.214304
GPER1	0.101084	0.756568	7.484519	0.114314
APOL6	0.278581	2.086918	7.491253	8.98E-05
NCEH1	3.380615	25.37174	7.505065	1.05E-05
GPR85	0.274533	2.060691	7.506157	9.00E-05
1-Dec	0.01023	0.07687	7.514257	0.095181
TP53I3	0.39825	2.993935	7.517736	0.000411
MYZAP	0.293851	2.210376	7.522097	0.000598
ZC3H12D	0.0228	0.171859	7.537656	0.19655
RAET1G	0.494641	3.743033	7.567172	0.00016
KRT16	0.032486	0.245831	7.567215	0.188132
ERICH6	0.014646	0.110992	7.578408	0.014831
KRT6B	0.008549	0.064835	7.584132	0.176447
S100A10	141.202	1071.698	7.589824	0.004589
TNFRSF10	0.022295	0.169482	7.601727	0.054985
PRR19	0.150887	1.148457	7.611394	0.002297
PLGLB2	0.006468	0.049284	7.619323	0.022898
PI3	5.301024	40.42661	7.62619	0.140251
ELMO1	0.581212	4.433653	7.628289	0.00159
FLRT2	0.166666	1.271446	7.628718	0.001009
ST8SIA5	1.530551	11.67957	7.630957	0.040384
ACTB	414.8393	3175.676	7.655196	0.000106
WDR1	26.17005	200.699	7.669033	0.000147
PCDHGB1	0.052864	0.40633	7.686277	0.000135
PAPPA	0.91838	7.063987	7.691793	0.001371

SLC35D2	1.084051	8.341071	7.694352	6.78E-05
VEGFA	30.12725	232.2185	7.707922	0.000164
DUSP6	4.032154	31.09293	7.711246	9.39E-05
ZNF311	0.030011	0.232327	7.741387	0.001126
HTRA1	28.01652	217.5519	7.765127	0.01649
CCL2	4.729995	36.75082	7.769738	3.46E-05
ADAMTS1	0.101989	0.792818	7.773596	0.009381
LOC10537	0.012435	0.096694	7.776176	0.014013
VCX3B	0.064102	0.498838	7.781939	0.101878
OSMR	2.906291	22.63373	7.78784	6.08E-05
PPL	0.158547	1.234872	7.788669	0.035398
PCDHB3	0.026576	0.206997	7.788908	0.006106
STEAP3	6.365943	49.62143	7.794828	0.001074
SLC6A17	0.14395	1.124434	7.811305	0.000258
UBE2L6	11.16289	87.25676	7.816681	9.75E-05
SPATA12	0.014815	0.115859	7.820467	0.013951
CSTB	75.3972	589.7451	7.821843	0.000713
TNIP3	0.103447	0.811581	7.845358	0.011277
GAS2L1	2.462592	19.33821	7.852785	7.44E-07
PLEKHO1	7.961192	62.73353	7.879916	0.001358
PADI1	0.019321	0.152409	7.888452	0.002626
CXXC5	6.132798	48.38097	7.88889	0.000141
LOC11226	0.008974	0.070986	7.910072	0.279855
MT1G	0.045935	0.36363	7.916112	0.169267
HIST1H2B	0.129484	1.025859	7.922662	0.114665
SBSN	2.792523	22.1774	7.941705	0.006321
TLR1	0.036748	0.291842	7.9418	0.001396
LAT	2.016715	16.02973	7.948437	0.003113
OTOA	0.003614	0.028741	7.953192	0.172975
HGF	0.234419	1.865545	7.958171	0.058176
AKR1C1	3.429235	27.35661	7.977469	0.008118
KCNK3	0.008827	0.070457	7.98209	0.007122
GPR141	0.001578	0.012597	7.984383	0.063792
GBE1	6.319101	50.60462	8.008199	0.007085
TTC16	0.005537	0.044345	8.009034	0.168485
HIST1H2A	0.23212	1.859559	8.011199	0.006742
TPM1	9.751441	78.28574	8.02812	0.000699
AHNAK	7.773584	62.40888	8.028328	0.004548
KRT7	0.234411	1.887644	8.052701	0.006334
SH3RF2	0.531051	4.28964	8.077649	2.37E-05
LOC11226	0.063637	0.51573	8.104297	0.001363
CNGB1	0.00561	0.045483	8.107924	0.118383
PLEKHG4E	0.102756	0.835693	8.132823	0.1348
IFI27L2	24.8444	202.2925	8.142377	6.49E-06
TCF7	0.497494	4.054331	8.149511	0.000574
KCTD16	0.051186	0.417996	8.166167	0.000398
MYLK2	0.033073	0.270131	8.167832	0.001106
SLC45A1	0.547646	4.47634	8.173781	0.003168
SPACA6	0.368636	3.015538	8.180262	3.83E-05
ESM1	0.054526	0.446167	8.182658	0.018173
C16orf71	0.024813	0.203319	8.194178	0.031145
LOC10798	0.00641	0.052582	8.203503	0.038618
NAPRT	0.760316	6.239179	8.206036	8.32E-06
TNC	23.84935	195.97	8.216998	0.005425
BICC1	0.209126	1.719953	8.224479	1.86E-05
BAALC	5.853167	48.2853	8.249431	0.014015
FAM167B	0.292213	2.414911	8.264205	0.006163
C17orf97	0.245574	2.034107	8.283069	0.000642

BTNL8	0.003742	0.031113	8.314078	0.191106
SLC1A1	2.169281	18.06401	8.32719	0.002871
WFDC10B	0.05441	0.453779	8.33996	0.110563
DOLK	1.714281	14.30984	8.347428	0.000748
CYP4F11	0.010848	0.090622	8.354004	0.018918
EBF1	0.048121	0.402718	8.368915	1.75E-06
ADGRE1	0.735049	6.155617	8.374427	6.35E-06
SLC2A1	7.551211	63.25734	8.377113	0.014581
STEAP1	5.284177	44.26974	8.377792	3.52E-05
RTP4	0.075172	0.6314	8.399413	0.009079
CHI3L1	10.22655	86.44204	8.452711	5.09E-05
PEG10	1.631397	13.80657	8.463036	0.013804
LNX1	0.134858	1.143616	8.480148	0.001683
RSPO3	0.089137	0.757686	8.500208	0.155487
CTSA	20.66861	175.7803	8.504699	4.27E-06
OSR1	0.242464	2.066633	8.523448	0.075218
PKM	264.7446	2263.032	8.547982	4.26E-07
SP140L	0.554099	4.73889	8.552425	2.09E-05
SYT11	3.140491	26.87709	8.558244	1.81E-05
LOC1005C	2.006037	17.19706	8.572656	2.69E-05
ARHGAP6	0.027716	0.238114	8.591094	0.002328
ITGB7	0.025014	0.215019	8.595913	0.087103
IFI35	1.889004	16.25935	8.607369	3.65E-06
TMEM240	0.323966	2.794906	8.627154	0.015424
CCL26	0.640698	5.529529	8.630473	0.021542
IQCN	0.030001	0.259379	8.645758	0.171112
GABRB1	0.009801	0.084813	8.653688	0.110175
APOH	0.237626	2.058553	8.662991	0.002778
DKK1	1.074405	9.310775	8.665979	0.005382
SERTAD1	3.196146	27.69809	8.66609	0.004123
RFTN1	3.586834	31.09607	8.669504	4.27E-06
CXCL14	0.274825	2.389798	8.695695	0.0253
NCF2	0.02236	0.194608	8.70357	0.244888
ZBTB38	0.778018	6.798317	8.737999	0.005114
XAF1	0.031773	0.278604	8.7686	0.008789
SOCS1	0.479229	4.207504	8.779739	0.002253
TNFSF13B	0.07789	0.684985	8.794259	0.009702
LOX	10.12108	89.12854	8.806225	3.07E-07
LIPC	0.019047	0.167885	8.814306	0.046746
HIST1H3A	0.039207	0.346319	8.833112	0.17432
DPYSL3	4.16754	36.86169	8.844953	0.013721
SSTR3	0.002464	0.021791	8.845437	0.11454
C3	14.85721	131.5008	8.850973	0.002185
CA12	9.186988	81.74682	8.898108	0.004778
PCDHB11	0.063681	0.566924	8.902523	0.001317
ANXA2	55.16365	491.7191	8.913824	0.000287
PPP1R3C	1.085646	9.686787	8.9226	0.001351
SP110	0.288715	2.578044	8.929378	9.52E-06
NPAS4	0.005715	0.051047	8.932163	0.001757
MLPH	2.390635	21.49426	8.991025	0.000478
ANKRD53	0.008358	0.075259	9.00455	0.021549
SLPI	0.191478	1.724752	9.007593	0.039927
RSPH9	0.304866	2.753307	9.03119	0.002933
ARSI	1.185798	10.73658	9.054311	5.58E-05
RPTN	0.005466	0.049498	9.055472	0.426537
FXYD7	0.741455	6.717567	9.059979	0.00024
S100Z	0.024834	0.22503	9.06146	0.003478
MICALCL	0.052261	0.474419	9.077798	0.008313

SNX19	0.216238	1.969141	9.106351	0.000146
GPR146	0.147562	1.344354	9.110436	0.019777
LANCL3	0.076826	0.702094	9.138701	6.13E-05
ZNF541	0.068885	0.63444	9.210096	0.000447
KRT14	0.011924	0.110069	9.230581	0.158552
NQO1	14.34782	132.4627	9.232255	0.000427
JOSD2	5.238323	48.38158	9.236082	8.69E-05
AKR1C2	0.240107	2.220379	9.247449	9.95E-05
SLFN13	0.001617	0.014979	9.265941	0.061987
PTPRE	0.157435	1.469997	9.337153	7.88E-05
EPAS1	6.531951	61.00821	9.339969	1.10E-05
LPXN	1.179403	11.02246	9.345796	0.014099
LOC10192	0.604369	5.672648	9.386071	0.003185
TG	0.012744	0.119634	9.387108	0.123093
FBN1	6.977409	65.6472	9.408535	0.027061
TBX15	0.457469	4.305805	9.412232	0.000361
ZIC1	0.27303	2.574989	9.431155	8.73E-06
CACNG7	0.68657	6.480319	9.43869	0.00663
APOL1	0.494217	4.669826	9.448937	0.002281
TGFBI	220.9473	2089.082	9.455118	0.000122
FOXC2	1.143262	10.81881	9.463105	0.001314
EHBP1L1	4.273512	40.49605	9.476059	9.15E-07
IL33	0.068973	0.653848	9.479741	0.035354
ACTG2	0.096869	0.919421	9.491431	0.028141
JADE2	0.947748	9.012004	9.508861	4.18E-06
COL13A1	2.93011	27.87806	9.51434	6.86E-05
MLKL	0.359841	3.430512	9.533421	0.004376
TMEM71	0.09684	0.928729	9.590378	0.0039
GNAI2	26.91055	258.5324	9.607102	0.000524
PAMR1	4.089831	39.34127	9.619291	0.003839
POU2F3	0.022082	0.212699	9.632051	0.001537
AKNAD1	0.049925	0.482375	9.661925	0.000302
TGFB2	0.733325	7.087802	9.665296	0.029521
ETV2	0.083341	0.807015	9.683233	0.001073
AGBL2	0.017831	0.173017	9.703039	0.035975
APLNR	0.004743	0.046154	9.731321	0.156301
SORCS3	0.003187	0.03113	9.768477	0.074666
VWA5A	1.717754	16.79721	9.778585	0.001919
RGS3	0.945655	9.253028	9.784778	4.08E-05
MSRA	0.514412	5.039322	9.796279	5.80E-05
TREM1	0.339801	3.33213	9.806111	0.057377
TMEM140	0.13617	1.335937	9.810827	0.00015
SRD5A2	0.006202	0.061014	9.837177	0.209103
TCEAL3	11.60635	114.3138	9.849246	0.000127
PTGER4	0.150438	1.484572	9.868304	0.020836
ZP1	0.605794	5.98297	9.876239	0.007795
HIST1H3D	0.041147	0.408233	9.921339	0.039193
MYH15	0.12348	1.226018	9.928895	0.007679
NLRC5	0.439055	4.364004	9.939532	2.23E-05
MYD88	0.935801	9.329422	9.969447	5.87E-05
RAB32	7.458992	74.68744	10.01308	1.10E-07
LTBP1	7.738056	77.52104	10.01815	1.36E-05
KCNN4	1.890573	18.94824	10.02249	0.006132
CCDC149	0.455056	4.566812	10.03572	4.71E-06
CPLX3	0.009696	0.097708	10.07721	0.082549
CCDC96	0.173715	1.752577	10.08878	0.000439
ACOT4	0.011954	0.1206	10.089	0.005843
ADAMTS1	0.51736	5.225875	10.10104	0.000511

FBXO43	0.138831	1.402384	10.10137	0.000729
HSPB3	0.361486	3.654545	10.10977	0.004657
FCMR	0.128481	1.299549	10.1147	0.000104
IRS1	0.806927	8.178397	10.13524	7.30E-05
ARHGAP3	0.647383	6.590537	10.18027	0.000211
CYBA	6.915065	70.46889	10.19063	4.09E-06
TGFBR3L	0.163596	1.67125	10.21568	5.56E-05
TMEM270	0.098174	1.008616	10.27376	0.000732
SMPD1	6.508105	66.88686	10.27747	2.94E-05
FAM129B	19.76658	203.6452	10.3025	1.14E-06
PDE6G	0.038828	0.40097	10.32683	0.004785
CARS2	5.068509	52.37873	10.33415	6.55E-06
CCDC87	0.026001	0.268827	10.33914	0.041779
ARPC4	8.530714	88.51845	10.37644	4.67E-05
TM4SF19	0.284941	2.956786	10.37684	0.001123
CGB5	0.086373	0.89812	10.39812	0.327549
C1QTNF2	0.113845	1.185585	10.414	0.001362
TCIM	2.01672	21.04654	10.43602	0.002915
GRIA3	0.108288	1.131031	10.44466	0.00035
REP15	0.071348	0.747754	10.48041	0.004751
LIPE	0.135919	1.426336	10.494	0.000224
TIMP1	88.1815	927.1557	10.51417	0.096035
ZNF785	0.157614	1.660236	10.53355	0.003133
ETV1	1.260961	13.32833	10.56998	9.41E-05
ICAM3	0.81841	8.652203	10.57197	0.000143
PCDHB6	0.043924	0.465059	10.58772	0.001568
CD82	14.8023	157.041	10.60923	0.001261
PARP10	0.634992	6.745008	10.6222	0.00025
TMEM273	0.450037	4.801155	10.66837	1.75E-06
PTGER2	0.521594	5.568073	10.67511	6.37E-05
ENG	1.856801	19.8521	10.69156	0.00027
LY6D	0.024325	0.261741	10.76035	0.302991
LGALS8	0.481842	5.214421	10.82186	0.000159
ADCY8	0.705397	7.698407	10.91359	0.005407
DNAH5	0.020701	0.226455	10.93937	0.000303
EREG	2.643784	28.93696	10.94528	0.03291
PCDHB12	0.11283	1.239605	10.98651	0.006509
TMSB4X	234.8995	2585.191	11.00552	0.008977
WNT5A	1.731791	19.07176	11.01274	0.000102
DGKI	0.053712	0.593039	11.04099	0.000293
KCNU1	0.005103	0.056341	11.04103	0.256074
ARTN	1.061427	11.72155	11.04321	0.018642
EPM2AIP1	0.349517	3.860785	11.04606	3.66E-05
C11orf91	0.106095	1.175059	11.07556	0.000256
AR	0.101701	1.128129	11.09258	3.86E-06
ID1	19.29739	214.7817	11.13009	0.132664
SYNPO	2.000849	22.2748	11.13267	0.023859
ANGPTL4	3.300702	36.78799	11.1455	0.00025
PCYT2	0.764292	8.543466	11.17827	0.001999
BEND5	0.105557	1.180623	11.18465	0.016933
MYL9	6.322999	71.08503	11.2423	0.020116
MRVI1	0.01463	0.164605	11.25084	0.016285
FAM180A	0.00941	0.105973	11.2612	0.017822
CD33	0.61655	6.948172	11.26944	4.41E-05
AKR1B1	149.4237	1686.533	11.28692	0.002425
XYLT1	1.625616	18.34996	11.28801	2.27E-05
ADRA2A	0.03882	0.43885	11.30483	0.017057
FAM20C	7.087524	80.18179	11.31309	9.59E-06

TRPV2	0.229158	2.599639	11.3443	0.000701
SLCO3A1	0.277469	3.165138	11.40716	8.28E-06
CD68	16.3929	187.8472	11.45906	5.59E-06
ZNF804A	0.168901	1.942529	11.501	0.000879
LOC10537	0.174543	2.024861	11.6009	0.105419
ZNF474	0.018725	0.218289	11.6576	0.0061
DPY19L2	0.03213	0.375187	11.67733	0.000225
IFIT3	0.316222	3.697485	11.69269	0.00417
UBA7	0.665262	7.797961	11.72164	0.002443
CH25H	0.19329	2.267514	11.73116	0.008628
FRMD3	0.148736	1.746556	11.74268	2.10E-05
SYNDIG1	0.242279	2.847541	11.75316	0.006912
FILIP1L	0.45658	5.394043	11.81402	0.0001
ARMCX2	1.711827	20.2247	11.81469	7.69E-07
HPCAL1	5.442197	64.36709	11.82741	0.000287
CCND1	12.20589	144.5119	11.83952	0.002678
KCNS1	0.080371	0.953632	11.86542	0.050341
FRMPD4	0.012578	0.149277	11.86839	0.004871
STS	0.125267	1.487858	11.87748	0.000417
IL11	2.55564	30.42746	11.906	0.081853
FBXL13	0.152466	1.819206	11.93187	0.000144
CDH13	5.763417	68.86105	11.94795	7.33E-06
IGFBP6	32.31762	386.1804	11.94953	0.000159
COPZ2	2.790782	33.38213	11.96157	3.87E-06
S100A2	1.708258	20.44159	11.96634	0.000397
MGST1	7.597855	91.33248	12.02082	5.04E-06
ECM2	0.011323	0.13631	12.03884	0.023788
FBXL7	0.240331	2.894167	12.04242	0.000388
MILR1	0.468279	5.643487	12.05155	0.000325
CLMP	1.873915	22.58777	12.05379	1.57E-05
ZNF365	0.113642	1.372363	12.07623	0.000321
ITGA3	26.42595	319.1674	12.0778	0.005759
SVEP1	0.323703	3.925683	12.12743	2.35E-06
PLAUR	7.353486	89.322	12.14689	0.00384
IFI44	1.066329	12.95781	12.1518	1.54E-05
DUSP23	1.808538	21.98743	12.15757	9.42E-08
KIFC3	1.779133	21.83828	12.27468	3.56E-06
GRAP	0.027021	0.332949	12.32197	0.309877
SP100	0.702853	8.674736	12.34218	1.69E-05
COLEC10	0.015203	0.187651	12.34275	0.001927
RGS20	1.067658	13.21477	12.37734	0.001637
TUBB6	15.82258	196.1935	12.39959	0.000525
HRH1	0.516696	6.415902	12.41717	0.000611
NTRK3	0.006582	0.081866	12.43772	0.000858
HIST1H2B	0.086532	1.078432	12.46284	0.010577
KDR	0.104939	1.308358	12.46782	0.018488
EMP1	9.428502	117.9126	12.50597	5.14E-05
SRGN	1.751202	21.91063	12.51176	2.27E-05
NNMT	0.920695	11.53134	12.5246	0.005109
GOLGA8S	0.003336	0.041824	12.53544	0.05632
CCDC188	0.12148	1.52486	12.55234	0.000526
CCL3	0.427835	5.375614	12.56468	0.000118
FANCF	0.431228	5.419508	12.56761	0.00015
PANX3	0.012193	0.153553	12.59386	0.011691
ZNF575	0.137691	1.734673	12.59831	0.001324
FJX1	3.232467	40.75406	12.60773	0.015444
MT3	0.154672	1.956256	12.64773	0.03312
OAS2	0.064914	0.821935	12.66189	0.000334

ABCC3	0.353245	4.483955	12.6936	5.98E-05
TNFSF14	0.02008	0.258389	12.86805	0.094725
MICAL2	2.396364	30.90679	12.89737	0.000246
FAM107A	0.012705	0.16409	12.91509	0.018765
CLCA2	0.008536	0.110457	12.94047	2.21E-05
RAB20	2.034908	26.34018	12.94417	3.38E-05
PSCA	0.25927	3.368817	12.99347	0.003835
GABRQ	0.20005	2.610533	13.0494	0.000828
TM4SF1	11.70604	152.9377	13.06485	0.002945
NPR2	0.420955	5.504307	13.07575	6.96E-05
ZKSCAN7	0.028455	0.37317	13.11447	0.000328
IFIT2	0.081274	1.070307	13.16911	0.000254
TSPAN1	0.20575	2.713289	13.18733	0.009445
HIST1H1D	0.071552	0.944768	13.20398	0.046023
PSMB9	2.620345	34.67232	13.23197	0.000634
PARP12	0.430551	5.702118	13.24378	1.22E-05
BEST3	0.01272	0.170035	13.36726	0.016039
MVP	11.75287	157.6615	13.41472	0.000172
LHB	0.243685	3.270805	13.42227	0.136289
MAP1LC3/	1.789853	24.24903	13.54806	8.00E-05
LRRC74B	0.012265	0.167313	13.64146	0.022602
TMEM154	0.14943	2.039916	13.65136	0.000119
FERMT3	0.329797	4.506235	13.66366	0.001284
TNFAIP8L:	0.548984	7.505091	13.67086	3.97E-05
NAGS	0.350905	4.820525	13.73742	9.23E-05
SPOCK1	3.335877	45.93401	13.76969	8.64E-05
PAX8	0.285244	3.930058	13.77787	0.00271
TGFBR2	2.048568	28.31319	13.82097	0.000291
SHC3	0.143162	1.980404	13.83335	0.000322
FAIM2	0.473468	6.567746	13.87157	8.07E-06
PCDHGC5	0.043234	0.600518	13.88996	1.74E-05
HSPA2	0.629485	8.750474	13.90101	7.75E-05
ACSM5	0.048452	0.676825	13.9689	0.000459
SERPINE1	35.39731	494.9396	13.98241	0.000128
PLPP4	0.125855	1.766501	14.03603	0.012604
DDO	0.17881	2.513083	14.05451	0.000426
ARMCX1	0.832222	11.70291	14.06226	0.001584
TCN2	0.280353	3.960579	14.12709	0.001207
SLX1B	0.498	7.066646	14.19005	0.332351
CNTNAP5	0.001742	0.024721	14.19367	0.09402
LOXL1	5.936567	84.3518	14.20885	0.010687
NTF3	0.01114	0.158645	14.24098	0.132429
CASP4	1.894949	27.01017	14.25377	0.000515
MYOF	1.889701	26.94004	14.25624	0.001609
APCDD1L	1.287293	18.3602	14.26264	6.64E-06
GALNT15	0.013849	0.198419	14.32748	0.005546
C6orf132	0.002727	0.039161	14.35831	0.008017
PCDHGA2	0.019246	0.277453	14.41631	8.08E-06
KCNJ2	0.070535	1.018298	14.43679	6.83E-06
ERAP2	1.097093	15.9785	14.5644	2.96E-05
CATSPER1	0.13115	1.914903	14.60085	0.001125
MUC12	0.002335	0.034168	14.63211	0.010711
POU2F2	0.385426	5.643668	14.64266	0.000484
CTSS	1.184798	17.43028	14.71161	0.007807
PLEKHN1	0.156492	2.309497	14.75793	0.003063
CCDC9B	1.13542	16.76232	14.76309	7.24E-07
DOCK10	0.262905	3.883514	14.77156	6.37E-05
CD6	0.05191	0.772105	14.87383	0.002558

LAT2	0.15743	2.34635	14.90406	0.000117
IGFBP4	6.350537	94.87818	14.94018	0.000712
MAP10	0.013689	0.204749	14.95742	0.000207
EVI2A	0.120824	1.825391	15.1079	0.001556
PSMB8	2.269391	34.50471	15.20439	3.32E-06
MAATS1	0.067124	1.022078	15.2268	0.000339
IGFL3	0.027604	0.420618	15.23749	0.029262
ADTRP	0.074267	1.133712	15.26526	0.001388
KRT79	0.768405	11.73461	15.27138	0.079059
LRRC15	1.585007	24.23782	15.29193	4.73E-06
NMNAT2	0.834407	12.80643	15.34793	0.00405
MAP1A	2.306934	35.5514	15.41067	0.000261
EVA1C	0.60925	9.39309	15.41746	0.000175
STEAP1B	0.99004	15.36876	15.52338	9.63E-05
ADAM12	0.543616	8.453431	15.55036	0.000803
LOC10192	0.009755	0.151999	15.58174	0.005886
SPP1	1.342188	20.93128	15.59489	0.028889
PTPN22	0.071223	1.113206	15.62978	0.00029
TRIM22	0.882463	13.8088	15.64803	9.35E-05
RAB42	0.033393	0.526085	15.75427	0.000376
RTN1	0.35444	5.600375	15.80064	6.58E-06
CD177	0.00653	0.103945	15.91821	0.001439
SPDYA	0.009044	0.143999	15.92178	0.014343
NALCN	0.036337	0.580088	15.9642	0.020948
B3GNT9	1.797539	28.75557	15.99718	3.13E-05
KIAA0825	0.00384	0.061674	16.0607	0.001788
ZNF671	0.063429	1.021418	16.10336	0.002467
PTGES	5.364792	86.45348	16.11497	0.213119
SH2B3	5.087853	82.004	16.1176	0.015179
FGD3	0.191869	3.095552	16.13366	0.18987
SPINK1	0.146071	2.361424	16.16626	0.043518
NGF	0.126674	2.04856	16.17193	0.002771
FAM196A	0.029388	0.475977	16.19643	0.005801
HTR7	0.137997	2.244459	16.26455	0.018644
APOL3	0.0726	1.184451	16.31478	0.000856
PTPRN2	0.166084	2.711468	16.32585	7.79E-06
ABLIM3	1.027408	16.85881	16.40908	1.63E-05
SAMD9	0.196361	3.224899	16.42332	6.73E-05
HOMER3	6.26667	103.0004	16.43622	0.000113
CCDC198	0.009634	0.158364	16.43846	0.000286
SP5	0.012185	0.201649	16.54856	0.040239
NAALADL	0.009521	0.1577	16.56253	0.00886
TLR3	0.037456	0.62269	16.62443	0.000729
C2CD2	0.540946	9.005035	16.64681	3.66E-05
ELOVL3	0.081398	1.362329	16.73663	0.000349
MLH1	1.351068	22.71748	16.81446	5.01E-06
PTGS2	1.436699	24.45498	17.02164	0.293837
P4HTM	1.09287	18.61462	17.03279	8.55E-05
NKX3-1	0.524338	9.016286	17.19555	0.000561
PLP2	18.05202	310.748	17.21403	2.56E-06
ARHGDIB	0.1279	2.212129	17.29583	0.001239
TIMP4	0.698419	12.13823	17.37959	0.000831
SRPX	9.908308	172.6124	17.42098	1.76E-06
IL31RA	0.230534	4.096952	17.77161	4.01E-06
MAGEA1	0.486078	8.651648	17.79888	3.24E-06
LOC10272	0.01816	0.323642	17.82171	0.002728
ARSD	0.186633	3.367268	18.04216	2.15E-06
GSDMD	0.519853	9.396046	18.07444	0.00043

MGLL	2.00706	36.42158	18.14673	1.19E-05
MCTP2	0.214615	3.896668	18.15658	0.01154
PTGR1	2.637594	48.40834	18.35322	3.22E-06
PSG4	0.037151	0.68545	18.4504	0.03242
HRCT1	0.137579	2.550768	18.54043	0.002031
CSRP1	4.891912	91.40903	18.68575	0.000174
PCDHB13	0.152145	2.847904	18.71836	7.56E-07
ZNF385D	0.090456	1.700903	18.80359	0.000819
IGFBP3	10.52857	198.3208	18.83645	0.017548
ADAMTSL	0.104058	1.961445	18.84948	0.010101
TVP23A	0.014917	0.281178	18.84967	0.007774
PLA2G3	0.006043	0.114052	18.87418	0.048465
EVA1A	1.374245	26.1302	19.01423	1.28E-05
RARRES3	0.59198	11.28068	19.05584	0.008987
IL1RL1	0.01134	0.217184	19.15233	0.126763
RFX8	0.159399	3.069803	19.25856	0.00118
KCNMA1	0.207759	4.011407	19.30802	0.004504
CLCF1	0.711379	13.75803	19.33994	0.002383
TDO2	0.591546	11.45072	19.35728	0.229936
PPP2R2B	0.114796	2.234022	19.46074	0.039987
GABRA3	0.458618	8.934573	19.48153	0.000864
METTL7B	0.881892	17.20561	19.50988	6.45E-05
COL17A1	0.026575	0.51988	19.56246	0.003307
GBP1	0.33878	6.638487	19.59526	2.45E-06
B3GALNT1	0.101013	1.993152	19.73171	0.000179
NRG1	0.190088	3.753684	19.7471	4.17E-05
VASN	1.134617	22.42674	19.76592	0.00453
HS3ST1	0.001513	0.029954	19.79704	0.036694
CGB2	0.017501	0.348767	19.92811	0.336048
MYLK	1.114759	22.26737	19.97505	0.004954
ALDH3B1	0.997735	20.01436	20.0598	0.001362
CD44	14.93306	299.9951	20.08933	0.002829
CYTH4	0.192498	3.872753	20.11841	2.16E-06
SERPINA6	0.074987	1.51873	20.25333	0.000394
SEMA7A	5.060465	102.5616	20.26722	0.000319
RIN1	1.184599	24.05935	20.31013	0.000737
KLRC2	0.230661	4.684805	20.31032	0.071632
PCDHB14	0.185405	3.767911	20.32262	0.000243
CLCN4	0.158619	3.24264	20.44295	0.000315
COL10A1	0.005403	0.11062	20.47278	0.272362
MFAP4	0.48919	10.04305	20.52998	4.23E-06
APBB1IP	0.283518	5.836171	20.58483	0.00301
HMSD	0.058124	1.198365	20.61734	0.003298
CD163L1	0.32274	6.725475	20.8387	2.22E-06
CFAP52	0.017791	0.373017	20.96611	0.024883
NCR1	0.009066	0.190279	20.98769	0.089655
AHNAK2	0.598743	12.61979	21.07714	0.00959
MAP3K7C	0.150824	3.181361	21.09315	0.007219
THBD	0.262764	5.548708	21.11673	0.082088
CD274	0.162416	3.448948	21.23526	0.001013
SLAMF7	0.036272	0.772981	21.31068	0.001726
C19orf84	0.013951	0.29815	21.37053	0.046002
NOV	0.32049	6.928028	21.61695	0.001307
PSMD5	0.699449	15.13696	21.64127	2.10E-05
SLITRK2	0.006129	0.132847	21.67586	0.006527
WWC3	0.253662	5.516988	21.74933	1.14E-05
HFE	0.207454	4.517705	21.77685	0.000405
VAMP5	3.915284	85.38926	21.80921	0.000218

GNG3	0.073012	1.595903	21.85815	0.03766
MMP1	0.722957	15.87483	21.9582	0.138226
SAMD9L	0.095996	2.148516	22.38123	0.000336
LY96	0.60339	13.52318	22.41202	0.002841
CRACR2B	0.563239	12.6855	22.5224	0.001624
CAVIN1	8.143352	183.5702	22.54234	0.00069
TCEAL7	1.902303	43.01746	22.61335	4.63E-06
MT1F	3.172739	71.98672	22.68914	9.78E-06
TMEM255	0.289899	6.609375	22.79889	2.63E-05
PLXNA4	0.041808	0.955492	22.85442	0.000208
ADAM28	0.011859	0.271881	22.92601	0.000307
AKR1B10	0.15714	3.606179	22.94887	2.16E-05
TUBA4A	6.07621	140.0148	23.04311	0.00016
ACTL8	0.009287	0.216643	23.32789	0.025794
GRM1	0.002699	0.063074	23.36617	0.102818
ZIC4	0.015379	0.359629	23.38515	0.001094
ANXA1	32.5267	761.5503	23.41308	1.30E-06
SPOCD1	1.836379	43.03558	23.43502	7.12E-05
RARRES1	0.024944	0.584784	23.44394	0.008867
CEMIP	0.196518	4.611437	23.46569	0.251049
C10orf90	0.288303	6.779539	23.5153	3.25E-05
ACOX2	0.242908	5.792946	23.84831	0.000181
SQOR	2.663106	63.52462	23.85358	2.48E-05
ITGA2	1.546218	36.88883	23.85746	0.030268
STAC	0.853897	20.71725	24.262	0.00651
NAV3	0.038348	0.932466	24.3158	0.000137
FOSL1	10.00616	244.2815	24.41311	3.08E-06
ACTL10	0.055061	1.345122	24.42956	0.000362
CA9	11.52588	282.0619	24.47204	0.02441
CSMD2	0.201811	4.944462	24.50051	1.85E-06
ANKRD30	0.010819	0.265311	24.52334	0.000132
FAM212B	0.912405	22.5569	24.72246	0.010276
DGKG	0.170801	4.233024	24.78333	3.67E-06
CORO2B	0.889205	22.37374	25.16152	0.000265
ADGRG1	1.702676	42.94777	25.22369	3.88E-06
C16orf45	2.796386	70.79074	25.31508	0.001282
WFDC3	0.426956	10.87529	25.4717	0.01389
KRT32	0.00973	0.247913	25.47969	0.054829
ALDH1A3	9.136388	233.3543	25.54119	0.001165
SMIM3	3.724551	95.18224	25.55536	1.10E-07
CRYBG1	0.440925	11.30257	25.63378	0.001691
DRD1	0.004523	0.11771	26.02198	0.015837
LIF	3.049435	79.89196	26.19894	0.071703
MT1X	12.4726	327.0022	26.21765	8.99E-07
CGB8	0.29572	7.785202	26.32622	0.292519
TMEM200	0.044855	1.204142	26.8451	5.46E-07
ATP8B4	0.006356	0.170811	26.87398	0.001704
TGM2	5.999064	163.734	27.29326	2.31E-07
FDCSP	0.663845	18.2233	27.45115	0.003751
ITK	0.016746	0.463138	27.65663	0.001974
GPR39	0.242499	6.707944	27.66173	0.00662
PECAM1	0.015218	0.42226	27.74806	0.021335
P2RX6	0.279906	7.76869	27.75462	5.13E-06
ATOH8	0.209259	5.844709	27.93053	0.027327
AQP9	0.227716	6.398975	28.10072	0.021809
LOC10798	0.076931	2.171559	28.22725	4.12E-05
PPP1R36	0.011941	0.337757	28.2858	0.010068
LGALS1	188.213	5336.922	28.35575	0.003428

SH3BGRL3	31.41051	892.4421	28.41221	5.92E-06
XDH	0.006332	0.18074	28.54477	0.002682
MAGEC1	0.095736	2.736323	28.58209	4.67E-05
OASL	0.472625	13.5438	28.65653	3.48E-05
CARD16	0.154785	4.450743	28.75438	0.001273
MYOZ2	0.01675	0.481715	28.75877	0.014237
SLC22A4	0.288827	8.386472	29.0363	0.026339
ANXA8	0.028229	0.819813	29.04159	0.000371
S100A6	131.2099	3812.711	29.0581	0.000649
TAGLN3	0.047655	1.384947	29.06204	8.78E-05
SIRPB1	0.015322	0.446255	29.12577	0.000147
SYNC	0.606177	17.6866	29.17729	2.46E-05
LINGO2	0.004933	0.14588	29.57402	0.006213
PID1	0.133848	4.009177	29.95331	1.02E-06
B3GALT5	0.005522	0.166847	30.21543	0.032783
SERTM2	0.008097	0.247779	30.59966	0.090185
SLC6A7	0.003163	0.096917	30.64248	0.067616
BDKRB2	0.723235	22.50208	31.1131	0.002081
PLAC9	0.027515	0.858355	31.19562	0.001116
RRAD	1.623814	50.79029	31.2784	8.24E-06
DCBLD2	5.62378	175.9592	31.28843	6.65E-06
FOXL1	1.59962	50.74715	31.72451	0.00074
KLF17	0.013842	0.442539	31.97188	0.045091
PRR16	0.135575	4.339105	32.0052	0.00368
HSPB6	0.730531	23.41719	32.05504	0.000707
HAS1	1.993142	63.96696	32.09353	0.104954
SNAP25	0.429803	13.83028	32.17819	0.000228
CADPS	0.125542	4.065503	32.38371	1.45E-05
USP41	0.037484	1.215289	32.42155	0.023179
TENM2	0.01961	0.635886	32.42594	0.012357
SLC15A3	0.113363	3.730504	32.90768	0.000596
LOC10537	0.027177	0.899651	33.10296	0.015085
NLRP3	0.038428	1.282269	33.36773	3.48E-05
RIOX1	0.022981	0.769237	33.47338	0.020257
SGIP1	0.03037	1.021704	33.64151	9.94E-06
ITGA11	0.257224	8.76295	34.0674	0.001704
LTF	0.005378	0.183494	34.11819	0.007621
CNGA3	0.020064	0.686439	34.21294	0.000436
ADGRF4	0.278602	9.550891	34.28152	4.73E-06
RAB27B	0.38515	13.26337	34.43688	0.002622
CAV1	9.685981	333.8093	34.46313	0.008328
SLC43A3	2.941596	102.9529	34.99899	9.21E-05
SLC24A2	0.028458	0.999819	35.13272	0.000497
FAM111A	0.492244	17.39904	35.34637	0.000314
PLPPR4	0.222761	7.889883	35.41854	0.006125
PADI4	0.012573	0.446007	35.47217	0.010534
MYPN	0.043902	1.579512	35.97823	0.000361
P2RY6	0.002634	0.096494	36.63901	0.00063
AXL	3.400078	125.4043	36.88277	0.007731
TMEM132	0.026894	0.994	36.95958	0.004436
ARL14EPL	0.011976	0.448254	37.43052	0.180874
FAM155A	0.144135	5.434609	37.705	0.000231
S100A8	0.107484	4.082965	37.98682	0.192652
WISP1	0.192472	7.373873	38.31149	0.003352
KYNU	0.480225	18.55417	38.63642	0.00372
TNFRSF1B	0.430818	16.65616	38.66169	0.000182
CEND1	0.621685	24.21903	38.9571	9.85E-05
DNAJC15	0.481121	18.95807	39.40396	2.15E-07

KY	0.023203	0.914667	39.42025	9.76E-05
S100A9	0.298019	11.80446	39.60977	0.274575
IL6	0.206613	8.185642	39.61828	0.09154
DSEL	0.207665	8.388963	40.39665	0.000674
CSF2	0.119648	4.86507	40.66149	0.000218
TMEM158	32.43843	1322.354	40.76504	0.00012
DNER	2.460905	100.3251	40.76756	0.005292
GALNT9	0.730792	29.86347	40.86453	0.05073
ACSL5	0.015332	0.626666	40.87192	0.000149
JPH2	0.022915	0.940593	41.04685	6.44E-05
S100A16	6.269777	259.3158	41.35965	0.000401
BDNF	0.198501	8.279278	41.70891	1.98E-05
CCR1	0.310832	12.99863	41.81879	0.003961
STC1	4.07118	171.1828	42.04747	7.97E-05
THBS1	0.892321	37.76916	42.32687	1.53E-05
TSGA10IP	0.014817	0.633661	42.76553	0.005867
CACNA1A	0.197398	8.626028	43.69867	3.00E-06
HCAR3	0.009129	0.400328	43.85317	0.261732
DCC	0.032238	1.414735	43.88362	0.003294
KRT80	0.142641	6.384299	44.75773	4.91E-05
SFTPB	0.005083	0.239876	47.19254	0.265334
PLAU	8.05847	381.751	47.37264	0.091513
RAC2	6.604763	313.3313	47.44021	2.08E-06
NT5E	5.932212	281.7289	47.49137	0.002729
LRRN3	0.135626	6.489347	47.84739	0.002841
SLC16A6	0.451691	21.63559	47.89912	0.112277
BLK	0.272846	13.08386	47.95332	3.14E-07
TMEM156	0.033109	1.594555	48.16062	0.010071
PALMD	1.346597	65.79365	48.85918	0.000195
CDCP1	0.02715	1.327308	48.88825	0.014873
SERPINB8	0.235765	11.54202	48.95565	0.014767
NDN	0.130146	6.396565	49.1493	1.56E-06
PINLYP	0.118957	5.859523	49.25741	0.100808
APLN	0.503114	24.78518	49.26353	0.045571
SCG5	2.588426	131.4771	50.79421	8.78E-05
DMBT1	0.002265	0.115259	50.87674	0.05179
NEGR1	0.024127	1.23032	50.99353	2.57E-05
CSF1R	0.052567	2.681149	51.00468	0.003721
SERPINA9	0.011351	0.582961	51.35938	0.262877
BHMT2	0.212445	10.95329	51.55814	0.000663
MMP3	2.925421	151.5217	51.79485	0.22163
KRT78	0.025247	1.318455	52.22145	0.032658
EVC2	0.017492	0.913721	52.23794	3.64E-05
WNT7B	1.15587	60.7283	52.53902	0.003063
ITGBL1	0.129949	6.865538	52.83268	0.029142
PRLR	0.004337	0.229436	52.90414	0.00745
IL36RN	0.196188	10.72501	54.66711	0.147573
SLC26A8	0.005156	0.285099	55.29167	0.024846
KCNJ15	0.016202	0.899806	55.5384	0.023171
NME5	0.043001	2.436653	56.66521	3.55E-05
NTNG1	0.052961	3.011728	56.86709	4.31E-05
MRPS24	0.02673	1.559909	58.35865	0.000336
OCIAD2	1.365167	79.71872	58.39485	3.18E-06
LOC10798	0.003937	0.231122	58.70963	0.001262
SLC16A3	2.911547	171.3483	58.85131	0.00311
FAM84A	0.017053	1.007074	59.05459	0.017189
TNFRSF11	0.088295	5.238385	59.32826	0.006714
DAW1	0.017735	1.058385	59.67837	0.082186

ADAMTS5	0.039961	2.391452	59.84413	0.061804
CPNE4	0.002024	0.125162	61.82531	0.001379
NFE4	0.057792	3.593435	62.17893	0.000138
FGF1	0.028037	1.766731	63.01461	0.010925
ZNF132	0.006436	0.40641	63.14391	0.000249
HKDC1	0.053265	3.365242	63.17951	5.81E-05
CLEC2B	0.065463	4.167156	63.65691	0.000127
VEPH1	0.092222	5.952886	64.54925	1.15E-07
MT2A	48.5277	3132.547	64.55172	0.000283
HCAR2	0.008905	0.581359	65.28743	0.139089
CPA4	0.924805	61.04883	66.01263	0.064269
GDF5	0.122865	8.162678	66.43628	0.000486
KIRREL3	0.004216	0.282526	67.0098	0.038445
NAP1L5	0.097288	6.593431	67.77208	2.30E-05
NLRP11	0.018201	1.264458	69.47019	0.000148
4-Mar	0.029826	2.116153	70.95058	0.001066
SLA	0.005084	0.362706	71.34584	0.000634
KCNQ3	0.003805	0.277822	73.0183	1.31E-05
GLIPR1	0.904238	66.62416	73.67988	8.23E-05
SERPINB2	0.512911	38.04742	74.17936	0.172856
ALK	0.022527	1.681312	74.63534	0.000262
NXF3	0.010289	0.769613	74.79821	0.00211
TMEM233	0.146346	11.04202	75.45147	1.50E-05
PRSS3	0.171034	13.0207	76.12913	1.39E-07
CSF3	6.783806	519.0526	76.51348	0.107183
LOC10537	0.027566	2.181176	79.12588	0.000515
IL1B	8.875454	746.1362	84.06738	0.186349
TFPI2	12.17842	1027.706	84.38747	0.112926
S100A3	0.580255	49.74287	85.72594	2.53E-06
PDCD1LG2	0.091765	7.868161	85.74208	0.001775
CASP1	0.053736	4.763678	88.64989	0.001273
BEX1	0.692121	61.43848	88.76843	0.000281
GRIN2A	0.004484	0.402715	89.82041	3.01E-05
CAVIN3	2.015798	186.0312	92.28663	0.000634
GNG11	7.342567	679.0552	92.48199	0.00442
ELF5	0.015129	1.399271	92.49194	0.001241
CCRL2	0.027617	2.714229	98.27953	0.000487
PSG9	0.004569	0.463212	101.3876	0.02502
IL7R	0.040327	4.46457	110.7094	3.59E-05
KRT34	0.049602	5.63271	113.5572	0.000414
VAT1L	0.536685	61.00792	113.6755	0.001209
CCDC152	0.037698	4.38006	116.1877	6.76E-06
C1orf94	0.138592	16.13767	116.4397	0.00015
GABRG3	0.015751	1.884446	119.6366	0.001544
CTNND2	0.002878	0.351535	122.1614	0.070605
CDH4	0.09727	12.04729	123.8536	0.001158
FPR1	0.485047	60.34798	124.4167	0.007785
C10orf55	0.007317	0.917862	125.4397	0.009626
SERPINB7	0.13303	17.06834	128.3045	0.000132
FAM111B	0.063466	8.326889	131.2033	0.000187
GPR68	0.082079	11.14645	135.8021	0.036119
ANKRD35	0.033396	4.734213	141.7586	2.34E-05
TLX2	0.03522	4.997385	141.8921	1.46E-05
GSTM1	0.107209	16.23986	151.4788	0.000265
KRT15	0.019368	3.058934	157.9357	0.000119
DOCK2	0.006989	1.190043	170.2682	0.000296
SLC35F4	0.002884	0.491607	170.4414	9.27E-05
AOX1	0.058162	10.05783	172.9279	0.004698

SPTLC3	0.058004	10.47451	180.5836	0.003981
FAM196B	0.031195	5.836727	187.1039	0.001585
SIGLEC15	0.453907	92.73288	204.2991	0.000568
BDKRB1	0.320383	68.36394	213.3819	0.018794
LOC3896C	0.006828	1.741696	255.0785	0.067255
RAET1E	0.003742	1.056543	282.3291	0.001074
MT1E	0.707001	199.9316	282.7881	0.001531
GREM1	0.399411	116.6436	292.0393	2.61E-05
KRTAP1-5	0.064395	19.43158	301.7572	5.32E-05
EVI2B	0.008838	2.852309	322.7346	0.038809
ACP7	0.028666	9.43021	328.9677	0.015692
DIO2	0.010472	3.48452	332.7505	0.112886
ANXA8L1	0.008811	4.557484	517.2297	0.014421
NDP	0.085484	60.85263	711.8621	0.005917
MEST	0.052149	38.71269	742.3495	0.070936
SERPIND1	0.016753	13.31176	794.6042	0.230708
KCTD4	0.026033	21.84865	839.2654	0.000264
FGF7	0.004956	6.598825	1331.381	0.013057
MPP4	0.004844	7.529129	1554.201	0.004709
IGFBP1	0.024523	46.30794	1888.323	1.52E-05
